# Studying the functional connectivity of the primary motor cortex with the binarized cross recurrence plot: The influence of Parkinson’s disease

**DOI:** 10.1371/journal.pone.0252565

**Published:** 2021-06-07

**Authors:** Clara Rodriguez-Sabate, Manuel Rodriguez, Ingrid Morales

**Affiliations:** 1 Laboratory of Neurobiology and Experimental Neurology, Department of Physiology, Faculty of Medicine, University of La Laguna, Tenerife, Canary Islands, Spain; 2 Centro de Investigación Biomédica en Red sobre Enfermedades Neurodegenerativas (CIBERNED), Madrid, Spain; 3 Department of Psychiatry, Getafe University Hospital, Madrid, Spain; Universidade do Estado do Rio de Janeiro, BRAZIL

## Abstract

Two new recurrence plot methods (the binary recurrence plot and binary cross recurrence plot) were introduced here to study the long-term dynamic of the primary motor cortex and its interaction with the primary somatosensory cortex, the anterior motor thalamus of the basal ganglia motor loop and the precuneous nucleus of the default mode network. These recurrence plot methods: 1. identify short-term transient interactions; 2. identify long-lasting delayed interactions that are common in complex systems; 3. work with non-stationary blood oxygen level dependent (BOLD) data; 4. may study the relationship of centers with non-linear functional interactions; 5 may compare different experimental groups performing different tasks. These methods were applied to BOLD time-series obtained in 20 control subjects and 20 Parkinson´s patients during the execution of motor activity and body posture tasks (task-block design). The binary recurrence plot showed the task-block BOLD response normally observed in the primary motor cortex with functional magnetic resonance imaging methods, but also shorter and longer BOLD-fluctuations than the task-block and which provided information about the long-term dynamic of this center. The binary cross recurrence plot showed short-lasting and long-lasting functional interactions between the primary motor cortex and the primary somatosensory cortex, anterior motor thalamus and precuneous nucleus, interactions which changed with the resting and motor tasks. Most of the interactions found in healthy controls were disrupted in Parkinson’s patients, and may be at the basis of some of the motor disorders and side-effects of dopaminergic drugs commonly observed in these patients.

## Introduction

The primary motor cortex (M1) plays a key role in the control of body posture and the planning (proactive control) and execution (online control) of movements [1]. The M1 uses the motor information provided by the basal ganglia (BG) through the anterior motor thalamus (MT) [2–4] and the proprioceptive sensitivity coming from the primary somatosensory cortex (S1) to perform these functions [5, 6]. The motor control works in parallel with other brain tasks which are normally running at the same time, including those performed by the default mode network (DMN) [7, 8]. The DMN is activated when the other brain tasks do not require all the cognitive resources, and a part of the mental activity can be used by the DMN to solve pending problems or to analyze previous events [8–10]. The DMN is made up of different cortical regions which are coordinated by two integrative hubs, the medial prefrontal cortex and the precuneous nucleus (PCn). The PCn sends projections to the M1 and may be particularly relevant for integrating the M1 and DMN activities [7]. The aim of the present work was to study the functional connectivity of the M1 with the S1, MT and PCn, paying particular attention to these interactions in the Parkinson’s disease (PD) patients, where they could be involved in motor and posture disorders [11, 12].

Motor and non-motor mental functions present fast fluctuations whose study needs methods able to detect brain oscillations of a few seconds. EEG based methods have a suitable time-resolution but their space-resolution is not enough to unequivocally identify some centers, including the PCn which is located away from and perpendicular to the scalp and the MT which is located deep in the brain. Functional magnetic resonance imaging (fMRI) and functional connectivity magnetic resonance imaging (fcMRI) have a suitable space-resolution but their time-resolution is limited, a weakness that was compensated for here by using a new method to process the blood-oxygen-level-dependent (BOLD) signal. fMRI assumes that the brain centers involved in a task are those which increase the average BOLD-level when the task is repeatedly performed (task-block design). The time-resolution of this method is the time spent on repeating the same task (task-block), which is often more than 20 sec. fcMRI assumes that the brain centers of neural networks are those showing BOLD co-fluctuations (identified by correlation, independent component analysis or other methods) during a time interval that is often greater than 60 sec. These methods generally assume a linear and stable (stationary) functional interaction of centers during this long-time interval, a circumstance which probably does not occur for the M1 interactions [13]. The authors have recently introduced a fcMRI method (Multiple Covariance method) which does not need the accumulation of BOLD-data, and whose time-resolution is just the time elapsed between two successive BOLD-recordings of the same brain area. However, this method does not provide information about the complex long-term dynamic of networks. The analytical procedures introduced here are based on the recurrence plot method and can be used to study the long-term dynamic of both the activity of individual brain areas and the functional connection between two brain areas.

The recurrence plot (RP) is a topological method introduced by Eckmann at the end of the 1980s to visualize, in two dimensions, the long-term dynamic of complex systems with multiple dimensions [14–22]. The Cross Recurrence Plot (CRP) is an extension of the RP which studies the long-term interactions of two systems [23–26]. Although the RP and CRP may be used to analyze the BOLD-dynamic of individual subjects, the lack of well-defined procedures to group individual data into experimental groups and to compare the resulting experimental groups have limited their use in MRI studies. An adaptation of recurrence plot methods which facilitates the grouping of fMRI and fcMRI data was used here to study the long-term dynamic of the M1 (Binarized Recurrence Plot; bRP), and the long-term functional interaction of the M1 with the S1, MT and PCn (Binarized Cross Recurrence Plot; bCRP). M1 activity is involved in the motor disorders which appear in PD patients when they are practicing voluntary motor actions (e.g., brady/hypokinesia) or when they remain at rest (e.g., resting tremor, hypertonia, body posture abnormalities) [11, 13, 27]. Thus, M1 connectivity was studied also in PD patients which were at rest or performing a voluntary motor task.

## Materials and methods

### Participants

Twenty right-handed volunteers with no history of neurological or mental diseases (control group; 10 males and 10 females; 37–72 years of age) and twenty right-handed PD patients (10 males and 10 females between; 36–75 years of age) were included in the study. Patients were diagnosed with idiopathic PD by two experienced neurologists who used the medical history, physical and neurological examinations, response to levodopa, laboratory examinations and MRI. Patients with a history of head injury, stroke and other neurological (post-encephalitic parkinsonism, Shy-Drager syndrome, drug- or toxin-induced parkinsonism, etc.) or psychiatric diseases (such as drug abuse, depression or dementia) were not included in the study. The mental status of PD patients was tested with the Montreal Cognitive Assessment and Mini Mental State Examination. The severity of motor disorders was assessed with the Hoehn & Yahr, the Schwab and England and the Unified Parkinson’s Disease Rating Scale III (UPDRS III) scales, which were used to select patients with a short evolution of PD (< 2 years) and a slight to moderate motor alteration during the “on” medication state (Table 1). In order to reduce the impact of medication on the studies, anti-parkinson drugs were not administered within 24 hours prior to the study. Written informed consent was provided by all participants, all procedures were in accordance with the ethical standards of the 1964 Helsinki declaration, and the study was approved by an institutional review board (Institutional Human Studies Committee of La Laguna University).

**Table 1:** The mental status (Montreal Cognitive Assessment and Mini Mental State test) and severity of motor disorders (Hoehn & Yahr, the Schwab and England and the Unified Parkinson’s Disease Rating Scale III -UPDRS II- scales) of patients included in this study.

Age (years of age)	61.3 ± 7.9
PD progression (months)	19.4 ± 6.6
Minimental test (max 35)	34.2 ± 0.7
Montreal Cognitive Assessment (max 30)	29.2 ± 0.6
Schwap & England scale (max 100)	93.2 ± 5.1
Hoehn & Yahr scale (max 5)	1.7 ± 0.17
UPDRS III (max 159)	12.2 ± 1.57

All tests were performed during the “on” medication state.

### Data collection

The basic experimental procedures were similar to those previously reported [28, 29]. In order to prevent involuntary head movements during the MRI studies, the head was attached to the head-coil of the MRI equipment. BOLD contrast images (64x64 sampling matrix with brain slices 4-mm thick and 4x4 mm voxels in-plane resolution) were acquired (General Electric Medical System 3.0 T) in a coronal plane (250 x 250 mm field of view) with gradient-echo (echo-planar imaging with repetition time 1600 ms; echo time 21.6 msec; flip angle 90°). These images were co-registered with 3D anatomical images (1x1x1 mm voxel resolution; repetition time 7.6 ms; echo time 1.6 ms; flip angle 12°; 250 x 250 mm field of view; 256x256 sampling matrix). The functional and anatomical studies were always obtained in a single session and with the head fixed in the same position of the field-of-view to facilitate a stable relationship between each fcMRI voxel (4x4x4 mm^3^) with the corresponding 64 structural voxels (1x1x1 mm^3^) during the whole study. A representative region of interest (ROI) of each brain region was located on a subject-by-subject basis by considering: 1. the Talairach coordinates, 2. the shape of the nucleus, and 3. the anatomical relationship of the nucleus with neighboring structures. The M1 [30], S1 [13], MT [28] and PCm [31] were identified in coronal slices located 4–27 mm posterior to the anterior commissure and according to previously reported procedures. All data sets were normalized to the Talairach space (Table 2 shows the position and size of ROIs). The data is available to those researchers who request it and who agree to sing a data sharing agreement.

**Table 2:** Coordinates (Talairach) are shown in mm.

	X	Y	Z	Size
**Primary somatosensory cortex**	32.2±4.7	-21.2±4.8	48.6±4.72	35.1±7.2
**Primary motor cortex**	38.6±4.8	-17.6±4.3	46.3±6.0	35.9±12.4
**Putamen**	24.1±1.0	-5.1±1.2	0.1±0.5	22.8±3.6
**External pallidum**	12.4±4.1	-2.3±0.9	2.3±1.3	9.8±2.4
**Internal pallidum**	13.6±1.4	-6.0±1.6	-1.1±1.1	11.7±1.3
**Subthalamic nucleus**	9.8±1.1	-12.4±2.5	-4.4±2.2	2.5±0.6
**Substantia nigra**	7.4±1.4	-18.4±1.9	-8.1±2.2	46.1±8.2
**Ventral-anterior thalamus**	9.1±1.7	-9.5±1.3	7.0±2.4	26.7±8.5

The size of the ROIs is shown by the number of their voxels.

### Data preprocessing

The data preprocessing included a slice scan time correction, a 3D motion correction, and a time filtering which eliminates frequencies below 0.009 Hz. Studies with images showing a translation >0.5mm or a rotation >0.5degrees were removed. No spatial smoothing was performed. Residual motion artifacts and physiological signals unrelated to neural activity (e.g., respiration, cardiac activity) were removed by regressing the BOLD-signals recorded throughout the brain with the mean average of the BOLD-signals recorded in white matter and brain ventricles [32, 33].

### Binarized recurrence plot (bRP) and binarized cross recurrence Plot (bCRP*)*

**Fig 1:**
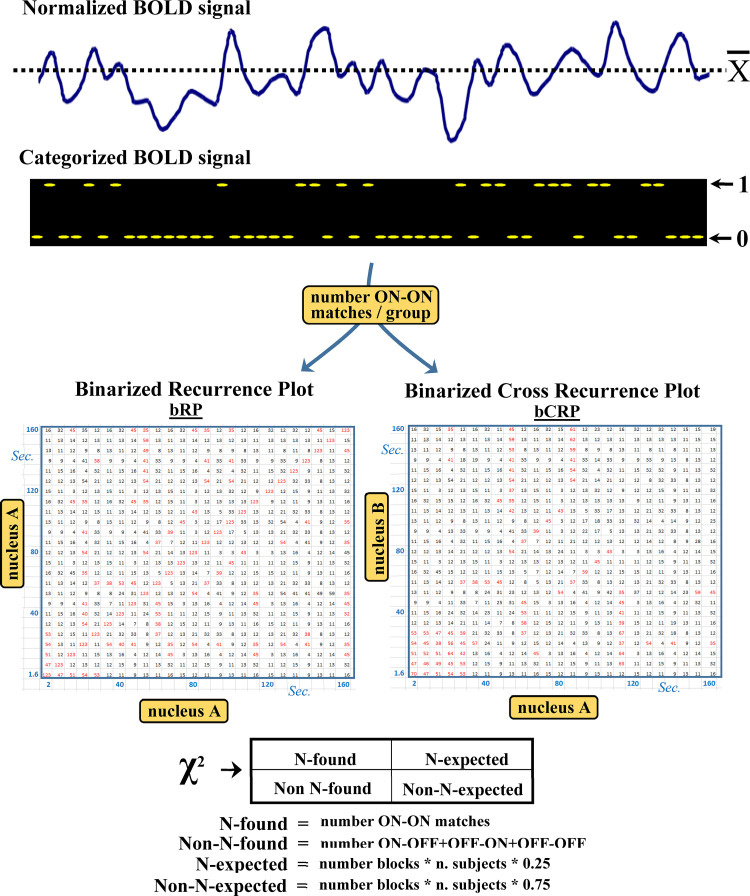
The binarized recurrence plot (bRP) and binarized cross recurrence plot (bCRP) methods. BOLD data are normalized as percentages of the mean BOLD-value of each subject (A) and binarized by replacing the data higher than the mean value with the number 1 (which represent a status of high-metabolic activity) and those lower or equal to the mean value with the number 0 (low metabolic activity) (B). The next step is to calculate the number of 1–1 matches (ON-ON matches) for the bRP and bCRP. The bRP computes the ON-ON match matrix of each task-block of each subject (ON-ON matches of each time-point regarding all the time-points of the same block). This is a 100x100 matrix whose elements can be 1 (ON-ON match) or 0 (ON-ON mismatch). The bRP was computed by adding the two ON-ON match matrices of the same experimental-block of each subject to those of all the other subjects of the experimental group (C). Thus, each of the 100x100 elements of the bRP have a value between 0 (no ON-ON matches) and 40 (the 20 subjects of each group showed ON-ON matches in both task-blocks). The bCRP was computed with the same procedure but using the ON-ON matches of a particular brain region with those recorded in another brain region (D). Thus, whereas the bRP shows the time-dynamic of a brain region, the bCRP shows the time-dynamic of the relationship between two brain regions. The statistical significance (D) of each value of the bRP and bCRP matrices was computed with a Chi^2^ test. The bRP and bCRP values are between 0 and 40 and each of the four possible combinations of the ON and OFF status should be found 25% of times. The distance between the expected and found numbers of ON-ON matches was established using the Chi^2^ distribution, and only values with a Chi^2^ probability lower than 0.01% were considered.

Recurrence plot methods display the long-term dynamic of complex systems with multidimensional states in two dimensions, a representation that can be used to study the dynamic of an isolated system (RP) or the interaction between two systems (CRP). The first RP and CRP studies were performed here with the Recurrence Plot Toolbox 5.8 (http://www.agnld.uni-potsdam.de/~marwan/toolbox/) developed and kindly supplied by Norbert Marwan. The recurrence plot methods were then adapted to facilitate the study of BOLD-signals of subjects recorded in different experimental (resting-task vs. motor-task) and clinical (control subjects vs. PD patients) circumstances. To facilitate the data grouping, the BOLD-activity of all subjects was recorded following a similar task-timetable which made each point in the time-series have the same representation of each of the group members. BOLD-data were recorded during the sequential execution of a motor-task (a repetitive sequence of finger extensions/flexions with the right-hand) and a resting-task (subjects did not perform voluntary tasks except for maintaining a stable body posture and head position). Each experimental block had 100 volumes, and the block sequence was: motor-task block → resting-task block → motor-task block → resting-task block (100 volumes x 2 motor-blocks x 2 resting-blocks = 400 total volumes / subject). Therefore, each subject provided two BOLD points (subjects repeated each task twice) to each of the 100 time-points of each of the two task-blocks of his experimental group. In other words, each of the100 BOLD-points of each experimental condition included 2 data point of the same subjects * 20 subjects = 40 data points. Two additional facts that facilitated the inter-subject grouping of data (see top Fig 1) were the normalization (as percentage of the mean BOLD-value of each subject) and binarization (by replacing the data higher than the mean value with the number 1 -which represents an status of high-metabolic activity- and those lower or equal to the mean value with the number 0 -low metabolic activity-) of the BOLD-series of each subject.

The next step was to compute for each time point of each task-block the number of 1–1 matches (ON-ON matches), a procedure that was different for the bRP and bCRP. **bRP** computation began calculating the ON-ON match matrix of each task-block of each subject (ON-ON matches of each time-point of an experimental block with all the time-points of the same block). This is a 100x100 matrix whose elements can be 1 (ON-ON match) or 0 (ON-ON mismatch). The next step was to add the two ON-ON match matrices of the same experimental-block of each subject to those of all the other subjects of the experimental group. Thus, the new matrix (binarized recurrence plot matrix) had 100x100 elements, each of which with values between 0 (no ON-ON matches) and 40 (the 20 subjects of each group showed ON-ON matches in both task-blocks). This procedure created four bRP matrixes: 1. control-subjects resting-task block matrix; 2. control-subjects motor-task block matrix; 3. PD-patients resting-task block matrix; 4. PD-patients motor-task block matrix. Exactly the same procedure was performed for the **bCRP** but, in this case, the ON-ON matches were established between the binarized/normalized BOLD-data recorded in a particular brain region with those recorded in other brain regions. Thus, whereas the bRP shows the time-dynamic of a brain region, the bCRP shows the time-dynamic of the relationship between two brain regions.

The statistical significance of each value of the bRP and bCRP matrices was computed with a Chi^2^ test (bottom Fig 1). The values of bRP and bCRP matrices can be between 0 and 40 and, bearing in mind that there are four possible combination of the ON and OFF status (ON-ON, ON-OFF, OFF-ON and OFF-OFF), the number of ON-ON matches should be around 10 (25%). The distance between the expected and found numbers of ON-ON matches was established using the Chi^2^ distribution, and only values with a Chi^2^ probability lower than 0.01% were considered. Because the DMN normally works when other networks are not demanding attention, an inverse relationship between the BDM and M1 (ON-OFF or OFF-ON **anti-activations**) should be expected. This possible M1-DMN anti-activation was studied here by computing the bCRP for ON-OFF + OFF-ON matches. In this case the number of ON-OFF + OFF-ON matches should be around 20 (50% of cases), a fact that was taken into account for the Chi^2^ computation.

## Results

**Fig 2:**
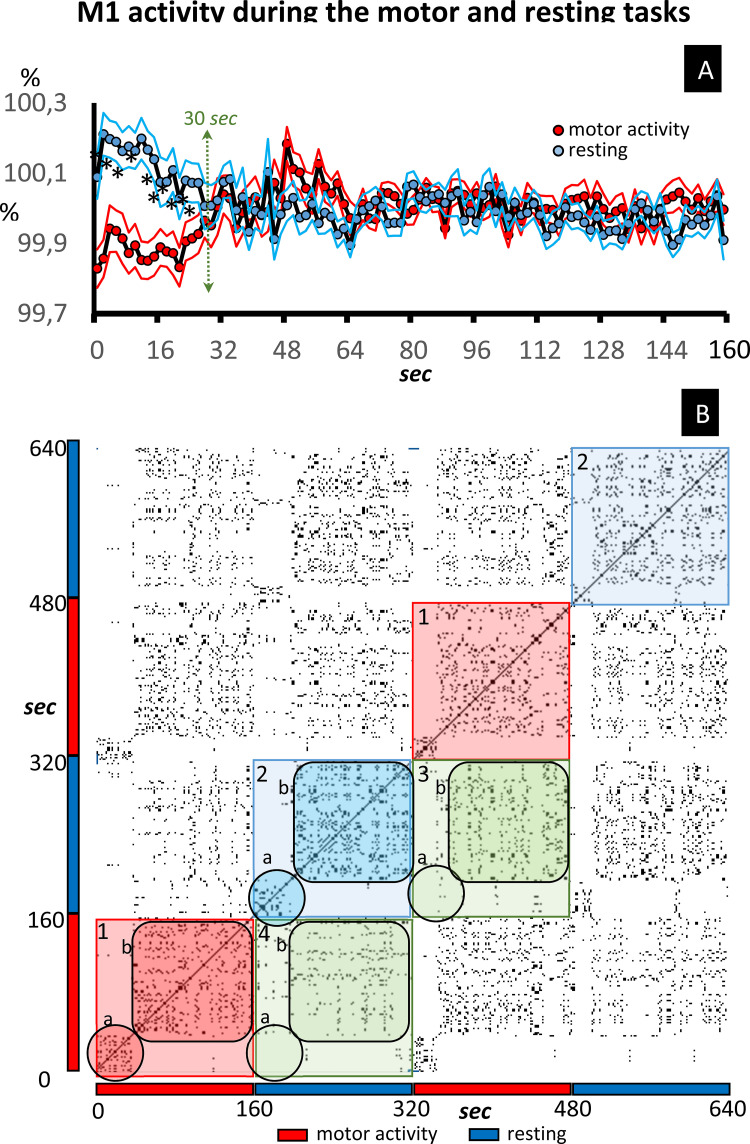
Recurrence plot of the M1 activity. Fig 2A shows the mean ± standard error of the BOLD signal (data normalized as a percentage of the mean value of each subject) recorded in the M1 of control subjects. The data recorded during the motor-task block are shown in red (grouping the 1–160 sec and 320–480 sec time-intervals). The data recorded during the resting-task block are shown in blue (grouping 160–320 sec and 480–640 sec time-intervals). Fig 2B shows a typical RP example of the M1 dynamic in control subjects. The identity line is the diagonal line which goes from the bottom-left to the top-right. The checkerboard distribution shows the limits that coincide with the task-transitions (1: motor-block and 2: resting-block). The points included in squares 3 and 4 show the functional link between the motor-block and the resting block. The initial and late responses of each block are indicated by the letters a and b, respectively.

Top Fig 2 shows the mean ± standard error of the BOLD signal (data normalized as a percentage of the mean value of each subject) recorded in the M1 of control group subjects. Data were continuously recorded for the 640 sec of the study, with the motor-task being performed for between 1–160 sec and 320–480 sec (motor-task block), and the resting-task for between 160–320 sec and 480–640 sec (resting-task block). All task-blocks showed an initial interval of about 30–40 sec that was different to that recoded during the rest of the block (late interval). Regarding initial intervals, the BOLD-signal level of late intervals increased during the motor-task blocks and decreased during the resting-task blocks, a fact that reached statistical value when the two motor-task (red) and the two resting-task (blue) blocks were grouped (Fig 2A).

### Individual RP and bRP of experimental groups

Fig 2B shows a typical RP example of the M1 dynamic in control subjects. The main diagonal line which goes from the bottom-left to the top-right shows the self-association of the time point (**identity line**). The other points show the characteristic symmetrical distribution around the identity line of the RP. The RP detected the task transitions, which produced a checkerboard type distribution whose limits coincided with these transitions (see 1 and 2 in Fig 2B). In addition, the RP detected the transition between the initial and late intervals of each task-block, a fact observed during both the motor-task block (see 1a and 1b in Fig 2B) and the resting block (see 2a and 2b in Fig 2B). The late intervals showed a functional link between the motor-task block and the resting block (3b and 4b in Fig 2B), a fact not observed in the initial interval (3a and 4a in Fig 2B).

**Fig 3:**
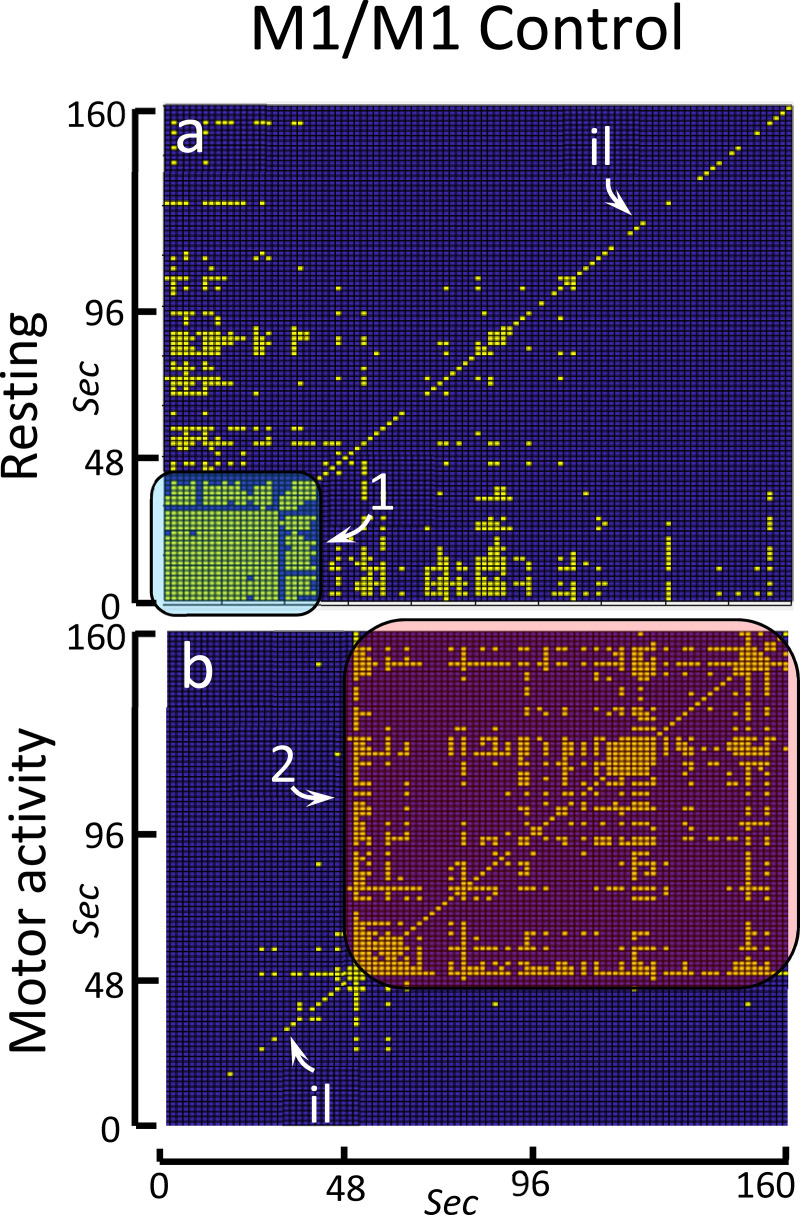
Binarized recurrence plot of the M1 activity in the control group. The bRP shows the main facts observed in the RP of the individual members of the control group. Yellow spots indicate ON-ON matches with a significant high frequency (p<0.01). The significant ON-ON matches are shown is a (resting-task block) and b (motor-task block). Id: identity line. 1: initial response. 2: late response.

The bRP of the control group showed the main findings observed in the RP of its individual members. Yellow spots in Fig 3 indicate the ON-ON matches whose frequency was higher than expected at random (p<0.01), Fig 3A showing matches during the resting block and Fig 3B matches during the motor-task block. The identity line was observed in both blocks. This was a discontinuous line because the OFF-OFF coincidences are not shown in these figures. The bRP also identified the initial (1 in Fig 3A) and late (2 in Fig 3B) intervals found in the RP. As a whole, the characteristics of the bRP of the experimental groups were similar to those found in the RP of the individual members of these groups. Of course, only the behavioral responses which occurred with a phase-locked link to the task-starting can be identified by the bRP, with the other RP characteristics of the members of the group not being detected by the bRP.

### The bCRP between the somatosensory primary cortex (S1) and M1

**Fig 4:**
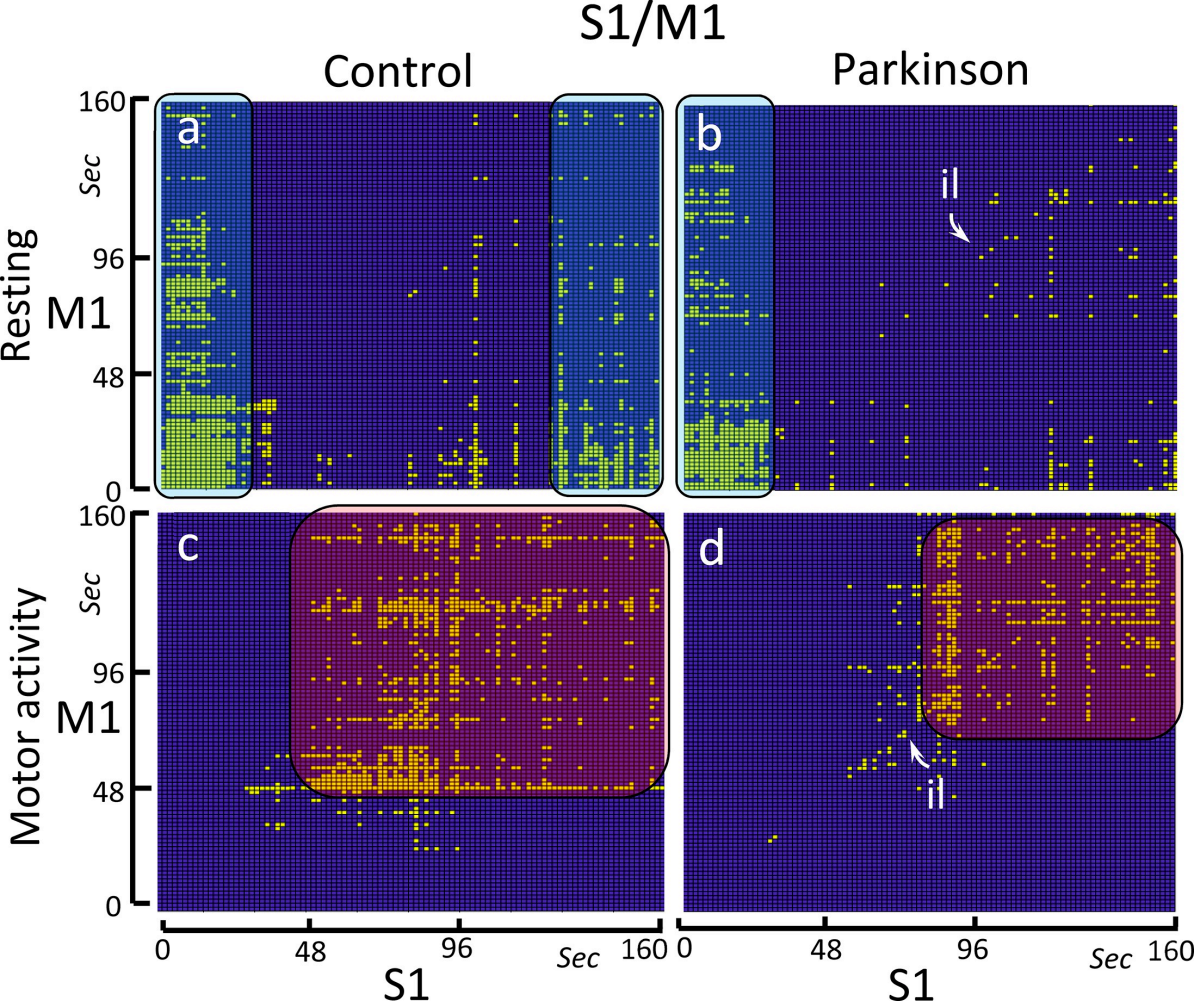
Binarized cross recurrence plot of the S1-M1 functional connectivity. Images show the significant ON-ON matches of S1-M1 in the control group (left-side) and PD patients (right-side), and during resting-task (top) and the motor-task (bottom). The control group shows a high-density ON-ON match at the beginning (0–25 sec) and at the end (130–160 sec) of the resting-task block (Fig 4A), and between the 45–160 sec of the motor-task block (Fig 4C). The PD group shows an identity line (il) which is particularly evident during the motor-task block (Fig 4D). In this group, the high-density ON-ON match at the end (130–160 sec) of the resting-task block vanished (Fig 4B) and the amplitude of the ON-ON match during the motor-task block decreased (Fig 4D).

The bCRP represents in 2-dimensions the long-term interaction between two centers, even when the complexity of this interaction needs a topological representation in a phase-space with many more dimensions. Fig 4 shows the S1-M1 ON-ON matches in the control group (left-side) and PD patients (right-side), and during resting (top) and the motor (bottom) tasks.

As expected for a cross recurrence plot, the control group showed no identity line during the motor (Fig 4A) and resting (Fig 4C) tasks. Instead, long vertical and horizontal lines were identified in both experimental conditions. The S1 presented vertical lines during the first 25 sec and the last 30 sec of the resting-task block (Fig 4A), lines that projected over almost the entire the M1 recording (although this interaction presented a progressive reduction from the 45 to 160 seconds of the block). During the motor task, the S1-M1 relationship started 50 sec after the onset of the block, showing discontinuous horizontal and vertical lines that in some cases project to the end of the block.

The PD group showed an unexpected identity line which was particularly evident during the motor-task block (Fig 4D). Vertical lines found during the first 25 sec of the resting-task block were similar to those observed in the control group (Fig 4B). However, the vertical lines observed in the control group during the last 30 sec of the resting block were not found in the PD group (Fig 4B). The S1-M1 relationship that started 50 sec after the onset of the motor-task block in the control group, does not appear in the PD group until after the first 90 sec of the block (Fig 4D).

### The bCRP between the motor thalamus (MT) and M1

**Fig 5:**
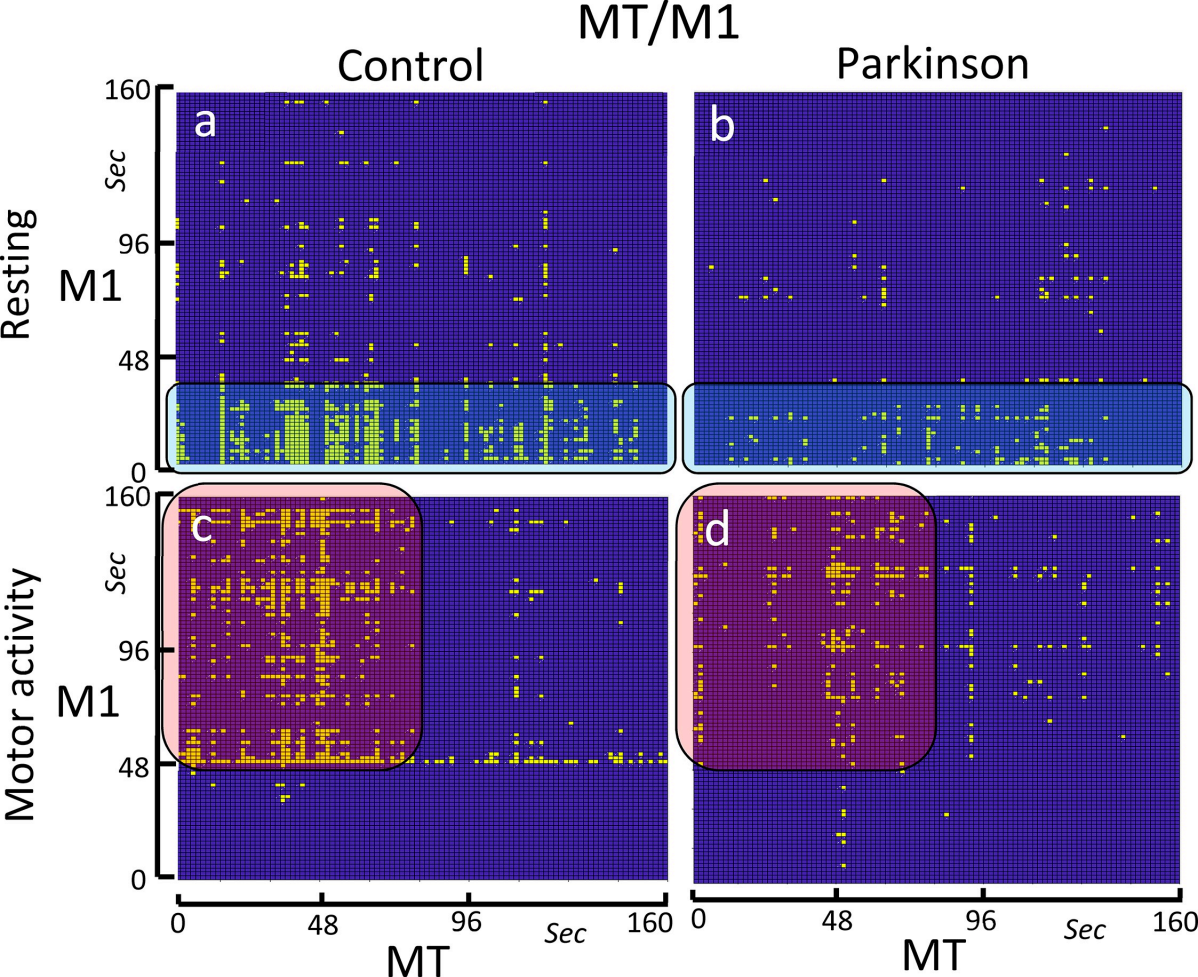
Binarized cross recurrence plot of the MT-M1 functional connectivity. Images show the significant ON-ON matches of MT-M1 in the control group (left-side) and PD patients (right-side), and during resting-task (top) and the motor-task (bottom). The control group shows a high-density ON-ON match at the beginning (0–40 sec) of the resting-task block (Fig 5A), and between the 50–160 sec of the motor-task block (Fig 5C). The PD group shows a decrease of the ON-ON match density in both the resting-task block (Fig 5B) and the motor-task block (Fig 5D).

Neither the control group nor the PD group showed identity lines during the motor-task or the resting-task blocks (Fig 5). The control group showed a short-lasting MT-M1 co-activation which lasted for the entire the resting-task block in the MT (x axis in Fig 5A) and was only found for the first 40 sec in the M1 (y axis in Fig 5A). This early interaction vanished in the motor-task block (Fig 5C), being replaced by an MT-M1 co-activation that started 50 sec after the block onset and that lasted until the end of the block. The MT only showed this interaction with the M1 during the first 65 sec of the motor-task block. PD patients showed both the initial co-activation during the resting-task block (Fig 5B) and the later co-activation during the motor-task block (Fig 5D). However, both co-activations had a much weaker density than that observed in the control group.

### The bCRP between the brain default network (PCn) and M1

**Fig 6:**
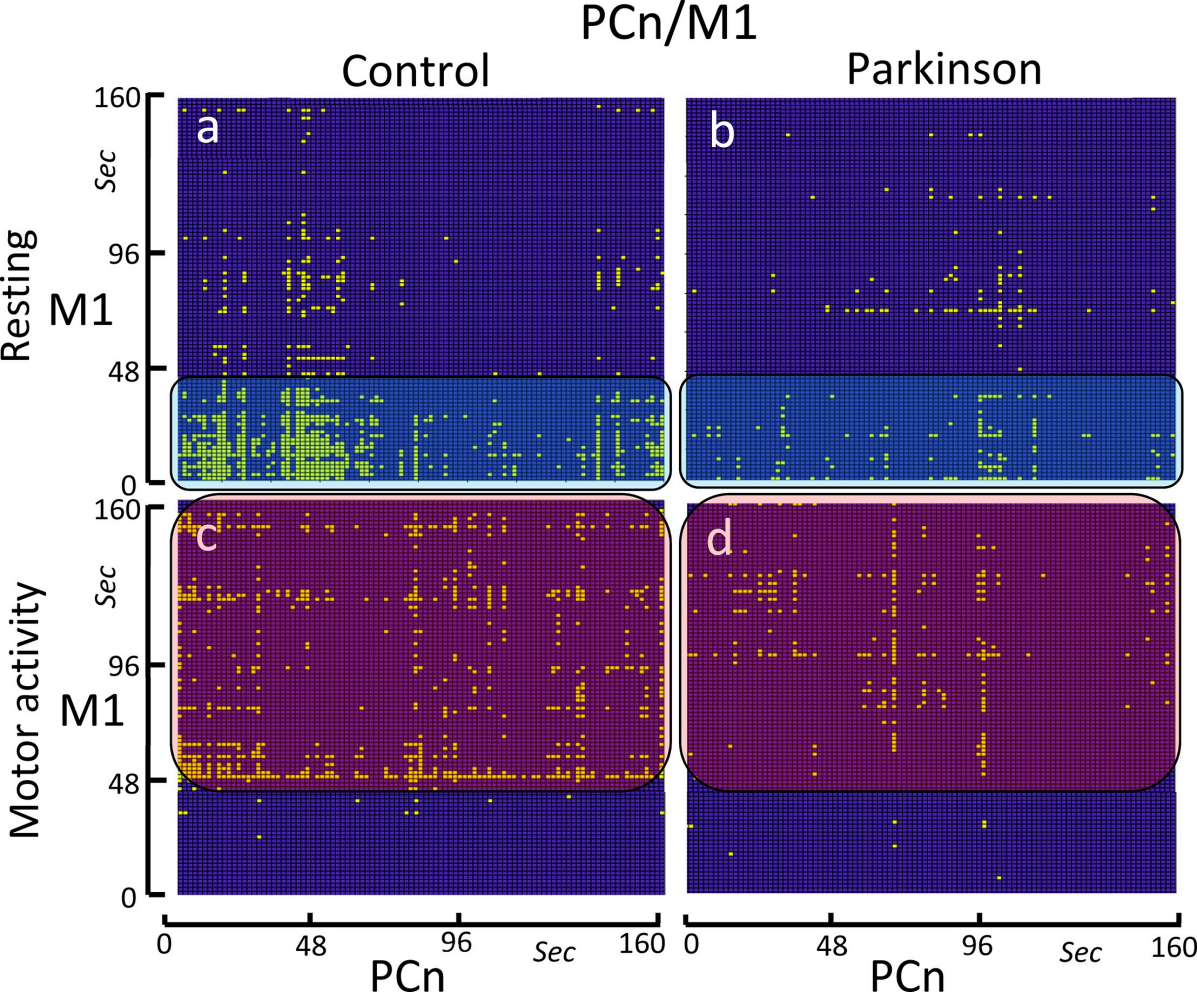
Binarized cross recurrence plot of the PCn-M1 functional connectivity. Images show the significant ON-ON matches of PCn-M1 in the control group (left-side) and PD patients (right-side), and during resting-task (top) and the motor-task (bottom). The control group shows a high-density ON-ON match at the beginning (0–40 sec) of the resting-task block (Fig 6A), and between the 50–160 sec of the motor-task block (Fig 6C). The PD group shows a decrease of the ON-ON match density in both the resting-task block (Fig 6B) and the motor-task block (Fig 6D).

Neither the control group nor the PD group showed identity lines during the motor-task or the resting blocks (Fig 6). The control group showed a PCn-M1 early and short-lasting co-activation (first 40 sec of the block) during the resting-task block (Fig 6A), and a tardive and long-lasting co-activation during the motor-task block (Fig 6C). For the PCn, both co-activations were operative during the whole task blocks. PD patients showed the same PCn-M1 co-activations as the control subjects but, in PD patients, these co-activations had a very weak density (Fig 6B and 6D).

**Fig 7:**
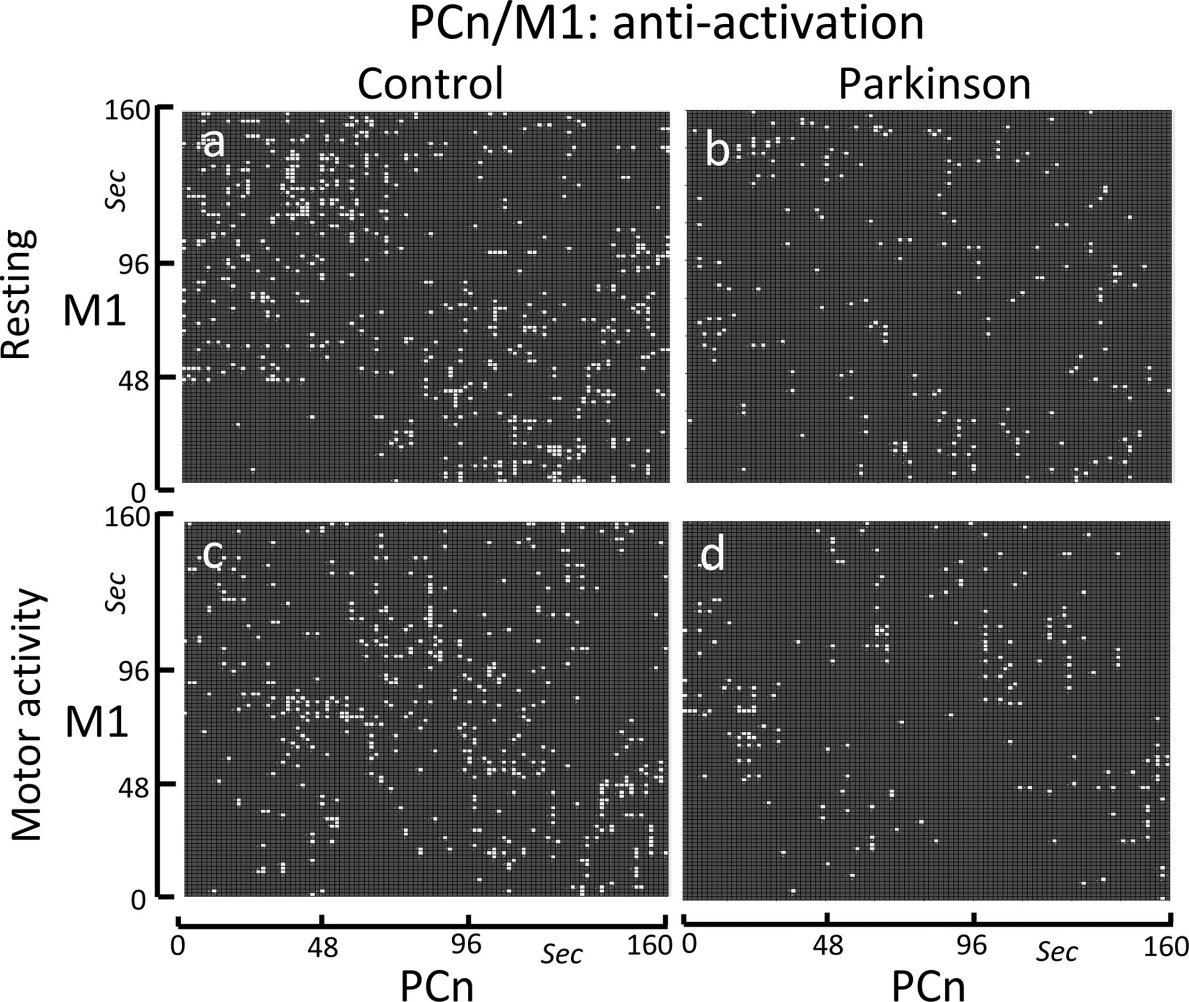
Binarized cross recurrence plot of the anti-activations of the PCn-M1 functional connectivity. Images show the significant anti-activations (ON-OFF + OFF-ON matches) of PCn-M1 in the control group (left-side) and PD patients (right-side), and during resting-task (top) and the motor-task (bottom). The anti-activations showed a short-lasting scattered distribution in the control group during both the resting-task block (Fig 7A) and motor-task block (Fig 7C). PD patients showed a similar short-lasting scattered distribution, but the anti-activation density was much lower in this group (Fig 7B and 7D).

Because the DMN normally works when other networks are not demanding attention, the possible inverse relationship between the PCn and M1 was also studied (see anti-activations in Fig 7). Anti-activations displayed a short-lasting scattered distribution that only occasionally persisted for more than one or two points (1.6–3.2 sec). The density and distribution of the bCRP anti-activations in the control group did not change with the motor task (compare Fig 7A and 7C). PD patients showed a similar short-lasting scattered distribution, but the bCRP density was much lower in this group (compare Fig 7A vs. 7C and Fig 7B vs. 7D).

## Discussion

Two recurrence plot methods that facilitate the study of the long-term dynamic of fMRI and fcMRI data are introduced here. These methods can work with non-stationary and non-linear interactions, and their high-time resolution facilitated the identification in healthy subjects of short and long-term interactions between the M1 and S1, MT and PCn. Most of these interactions were disrupted in PD, and may be at the basis of some motor disorders and drug side-effects often found in PD patients.

### The bRP: Methodological remarks

fMRI usually groups BOLD-data into time-intervals (task-blocks) that are used as time-units. The RP showed these task-blocks, but also other BOLD-responses that were shorter (0–45 vs. 50–160 sec) or longer than the task-blocks, and whose interpretation according to criteria established for the RP may help to understand the complex short and long-term dynamic of individual brain areas [15, 34, 35]. The bRP is an adaptation of the RP which assembles the recurrence plot of the subjects to form the recurrence plot of an experimental groups. The bRP showed the main features observed in the RP of the M1 during the motor task, including the 0–45 sec early and 50–160 sec late RP responses. However, whereas the early and late RP responses were found during both the resting and motor blocks, the early bRP response only appeared during the resting-task block and the late bRP response during the motor-task block. This bRP selectivity was observed in the bRP computed for the ON-ON matches. The bRP was able to distinguish the ON-ON and OFF-OFF matches, which may be relevant for fMRI studies where the physiological meaning of OFF-OFF matches is not evident.

In addition, the RP computation requires some variables whose correct value is not always obvious. This is the case of the “size of the neighborhood”, a parameter used to detect close trajectories in the phase-space. The bRP does not require specific parameters, and only the ON-ON matches with an improbable and statistically high frequency are provided. The aim of this work was to study the functional connectivity of the M1, with the bRP being introduced here only to contrast the present recurrence approach with those previously reported. Thus, no additional comments about the bRP of individual brain regions are provided here.

### The bCRP: Methodological remarks

fcMRI methods use the co-fluctuation of BOLD-data recorded in two brain areas during long-time intervals (> 60 sec) to establish their functional connectivity. This experimental approach assumes that the functional connectivity of centers is stable during the recording interval, a fact that may not always be true. The bCRP showed functional interactions of brain areas that were shorter or longer than the recording intervals normally used in fcMRI studies. Similarly to that performed with the CRP methods in individual recordings, the bCRP features may help to study the complex dynamics of the functional interaction of two brain centers in groups composed of subjects with particular characteristics and which perform different tasks.

**Fig 8:**
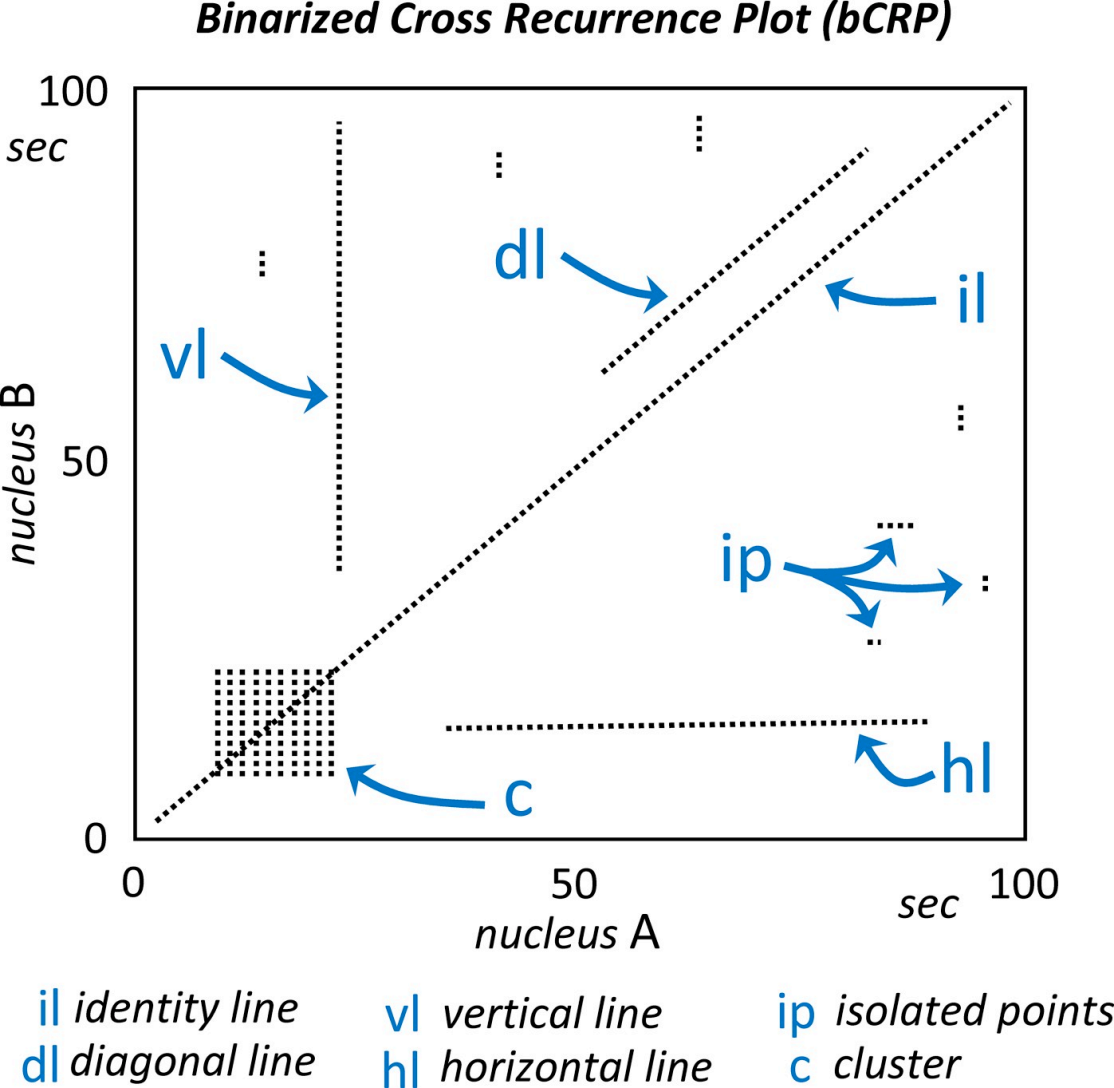
The interpretation of the bCRP. The central oblique line (*identity line*; il) indicates the instantaneous functional coupling between the nucleous A and B. The other oblique lines (*diagonal lines*; dl) reveal a delayed functional coupling of the two regions (the length of these lines are considered as an indicator of predictability). The *vertical* (vl) and *horizontal* (hl) *lines* indicate the persistent action of a brain area on the dynamic of another area (what happens at a specific moment in one area affects the other area over a long period of time). The *isolated points* (ip) indicate short-term couplings in the activity of two brain areas, and *clusters* forming squares (C) indicate that the two areas have an intensive coupling during the time-interval corresponding to the side of the square.

The bCRP compress the multidimensional long-term fluctuations of the interaction of two brain areas into a motionless two-dimension image, and this compression makes it difficult to intuitively assess all the bCRP features. The interpretation of the bCRP proposed here is based on the standards previously used with the CRP and which are shown in Fig 8. The bCRP may present a central oblique line (*identity line*; il) which indicates the instantaneous functional coupling between two brain regions. The *identity line*, that is always present in the RP, is not usual in the CRP, and its finding in the bCRP indicates that, in most subjects of the group, what happens in one brain region happens simultaneously in the other. The other oblique lines (*diagonal lines*; dl) reveal a delayed functional coupling of two regions which have a similar time evolution (what is now occurring in one nuclei will happen later in the other nuclei). The length of oblique lines are often considered as an indicator of predictability, with very short diagonals being associated with weakly correlated or chaotic interactions and long diagonals being associated with highly deterministic interactions (the length of these lines has been associated with the Lyapunov exponent). The *Vertical* (vl) and *horizontal* (hl) *lines* indicate the persistent action of a brain area on the dynamic of another area? (what happens at a specific moment in one area affects the other area over a long period of time). The *Isolated points* (ip) indicate short-term couplings in the activity of two brain areas, and *clusters* forming squares (C) indicate that the two areas have an intensive coupling during the time-interval corresponding to the side of the square.

The usefulness of bCRP to identify short and long-term interactions between brain centers has some limitations. The bCRP can identify the functional interaction between two nuclei, but does not provide a direct description of the dynamic of networks composed by several nuclei. Similar to what happens with the CRP, this method may help to understand complex interactions between nuclei but, on its own, it does not identify the basic nature of these interactions (e.g., deterministic chaos, fractal behavior.). These limitations are accompanied by others derived from the nature of MRI recordings, a method based on the metabolic response to the brain processing of information, and which, at the moment, has a limited space and time resolution. Despite these methodological limitations, the bCRP provides relevant information about the brain functional connectivity which cannot be obtained with other methods. The future incorporation of procedures to quantify the bCRP features (similar to those used with the CRP may improve the usefulness of these methods in fMRI and fcMRI studies.

### S1-M1 functional coupling: PD effects

The M1 uses the somatosensory information provided by the S1 to modulate the muscle tone and body posture during motor resting [30, 36, 37], and the S1-M1 co-activation found during the resting-task block could be at the basis of these M1 functions. The S1-M1 co-activation showed three successive patterns, a high density pattern between 0–40 sec, a low-density pattern between 40–120 sec, and a high-density pattern between 120–160 sec. The initial high-density co-activation could be necessary to prepare a body posture that will remain stable and free of movement for the 160 sec of the resting-block. The second low-density co-activation may facilitate the testing and adjusting of the body posture that are necessary to stay in the initial position. The third high-density co-activation could be involved in the adaptation of the body posture necessary to support the motor activity to be performed during the following motor-task block (note that resting and motor task blocks were performed consecutively).

The S1-M1 co-activation during the resting-task block was different in PD patients. The first and second co-activation patterns were similar in the control and PD groups. However, the third S1-M1 co-activation which occupied the last 40 sec of the block in the control group, vanished in PD-patients. PD patients have problems using the somatosensory information for preparing the next motor pattern, a deficiency which may be compensated for by visual or auditory stimuli [38–40]. The lack of the third S1-M1 co-activation pattern could be an indicator of this PD disorder.

M1 activity is critical for the planning and execution of finger movements [30, 41], a fact that could be at the basis of the S1-M1 co-activation found between the 45–160 seconds of the motor-task block. Prefrontal areas may be particularly relevant to supervise the initial execution of movements, which could justifying the absence of S1-M1 co-activations found during the first 45 sec of the block. The repetition of simple movements facilitates their unsupervised execution, and the somatosensory information provided by the S1 to M1 may be critical for executing these automatic movements [42]. This could be at the basis of the S1-M1 co-activation found between the second 45 and the end of the motor-block, a time-interval where the finger movements can be performed automatically and without prefrontal supervision (unless the somatosensory S1 information indicates significant differences between actual and planned movements) [6, 43, 44].

The marked reduction of the S1-M1 co-activation found during the motor-task block could be at the basis of the hypokinesia of PD patients. In these patients, a portion of the S1-M1 co-activation was moved to the identity line, a line observed in all bRPs but never found in the bCRP of control subjects. Its finding in the bCRP reveals a fast (which occurs in less than 1.6 sec), generalized (observed during both the resting-task and the motor-task blocks), persistent (extends beyond the S1-M1 co-activation interval found in the motor-task block) and highly deterministic S1-M1 interaction in PD patients. In these patients, the somatosensory S1 information could exert a powerful “online” influence on the M1 activity that imposes itself on the motor action performed by other brain centers. This could be at the basis of PD dyskinesias, repetitive motor patterns that are executed without voluntary control and that hamper the execution of voluntary movements [45]. The concentration of the S1-M1 co-activation in the identity line could also explain a motor paradox observed in PD patients, who present involuntary repetitive motor patterns (dyskinesias) [46, 47] and yet cannot automate the repetition of simple motor patterns [48, 49].

### MT-M1 functional coupling: PD effects

The MT-M1 co-activation showed a complementary distribution during the resting (0–40 sec) and motor (50–160 sec) blocks. The vertical lines preponderated over the horizontal lines, showing a laminar state suggesting that the MT induced a persistent influence on the M1 activity during both task-blocks. The muscle tone and motor functions of the M1 are influenced by BG throughout the MT→M1 projections of the posterior cortico-subcortical loop of BG (M1→striatum→external pallidum→ subthalamus→substantia nigra/internal pallidum →MT→M1) [4, 49, 50]. The glutamatergic MT→M1 projection stimulates the M1 activity, and its depression has been involved in the hypokinesia (a partial loss of muscle movements) and hypertonia (an increase of the muscle tone that hampers the voluntary movement and alters the body position) of PD patients [51–53]. The bCRP of the control subjects suggests that the MT-M1 co-activation may be particularly relevant for muscle tone during the first 40 sec of the resting-task block and for the motor activity during the 50–160 sec of the motor-task block. PD patients showed the same MT-M1 co-activations observed in the control subjects, but their density was much slower in the patients. This low MT-M1 co-activation could be associated with hypertonia during the resting-block and with hypokinesia during the motor-block.

### PCn-M1 functional coupling: PD effects

The bCRP showed long vertical PCn-M1 co-activations during both the resting-task and the motor-task blocks. The DMN is activated when complex motor plans or other cognitive demanding mental tasks are not being executed, and the finding of M1-PCn co-activation suggests that these centers do not always present the temporary incompatibility that would be expected for areas involved in voluntary (M1) and passive (DMN) brain activities. The PCn-M1 co-activations were different during the resting-block and motor-block (more marked during the first 40 sec of the resting-block and during the last 120 sec of the motor-block), showing a M1-DMN collaboration that is task-dependent. It is possible that the M1-PCn co-activation may be produced when the M1 is performing automatic unsupervised motor actions and the mental resources can be shifted towards the DMN, a coordination that may be assisted by the direct PCn-M1 projections [7].

On the other hand, there were M1-PCn anti-activations (ON-OFF and OFF-ON matches) found which are compatible with the common idea that the active motor function of the M1 and the passive mental function of the DMN are incompatible and cannot be performed at the same time. M1-PCn anti-activations were scattered throughout the resting and motor blocks, suggesting that the incompatible activities of these centers may be performed at any time and independently of the motor task in progress. Anti-activations rarely exceeded 3.2–4.8 seconds, suggesting that the incompatible activities of the M1 and PNc are highly dynamic and of a short duration. M1-PCn anti-activations could appear when subjects need to supervise the body position or the execution of automatic movements, and the attentional resources must be withdrawn from the DMN. When this motor supervision is not necessary, both centers may be working at the same time (the M1 executing automatic movements and the DMN analyzing past events or performing any of its other mental tasks) and the co-activation of both centers will be shown by the bCRP.

PD patients showed a marked decrease of the density of both the co-activation and anti-activations of PCn-M1. These patients have a particular impediment when performing automatic movements, and many of their actions require attentional supervision [30, 54]. A deficiency in the mechanisms involved in the PCn-M1 anti-activation could allow a DMN activation during the execution of movements, thus withdrawing the mental resources that in PD patients (and in some aged subjects) are necessary for the supervision of motor actions [31, 55, 56]. Therefore, the extemporaneous activation of the DMN would hinder the periodic supervision of the ongoing motor actions of the M1, generating an interference that could be at the basis of the motor blocks of the gait, speech, handwriting and other complex motor patterns frequently observed in PD patients [57–60].

## Conclusions

In summary, two recurrence plot methods which were used with the aim of studying the long-term dynamic of single brain centers (bRP) and the interaction of two brain centers (bCRP) with BOLD-signal recordings are introduced here. These methods were used to group BOLD-data according to the characteristics of subjects (e.g., control and PD patients) and the performed task (e.g., body posture vs. motor activity). The high time-resolution of the bCRP and the possibility of aggregating individual subjects with non-stationary data in experimental groups facilitated the finding of short-lasting and long-lasting functional interactions of the M1 with the S1, MT and PCn. Most of these interactions were disrupted in PD, and may be at the basis of the PD motor disorders and of some of the side-effects induced by anti-parkinsonian drugs. The ability of the bCRP to identify long-lasting interactions between centers could help to understand the complex dynamic of BG and other brain networks, and to facilitate the diagnosis and the understanding of the clinical expression of BG diseases.

## Supporting information

S1 Data A table showing the M1, S1, MT and PCn data recorded in this study. Values are anonymized and normalized for each subject around 100 (according to the procedure specified in Fig 1).
(PDF)
