# Supplementary material for: Studying the functional connectivity of the primary motor cortex with the binarized cross recurrence plot: The influence of Parkinson’s disease
Source: PLoS One. 2021 Jun 7;16(6):e0252565. doi: 10.1371/journal.pone.0252565 (PMC8183987; doi:10.1371/journal.pone.0252565)
Supplement: S1 Data — Values are anonymized and normalized for each subject around 100 (according to the procedure specified in Fig 1). (PDF) [file pone.0252565.s001.pdf]

M1	S1	MT	PCn
99.6949343	100.009844	100.179305	99.4657877
99.6290459	99.9042568	100.218145	99.1949489
99.5506563	99.8268081	100.063196	99.1550466
99.6291929	99.8629866	100.130245	99.3704898
99.4978573	99.7015235	100.505557	99.4447403
99.6125738	99.6033057	100.308089	99.6259815
99.5150648	99.6544915	100.029467	99.8446404
99.4878564	99.7918356	99.5713651	100.245125
99.5674225	99.8230563	99.4624102	100.209754
99.6709615	99.899701	99.6633534	100.097647
99.6562543	99.688392	99.5143324	100.24191
99.613015	99.7607489	99.4848962	99.9222525
99.6868453	99.9163163	99.5889451	99.83894
99.4447642	100.033427	99.7678112	99.8912661
99.6084557	100.095467	99.6909499	99.8224237
99.6878748	99.8312299	99.8313853	100.183445
99.3557854	99.8227883	100.077301	100.334284
99.3929947	100.018286	99.94218	100.368194
99.5983077	100.228791	99.9832681	100.186222
99.5505092	100.248488	99.9442242	100.268073
99.5965429	100.238707	99.6645799	100.385879
99.4680016	100.166886	100.080776	100.113287
99.5577158	99.970182	100.103466	100.181983
99.5621279	99.8408775	99.7434854	99.9653704
99.6325756	100.055804	99.8957771	99.5546543
99.7737651	100.190603	99.790706	99.8222775
100.238661	99.7890217	100.188913	100.182421
100.261751	100.367073	99.9808151	99.7738978
100.167478	100.597275	99.8317941	99.6226198
100.146299	100.414641	100.21283	99.3984068
100.275723	100.321247	100.660915	99.5920719
100.272341	100.05701	100.900289	99.7749209
100.170861	100.10029	100.372685	99.8494637
100.089088	100.261352	99.9360475	99.8770884
100.177479	100.252508	99.7612698	99.8336782
100.07041	100.249158	99.8436504	99.5685398
100.141005	100.279709	99.7847779	99.7974299
100.101148	100.297798	99.8450813	100.133457
100.003345	100.38677	99.9474949	100.19163
100.183656	100.404591	99.9333901	99.7456885
100.164389	100.281317	99.9041582	99.7553352
100.062174	100.203868	99.8781971	99.6717303
100.138211	100.197704	100.034782	99.6167733
100.10659	100.139283	99.9908316	100.016966
99.9542229	99.8570908	99.9215338	100.394941

99.9770192	99.8423515	100.091814	100.380325
100.160566	99.922614	100.232045	100.117233
100.089971	100.16434	100.125952	99.6403054
100.269105	100.186717	100.579352	99.748904
100.328081	100.217535	100.266183	99.6468828
100.153506	100.296458	99.9368652	99.6230583
100.062762	100.25693	99.7929546	100.146758
100.270723	100.285337	99.8516227	100.300959
100.274105	100.362383	100.130654	100.184614
100.214247	100.055	99.966097	100.328291
100.198363	99.9369514	99.930937	100.339838
100.009081	99.9341375	100.104897	100.342469
100.218953	99.8222523	100.034373	100.487901
100.2819	99.5856184	100.084455	100.830651
99.9936383	99.5838765	100.053997	100.786218
99.7830307	99.8420835	99.9867433	100.694866
99.7745005	100.015204	99.8743132	100.710944
99.9130427	100.020966	99.8342472	100.792211
99.8774511	99.8473092	99.7606565	100.694136
100.103501	99.9514228	99.7716951	100.375063
100.140564	100.10699	100.023539	99.8215467
100.060703	100.089571	99.894755	99.6066881
100.016435	100.031149	99.6817511	99.7147021
100.211453	100.165948	100.108168	99.5112441
100.243073	100.074296	100.58712	99.1557774
100.303226	99.9758098	100.338547	99.2757767
100.212629	100.003145	99.7849823	99.9259066
99.9226024	99.8025552	99.9493347	100.325807
99.9881967	99.6317125	100.06708	100.257695
100.068057	99.5758368	100.121251	100.581591
99.6414	99.6313105	100.015975	100.565659
99.8431833	99.6256827	99.9857212	100.43309
99.9370155	99.7500294	100.16377	100.282981
100.026141	99.7322082	99.8342472	100.354016
100.07394	99.5785167	99.9370696	100.494332
100.140858	99.6590473	100.547667	100.322591
100.236896	99.7938455	100.47101	99.873142
100.309697	100.139417	100.238178	99.4119999
100.136446	100.265773	100.00739	99.30764
100.35426	100.39414	100.059516	98.8804076
100.417501	100.099486	100.015771	99.1522695
99.9568702	100.040931	100.048886	99.4581872
100.113502	99.853473	100.354492	99.7617663
100.079087	99.9088126	100.029467	99.5857869
100.019817	100.073626	99.9295061	99.4521946
100.142034	100.141159	99.8845341	99.1212831

100.222924	100.129099	100.180123	98.9885677
100.305726	100.120524	100.49227	99.1625009
100.101589	99.875448	100.033351	99.99738
100.205128	99.7390419	100.045411	100.378133
100.231013	99.5084377	100.05645	100.635525
99.8373004	99.5054898	99.850805	100.704075
100.133357	99.6705708	99.9417712	100.485708
100.092765	99.6031717	99.9006831	100.389972
100.16939	99.8404755	100.249421	100.177744
100.420097	99.7294108	100.375056	100.386493
100.438526	99.6981721	100.022537	100.366143
100.420835	99.8071722	100.128599	100.469652
100.22122	99.8616052	100.071378	100.571989
100.50413	99.9786495	99.9773733	100.69292
100.468895	100.132295	99.9230138	100.441835
100.487619	100.074511	100.138817	100.358676
100.488651	100.153345	100.489702	99.8909105
100.245693	99.9962128	99.9311881	99.9737761
100.417296	100.019407	100.046855	99.6647139
100.667478	100.054802	100.244675	99.5953177
100.487913	100.24277	100.063	99.4421775
100.27046	100.388238	100.148831	99.5101096
100.367172	100.240893	100.088136	99.3518452
100.496612	100.109101	99.988613	99.5988314
100.468601	99.8953912	99.8075509	100.096757
100.425995	99.9285068	100.195424	99.9947121
100.421424	99.8755486	100.496241	99.6982408
100.34491	99.9395006	99.953872	99.8401078
100.371742	99.7406728	100.093041	100.001447
100.306432	99.8746101	99.7766926	99.9254622
100.313214	99.9086642	100.092632	99.9267799
100.556909	100.306454	99.8766242	99.9827068
100.244513	100.463988	99.8512837	99.7702723
100.01615	100.126128	100.149648	99.7510932
99.77275	100.295192	99.8811201	100.079627
99.7619879	100.092074	99.996583	100.038487
99.8529497	100.202683	99.8911337	99.9007197
99.8878897	100.131089	99.9485586	99.6172785
99.8581096	99.9023629	99.5488323	100.019455
99.868282	99.9746273	99.9174961	99.8680713
99.8073951	99.9228758	100.464974	99.8859327
99.8040043	99.9086642	100.09161	99.9619172
99.6670455	99.7483148	99.9969917	99.9269263
99.9375722	99.8925757	100.171514	99.7035114
100.010106	99.8931119	100.255302	99.641289
99.8920176	100.02048	100.032959	99.9603068

99.9136892	100.025038	99.8525099	100.232475
100.022932	100.057349	99.7670878	100.000275
100.041213	99.9600136	100.158027	99.8427431
99.8743265	99.9145634	100.052782	99.8382045
99.9663203	100.13739	100.079144	99.9076008
99.8281821	100.209118	99.9465151	99.8168293
99.9130995	100.206839	99.9005343	100.085923
99.9467126	100.266367	100.068109	99.9573787
99.8323101	100.197052	100.090384	100.066012
99.7782047	100.442402	99.8991037	100.060155
99.8949661	100.487316	99.8514881	99.4538899
99.9495137	100.310342	99.9187222	99.4417382
99.8249388	100.28755	99.9031909	99.8364477
99.8006135	100.372015	99.9724687	99.4721906
99.8814029	100.245854	100.028667	99.2294502
100.183774	100.302298	100.239157	99.3487707
100.074384	100.173455	100.094471	99.814926
99.8626798	100.049573	99.5935869	100.201876
99.7963381	99.9825376	99.7221288	100.155905
99.8458733	99.925155	100.046651	99.9383459
99.8466104	99.6183996	99.9687902	99.8740739
99.8948187	99.8295621	100.162931	99.7169807
99.8137344	100.220648	100.082823	99.6726198
99.8917227	100.34681	99.845766	99.7259115
99.9400785	100.32214	99.7854801	99.8302986
99.9810629	100.43342	100.019676	99.7354278
99.77275	100.206169	99.9070737	99.4773148
99.8020877	100.090867	99.8888858	99.5342666
99.8339318	100.188739	99.9121827	99.4714586
99.8616478	99.9109434	100.077101	99.4493513
100.054923	99.8129372	100.12206	99.8124371
100.054334	99.8498069	99.9712425	99.776275
100.184216	99.853829	99.9438584	99.6970696
100.082492	99.9070554	99.652851	100.028825
100.324565	99.8756827	99.8236952	100.234378
100.010843	99.718551	100.078327	100.471994
100.021163	99.8010049	100.078531	100.49293
99.9108881	99.9743592	100.214021	100.529092
100.040918	99.9065191	99.911774	100.743577
99.8881845	99.8784982	100.359933	100.516648
99.8283296	100.059226	100.328871	100.165421
99.901158	100.029865	99.8120468	99.5535921
100.00642	100.003989	99.9085043	99.1921167
100.008779	100.208582	99.7695401	99.6083478
100.153109	100.08765	99.6861616	100.195434
100.010401	99.8337183	100.258163	100.411529

99.8219903	99.6312705	100.318857	100.772565
99.7581548	99.5992274	100.016406	100.87783
100.119791	99.6051266	100.019471	101.079138
100.059936	99.620947	100.026624	100.939614
99.8330472	99.6548671	99.7053713	100.726154
99.8507383	99.5305829	99.7464475	100.616936
99.7083248	99.6853013	99.8061204	100.431293
99.8625324	99.6456161	99.9078912	100.605077
99.7114208	99.7951057	100.12819	100.804335
99.646111	99.8378745	100.034389	100.820732
99.836438	99.7833075	99.499173	100.775493
99.8777173	99.6977699	100.019471	100.705218
99.8432196	99.5414427	100.364838	100.604345
99.730881	99.7821008	100.05973	100.627331
99.692108	99.8699176	100.060752	100.031753
99.7780573	99.8755486	99.9013517	99.9862206
99.4205003	99.9255096	100.078054	100.009175
99.4923777	99.8680691	100.064145	100.318074
99.590272	99.8820266	100.107714	100.5092
99.5408838	99.7166839	100.027941	100.682352
99.614966	99.7354729	100.089304	100.82219
99.5494092	99.6326705	100.2601	100.662772
99.5505851	99.673872	99.865941	100.805824
99.6640602	99.6579014	100.159873	100.58489
99.6681759	99.7101078	100.1791	100.560196
99.3082011	99.7326545	100.058623	100.474423
99.5220693	99.8131786	100.038782	100.554497
99.666265	99.8622982	100.045736	100.470185
99.5420598	99.9837553	99.9964409	100.535355
99.4937005	99.8463276	99.5623959	100.712892
99.6446577	99.9354409	99.9223955	100.396979
99.9073261	100.063743	100.249668	99.9193107
100.216443	100.229891	100.494918	99.4771497
100.284498	100.169632	100.524577	99.0538384
100.036088	100.093134	100.084395	99.4306834
100.109435	100.119573	100.0486	99.4369666
100.278766	100.014086	100.232691	99.0535461
100.078862	100.056495	100.139213	99.3399425
99.9321671	100.10226	100.127964	99.5402738
100.166467	99.969932	100.114054	99.5822104
100.11605	100.073942	100.181759	99.6283846
99.9142346	100.129101	100.025895	99.8589629
100.060488	100.031399	99.5707823	100.06207
99.9930204	99.9069891	100.020168	99.9191645
100.17705	99.9355751	100.409622	99.862762
100.174404	100.142253	100.196895	99.9478042

00.310809	100.275789	100.222668	99.7259931
100.246281	100.403554	100.007691	99.1185698
100.125016	100.322493	99.9330319	99.0858388
100.15118	99.934233	100.062304	99.2335667
100.179402	100.023749	100.029782	99.0924142
99.8355957	100.18171	100.027941	99.0868616
100.02183	100.265321	100.164986	99.0696194
100.138098	100.258879	100.227782	99.2066805
100.067984	100.234722	99.8141911	99.5979915
100.049905	100.106823	99.7464866	99.2741882
100.130602	100.249753	100.214486	98.950385
99.8691091	100.043746	100.106895	99.1847624
99.8181041	99.9069891	99.9211683	99.2094568
100.048288	99.8600167	99.8810774	99.4558161
99.984789	100.046161	100.026918	99.5379359
100.127956	100.237003	99.97885	99.5094424
99.9746468	100.238882	99.879441	99.5760734
100.132512	100.265724	100.013009	99.5611691
100.164115	100.254719	100.068032	99.6538095
100.023594	100.164398	99.5259869	99.7080203
100.040498	100.338061	99.8039638	99.4993601
100.362549	100.10736	99.8745319	99.8345607
100.268771	100.021736	100.173168	99.930562
99.9752348	100.253242	99.8258502	99.8392366
100.094148	100.227743	99.7565093	99.8427435
99.9002706	100.139569	99.96085	99.6764582
99.9744998	100.117023	99.9434637	99.9876951
100.135011	100.088168	99.8465093	100.257726
100.084447	99.8790741	99.9285319	100.410714
99.9821432	99.695345	99.9074637	100.647576
99.9167334	99.6949424	99.703123	100.736563
99.9911095	99.6757509	99.8962137	100.553912
100.040057	99.4944375	100.079895	100.559903
99.7857666	99.6734693	100.075395	100.347736
99.9490708	99.7605695	99.9843727	100.39391
100.085623	99.8005631	99.9622819	100.404139
100.119724	99.9252412	99.7524184	100.433655
99.946131	99.8507565	99.9140092	100.234493
99.8483837	99.6792402	99.7395321	100.147113
100.058871	99.7050079	99.7133503	100.135131
100.044172	99.7695614	100.175213	100.153834
100.008895	99.9975786	100.137373	100.019111
99.9533335	100.250424	99.9596228	99.7733362
100.159411	100.220093	100.088895	99.7229246
100.193954	100.190434	99.8970319	100.009467
100.218501	100.072869	99.9710773	100.383244

100.252455	100.151245	99.8737138	100.600963
100.270094	100.094744	99.7139639	100.591027
100.217913	100.024286	99.6080095	100.559757
100.224674	99.8365305	100.050441	100.691704
100.093707	99.5168501	100.023236	100.566917
99.8613187	99.6490437	100.0126	100.618644
99.7262363	99.8210968	100.173986	100.618498
99.8132535	99.8633719	100.053714	100.485674
99.8348608	99.7982816	100.039395	100.117158
99.7716557	99.7871425	99.9475546	100.297617
99.7712148	99.7513093	99.9680091	100.4715
99.7565159	99.8758531	99.8231911	100.338823
99.7027181	99.790095	100.048395	100.389235
99.8367716	99.8824292	100.2196	100.109414
99.9155574	99.838141	100.140236	100.256995
99.9436322	99.7644615	100.221645	100.392595
99.9173213	99.9281937	100.165191	100.362787
100.082242	100.106286	99.9962364	100.219588
100.003604	100.141448	99.8608274	100.318074
100.013158	99.9872447	99.6206913	100.555374
100.106789	100.116083	99.8180775	100.325964
100.239373	100.157016	100.025077	100.332832
100.185428	100.112728	100.197304	100.558442
100.212915	100.342356	100.182373	100.477491
100.20439	100.507296	100.042668	99.7609159
100.481933	100.243385	99.8199728	99.3440352
100.488119	100.130072	99.6628397	99.3106563
100.623036	100.059084	99.9370563	99.6529367
100.580175	100.050512	100.251118	99.8032883
100.411234	99.9440304	100.081725	100.181583
100.479723	99.9496559	99.9677065	100.109555
100.525236	100.076363	99.9875269	99.4551519
100.655588	100.186327	99.7239356	99.5292297
100.492685	100.232536	99.9589201	99.8129506
100.440839	100.50738	99.8222204	99.8400344
100.188678	100.341831	99.8986415	99.8057771
100.074529	100.27245	100.373923	99.7606862
99.9643558	100.401166	99.9946786	100.048213
99.8559504	100.142797	99.7656198	100.440123
99.906029	100.168915	99.9066105	100.038844
100.074529	100.005241	99.9284743	99.7333097
99.7494598	100.007786	99.9168272	99.6694798
99.7198545	100.049843	99.9339913	99.7394584
99.7836311	99.9898377	99.8673783	99.9057674
99.9020522	100.109446	99.8001523	99.9512975
99.953751	100.155119	100.418264	99.8485255

100.002062	100.143064	100.372288	99.7562943
99.8933621	100.099668	100.077843	99.4832605
99.9530145	99.82094	99.9425734	99.3173906
100.094413	99.7849103	99.9646415	99.7482423
99.9170758	99.7243697	100.016951	99.9558358
99.9861547	99.796563	99.7858489	100.171481
100.030342	99.8579073	99.865335	100.336766
100.11194	100.066853	99.7218923	100.145569
100.066722	100.361118	99.6742824	99.8491111
99.775972	100.146011	99.7051369	99.9082562
99.9497741	99.7973667	99.8755517	100.219061
99.7993911	99.6460151	99.8536879	100.626196
99.6853887	99.7983042	100.03718	100.761322
99.7646307	99.7219587	99.9329696	100.86263
99.9061763	99.6445417	99.8455146	100.671287
99.7818636	99.5573471	100.10175	100.482725
99.7562352	99.4014416	99.9887529	100.627367
99.7441574	99.5736877	99.7570378	100.494144
99.7223585	99.6535157	99.9194836	100.47482
99.9232619	99.6666417	99.8667653	100.535575
99.870974	99.6232454	100.10175	100.315831
99.8672918	99.7577206	100.007347	100.3618
99.9088275	99.7486127	99.8841337	100.322418
100.018853	99.681509	99.8322328	100.229894
99.8614002	99.7057521	99.7112669	100.288308
99.8538884	99.9784528	99.9891616	100.165772
99.7560879	100.112526	100.189818	99.9501263
99.7600647	100.415765	100.26297	99.6129698
99.8298801	100.10824	100.166728	99.3535511
99.7954143	100.151503	99.9156012	99.4223586
99.9815888	100.135564	99.7670502	99.2188642
100.149499	100.090426	99.9491121	99.4305569
100.081157	100.031627	99.9517684	99.3371545
99.9075019	100.125652	100.117075	99.0701231
99.8256087	100.235751	100.123818	99.2645407
100.03476	100.407729	100.037793	99.7299425
100.078064	100.350805	100.19084	99.5822261
100.077916	100.130742	99.8387715	99.4674494
100.03314	100.147083	99.9227529	99.2863541
99.9951394	100.258386	99.9758799	99.2597095
100.23699	100.339554	99.8107777	99.6016971
100.4837	100.137841	100.1277	99.6183866
100.55543	99.9409498	99.9811925	99.6787029
100.434358	99.8771946	100.023898	99.565683
100.183081	99.6182896	100.088877	99.8359352
100.004861	99.7543721	100.123614	99.9268489

99.9010212	99.8362091	99.9916136	100.084667
99.9198743	99.8730425	100.341638	99.9347544
100.089994	99.8849631	100.308945	100.251708
99.8447564	99.8884455	99.8921028	100.663381
99.9868912	99.9501916	99.9948829	100.703641
99.8942458	99.7950897	99.9010935	100.897912
99.6915749	99.6209684	100.049849	100.964524
99.8306166	99.7599976	100.03861	100.905525
99.855214	99.7819636	100.074573	100.888689
99.8731834	99.754506	100.42705	100.56544
99.900432	99.871837	100.116666	100.65123
99.9734878	99.9676037	99.9869139	100.45813
100.063924	99.9635856	99.870239	100.286404
100.202082	99.7849103	99.7455951	100.336766
100.101041	99.7042787	100.088059	100.408647
99.8256087	99.9327795	99.9623938	100.15201
100.086754	99.882954	100.008982	100.133857
100.054792	99.9384049	100.297093	100.216279
99.9129516	100.037788	100.027168	100.439245
100.108847	99.7731236	99.5780409	100.787674
100.044776	99.9276898	100.325496	100.708765
100.045954	100.226241	100.291781	100.191977
100.156569	100.397817	100.022059	99.5915956
99.9316575	100.22758	100.030437	99.3353977
99.9955813	100.08172	99.8526663	99.4025947
100.008101	100.259726	100.161415	99.5848613
100.047133	100.177085	100.246623	99.426165
100.38369	100.111321	100.253162	99.5833973
100.172624	100.113866	100.042289	99.8089978
100.280145	100.304194	99.8596136	99.7996283
100.167616	100.374914	100.066196	99.5561671
99.9832089	100.48086	100.297911	99.5371352
99.9287117	100.539793	100.154264	99.8394488
99.7703566	99.7518556	99.3411605	99.9895877
99.898032	99.8537113	99.2832731	100.058822
99.9331778	99.6814002	99.1342668	100.08654
99.8062799	99.7084263	99.631455	100.022849
99.7873074	100.170839	99.8134786	100.021371
99.935977	100.323466	99.7272907	99.9988271
100.107507	100.114755	99.7298635	100.092576
99.9226029	99.6771823	99.9337557	99.9467169
99.8316283	99.7146751	99.8252705	99.9688914
99.6728505	99.5512686	99.9168183	99.8426197
100.144985	99.9260413	99.9498355	100.003385
99.9715893	99.913075	100.229839	99.9119767
99.9132722	99.8409012	99.9286101	99.8666421

99.9367545	99.5722022	100.002149	99.9491807
99.8107897	99.3603674	100.15287	99.8983024
99.8450024	99.818093	99.8359903	99.8157639
99.8387819	99.7662279	99.3664594	99.8713234
99.6874686	99.5226803	99.3846832	100.029995
99.8047248	99.6048522	99.4785894	100.052785
99.8089236	99.5584548	99.6724049	100.114874
99.8454689	99.7373271	99.9084568	100.059068
99.92929	99.7879425	99.7386538	100.071141
99.7652247	99.5925108	100.060036	99.9005199
99.8294512	99.7331091	100.037095	100.018538
99.9176266	99.7715394	99.9343989	100.00166
99.8003704	100.015712	100.134003	99.7662407
99.8117228	100.426571	100.152441	99.7446821
99.7128171	100.154279	100.427299	99.8066477
99.8639749	100.011338	100.453241	99.8067708
100.138609	100.169901	100.404144	99.9601448
100.144208	100.296284	100.366625	99.9648261
100.160692	100.174588	100.314312	100.062025
99.9927389	99.7110821	100.276149	99.8025823
99.8575989	99.5403332	100.130787	99.8930052
99.9470183	99.7776319	100.149869	99.9546012
99.9625696	99.9554107	100.076759	99.9315643
99.9303785	99.8480873	100.253208	100.023096
99.9504396	100.017743	100.216546	99.9961168
99.9692566	100.249574	100.19382	99.9536156
100.076871	100.26379	100.06218	100.012748
100.166601	100.31253	100.151584	100.129287
100.128657	100.575918	100.119424	100.068184
100.294899	100.599351	100.045886	100.17031
100.218543	100.59607	100.079975	100.12941
100.111395	100.332527	100.325031	99.8790845
100.065052	100.527021	100.166806	99.8610985
100.19055	100.748698	99.9594834	100.048227
100.047324	100.461877	99.9309685	100.01361
99.9656798	100.146156	99.4841638	100.012871
99.910784	100.01618	99.3971183	100.042067
100.081847	100.103039	99.6211639	99.9458545
100.222275	100.015399	99.407195	100.061778
100.163336	99.6200056	100.112135	100.00745
99.9045635	99.6484377	100.082548	100.072003
99.9014533	99.9188552	100.108275	100.200246
100.044058	99.7476376	100.290942	100.134092
100.127101	99.8777692	100.197679	100.069662
100.182775	100.003527	99.8672924	100.174252
100.251978	99.9935285	100.139149	100.160701

100.201125	99.952755	100.128643	100.105388
100.229584	100.098352	100.268645	100.095286
100.195682	100.415948	100.197894	100.172774
100.202058	100.25551	99.9873551	100.198521
100.198481	99.8798001	99.9133879	100.111671
100.099109	100.021023	99.7579496	100.074467
100.166912	100.025554	99.8775835	100.132737
100.271728	100.002433	99.9665585	100.064735
100.267995	100.09351	100.034951	100.07767
100.185729	100.004151	100.243775	99.8949763
99.7681794	99.8063765	99.9834959	99.9833049
99.6400374	99.677026	100.013297	99.9775149
99.7513841	99.8155935	99.7671687	99.9149334
99.6614981	99.869802	99.9757776	100.025313
99.6728505	99.9835304	100.007723	99.9170276
99.782642	100.159591	99.9453332	99.9860151
100.060231	100.029615	99.9496211	99.8804396
99.8829473	99.8585541	100.250207	99.6859196
99.775644	99.8357459	100.144294	99.8112058
99.9224474	100.20755	100.108275	99.9809642
99.7770436	100.390641	100.071399	99.8151479
99.9179376	100.416417	100.009009	99.8353514
99.9607034	100.323466	100.150083	100.013733
99.9518392	99.9938409	99.9914287	100.04601
100.002536	100.003839	100.071185	99.892882
99.8230752	99.6053209	99.994859	100.052416
100.048101	99.7007715	100.070327	100.159839
100.314805	100.118661	100.097556	100.150723
100.281836	100.180056	100.182457	100.178318
100.03395	99.6698399	100.018228	100.138157
100.070028	99.6003218	99.368389	100.104279
100.055877	99.6906172	99.2822012	100.151215
100.073139	100.137564	99.924751	100.141237
100.343108	100.565451	99.8655772	100.098366
100.44699	100.214268	99.8745819	100.087525
100.169556	100.167402	100.274005	99.8231554
99.8516894	100.378768	99.9509075	99.8810556
100.061475	100.573418	99.8387775	100.012501
100.081381	100.071326	100.028305	100.021618
99.8790595	99.7413888	100.142579	100.024821
100.037526	100.044144	100.087479	100.005972
100.027407	99.912264	100.095395	100.095192
100.082296	100.064424	99.8556008	99.9765939
100.193939	100.188464	99.9507471	100.006644
99.9858903	99.7051142	100.009678	99.9777023
99.9056562	99.5893541	99.6477361	99.9616921

100.034559	99.7560424	99.484016	99.928317
99.9692526	99.9952176	99.6263068	99.8809022
100.30154	100.355308	99.8500292	100.111818
100.21633	100.673532	100.226757	100.209481
100.435108	100.439668	99.9108885	100.243718
100.399967	100.119414	100.275187	99.969574
100.162374	100.284384	100.267258	99.8443251
100.038602	100.528089	100.146183	99.7642742
99.9991072	100.552304	100.025321	99.8965429
100.266554	100.689622	100.232972	99.9461745
100.330773	100.740863	100.304974	100.050118
100.481134	100.493564	100.335618	99.9797959
100.387372	100.619635	100.379334	99.9572585
100.320355	100.847562	100.483266	99.9876779
100.279771	100.406393	100.345476	100.068098
100.296098	100.360776	100.253758	100.056522
100.409452	100.381866	100.445122	100.276723
100.216952	100.146752	100.504481	100.19815
99.9190285	99.8828943	100.252258	100.104429
99.8653836	99.8083766	99.825814	100.211944
99.7343034	99.65278	99.4670868	100.192362
99.7803292	99.7929107	99.6560936	100.120439
99.6985401	99.7491687	99.6792373	100.194332
99.9840244	99.8252486	99.5585901	100.199998
99.8922838	99.6852741	100.203399	99.926716
99.7750424	99.9735028	100.126039	100.098394
99.9739174	99.7179244	99.9670334	100.133617
100.173725	99.6354394	100.080395	100.001348
99.7196871	99.5529544	99.988677	99.9350905
99.6554687	99.784631	100.117253	100.108863
99.7133119	99.7393267	100.234472	100.230171
100.103598	99.8482131	100.143397	100.236205
99.7161108	99.377049	100.120467	100.229924
99.8582309	99.474375	100.140825	100.103074
99.8339741	99.831185	100.279688	99.9286865
99.7677343	99.632315	100.247972	99.8099648
100.158176	100.090981	100.183041	99.8802864
100.155066	100.11504	100.207685	100.001841
100.1815	100.242829	100.026178	100.11773
100.033782	99.8411832	99.4310855	100.097902
99.8761126	99.7583858	99.3325105	99.8679709
99.8353735	99.7826001	99.5142313	99.8587343
99.9967748	100.058644	99.6599509	99.8857053
99.7797072	99.9480387	99.5650189	99.8121816
100.009525	100.110509	99.4600151	99.8762223
99.6982291	99.7672904	99.7561686	99.8136594

99.9992627	100.046927	99.6314498	100.139528
99.8754906	99.5884167	99.723596	99.9943282
100.081674	99.7754139	99.4880875	100.053935
99.8658501	99.4121988	99.5069454	99.9460514
99.8919728	99.8610233	99.5750907	99.8880452
99.9193395	99.8719588	100.028536	99.7913683
99.84688	99.8613357	99.5095169	99.9019618
99.8622738	100.289695	99.6543792	100.068098
100.012013	100.411704	99.5313748	100.167731
100.044666	100.379054	99.55709	99.8825032
100.118214	100.793353	99.351154	99.8499902
99.9812255	100.528558	99.4640867	99.925115
100.06628	100.177841	99.5219459	99.8571333
99.9515265	99.8289979	99.51766	99.8405073
100.006104	99.6743386	99.6438789	99.8692025
100.164551	99.9444456	99.6230924	99.9826285
100.113239	99.6618408	99.9027453	100.148149
100.196738	99.9795955	100.024893	100.174997
99.9327119	100.239704	99.995963	100.232141
100.088049	100.107853	100.076109	100.157263
100.105464	99.9752212	99.9991774	100.069576
100.072033	99.6071633	100.172755	100.145193
100.340258	99.7493249	100.021892	100.072901
100.471804	100.123475	99.9918914	99.9099669
100.483622	100.474505	100.278616	99.9975303
100.072655	100.755392	100.190327	99.8587343
99.7952565	100.275323	100.108038	99.7985113
99.7817286	99.9908434	100.062394	99.8030681
99.7640025	99.7840061	100.160969	99.7368105
99.7588712	99.7416701	100.348904	99.7518355
99.7290167	99.7555738	100.089181	99.8471577
99.7571608	99.7251106	100.492052	100.01859
99.8047415	99.8568053	100.192684	100.047778
99.7520296	99.5895103	100.350404	100.012432
99.8106502	99.6546547	100.226328	100.000486
99.7943235	99.6015393	100.204471	100.07401
99.8530997	99.5804494	100.331761	100.082015
100.127389	99.8725837	100.296617	100.214776
100.050575	99.7782259	100.243472	100.100981
99.6809695	99.4462551	100.264687	99.9201888
99.83584	99.4978082	100.266401	99.8921094
99.8143821	99.8483694	100.046965	99.9275781
99.888863	99.9541313	100.310117	99.9912494
99.8224677	99.8321223	100.215614	100.076596
100.106086	100.063643	100.134611	99.9193267
100.043578	100.219083	100.281616	99.928317

100.12148	100.368275	100.277759	99.8283149
99.9439073	100.130662	100.275187	99.6843464
99.5285439	99.7889922	100.282662	99.7793887
99.6188633	99.954845	100.373371	99.8182852
99.594654	100.254596	100.377874	99.6737772
99.5879809	100.010805	100.368653	99.7992062
99.7931394	99.8120619	100.469656	99.7836968
99.7529457	99.7590638	100.300461	99.666638
99.5010757	99.5020232	100.392885	99.7323682
99.3851503	99.7620255	100.174583	99.7018418
99.4593301	99.5847937	100.137485	99.666638
99.8198317	99.684243	100.097384	99.959962
99.9196175	99.775275	100.426553	99.980395
100.033836	100.037148	100.065218	100.02003
100.000626	99.9737061	99.7757204	100.082067
99.6193289	99.3880773	99.9494187	99.8113921
99.6002408	99.4710037	99.8078868	99.8587818
99.9553108	99.5910288	100.328553	99.9541768
100.228907	99.8371581	100.085161	100.00846
100.240701	100.133012	100.003673	99.9252506
100.121827	100.345784	100.020185	99.8288709
100.028404	100.286083	99.743983	99.7381534
99.8580079	100.08204	99.8473442	99.7775423
99.8272807	99.9115113	99.6202497	99.8804457
99.988521	100.091393	99.5835801	99.8742912
100.194766	100.336275	99.9944516	100.01843
100.062235	100.218432	99.8790816	100.040586
100.029491	100.14065	99.6041666	100.00489
100.265687	100.222329	100.267866	100.070497
100.005592	100.113839	100.332842	100.081329
100.031198	100.272678	100.149922	100.306461
100.518643	100.302294	100.262933	100.310646
100.316278	100.150782	100.419476	99.8927547
100.015369	100.236982	100.559722	99.8346562
99.8114516	100.481552	100.244062	99.8581664
100.140916	100.699156	100.201388	99.9907345
100.35135	100.232617	100.646142	100.066804
99.9993841	99.8965471	100.499678	100.130073
100.062856	99.9617036	100.281375	100.023107
100.063477	100.106513	100.410899	99.771634
100.013196	100.145482	100.193668	99.9225426
100.000315	100.015013	99.6957334	99.8938625
100.002333	99.9984905	99.7257554	99.9317743
100.032284	100.050553	99.7032389	100.15346
100.111119	100.333314	99.6661404	100.238884
100.208112	100.650679	100.292526	100.172293

100.231235	100.413902	100.159572	100.101023
99.7423929	99.8965471	100.232054	99.9112183
100.031042	100.198948	100.228623	100.041571
100.162952	100.177593	100.407253	100.011168
99.9231869	99.8527457	100.336702	99.9458066
99.8784927	99.7356823	99.9955238	99.9684552
100.15302	100.136441	99.3140261	100.155552
99.9712951	99.7985006	99.9086747	100.086129
99.8916837	99.9168111	99.7718605	100.164046
100.233252	99.9755766	100.045274	100.495404
100.147278	99.7894598	100.191739	100.192479
100.00016	100.111501	100.522837	100.080837
99.8653018	100.175722	100.36608	99.9775639
99.8518004	100.254752	100.036911	99.9001401
99.9584145	100.311647	99.4064508	100.079852
100.118413	100.130986	99.4315405	100.138689
99.9550004	100.1433	99.9850161	100.083298
100.081479	100.140962	99.9425565	100.188294
99.8524212	99.9394132	100.050635	99.9118337
99.8780272	99.8249997	100.36608	100.08736
100.001712	99.560321	100.044846	100.102747
99.8603358	99.5914964	99.5464816	100.111363
100.060218	99.9010675	99.5769324	99.9991047
99.911703	99.9997375	100.065003	100.13118
100.063322	99.8703598	99.7915892	100.163553
100.077909	99.7179124	99.953922	99.9806411
100.149761	100.040733	100.310968	100.010675
99.8603358	99.6702141	100.18359	100.195434
99.9140308	99.7635843	99.3854354	100.051049
100.032129	99.7244592	99.2741399	99.9333745
100.026387	100.14065	100.188522	99.9251275
100.299673	100.20113	99.7941625	100.113948
99.939016	99.7913303	100.144132	99.839949
99.9563971	99.995217	100.188522	100.023353
100.046096	99.9565596	99.9882328	99.9651318
100.027473	100.079235	99.4825778	99.8628438
100.120586	100.338458	99.6208931	99.9656241
100.226424	100.410005	99.9219701	100.01843
100.013196	100.282498	100.031765	99.8852462
100.117017	100.199104	100.185091	100.016953
100.224562	99.8426138	100.114539	100.168846
99.9079785	99.7316295	99.7487007	99.9728865
100.002333	99.919461	99.5953744	100.260179
100.290051	100.087808	100.224763	100.28332
99.9506551	100.14595	100.408325	99.8964474
99.836592	100.16372	100.186163	99.7862817

99.7922082	100.042292	99.9131779	99.8765068
100.021421	99.8781536	99.7414097	99.9161419
99.7473589	99.369528	99.5535582	99.8052376
100.055252	99.709495	99.7090289	100.022738
99.9238076	99.8189205	100.170294	99.9001401
99.9716055	99.9635741	100.129765	99.86986
99.9745541	99.9172787	99.9140357	99.9214348
99.9438269	99.884077	99.3451203	99.919096
100.000781	99.7668577	99.5153875	100.024215
100.059287	99.7294473	100.076154	100.115056
99.8431099	99.8474459	100.185091	99.9309127
99.9974782	100.014796	100.107155	100.191915
100.119971	99.7766568	99.8125047	100.1998
100.255693	100.023083	99.4702471	100.229863
100.552352	100.578325	100.08378	100.202388
100.252425	100.562063	100.129672	99.8668919
100.257561	100.540642	100.073058	99.8963387
100.318418	100.166155	99.9632611	99.9584356
100.155146	100.253561	100.065981	99.9335475
100.098025	100.281393	99.2641633	100.046529
100.017089	100.170533	99.3188474	100.089159
99.9146751	100.032621	99.2249195	100.119099
99.9929645	100.268885	99.8283738	100.12834
100.167131	100.595525	99.9518954	100.435744
100.098959	100.312353	99.9847058	100.12181
99.8530398	100.310946	99.858182	100.037782
99.751715	100.002913	100.198295	100.165056
99.8080584	100.059047	100.08764	100.08534
99.9238582	99.8965865	100.246546	100.051951
99.8841687	99.8750085	100.098577	100.095443
99.8922623	99.6252983	99.8918502	99.9808595
99.8049455	99.7921366	99.5573126	99.8126803
99.8763865	99.6184184	99.5298634	99.8548175
100.064094	99.9789893	99.9491076	100.138443
100.132111	100.061392	99.9907103	100.234545
100.052577	100.06061	100.290722	99.9733438
99.8597325	99.9000264	100.254051	99.8670151
99.9802016	99.8018311	100.112731	100.251794
99.7409755	99.4778489	100.254266	100.188342
99.8838575	99.7455407	99.7979223	100.111707
99.8757639	99.6362437	99.6437348	100.122795
100.031098	100.108926	99.6859809	99.9820916
100.306745	100.307975	99.6576739	99.9658281
100.23079	100.099075	99.9553265	99.9717421
100.36231	100.249027	100.045394	100.07536
100.401221	100.702476	100.170846	100.098154

100.225654	100.863529	100.194435	99.9691547
100.092266	100.855555	100.023521	100.081521
100.027518	100.331586	100.093002	100.101234
100.274993	100.631801	99.971839	100.070309
100.241062	100.306255	100.171704	99.9981086
100.155458	100.268259	100.038746	100.012154
99.9973226	100.066708	100.331252	99.9526448
99.8279809	99.7727477	100.199582	100.003899
99.6107007	99.7813476	100.228961	99.9480861
99.6376273	99.6251419	100.033814	99.8755165
99.8061907	99.6076294	100.151331	100.140291
99.8718728	99.4326603	100.145756	100.150394
99.8337398	99.3591701	100.422821	100.08497
99.8001205	99.6720506	100.02588	100.072034
99.9447146	99.5860514	100.320316	100.14103
100.113745	99.768995	100.082065	100.024229
99.9764662	99.6804941	99.5884074	100.103944
99.83981	99.2126589	99.6525271	99.8986796
100.181606	99.7843185	99.667324	100.038151
100.258183	99.7272464	99.7488139	99.9880056
100.106897	99.7810349	100.19851	99.9523984
100.326823	100.040752	100.148758	100.068584
100.149699	99.6027822	100.064051	99.8636885
100.091955	99.734439	100.093002	99.8755165
99.8757639	99.6667343	100.136106	99.8434823
99.9907855	100.06749	100.074988	99.8782271
100.108142	100.063737	100.530903	99.9982318
100.0546	99.757737	100.249334	100.000573
99.9573218	99.7281845	100.104153	100.024352
99.7847117	99.4881688	100.276568	99.9265247
100.018023	99.6418727	100.230248	99.924923
99.9901629	99.4558018	100.327392	99.850382
99.9291501	99.9186335	100.007223	99.9250462
99.9844041	99.6460945	99.6664662	100.014249
99.8209769	99.436413	99.4837572	99.9494414
99.7506254	99.4457947	99.7955634	99.8920264
99.8359188	99.6095057	99.9360263	99.9257854
99.7865794	99.7802531	99.7503151	99.9345332
99.6731143	99.7069193	100.051399	99.9000349
100.019891	100.18742	99.8931369	99.9543697
100.136158	99.9151935	99.9615455	100.028048
100.224875	99.8817321	99.7243669	100.182428
100.067051	99.4251549	99.78913	100.013756
99.9979452	99.881263	99.6559583	99.8968315
99.9380219	100.246525	99.537369	99.9287424
99.910784	100.646968	99.7814099	99.7139905

100.08106	100.863998	99.7395927	99.7749786
99.9233913	100.406952	99.6029898	99.8522301
99.9339751	100.133788	100.147471	99.9021294
99.6796514	99.7208355	100.501952	99.9239373
99.7447109	99.6987885	100.391512	99.8749004
99.9521856	100.033872	100.291794	99.8132963
100.175847	100.383967	100.374142	99.9710028
99.9618355	100.277328	100.642201	100.021025
100.027518	100.554245	100.195722	100.174789
99.844168	100.30563	100.222742	99.8434823
100.221918	100.31376	100.188216	100.062423
100.072343	100.271855	100.387652	100.030636
100.076235	100.384592	100.192505	99.8692329
100.178805	100.640401	100.105868	100.021641
100.009619	100.41305	99.6750441	100.063409
100.203396	100.309539	99.5682494	100.027555
100.036701	100.255125	99.8182948	99.8974475
100.086351	100.338622	100.092573	99.9053329
100.064561	100.2115	100.422821	99.9522752
99.9332225	100.574545	100.222145	100.132397
99.8672794	100.583295	99.9963161	99.9405554
99.7723972	100.109355	100.050574	100.053728
99.6123626	100.442571	100.549365	99.9735258
99.5801027	99.7984507	100.249378	100.234375
100.073332	100.403781	100.335478	100.362269
99.6607525	100.479028	100.427443	100.110315
99.8666468	100.43703	99.6699333	99.7264781
99.5940187	100.276911	100.570105	99.877375
99.598921	100.369658	100.25273	99.8562126
99.6196369	100.300681	100.196587	99.9542036
99.5789957	99.9773812	100.235552	100.083478
100.239217	100.053357	99.7947884	100.238976
100.00217	99.977527	100.208737	100.363496
99.7735041	100.366158	100.067542	100.021371
99.9115577	99.9979429	99.8702042	100.129177
99.6425668	100.538088	100.103784	99.992848
99.6525294	100.242933	100.431423	100.001436
99.6628083	100.334367	100.275145	100.326232
99.6371901	100.305931	100.105669	99.8792152
99.7205283	100.021713	99.8710421	100.014317
99.6468365	99.8720937	100.147986	100.078111
99.6934869	100.201664	100.339039	100.186223
99.9365433	100.809182	99.998411	99.732152
99.8347031	100.467508	100.466617	99.9287475
99.705189	100.19656	100.234504	100.243116
99.5549589	100.367324	100.046803	100.046521

99.8674375	100.667292	100.421787	99.7928789
99.9259482	101.051256	100.598386	99.9281341
100.125833	100.708707	100.501811	100.109394
100.352444	101.085963	100.141701	100.00849
100.033007	100.84943	100.306359	99.8920967
100.279068	100.727081	100.394553	100.004196
100.18213	100.569587	100.4	100.017691
99.8612702	100.737143	100.340296	99.6705051
99.9775008	100.144937	100.008466	99.8628067
100.192093	100.451904	100.184646	99.7950258
99.991575	100.536339	100.063981	99.6103917
100.174065	100.203122	100.088281	99.8812088
100.218185	100.623398	99.9133588	99.6559368
99.8717072	100.418656	100.013704	99.7928789
99.9787659	100.67925	100.211042	99.8424111
100.055937	100.202977	99.9252996	99.8005464
99.8944789	100.282015	99.9755768	99.8195619
99.8698095	100.211726	100.103993	99.9292075
100.198576	100.50484	100.038214	100.139298
99.9969517	100.142021	99.7849424	99.7726366
100.055779	100.372866	100.134159	99.7195773
100.088513	100.632731	99.9393353	99.4808105
99.8162011	99.9999845	99.7633651	99.690594
99.9718079	100.241621	99.9588177	99.6554768
100.277328	100.109355	99.8932479	99.9474562
99.9822449	100.139833	100.019988	99.8198686
99.9912587	99.9957555	100.052668	100.029345
100.14481	100.036441	99.9485528	99.8637268
99.9890448	99.9579861	100.119076	99.9089652
100.091518	99.8630523	100.013075	99.8364304
100.139117	99.8114294	99.7176967	99.9331946
100.000114	100.0239	99.9707586	99.927674
100.113973	99.5175867	100.21565	100.065383
100.430247	99.6820803	99.5959839	100.069677
99.872656	99.9117588	99.6686764	99.7861314
100.010551	99.9600277	100.097499	99.8453247
100.324928	99.7236411	100.130179	100.205239
100.356239	99.8400116	99.8176226	100.015391
100.276696	99.5280863	99.7401119	100.480809
100.045342	99.5194824	99.8934574	100.359049
100.207432	99.6540813	99.7453491	100.254617
100.117294	99.6335197	99.7543571	99.9402487
100.046765	99.8933845	100.353075	99.9285941
100.006914	99.4016537	99.7709067	99.9031379
100.035853	99.2600551	99.6292926	100.087619
100.067323	99.3985913	99.6158853	99.9968351

100.171535	99.5301279	99.9776717	99.8756882
99.9711753	98.9615459	99.9521141	100.249403
100.230994	99.3475517	99.5961934	100.152639
100.240166	99.5785426	99.9340981	100.362116
100.024626	99.7891177	100.004486	100.145585
100.381699	99.9009675	99.4189663	100.020298
99.8574749	99.3145946	99.7499579	100.141751
100.200316	99.2275355	99.6931865	100.318258
99.9055485	99.395529	99.7516338	100.10234
100.122196	99.4594015	99.6605064	99.912799
100.036644	99.3068658	100.06021	99.9851805
100.305477	99.5107328	99.9248806	100.170581
100.264836	99.7194121	99.6646961	100.032106
100.123936	99.4869629	99.8012825	99.9442359
99.9414456	99.8308244	100.169144	100.006343
100.400517	99.7057043	99.5945175	100.161227
100.053248	99.5762094	99.5697979	100.266732
99.9181995	99.3891125	99.9035128	99.9804266
100.174223	99.6521856	99.890734	100.171655
100.070327	99.5396067	99.7512148	99.9821135
100.094048	99.6464983	100.134369	100.025512
100.01071	99.4322775	99.7878752	100.058789
100.035221	99.7544108	99.8297729	100.201711
99.7216352	99.6275407	100.041147	100.258911
99.9207296	99.5737303	100.168306	100.387266
100.008021	99.7920343	99.8708326	100.118442
99.7755599	99.4513809	99.809662	100.10142
100.253152	99.7995174	100.028731	100.037064
100.393352	99.9673203	99.9013724	100.070605
100.264095	100.037226	100.058106	99.8891142
100.393352	100.24665	100.050972	99.9218898
100.333085	99.8078709	99.7731743	100.036605
100.383836	99.5172567	99.682953	100.384271
100.236816	99.9904756	100.136157	100.332045
100.285664	100.16121	99.8806005	100.334189
100.282175	100.270098	99.5568531	100.287017
100.268853	100.246064	100.067967	99.9197456
100.299462	100.504876	100.023486	100.097714
100.30422	100.45197	99.8432531	100.02573
100.234913	100.312745	100.059574	100.124057
100.208428	100.218365	100.301074	100.401884
100.430464	99.9280442	99.8757747	100.292377
100.163386	99.8383539	99.8201732	100.298963
100.103753	100.14758	100.337792	100.123598
100.045548	100.522609	100.314082	100.049317
100.369087	100.443617	100.075101	100.048857

100.248394	100.218219	100.103636	100.066317
100.362108	100.11402	100.049083	100.200789
100.339587	100.007476	99.9978881	100.240763
100.211124	100.643075	100.232673	100.128193
99.8135203	99.9890101	99.6185392	100.144887
99.5997311	99.531912	100.020548	100.054218
99.4268601	100.121054	100.183366	100.001991
99.7086875	99.4546787	99.9559248	100.29881
99.6224106	99.9491478	99.9714512	100.064632
99.4725361	99.7325427	100.038383	100.126967
99.4022775	99.2990396	99.7966738	100.06984
99.7177275	99.6822751	100.056847	100.111039
99.6971099	100.109477	100.348702	100.230655
100.127226	100.468238	100.071954	99.8901863
99.7551565	100.328866	99.7213495	100.04656
100.106925	99.8301469	99.8176554	100.340009
100.075047	99.8827593	100.263726	100.253475
99.9128022	99.8681041	100.056637	100.226979
100.26013	99.6072401	99.9624291	100.417813
99.9188289	99.8918456	99.8510163	100.183329
100.010657	99.7857414	99.9861384	100.145499
100.01478	99.7385514	100.329189	100.094804
100.29978	100.004398	100.124408	99.9421066
100.105974	99.6774389	100.056847	100.016694
100.169571	99.9963378	99.9009527	99.838266
99.795123	99.8377676	99.9443848	100.003063
100.194947	99.7338617	99.8715784	100.154842
100.053795	99.8241382	0	99.9715128
99.983378	100.004984	100.058735	100.065704
99.8054318	99.5036273	100.000196	100.195582
99.8028943	99.4099801	100.123149	100.093273
100.405405	99.7565774	99.6273515	100.302485
99.9362746	99.6749475	99.878922	100.224988
100.056333	99.9021043	100.241905	100.137841
100.174805	99.9878377	100.388987	100.187158
100.285506	99.7504222	99.8894128	100.072903
99.9541961	99.9101647	100.014883	99.4392916
99.9070927	99.9975102	99.8361193	99.599494
99.9549891	99.829121	99.8323426	99.7529575
100.029371	100.060821	100.384161	99.6048545
100.139755	99.7439739	99.9225639	99.7384075
99.8744216	99.7200858	100.019919	99.5766735
100.23872	100.09233	99.8256285	99.6633605
99.8493633	100.26409	99.9290682	99.5063744
99.5675359	99.9642427	99.999147	99.9231151
99.9348472	99.7219909	100.104055	99.9975495

99.993211	100.07958	100.320167	99.8894205
99.9172429	99.9258459	99.836539	99.9107094
99.9553063	99.6025504	99.5467818	99.9404219
99.8146305	99.6255592	99.8117806	100.001838
99.936116	99.5941969	99.9061982	99.973657
99.7825938	99.6891631	100.16763	99.9728912
99.6428696	99.5244378	100.026423	99.6368643
100.481532	99.9262855	99.708131	99.8881953
100.058553	99.9405011	100.037544	99.8955468
100.062518	100.213969	99.7431704	99.8413292
100.109304	100.102735	100.025584	99.9989279
100.010974	100.162236	100.203719	99.6737752
100.17306	100.724998	100.073632	99.9577286
99.9261244	100.37972	99.7729644	99.77685
99.7814836	100.284167	100.120631	99.8117698
99.2233798	100.08383	100.19029	99.7861926
100.268853	100.410056	99.9273897	99.8495996
100.610313	99.9786049	99.6980598	100.265421
99.7513501	100.137615	99.9817322	99.7708769
99.8512664	100.255883	99.7970934	99.7970667
100.107718	99.983148	100.252396	99.8229503
99.9553063	100.161796	99.9479517	99.8719606
99.8266839	99.9613116	99.9573935	99.8906458
99.7253402	99.6632233	99.9737592	99.8987631
100.166241	99.8565264	99.9011626	100.024352
99.642711	100.321831	99.7878614	99.7621469
99.6669764	100.22965	100.032088	99.8071751
99.8517422	99.9639496	100.287226	100.005667
100.213978	100.359935	100.152104	100.125589
99.9992377	100.194917	99.9894955	99.9535934
100.041107	100.182313	99.9401885	99.88115
99.7549979	100.599256	99.9082963	99.5958182
99.9698972	100.150658	99.911024	99.9983153
100.171474	100.071373	100.363179	100.041046
99.5583503	99.7513963	99.7063744	100.129776
99.3282966	99.6846279	99.8808885	99.8561176
99.1881816	99.7050375	99.753874	99.8351258
99.6285656	100.094423	99.7388081	99.6566195
99.2691264	100.110167	99.5628292	99.3780578
99.3499135	99.9544714	99.8352721	99.6497244
99.3300323	99.5487586	99.2947805	99.4606457
99.5064384	99.7976094	100.161283	99.7134657
99.373266	100.3389	99.8859105	99.8023358
99.4908174	100.388174	99.7992812	99.7902311
99.1512594	99.7512505	99.7503168	99.8634723
99.2203702	99.7438156	99.664106	99.7718442

99.6919961	100.086113	99.7484336	99.8818592
99.0707879	100.234957	99.8331796	99.5662172
99.4237579	100.105939	99.7584775	99.7513121
99.5247417	100.179705	100.519518	99.7251108
99.6369284	100.367765	100.493153	99.9305846
99.8198037	100.46092	100.093486	99.9186331
99.7088793	100.894477	100.169862	99.7407397
99.6886826	100.737323	99.8277391	99.6805225
99.8982239	100.256679	99.8792145	99.7873198
100.264764	100.181601	100.274487	100.06864
99.9974721	100.059581	100.137429	100.045656
100.290167	100.003892	99.7262531	100.050253
99.8316377	100.034506	100.040546	100.050406
99.838896	99.8445513	100.247285	100.108784
99.8068652	99.9235655	100.454232	99.9883501
99.7336519	99.9135065	100.202087	100.189074
100.106661	99.6515353	99.9861411	100.119817
99.8387382	99.8428019	99.8308779	100.037229
100.038655	100.013951	100.143706	99.9422296
100.135063	100.175186	100.23745	100.001374
100.119284	100.259157	99.8721001	100.060825
100.459	99.9363943	99.9252494	99.9826808
99.9108469	100.233499	99.8528491	99.835279
100.186501	100.30639	100.102693	99.8349726
100.162044	99.8522778	100.188485	100.21359
99.9794843	100.028675	100.105204	100.15659
100.370481	100.076491	100.113365	100.105107
99.9850069	100.161774	99.9920001	99.9799228
99.8418939	100.270528	100.392713	99.9132702
99.9135293	100.249681	100.302736	100.156437
99.9947897	100.171833	100.056449	100.001068
100.33561	100.380594	100.031967	100.013326
99.8616173	100.355665	99.8344351	100.084269
99.6880514	100.009723	100.126548	99.863932
99.8467853	100.294874	100.274278	99.9580117
99.9474535	100.231312	100.040546	99.9935597
100.161886	99.9119029	100.006857	100.149695
100.140427	100.367765	99.8040939	100.030334
100.190446	99.7642252	99.8068142	100.227686
100.127647	100.065995	100.443142	99.8481499
100.132696	100.316158	100.100182	99.944528
99.9458757	100.04369	100.106669	100.0406
100.486139	100.249389	99.79405	100.210985
99.878185	100.055936	99.893862	99.9209314
99.8103365	99.9228365	100.051427	100.204703
100.116286	99.7369638	100.172582	100.279629

100.172616	100.123433	100.16484	100.305065
99.885601	99.9232739	99.9281789	100.121655
100.35202	100.304349	99.8639393	100.205929
100.061218	100.345314	99.8934435	100.065882
99.9979454	100.005058	99.8733556	100.022826
99.893017	100.128244	100.385389	99.7502396
100.218216	100.462669	100.377019	100.055156
100.082835	100.052146	99.8610098	99.9419232
100.036919	100.202447	100.118805	99.7539169
99.9203141	100.064391	99.7195571	99.8194969
99.9924229	100.072993	100.082814	99.9646003
100.087095	99.9452871	100.306502	99.9321168
100.15163	100.273589	100.002253	99.9894227
100.153839	100.252888	100.033222	99.9872775
100.057431	100.160899	100.156052	99.9229233
100.172616	100.020802	99.7768915	99.9414635
100.122597	99.8034407	99.669128	100.055616
100.123386	99.8783729	100.195809	100.041212
100.142952	99.8473212	100.156261	100.288976
100.275335	99.9256064	99.7662198	100.170381
100.389258	99.8572344	99.8476179	100.336935
100.170407	100.052583	99.6785442	99.8712868
99.8799206	99.9075294	99.8264836	100.290049
100.338608	99.3537016	99.9175072	100.300162
100.301055	99.7481891	100.093277	100.281315
100.2807	99.7955684	99.8658226	100.240557
100.117233	99.650369	99.9551721	100.019301
99.8915969	99.7658288	99.9620774	99.8438596
100.505547	100.009869	99.9737953	100.11951
100.296005	99.5340346	99.6105381	99.972568
99.7708897	99.9726942	99.88047	99.747788
99.9084801	99.7929443	99.9654254	100.030334
100.178138	99.3026777	100.146635	99.9089799
100.405825	99.6388522	100.02255	100.158123
100.21869	99.647016	99.6287428	100.271202
99.9824823	99.4099736	99.8189507	100.094381
100.638088	99.2621502	99.7132796	100.314411
100.090251	99.5802476	99.6308353	99.8674561
99.9280457	99.7228229	100.061471	100.031406
100.13806	99.498901	99.8302501	99.947286
99.9815356	99.5344719	100.576225	99.818884
100.393518	99.55182	100.205853	100.186929
100.284803	99.8272032	99.907254	100.015011
100.538069	99.6375596	100.258231	99.9968412
101.298471	99.8135768	100.06267	100.231838
100.544725	99.8402859	100.003561	99.9959215

100.606851	99.6673336	100.134145	100.351252
100.82524	99.3342729	99.7530832	100.515428
100.792434	99.7896408	100.779309	100.144615
100.7124	99.6028232	100.19514	100.121468
100.826508	99.3170506	99.8050652	100.218961
100.781024	99.3964481	99.7099045	100.190142
100.590685	98.9645784	100.007753	100.243028
100.376099	99.5537836	100.409985	100.020142
100.75218	99.9678473	99.8323138	100.39816
100.384816	99.8970609	99.8675275	100.304805
100.031557	100.102122	99.9362779	100.19796
99.915864	99.8078847	99.739878	100.389116
99.7597581	99.8560486	99.8790557	100.233984
100.381646	100.021995	99.9117541	100.656763
100.20985	99.651133	99.8113533	100.392795
99.8800468	99.5647299	99.959544	100.056319
99.80762	99.7397255	99.7836855	99.9557589
99.7142734	100.026958	99.8409076	100.060611
99.9825854	99.8732708	99.941518	100.131278
100.048514	99.5771358	100.206668	100.618134
100.202719	99.2723896	99.4845792	100.285643
100.308585	99.7408931	100.286318	100.225246
100.108421	99.4040376	100.560691	100.148447
100.106836	99.8881579	100.535329	100.306338
100.221103	99.5691084	99.9645745	100.122234
100.202719	99.9628849	99.7912313	100.296834
100.078151	99.8335721	99.8920512	100.142009
100.189089	99.8777953	99.7926985	100.283497
99.9586544	100.272302	99.9408892	99.8815656
100.185285	100.150578	100.167472	100.021828
99.8104727	100.315357	100.236223	99.9775264
100.355496	100.353159	99.8729772	100.354012
100.276572	99.9996646	99.7122102	100.13557
99.821408	99.9771881	99.747843	99.9526931
99.7204543	99.9516467	99.9867927	99.9861108
100.049307	99.9695987	100.292606	100.172207
99.873866	99.8655354	100.59318	99.9747672
100.162622	99.9167643	99.9798757	100.329638
99.9505718	100.112631	99.8962433	99.8731345
100.047247	100.156708	99.9792469	99.826687
99.7619768	100.096284	100.378754	99.7397705
100.51081	100.240046	99.8880687	99.8387971
99.9467682	100.039947	99.7415549	99.7037468
99.9088907	100.190715	99.9735875	99.8691489
100.147566	99.6775502	99.7472142	100.103226
99.742008	100.086068	100.330126	99.8716016

99.990668	100.309227	99.8876495	100.195814
99.9172903	100.028417	99.9316666	100.021675
99.9001742	100.328347	99.7384108	99.9151365
99.849935	99.8509403	100.189481	100.209917
99.6943045	100.174368	100.115909	99.963117
99.9705407	100.56581	100.028504	100.054939
100.321422	100.006086	99.6933458	100.157645
100.134412	100.078624	99.8402788	99.9327651
99.7893944	99.7861379	99.7013107	99.7465153
99.8290152	100.310687	99.9012738	99.5771276
99.6183911	100.376511	99.7994059	99.4184703
99.8906652	100.583178	100.033115	99.5990484
99.9987507	100.693079	100.143577	99.7123312
100.070227	100.259312	100.19493	100.12576
100.050733	99.9338406	99.8163838	100.146914
100.154698	100.151016	99.881571	99.7399238
100.049465	99.7133083	99.9060948	100.172514
99.9900341	99.6705446	99.5093126	99.9300059
99.9312369	100.028271	100.179	99.7163168
99.7256842	99.8983745	99.9467581	99.7618445
99.6744941	99.7667264	100.079228	99.7282736
99.6727508	100.034693	100.194511	99.8673094
99.8451805	99.5216743	99.7400877	99.8783465
99.7740216	99.2084629	99.6975379	99.9828916
99.7267936	99.8124092	99.5478799	99.6102387
99.8439126	100.199618	100.066862	99.710645
99.9126943	100.200785	99.9853254	99.618823
99.7806779	99.8881579	99.7606289	99.486532
99.9255315	99.9347163	99.9781989	99.6202027
99.958179	99.9315054	100.00922	99.8389504
100.05945	100.029147	99.7855719	99.9349112
99.9120604	100.022433	99.8752828	99.9119174
99.7485058	100.380451	100.108992	99.8967415
99.5795627	99.9704744	100.071683	99.9785995
99.6391524	100.158022	100.768619	100.084831
99.8700624	100.228808	100.698192	100.1998
99.8672097	100.427594	100.484814	99.9936221
99.7004854	100.191736	100.74703	99.9621972
99.8028655	100.086214	100.394474	99.9871838
99.9583375	100.37797	100.285689	100.371027
99.8816316	100.447297	99.8901648	100.002666
99.7817872	100.267631	99.9972729	99.9689421
99.8806807	100.005795	99.8685755	100.057698
99.8806807	100.506407	100.172293	99.9543793
100.000494	100.313314	100.022845	99.987797
100.265795	100.327617	99.9421468	100.067815

100.034885	99.992513	99.9742164	99.961584
99.6984251	99.9449329	100.07839	99.9755336
99.816812	100.161816	99.7660787	99.6589856
99.7817872	100.396944	99.9890983	99.620356
100.045028	100.676878	99.9704435	99.3717163
100.336657	99.7920954	99.9391778	99.9631501
100.195625	99.7298825	100.093238	100.006658
100.198732	99.6538757	99.9833755	99.771944
99.8093417	99.5300129	100.068824	99.530074
100.132876	99.8029334	99.9905313	99.3108167
99.8618402	99.6213617	100.150485	99.4085665
99.5036698	99.4402123	100.050724	99.9391062
99.8590444	99.6807596	99.9454918	99.8997486
99.6706399	99.4594955	100.030099	99.6646052
99.6939381	99.6599281	100.081242	100.321805
100.036111	99.7504325	99.8248952	100.63781
99.8848277	99.4704742	99.8286836	100.395367
100.186927	100.095419	99.8272103	100.519737
100.434043	100.099501	99.941072	100.055318
100.229951	100.274035	99.974536	99.6312586
99.9929312	100.60959	100.106498	99.9770326
99.610686	100.06023	100.182476	99.6948032
99.5676621	100.046859	99.980429	100.10956
99.962333	99.9894315	100.047357	99.994493
99.966682	99.9177881	100.002317	99.3932529
100.224981	100.229416	99.8888766	99.2726041
99.776103	99.7446616	100.221622	99.4834174
99.6673782	99.8453002	100.207731	99.7821054
99.8536082	99.998862	100.147538	99.9548492
99.3210121	99.5826546	99.8905603	99.9834729
99.3811214	99.6265696	100.044831	100.415833
99.8236312	99.6841377	100.045042	100.169527
100.013123	100.175648	100.051987	99.5617031
100.073543	100.63014	100.208994	99.3604788
99.8674318	100.234905	99.9459127	99.358332
99.9155813	99.945798	99.6798847	99.2408318
99.9848545	100.214918	99.9627499	99.8307656
99.8505018	99.9198994	100.000634	100.147343
99.9166686	99.7476174	99.8768801	100.33125
100.082862	99.892171	99.916658	100.647971
100.132565	99.6707661	99.8520452	100.546214
100.137069	99.7113031	99.9332848	100.169956
100.219079	99.6921606	99.8844569	100.043583
100.08659	99.2879172	100.009263	99.7053939
100.196247	99.3929192	99.9419139	99.9137742
100.230573	99.7715455	100.008842	100.136609

100.009085	99.4411976	99.8608848	100.091813
99.9370156	99.2513214	99.7779615	100.093245
99.9429178	99.4252924	99.9680115	100.144052
99.9300262	99.3526637	100.304545	99.9735977
99.5814856	99.6659805	100.471655	99.9575685
99.9452477	100.398461	100.164796	100.340267
99.8153992	100.381007	100.551	100.346135
99.7568432	100.369606	100.338851	100.435727
100.01654	100.106257	100.310017	100.264128
99.5619152	99.8644426	100.266662	100.031418
99.7936543	100.031235	100.052198	100.141332
99.6799592	99.4668146	100.171952	99.8966
99.7928777	99.9785935	100.015577	99.8990331
100.032693	100.408032	100.202891	100.028412
99.7416217	100.474327	100.253613	100.063619
99.8682084	100.453636	100.267714	100.130455
100.142816	100.605931	100.024416	99.7924099
99.9472668	100.377911	100.003159	99.6946601
99.9495966	100.81129	100.145223	99.7823916
100.302952	100.762167	99.9747464	100.18985
100.257288	100.040666	100.274028	99.8976019
99.8581125	99.4765266	100.416092	99.6895078
99.9607798	100.043903	100.253823	99.8639691
100.085347	100.287406	100.187948	100.205449
99.9965036	100.253485	100.051356	100.013385
100.061583	100.769205	100.179319	99.6225284
100.294565	100.902357	100.094712	99.6282532
100.053817	100.496847	99.9471755	99.5831709
99.9690118	100.287265	100.041464	99.7424616
100.168755	100.299511	99.8718289	99.913488
100.154465	100.007166	99.9673801	100.161655
99.8975641	100.04897	99.974536	100.141619
100.060807	100.404091	100.019786	100.272858
100.386826	100.585522	99.793115	100.095391
100.080222	100.213088	100.248562	99.9973554
99.9965036	100.68236	99.9793767	99.518052
100.268937	100.613532	99.6849359	99.7770962
100.547894	100.118362	100.065246	99.948552
100.281828	99.5701275	99.9886371	100.070489
99.9713416	99.2711676	99.8960324	100.286884
100.196091	99.5936333	99.8335243	100.142191
100.153378	99.7556404	99.6300045	100.232356
99.9850099	99.4371157	100.137646	100.066768
99.8495699	99.4938393	99.9191836	100.172246
100.132565	99.8058893	99.6891452	100.43172
100.261637	99.5756169	100.007369	100.045729

99.8132247	99.3737767	99.946965	99.7351626
100.073698	99.8322101	99.6592591	99.9753151
100.420841	100.329632	99.5565521	100.212176
100.152446	100.475453	99.8478359	100.132173
100.020423	100.128355	99.7722789	100.163659
100.122624	99.8430481	99.7396568	100.364597
100.30435	99.6210802	99.789958	100.271284
99.7941202	99.5066478	99.7960615	100.113854
100.0504	100.089085	99.9360208	99.8938808
100.316931	100.48432	99.992636	99.9233632
100.620894	100.590307	99.6596801	100.108129
100.196557	100.18902	99.9330743	99.9249375
100.080998	100.324143	99.5491858	100.074067
99.9664383	99.9449935	100.128431	100.114173
99.8008724	99.8677657	100.078476	99.9632247
100.117729	100.659597	99.9682378	99.9975962
100.109349	100.3075	100.162578	99.9953047
99.8601472	99.8386471	100.181969	100.404038
100.125177	100.279507	99.9361992	100.532215
100.184141	100.536089	99.6347827	100.516032
100.15559	100.172738	99.8845579	100.183345
100.404482	100.156139	99.8556809	100.101856
100.235347	99.9120768	100.103981	100.043281
99.9608522	99.8582002	100.136652	100.329996
100.135108	100.264455	99.9893159	100.217716
99.8756641	100.188493	100.147612	99.9320041
99.7375632	99.8690318	99.8986802	100.260967
100.037661	100.092134	99.9003664	100.137373
100.024782	99.8570748	99.9300865	99.8172895
99.8922673	99.7051513	99.8171081	100.23748
99.9881621	99.6969925	100.125269	100.444997
99.9484387	99.5063847	100.249419	100.152411
100.161952	99.7621226	100.039693	100.141097
100.351414	99.8205006	99.8346029	100.030822
100.07366	99.8981504	99.7070805	100.130499
100.255364	100.371223	100.070677	100.413204
100.378258	100.247856	100.115363	100.371672
100.087626	100.037414	100.197778	100.310233
99.7349253	100.288931	100.330359	100.031395
99.6826332	100.460408	99.7679962	100.242492
99.8337684	100.55747	99.8860334	100.09269
99.3781905	100.04501	100.367878	100.259248
99.7617697	99.778581	100.153725	100.412488
99.8471129	99.8859122	99.9045821	100.427382
99.7100982	99.7299092	99.658601	99.8837409
99.7034259	100.016735	99.5007262	99.8916176

99.8272512	99.7157016	99.9020527	99.8970598
99.9450249	99.8760653	99.896994	100.354486
99.6196344	99.617936	100.016928	100.501567
99.567187	99.4405512	100.132225	100.855449
99.7952863	99.3941301	99.8769698	100.523765
99.6391857	99.8150145	99.9009988	100.538803
100.005696	99.9845218	99.7013894	100.309087
100.116332	99.7584652	99.7861233	99.9208333
100.271812	100.109718	99.7536631	100.220867
99.9153875	99.938804	100.004492	99.8760073
99.9430077	100.342245	100.007865	99.931288
100.061092	100.321004	100.048967	100.185064
99.9881621	100.230413	99.9189151	99.4747211
100.569738	100.858223	99.5694407	98.9296479
100.464998	100.656361	99.7865449	99.4330458
100.249157	100.044869	100.089226	99.9496194
100.137901	99.9188289	100.061824	99.9828451
100.483463	100.361376	99.9853111	99.5839935
100.464843	100.154029	99.9062683	99.3002862
100.483774	99.849338	99.8453527	99.4575354
100.362741	100.262345	100.192509	99.5731093
100.318208	100.349982	99.9302973	99.7988148
99.7983897	99.6795494	99.9197583	100.107585
100.021679	99.4653091	99.990159	100.046146
100.360879	99.8641083	100.211057	100.17375
100.096005	99.4961158	100.17143	100.032254
99.4011556	99.1033654	100.137706	100.238196
99.5943417	99.4430832	100.11452	100.452731
99.8246133	99.7213284	100.149298	100.399885
99.8106481	99.5298766	100.017139	100.100424
99.682478	99.5643407	100.091966	99.9384487
99.9065429	99.9871945	99.8390293	100.050156
99.9349389	100.105217	99.8750728	99.9448933
99.7065293	99.7465083	100.223493	99.965373
100.017489	100.079896	99.9123809	99.9195444
100.118815	100.428476	100.140235	99.3224844
100.367241	100.693639	100.13349	99.1921595
100.404948	100.522022	99.948846	99.3011455
100.089177	100.231819	99.9163858	99.3216251
100.050851	100.442262	100.231925	99.4857486
99.8922673	100.005341	100.167426	99.9663755
100.400137	99.9900079	99.7203597	99.8508016
100.350948	99.4536335	99.8955185	99.5534889
100.277708	99.9061686	99.7772705	99.3744712
100.64515	100.447185	99.7989809	99.3976719
100.742286	100.551984	99.8917244	99.7098788

100.37531	100.040649	100.186817	99.977403
99.9540248	99.6293302	100.274502	99.9042205
99.867285	100.125754	100.056766	99.6858188
99.8795434	100.246168	99.9985903	99.7358006
99.6034967	99.8867562	100.298531	99.923268
99.3418808	100.0135	100.310967	100.266266
99.4081382	100.028551	100.213587	100.579475
99.9098014	99.950339	99.8805531	100.429817
99.5659457	99.4828928	100.011026	100.294479
99.6155999	99.7058547	99.9170181	100.394156
99.9318355	100.174426	99.9933207	100.294336
99.7018742	99.6545101	100.28188	100.18578
99.4726888	99.6139972	99.9389393	100.333147
99.6169965	100.008858	100.126956	100.404897
100.042006	100.200872	100.106721	99.939308
100.224175	100.201575	99.9328267	99.2969923
100.104229	100.499936	100.160891	99.5022182
100.112608	100.73598	100.02009	99.439204
99.9516972	99.8849275	100.48444	99.7217656
100.055506	99.7415848	100.007443	100.235189
100.144066	99.8827676	99.8752774	100.486764
100.290276	99.9816033	99.5462185	100.347842
100.034602	99.7383804	99.5963568	100.32865
99.6601605	99.6620394	99.9405836	100.532308
99.8919575	99.983431	99.9180424	100.336671
99.9324251	99.5914626	100.151459	99.9326493
99.8206354	99.5352261	100.061927	99.5400854
100.274771	99.8775657	99.3958036	99.3934289
100.273221	99.6891735	100.0377	99.3993009
100.072589	99.511185	99.8470483	99.7583515
100.13988	99.9720431	99.9079305	99.8398432
99.81955	99.818658	99.9690234	99.9544186
99.462319	99.4858785	100.041492	100.1618
99.7179935	100.083954	99.8685361	100.054672
100.213217	100.274033	99.7758434	99.4796469
100.469667	100.316773	99.6787268	98.9758016
100.668594	100.775241	99.564757	99.2891653
100.39757	100.467768	99.7033746	99.1997965
100.182673	100.475781	99.5936181	99.6563794
100.330899	100.38735	99.6374365	99.7421677
99.9612641	99.7750747	99.9985165	99.7573489
99.9697917	99.9017474	100.296608	99.9521271
99.9007953	100.330973	100.168102	100.53059
99.9294792	100.499963	99.8946586	100.391237
99.93196	99.9577028	100.260584	100.331515
100.004833	99.7475188	100.157358	100.295567

99.9131991	100.049228	100.198016	100.123274
100.119568	99.93802	100.103849	100.203047
99.8756775	99.8613978	100.244995	100.109525
99.9908783	100.191506	100.292605	100.181421
100.191511	100.667829	100.173579	100.099356
100.055223	100.455536	100.243731	100.119407
100.202519	100.346297	100.21508	100.155355
100.363304	100.286264	99.9622821	100.294851
99.7755164	99.9124324	100.326522	100.116972
99.7945873	99.8630848	100.07878	100.035337
99.9685513	99.970356	100.022322	100.158363
99.9347508	99.9215708	100.049708	100.079449
99.8962989	99.602991	100.061295	100.231118
100.129801	99.9238203	99.8167125	99.9842082
100.080031	100.081564	99.7362384	99.7218306
99.9344407	99.5331172	99.9865086	99.6701284
99.8104022	99.1734848	100.013895	100.048943
99.9887076	99.7279767	99.9696554	100.321346
100.407803	99.8564771	100.070353	100.35443
99.8997099	99.5790905	100.335791	100.234699
99.9299443	100.035169	100.20223	99.8613261
100.135228	99.6223926	99.8668508	99.7288483
100.406717	99.3815598	99.7168572	99.7398762
99.8545909	99.5980704	100.105956	99.842994
99.9656054	100.155655	100.039386	99.8441398
100.25353	100.31846	100.041492	100.054958
100.054758	100.20683	100.136502	100.066702
99.6711689	99.6150819	100.052025	99.835117
99.6086845	99.5165274	99.7244412	99.5471031
99.9069972	100.267566	100.04718	99.6732792
99.8890116	100.25182	99.8580028	99.6908952
99.8280777	100.187148	100.080255	99.8911157
99.9099431	99.8837518	100.158622	100.026315
100.017392	99.8874071	99.87612	99.9090181
100.265934	100.081564	99.8160805	99.6218635
100.139259	100.103918	99.9664954	99.7917216
100.010879	100.292872	100.026324	99.6629675
99.7321029	99.8931714	100.066772	99.5682996
99.6693084	99.9162283	100.170419	99.7199687
99.7472976	100.077908	100.032855	100.160798
99.973823	99.8954208	99.9584901	100.411145
99.7603216	99.6056623	100.271749	100.389375
99.6733396	99.8105037	100.2846	100.36689
99.8704058	100.045853	99.9129864	99.9163223
99.8873061	100.087609	99.7383451	99.5397989
99.9145946	99.7433011	99.87612	99.6331779

99.9088578	99.8539464	99.786798	99.9582855
100.027935	99.8968267	100.128076	100.421457
100.083752	99.7610156	100.206232	100.483041
99.7834238	99.5793717	100.097318	100.3709
99.7116365	99.9191808	99.9595434	100.541617
100.064371	100.41603	99.9018212	100.535459
99.6122506	99.8398873	100.027588	100.720355
99.8023397	99.8997792	99.978714	101.100602
100.070263	100.23298	99.9403729	100.830347
99.9659155	100.200785	100.086153	100.379493
99.7589262	99.8370755	100.049287	100.032473
99.9328903	99.9974901	99.7655209	100.031041
100.147167	100.42784	99.7151719	99.9779065
99.7372195	100.143283	100.111222	100.105801
99.9786295	100.47536	100.190432	100.123131
100.49556	100.350093	100.093526	100.46743
100.417416	100.057241	99.969234	100.373192
100.027935	100.077908	99.9778713	99.9956657
100.016771	100.215266	99.8912879	99.6760004
100.334	100.125288	99.6222686	99.93179
100.230738	99.7583444	100.182006	99.8335416
100.159106	100.057944	100.060663	100.073147
100.233064	100.300464	100.121756	100.084175
100.078015	100.231574	100.165785	99.3934289
99.9446739	99.9166501	100.03433	99.694046
99.9888626	100.195864	100.10722	100.134302
100.037548	100.380039	99.8588455	100.249737
99.8852905	100.00466	99.9648101	100.036483
99.6150415	99.5407091	100.21803	100.004832
100.216906	99.9795188	99.8381641	100.170531
100.425379	100.345038	99.5821812	100.104673
100.006105	100.248382	100.025787	99.8771753
100.281637	99.957008	100.054393	99.4545372
100.313148	100.203923	99.9933945	99.6095904
100.21287	100.108252	99.9742536	99.7510424
100.153262	99.9523652	99.8476293	99.5912646
100.12827	99.8498004	99.8552016	99.7322872
100.089929	100.210395	100.221192	99.8128919
100.163197	100.40047	100.420383	100.20031
100.410477	100.91048	100.331199	100.158218
99.8257291	100.711401	100.128012	100.483644
99.3055568	100.425373	100.154725	100.960973
99.7285557	100.605318	100.034621	100.51371
99.6225344	100.546509	99.8021961	100.045973
99.8038418	100.60363	100.037145	100.605625
99.8025999	100.644572	99.8249127	100.435825

99.6281226	99.9634799	99.7746416	99.9786829
99.8202961	99.7359803	99.8875938	100.211907
99.8699693	99.6383398	99.9923428	100.194583
99.9415299	100.012159	100.204154	100.103957
99.5900915	99.9276033	100.130956	100.163659
99.9975675	100.139064	99.8667702	100.413347
99.9593812	99.8071706	100.165242	99.8413828
100.450991	100.131467	99.811451	99.6326408
100.334725	100.228263	100.254215	99.2593963
100.428949	100.726595	100.204996	99.6598431
100.23662	100.354745	100.163769	100.014762
99.8013581	99.5432317	100.104033	100.091501
100.131064	99.8364346	99.7088054	100.013187
100.294365	99.9209908	100.006856	99.6473873
100.220321	99.849519	99.8055615	99.6596999
100.597372	100.303955	99.8533085	100.137172
100.100174	100.012019	100.210254	99.6956356
100.03327	100.02651	99.8960074	99.9381658
100.017747	100.132733	100.069117	100.107106
99.4665292	99.5647577	100.23844	100.020918
99.6247076	99.9700924	99.9555334	100.138604
99.7311946	99.727398	100.224768	100.354361
99.4974198	99.4837189	100.280718	100.446992
99.3650095	100.003296	100.252112	100.250849
99.5813987	100.178317	100.030835	100.061292
99.4952466	100.030449	99.8554119	100.185993
99.9615544	100.287776	99.9220894	100.340903
99.4642008	99.9032635	100.279035	100.041535
99.9631067	100.030731	100.191324	100.164804
99.9843731	99.9174734	100.290604	100.383997
99.8951164	100.091229	99.8667702	100.709137
99.7035639	100.033545	99.7813725	100.553797
99.8165706	100.09348	99.8211266	100.468181
100.243605	100.334204	99.965209	100.108968
99.9728861	100.160731	99.9845602	100.011469
99.9404433	100.118101	100.003491	99.9471855
100.281947	100.050568	99.6808303	100.071314
99.7411293	99.8555688	99.9557437	100.15922
99.8024447	100.045504	100.311848	100.152921
100.22327	100.054789	100.141473	99.8886289
100.301816	99.8284152	100.142946	99.2210268
100.458132	100.319291	99.9605815	99.1492985
100.479243	100.248663	100.206468	99.706946
100.357388	99.86598	100.262629	99.7123865
99.988409	99.8910233	100.198686	100.003881
99.9050511	99.9834582	100.118126	99.9650817

100.059193	100.170438	99.7855793	99.913111
99.9514645	99.8517701	99.8259644	99.6711535
100.512307	100.084757	99.6414969	99.405573
100.565085	100.294529	100.007908	99.4734356
100.426931	100.133296	100.051869	99.3825226
100.227151	100.250492	99.9957082	99.4034255
99.8845608	100.201531	100.248746	99.9520532
100.163973	99.8246165	100.032307	100.031656
99.649389	99.5323984	99.9917117	100.145762
99.411423	99.8631662	100.075006	100.466034
100.047551	99.9944322	99.9366028	100.183273
99.928025	99.4364462	100.31942	99.9778239
100.067265	99.5155153	99.8669806	99.6240506
100.430501	100.400611	99.9700468	99.6727284
100.159006	100.426358	99.9921324	99.8232002
99.8280575	100.075893	100.259684	100.111974
99.6284331	100.151586	100.043876	100.085058
100.231342	99.6473441	99.5855466	99.7755245
100.268442	99.3706022	99.6730477	99.723697
99.6380572	99.6196277	100.140632	99.9649386
99.6458187	99.4669765	100.10214	100.137459
99.7017011	99.5959914	99.8648772	100.136027
100.021007	100.265968	99.8875938	100.221929
100.017282	99.656067	100.094988	100.247699
100.0297	99.6391839	100.199737	100.229517
99.7878532	100.184367	100.21972	100.369824
99.835198	99.8499411	100.237598	100.132448
99.5713088	99.2804184	100.027259	99.8617129
100.387037	99.9232418	99.5131899	99.626914
100.612429	99.7243028	99.60006	99.4127315
100.249349	99.9140968	99.718481	99.8461074
100.236775	99.8405147	99.9092587	99.7242696
100.44649	100.086023	99.4414642	99.912252
100.320444	99.7926793	99.7550801	100.181269
100.132617	99.4595198	99.6987091	100.063726
100.108711	99.659725	99.8211266	99.956062
100.084496	99.5931775	99.7744313	99.628632
99.7604204	99.7164764	99.8721793	
99.5053319	99.5342748	99.9578124	
99.8442724	99.7549143	99.9382852	
99.9491338	99.849647	99.6607838	
99.4274295	99.4375748	99.9533337	
99.4514138	99.3774967	100.046133	
99.2028327	99.524287	100.073184	
99.8853617	99.7674747	99.6507515	
99.4605241	99.5831545	99.6000524	

99.2610271	99.4334889	99.936852
99.544562	99.718141	99.7974743
99.532105	99.5504671	99.9556626
99.4999401	99.4902377	99.5901992
99.6966483	99.9513409	99.6407192
99.3517582	99.5957149	100.032338
99.7851484	99.961026	100.079813
99.7524256	99.6538257	99.8042819
99.6654129	99.6261322	99.9630077
99.5681744	99.4109407	100.070139
99.6964623	99.5488025	99.7752599
99.9898513	100.084814	99.5418291
101.192597	101.364462	99.9146375
99.8844321	100.053337	100.086441
98.928594	99.4599717	100.123704
98.8966149	99.1249266	100.17709
98.8464153	99.1662397	100.213099
98.5199318	98.9304672	100.397622
99.0301088	99.4297056	100.47591
99.3266584	99.4056441	100.233701
99.965867	99.8342113	100.086441
100.439417	100.098131	100.052045
100.276919	100.155788	99.8960061
100.300903	100.258087	100.098802
100.472511	100.071648	100.012811
100.48125	100.211326	100.021589
100.325259	100.051975	100.201634
100.161646	99.8811237	100.244629
99.8215896	99.7061859	100.44241
99.9517368	100.058785	100.219907
100.02369	99.9673819	100.224027
99.9820425	99.9549728	99.975369
100.075005	99.9752511	100.195005
100.01179	99.8891442	100.461579
99.860076	100.007333	100.420374
100.374529	100.327548	100.397264
99.7819876	100.074978	100.401564
100.00175	100.296071	99.8908108
100.181539	100.339957	100.184435
100.28417	100.203457	100.224386
100.586111	100.392317	100.138036
100.39275	100.213747	100.071393
100.151234	100.067714	100.238001
100.301647	100.155031	100.247138
100.100105	100.099796	100.369675
100.389589	100.455876	100.019977

100.298114	100.200884	99.6108013
99.9770225	100.060753	100.005287
99.7159844	99.8997373	99.9476009
99.8431568	99.9817583	100.007616
100.386242	100.424248	100.265948
100.410413	100.2083	99.9046052
100.371368	100.266864	99.7637943
100.296999	100.20497	99.913921
99.9710729	100.004306	100.008511
100.28826	100.337384	100.175299
100.388659	100.087689	99.8033862
100.447598	100.21632	99.6249541
100.456894	100.280333	99.8752248
100.320425	100.008846	100.019081
100.10122	100.012176	99.9540503
100.406694	100.097526	99.844053
100.460984	100.143984	99.7833215
100.434025	100.208754	99.9658741
100.082628	100.014294	100.034488
99.9569426	100.209056	99.8429781
99.7165422	100.05591	99.9106963
99.7085474	100.060299	99.4902343
99.9528523	100.200884	99.7322642
99.9926402	99.9870548	100.117971
100.122787	100.027914	100.201275
100.63817	100.238717	99.7915624
100.643748	100.413049	99.6804902
100.73169	100.303184	99.9413307
100.340691	100.063477	100.072468
100.358168	100.236447	100.021052
100.27506	100.230848	100.572113
100.110702	100.077096	100.39189
100.356495	100.244922	99.3838199
100.011604	100.122495	99.5608189
99.9628922	100.128246	99.6856855
100.256467	100.304546	99.607935
100.059201	100.165322	99.9003057
100.12037	100.166684	99.8877652
99.9736759	99.9436231	99.8110896
99.9561989	100.027006	100.127645
99.9974742	100.023223	99.8617887
99.7782692	99.9554268	99.9929255
99.534708	99.8432911	99.8591014
99.8569152	99.8941381	99.9012014
99.7167281	99.8355733	100.276697
100.062617	100.0218	100.206075

100.139752	99.9006765	100.404828
100.178692	99.9984547	100.455638
100.301475	100.032109	100.251499
100.220613	100.189766	100.400698
100.112177	100.493864	100.206255
100.008212	100.361826	100.367842
100.172543	100.333629	100.524043
100.260299	100.350456	100.139286
99.8843112	100.139892	99.9934979
99.9554843	100.329688	99.64806
99.803077	100.205078	99.8182654
99.7926433	100.289667	99.903009
99.6551414	99.9842049	100.260117
99.7244513	100.163086	100.444147
99.898285	100.31377	100.077882
100.086279	100.323624	100.084705
99.898285	100.247372	100.101761
100.383827	100.442474	99.9263494
99.8915776	100.004973	99.8990591
99.9066692	100.130493	100.225106
100.207012	100.34318	100.378435
100.59362	100.250859	100.210923
99.9375979	99.5256327	100.350965
99.6873742	99.2664067	100.586344
99.8507742	99.4172429	99.9412513
99.6646436	99.4530191	100.07483
99.6987396	99.4369501	100.226184
99.4473981	99.305518	100.175373
100.019391	99.8188157	100.285253
99.8258077	99.6549424	99.8846957
99.9713212	99.7177024	99.805877
100.099694	99.804869	100.044129
99.8919502	99.90386	100.20823
100.211484	99.9176551	100.280944
100.061685	99.9675295	100.146108
99.9992688	100.058183	100.005886
99.9444916	99.9963324	100.110559
99.6033453	99.7164897	100.112534
99.7309724	99.7869809	99.9735688
99.6165738	99.7531754	100.171064
99.9953561	100.085925	99.8062361
99.9648001	99.9929973	100.032099
99.6769405	99.6422085	100.303746
99.8515194	99.9229608	100.146647
99.6711647	100.061215	100.316134
99.7332082	100.041811	99.9107293

99.6469435	100.090927	100.030304
99.4671477	99.7311943	100.490289
99.6473161	99.7424122	100.409855
99.3637418	99.5557999	100.030483
99.6096801	99.7078488	99.7225697
99.5448418	99.6299294	99.9572305
99.5211796	99.6146184	100.107507
99.6603583	99.5100185	100.103736
99.4494475	99.5253295	100.294589
99.4542918	99.6247752	100.252756
99.7801601	99.8047174	99.8288583
99.8351236	99.8639907	100.320623
99.5198753	99.7924383	100.381128
99.6113569	99.9082562	100.418293
99.8308383	99.9308437	99.9787755
99.9633096	100.23191	99.6121517
100.045662	100.327566	99.6936635
99.894745	100.317864	99.7814594
100.187449	100.40791	100.120075
100.274273	100.442625	99.6130494
100.702801	100.771282	99.5651118
100.432269	100.401998	99.8631507
100.512199	100.459604	100.059749
100.12317	100.218266	99.7588371
100.023304	100.206593	99.4692366
100.332962	100.389871	99.5491326
99.9118861	100.149291	99.9879321
99.8438805	100.076829	99.9294016
99.864189	99.7593908	99.6218469
100.755902	100.164905	99.7234674
100.400595	99.9162907	99.6866614
99.8159329	99.7128514	99.5218423
100.029079	99.8912777	99.8994181
99.0986128	99.2262343	100.362097
100.189685	100.063489	99.7514759
100.028707	99.934482	99.4466144
100.493009	100.37365	99.6943817
100.129318	99.9273571	99.8170086
100.909986	100.460362	99.697793
100.322901	99.9857208	100.045924
100.193411	99.8890038	99.8606372
100.462825	100.071371	99.7511168
100.169749	100.186886	99.7306491
100.499343	100.48492	99.993857
100.403576	100.357581	99.9881116
100.859308	100.536311	100.168192

100.556729	100.248585	99.9346083
100.885392	100.316044	99.6358512
100.174779	99.7148221	99.8724869
100.667774	100.127765	99.9319152
100.408234	99.7460505	99.8486079
100.213533	100.007247	99.7836139
99.5048246	99.3843824	100.187303
99.3362332	99.1100292	100.397708
99.3583527	99.0342569	100.277092
99.2918083	99.1706773	100.124396
99.325824	99.1930611	100.243578
99.1761922	99.1470835	100.155401
99.6243443	99.6219838	100.197339
100.003536	99.8701722	99.7412221
100.843147	100.416156	99.4548273
100.385701	99.7931899	99.6810039
99.9247233	99.7773095	99.8955312
99.5661644	99.4232517	99.698926
100.349827	100.051209	99.3164688
100.438119	100.040925	99.5155831
99.9533485	100.066031	99.7320819
99.8039025	100.116848	99.9021624
100.035135	100.159347	100.479074
100.210232	100.154507	100.668152
100.929394	100.457899	100.63643
100.822515	100.674175	100.392331
100.803927	100.556358	100.595747
100.624926	100.352635	100.379786
100.368228	100.423567	100.281931
100.501317	100.441565	100.016864
100.634778	100.500096	100.40882
100.339603	100.294558	100.637864
100.378823	100.236481	99.8383598
100.301684	100.478014	99.7684637
100.13681	100.421903	99.7879988
99.9821596	100.242682	100.215799
99.9563226	100.332368	100.07063
100.260233	100.497827	99.9478637
100.039038	100.468032	99.8842403
100.007067	100.207291	99.7173858
99.8434945	100.057259	99.8770715
100.117851	100.155566	100.106653
100.036436	100.061342	100.028692
99.9583672	100.246009	99.5084143
99.5780606	99.8128514	99.6365572
99.4847498	99.8489983	99.7172065

99.5555694	99.6301509	99.7935546
99.9026038	99.6213788	99.3512376
99.6784348	99.4524414	99.8915884
99.6254595	99.4848072	100.698261
99.6535271	99.5829633	100.611698
99.996844	99.9081339	100.421365
100.168967	100.040017	99.9614845
99.9656165	99.7948536	100.015609
99.9436828	100.021717	99.9665027
99.975468	99.9267367	100.145007
100.088482	99.9932833	99.9417702
100.211905	100.215155	99.7915832
99.6009236	99.8060455	99.9320923
99.5602163	99.8081629	99.7869234
99.4479459	99.6179003	99.8388975
99.9462851	99.8712309	100.001092
99.8304831	100.077071	99.6338689
99.571369	99.994342	99.8224092
99.5351228	99.6705325	99.8573572
99.5524094	99.8866576	99.9679365
99.9498168	100.084029	100.109879
99.6395863	99.7512958	100.088194
99.9089236	99.8899849	99.8614793
99.7327112	100.026254	99.8808351
99.800185	99.9497255	100.066866
100.12417	100.009466	100.409357
100.325105	100.158742	100.301108
100.631432	100.492382	100.018477
100.486261	100.260377	99.9019832
100.475852	100.164489	100.072781
100.459123	100.212433	99.9482222
100.197592	100.307262	99.7819052
99.7830842	100.099002	99.9561079
99.7553883	100.085692	99.7507208
100.111717	99.9321814	99.3372584
100.136996	99.8081629	99.3867234
99.9262103	99.7599166	100.138375
99.9429393	99.7950048	100.448786
99.8682163	99.954414	100.194472
100.117665	100.111555	99.948939
99.6078011	99.6830856	99.8197208
99.5433014	99.771411	99.9270741
99.6566871	99.7000248	100.015609
99.9894089	99.9132762	100.149308
100.1738	99.9656059	99.6286715
99.9961005	99.906924	99.259835

99.7868017	99.4026827	99.9942819
99.3843756	99.3063415	100.77676
99.9553932	99.7266434	100.293043
100.083463	100.110193	99.8917676
99.7565036	99.74328	100.279243
99.3577951	99.4373172	100.545924
99.6011095	99.5932478	100.451653
99.7353135	99.9067728	100.053425
99.9375489	99.9660597	100.065253
99.9726798	99.978764	100.369212
99.8330854	99.9159985	100.502911
99.6747172	99.8718358	100.200744
100.113204	100.077676	99.9215183
100.329194	100.333729	99.4044662
100.083463	100.174774	99.6793909
99.8684348	100.126321	99.8033834
99.6634579	99.9617785	99.7279497
100.148812	100.01299	99.6320899
99.7556138	99.9135974	99.9196695
99.8896586	99.9978385	100.22875
100.387672	100.261774	99.9159067
100.226074	100.32344	99.9064103
100.218627	100.337833	99.7709523
100.020538	100.205563	99.9379456
99.9784633	100.140412	100.411153
100.118466	100.056474	100.602335
100.426024	100.021929	100.212624
100.101524	99.6843586	100.474761
99.6682984	99.4031508	100.731164
100.025565	99.5955721	99.9845317
99.878302	99.7151156	100.078062
99.9025046	99.5782996	100.126619
99.5942014	99.4301201	100.371376
99.7608266	99.7052673	100.200799
99.8578229	99.826326	99.9128607
100.213786	100.215411	99.7713106
100.128705	100.195411	99.8402939
100.213973	100.232683	100.049215
100.045858	100.290561	100.154929
100.241526	100.394954	100.175893
100.192004	100.322379	99.6758091
100.766721	100.556163	99.5014696
100.449854	100.195866	99.8184343
100.014767	99.8763253	99.7266955
99.6405586	99.6769344	99.9445751
99.8146307	100.134352	99.751422

100.0224	100.316773	99.6677461
100.614245	100.833735	99.6666711
100.387859	100.676464	99.6052133
100.297192	100.393893	100.16568
99.9207496	99.9687481	100.608427
99.8146307	99.684207	100.485153
99.4238528	99.4048174	100.305976
99.1777315	99.268759	100.325327
99.1133155	98.9999753	100.467056
99.6018345	99.346182	100.184852
99.8006677	99.4769375	99.8404731
99.4084004	99.5101188	100.105476
99.7718108	99.7473879	99.9830982
100.006575	99.9122338	100.073404
99.9509096	99.9023855	100.033626
99.7649224	99.9642027	99.8402939
99.9073451	100.236471	100.07627
99.7989922	99.9232942	99.9922363
99.6273403	100.066474	100.170159
99.6241753	100.077686	100.197932
99.7747896	100.075565	99.6317315
100.225143	100.414196	99.793887
100.241526	100.240107	99.8539114
100.15961	100.253289	99.6568163
99.7392305	100.045717	100.089888
99.2428922	99.5919357	100.590689
99.7749758	99.6286018	100.140416
100.107668	99.9908689	99.8533739
99.4886411	99.4817859	100.265482
99.6962244	99.6663285	100.088275
100.474802	100.424044	99.6577122
100.227563	100.088898	100.033985
99.9231698	99.8935977	100.392877
100.024076	100.05405	99.9200278
100.524324	100.740554	99.6428405
99.9388084	100.07011	100.230005
99.8051359	99.9948083	100.029864
100.139317	100.509649	99.9882944
100.583713	100.687222	100.281608
100.029289	99.7817813	100.600185
99.9313614	99.5955721	100.153317
99.9533299	99.6663285	99.9544299
99.9970806	99.8358713	99.8601826
100.244877	100.311773	99.7892284
99.9764154	100.066777	99.8157466
99.8038327	100.13617	99.8797129

100.155887	100.328136	99.7981872	
100.409269	100.532224	99.4434161	
100.213042	100.394954	99.5353341	
100.059076	100.053595	99.7770443	
100.581665	100.254047	99.5292421	
100.574404	100.14526	99.4434161	
100.806004	100.305106	99.3900213	
100.521903	100.286016	99.6652377	
100.792786	100.487376	99.7689813	
100.427886	100.241017	99.5982253	
100.345225	100.324349	99.659504	
100.411503	100.481467	99.5828161	
100.123865	100.516467	99.4143894	
99.5001839	100.225865	99.9900862	
99.2172003	99.6113294	100.463831	
99.7548691	99.3522425	100.395564	
99.9626386	99.4976948	100.011229	
99.6569419	99.4916343	100.427996	
99.7679012	99.6354199	100.181627	
99.7295495	99.5998144	100.398073	
99.6945489	99.4781496	100.443763	
99.7788854	99.7286003	100.028251	
99.952399	99.9579907	99.9325702	
99.7938039	99.7272358	99.8107662	100.116468
99.6185952	99.7223884	99.9165284	100.133907
99.5861491	99.7662574	100.225662	100.069198
99.8000838	99.7185105	100.083891	100.006783
99.4632728	99.5999916	100.058073	100.079447
99.6363882	99.5023165	99.4801177	100.143392
99.6545998	99.6734297	99.7480335	100.11555
99.4283148	99.3888874	100.000776	100.025446
99.3456297	99.4683847	99.959105	100.082813
99.5116279	99.7458983	100.120126	100.056501
99.75445	99.9121641	99.3134346	100.00969
99.5997555	99.6019306	99.9799404	99.7659968
99.225056	99.2449196	100.13462	99.7136785
99.1771195	99.2534026	100.11537	99.9974515
99.132323	99.348654	99.6696744	100.076235
99.3921008	99.4177294	99.7620748	100.042121
99.3935661	99.578663	99.9058842	100.099181
99.2346851	99.5689683	100.273221	100.147675
99.151372	99.5728462	99.8372633	100.107136
99.3854023	99.2660058	99.8295633	100.029577
99.7693123	99.9371282	99.7459953	100.253383
99.3324419	99.3055121	99.728557	100.177965
99.57338	99.7458983	100.059658	100.134825

99.4339667	99.7621371	100.359507	99.9564535
99.5380034	99.6976667	100.214338	99.967162
99.487555	99.492137	100.223624	100.179647
99.3994273	99.6831245	99.9507256	100.075776
99.6282243	99.7410509	99.2597609	100.142015
99.8538814	100.156958	100.180594	100.035696
99.53905	99.8794442	100.315571	100.145839
99.8287619	100.091518	100.190332	100.220951
99.9461957	99.9705753	100.052411	100.293616
99.9947601	100.054435	99.47355	100.230589
99.998528	100.141931	99.8259398	99.9876609
99.611478	99.911437	100.207544	99.8225984
99.6621357	99.7652879	100.135753	99.9047472
99.6926978	99.763349	99.9980581	99.8908263
99.8494855	99.7747403	100.156362	99.9495696
100.051279	100.170288	99.4712853	100.054665
99.9139589	99.7938876	99.9692962	99.9015347
100.125382	100.094184	100.18603	99.9148437
100.186924	100.214884	100.393704	99.8190799
100.418024	100.434714	100.123523	99.8527349
100.152176	100.388421	100.285451	99.8486045
100.379717	100.299956	100.01912	99.6826241
100.035998	99.9136183	100.647352	99.7898612
100.021973	100.005961	99.8311486	99.9767995
100.074724	99.7587439	99.9532168	100.083578
100.218952	100.08352	100.134168	99.8603838
99.6012209	99.7403238	100.453039	99.5629959
100.015693	100.228942	100.298812	99.7328007
99.8486482	99.9218589	100.204374	99.8409557
100.149454	100.117694	99.4262175	99.9653262
100.25705	100.048618	100.080947	100.051452
100.07912	100.098304	99.9720139	99.8553355
100.151129	100.060495	100.09227	99.8039351
100.262492	100.173197	100.255556	99.8571713
100.106123	99.9223436	99.5752358	100.0796
100.075561	100.139022	100.255556	100.084343
100.091889	100.047891	100.337765	100.089544
100.138151	100.172954	100.125788	100.132072
100.198647	100.280082	100.389854	100.052676
100.02281	100.330737	100.139829	99.9884258
100.220626	100.123511	99.1927253	99.9879669
100.360249	100.270145	99.5011796	100.1541
100.083934	100.036985	99.9819786	100.210396
99.8149461	100.025593	100.095894	100.169245
99.9034925	100.072128	100.289527	100.209784
99.8896767	100.054435	99.8970518	100.149511

100.047092	100.192101	100.202109	100.025905
99.9045391	99.8905932	100.327348	100.101017
100.07912	100.13878	100.027046	99.9238694
100.215603	99.9708176	99.3736761	99.7918499
100.663149	100.431078	99.6205301	99.6419322
100.373856	100.266994	99.9013548	99.6457566
100.526457	100.274022	100.087061	99.8235162
100.19446	100.088125	100.159985	99.9648673
100.32571	100.192344	100.169724	100.117998
100.037254	99.9640313	100.240609	100.115703
100.201368	100.242757	99.7745307	99.9146907
100.256631	99.9334927	99.3926997	100.102241
100.391439	100.168592	100.181727	99.9795531
100.103402	99.874112	100.249668	100.047322
100.466379	100.443197	99.9991905	99.865432
100.342875	100.289292	100.037011	99.7179619
100.901784	100.628367	99.6864333	99.9916383
100.65352	100.288807	100.034973	100.056654
100.855522	100.62255	100.09295	99.9924032
100.726366	100.400782	100.157494	99.9720572
100.478102	100.418475	100.164741	100.09245
100.303312	100.197918	99.5396797	100.095816
100.29431	100.156958	100.326668	100.02254
100.19467	100.028744	100.42156	99.9200449
100.326547	100.161563	100.190785	100.094286
100.203671	100.095396	100.206412	100.209325
100.364017	100.369759	100.11605	100.074705
100.530225	100.180225	99.3598613	100.107595
100.637192	100.364669	99.5790858	100.203818
100.418233	100.295836	100.044938	100.127941
100.607886	100.489974	100.02682	99.8583951
100.511213	100.152676	100.249113	99.6855165
100.496137	100.142994	100.084147	99.7968898
100.407773	100.109592	100.666061	99.7076994
100.373013	100.048839	99.7727963	99.8508936
100.647319	100.256756	99.9286984	99.8296287
100.561258	100.185836	100.150315	99.7344718
100.776933	100.29161	100.307577	99.680315
100.623239	100.218997	100.242315	99.7274345
100.647738	100.179785	100.237783	99.7594084
100.449023	100.252641	100.395725	99.9156064
100.609837	100.158001	99.9008264	99.8663451
100.628892	100.132586	99.6368352	99.8567071
100.596017	100.277088	100.346779	99.8106585
100.442951	100.064572	100.371932	99.8600728
100.464518	100.352848	100.192236	99.7513002

100.407144	99.9723521	100.234611	99.8285578
100.365894	100.203748	100.135359	99.6552255
100.5778	100.051259	99.5078987	99.5775089
100.764998	100.556408	99.6708255	99.9451326
100.67789	100.546	100.359015	100.030039
100.687732	100.439258	100.090945	100.030651
100.387671	100.067234	100.184985	100.004644
100.572565	100.367129	100.101142	100.108674
100.462843	100.102573	99.5631896	100.17767
100.300772	100.079578	99.6381948	100.289808
100.114831	99.6666485	100.202433	100.160536
100.09787	100.026812	100.007103	99.9963826
100.219318	99.9781612	100.26135	99.8176958
100.158175	100.027781	99.6189337	99.9354945
99.9634394	99.9328987	99.8262743	100.248808
99.5999322	99.7102167	100.227586	100.244678
99.806813	99.8377748	100.110886	100.369055
99.9347525	99.5826587	100.268148	100.570689
99.8637681	99.8767441	99.3601543	100.441417
99.8543454	99.9793715	100.125162	100.253092
99.8556017	100.013742	100.007329	100.069204
99.7442044	99.8222838	100.060807	100.110663
99.7739383	100.006481	99.8181167	100.046409
99.7454607	100.016646	99.5348644	99.9229497
99.8262867	99.9486317	99.7150128	100.140954
99.8840793	99.9389498	99.9953193	100.181189
100.053269	100.057068	99.9697133	100.073181
99.9728621	99.7206247	100.41272	99.7173375
100.344326	100.303713	99.5400762	99.8082107
99.9330774	99.8130861	100.359922	99.7777667
99.6173119	99.8060668	100.414986	99.9842968
99.6771984	99.7138474	99.9280186	100.153346
99.5195251	99.5957291	99.7134266	100.222036
99.703582	100.016646	99.3819081	100.28476
99.7779167	100.036736	100.069418	99.8545653
100.081328	100.07861	99.974472	99.9983714
99.8591614	99.8489089	100.039733	100.049163
100.421383	100.339777	99.4301743	100.066603
100.030445	100.042303	100.142837	99.9862856
99.9854258	100.088292	100.079389	100.146614
99.8939208	99.7854929	100.472996	99.9237146
100.083841	100.253851	100.067832	99.9358005
100.193772	100.21706	99.6214263	100.000819
100.259941	100.156307	99.804294	100.234122
99.9066938	99.9188601	100.037241	99.9024497
99.9925451	99.8322077	100.264296	99.7058636

99.8218894	99.7668554	100.286729	99.7203972
99.9883573	99.84431	99.8006683	99.9174422
99.5385799	99.6523678	100.08732	99.9585953
99.5331357	99.8641577	100.40139	99.9273863
99.6143804	99.6930315	100.56409	99.8758302
99.7083981	99.6141246	100.358109	100.200924
99.3633175	99.717236	99.3565287	100.226625
99.6346915	99.934593	99.4267753	100.023614
99.4112686	99.5611166	99.4387852	100.270685
99.6996035	100.022698	99.6425002	100.364006
99.6983472	99.8031622	100.029989	100.251715
99.9184198	100.220691	99.6760373	100.33754
99.9247016	100.265469	99.342706	100.234122
99.9984082	100.007691	100.037694	100.247126
99.8736096	100.218997	100.000984	100.14279
99.8662808	99.9832442	100.299192	99.8793489
99.8455509	99.9592817	99.9180481	99.8443153
99.5915565	99.8643998	100.17796	99.8493638
99.9684649	100.126535	100.019792	99.7926062
99.5787835	99.4536483	100.099783	99.9766475
99.8960147	99.9036112	99.443997	100.232592
99.9508758	100.215366	99.8654765	100.262577
100.0428	100.068686	100.359922	100.110816
99.8635587	100.410455	100.169803	100.049469
99.7395977	100.093375	99.9769646	99.9833789
99.855183	100.159453	100.02863	99.9992894
99.6434861	100.017615	99.7036827	99.9183601
100.142052	100.29161	99.7231705	99.9561475
99.6962532	99.6472848	100.535312	99.8479869
100.08049	99.9798556	100.342927	99.9985244
99.6043295	99.8280929	100.410454	100.051151
99.8585333	99.8140543	99.6640274	99.8928116
99.6954157	99.8961078	100.272453	99.6614979
99.45566	99.7627406	100.336129	99.6980613
100.128651	100.167683	100.004383	99.8753713
99.6231749	99.6184814	100.049251	99.784651
100.343489	100.360109	99.7279291	99.78511
99.6078892	99.8871521	100.437873	99.6408448
99.3444754	99.9101683	100.350735	99.7426419
99.3208731	99.813265	100.27142	99.9349104
99.3488617	99.725303	99.5863696	100.103758
99.7344359	100.07691	100.080386	99.8493387
99.485045	99.8374304	100.244906	99.7372841
99.8077493	100.160764	100.054325	99.8321937
99.224584	99.6054426	100.163779	99.978232
99.2588387	99.8688455	99.4189027	100.256685

98.8456936	99.6114839	100.05999	100.43885
99.0802547	99.5749941	100.331699	100.313477
99.4938176	99.934092	99.9373928	100.276126
99.3624383	99.7429437	100.044581	100.465792
99.779343	100.317355	100.050246	100.269084
99.5884357	100.006347	99.9208501	100.148916
99.377268	100.073043	99.706701	100.316845
99.192418	99.7980408	99.6749752	100.117994
99.716682	100.187104	99.4173164	100.190401
99.8551629	100.373661	100.19981	100.075438
99.9247166	100.249692	100.152675	100.170501
99.8681128	99.9526994	100.008776	100.612596
100.003878	100.259117	100.209555	100.41053
99.6594516	99.6146254	100.431409	100.185809
99.6385646	100.038487	100.358666	100.286841
99.684516	100.071593	99.3187398	100.369351
99.7304674	100.01408	100.199357	100.200198
99.2939289	99.6822885	100.034836	100.180604
99.7611713	100.071593	100.121402	100.037474
99.8234146	100.175021	99.5945276	100.228671
100.049621	100.21876	98.9672633	100.162693
100.095781	99.9502829	99.9281017	100.180298
99.9126021	100.118232	99.4979906	100.015584
100.180583	100.370519	100.27006	99.9433298
100.135467	99.9855644	100.457469	100.292811
99.8608024	100.052986	100.004244	100.025534
99.990093	99.9688902	100.026452	99.8873025
100.208153	100.480713	100.065202	99.7019226
100.062571	100.130073	100.18984	99.777238
100.008474	100.176713	100.11619	99.6948809
100.014113	99.9580158	100.121856	99.7372841
100.183507	100.308172	100.0473	99.8580642
100.032076	100.015288	100.17375	100.067018
100.260789	100.279657	99.9419251	99.9440952
100.109149	99.9502829	100.246266	99.6953402
99.9669084	100.246792	99.7853356	99.7533575
99.9959413	99.934092	99.639397	99.710342
100.201887	100.112191	99.5365147	99.6685512
100.276036	100.127899	99.6115236	99.7937706
100.275827	100.173571	99.4481357	99.9359819
99.9230457	99.7320693	100.006963	99.9053659
99.843675	99.9406167	100.268248	99.909346
99.8190283	99.947383	100.105993	99.9933869
100.043146	99.9128265	100.308131	99.9613932
100.091813	99.9548743	100.007869	100.138201
100.13672	99.9879809	100.093982	100.315161

99.8152687	99.7272362	100.373396	100.135598
99.9961502	100.069902	100.330793	100.338735
99.9756809	99.8504797	100.079026	100.102074
100.137555	100.022054	100.099194	99.9292464
100.345172	100.055402	99.1863979	100.031657
100.21254	100.215377	100.046847	100.04819
100.599367	100.491588	100.038915	100.099624
100.226743	100.12959	100.237202	100.104982
100.394048	100.339346	100.036196	100.110646
99.9163618	99.8717453	99.9831686	100.053088
100.071761	99.9408584	99.4155035	100.232957
99.834067	99.596743	100.300427	100.170041
99.8685306	99.9297423	100.364331	100.165296
99.8793918	99.9495579	100.344616	99.9665979
99.8827337	99.8352555	100.032117	99.9148569
99.9364133	99.6126922	99.7853356	100.014359
100.378591	100.218519	98.9890181	99.983896
100.59707	100.467664	99.3561309	99.9160815
100.758526	100.532911	99.9473638	99.6783483
100.300474	99.8758534	100.244453	99.6755929
100.261624	100.021087	100.065429	99.7787688
99.9547939	99.6537734	100.330339	99.760093
100.160531	99.8741618	100.217486	99.7911683
100.313215	99.8403303	99.9627735	100.00594
100.166588	99.8357389	100.209555	100.118913
99.983618	99.9790397	100.591624	99.8790362
99.888791	99.7646926	100.567603	99.7665224
100.110193	99.9667153	100.369544	99.8003531
100.105389	99.838397	99.7051147	99.8536249
100.174943	100.009246	100.150409	99.9554231
99.6696862	99.6844634	100.065656	99.939962
100.176405	100.112674	99.8059574	99.7795342
99.8831515	99.7257863	100.027811	99.6769706
100.130454	99.807707	99.9915533	99.49603
100.207736	100.094792	99.5095478	99.8692391
100.124396	99.8939775	100.057044	99.8847001
100.237186	100.38671	100.276406	99.9721088
100.09411	99.9514911	100.324674	99.8456647
100.281467	100.182754	100.063616	99.7732579
100.333893	100.021087	99.8564921	99.8089255
100.471747	100.028095	99.8508267	100.025228
100.626729	99.9998219	100.389485	100.111871
100.397807	100.033895	100.119363	99.9868045
100.344546	99.8359805	100.014441	99.8594419
100.184969	99.5933599	100.01784	99.7404988
100.301101	99.8717453	99.9251557	99.5108788

100.447519	100.002963	100.121629	99.7385087
100.853211	100.356465	100.116343	99.8530256
100.861179	100.352583	100.047037	99.8886576
100.695953	100.068708	100.273979	99.7478117
100.818824	100.371993	100.273526	100.133647
100.728453	100.082052	100.323806	99.9611451
100.854679	100.306484	99.5424207	99.938359
101.063518	100.493793	99.893931	99.9871427
100.77605	100.239518	100.150543	99.652691
100.791147	100.393102	100.239779	99.6224114
100.723421	100.105345	100.221434	99.5880028
100.806873	100.175222	100.228228	99.6092597
100.598453	100.250436	99.4450306	100.104132
100.300711	100.047356	100.180892	99.9995298
100.198179	100.194389	100.096865	99.6896994
100.53576	100.513203	100.118381	99.6091068
100.43763	100.263781	100.047717	99.6786886
100.064824	100.06434	99.6812583	99.7915489
100.130872	100.166487	100.014876	100.040056
99.7805011	99.798177	100.249065	100.020328
100.066082	100.248738	100.050435	99.8923279
100.058114	100.001257	99.9641425	99.8193816
99.9503399	100.058517	100.071498	99.9423351
99.9249689	99.8828543	99.616256	100.048925
100.103404	99.9083303	100.211921	100.290856
100.038614	99.9522461	100.332866	100.078287
99.9461463	100.260627	100.221207	100.134106
99.8192914	99.8105511	100.018047	99.9258189
99.8014688	99.9794205	100.085087	99.8933984
99.7666624	100.021153	100.160961	99.7415417
99.7215817	99.9389015	99.9179388	99.6945931
99.8956141	99.8971694	99.3634947	99.9247484
99.733114	99.6511442	99.7383334	99.9758261
99.7723237	99.7086471	99.9761464	99.8874342
99.4498396	99.5536075	100.261975	100.039903
99.7547108	99.7275721	100.284397	100.030574
99.7236785	99.7445561	100.296628	100.122636
99.8178237	100.022366	100.463323	100.16148
99.6146462	99.5400203	99.9066144	99.8450734
99.7689688	99.8692671	99.2611219	99.8219814
100.045114	100.333173	99.418305	100.170502
100.079921	100.083265	99.9752405	99.992954
99.8065011	99.7588712	100.199691	99.9545692
99.8402592	100.19633	100.137633	100.158574
99.5714526	99.5385646	100.020991	100.108567
99.8794689	99.7431004	100.134688	99.9360651

99.9834689	99.9621938	99.9118236	100.052137
100.202792	100.088118	99.4053951	100.007788
100.292953	100.065068	100.058588	99.8929396
99.9044205	99.7918687	100.491181	99.9417234
100.331534	100.246797	100.196973	100.044949
100.240743	99.9881551	100.265146	99.9564044
100.374308	100.322982	99.5329082	100.064065
99.9683722	99.7608122	100.317238	99.908997
100.074469	100.093213	100.08237	100.096027
99.7775656	99.6237272	100.27715	100.123095
99.9679528	100.076229	100.081464	100.044032
99.7846946	99.8304466	99.3347307	100.07844
100.000033	99.9631644	99.8239461	99.9680268
100.118501	99.8248661	100.197879	100.027363
100.13234	99.8277777	100.184969	100.136553
100.229211	100.080354	100.002646	99.8718357
99.9352431	100.0015	100.34872	99.7941487
99.9276947	99.9862141	99.6751431	100.195888
99.8811463	99.9607381	99.5632577	100.352027
100.003388	100.056819	99.6398108	100.269752
99.5964042	99.8260793	99.7668709	100.006564
99.5309848	99.7933244	99.9093323	100.030268
99.9834689	100.227387	99.8119422	100.029045
100.167146	100.096853	99.4033567	100.137164
100.10655	100.049055	100.074669	100.326029
99.8140495	99.868054	100.51451	100.12126
100.22963	100.280765	100.240459	100.081346
100.130663	99.8420928	100.236155	100.125389
100.198808	100.169156	99.5238487	100.195735
100.02855	100.121601	99.6411698	100.298808
100.199227	100.436047	99.8604107	100.2592
100.173646	100.338996	100.025974	100.016658
99.9843076	100.018241	100.163679	99.9735322
99.8471785	100.061672	100.00944	100.05076
99.6855172	99.8585915	100.277376	99.908844
99.4689203	99.869995	100.193349	99.9775083
99.5668397	99.510177	100.313841	99.8592956
99.7100494	99.8947431	100.043867	99.8467556
99.565372	99.8229251	100.073763	100.147716
99.7557591	99.8658703	100.164358	100.239931
99.8564044	100.12063	100.105698	100.110249
99.9258076	99.9777221	100.125629	99.86893
99.8094366	99.9471509	100.127441	99.8175465
100.157921	100.066767	100.057229	100.126306
100.006953	99.8743623	100.193802	100.038373
99.7716946	100.07259	100.10298	100.109332

99.5873881	99.8357844	100.318144	100.199406
99.753872	100.09103	100.054285	100.125848
99.8876463	100.090059	100.245668	99.7733506
99.9966786	100.019939	100.053379	99.8906457
100.064614	100.015815	99.5177335	100.033327
99.9721463	99.9636496	99.0969177	100.215922
100.031066	99.9937355	99.7469399	100.109637
100.206566	100.41227	99.7564525	100.159492
99.939856	99.8804281	100.20105	99.9785788
99.8688373	99.6751728	99.8510773	100.029543
99.920585	99.7473019	99.8348726	100.048166
100.037062	99.837542	99.7923801	100.042498
100.230004	99.8548656	99.7812169	100.017802
100.324253	99.9914069	99.904913	100.054374
99.993672	99.726986	99.9207577	100.054509
100.035817	99.9055764	99.7585302	100.073672
100.022302	99.8112416	99.6951517	100.142362
100.328165	99.8608501	99.8427949	100.007951
99.9182733	99.7824215	99.8379335	99.9906769
99.9189846	99.7345454	99.8181277	100.048301
99.8924883	99.8444714	99.9198574	99.9246858
100.170077	99.9630592	99.8914091	99.8847403
100.037417	99.7241513	99.7905796	99.8419608
99.901024	99.5027244	99.5788377	99.8690859
100.166876	99.8005325	99.4722465	99.9492469
99.7504044	99.3488595	99.740525	99.9335926
99.8787956	99.6482425	99.655	100.061796
99.9244972	99.7531289	99.5048361	100.179473
99.9550835	99.7502941	99.6897501	100.444652
99.7246194	99.6202098	99.9915184	100.210917
99.4692595	99.2274369	99.8678222	100.178799
99.6378397	99.5694989	99.7362037	100.081364
100.116728	99.825573	99.7822972	99.943579
100.22787	100.146532	100.008623	99.8078181
100.004697	100.023849	99.9580286	99.890948
100.079918	100.074088	100.126558	99.7680076
100.001141	99.7852563	100.055797	100.165574
100.14358	99.7347029	100.013125	99.7441212
100.160118	99.7356478	100.171211	99.9064674
100.224136	99.67848	99.8231691	100.078935
99.8891096	99.7756495	99.4884513	100.038989
100.130421	100.074088	99.5658739	100.254236
99.8446528	99.7688776	99.8528778	99.9620673
99.728176	99.8151788	99.9445246	100.019826
99.9577509	100.107947	99.805524	100.089056
100.377601	100.392212	100.143123	99.9746177

100.128643	100.37536	100.096669	100.06598
100.053778	100.452214	100.083885	100.20201
99.944236	100.461978	100.54464	99.9580187
99.9223633	100.412055	100.404919	100.002283
99.6191679	99.9880997	100.3383	100.060717
99.6364171	100.100388	100.258536	100.103901
99.8942666	100.272049	100.27168	100.035211
99.7560948	99.9838475	100.092888	100.015103
100.069071	100.2952	100.20254	100.069083
99.8115769	100.205274	100.473339	99.9971545
99.7520048	100.147477	99.9585687	99.9698944
100.00772	100.267482	100.251334	99.9333227
99.7715658	100.103695	100.432467	99.9752925
99.7424021	99.7918707	99.9958396	100.05154
100.031371	100.079285	99.9540674	100.02455
100.030838	100.051095	99.9994407	100.016453
99.9623744	100.150784	100.256916	99.8271161
99.9817576	100.025897	100.247553	99.990272
100.389338	100.035661	100.082805	99.9299489
100.187859	100.079127	99.956048	99.9408799
99.9237859	99.7666728	100.122777	100.015238
99.3511822	99.3162596	100.025368	100.0371
99.755917	99.8644723	100.017986	99.9009344
99.8576342	99.8808509	100.140422	100.09378
99.5862698	99.610918	100.16707	100.025224
99.9630857	100.014085	100.008983	100.07934
99.8688373	99.8340773	100.246473	99.9734031
99.7313768	99.8269903	100.147804	99.9663857
99.8441193	99.8079344	99.9913383	100.177584
99.4434746	99.1949945	99.9960197	100.126573
99.8211796	99.8271478	100.199119	100.019961
100.129176	100.037078	99.9980003	100.058287
99.785792	99.5970592	99.8028232	100.030218
99.8457197	99.6685583	99.79202	100.185951
100.334389	100.118184	99.7707738	100.013619
99.649932	99.4876056	100.140242	99.9786662
99.9511713	99.9657365	100.063179	99.9561294
100.35395	100.516154	100.109633	99.9287343
99.8035747	99.9844775	99.9875572	100.019691
100.056801	100.426229	100.083525	99.984739
99.6984788	100.037866	99.9672113	99.9642265
100.091477	100.121019	99.9238186	99.9898672
99.9029801	100.036921	100.16653	99.8790723
99.5804015	99.9076238	100.062999	99.9763721
100.089343	100.251733	100.081905	100.163279
99.6129439	99.9882572	100.310392	99.8222579

99.7957503	100.093774	99.9281398	99.8437151
100.143758	100.109995	100.001961	99.8978305
100.605042	100.430481	100.143123	99.7852813
100.491233	100.416937	100.017446	99.9063324
100.645053	100.72262	99.8136264	99.9954002
100.093789	100.278033	100.250254	99.9333227
99.8960449	100.054244	100.109993	100.024145
100.422413	100.419457	100.086586	100.155317
100.073694	99.8309275	99.9654107	99.932513
100.239429	100.436465	99.8939298	99.957209
100.70178	100.669231	99.938943	100.028463
100.676351	100.7527	99.9317409	99.7989114
100.092722	100.151414	100.077944	99.9329178
100.378134	100.358194	100.025548	100.034131
100.429704	100.095506	100.120976	99.9318382
100.401785	100.365596	99.9724328	99.9285994
100.569654	100.661357	99.9652307	99.9111907
99.7715346	100.395801	100.168747	99.9692511
99.9046853	100.505399	99.9920921	99.8615793
99.9119449	100.586139	100.186755	99.8538884
99.7486935	99.9777497	100.122107	99.9974509
99.8542226	100.28431	99.8249809	99.986117
99.8864479	100.359531	99.8255212	99.9240505
99.8251844	100.361581	100.143357	99.7845359
99.792605	100.261286	99.973004	100.028889
99.7669309	100.215397	100.05656	100.023357
99.818456	100.228013	100.086452	100.006896
99.5925247	100.230063	99.9016936	99.9969112
99.9149549	100.11321	99.9233028	100.298878
100.016412	100.305126	99.937709	100.070716
99.8533373	100.396747	100.201341	100.055739
100.006496	100.312853	100.19846	100.062081
99.8664399	99.8246272	100.468935	99.8189423
99.9330153	99.5098668	99.8701802	99.8352685
100.157353	99.5335212	99.5516245	99.9793707
100.265538	99.7902496	99.7308008	99.799243
100.086174	99.8120116	99.8537932	99.78629
99.6396232	99.4638197	100.020184	99.9718147
100.025265	99.7743223	99.9913718	100.015801
99.6633496	99.714398	99.9405902	99.9738386
99.8595345	99.9919423	100.001096	100.035635
100.028983	99.8422891	100.03477	100.109036
100.333884	100.108006	100.231954	99.9127167
99.9432849	99.9006365	100.108242	99.9827438
99.858295	99.6719779	100.12769	100.000689
99.8717518	99.8370852	100.090054	100.012698

100.030577	99.788988	100.302364	100.126171
99.9880816	99.9367488	100.183874	100.110385
99.9586893	99.8492277	100.050437	99.9642588
100.133981	100.088137	100.211245	100.159229
99.8627216	99.9157753	99.9414906	100.333824
99.7602026	99.8120116	100.229433	100.076788
99.7263837	99.8460738	99.8761227	99.9461786
100.042617	99.7793686	99.8575748	99.9507661
100.019953	99.5415636	99.9726438	99.9156851
100.116098	99.8022345	99.7624943	99.8452531
99.8136754	99.7664376	99.8118353	99.8775007
99.9590434	99.9127791	99.9202415	99.8828978
99.8308504	100.007081	99.8966515	99.9727592
100.131502	100.146326	100.002537	100.223049
99.6916795	99.6907437	100.205303	99.9225663
99.7607338	99.8730398	100.119046	100.025246
99.9916229	99.8831323	99.7378238	100.181491
100.018005	99.8586895	99.9319465	99.9568378
99.7874702	99.7243328	99.9825481	99.8730481
99.7972086	99.9400604	100.076548	99.980585
99.8150919	99.9756996	100.009379	99.976942
100.183735	100.100595	99.9654408	99.8953111
99.9386813	99.9009519	100.149839	99.8386417
99.7368304	99.9201908	99.7844637	100.018095
99.7471	99.9012673	100.012621	100.049938
99.9119449	100.148061	100.170548	100.025111
99.898134	100.143488	99.9409503	99.943615
100.255623	100.284941	99.9992952	100.056819
99.9092889	99.8738283	100.249422	99.9792357
99.8453695	99.7492487	100.05764	100.016476
99.858295	99.7167634	99.6931648	100.088392
100.355486	100.105798	99.8660384	100.025786
99.8820214	99.712821	100.035491	100.161117
99.8558162	99.8996903	99.8150767	100.047644
99.9108825	99.8561664	100.059801	100.062081
99.9583352	100.176288	99.9805672	100.094868
99.6421021	99.7855187	100.224571	100.124822
99.7035427	99.432596	99.871981	100.15599
100.042086	99.7940343	99.6006054	100.198357
99.9682507	99.8128001	99.5952031	100.376596
100.28112	100.075836	100.003077	100.171507
99.8377558	99.8567971	100.100858	99.9902998
100.048637	99.8416584	100.115085	99.9619651
99.9670112	99.9960423	99.9667013	99.852809
99.8983111	100.127876	100.132552	99.8675161
100.052178	100.321053	100.177211	99.9147406

100.347341	100.449733	100.166586	100.014991
100.063333	100.125668	100.006318	99.969521
100.426842	100.325784	100.048636	100.032127
100.283067	100.110845	99.8478507	100.001769
100.711558	100.417563	99.9596783	100.075978
100.416041	100.212085	100.077629	100.064914
100.12318	100.101225	99.8465901	100.029698
100.303606	100.348019	100.189456	100.127656
100.136814	99.8702013	99.9047549	100.022952
100.169924	99.9990386	99.7610537	99.913931
99.9337235	99.9563031	99.8797243	99.8497057
100.158947	100.170769	100.051157	99.9015177
100.199848	100.291091	99.9760653	99.8938269
100.249071	100.480326	100.19828	99.9580522
100.471638	100.391701	100.084291	99.9665526
100.702705	100.567216	99.9456323	99.8275777
100.231188	100.336192	100.208004	99.8526741
100.022963	100.212874	100.024146	99.9542742
99.9354941	100.077886	99.9409503	99.9904347
100.158947	100.388389	99.8165173	99.9167645
100.129554	100.279421	99.9521151	100.025246
100.131856	100.202624	99.9983948	99.9728942
99.8071241	99.9004788	100.180632	99.917574
99.84112	99.9127791	99.8426285	99.938083
99.5928463	99.3363264	99.6135695	99.9076231
99.8041534	99.5128237	99.6536866	99.9675373
99.8639473	99.7265937	99.8245889	100.099645
100.114941	99.8437365	100.094974	99.8629574
99.8411938	99.6493859	100.324163	99.7860406
99.6701021	99.2884044	99.7875301	99.8860325
99.6653398	99.2948253	99.8116363	100.072927
99.6831545	99.3967771	99.7376985	100.103559
99.852306	99.732858	99.8091177	100.244168
99.7919829	99.5837671	99.952136	100.125959
99.9299146	99.4486144	99.9208339	100.180205
99.7106702	99.3557458	99.988835	100.18142
99.7863386	99.4548787	99.7353599	100.05282
100.254636	99.6875983	100.089038	99.8656563
99.8355496	99.6725639	100.118901	99.7590523
99.67557	99.5123539	100.133652	100.034198
99.946671	99.5981751	100.021756	100.143231
100.062378	99.8442063	99.8857539	99.8872469
100.074549	99.9572773	99.6594433	99.8834686
99.9267397	100.15617	99.6711366	99.7473124
99.7690531	100.084756	99.7986837	99.859314
100.245111	100.583083	99.8638065	99.9189583

99.9634274	100.517151	100.292681	99.8837385
99.7314835	99.9613491	100.06835	100.046208
100.159389	100.445425	100.233495	100.048232
100.530499	100.853075	100.409075	99.9216571
100.678838	101.018767	100.496325	99.9016857
100.041565	100.561784	100.48787	99.9145052
100.350589	100.68065	100.157758	100.128253
100.649735	100.909298	100.209209	100.167116
100.319898	100.702262	100.508918	99.8468994
100.186905	100.746112	100.447753	99.8751022
99.8893464	100.302128	100.266596	100.081698
100.286738	100.631162	100.009163	100.104638
100.452538	100.322017	100.036508	100.160639
100.064848	99.9663606	100.10343	99.8458198
100.27492	100.539389	100.069069	99.7617511
100.239644	100.453881	100.169092	99.6919863
100.062378	100.379649	100.430303	99.7537896
99.9960583	100.297117	100.1556	100.200852
100.456595	100.553328	99.9972902	100.267108
100.076489	100.043725	100.309951	100.195454
100.081957	99.98578	100.134192	100.407043
99.7965689	99.7021628	99.9141777	100.553319
99.8359023	100.017885	99.8796374	100.373442
100.426786	100.277697	99.7416563	99.9873737
99.8071519	99.7265937	99.8012022	99.7904937
100.209658	100.422716	99.672216	99.9563371
100.50757	100.4946	99.9879355	100.034064
99.9235648	99.9254859	100.072307	99.8460897
99.8468381	99.955398	100.106308	100.035008
100.085661	100.252014	100.177727	100.1574
99.9128054	99.8983927	100.426885	100.038517
100.02816	99.7265937	99.9638293	100.198692
100.322367	99.974974	99.8076785	99.954178
99.9625455	99.8158603	99.9456597	99.9320476
99.7454177	99.7762384	99.6923644	100.021514
100.485875	100.344569	99.8713621	100.017331
100.056029	99.8752147	100.131314	99.9185534
100.03839	100.023366	99.985417	99.9827857
100.502984	100.17277	100.038846	99.9389296
100.180555	99.9610359	100.052699	99.6465109
100.165563	100.078805	100.018878	99.7821273
100.189551	100.071914	99.722947	99.7556788
99.9902377	99.993767	99.8481554	99.9041147
100.164504	99.920631	99.8616477	99.9918268
100.03839	100.021956	100.088498	99.9524238
99.9921779	100.174493	99.8862936	99.9197679

100.320427	100.355062	99.9271303	99.7566233
99.9994096	99.9461582	100.153621	99.8118145
100.220771	100.222571	100.094794	99.7513606
100.261868	99.9120176	100.118181	99.9061388
100.047562	99.8841414	100.029492	99.7695778
100.014755	99.8410742	100.023915	99.9624095
99.8351968	99.4976327	99.7769161	100.21664
99.8614779	99.8329305	99.6648402	100.075895
99.616658	99.7904898	99.6927242	100.060107
99.7623505	99.5109444	99.7062165	99.9925015
99.702733	99.8384118	100.03255	99.9270548
99.7080245	99.6381101	99.8197317	99.8510826
100.103652	99.6968381	99.8339435	100.056194
100.009111	99.8435799	100.24285	99.8742925
99.6600483	99.596609	99.7020789	99.8122193
100.065906	100.00238	99.8152342	99.9062737
99.5970795	99.5955127	100.000708	99.8158628
99.6962068	99.7958144	99.8893519	100.114084
99.689857	99.7276899	100.047122	100.110171
99.3854196	99.4525296	99.9789407	100.103963
99.7831637	100.074733	99.9855969	100.028801
99.6501708	99.6639505	100.030031	99.9065436
99.5702692	99.6335685	100.087239	99.9031701
99.8565392	100.117018	99.908421	99.9788724
99.960958	100.096972	100.024994	100.028396
99.9078667	99.8830451	100.057016	100.153622
100.044564	100.345665	100.053598	100.247676
100.172618	100.048423	100.045323	100.37884
99.6939138	99.6656731	100.128795	100.324053
99.8124434	99.8287021	100.04982	100.35401
99.5900241	99.3842485	99.6596232	100.238905
99.8004493	99.7455432	99.6607025	99.8379932
99.6794504	99.6927663	99.8724415	99.7926528
100.769459	100.753436	99.9416987	99.971504
100.621968	100.767021	100.181658	99.8364325
100.500662	100.579041	99.9989862	99.7122909
100.833051	100.804459	100.00367	99.7529068
100.539495	100.68788	100.23282	99.7048694
100.569598	100.862275	99.9914199	99.7662655
100.702305	101.009658	100.144006	99.8294158
100.357625	100.44003	100.259302	99.7852916
100.596318	100.355044	100.036818	99.9534225
100.421751	100.385373	100.049788	99.7507478
100.369025	100.006253	100.080954	99.9070043
100.769816	100.148739	100.101131	100.099289
100.346937	99.7054843	100.502864	100.002944

99.8937763	99.5342483	99.8986429	100.07473
99.9701937	99.4641111	99.9806109	100.002
100.063712	99.6991657	99.8373921	99.9281894
100.280851	99.773884	99.94332	99.9383096
100.282276	99.8080048	100.020784	99.7430564
100.052133	99.7367618	100.283082	99.8062067
100.027908	99.9654977	99.9721439	99.8604512
99.9750032	99.7988427	100.153374	100.104282
100.028442	99.8700858	99.9490848	99.9890457
100.205859	99.897414	99.9966442	99.9594946
100.037705	100.12378	100.070506	99.7288881
99.8419407	99.8952025	99.9528679	99.8435842
100.017754	100.294858	100.185261	99.7852916
99.7940239	100.099296	100.262185	99.767345
99.9787439	100.139893	100.059156	99.8605862
99.7844049	99.7143305	99.9051283	100.047878
100.007245	99.8980459	99.8390135	99.9080838
99.9201394	99.7762535	99.6298599	100.042616
100.082593	100.054907	99.6448123	100.138151
99.5512338	99.8108482	99.8981025	100.136666
99.8193183	100.031844	99.6235547	100.044235
99.7236629	99.7590351	99.6401284	99.9358807
99.6281857	99.5331426	99.9364743	100.080803
99.7323912	99.7719884	99.6876879	99.937365
99.5938067	99.6530394	99.8368517	100.017787
99.6698679	99.7552439	99.7930753	100.033305
99.5686905	99.6659926	99.8354105	99.9743377
99.3442477	99.6840009	99.7040815	99.9953878
99.482476	99.8264869	99.8883744	99.9836483
99.6607833	99.7699348	99.8038843	99.9828387
99.8895012	99.6857385	100.12455	100.08566
100.078318	99.8301202	99.9501657	99.9975467
100.020604	99.9160541	99.8487415	100.060022
100.198199	100.102929	99.5662672	99.9669161
99.9461462	99.8898316	99.4497105	99.9352061
99.8291154	99.6293443	99.5729327	100.055839
99.657577	99.5898526	99.8409951	100.127086
99.907136	99.9852435	99.8934186	99.9361506
99.7820893	99.8029499	100.086719	99.9319676
99.8581504	99.7707246	99.9557503	100.024129
100.108244	99.9460678	100.069244	100.046934
99.6996155	99.5078679	100.029431	100.032495
99.8371312	99.8143235	99.8851317	100.118585
99.7738953	99.7277577	100.074469	100.113862
100.105572	99.7860474	100.16022	99.9306182
99.6333514	99.7557178	100.023847	99.9728534

99.6641677	99.7242824	100.213544	99.967186
99.6826932	99.9263219	100.427922	99.9663764
99.9249489	100.251892	100.122208	99.9789255
99.9641373	100.029158	100.050149	100.088089
100.161326	100.047009	100.3537	100.012255
100.056586	100.034529	100.456566	100.195903
99.8401594	99.9478054	99.9676402	100.224375
99.8063148	99.8395982	100.164183	100.254736
99.7826237	100.004042	100.158779	100.043695
99.9028609	100.102139	100.276777	100.017787
99.9203175	99.9816103	99.9379155	100.240163
99.8884324	100.038478	100.058976	100.175258
100.032895	100.432921	100.159499	100.223161
100.148679	100.458512	100.182018	100.282398
100.169164	100.414913	100.032134	100.143278
100.316833	100.345092	100.22111	99.9639475
100.936366	100.753594	99.9334118	99.9871566
100.820582	100.667186	100.189945	99.9037659
100.786025	100.717736	100.241467	99.8815013
100.048392	99.9332725	100.411348	99.8574826
99.9990506	99.7020091	99.713089	99.9219823
100.325918	100.044797	99.9254852	100.032765
100.462365	100.373052	99.8876538	99.963003
100.335893	100.40275	100.080954	99.9327772
100.104503	100.04764	100.177694	100.069468
100.360475	100.245099	100.129234	100.177957
99.9796345	100.041164	99.8617123	100.248529
99.9929942	100.298018	99.8831501	100.382116
99.8761415	100.197235	100.036457	100.269039
99.9636029	100.074179	100.134098	99.9845928
99.9012577	100.041796	100.030692	99.8873036
99.696231	99.9446461	99.9681806	99.9238714
99.7922426	100.161534	100.168687	99.8945902
100.026126	100.435291	99.9485443	99.8330591
100.019357	100.305442	100.014299	99.9598994
99.7754985	99.9392752	99.9362942	99.8895975
99.9764282	100.079076	100.028891	99.9423577
99.8392687	100.010044	99.7078646	100.027233
100.049996	100.174172	99.9244043	100.088224
99.8072055	100.05001	99.9798903	99.9540972
99.9545183	100.185387	99.9654784	99.8956697
99.8447828	99.5031516	100.708574	100.119572
99.7116901	99.293633	99.9302258	100.150679
99.6318345	98.9516035	99.8185852	99.8970166
100.080289	99.3425982	99.8123132	99.9822775
100.049761	99.4079819	100.227516	99.8941887

99.7022157	99.0913794	100.297762	99.9947203
99.8122992	99.3221113	100.037894	100.034876
99.8198185	99.5277068	100.289817	99.9486256
99.7398125	99.2003522	100.071763	99.9148323
99.8235782	99.2218562	100.026605	99.884998
99.9359174	99.3237096	99.8597709	99.9672897
99.7542497	99.0534568	100.293789	99.7639642
99.8819284	99.4056572	99.9643033	99.9022482
99.7799659	99.3699141	100.092251	100.160718
99.8208712	99.1200029	100.002353	100.173302
99.5626564	98.8911598	100.099777	100.166091
99.8434293	99.3059833	100.104168	100.123531
99.6820638	99.2728556	100.093296	99.9913268
99.8330526	99.6018084	99.6975367	100.216286
100.009908	99.64903	100.085979	100.201015
99.9787778	99.9094026	99.8879947	99.9513121
99.976522	99.5866975	100.271838	99.9146909
99.891854	99.7190632	100.031831	99.8073724
100.03397	99.6901491	100.245704	99.8775041
99.8405719	99.6997387	99.927926	100.168636
99.682515	99.3177524	100.065491	100.107836
99.8438805	99.5638858	100.117548	100.386101
100.215036	100.097127	100.330794	100.239333
100.241053	100.282671	100.560765	100.097656
100.370837	100.577479	100.397276	100.103029
100.481372	100.579223	99.9293895	100.005042
99.9960723	100.194476	100.10354	99.9080453
100.182402	100.263492	100.126956	100.184048
99.9229841	99.8552067	99.998799	100.006456
100.148565	100.286449	99.9873004	100.150254
99.8942602	99.8671211	100.09685	100.046329
99.9491515	100.019393	100.211418	99.9030965
100.169469	100.150741	99.9354524	99.9950031
100.060588	100.068794	99.7090353	99.9175188
100.260603	100.245184	99.9402609	100.236081
100.073522	100.132143	99.8610253	100.174291
99.7422187	99.9201546	100.416928	99.9621995
99.9781763	100.132579	99.9705753	100.00377
100.019382	100.012128	100.221662	99.9745008
100.140143	100.19186	99.9206088	99.9671483
100.151873	100.279765	99.9904364	99.8514875
100.157287	100.320594	99.8129404	99.8598298
100.120292	100.094511	99.6787209	100.166373
99.9948692	100.173408	99.6860381	99.8506391
100.268574	100.239372	99.6599051	99.8792008
99.9157656	100.110784	99.8125223	99.8311267

99.8190666	99.967812	99.986046	99.958806
99.679808	99.9298895	99.9170547	100.134701
99.8652355	99.9941108	99.6780937	100.062731
100.103599	100.128365	99.7136347	100.176412
100.09608	100.128801	99.992318	100.03021
100.238196	100.361422	100.13051	99.9169532
100.082244	100.107152	100.240269	100.286983
100.10014	100.151322	100.218735	100.025262
100.151573	100.12575	99.9201906	99.9658758
100.187515	100.273517	99.530494	99.8348029
99.8489936	99.9861195	99.8292475	99.8584158
100.203456	100.21874	99.8334288	99.9777529
99.7362032	99.6982857	99.9484144	99.8616679
99.9918615	100.076204	100.098941	100.308192
99.8774168	99.950667	100.141172	100.252483
99.8289921	99.9035907	99.7838804	100.056368
100.114277	100.187937	99.9994262	100.018757
100.013066	100.007188	100.284172	99.8372066
99.9589267	100.064435	99.9254173	100.042936
99.8407223	99.8865909	99.9224904	99.9463633
99.9323082	99.9265476	100.028277	100.028796
99.9419329	100.105263	99.6226915	99.9621995
99.9410306	99.9540088	99.9918999	100.04237
100.027353	100.171809	99.8790049	100.034311
99.9483996	100.05252	100.004653	100.004618
100.100291	100.338029	99.7767722	99.9196397
99.9276461	100.234287	100.102077	99.8070896
100.015923	100.178057	100.002771	99.806524
100.012013	100.294585	99.785762	99.8611023
100.101644	100.29662	99.9856279	100.162414
99.9094493	100.141587	99.7192794	99.8775041
99.9264431	100.205228	100.214972	100.168211
99.9850941	100.053537	100.061727	100.01805
99.9261423	100.110494	100.005698	99.8483768
100.074274	100.348927	99.921445	99.9240229
99.9909592	100.260441	99.7709184	99.8161388
100.380612	100.37508	99.8553806	99.9860952
100.227819	100.429421	100.078244	99.705851
100.34467	100.539266	99.9693209	100.092848
100.057581	100.442789	99.876078	99.768913
100.101343	100.405447	99.9668121	99.8247639
100.087658	100.308098	99.8254843	99.8328234
100.051415	100.238501	100.157061	99.8017166
100.009306	100.137664	100.11044	100.058348
99.867792	100.118631	100.126119	100.033462
100.056528	100.277731	100.341038	100.067963

100.023894	100.391353	99.9538501	99.975632
100.078184	100.298944	99.9824919	100.07376
100.21278	100.370285	100.163333	99.9685623
100.369172	100.730789	99.851572	99.7971374
100.106681	100.589864	100.085701	99.8378357
100.093587	100.607789	100.053509	99.9197976
100.095844	100.496447	100.015253	100.079341
100.153641	100.473713	100.192732	100.109158
100.230702	100.579079	100.017344	99.8395315
99.9934973	100.47211	100.27844	99.8792406
100.099156	100.637956	100.541208	100.080613
100.084707	100.542354	100.159494	99.8634135
100.296325	100.684883	99.8549167	100.051078
100.279317	100.6467	100.052463	100.021402
100.189312	100.470069	100.568593	99.771277
100.380309	100.820562	100.146742	99.8530976
100.10051	100.608809	99.7516489	99.8180518
100.080342	100.559842	99.9999933	99.8562065
100.07101	100.626152	100.133991	99.7448513
100.328082	100.979122	99.9640377	99.7750924
100.188559	100.829744	99.90885	99.8304874
100.106681	100.597296	99.8124807	99.9132972
100.223026	100.674827	99.7136029	100.041186
100.060023	100.592487	99.9765803	100.11156
100.287745	100.748569	100.105979	100.148443
100.021643	100.228295	100.811502	99.9607786
99.9451834	99.8364131	100.44024	100.226166
99.8625531	99.3595679	100.23036	100.192533
99.86752	99.4652258	100.35934	100.115658
100.172304	99.6006137	100.153223	100.152683
99.8800123	99.3503866	100.230151	100.110006
99.8917522	99.6759588	99.9462689	100.040197
99.8243234	99.500348	100.10577	100.050654
99.6780272	99.5310981	99.8093451	99.9630396
99.6455169	99.3178876	100.020898	99.9491909
99.7501217	99.3624826	100.27405	100.039914
100.152888	99.9779219	100.204229	100.095592
100.049036	99.7386249	100.147787	99.962757
100.109089	99.7259459	99.894217	100.099973
100.006291	99.8761988	99.7485132	100.104919
99.7344686	99.8085777	100.197122	100.065209
99.9734794	99.762234	100.186461	100.027337
99.9313364	99.5258516	100.115177	99.8685008
100.160414	100.046126	100.088419	100.104495
99.9772422	99.8419511	100.040966	100.324096
100.116314	100.167086	100.051	100.314911

100.038199	100.171604	99.7809151	100.076656
99.9417217	100.225526	99.6552796	99.9754752
99.7585503	99.7491178	100.061034	100.036381
99.7532824	99.5386763	100.05539	100.109723
99.8387724	99.5558731	100.104515	100.021826
100.032028	99.6106695	100.508179	99.9863564
100.088469	99.5984277	100.099498	99.9599307
100.343735	99.8906266	99.9964395	99.8238457
100.074622	100.05385	99.6922804	99.756439
100.104574	100.129924	99.7905311	100.197196
99.9200481	99.8485091	99.9721904	100.091211
100.047982	99.9397393	99.8028647	99.9860738
99.718364	99.6303437	100.206738	100.011228
99.8929562	100.065654	99.7800789	100.121452
99.8998797	99.8174676	99.9730266	99.9255915
100.092232	99.9642228	99.7152752	99.9737795
99.9311859	99.8770732	99.9711452	99.919515
99.8888925	100.078916	99.7880226	99.9000137
99.8437393	99.8005623	99.8252324	99.9014269
99.9254665	99.8110552	99.9688457	99.7536128
100.036092	99.8963103	99.7395243	99.8419338
99.9260686	100.121617	100.223252	99.8639787
100.03549	100.268226	99.6791106	100.023381
99.9758876	100.122491	99.481773	99.9812691
99.7719458	100.039276	100.073159	100.106614
99.811229	99.8594393	100.091555	99.9183845
100.078084	100.147558	99.74266	100.050089
99.7639687	99.8954358	99.9711452	99.9335051
100.091781	100.175247	99.8275319	99.9467886
99.9347982	99.8483634	99.8597247	99.9829649
99.8776042	99.8103266	100.032186	99.9739208
99.8858822	99.8880034	100.078594	99.8899805
99.9665559	99.7900694	100.039084	100.131486
99.9235099	99.6229112	100.054972	100.147878
99.8800123	99.8129498	99.927455	99.8930894
99.7389839	99.632384	99.9023697	99.9203629
99.9360023	99.8289806	100.176426	100.089798
99.8792598	99.9000994	100.038875	100.095027
100.021492	100.109958	99.8250234	100.131486
100.128355	99.9374076	99.870804	100.021967
100.297679	100.254236	99.6182788	100.04373
100.390845	100.368346	99.7255184	100.105343
100.367366	100.58622	99.9581844	100.146323
99.7686345	100.056182	100.10138	100.147595
99.81153	99.7097693	99.7675363	99.9801386
100.086663	99.8314581	99.899234	99.9522998

99.9746835	99.691261	99.9203475	99.837977
99.925617	99.7208452	99.7934577	100.056024
99.9304334	99.4528384	100.159076	99.9997812
99.88227	99.600468	99.9642467	100.00289
99.9069537	99.8142614	100.211755	100.109582
100.159811	100.147558	99.9397886	99.9571045
100.066495	99.9973047	99.9650829	100.005292
99.8873874	100.067549	99.9772075	99.8699139
100.28609	100.334681	99.8620242	99.9384511
99.9417217	100.111852	99.7301174	99.8480103
99.9125585	99.7827322	100.162532	99.9932535
100.005085	99.8389036	99.9921643	99.8685758
100.128454	99.9345546	100.040243	100.121465
100.076248	99.7714108	100.047978	100.205997
99.8472633	99.4494776	100.348159	99.9878819
99.7348772	99.4526709	100.157933	100.019405
99.8382363	99.394032	100.074735	99.9675263
100.044654	99.5862049	100.113198	100.196668
100.108143	99.562401	99.7712096	100.105209
100.202776	99.7641535	100.183854	100.024494
100.148614	99.8875274	99.5642605	99.9293597
99.9552863	99.7087079	99.9700061	100.227908
99.9483656	99.7753297	99.8797011	100.273708
99.7991193	99.3210237	99.9582999	100.147616
99.6891405	99.2442416	100.278967	100.114539
100.094904	99.6751792	100.363418	100.528576
100.239637	99.9859361	100.439927	100.185076
100.249265	100.287694	100.316385	100.138004
100.258894	100.226733	100.172356	100.448426
100.082416	100.024109	100.089786	100.112842
100.163509	99.9643094	100.252418	100.218861
100.01682	99.9320871	100.235486	99.8234826
99.9271522	100.066202	99.9043677	99.8304091
100.037582	100.061412	99.6844582	100.002866
99.9069919	99.7937632	100.027074	99.7858813
100.071735	100.067943	100.108181	100.07411
99.861556	99.8793992	100.045887	99.8133048
100.115666	100.085651	100.160441	99.8868109
100.239787	100.19393	99.7636841	99.8873764
100.029308	99.9476177	100.091667	99.9895782
99.9646142	99.8776575	99.8941248	99.8080745
100.106037	99.9545847	99.9969722	99.7371128
100.085275	100.139936	99.7302378	99.72849
99.9844736	99.8513861	99.8909892	100.017284
99.9176738	100.055461	100.013068	99.9097109
99.8624587	99.9477628	100.101492	99.7793789

99.9424981	100.053139	100.043797	100.033682
99.7169737	99.9062512	100.250746	100.00852
99.9393386	100.075636	100.078916	100.004138
100.020882	100.090441	99.990701	99.9572072
100.225494	100.341979	100.083933	100.092063
100.044353	100.112068	100.013277	100.066477
99.9173729	99.9045094	100.19995	100.224232
99.9534809	99.817422	99.6512209	99.9351554
100.096709	99.7667661	99.9415768	100.030148
99.9757475	100.130501	99.6606277	99.98873
99.7231421	99.8804152	99.6073226	99.940527
100.035777	100.028028	99.5375034	99.8897795
99.8447056	99.9532784	99.7862604	99.9050461
100.231964	100.101327	99.9890287	100.061247
100.288984	100.325867	99.9196276	100.128674
100.169828	100.137468	100.320774	100.004279
99.9781547	100.057928	100.294435	99.8352153
100.01336	100.16461	99.9261078	99.8962819
99.9977132	100.121212	100.016204	100.099837
99.938135	99.9608259	99.9593451	99.8992504
100.059548	100.147628	99.9204638	99.9690813
99.9698799	100.091022	99.9806671	99.9184751
99.9819159	100.002483	99.9037406	99.9621547
99.6888396	99.8284531	100.151661	99.9911331
99.9282053	99.9734536	100.019758	99.9311973
100.08362	99.8750448	99.6652265	99.8928893
100.066469	99.9413764	99.9894468	99.9095696
100.081213	100.199591	99.9654073	100.064498
100.065867	100.087538	100.344187	100.075524
100.142596	100.212073	100.229006	100.178574
100.083319	100.209606	99.826605	100.020112
100.07038	100.108584	100.000944	100.377606
99.9355774	100.160546	99.9507745	100.092911
100.104533	100.165336	100.113198	99.974877
99.9078946	100.101908	100.150407	99.8705548
100.070832	100.266212	100.27395	99.9254017
100.205484	100.395537	100.177582	99.8005826
99.9864294	100.291468	100.342515	99.8834183
100.082567	100.13921	99.9491022	99.9292183
99.9209837	99.7288831	99.7237576	99.9615893
100.150721	99.9727279	99.6869666	99.9706362
100.000873	100.071717	99.7327463	99.8172628
100.022537	100.169981	99.8523169	100.233845
99.8225895	100.021061	100.16943	100.355978
99.9229396	100.180722	100.267678	100.204301
100.074292	100.300757	99.6257181	99.8692825

100.214662	100.241683	99.8098819	100.014316
100.01005	100.072008	99.7910683	99.8858214
100.05699	100.206122	100.114661	99.9996146
100.057442	100.260987	100.102537	99.978835
99.9077441	100.206703	100.084769	99.9740288
99.9245945	100.269261	99.6313621	100.021666
99.8422984	100.190446	100.022684	99.7512486
99.8833713	100.127308	100.085605	99.8223517
99.9306125	99.8756254	99.9281982	99.7183122
100.295453	100.143274	99.6898932	99.8968474
99.9769511	99.5214699	99.5293509	99.8404455
100.015015	99.6056544	99.7806163	99.9002399
100.017121	99.9007356	99.8215881	100.003573
99.9285062	99.9502303	100.303842	100.329686
99.7592501	99.9172822	100.291509	100.043012
99.641147	99.7818613	100.419441	99.8207967
99.7873842	100.028899	99.8328762	99.9249776
99.6467136	99.8558856	100.085187	100.124292
99.6698829	100.009595	100.313667	100.076089
99.8192564	100.39212	100.18885	100.135969
99.9209374	100.229884	99.9698481	100.190255
100.006825	100.358624	100.001821	100.057226
99.9991535	100.558725	100.036719	100.094406
99.8216631	100.286099	99.7989094	100.113067
99.9794491	100.48285	99.9721468	100.262495
100.022769	100.449063	100.070572	100.199161
100.176494	100.68863	100.035047	100.088045
100.196048	100.699698	100.082275	99.9102017
100.356541	100.950043	99.9978503	100.241289
100.178299	100.646251	100.291873	100.430442
100.148516	100.308381	100.470753	100.344348
99.7778922	99.4015201	100.522787	100.171736
99.6659829	99.3913257	100.121143	100.191527
99.9155224	99.5783193	100.693307	100.279177
100.184766	99.6450195	100.177566	100.145158
100.177847	99.450453	100.288112	99.9791901
100.219663	99.7192927	99.7596228	99.8183114
100.028785	99.5353574	100.133682	99.8079914
99.8794227	99.6655539	100.041107	99.9167047
99.8893501	99.4217631	100.630825	99.8728801
100.179652	99.9528891	100.068065	99.9448372
99.9212382	100.038813	99.8983799	99.8928132
100.123096	100.148184	99.6275529	99.9186839
100.128361	100.200466	99.5849227	99.8277831
100.24027	100.452267	99.7600408	100.034042
100.016	100.394013	99.743741	100.034607

99.9149208	100.398528	99.9495779	100.268857
99.6966677	100.206874	100.419973	99.9428581
99.7530736	99.8317219	100.618495	99.9779178
99.7827055	99.3868111	100.343907	99.9497852
99.6646292	99.4990946	100.348713	99.9863999
99.5746807	99.3836071	100.110068	99.8689217
99.6304849	99.4794341	100.143294	99.9431408
99.8833335	99.8598292	100.414957	100.021742
100.003365	99.9777924	99.9717288	99.9473819
100.224326	100.28071	99.7713252	100.078856
100.1428	100.180223	99.8925287	99.9687287
100.260726	100.45984	99.6166864	100.145865
100.011036	99.9116747	100.147056	100.233514
99.6241674	99.3888499	100.091887	100.135686
99.7467561	99.8260422	99.8501075	100.171453
100.005471	99.976773	99.8005812	100.101192
100.26945	100.279545	99.5408297	99.8911168
100.184917	100.416878	99.728695	99.8390927
100.244782	100.156776	99.8718405	99.9821588
100.13543	99.9990549	99.677497	100.043089
100.01239	100.202505	99.6837662	100.032486
99.6793699	99.8339064	100.006627	100.124518
99.5993488	99.6456021	100.121979	99.8861688
99.5432438	99.3659855	100.008508	99.9445545
99.9620009	99.8923055	99.8187617	100.068253
100.017354	99.8550233	99.8005812	99.8942269
100.113921	99.9703651	99.7961928	99.9767868
100.092712	100.059493	99.7092607	99.9162806
100.101887	100.204107	100.076632	100.057509
99.9520734	99.8711886	100.235869	100.004495
99.8734061	99.9217235	99.9088284	100.02768
99.794137	99.7482738	100.123233	99.9171288
99.9058958	100.139009	99.6676754	99.8150599
99.8795731	99.9534716	99.9000516	99.7645909
100.288252	100.156339	99.9286807	99.7022469
100.542154	100.126193	99.6871097	99.9801797
100.229891	99.8724993	99.6219106	99.7705284
99.9802011	100.00823	99.9514586	100.11957
100.040969	99.7771092	99.6348669	100.008595
99.9137174	99.6670102	99.9953426	99.9654772
99.6279278	99.6756026	99.8183438	100.095255
99.3194254	99.223847	100.398031	100.037717
99.7180267	99.5767173	100.159594	100.055812
99.6644788	99.535503	100.240675	100.066981
99.9212382	99.9428403	100.09753	100.086348
99.9943402	99.9156068	100.099828	100.121691

99.8935618	99.9279857	99.9731916	100.01015
99.957338	99.8100225	100.007881	99.8183114
99.9462072	100.074784	99.8106118	99.9357896
100.03044	100.102018	100.189268	99.9400307
99.9863682	100.230612	100.0223	100.025277
99.804967	99.9464812	100.366685	99.8385272
99.7094531	99.7168169	100.308173	99.9171288
99.8130894	99.6996321	100.235451	100.035455
100.093765	100.206	99.9295166	99.9370619
99.946508	100.182116	99.8710046	99.95544
99.7867667	99.9884237	99.9838492	100.009302
100.056612	100.053959	99.9284717	100.139221
100.409036	100.411053	100.06911	100.070232
100.386173	100.198427	99.6935878	99.9004472
100.131218	100.191437	99.9915811	99.8528056
99.8144431	99.9167719	100.197	100.105999
99.9683183	100.013764	100.047376	99.8598741
100.047888	100.125465	99.8329718	99.7949854
100.316831	100.313478	99.8158361	99.845313
100.312018	100.317701	100.08123	99.8967716
100.133023	100.508481	99.8264937	100.008595
100.108506	100.270225	99.9577277	100.194637
100.185067	100.319012	99.9297256	99.9936098
100.376546	100.515909	99.8302552	100.019056
100.325405	100.535715	99.6419719	99.8460198
100.31277	100.40348	99.7897147	100.031214
100.199507	100.242409	99.8687059	100.031921
100.165045	99.6490283	99.9299578	100.04948
100.31082	99.923041	99.8965519	100.136769
100.258804	99.8916256	100.148504	100.019174
100.176296	99.8280674	100.461634	100.113306
99.8738218	99.7467653	100.15756	100.056882
100.137162	99.9340946	99.9217069	100.009258
100.241357	99.8202135	100.089742	100.017079
100.21755	99.7357117	100.073442	100.067497
100.16211	99.9102422	99.9277441	100.147663
100.22179	99.9362763	100.286354	100.206181
100.127705	100.007397	100.32137	100.126993
100.144011	100.088118	99.7810402	100.182578
100.046012	99.8916256	99.9237193	99.8409654
100.252445	99.655864	100.316741	99.9635884
100.267446	99.535438	100.199418	100.044034
100.274621	99.64961	100.107653	100.008839
100.462302	99.5238027	100.065795	100.022805
100.304787	99.5902697	100.043055	100.117077
100.440778	99.8735908	99.6331287	100.16093

100.26712	99.7556373	99.899973	100.046547
100.16048	99.5047498	100.249124	100.005766
100.031337	99.6538278	99.8571089	100.15702
100.04911	99.7044417	100.288366	100.164422
100.169611	99.7543283	99.9895248	100.118613
100.122976	99.5860519	100.039231	100.083139
100.107322	99.6891703	100.018906	99.9688955
100.283752	99.729894	100.29702	99.9077237
100.071123	99.653537	100.33284	100.088865
99.7707685	100.060484	100.205053	100.230761
99.7409287	100.173492	100.031986	100.119172
99.7952273	100.142077	99.8208857	100.141657
99.6020024	100.158657	99.9832864	100.156461
99.7362	100.135532	99.9482706	100.133836
99.9220873	100.100626	99.9104375	100.08691
99.7812043	100.302499	99.818672	99.9249021
99.7963688	100.15313	99.8575114	100.154646
100.065742	99.9668191	100.027157	99.9600969
100.125096	99.9054426	100.013271	100.098083
100.023836	100.094663	100.101616	100.17364
99.9287727	99.8589011	100.189356	100.188444
99.9664393	99.6277937	100.002002	100.024341
99.8995851	100.015688	99.7671546	100.066519
99.9426327	100.247668	100.199821	99.8973887
100.045034	100.176256	100.176678	99.8350996
100.036229	100.26163	99.9239206	99.8876124
99.841373	100.344823	100.044262	99.8398481
99.9908982	100.332751	100.290379	99.7570287
99.8886602	100.073283	100.060965	99.73999
99.9856803	100.047685	99.9728219	99.6708574
100.105692	99.9839813	99.9790603	99.692086
99.846754	99.8557014	99.718857	99.8455742
99.9607323	100.068483	99.863951	99.9714095
100.062155	100.216834	100.169635	99.8493451
99.9010527	99.8420298	100.023333	99.7441798
100.02938	100.142077	100.010856	99.8596801
99.9762229	100.137713	100.241679	99.9383096
100.089549	100.028341	100.051104	100.063586
99.7738666	100.128114	100.003008	100.036213
99.9178477	100.159821	100.323181	100.160372
99.9416544	99.8622463	100.177684	100.103948
100.038022	100.137423	99.6941045	100.081323
100.091343	100.347732	100.132003	99.8774171
100.023021	100.132914	100.268846	99.7753244
100.002801	99.8763542	99.8784403	99.8614957
99.9346428	99.9964893	99.7818451	99.8968301

100.044218	100.11677	100.301849	99.8781154
99.9070858	99.956929	99.6800177	99.9904035
99.8676256	100.053357	99.6613023	100.140679
99.8707237	100.013651	100.111074	100.013308
99.9502965	99.9959075	100.029773	99.9211312
99.9044769	99.9343855	99.8752205	99.9480859
99.8381119	99.9409304	99.96115	99.9986435
99.971168	99.9297314	100.213102	99.805631
99.9895937	100.014379	99.823703	99.7802125
99.9881262	99.9636194	99.6854511	99.8758808
100.115312	99.9534384	99.8973569	99.9237848
100.107159	99.953293	99.7196619	99.928254
99.932523	99.9060243	99.5791964	100.029229
100.052371	100.061793	100.14287	99.9982245
99.901868	100.109934	100.021321	100.145009
99.8790397	100.312826	99.7613186	100.19403
99.7430485	100.248249	100.23383	100.040263
99.8469171	100.160112	100.153535	100.00814
99.9277943	100.230069	99.8887036	100.010096
99.6629865	100.126369	99.8661647	99.9575829
99.84121	99.9848539	100.092761	100.010096
99.7531581	99.9652192	99.8699882	100.019174
99.8511566	100.331588	99.8039815	99.9321645
99.8160989	100.42118	99.9321714	99.8352393
99.8553961	100.176256	99.9013817	99.804374
99.9339906	100.191091	99.7939195	99.8580041
99.7826718	100.070956	99.7462256	99.9582813
99.8973023	100.020633	100.249929	100.026017
99.9946485	99.9445665	99.925933	100.073223
100.126074	100.304099	99.8271241	100.104926
100.110094	100.201999	99.956119	100.074619
100.191298	99.85032	99.925128	100.126294
100.220648	99.7649456	99.990531	100.040402
100.150207	100.173347	99.7921084	100.196125
99.9703527	100.271229	100.181709	100.062748
100.093697	100.180867	100.002909	100.112821
99.9401309	100.272752	99.6913263	99.8582656
100.01398	100.237367	99.974327	99.9770953
100.103804	100.229067	99.9354798	100.047053
100.019033	100.413711	99.7182979	99.9681586
99.8682386	100.397256	100.000695	99.8938726
100.000449	100.283674	100.124885	99.8831207
100.012675	100.459872	99.6685815	99.8518424
99.9275783	100.296051	99.794382	99.9064398
99.7691217	99.9831177	100.118041	99.862315
99.8144416	100.01064	100.013979	100.026945

99.9678445	100.251055	99.8267882	100.182639
100.11114	100.151598	100.324153	100.151221
100.044954	100.169072	100.05806	100.008094
99.901984	100.339737	99.8954249	99.8384374
99.9463257	100.424487	100.043568	99.6602627
99.9523575	100.237076	100.167154	99.6784153
99.8780198	100.218	99.7579502	99.8711121
99.917471	99.9957865	99.9175658	99.814839
100.010882	99.7504197	99.7706309	99.7623361
99.833026	99.7799802	99.8869711	99.9889643
99.7229867	99.7780871	100.154473	100.155829
99.917797	99.7566812	99.7780783	100.204282
99.9730612	99.7447405	99.7670078	100.129158
99.8649782	99.8497313	99.7873372	100.287225
99.8555229	99.9168614	99.8891852	100.140888
100.045769	100.117378	100.098517	99.8010151
99.9533356	99.9893793	100.273028	100.059201
100.049518	99.9037557	99.8650315	99.9053227
100.011534	99.6840177	99.8445009	99.7282651
100.195422	99.774301	100.028471	99.767363
100.353227	99.9772929	99.6261113	99.7254724
100.372137	100.052286	99.7655989	99.7432061
100.253784	99.8916694	99.6713995	99.7270084
99.8385687	99.7255189	100.043165	99.8813054
99.7973244	99.9321513	100.303824	100.098857
99.9774628	100.137036	99.7859282	100.224948
99.8379166	99.9675365	99.9727167	100.075119
99.8152567	100.056509	99.6315459	100.175796
99.8873121	100.057237	99.8092768	100.154432
99.9988185	99.8747777	100.342872	100.076935
100.084731	100.117378	99.9099171	99.984496
100.11489	100.093351	100.09107	99.8551936
99.9285564	99.8907957	99.8946198	99.899458
100.132985	100.287606	99.5971269	100.075817
99.994743	100.018212	99.916962	100.042026
100.09924	99.9925829	99.9211889	100.15918
99.8739443	99.8877377	99.9817744	100.084335
99.9955581	99.9129297	99.9354798	100.283595
100.117498	99.8682248	100.324153	100.259857
100.017403	99.8532262	100.032497	100.301887
99.925133	99.8463821	99.7015916	100.20889
99.8449265	99.8877377	100.236193	100.152058
100.112933	99.9541396	99.9853974	100.049147
100.093534	99.7387702	99.8088742	100.010747
99.9887112	99.9531203	99.8791212	99.8687383
100.030608	100.199652	100.34891	99.8931745

100.067124	100.352114	100.054839	100.049008
100.053105	100.478365	99.8078678	100.019963
100.124834	100.273044	100.093284	99.9254302
100.034194	100.03787	100.338041	99.9754197
100.065494	99.9866125	100.113211	99.9216601
100.039737	99.9638961	99.9322593	99.8969446
100.101033	99.8816217	100.225727	99.9110478
100.064842	99.7217328	100.143403	100.05557
100.057506	100.084031	99.724135	100.153874
99.8666084	99.9210843	99.6033666	100.068556
99.9768107	99.8543911	100.110191	100.191994
100.002405	99.8931256	100.210228	100.090898
99.9089939	99.8478383	99.8783161	100.162671
99.9657252	99.9270546	99.7446657	100.320598
100.060441	100.067285	100.236797	100.122875
100.056202	99.8519156	100.148032	100.194647
100.229004	100.309011	99.8519483	100.259298
99.9800711	100.170965	100.061079	100.056967
99.9528466	100.123057	99.9531925	100.027504
99.9208944	100.00365	99.6794507	99.9044849
100.056691	99.9419077	100.017401	99.7411116
100.014958	99.705278	100.326367	99.6301015
100.381429	99.7984737	100.817895	99.636385
100.336761	99.7012007	100.474107	99.6481144
100.782298	100.356629	99.7203107	99.8049249
100.355346	100.085196	100.005123	99.9763971
100.099892	99.9244335	100.125288	99.8892647
99.9590414	100.239988	99.7808962	99.7127656
99.6647881	99.8364801	99.7559374	99.7588452
99.8455786	100.084905	99.6242998	99.9241735
100.021641	100.249162	100.008343	99.8017133
100.012675	100.207661	100.281884	99.9645281
99.7438534	99.8941449	100.222909	100.221038
99.9044293	99.8864271	100.062085	100.342521
100.003383	100.166014	100.365214	100.152617
99.8545448	99.9873406	100.099926	100.202048
99.7877061	99.8290535	100.093888	100.225646
99.8604136	99.847547	100.26719	100.04412
99.9067116	99.989088	100.082213	100.025409
99.8348192	99.9172982	100.039139	100.03672
99.8842146	99.8669143	100.451564	100.089222
99.9955581	99.6968321	100.096907	100.185711
100.125551	99.885557	100.02913	100.298938
99.8996016	99.895594	100.38534	100.045831
99.9196679	99.803661	100.16364	100.095838
99.9777459	99.6912178	100.048259	100.098492

100.095044	99.6229954	100.058529	100.168334
100.110542	99.7911512	100.470113	100.037869
100.147412	99.8798839	100.282443	99.9617413
99.9524591	99.6919451	99.9675128	99.9500079
100.006132	99.6619796	100.03356	100.058123
100.029951	99.670562	100.609254	99.9838114
100.125878	99.6429239	100.066382	100.041361
100.172699	99.823735	100.068395	99.856699
100.090802	99.8461364	100.049266	99.9180202
100.037782	99.660525	99.9516052	99.8881279
100.061437	99.4903327	100.480987	99.9477729
99.9540905	99.542845	100.645298	99.8734611
99.8387502	99.706055	100.165049	99.8965089
99.7377662	99.8775565	99.9584515	99.8709467
99.4571644	99.8692651	99.5788836	99.9146678
99.8888343	100.254161	99.6761416	100.123775
99.8131371	100.139682	99.6155316	100.273656
99.6625584	100.070005	100.14149	100.123495
99.6033384	99.9860723	100.023492	100.1126
99.8110163	99.9188681	100.50072	100.088295
99.8155842	100.00411	99.7621233	100.030466
99.8529434	100.074369	99.9840246	99.9856273
99.8772513	99.917559	99.9369058	100.140816
99.7917657	99.6640161	99.8128666	100.17881
99.7506543	99.8721743	99.938718	100.104638
99.8599584	99.8587917	100.393595	100.036472
99.9780722	99.8320264	100.346275	100.088574
99.8584902	99.859519	100.03658	100.129781
99.9274986	100.016329	99.7383626	100.035355
99.8382607	100.017783	100.143101	100.027951
99.7896449	99.9598888	100.1725	100.073069
99.828146	100.06055	99.9185818	100.242366
99.92913	99.9729805	100.103634	100.080054
99.9498489	99.7558036	100.037386	100.042758
99.9379396	99.9009761	100.01	99.8931565
99.8968282	99.877702	100.134845	99.9329664
99.9800299	99.7834416	100.080276	99.9114551
99.7824667	99.9219229	99.7007078	99.9444205
99.8410341	99.8758109	99.9946968	100.040942
99.8255358	99.8887572	100.175721	99.8828199
99.9860661	100.128917	100.133637	99.8583752
100.059806	100.503049	99.7295026	100.001132
100.066658	100.449373	100.200288	99.8420322
100.149696	100.302746	100.231499	99.6639351
100.030767	100.293728	99.9121382	99.7548694
100.085256	100.375478	99.8563609	99.822197

100.219683	100.179102	100.199684	99.8284828
100.122778	99.8672286	100.158002	99.8854739
100.272541	100.178666	99.9652979	99.8913406
100.164379	99.9229411	99.9207968	99.9576905
100.022936	99.6401601	100.231096	99.8818421
100.121473	99.8601009	99.9872463	99.9276584
99.9589847	100.143464	99.6870152	99.8680134
100.083135	99.9366147	99.7993753	99.977805
100.020652	99.8971941	100.070409	99.8579561
100.157853	99.871447	100.03658	99.8772325
100.175472	99.917268	100.031345	99.9145281
99.9235833	99.8606827	99.9624788	100.052396
99.9004173	99.8371176	100.112494	100.044993
100.107769	99.8782838	100.273382	99.8400766
100.060458	99.8887572	100.360371	99.8242923
100.121962	100.379406	99.7566865	99.8562799
100.048549	100.575345	99.8485077	99.7407613
99.9876975	100.378242	100.231096	99.8212193
100.023099	100.080478	100.006174	99.9099186
100.293423	100.136191	99.6161357	100.109527
100.221315	99.8999579	100.12075	99.8357464
100.195702	99.9562522	100.069201	99.8734611
100.16601	100.190448	99.8110544	99.873042
100.072367	100.16281	100.101217	99.8026413
100.107606	100.1471	100.008188	99.864661
100.055564	100.387842	99.7377585	100.009094
99.9527854	100.244852	100.073027	99.888966
100.150512	100.090515	100.135852	99.9391125
99.9950388	100.318165	99.6783566	99.8478989
100.072694	100.582182	99.7975631	99.8682927
100.078404	100.345949	100.208342	99.9166234
99.8764356	100.262307	99.8768998	99.9495888
99.9653472	100.122372	99.7723928	100.008954
100.091944	99.9485427	100.060341	99.9748716
100.144639	99.9227956	99.8662276	99.9666302
100.19146	99.9795264	99.71581	99.945957
100.276293	99.7768958	100.0247	99.9421856
100.362431	99.8848297	99.9491889	99.9365982
99.9188522	99.9374874	99.8462928	99.9793415
99.8508226	100.097934	100.096989	100.186493
99.9978122	100.039166	100.25244	100.199064
99.8961756	100.315402	99.639091	100.289999
99.9664892	100.305801	99.6910424	100.409847
100.05589	100.097206	100.025908	100.350202
100.031419	100.063604	99.885357	100.384844
99.9152631	99.9645437	100.002751	100.433733

99.9299457	99.8107888	100.240359	100.32017
100.145291	100.028693	99.5810986	100.445327
100.22784	100.032475	99.8392451	100.308996
99.9865555	99.7427118	99.8775039	100.222531
100.233713	100.011819	99.4620935	100.329529
100.213095	100.097557	99.6850143	100.234546
100.007901	100.043999	99.6630834	100.178266
99.8509497	99.8783761	99.484215	100.119472
99.8656181	99.9041365	99.8011078	100.189857
99.9607994	99.9881123	100.168301	100.003979
99.8638253	99.9013712	99.6139901	100.127572
99.9973073	99.8884183	99.6455788	100.034842
100.349348	99.8063345	99.9381262	99.7227171
100.307299	99.6846642	99.8926546	99.7676854
100.423016	99.9048642	99.8713272	99.9023111
100.514286	100.061755	99.7025189	100.021575
100.38553	99.789452	99.5984976	99.9594293
100.522761	100.00907	99.641756	99.9740928
100.517708	100.205693	99.5339118	100.049925
100.365646	100.146167	99.7226391	99.8732632
99.9845948	99.8785217	99.9540211	99.8778718
99.9673187	99.7926539	99.9381262	99.9359675
99.9570508	99.9836006	99.7578494	100.016687
99.3763468	99.7911985	100.394452	99.9921081
99.9560729	100.14966	100.180172	99.9394589
100.254819	100.102215	99.7598614	100.137208
100.531725	100.157665	99.7178102	100.021854
100.264109	99.9996098	99.6721374	99.8750787
99.8310659	99.9684646	99.4182208	99.8634875
99.6491783	100.05637	99.6914528	100.146285
99.7468043	100.086933	99.6045336	100.12031
99.884361	100.321832	99.8254532	99.8005039
100.104223	100.129576	99.8258556	100.022832
100.222222	100.097703	99.9771593	100.169887
100.049787	100.012272	100.313569	100.127432
99.9220098	100.031483	99.8890329	100.225748
99.7750002	99.9274227	99.7419544	100.191533
99.8082485	99.8959863	99.7968825	100.244601
99.8648031	100.018093	100.229466	99.9739532
99.9417305	100.014891	100.109751	100.014732
99.9813351	100.213406	99.754429	100.179942
99.9744899	100.068886	100.13973	100.131482
100.025503	99.9117045	100.658831	100.073666
99.9834539	99.8090997	100.222625	100.28133
100.158659	100.181388	100.087217	100.287195
100.003175	100.21981	100.299283	100.236501

99.9453161	99.9518732	99.9964747	100.260941
99.8807754	100.114294	99.785414	100.196141
99.8828941	100.125355	99.7650927	100.264013
99.8237317	100.115168	99.9453694	99.9859634
99.9018	100.041671	100.085205	100.001744
99.8939769	99.8952586	100.026856	99.9809359
100.035119	100.14282	99.8308856	100.095033
100.093304	100.07398	100.016796	100.139163
100.097541	99.8112828	100.52141	100.121148
100.169905	99.8961318	100.047781	100.151732
100.017843	99.8085176	100.071322	100.225888
99.8783306	99.9028266	99.5982964	100.045316
99.9638961	100.097121	99.7785732	99.7229964
99.919565	99.900498	100.032087	99.7498098
99.9275511	99.7942548	99.761471	99.7935213
100.025177	99.8318038	99.6568461	100.096848
100.021918	99.7977477	99.9127748	100.003141
100.229393	99.870226	100.120817	99.9228401
100.25547	100.035558	99.7357172	100.003839
99.8639882	100.083295	99.9017087	99.9062214
99.6010986	99.7094058	100.775528	99.8059504
99.8652921	99.7245418	100.767681	99.6005205
100.368417	100.158975	100.270713	99.741291
100.232327	100.063647	100.365881	99.5545746
100.272584	100.075144	100.013979	99.7986884
100.020288	100.212824	99.6703266	99.744084
99.9184242	100.179205	99.5846147	99.9201867
99.937819	100.152716	99.6421584	100.065286
99.8465492	100.136998	100.167697	99.8942112
99.8983774	100.163777	100.423022	99.9531449
100.011487	100.074853	100.422016	100.005654
99.7102964	99.9360094	100.24355	100.153268
99.7999364	99.8974417	100.029673	100.320852
99.9332555	99.9894221	100.26186	100.277839
99.9500426	100.01038	100.012772	100.003979
99.9389599	100.100905	100.221619	99.7780198
99.8087374	100.112693	100.13631	99.8087435
99.929018	100.014309	99.9071411	99.8010625
99.8812643	99.8377708	100.151802	99.9041266
99.8936509	99.8919112	99.9514055	100.07185
99.8543723	100.016783	99.9532163	100.062773
99.9585177	100.128994	99.8467806	100.151871
99.9640591	100.04691	100.370509	100.056069
99.8426376	99.8449022	100.480365	100.104808
99.9461311	100.045163	100.115787	100.116958
99.8185163	100.074999	99.9872194	100.102434

99.7823344	99.7917806	100.114781	100.14852
100.072279	100.111384	100.010961	100.26513
99.9133717	99.9617698	100.295662	100.058304
99.8977255	99.7655837	100.445959	100.018363
99.8154197	99.9709387	100.047781	100.13637
99.8154197	99.9991732	99.8369217	100.050064
99.9929068	100.01853	100.271518	99.8430981
100.01442	99.8559632	100.09285	99.7236947
100.006108	99.9448873	99.9946639	99.8863906
99.9883433	100.165961	99.7978885	99.8933733
99.9844318	100.066994	99.9735377	99.8080452
100.065434	100.116041	100.154619	99.7630769
100.021266	100.023769	100.091442	99.7465978
99.8213855	100.208079	99.9292943	99.9056596
100.003333	100.176854	100.027231	100.193931
99.6394378	99.8997722	100.464877	100.150035
99.4753479	100.048174	99.9695897	99.9155289
99.5794625	100.008896	99.9813315	99.9195061
99.4911841	99.778816	100.202823	100.093323
99.4460342	99.8887612	100.653278	100.164913
99.5100528	99.8412662	100.567083	100.191722
99.5675011	99.8588509	101.010067	100.214554
99.4573216	99.4719884	100.457939	100.307649
99.4556369	99.7697772	100.242585	100.211608
99.7416991	99.6501358	99.9252914	100.099068
99.6461766	99.5257284	99.6971283	100.381301
99.7219881	99.4599913	100.564148	100.935896
99.3385502	99.0165949	99.8695182	100.083454
99.4323881	99.5827553	100.3576	99.7022344
99.3082256	99.1365651	100.271405	99.6072241
99.7231674	99.7447971	99.8996731	100.085222
99.6901473	99.832063	100.401899	99.7559999
99.5103897	99.7355939	100.645273	99.9544169
99.7794364	99.8705192	99.9052771	100.194521
99.6402802	99.7587662	100.112625	100.078446
99.6608335	99.8746278	100.070996	100.37202
99.9100007	100.058527	99.7219461	100.199382
99.8042015	99.9498967	99.7334209	100.172278
99.9479065	100.123936	99.8962039	100.093323
99.9408308	100.351221	99.982132	100.10172
99.7191241	100.048338	100.294355	99.9647281
100.204655	100.206271	100.091277	99.85867
100.038712	99.967317	100.572154	99.8585227
100.206003	100.107008	100.209495	99.80078
100.264125	100.049324	99.907412	99.6036888
100.138952	100.104543	99.7953318	99.6107593

99.9721662	100.18918	99.7761181	99.9987551
100.210551	100.448677	99.8414982	99.8352489
100.235316	100.417123	99.9031422	99.8199294
100.085378	100.231416	99.9258251	99.7646908
99.9689653	100.05294	100.297024	99.8488007
100.131033	100.277596	100.260464	100.029247
100.059434	100.153682	100.313035	100.228989
99.9062944	99.9594286	100.365606	100.072701
99.8232387	99.8442244	100.075532	99.9050704
99.9388091	99.9867095	99.7222129	99.8896036
100.018495	100.03404	99.6781814	100.126025
100.000469	99.9954196	99.547955	99.9134667
100.26463	100.314737	99.7112718	99.9109625
100.253174	100.368313	99.7094038	99.7393547
100.14569	100.198876	100.063257	99.697668
100.272043	100.183592	99.802537	99.52061
100.309949	100.329693	99.95598	99.7733817
100.19421	100.15286	100.438191	99.664083
100.04663	100.038806	100.842747	99.8012219
100.064151	100.154339	100.277276	99.9280497
100.199264	100.201341	99.7531684	100.033519
100.312139	100.394608	99.4796395	99.900504
100.12918	100.255903	100.112092	99.7425954
100.13255	100.199369	99.7494324	100.059002
99.9691337	100.011525	99.6063968	100.184651
100.041576	100.038806	99.845501	100.31855
100.017485	99.9758628	99.9453057	100.186714
100.043934	100.111774	99.6576334	100.202328
99.9733455	99.9707682	99.7102043	100.230904
99.9689653	100.06165	99.4887127	100.197025
100.044945	100.138726	99.6069305	100.258008
99.9666067	99.9709326	99.6829849	100.215879
100.159842	100.155654	99.7470307	100.312657
100.333366	100.23651	99.9495754	100.400008
99.8931537	99.9844087	100.172135	100.255946
99.8699049	99.9416796	99.8254867	100.340203
99.7937564	99.778816	99.9413028	100.370695
100.001648	99.8867891	99.9255583	100.498701
100.163885	100.106351	100.033902	100.209987
100.168602	100.028781	99.9234234	100.010687
100.039386	100.078084	100.665287	99.8006327
100.182417	100.023358	100.499302	99.7004668
100.298661	100.113746	99.8884651	99.6676182
100.257049	100.005773	99.7219461	99.6886825
100.059771	99.9717543	99.8345599	99.9010933
100.092791	100.039299	99.8054724	99.7210892

100.097171	100.018592	99.8729873	99.6992883
100.155125	100.094683	99.9538451	99.7803049
100.160179	100.224678	99.8975382	99.8966742
99.9276901	99.9107832	99.6784483	99.8706016
99.9435263	99.8667393	100.10035	100.098774
100.16776	100.12147	100.04004	100.12028
100.210046	100.018921	100.066993	100.285112
100.190166	100.038642	100.457405	100.340203
100.102562	99.9066746	100.728532	100.483381
100.277434	100.106844	100.140378	100.134568
100.255364	100.212023	99.926092	100.066956
100.299841	100.161406	99.7200781	99.6584855
100.183259	99.9337911	99.7230135	99.7593879
100.05438	99.9134126	99.4980527	99.9497032
100.081335	99.8789007	99.7078026	100.16668
100.131876	100.098791	99.6456248	99.7110726
100.086726	99.9395431	99.7937307	99.7031182
100.177194	99.9993638	99.8695182	99.7561472
100.14131	99.9664953	99.903676	99.9050704
99.8913006	99.6501358	99.8265542	99.6766037
100.093465	99.8277901	100.168399	99.6223962
100.514166	100.181128	100.280959	99.7186905
100.444048	100.154427	100.244378	99.8308913
100.373929	100.04136	100.519673	99.8942067
100.404511	100.150307	99.9533294	99.9997815
100.418704	100.243596	99.946654	99.8311858
100.443879	100.228102	99.8321036	99.9906523
100.475981	100.28579	99.7941872	99.7968777
100.503184	100.394078	99.5843117	99.749612
100.244506	100.008395	99.7613441	99.9996343
100.500649	100.24376	99.6003328	99.8158723
100.414818	100.165965	99.9325021	99.6941006
100.198548	99.8374754	99.7720248	99.6117905
100.01303	99.6614461	100.377887	99.8276519
100.107986	99.7735247	99.9071355	99.8784515
100.375112	100.066412	99.931167	100.189875
100.300431	99.9439501	99.9341042	100.185016
100.260557	99.7145186	100.689763	99.9953642
100.239775	99.9174138	100.105796	99.6023668
100.382884	100.136462	99.7765641	99.4696989
100.469054	100.104321	99.790716	99.2843173
100.310231	100.099212	99.8654808	99.5478861
100.482402	100.093278	100.932481	99.9719522
99.9462906	99.8872515	101.167189	99.6580255
100.440838	100.504508	100.587228	99.8622545
100.410594	100.627794	99.8969888	99.8568065

100.034657	100.130363	100.673475	99.7555018
99.7227561	99.9965281	100.7178	99.7923131
99.918919	100.267165	100.974136	100.043366
99.8028433	100.305568	101.256373	100.170586
99.8288632	100.27623	100.125021	100.282787
99.5166245	100.005099	100.107398	99.9402945
99.3434401	99.5706294	100.407259	100.120817
99.484522	99.5439284	99.6230292	100.1351
99.3542536	99.3677342	100.205661	99.924392
99.5105419	99.4944819	100.426751	99.8964154
100.177259	100.041854	99.3309125	100.134658
100.261064	100.360289	99.7426529	99.9429449
99.7908471	100.317106	100.252923	100.046016
99.8368043	99.8546168	100.412065	100.28205
100.466351	100.348257	101.158111	100.037329
99.8342699	100.337873	100.605118	99.4028496
99.795578	100.108442	100.468673	99.5527452
100.28928	100.236014	99.8035328	99.7797972
100.26326	100.144208	99.370431	100.002579
100.151746	100.050919	99.9068684	100.165138
100.075714	99.8346734	99.5931233	100.061477
100.159181	100.066083	100.137304	100.038801
100.329831	100.351224	99.5471963	99.8984768
100.322903	100.184425	99.4406565	100.022752
99.9751828	99.9302699	100.305792	100.154978
99.9266912	99.81473	100.285232	100.212404
99.9897134	99.824949	99.7210245	100.332703
99.6999464	99.6197462	99.8710881	100.049256
99.816867	99.4873946	99.9076695	100.055882
100.254136	99.8338493	99.3504047	100.338004
99.8656965	99.6871582	99.6016678	100.298689
99.6991016	99.4964598	100.036906	100.375404
99.8915474	99.8592318	99.5375837	100.328874
100.119982	99.980046	99.2366554	100.625721
99.6857538	99.5055249	99.6235632	100.499384
99.6830504	99.5831559	100.283897	100.429296
99.9393632	99.8656599	100.231828	100.430768
99.8329182	99.8236304	100.233163	100.425615
99.6065114	99.4776701	100.137037	100.088129
100.030433	99.8399477	99.6644169	100.144671
100.155801	100.406604	99.323169	100.321218
99.6644648	99.7811065	100.100456	100.301192
99.7355971	99.8188506	100.383761	100.17088
100.130626	100.285131	99.5204946	100.325193
99.8315665	99.8732417	99.930633	100.238319
100.083824	100.041689	100.356525	100.09873

100.233354	100.259254	99.7122129	100.10624
100.184525	100.171404	99.3912583	100.111835
99.8454213	99.8628579	100.011806	99.9670931
99.8670482	99.7084202	99.7629462	99.6025141
99.8670482	99.7184743	99.7335743	99.610907
99.7722615	99.6016159	100.043047	99.6455097
99.7163356	99.5871116	100.122885	99.4705824
99.6543271	100.116353	100.77681	99.946626
99.8572485	100.371827	100.683087	100.129652
99.5904601	99.9134581	100.415002	99.6754004
100.162729	100.48374	99.3776405	99.56585
99.9033747	100.124924	99.7501294	99.8657884
99.7932125	100.052732	100.411531	99.8303023
99.9452768	100.115364	99.5864479	99.7116227
100.082811	100.275736	99.868685	99.7494647
99.9211155	100.052897	100.468673	99.9207109
100.053411	100.27178	99.8911144	100.053379
100.138399	100.39655	99.5138192	100.175592
99.667506	100.025207	99.9122088	100.110215
99.7489449	99.9574654	99.9821673	100.244503
100.02114	100.111244	99.1880583	100.192673
99.9680865	100.130858	99.2753729	100.078852
99.8439006	100.011197	100.177624	100.177654
99.8202462	99.8109391	99.4080804	99.9582584
100.104269	100.118002	99.4307769	100.020985
99.9092883	99.928292	100.271347	100.047342
100.135695	100.056523	100.006466	100.056324
100.225413	100.270461	99.4905887	100.302076
99.3294047	100.01716	100.789027	100.146237
99.59625	100.226621	100.871497	99.6117221
99.8521591	100.389114	100.560567	99.7166793
100.112947	101.108138	100.152755	99.9538648
99.7769511	100.718221	99.9899508	99.8925415
99.3292364	100.217228	100.406036	100.088452
99.3985556	100.085552	99.6782203	100.246919
99.3522867	99.8055564	100.122063	100.197094
99.267152	99.6186728	100.707091	99.6907348
99.3151034	99.6381193	100.236826	99.557622
99.6753278	100.201901	99.664075	99.5110399
99.4318693	100.14109	100.128735	99.8831071
99.6408364	100.23206	99.8861296	99.7271455
99.7289997	100.016666	99.8639775	99.6955994
100.229209	100.341982	99.4492265	99.9302789
100.075932	100.063304	99.5658586	100.127958
99.9552967	100.084893	99.8871972	99.9537174
100.441373	100.484204	99.6856933	99.8434534

100.150299	100.155757	99.7083792	99.7872895
100.214571	100.225303	100.122596	99.8459594
100.864859	101.105007	99.9536534	100.085503
100.052882	100.191189	100.024113	100.015041
100.16174	100.134498	100.142346	99.9770084
100.230723	100.185092	100.147951	99.9457571
100.117995	100.173391	100.314492	100.061033
100.161572	100.162679	100.292874	99.8539196
100.162918	100.133179	100.218411	100.249131
100.162918	100.139936	99.9307007	100.363522
100.15602	100.159877	100.166367	100.339936
100.110255	100.154274	99.6176357	100.026539
100.142223	100.102691	99.47298	100.004869
100.088383	100.185916	99.3080404	99.8686608
99.960849	100.120819	99.8236767	99.6490173
100.237116	100.469702	99.5373011	99.7365799
100.099319	100.392246	99.8989404	100.128695
99.9229926	100.077312	99.8268795	99.9548967
100.014857	100.147847	99.9888832	99.9655103
99.9118881	99.884331	100.462351	99.703707
99.9620267	99.9421759	100.311556	99.8247321
100.045816	100.071544	99.7372036	99.72803
99.8827807	99.9664015	99.6315141	99.9801041
99.9238339	99.8894398	99.7006393	100.072973
99.8972503	99.8841662	99.6950346	100.050862
99.7907477	99.8404941	99.7697645	99.9895384
99.9462112	99.9378911	100.082029	100.243234
100.018391	99.983376	99.989417	100.11646
100.01368	99.8993278	100.680669	100.000447
99.9694298	99.8905934	100.450074	99.6706868
99.9978641	99.8343965	100.011569	99.8094013
99.957484	99.9644239	100.018775	99.8318078
99.8673017	99.8568093	99.7420076	99.8267958
99.9098691	99.8680157	99.8735857	99.7147629
99.9549602	99.7936908	99.8418254	99.8953423
100.212888	100.153615	99.9061465	100.150659
100.028822	100.063139	99.6646088	100.226134
99.7289997	99.7037098	99.9549879	100.300872
99.9840676	99.9050958	100.273391	99.8424215
100.166451	100.081103	99.9899508	99.6939779
99.9090278	99.8281341	99.9619271	99.7265559
100.216758	100.072863	100.673196	99.8434534
100.136839	100.238157	100.167167	99.6330968
100.060285	99.9902976	99.7820415	99.7819827
100.127249	100.092309	100.059877	99.8226683
100.043965	100.148341	100.052671	100.198716

99.9714488	100.154933	99.5706626	100.042901
100.088888	100.133344	99.9013425	99.918486
100.252427	100.305231	99.7270617	99.9490002
99.978347	99.997384	100.471425	99.9584345
99.9401542	99.8327485	99.8391565	99.7370221
99.9835628	99.9401983	100.112454	99.9162748
100.131287	99.985024	99.6688791	99.9324901
100.208346	100.111591	100.033988	100.053663
100.166115	99.9474495	100.325968	99.9669844
100.097468	99.8675213	100.370006	99.8696927
100.280862	100.220853	99.6288452	99.6905874
100.204981	100.024906	100.208269	99.786405
100.057593	99.8729598	100.040393	99.8738202
100.061631	99.9952416	99.758555	100.348781
99.9224879	99.7780348	99.6838251	100.378411
99.9287131	99.6905258	99.6851595	100.362785
100.023438	99.979256	99.6654095	100.259597
100.198251	100.180972	99.8332849	100.330944
99.9416684	99.9167966	99.7299975	100.205349
99.9076818	99.8342317	100.057741	100.205497
99.8639367	99.675529	99.9021432	100.167612
99.7567611	99.6043352	100.341715	100.379737
99.9992101	99.7218378	100.166367	100.196947
100.126744	99.8060508	100.223215	100.302346
100.003248	99.8690045	100.243499	100.261071
99.8250708	99.7221674	100.363067	100.139309
99.9487349	99.8788926	100.772747	100.361163
99.9213101	99.8367037	100.656381	100.241465
99.9778423	99.8222013	101.032433	100.293944
100.160394	99.8142908	100.314492	100.294239
100.057088	99.8910878	100.104981	100.194883
99.9253481	99.692009	99.9611264	99.9753869
99.9521	99.8136316	100.020377	100.11189
100.036057	99.9228943	99.6128317	100.135771
99.9512587	99.8048972	99.9659305	100.24117
99.8922028	99.611092	100.060144	99.9946978
100.068025	99.8296173	100.090836	100.018284
100.521258	99.7523268	100.038267	99.8854503
100.356196	99.5553216	99.9787952	100.100489
100.073665	99.1695266	99.6368971	100.045109
99.9938343	99.1825069	99.7939782	100.087233
100.29628	99.4289688	99.9427919	99.9499618
100.346744	99.4554224	99.6280963	99.7062026
100.50303	99.6376398	99.780377	99.7606986
100.323622	99.535276	99.516086	99.834931
100.2605	99.4654451	99.8227809	99.9543803

100.270458	99.440799	100.163612	99.8228535
100.546406	99.8182143	100.7594	99.9233029
100.388432	99.7105926	100.862076	99.6771871
100.233158	99.9008611	101.123167	99.3369552
99.8183076	99.8633989	101.553073	99.1569711
99.9013452	100.088501	100.662858	99.4824743
100.135943	100.182978	100.356963	99.736249
99.6439624	99.9733209	100.139877	99.9272796
99.4145963	99.5094797	100.262021	99.8492178
99.5678446	99.6787168	100.405234	99.7545125
99.7705441	99.9378304	100.268955	100.004016
99.8701218	99.7874887	100.226285	100.041132
99.6323169	99.6685297	100.876478	99.9402408
99.8903748	100.105917	100.170013	99.831838
99.7253122	99.6769094	100.453239	100.002543
99.6336671	99.7963613	100.829807	99.7682102
99.8444679	99.8399029	100.433504	99.5104588
100.2605	100.499764	99.871852	100.509945
100.433326	100.551192	100.210016	100.637495
99.4733302	99.625974	100.729798	100.164411
99.6112199	99.604614	100.342295	100.158225
99.9693618	99.9782502	99.5838256	100.613046
99.8012613	99.9186064	100.422036	100.307279
99.7528227	99.757256	100.001464	100.206829
99.9595728	99.9319153	99.5456887	100.147031
99.955691	99.9698705	100.093739	100.475038
100.198728	100.362731	99.4662147	100.218023
100.317377	100.466409	99.504885	100.301535
100.199741	100.173612	100.362297	100.396534
100.345056	100.423032	99.5923597	100.221264
100.257968	100.248208	99.7393065	100.092682
100.09645	100.008154	100.799137	100.013148
99.9637922	99.8964248	100.106274	99.8908999
99.8419362	100.020313	99.8126466	100.167651
99.9705433	99.9325726	100.092139	100.126116
99.7263249	99.7704007	100.374298	100.146147
99.8787293	100.014727	99.4920838	100.089442
100.032146	100.072892	100.006264	100.195341
100.027083	99.8905097	99.882253	100.384457
100.105564	100.042495	99.0933804	100.371643
99.8535817	99.9169633	99.8630512	99.9879617
99.9847204	100.10181	99.4243442	99.738311
100.017632	100.232763	99.1120488	99.9889927
99.9389822	100.170162	100.425236	99.9178533
99.7934976	99.8652063	100.192682	99.7933962
99.9651424	100.039373	99.9537262	99.9797136

100.120247	100.169505	99.5054183	99.9038611
100.242947	100.238678	99.70437	99.7764582
100.257799	100.40693	99.2424608	99.7568691
100.395014	100.566801	99.4155434	99.7782257
100.03991	100.189879	100.106007	99.7437606
100.062526	100.090144	99.617162	99.8531945
100.063032	100.126292	99.5566231	100.072062
99.8939191	99.8247866	100.248687	99.8154892
100.139994	99.9825222	99.907322	99.7766055
100.384381	100.571073	99.1997902	100.203589
100.045648	100.270554	99.1675206	99.9601245
99.9847204	99.9595191	99.3840738	100.028171
100.091218	100.181006	99.4443461	100.11507
100.240416	100.32839	99.3840738	100.230984
100.574254	100.611329	99.0917803	100.200054
100.516364	100.670479	99.1221831	100.081489
100.272652	100.477089	99.9702611	99.8922254
99.848856	100.025078	100.586584	99.4139861
99.8351852	99.9961597	100.446305	99.3655288
100.267926	99.7171649	100.597252	100.495069
100.139656	99.3685035	101.51387	100.169271
99.906071	99.884759	101.316252	99.9807446
100.0578	100.090801	100.088139	100.225682
99.8761977	99.8773651	99.8294481	100.50317
99.5502919	99.2861852	99.889187	100.152628
99.5308827	99.2968652	99.9163895	100.082372
99.3580564	99.4912415	99.8107798	99.681753
99.6611775	99.5241031	99.9483924	99.4450635
99.9440455	99.9107196	99.7083703	99.9331711
99.7258186	99.7955398	100.109741	99.9748532
99.6097009	99.7707293	99.9347912	99.9137293
99.9519779	100.049396	100.039867	100.007403
99.8554383	99.9223855	100.530046	99.9869307
99.9188979	99.9554114	100.014799	99.9315509
99.9479273	99.9637911	100.060936	99.8627681
99.8633707	99.931751	99.4883501	99.9316982
99.9853955	100.206145	99.3339359	100.191364
100.152146	100.255931	99.0629777	100.100489
100.229108	100.389513	99.6566322	99.9969462
99.9561973	100.229641	99.7998454	99.9657214
99.9607543	100.212718	99.2160584	100.124938
100.021514	100.275319	100.261221	100.153364
100.122104	100.278276	100.317226	100.154837
100.131049	100.303087	99.8366488	100.292256
100.160922	100.222083	99.857184	100.218612
100.869266	100.298594	99.6230862	100.479996

100.500922	100.100512	100.01449	100.415469
101.077884	100.286658	100.064102	100.590848
100.765117	99.8945255	100.123911	100.330961
101.240159	100.238428	100.193524	100.265841
100.750307	100.083575	100.610616	100.367665
100.830728	100.433445	100.354125	100.509004
100.604912	99.9216247	99.7313301	100.000035
100.540256	100.201327	99.9242868	100.452765
100.747759	100.16826	99.795649	100.335401
100.676733	100.257623	99.8470257	100.138266
100.043079	99.8187121	100.150187	99.9392069
100.786775	100.232459	99.7946686	100.225438
100.366674	100.179551	100.013117	100.479108
100.713042	100.264882	99.8985985	100.454245
100.530701	100.156969	99.5642579	100.205458
100.506655	99.938723	99.5621009	100.310241
100.513662	100.229394	99.8772242	100.210786
100.78152	100.143903	99.8338874	100.013207
101.038071	100.041958	99.4320905	99.8934751
100.754288	99.7283814	99.5909267	99.8841512
101.10416	100.121643	99.6117127	100.215522
100.502037	99.9321095	99.8829109	100.20531
100.670841	100.031473	99.7921193	100.15129
100.604116	99.6449867	99.8687921	99.9326949
101.025968	100.169712	99.943504	100.232394
100.638832	99.9108173	99.6989746	99.9004311
100.707309	99.8838793	99.8940883	99.9523789
101.242229	100.319403	99.9758596	99.7571677
100.879618	99.8948481	99.8427116	99.6173084
100.938062	99.9834045	99.9966455	99.5675806
100.669249	100.098415	100.272746	99.6204164
100.852386	100.519905	99.8964414	99.7432558
100.711291	100.14729	99.9076188	99.7312679
100.661923	100.219716	99.9109524	99.692936
100.731993	100.412153	99.5842595	99.5752766
100.57561	100.174228	99.449935	99.7333399
100.414449	99.9734036	99.6275963	99.6575642
100.393587	100.192133	99.8219256	99.8766032
100.388332	100.372795	99.6646581	100.010247
100.676256	100.199392	99.828985	99.8560313
100.342309	100.218103	100.088614	99.9393549
100.528472	100.582491	99.9509556	100.257701
100.376548	100.405217	99.8356522	99.8554393
100.176371	100.320209	100.150972	99.8659473
99.9774679	100.238589	99.9005594	99.8764552
99.9725311	100.178745	99.8032967	99.8102995

100.116015	100.512807	99.9842916	99.9659948
100.070947	100.360374	99.5883775	100.261401
100.086872	100.16955	99.7389778	100.065154
99.9397257	100.293271	99.8085912	100.075366
99.8077076	100.179874	99.3762036	99.9910067
99.9523064	100.146806	100.146658	99.8894791
99.9675944	100.470384	99.9801736	99.9750228
99.889562	100.438446	100.0843	99.8594353
99.7769724	100.261172	100.248627	100.250153
99.9621799	100.082607	100.084888	99.9580028
100.063622	100.364084	100.139598	99.9329909
100.184652	100.09406	99.8191803	99.8911071
99.9015057	100.031473	100.126656	99.8968791
99.8268176	99.9322708	100.176464	100.010839
99.6331699	99.9311417	100.065475	100.115622
100.195003	99.7633846	99.8921274	100.027415
100.056456	99.9456591	100.359812	100.21419
99.700851	99.8029043	100.132931	99.9612588
99.5672405	99.4628735	99.935072	99.8761592
100.03432	99.6528907	100.285688	99.8245074
99.7768132	99.4564213	100.021157	99.711288
99.434267	99.4809396	100.319613	99.8023075
99.8456091	100.049539	100.074691	99.9819787
99.404965	99.654665	100.037825	100.008175
99.3202442	99.9050103	100.283139	100.074626
99.5021074	100.316177	99.9209532	100.289225
99.3549606	99.990018	100.460604	100.079658
99.0734071	99.8069369	100.433347	100.06397
99.2138653	99.9340452	100.145873	100.024899
99.2965159	99.8337135	100.509628	100.003291
99.0520676	99.7332205	100.027236	100.064118
99.1393364	99.7349949	100.207055	100.020459
99.3798036	100.111803	100.228037	100.262437
99.2000106	99.798549	100.053121	100.064562
99.1515987	99.5944983	100.131951	99.9482349
99.076592	99.7730629	100.025864	100.177042
99.1302592	99.4928762	100.207643	99.8733472
99.1797859	99.6932169	100.461193	99.5425687
98.9882084	99.6528907	100.471978	99.9476429
99.0262691	99.7374145	100.160384	99.9682148
99.1853596	99.9071072	100.084692	99.9819787
98.8319844	99.7888707	100.287257	100.274721
99.4293302	100.099544	100.054493	100.261845
99.7842979	100.295691	100.189406	99.9584468
99.2543148	99.6206297	100.438053	100.092682
98.961773	99.344637	100.385696	99.5971805

98.7552261	99.3365717	100.18117	99.6565282
99.125004	99.6830547	100.002528	100.118878
99.3573493	99.4435169	100.265098	99.931807
99.605142	99.628211	100.050964	100.146702
99.712317	99.6901521	99.8274163	99.919079
99.2227833	99.9172695	100.330202	100.251781
99.2981084	99.7364466	100.17568	100.173342
99.3365274	99.9455658	99.8091914	100.129481
100.12338	100.083936	100.128898	100.235706
99.8816522	99.7894352	100.143564	99.9035322
99.9191974	99.9891597	100.244658	100.061979
100.021928	100.045024	99.9513485	100.195056
99.7527203	99.6854557	99.9990601	99.7777246
100.210133	100.216655	100.031911	100.099216
100.272602	99.8427166	99.9409849	99.8522003
99.9752756	100.01354	100.022134	99.8450792
100.128812	100.175806	100.083729	100.157966
100.321011	99.901972	100.199488	99.8179296
100.201985	100.05003	99.8524056	99.9676229
100.259661	100.02242	99.6441561	100.013762
100.646457	100.567182	99.8821276	100.137344
100.703493	100.212942	99.7299979	99.996256
100.227548	100.224728	99.8936644	99.8790531
100.13648	100.200671	99.4640643	100.244162
100.521998	100.128014	99.4769699	100.038983
100.279632	100.100405	99.8316784	100.056638
100.552514	100.202447	99.6648833	100.381393
100.63751	100.163697	99.6828729	100.027263
100.560821	100.267999	99.74525	99.7069579
100.572644	100.240551	100.003558	100.079485
100.180576	99.9215085	100.145715	99.8343974
99.7741291	100.320473	100.015681	100.263449
100.032312	100.211327	100.207114	100.114497
100.229145	100.102988	100.101327	100.053225
100.282827	99.9050397	100.036213	99.8103634
99.9656896	100.202608	100.197728	100.212117
100.022567	100.092978	100.179348	99.9401766
99.9698436	100.297869	99.9648407	100.30855
100.547721	99.9755972	99.8907313	99.7051776
99.7757267	100.107832	100.227255	100.06005
99.7787623	100.15078	100.404413	100.140905
100.308869	100.45852	100.006491	100.073699
100.054999	100.162244	99.8991395	100.246684
99.9648908	100.229249	100.034844	100.149361
100.230264	100.577031	100.180716	100.03928
100.049567	100.033076	100.549308	99.9112468

99.5729825	100.098306	99.8729372	99.9949207
99.2825262	100.1109	100.162336	100.060347
99.5830478	100.399265	99.884083	100.244904
99.4472459	100.038082	99.7542448	99.9094665
99.459548	100.333228	99.7794694	99.9435889
99.935973	100.269291	100.04599	99.7614052
99.9199963	99.8559562	99.9140005	99.683814
99.8367577	99.4355175	99.9255373	99.7933022
99.5530116	99.1862252	100.443717	99.3827956
99.565793	99.6207108	100.249155	99.8244574
99.7551168	99.3210433	100.048727	99.7639273
99.7624661	99.9176335	99.9963226	100.137344
99.8308463	100.24927	99.874306	100.275317
100.202304	100.55039	99.8007832	100.261075
99.8473023	100.202931	99.9581924	100.016729
99.8222189	100.032754	100.110713	100.097436
99.900984	99.8086488	100.347902	99.8142207
100.096059	100.423968	99.8647246	100.071028
100.036786	99.8759771	99.800001	99.7855876
99.9158423	100.237968	99.8715684	100.021774
99.4729684	100.075056	99.9867412	99.9907667
100.049727	100.508573	99.7671504	99.9226704
99.9738377	100.338879	100.126161	99.8566511
99.8671134	99.9022949	99.8146665	99.916291
100.055958	100.259603	99.534654	100.100552
99.8784569	100.068436	99.7284336	100.086161
99.6038175	100.113483	99.9229953	100.267009
100.115551	100.141738	99.921431	99.9668811
99.8835694	100.050837	99.8570985	100.180962
100.035508	99.8869563	100.153927	99.7798016
99.9131263	100.078769	100.015681	99.9993715
99.9425234	100.201639	99.7630441	100.290747
99.6661266	100.127207	99.8044984	100.03023
99.6701208	99.8272165	100.015681	100.087941
99.8087985	99.7855602	99.8375446	100.015097
100.009146	99.5656533	100.007664	100.001597
99.8466633	100.109285	100.106998	100.037945
99.6983995	99.6991797	99.8838874	100.19194
99.8311659	99.8251176	100.177588	100.126366
99.900984	99.7122579	99.93375	100.262707
100.094941	100.220207	100.001798	100.043582
100.056917	99.8772688	100.172699	99.9613918
100.145108	100.11203	100.175241	100.084232
99.7244416	99.6986954	100.140631	100.125921
99.792023	99.6129607	99.9259284	100.057231
99.8310061	99.8083259	100.110713	100.137641

100.179937	99.4800802	99.9572147	99.942402
100.265572	99.7103204	100.143759	99.998333
100.180576	99.6791588	100.317789	99.9524903
100.099415	99.2509702	100.417906	99.677583
100.221317	99.778456	100.15119	99.8927021
99.8041653	99.8577322	99.8316784	99.782027
99.9092919	99.4203404	100.081773	99.7851425
99.7476078	99.4020956	100.245831	100.065242
100.142072	99.5249657	100.106607	99.6669012
100.115231	99.7621487	99.9190845	100.039873
99.8714271	99.8691958	100.067108	100.079188
99.7223646	99.5934242	100.124596	100.0826
99.9148837	99.9812482	100.392877	99.9993715
100.254868	99.8336749	100.460533	99.5065262
99.0886666	100.332673	100.615422	100.25873
99.2878601	99.4519576	100.564534	99.6895869
99.8045231	99.8399401	100.239242	99.5774265
99.5428532	99.801288	99.8736308	99.9913624
100.1859	100.068117	100.20499	99.8139613
99.5655863	99.7853724	100.268209	99.9451875
99.7285339	99.7718929	100.401301	100.199447
99.6205911	99.4173657	100.527542	100.099054
99.2009017	99.4154168	100.553769	100.03426
99.4595512	99.4548809	100.379184	99.894544
99.567494	99.8360424	100.270362	100.057646
99.4824433	100.249522	100.006723	100.166529
99.3223573	100.194467	99.9683608	99.9177804
98.9581495	100.034987	99.9820615	100.155209
99.3609878	99.715214	100.071116	99.8811384
100.294638	100.222726	100.100865	99.8007046
100.208792	100.598528	99.5894407	100.252623
100.111183	100.164747	100.17387	100.043942
99.7522209	99.7550029	100.622859	99.9782547
99.7919642	99.4452991	100.481156	99.582044
99.0214209	99.4414014	100.097734	99.6757344
100.007691	99.4103823	100.719155	99.3517651
99.7067546	100.096213	100.148426	99.7053757
99.5647915	99.6671425	100.160561	99.7181855
99.2218862	99.9485881	100.091275	100.173083
99.7663696	99.7182997	100.077574	99.9591889
100.479365	99.705145	100.11574	99.7134191
99.8108821	99.8440001	100.112413	99.7086527
98.806648	99.5325099	99.9372408	99.9429532
100.039009	100.056099	100.409129	100.066881
99.9545939	100.14331	100.202054	99.8783083
100.10848	99.9258516	99.7240983	99.9422084

99.8771739	99.9468017	100.211058	99.7721059
100.032014	99.8673863	100.149405	99.5203781
99.3579673	99.5869151	100.177002	99.8586467
99.008544	99.7077435	100.114566	100.280924
100.576338	100.003318	99.8117823	99.9630616
100.435647	99.8961317	99.7988645	99.8382404
100.436919	99.7395746	99.6700787	99.7287611
100.576497	99.6323881	99.9994808	99.6395392
99.7870361	99.5182184	100.089709	99.6096
99.1485995	99.5224408	99.9260846	99.796534
99.5401506	99.1177309	100.282497	99.6547323
100.566959	99.3340526	99.9904776	99.477927
99.9173941	99.8555308	99.9112097	99.7293569
99.543966	99.5437157	99.9249103	100.017578
100.067942	99.6218319	100.39582	99.888288
100.458698	99.6167974	100.253334	99.7798514
100.450114	99.7907317	99.724294	99.771957
100.15331	99.8003136	100.16976	99.7299527
100.144249	99.3369758	100.478807	99.4372632
100.167459	99.5461518	99.9237359	99.3967485
100.378417	99.888986	99.9256932	99.7762766
100.454406	100.141524	99.705896	99.6915233
99.8375896	100.147857	99.8761752	99.6803519
99.9876603	100.207784	100.214776	100.039325
100.380324	100.163935	100.012594	100.001491
100.622123	99.9679142	99.6604882	100.090713
100.635317	99.5731108	99.795733	99.4740542
100.765516	100.028328	100.172892	99.8856069
100.465216	99.8452994	99.8405536	100.330823
100.532462	99.9934115	99.7156822	100.173381
100.322299	99.9531354	100.167998	100.071647
100.279694	99.845137	100.066614	99.9165888
100.412278	99.9615804	99.8313546	99.9033321
99.7293288	99.3819617	100.247854	99.6688826
99.6577908	99.335027	99.9679694	99.6265804
99.6137552	99.06024	99.9998723	99.710589
100.559646	100.217853	100.02375	100.036942
100.692707	99.8733952	100.152341	100.034856
100.441847	99.9450152	99.8489697	99.8662432
100.6226	100.309124	99.9164942	100.301777
100.220556	99.9874026	99.9092524	99.9980652
100.042824	100.468767	99.5980526	100.193787
100.256166	100.364829	99.783011	100.148059
99.6156629	99.9234155	99.8724565	99.8753293
99.6649446	99.8961317	100.036473	100.023685
99.9159634	99.9461521	99.6389587	99.8231963

100.090357	100.350375	99.7616771	100.101289
100.217059	100.325365	99.9386109	100.137037
100.025973	100.358657	99.8851785	100.204214
100.169049	100.605673	99.7419091	100.541887
100.236453	100.602263	99.9675779	100.696498
100.008963	100.216229	100.155276	100.262156
100.238202	100.602263	99.8857656	100.523715
99.8786047	100.344528	99.8971176	100.103374
99.9439427	100.452689	100.002025	100.453559
100.11627	100.558252	99.5888536	99.9930009
100.186218	100.523497	99.7775307	100.088926
100.112136	100.68785	99.6501149	100.527289
100.213879	100.508394	99.7240983	100.567655
100.215787	100.467305	99.9734496	100.34676
99.9458504	100.406079	99.9464398	100.522374
100.020409	100.671122	99.9219744	100.539503
100.24313	100.458373	99.9264761	100.315927
99.9790758	100.643351	99.9468313	100.545759
100.452339	100.831415	99.642286	100.395765
100.098624	100.966859	99.6240837	100.404256
99.92423	100.72975	99.6101874	100.408426
99.9925885	100.813713	99.6763418	100.561399
99.9890911	100.698893	99.8474039	100.625746
100.607119	99.8239819	100.267582	99.6427226
100.116426	99.9586045	99.9947029	99.561286
100.078202	99.6004696	100.540461	99.2710112
100.153056	99.5758318	99.8200368	99.5392641
99.8404205	99.9439506	100.216221	99.4737897
100.000163	99.7796981	99.8608118	99.7068668
100.426673	100.291779	99.7418193	99.5676413
100.2561	100.180184	99.8029818	99.9826574
100.39482	100.20917	99.6524278	99.9396482
100.516338	100.419478	99.8263099	100.011921
100.370452	99.5201147	99.6888901	100.032761
100.004144	99.1297735	100.818633	99.5209372
100.026123	99.4565071	100.587117	99.6753859
100.29958	99.6333201	99.7355462	99.9554626
100.331592	99.7375078	100.000976	99.8768342
100.141589	99.223494	100.02156	99.9201389
99.7905708	98.6706716	100.331489	99.2736716
100.146208	99.4199529	100.257388	99.6697695
100.146049	99.4571513	100.144669	99.7021372
99.9460128	99.4096469	100.542421	99.7906681
100.144297	99.8766393	99.6485072	99.9160006
100.501049	100.476644	99.8811993	100.368705
100.112603	100.081955	100.127222	100.151886

99.3967101	99.1986951	100.066059	99.5771004
99.5711045	99.2262316	100.55634	99.3440233
100.092377	99.9447558	99.8835517	100.145235
100.061798	99.5001468	100.277384	99.7971712
100.519842	99.7958013	100.255428	99.8582117
100.436228	99.6557036	99.8753183	99.6384364
100.418232	100.052003	100.455971	99.9944812
100.40995	99.9922602	100.058806	100.008522
99.8682918	99.7721296	100.248567	100.096018
99.2154673	98.9184996	100.123889	99.1814458
100.422532	100.127849	99.9188378	99.4492553
100.148119	99.9131935	100.153098	99.9196955
99.9891734	99.6840452	99.8439529	99.7121875
99.2887289	98.8890308	100.169369	99.2627346
100.263745	100.170522	99.7296652	100.004975
99.9044448	99.5586013	99.9690225	99.8821549
99.9190971	99.5545756	100.338154	99.7587438
100.099066	100.083887	99.7796538	100.31269
100.12407	99.7695531	100.006857	100.176864
100.232689	100.164886	100.056454	100.097201
100.004144	99.6357356	99.9978395	99.8267307
100.061957	100.04057	100.02646	99.9059503
99.8778476	99.6772818	99.6814408	99.9860567
99.8996669	99.8677826	99.9305999	100.248841
100.42062	100.208848	99.5981265	99.9298936
100.38399	100.665373	99.4438478	100.321114
100.21931	100.035739	99.838856	100.006896
99.9167081	99.9325174	100.138396	99.6353327
100.118815	99.8552221	100.117616	99.8760952
100.207047	99.9080405	99.9827449	99.9867957
100.139678	100.027848	99.6000868	99.9869435
100.006692	99.834932	100.071352	99.9028466
100.17599	100.301441	99.9013908	100.023302
100.023256	100.315129	99.7716164	100.196225
99.6716003	99.4901628	99.9068798	99.8287999
98.9418512	98.5014271	100.415392	99.6583891
99.5908533	99.6929019	100.303652	100.195634
99.9488795	100.178574	99.6565445	100.5771
99.9355013	99.991294	99.9225625	100.33412
99.8703622	100.210458	100.133691	100.382894
100.350544	100.118187	99.983529	100.336042
100.051446	100.562957	100.032733	100.355108
99.9734062	100.268752	99.9948989	100.29791
100.000959	100.029781	99.9213863	100.019755
99.8651065	100.18888	99.9339324	100.124395
99.9554094	100.187753	99.7218238	100.183219

100.209755	100.420283	100.170153	100.202433
99.3298191	99.8879115	99.9053115	99.4328498
100.021982	100.504019	99.9427539	100.199033
99.8064972	100.503375	99.9098203	99.9810316
100.004622	100.42608	100.029205	100.118483
99.8568248	100.244275	100.161724	100.078726
99.4362077	99.9652068	99.9249149	99.5533049
100.38399	100.532039	99.7555416	100.192235
99.9944291	100.506113	99.9570644	99.9564972
100.244156	100.528013	100.079782	100.146269
100.111011	100.353294	99.9927426	99.7519451
99.6779709	100.279863	100.188776	99.9526544
99.7058422	100.114322	100.085271	99.9217647
99.8963223	100.244275	100.159763	100.076213
99.8079306	100.00466	100.149178	100.072962
99.6246174	100.125434	99.7667156	99.9136358
99.8794403	100.432038	99.7884753	100.442752
99.790093	100.334614	99.7639711	100.456054
99.0219611	99.657958	100.136239	99.7055366
99.9769101	100.539447	99.7678918	100.27042
99.8940926	100.637837	100.153686	100.552122
100.154171	100.528496	99.8025898	100.241303
100.004463	100.457642	100.067235	100.17243
100.038545	100.483891	100.12781	100.451472
100.08298	100.266497	99.9525556	100.288451
99.7652478	100.374711	100.243078	100.189131
100.057339	100.385178	100.002544	100.396787
100.026123	100.633006	99.7843586	100.287564
99.878644	100.584696	99.8806112	100.340032
99.942509	100.736066	99.8729659	100.41334
99.8279979	100.414808	99.8743381	100.542811
100.370277	100.030595	100.099093	100.097886
100.431535	100.001165	100.225193	100.156241
100.640333	100.002896	100.2087	100.138108
100.519565	100.005493	100.33669	99.9000512
100.644116	100.062189	100.047725	100.131563
100.415238	100.026844	100.215744	99.9224116
100.471985	100.056562	99.9188764	100.078116
100.233794	99.8936879	100.035012	99.9177759
100.297816	99.8957076	99.938805	99.8832809
100.319351	99.9620692	99.9329639	99.7962934
100.380899	100.096379	99.824559	100.093387
100.21677	99.9626463	99.9946395	100.025488
100.26857	99.8695958	99.9199072	100.013626
100.288504	100.042857	99.9942959	100.019761
100.435172	100.068536	100.091018	100.143425

100.306692	100.041703	100.046694	100.20778
100.570346	100.20703	99.9408666	100.24132
100.438664	100.241221	99.8766139	100.103204
100.291851	100.175003	99.9016965	100.026715
100.311785	100.193469	99.9721339	99.9498167
100.240197	99.9252818	100.145307	99.9390455
100.421204	100.180341	100.018176	100.166876
100.414802	100.080366	100.039307	100.087933
100.418585	100.182649	99.914925	100.253727
100.258094	100.099842	100.069715	100.141653
100.606867	100.485893	99.8133921	100.324899
100.41	100.156393	100.070918	100.070208
100.332883	100.085848	99.7883095	100.206416
100.792822	100.479978	99.9432718	100.379028
100.705228	100.482864	100.0558	100.216233
100.585624	100.510707	99.8295411	100.056847
100.723998	100.776586	100.092565	100.339351
100.58184	100.579376	100.164376	100.118747
100.298253	100.433237	100.026422	100.160195
100.594208	100.733162	100.024876	100.323536
100.415966	100.566248	99.9296997	99.9725862
100.249945	100.47969	100.053395	100.005581
100.349616	100.573462	99.983129	100.299948
100.14591	100.365865	100.088957	99.9365913
99.741408	100.030306	99.865447	99.6904905
100.166135	100.299792	100.286353	100.114248
100.090909	100.355911	99.9577028	100.054665
99.8189618	100.140091	99.5941772	99.8917342
99.9669396	100.300369	99.6312857	100.138926
99.8586844	100.064353	99.8505006	99.9552705
100.00899	100.244827	100.113868	99.9795397
99.9523892	100.232998	100.13036	100.206689
99.7451911	100.153797	99.9741955	100.057528
99.5033631	99.8639695	99.9649184	99.7980659
99.8508272	100.089743	99.846721	100.076889
99.8528643	100.202414	100.021955	99.9814485
99.7446091	100.103304	99.917502	100.076889
99.8477716	100.083684	99.8113305	100.149424
99.6440657	99.8431954	99.911489	100.036941
99.843261	100.087579	99.8810807	100.248546
99.7681808	99.9842859	100.062844	99.9935831
99.7549399	99.9887581	99.8283386	100.062573
99.8420969	99.9617807	100.12933	100.008445
99.6762221	99.9190784	100.133796	99.9014146
99.8295836	99.8573333	100.13225	99.8884619
99.8613035	99.8359822	100.167984	99.8197446

100.035618	99.9726005	99.8242154	100.075935
100.051332	100.195777	100.248901	100.038031
99.7561039	99.8097261	100.476019	99.7706607
99.982363	100.116576	100.342875	100.08357
99.77371	99.9097013	100.382904	99.9078228
99.9916753	100.169377	100.212652	100.278815
99.8653776	99.8903698	100.037074	99.9848571
99.9913843	99.8619498	99.6170264	100.130472
99.9227063	99.8178049	99.8030842	99.9018236
99.7016854	99.6824849	99.7259466	99.7875674
99.9161586	99.9659644	99.7731912	100.041304
99.688299	99.6308383	99.79982	99.9220025
99.7013943	99.7412005	99.7960404	99.9162761
99.7868053	100.026267	99.9240303	99.9417724
99.7438816	99.8752221	100.053566	99.9503621
99.6311158	99.6827734	100.055113	99.9442266
99.5410487	99.5989558	99.9022119	100.010081
99.6621082	99.6894096	99.9338228	99.8414233
99.7534849	99.6690683	100.272953	99.7679338
99.8396234	99.8394445	99.935369	99.8787815
99.8738169	99.8214115	99.8324617	100.026715
99.7770566	99.6273759	99.8175152	99.9379548
99.6973202	99.6684913	99.8542801	99.9736769
99.8797825	99.7645713	99.9573592	100.022215
99.6773861	99.6520451	99.8008508	99.9383638
99.6775317	99.6817636	99.9917189	99.9892201
99.4569472	99.4748885	100.106309	99.9003238
99.4338121	99.5102333	100.069544	99.7898852
99.5669484	99.5623127	100.115586	99.7960207
99.3498561	99.3226896	99.9339946	99.7327571
99.4728072	99.444593	99.8188896	99.7990203
99.570004	99.3483687	100.409189	99.5964131
99.9649025	99.7977521	100.415718	99.7226676
99.7184183	99.7915488	100.137748	99.7297575
99.7510113	99.749135	100.223132	99.8854624
99.758432	99.6703667	100.503679	99.7769325
99.793935	99.9381213	100.328444	99.8969152
99.8528643	99.857189	100.162143	99.8204263
99.6443567	99.6438221	99.7510292	99.8606478
99.7732158	99.962514	99.7215514	99.9761728
99.7634696	99.7752969	99.6914923	99.7934245
99.8280563	99.8033217	99.835948	99.9730314
99.765797	99.8836402	100.136883	99.7266353
99.7260849	100.008018	99.8287338	99.9663388
99.7953266	100.021597	99.9759379	99.797522
99.6094213	99.8294685	99.997237	99.90993

99.7292851	99.9477793	100.134822	100.213828
99.6409874	99.8273017	100.304699	99.9474904
99.7983813	99.8969303	100.134822	99.878789
99.7803436	99.9307333	100.153029	99.6771922
99.9086444	100.24984	99.90225	100.118493
99.9124265	100.229327	99.8836992	99.96989
99.7928536	99.9963171	99.9671778	99.9842312
99.7291396	99.9629474	100.015444	99.8490139
99.6532065	100.044855	100.120565	99.8313946
99.7141567	99.8938966	100.223969	99.8944961
99.7817982	100.077213	100.16574	99.9428465
100.066038	100.242328	100.497937	99.9769923
100.001161	100.19668	99.6205526	99.9641535
100.247289	100.208525	99.5649002	100.011958
100.159864	100.092959	99.7220667	99.9331491
100.277691	99.8982304	99.6811863	100.034767
100.11957	99.6002143	99.8316539	99.9810898
100.124516	99.5300079	99.6028607	100.147448
100.352751	99.7947987	99.8201455	100.164521
100.101241	99.6143711	100.038461	100.039547
100.2758	99.7404826	99.8277032	99.9595097
100.166555	99.6782214	99.830795	99.9894214
100.454432	99.7344154	100.160931	100.043918
100.442213	100.09816	100.19683	100.116854
100.180811	100.127051	100.297657	100.014416
99.8689322	100.253307	100.272751	100.26409
99.7513959	99.9242327	100.255059	100.060308
100.081167	100.316146	99.8077783	100.136522
100.064875	100.135141	99.7979876	99.9987091
100.240597	100.257352	99.4470683	100.076425
99.8378026	99.8860959	99.8419599	99.8626722
99.7887806	99.9434456	99.7938652	100.036816
99.8300929	99.8008659	100.049282	99.9753533
99.7944538	99.9688702	99.9343704	100.301377
99.6972826	99.8742504	99.8519223	99.9901043
99.8252925	99.9597693	99.7571071	100.200852
99.8756237	99.908198	99.9946605	100.083937
99.9812318	100.048322	99.6853087	100.175857
100.078112	99.9103648	99.8855886	100.102785
100.059492	100.061467	99.5647285	100.129692
99.9885051	99.9775376	99.9659754	99.9326028
99.9810863	100.055256	100.225687	99.8767403
99.9438471	99.9294332	100.401576	99.7939708
100.065311	99.9641031	100.262616	99.9158031
99.9842866	99.9957393	100.214865	99.9558219
100.152009	100.14482	100.264334	99.9625145

100.051201	100.01293	100.297142	99.9670218
100.048146	100.084725	100.26004	99.9799972
99.9146085	100.161432	100.050656	99.8955888
100.145463	100.096715	100.302123	100.031352
100.011489	99.9025641	100.40003	99.9267297
100.222996	100.064501	100.201983	99.8797451
100.050764	99.9838938	100.23668	99.9227688
100.095131	100.045722	100.165912	99.8536577
99.9870504	100.021019	100.131215	99.9092471
100.008579	100.050778	99.9288738	100.071371
99.9879232	100.00614	100.020941	99.9287785
99.971631	100.081403	99.9451917	99.963334
99.7579419	99.9266885	99.9792015	99.8912181
100.072002	99.8875405	99.9183961	99.976446
99.8353296	100.004985	99.8591365	100.107702
99.8795512	99.8732392	100.025063	99.9768558
99.787035	99.9375229	99.8890239	100.043372
99.8034726	99.9263996	99.8756261	100.201399
99.7621604	99.9958837	99.8627436	100.142121
99.9471928	99.8979415	99.8675531	100.119995
99.7355401	99.9058866	99.8495176	100.045557
100.057165	100.059878	99.7244715	100.022475
99.9646487	100.025931	99.8251267	100.011002
100.131934	100.092959	100.072643	99.9515879
99.948502	99.9997841	100.04327	99.9078812
99.9578118	100.063923	100.046019	99.9565049
99.7132839	100.02362	99.9445046	99.9317833
99.9298824	100.029398	99.9446764	100.170667
100.00087	100.173567	99.915476	100.082571
100.063565	100.190613	100.154232	99.9055593
100.092513	100.292744	99.845567	99.9892848
100.11957	100.104371	100.003077	99.9907872
100.198703	100.243773	99.611449	100.05662
100.275945	99.9963171	99.889711	99.916486
100.32904	100.179923	99.866866	100.107156
100.198703	99.9698814	100.125718	100.014689
100.242634	100.120695	99.9974087	100.118629
100.20874	100.091659	100.249562	100.007041
100.221832	100.176167	100.187554	99.9612853
100.096586	100.01163	100.253685	99.9175786
100.159573	99.9476349	100.098408	100.008953
99.9770133	99.9243772	99.933168	100.034221
99.9871959	99.761429	100.099438	99.7669274
100.017162	99.8583601	100.001531	99.9524074
99.9930145	99.885807	100.055809	99.8822036
100.128152	100.149876	100.071955	100.073147

99.8889439	99.6875651	100.010154	99.4762729
99.8947512	99.8623908	99.7019266	99.6976213
99.8925734	99.8763711	100.202883	99.6156202
99.9329344	99.864841	99.9082127	99.7190069
99.7855733	99.8280887	99.8898495	99.6064939
99.9319182	99.9654414	99.616117	99.7699511
100.050243	99.8554727	99.8903643	99.9024877
100.141999	99.9609735	99.9171369	99.9030326
99.9928952	99.8197293	100.234117	99.7650474
99.9367092	99.6903035	99.8831563	99.7605523
99.699044	99.7142285	99.9859561	99.6372783
99.8284024	99.5872529	100.133205	99.6696973
99.8394363	99.725038	99.6959199	99.8911819
99.8272409	99.4808873	100.089786	99.7548313
100.080005	99.7310914	100.11776	99.6942159
100.28602	99.645336	100.012214	99.6236569
100.136627	99.4533591	100.173707	99.7549675
99.8660049	99.6554248	100.164097	99.9874855
99.9719888	99.4140125	100.028003	99.5149578
100.314912	100.42535	101.04742	100.27122
101.266154	100.317111	101.157428	100.306227
100.091039	99.1436308	100.211464	100.198209
100.347143	99.6513893	100.324561	100.265908
100.045742	99.6751702	99.8102182	99.8012804
100.520927	100.105244	99.500789	99.9373586
100.252628	100.003635	99.9068397	99.8409188
100.382277	100.201809	99.5433506	100.137866
100.055324	99.9681799	99.7673134	99.8663909
99.9307567	99.8922251	99.8100466	100.025081
99.9407743	100.049611	99.7582176	99.876607
100.082473	99.9719271	99.9643321	99.846776
100.184828	99.9302745	99.9104438	100.025489
99.7614728	99.7949395	100.019422	99.9542492
99.7320006	100.08694	99.835103	100.041563
99.6819123	99.9358955	100.075713	100.055048
99.9397581	99.905773	100.049627	99.9921168
99.7079002	99.8118023	99.9183382	99.9361327
99.9124636	100.163183	100.036412	100.122202
99.8337742	99.946705	100.055805	99.935724
99.8586005	100.189847	100.000372	100.112803
99.8358067	100.027992	99.8510636	100.006692
99.7219829	100.078149	99.8726876	99.9648739
99.9176902	99.9396428	99.7086197	99.930003
99.816207	99.8649851	99.8701133	99.9539767
100.020335	100.004212	99.8979156	100.124381
99.8619397	100.050188	99.9852697	99.928096

99.8349356	100.042982	100.023712	100.220821
99.9581963	99.9454078	100.012042	100.099045
99.7960265	99.8897749	99.9591836	100.101361
99.956309	100.038225	100.023026	100.195621
99.9095599	100.108559	100.174565	100.167697
99.8050279	100.04154	100.036412	100.102042
99.9380159	100.094002	100.083607	99.8894111
100.093652	100.233805	99.7789835	99.9680068
99.9686496	100.091985	100.035211	100.06431
100.009156	100.088814	99.7324748	100.175325
99.8188203	99.9781246	99.7396828	99.8925441
100.166825	100.212907	99.7709174	100.167152
99.9587771	100.031596	99.8658228	100.019632
99.9457106	100.10928	99.7995778	100.026851
99.8816847	100.092849	99.9797779	99.9103881
99.854245	100.097606	99.7856767	100.070849
100.016996	100.153239	99.8852157	100.091553
99.9608096	100.138105	100.095106	100.069487
100.106864	100.079157	99.6645136	100.008735
100.162469	100.142285	99.92383	100.066081
100.188312	100.147762	99.5191523	100.159252
100.189909	100.377356	99.9250314	100.175598
100.340174	100.471615	100.013072	100.165926
100.249434	100.299095	100.163239	100.182817
100.118769	100.187685	99.9131897	100.022493
100.04429	100.144591	100.131489	100.139501
100.374873	100.308463	100.002088	100.131328
100.020335	100.021651	99.9133613	100.110896
100.058083	100.03664	99.8634201	100.061314
99.8079315	100.085787	100.182975	99.9531595
100.097282	100.179614	100.14745	100.122883
100.01656	100.085499	100.300191	100.05532
100.282681	100.467002	99.8275517	100.062812
99.9908626	100.080743	100.03092	100.065809
100.163776	100.170678	100.119304	100.195485
99.9448395	99.8746416	100.411057	99.9530232
100.00088	100.084634	100.322158	100.145086
99.8275313	99.8933781	100.21747	99.9429434
99.9178354	100.016751	99.9298367	100.356082
99.9724243	99.9859074	100.077086	99.9775418
99.9978314	100.169381	100.021653	100.114574
99.8795069	100.040964	99.9895601	99.8541316
100.014382	100.234094	99.7551285	100.152986
99.8323223	99.9366161	100.056492	99.9531595
99.9043333	100.067051	100.007409	99.8879128
99.882701	100.081608	99.8980872	99.9332722

99.8276765	100.104956	100.081548	100.036795
99.9841842	100.077284	99.9437378	99.9170626
99.9309019	99.992249	100.195503	100.014592
99.9827323	100.07296	100.055977	99.8850523
99.8025597	99.9134117	100.32765	99.8836901
99.9789576	99.9497316	99.9555796	99.8757897
99.8408882	100.066763	100.02766	99.9336808
99.9849101	99.9337336	99.9318961	100.006011
99.7331621	99.9605411	100.158433	99.9485282
100.023882	100.109335	100.046201	100.234673
99.9300604	100.143551	100.249666	100.327866
100.136758	100.168239	99.9939598	100.546451
100.056464	100.126227	100.296064	100.392541
100.407749	100.354336	99.9657772	100.432792
100.123376	100.226855	100.326653	100.298803
100.318582	100.44789	100.107034	100.492146
100.150431	100.389563	100.218906	100.334415
100.307382	100.328205	100.02747	100.311629
100.420404	100.172715	100.27802	100.098637
100.883109	100.4733	99.987086	100.233445
100.543026	100.117564	100.092427	99.928353
100.674521	99.9681381	99.8740117	100.210113
100.262726	99.8187119	100.266335	100.346013
100.285563	100.161165	100.070087	100.516706
100.16265	99.9987452	100.313764	100.330458
100.289199	99.7948903	100.012175	100.255004
100.200178	99.915875	100.076102	100.329776
99.9575522	99.703069	99.9355324	100.047197
100.191596	99.9443165	99.8145531	100.410961
99.8439485	99.8100495	100.044482	100.201244
100.213851	100.014482	100.136248	100.420649
99.9363152	99.9054801	100.51637	100.145574
100.238143	99.8123595	100.222342	100.095908
100.059956	99.7886823	100.247432	100.110235
100.147813	99.653549	99.7645461	100.081172
99.8350755	99.645031	100.102394	99.9983496
99.9891169	99.6565809	99.7819025	99.9287624
99.9767528	99.9200618	100.02936	100.16495
100.079302	99.9409959	99.9102711	100.064935
100.246289	100.012172	100.032969	100.266602
99.97268	99.8623126	100.203783	100.171226
100.342583	100.09331	100.031938	100.193467
100.149413	99.9945584	99.9183478	99.9503208
100.155086	100.035849	99.5880608	100.100274
100.06621	100.305249	99.9872578	100.261962
100.249925	100.278973	99.8343154	100.185144

100.193487	100.130847	99.9851957	100.202336
100.110866	100.044656	100.264273	100.059204
100.228252	100.208519	100.150683	100.178185
99.7501273	99.9470596	100.274412	99.8361158
100.069265	99.8753062	99.9833054	100.026867
99.8022017	99.8380579	99.9496237	99.8252002
100.0153	99.8045633	99.8704029	99.8965612
99.8275116	99.7811749	100.059605	99.8324318
100.095302	99.9668387	99.6629854	100.042831
99.8151476	99.9246818	99.9429217	99.9119796
100.017481	100.030219	99.5870297	100.028504
99.9505702	100.110057	99.6770768	100.10505
100.09312	100.070066	99.6141813	99.9957571
99.8388575	99.7758331	99.9585596	99.8200153
99.8131112	99.9106776	99.9100992	99.8187872
99.6768159	99.8911872	100.192441	99.9593262
99.997699	100.046388	99.9226439	99.96724
99.9608978	99.8530727	99.9712762	99.9402238
99.7574003	99.8692425	100.063042	99.8853727
100.0345	99.9752124	99.8327688	100.03096
99.8212569	99.8383466	99.8757301	99.9224859
100.073338	100.11049	99.9595907	100.108461
99.896023	100.143696	100.016472	100.016497
100.330364	100.180078	99.9860549	100.120059
99.9757346	100.16463	100.353632	99.8669525
100.082793	100.119297	100.03211	99.9164823
99.7704916	99.9583207	100.164259	99.8898754
99.8907864	100.000766	99.7794967	99.8531715
99.7352905	99.9857516	100.147933	99.954687
99.7883831	99.8937859	99.8021803	99.8812793
99.6403056	99.8533614	99.9583878	99.9604177
99.6669247	100.002643	99.9384537	99.9636924
100.102575	100.435185	100.005817	100.065617
99.7873649	100.202311	100.153261	99.7492
99.8899137	100.140231	100.121985	99.8966977
99.6859798	99.9294461	100.237809	99.6051135
100.265489	100.069055	99.9589033	99.9156636
99.8881682	99.8448434	100.310327	99.6655589
99.9880986	99.9422953	99.9917258	99.7352826
99.9107143	99.9925371	100.210657	99.7887692
100.151449	99.971603	99.705775	99.9317642
99.9318059	100.084647	99.9716199	99.8624498
100.0425	100.147305	99.8549368	99.9287624
99.8040927	99.9607751	99.934845	99.7882234
99.8061291	99.9915265	99.8432513	99.8560369
99.8731859	100.003509	100.102051	99.9092506

99.9575522	99.9233824	99.645629	99.8284749
99.885259	100.039892	99.7695296	99.8376167
99.8785678	100.017225	99.7889482	99.8979257
99.9480974	100.141097	99.8023521	99.8743206
99.8161658	100.053607	100.268741	99.9282166
99.8064201	100.056206	100.241589	99.9602813
99.6101955	99.9454715	100.33387	99.7970924
99.7710735	99.9184737	99.9886326	99.816877
99.6209595	99.889166	100.050841	99.7758069
99.845985	99.9051914	99.6909962	99.6987152
99.7322358	99.9173187	100.152573	99.6706074
99.7520183	99.8626013	99.845829	99.7010347
99.7147807	99.8962403	99.923675	99.9291717
99.5831401	99.8660663	100.044654	99.8250637
100.080902	100.170838	99.8879312	99.9838864
99.9290422	100.19798	99.9580441	99.8919221
100.071156	100.215305	99.8908525	99.8819615
99.6612251	99.6332679	100.467302	99.8080598
99.7281353	99.7284379	100.088219	99.8866546
99.8141625	100.052709	99.5486167	99.8539546
100.106266	100.158105	99.6336801	100.193002
99.9734183	99.9214169	99.6863193	99.9585086
99.6242869	99.8356485	99.9457127	99.889523
99.8757263	99.7800639	101.178832	99.8442019
99.9641837	100.196536	99.315643	99.9329796
99.8093022	99.9821146	99.4943762	100.024482
99.8452684	99.7134284	99.6755113	99.9236572
100.008412	100.202639	99.2385855	100.13004
100.019591	100.242389	99.6376831	100.078982
99.6199126	99.8470293	100.072607	100.127746
99.4080036	99.6999034	100.436679	100.263278
99.4391095	99.6472877	101.445031	99.9397204
99.6568509	99.9426941	100.983887	100.059047
99.7605372	99.8598945	100.910232	100.20591
99.4784779	99.7175519	100.767926	100.066648
99.544902	99.5992904	98.9631802	99.6898808
99.5905888	99.730747	99.8274245	99.7164137
99.6968673	99.8387823	99.6370826	99.7039361
99.5095838	99.635742	100.154268	99.9037217
99.3712274	99.7820432	100.741706	99.8849335
99.4971091	99.6563594	99.8460384	99.8364572
99.4702154	99.5489839	99.6773126	99.6243373
99.9265974	100.282469	99.5037832	100.039828
99.8336037	100.035555	100.142059	100.281063
99.4977571	99.5999501	101.716833	99.9943639
99.5892927	99.6319484	101.089966	100.184397

99.7547049	99.7873212	100.487317	100.196301
100.42413	100.238925	99.2061614	99.9976625
100.795619	100.766566	99.3768886	100.18999
100.437253	100.332941	99.0546484	99.6191742
100.368885	100.390999	99.9012796	99.8004585
100.45718	100.339868	100.850988	99.7813835
100.365806	100.193237	99.7573723	99.7268834
100.208171	100.195216	99.5400103	99.7565716
100.15811	100.012299	100.091621	99.877619
99.8026598	99.8237728	100.1965	99.9597994
99.8754023	99.9544048	101.450835	99.963098
100.003228	100.027968	100.31759	99.9646757
99.9907534	100.073656	99.1509202	99.8473572
100.356896	100.350589	99.4923747	100.191281
100.212869	100.210061	100.14266	99.7382137
100.403717	100.464562	101.811704	99.6921755
100.283667	100.288902	100.077411	99.7029321
100.277511	100.322055	99.1737372	99.7554242
100.471437	100.221277	99.6338802	100.077692
100.517448	100.327333	99.3860955	99.9781573
100.369533	100.352898	99.8898711	100.005264
99.9555972	99.942859	100.77353	99.9956546
100.006468	100.166682	100.554767	100.13549
100.117445	99.9172934	99.2509948	100.050442
100.225992	100.110767	99.3955025	100.076544
100.151791	100.299458	99.4891724	100.001822
99.9627256	100.114066	100.071006	99.960373
99.5487903	99.6352471	101.338551	99.8529506
99.8013637	99.8471942	100.47891	99.8657151
99.7963414	99.8115673	99.1559239	100.171776
99.9078043	99.8387823	99.2696087	100.207344
100.074837	100.063265	99.4885719	100.443129
99.919955	99.9172934	100.118241	99.8003151
99.9802227	100.066234	101.730043	99.7389308
99.9960997	100.072336	99.9258979	99.8330151
100.118903	99.9280145	98.7083903	100.022618
99.983949	99.8783678	99.6162671	100.082855
100.172205	100.054193	99.4985794	100.138789
100.422672	100.253935	100.111036	100.21724
99.8410562	99.5908785	100.245136	99.952198
99.8757263	99.7592815	99.0400375	99.9547796
99.7926152	99.5526125	99.659099	99.6888768
99.9552732	99.7373446	100.014964	99.9002796
100.205417	100.17097	100.85459	99.9879099
99.9983678	99.7978773	100.286767	99.8466401
100.164914	99.9468176	99.5320043	99.9236572

100.284963	100.253935	99.5199953	100.18368
99.9722842	100.033081	99.4667556	100.041836
99.9257874	99.6286496	101.011707	99.9864757
99.8018498	99.6830796	100.817162	100.006124
100.202014	99.9887122	100.390644	100.157864
100.096222	100.121488	99.3698834	99.8209677
99.8757263	99.8339991	99.5432126	100.411719
100.268276	100.102026	99.7579728	100.246355
99.9265974	99.9829393	99.5936502	100.476976
99.9834629	100.106974	99.7451632	100.334702
100.066412	100.093119	99.6556965	100.617385
100.030446	99.9900317	99.9741339	100.191568
99.9379381	99.8351536	101.259292	99.8529506
100.172529	100.159754	100.97488	99.8814914
99.8721621	100.091304	99.1769396	99.8890927
100.138182	99.978321	99.711338	100.046139
100.099462	99.9370862	99.6010558	100.012865
100.326114	100.120004	99.4435383	99.9352743
100.273785	100.137487	100.37063	100.195871
100.39837	100.275707	100.476108	100.033231
99.9619156	100.031596	99.1725363	100.103364
99.8788045	99.9095413	99.4445391	100.187409
100.152277	100.156785	99.7297517	99.9728507
100.408577	100.254759	99.7473648	100.151123
100.021211	99.972878	99.9919472	100.135203
100.364809	100.138373	100.889992	99.9506214
100.670694	100.403663	100.064588	99.9405928
100.546092	100.303372	98.8876372	99.8978999
100.566289	100.245945	99.4194534	99.764377
100.571013	100.283844	100.014424	99.8109381
100.714183	100.360137	100.328597	100.013515
100.324904	99.991081	98.9939604	100.193312
100.568244	100.066216	100.132938	100.161794
101.022674	100.462745	99.5845342	100.202624
100.914686	100.475157	100.846823	100.258354
100.540555	100.143503	100.367569	99.968816
100.689914	100.159225	98.6420144	99.7947492
100.840088	100.43428	99.6019217	99.7884455
100.504559	99.8692759	100.701328	99.8526282
100.152253	99.7852039	99.7524131	99.8077863
100.475729	100.268949	99.117871	99.7815688
100.86794	100.493031	99.3179266	99.8246915
100.695452	100.440237	99.9324831	100.052339
100.464002	100.216818	100.602799	100.048758
100.653592	100.254551	99.289747	99.8053508
100.456509	100.128277	99.2493761	99.8603645

99.8326854	99.8398176	99.5143849	100.096179
99.192085	99.5194172	99.758009	100.116522
99.3543118	99.6160669	99.4128581	100.236435
99.2505583	99.6326165	99.8135689	100.164516
98.9034646	99.3655058	101.07266	100.210504
99.4588797	99.5732034	100.141332	99.9602201
99.4145768	99.7322451	98.7115642	99.9821396
99.5061144	99.484663	100.038607	100.083285
99.9870941	100.034772	99.6484881	100.158642
100.026022	100.144496	100.015423	100.074402
99.66997	99.9207452	100.013225	99.990449
99.540156	100.047019	99.6938553	100.203197
99.5528605	99.7732882	101.265521	100.200619
99.3508913	99.7186744	100.677745	100.099187
99.5671938	99.7979471	99.2291907	99.7477583
99.9014201	99.9033681	99.4858055	99.7298501
99.6077505	99.5871051	99.5809368	99.8493331
99.590974	99.5369597	100.316006	99.9805637
100.018367	100.300393	100.514463	100.012225
100.051105	100.164024	100.07478	100.082425
99.8515793	99.8600081	99.1928169	99.8109381
99.6452125	99.7898378	99.2641654	100.073256
99.7867537	99.5842916	99.8631332	99.9524838
100.12098	100.005479	99.6462897	99.9910221
99.9737381	100.060589	99.8113705	100.217524
100.079935	100.02418	99.5095883	100.292595
100.055014	100.071843	100.136936	100.075262
99.6033528	99.7511117	101.858693	100.107496
100.173101	100.584384	99.8399499	100.129702
100.379305	100.483597	98.4753347	100.322967
100.154044	100.261502	99.4014663	100.05893
100.223268	100.163362	99.3596965	100.078843
100.30943	100.28947	99.5993236	99.9466099
99.8812232	100.450002	99.8931115	99.8708228
99.5546521	100.020539	99.9106989	99.8378719
99.577618	99.5252095	99.2323884	99.7115122
99.872102	99.9316679	99.9094997	99.8352931
100.200302	100.225589	100.99032	99.8566396
100.058109	99.9569888	99.9260877	99.8755506
99.9533783	99.9708905	99.0199417	99.966094
99.8735679	99.8702689	100.000434	99.8851493
100.119351	100.385293	100.021419	100.032139
100.235809	100.275734	100.82344	100.117238
100.193461	99.9922395	100.876802	99.9364381
99.8149317	99.7711367	100.587411	100.098901
99.6544965	99.6648882	101.096843	100.078414

100.060878	100.308999	101.106036	100.05893
100.024882	100.296421	101.370845	100.009503
99.7352842	99.8557052	101.134016	99.857929
99.880083	99.8967482	101.021497	100.1638
99.810534	99.9856196	101.122624	100.141737
99.8348028	99.9662566	100.917772	100.474111
99.9737381	99.9005546	101.187378	100.233283
99.9611964	99.9702285	101.21136	99.9139455
99.7602046	99.7583935	100.891591	99.6169574
100.464002	100.173458	99.302138	99.6097942
100.256169	100.037089	99.2879483	99.8832869
99.8888785	100.082435	99.5247774	99.8764101
100.26301	100.252896	99.6880595	100.023543
100.176033	100.061086	99.6792659	99.8576424
100.210237	99.990088	99.7761959	100.040019
99.7615076	100.032621	101.091247	99.9606499
99.49455	99.8039049	101.659437	100.096035
99.886761	100.190504	99.686061	99.8335739
100.243953	100.232043	98.853262	100.169244
99.7432652	99.7888448	99.5215797	100.079846
99.8323597	99.7049383	99.4156561	99.8516253
99.9875827	99.9422597	99.990841	100.147038
99.815909	99.7669993	101.163795	100.259214
100.026674	100.015243	100.28383	99.8388747
99.9646169	99.8523953	98.768723	99.8587886
100.137919	99.7502842	99.9358807	99.8610808
100.228643	100.084421	99.0501199	99.7940328
100.175056	100.093358	99.3061351	100.083285
99.9072837	99.8520643	99.7855891	99.8358662
100.064787	100.338457	99.4760125	99.8573559
100.194438	100.177595	100.246656	100.096608
99.9424654	99.9871091	101.18438	100.076265
99.1904716	99.6525257	99.5622038	100.202789
99.3751459	99.5596832	99.6086563	100.245804
99.4230124	99.7597152	99.6342853	100.296131
99.5489855	99.7277232	99.849729	100.534292
99.4666745	99.7760409	100.526294	100.649286
99.4061944	99.6091552	100.978606	100.483391
99.4993401	99.5831	99.802876	100.311043
99.1595847	99.3766366	99.4016222	100.611002
99.1665383	99.3128177	99.9604542	100.517659
99.4225273	99.5410487	99.494327	100.615734
99.7116671	99.5207652	99.6599143	100.619892
99.6785163	99.774062	99.7668352	100.561535
99.5805192	99.8393651	99.9430345	100.476652
99.9563362	99.9295692	101.09754	100.113747

99.1849734	99.8152887	101.854997	99.9679258
98.7118062	99.5977767	102.233024	99.9279217
99.0334499	99.4797034	101.365643	99.8007402
99.7292936	99.800612	99.1080902	99.7225961
99.7474053	99.7880791	99.265268	100.199061
100.169633	100.242232	99.6907492	100.008934
100.737402	100.625806	99.6479007	100.36897
100.701664	100.30836	99.5656077	99.7810967
100.622425	100.428907	99.6965558	99.8740094
100.406217	100.363439	100.750948	99.8258324
100.291725	100.264825	101.29156	99.7531368
99.9270665	100.049951	100.367314	99.6890442
100.408481	100.198862	99.2460462	99.7043863
100.895717	100.714196	99.7103714	99.8031778
100.400072	100.172807	100.176298	99.7148533
100.284125	100.170003	100.564738	99.6867501
100.104302	100.108658	99.7151768	99.7590155
100.339754	100.177424	99.3019094	99.7777988
100.111903	100.06908	100.083593	99.8606747
99.5460747	99.2280556	100.570144	99.7562913
99.6992153	99.5112006	98.99296	99.7334932
99.9493827	99.8797673	99.636688	99.851068
99.9448547	99.9780518	100.719312	99.9822642
99.7938164	99.6831984	100.543313	99.9376718
100.222351	99.975908	99.4188417	100.088081
100.418345	100.478709	100.241372	100.134968
100.55014	100.614262	101.240101	99.9748082
100.172868	99.9696415	100.014716	99.6932024
100.02474	99.7704341	99.6200692	99.926918
99.9227003	99.8304602	100.387737	99.980687
99.9456633	99.7676307	100.321463	100.032018
99.955366	99.7377825	99.3701866	100.074173
100.293019	99.8654204	99.6737299	100.059978
100.107051	99.7862652	100.10682	100.012231
100.318246	100.050446	100.916936	99.8593842
100.098157	99.9816797	99.6629177	100.065857
100.178528	99.9834937	99.3373495	99.9468484
100.109153	100.18534	99.5435827	99.7545706
100.093791	100.06974	100.114829	99.9667787
99.9757416	99.873171	100.693283	100.07446
100.320348	100.004107	100.579955	99.9339438
99.7039049	99.8141344	99.0075765	100.202502
99.9414588	99.8596487	99.6114595	100.137836
99.9265814	99.8913108	99.3357477	100.185152
99.74029	99.7710937	99.8919768	100.072309
99.9927213	100.336559	100.969195	100.062416

99.8798468	99.8103415	100.257991	99.9143002
100.195346	100.143618	99.6895478	100.08292
100.471225	100.584084	99.3635792	100.040908
99.949221	100.3359	100.351697	100.231179
99.6424546	99.8171027	100.308848	99.7789459
100.220734	100.02604	99.0854646	100.067578
99.9729925	100.186659	99.8271034	100.218417
100.037839	100.117893	99.5898351	100.0736
99.9197895	100.251467	101.032667	100.12192
100.008731	99.9646943	100.537507	99.9111458
99.729617	99.9839884	99.2306288	99.9885731
99.9909425	99.9554595	99.4897218	99.9687861
99.8358613	99.9518316	100.2658	100.077328
99.652319	99.7265689	100.20473	100.092096
99.5953966	99.9275903	98.8415888	100.145292
100.198418	100.065782	99.6264765	99.7858283
100.499201	100.513339	100.667454	99.8965206
100.328919	100.228215	99.8667482	100.034456
100.448262	100.473102	99.4436697	100.409548
99.6773843	99.9007105	100.036741	100.268028
99.7786155	99.7979735	100.258191	100.410838
99.7731174	99.8840549	99.465094	100.404099
100.13244	99.864431	99.581826	100.131814
100.031855	100.178414	99.9334237	100.042055
99.9471187	99.9524912	101.317589	99.8046116
99.855105	99.988276	100.392343	99.829417
99.699377	99.6178953	99.2284263	99.8764469
99.7359238	99.7024925	99.637489	99.9005354
99.8502536	99.7552627	99.5658079	99.8949434
100.157505	100.245036	99.4883202	99.9196054
100.535101	100.358492	100.171293	99.8037513
100.130661	100.336724	101.031666	99.9626206
100.137453	100.277193	99.9326227	99.6109
100.090071	100.128612	99.4406663	99.8109205
100.099289	99.917531	99.8048783	99.7896997
100.030562	99.938639	99.5806246	100.004919
100.017463	99.7677956	100.175698	99.7965821
100.16931	100.294178	100.933154	99.6725551
100.086352	100.245201	99.8415197	99.6589336
100.387782	100.379765	99.5856303	100.003628
100.173191	100.254271	99.6312819	100.010654
100.497559	100.403564	100.069949	100.227826
100.309082	100.218886	101.131603	100.316443
100.372937	100.012073	100.020137	100.327914
100.173574	100.062124	99.4129914	99.8451094
100.401533	100.42669	99.720265	99.6912485

100.734293	100.696109	100.278799	99.9403225
100.812283	100.456424	99.8807034	100.088017
100.031729	99.9567352	99.5802315	100.270557
100.14579	100.068566	99.5710293	100.297802
99.6630611	99.9151083	99.7042611	100.356163
99.1616473	99.5956379	99.845695	100.220226
98.958872	99.4471354	100.763315	99.9256964
99.5975816	99.7324122	101.589112	99.9636956
99.348012	99.7023483	100.07475	100.051165
98.9174396	99.5715207	98.9324764	99.9920874
99.1795201	99.5774674	99.3921865	99.9922308
99.3238025	99.4907446	99.2751584	99.9084892
99.2872444	99.5875438	100.32421	99.8638939
99.5228407	99.8574582	101.110998	100.069663
99.6702102	99.8686909	100.57627	100.253063
99.4399758	99.6668332	99.3995882	100.128311
99.6256906	99.6924371	99.5648278	99.9788953
99.6497377	99.6636947	100.113159	99.9624051
99.4526493	99.768588	99.6140395	99.9589636
99.9240042	99.9070141	100.206181	100.142364
100.418594	100.204184	101.090793	99.9946685
100.652403	100.631191	100.739309	100.007717
100.28276	100.466005	99.2725578	100.01331
99.9033692	100.480706	99.3411742	100.098629
100.010281	100.379447	99.4680046	99.8828218
100.160738	100.281987	99.5148158	99.9013196
100.376836	100.217069	99.628643	99.8940065
100.419081	100.540173	99.708062	100.163729
100.537042	100.627392	100.164371	100.106515
100.390322	100.662907	101.191017	100.240014
99.6635485	100.108707	99.9647235	99.86246
99.6815838	99.9312965	99.3805837	99.9859215
100.037578	100.078147	99.4435988	99.9413263
99.9327782	100.174946	99.7408699	100.062924
99.9651117	99.9499626	101.050783	99.807397
99.782809	99.8437478	100.054545	99.8488376
100.319806	100.206332	99.4271948	99.7510435
100.582211	100.650353	99.8502961	99.9833405
100.41242	100.547772	100.212583	99.8109818
100.157163	100.252584	100.774917	99.7689676
100.285685	100.048248	99.369581	99.8968744
100.329067	100.168339	99.4481999	100.169752
100.065199	99.9550834	99.887505	100.099776
100.22508	100.127538	100.720904	100.189683
100.084697	100.172964	99.1181209	99.9301416
99.9482138	99.995554	99.0639079	99.7751335

99.6882455	99.581597	100.736108	99.7004257
99.9946831	99.4496132	101.434875	99.6721772
100.077386	100.029087	99.5358208	99.7942048
99.9532507	99.9362521	100.383024	99.9694313
100.435979	100.05816	99.4085904	100.01331
100.792623	100.09351	100.041142	100.267546
100.104845	100.043293	101.236028	100.127737
99.762174	99.6045579	99.6198409	100.141933
99.761849	99.4499435	99.8028847	100.12573
99.9371651	99.655105	99.7152638	100.035679
100.423306	99.9063534	99.9955308	99.8310569
100.639242	100.243664	100.78392	99.7701148
100.425418	100.078478	100.435636	99.8185817
100.034816	99.8954511	99.595235	99.8008009
99.9210796	99.8245862	99.3675806	99.875939
99.9764853	99.7259699	100.005533	100.099202
100.101758	99.9331136	100.40983	100.059482
99.581496	99.6927675	101.155809	100.016751
99.9769728	99.7167195	100.405029	99.7064482
100.040015	100.056343	98.9344769	99.875222
100.090709	100.323779	99.5350206	99.9920874
100.094446	100.213765	99.6178405	100.107232
100.069424	100.111184	101.422872	100.26898
100.154564	100.245151	100.100356	99.9061949
100.028479	100.126051	99.6782549	99.8532828
100.029129	99.9134564	99.6322439	99.6645774
99.8422767	99.753556	100.168972	99.7950652
99.8980074	99.7941919	100.652688	99.7527642
99.8279784	99.7163891	99.3929866	99.7937747
99.91783	99.6349522	100.362019	99.9245492
100.005082	99.8159965	100.03314	100.137632
99.8081559	99.5668955	100.166772	100.413377
99.9426894	99.8401137	99.5972355	99.8935763
100.001832	99.9374084	99.7064617	100.074108
100.09867	99.9798613	99.5848326	100.144084
100.34889	100.206167	99.8042851	100.228399
100.366763	100.130181	101.992409	100.431874
99.9745356	99.8470515	101.067988	100.130605
100.765002	100.24003	100.046543	99.924836
100.504546	100.227476	99.2497524	99.8922858
100.069261	100.196586	99.7402698	99.8418114
100.100295	100.155289	99.6236418	99.8005141
99.9140929	99.9977014	99.6388455	99.874505
99.8372398	99.8239254	100.20038	99.5828433
99.7316277	99.911309	99.6194408	99.8174345
100.064062	100.11168	99.6178405	100.406064

100.141565	100.275049	99.5896337	100.482779
100.177798	100.322623	99.8705009	100.42112
100.229792	100.378621	100.139365	100.130892
99.8578906	99.8498623	100.114877	100.192314
99.7235557	99.4527389	100.287155	99.9363116
99.7811917	99.6695318	99.8793157	99.9836196
99.8788898	99.77522	99.8898942	100.000237
99.7817874	99.9439248	99.4595753	100.036167
99.648495	99.6770621	99.6411102	99.8970879
99.7094074	99.7316236	99.8262342	100.12734
99.6732174	99.7958292	99.7797643	99.986015
99.6627923	99.7316236	100.130744	99.8452887
99.7243004	99.6548676	100.067273	99.9381082
99.7021098	99.7332089	100.342126	99.8933452
99.6930251	99.6992566	99.6586781	99.8858598
99.8205092	99.6729667	99.9146404	99.8367553
99.7899785	99.6317483	99.8863051	99.9493363
99.7671922	99.8247614	99.6717123	100.083625
99.7601925	99.693708	99.7627631	99.9352637
99.957227	99.8386329	99.8436132	100.043653
99.6306234	99.6811575	99.8097997	99.8186405
99.6958548	100.074186	99.6458328	100.206386
99.7240025	99.8810403	100.048572	100.051438
99.7412784	100.069958	99.7756085	100.107878
99.6021778	99.8268751	100.053672	99.8979862
99.5808808	99.7905448	99.92503	99.9199934
99.4245042	99.7181484	100.201394	99.8620561
99.5692642	99.708108	99.676246	99.9863144
99.499416	99.599117	99.6076746	100.028682
99.5689664	99.6341262	99.5289024	99.8002264
99.8672732	99.738097	99.8162224	99.950534
99.9435254	99.5956822	100.013058	99.6921366
100.239747	99.7495906	100.208383	99.930473
100.140858	99.6890841	100.018914	99.7210304
100.181813	99.8180237	99.9373086	99.8367553
100.14756	99.9168422	100.048005	99.8681941
99.9704817	99.7419282	99.9560099	99.8526244
100.146964	99.8890991	99.9193629	99.8976868
99.9893959	99.5849812	100.157946	99.6861482
100.06535	99.7988677	99.7903428	99.8919978
100.013225	100.03125	99.78222	100.035269
99.8683157	99.6700602	100.182692	99.8289704
100.062967	99.8402183	99.9858565	99.8643017
100.024841	99.9188239	99.9820784	99.8737334
99.9387597	99.7503833	100.30189	99.9120588
99.9022718	99.8250256	99.9569544	100.059073

100.030798	100.035081	99.9072732	100.005327
100.106604	100.136409	100.351949	99.965954
100.000566	100.061107	100.02137	99.9207419
99.9812047	99.9622881	100.200638	99.9562229
100.083966	100.045518	99.8940501	100.023292
100.171537	100.155169	99.9140737	100.088266
100.129241	100.064938	99.9401422	100.021197
100.160368	99.9818405	100.08503	99.8925967
100.131773	100.153452	99.9543098	100.068355
100.127752	100.166795	100.097875	100.202494
100.08769	99.996901	100.079174	100.054432
100.175707	100.316212	99.8392685	100.320764
100.001012	100.15649	99.8507915	100.125693
99.9071865	100.066787	100.166825	99.9834699
100.034671	100.000204	100.321913	100.008022
100.146219	100.113026	99.9027396	100.033922
100.0932	100.105099	99.9903901	100.116262
99.9983317	99.9473597	100.081252	100.169708
100.08203	100.129011	99.8812047	100.127041
100.162453	100.081716	100.206872	100.176145
100.036607	100.001129	99.9569544	100.124196
100.083222	100.296923	99.6212755	100.121801
100.083966	100.086208	100.076341	100.153689
100.078158	99.9102367	100.146234	100.000836
100.163942	100.254912	99.7202602	100.195607
100.124475	100.412388	100.036104	100.159977
99.9963956	100.106685	99.9968128	99.9529293
100.143538	99.9591175	99.9811339	100.090063
100.030054	100.206032	99.9248411	99.9686487
99.9494826	99.965723	100.098442	99.9930513
100.072946	100.043932	100.027037	100.012364
100.160219	100.335764	99.9025507	100.175696
100.091711	100.151074	100.221228	100.025089
100.054776	100.13958	100.081441	99.9872126
100.282937	100.190839	100.103542	100.202045
100.209514	100.147639	100.407675	99.931521
100.267597	100.373151	100.038938	99.9846676
100.149347	100.350032	99.9760336	100.139466
99.9683967	100.021738	100.173058	100.027933
100.206834	100.184762	100.077852	100.18932
100.273257	100.455059	99.9093511	100.047246
100.147411	100.216336	100.131122	100.068804
100.13773	100.123859	99.9414645	100.153839
100.013225	100.196388	99.9546876	100.041557
100.231407	100.062296	100.091831	100.073744
100.211748	100.279881	100.219339	99.9661037

100.33685	100.573562	100.206872	100.08093
100.220237	100.212769	100.23993	99.9990396
99.8921445	100.128219	100.009469	99.8433425
100.313468	100.3643	100.003424	100.088416
99.935781	99.896233	100.167769	99.8731345
99.9196966	99.9834258	100.078796	99.9250835
100.106306	100.179345	99.965455	100.021346
99.9770347	100.206956	100.1264	99.9069687
100.250619	100.461533	99.9136959	100.17345
100.189558	100.231397	100.320024	99.9969437
100.276831	100.06573	100.251642	99.9388567
100.269068	100.294903	99.7837716	100.053229
100.21888	100.176172	99.9650312	100.194444
100.111057	100.045806	100.080962	100.120317
100.127737	100.224696	99.7665897	100.002014
100.323575	100.149332	100.291676	99.9922806
100.330426	100.326636	99.9937306	99.9795518
100.358126	100.390761	100.077941	100.182763
100.286641	100.365904	100.038101	100.206723
100.119099	100.240959	99.9467164	100.050983
100.415909	100.431352	100.057927	100.362164
100.201605	100.209359	100.170648	100.109086
100.271302	100.099618	100.04471	100.081232
100.470565	100.426327	100.000528	100.285193
100.223646	100.334701	100.504656	100.031365
100.336532	100.182651	100.089458	99.9045268
100.305406	100.394728	99.950115	99.9487032
100.302725	100.438359	99.8870518	100.024327
100.205477	100.122359	99.8980029	99.9910826
100.141885	100.100147	100.001661	99.9786533
100.261026	100.194947	99.895926	100.014144
100.106441	99.8866157	100.256935	99.9022806
100.193265	99.9553688	100.083228	100.031665
100.103015	99.8806659	100.211431	99.813778
100.035701	100.07648	100.325473	99.8971891
99.9569187	100.294903	100.094934	100.068653
99.9747898	100.1689	100.194627	100.256291
99.7247426	100.125136	99.80152	100.081083
99.9901292	100.080579	100.117403	100.227389
99.7312954	99.6815463	100.202934	100.046341
99.8629462	99.8153504	99.8515552	99.9499012
99.8543085	99.5363715	100.6693	99.9010826
99.7892277	99.3945022	100.005626	99.8932955
100.045232	99.7066677	99.999395	99.8616982
99.9695774	99.8009387	100.31811	99.8718812
100.141736	99.9137203	100.104186	100.067006

100.005915	99.8431161	100.130431	99.8080875
99.9968309	99.9145136	100.191606	99.8808663
100.037041	99.925091	99.9289681	99.965625
100.001299	99.8039797	100.140815	99.8925468
99.8547553	99.6174209	99.8081284	99.8417814
99.9408346	99.7304668	100.227858	99.8543604
99.8824556	99.5900518	100.461796	99.7595684
99.8443304	99.9379161	99.7747086	99.9641275
99.8221404	99.7900969	100.148368	99.9338779
99.8635419	99.7000568	100.111927	99.8994353
99.9740452	99.7744952	99.8417369	99.972813
99.7977165	99.7140718	100.137794	99.7188363
99.8152898	100.086793	99.7835828	99.9940776
99.8449261	100.079124	100.359837	100.007705
99.8279485	100.155017	99.9136743	99.9398679
100.022297	100.199839	99.8241774	99.9964736
99.9068795	99.8759061	99.9839124	99.9747598
99.8231829	99.9141169	99.7866038	99.8284536
100.106143	100.0109	99.9861781	99.9380709
99.9439621	99.6319648	100.17178	99.8901508
99.8385223	99.6006292	100.125144	99.8742772
99.9244528	99.3929156	100.22748	99.8582539
100.071443	99.7520183	99.9087652	100.086324
99.8870723	99.7444819	100.051507	99.8398346
99.9680881	99.6110744	99.9299122	99.9139611
99.9129854	99.9205956	99.7086244	100.077189
99.5996446	99.7677522	100.005059	99.8205168
99.9180489	99.7443497	99.8096389	99.9969229
99.9232613	99.9812834	99.8925274	100.039302
99.8425433	99.8531647	99.8981917	99.8007498
100.058486	99.9420148	99.7516736	100.091715
99.8954121	99.8804015	99.977304	99.9174054
99.8231829	99.8584534	99.8168137	99.9340277
100.043743	100.202087	100.015444	100.058021
99.8587763	99.9249588	100.104186	99.6776549
99.942175	100.023064	99.7122119	99.944061
99.8797749	99.9672684	99.9114086	100.000816
99.9457492	99.8942843	99.9287793	99.8453754
99.7735905	99.8523713	100.076997	99.8799678
99.8907954	99.7644467	100.037535	99.874427
99.8540106	99.9298508	99.8513663	99.9912324
99.7595914	99.8954743	99.971262	99.7459411
99.8970503	100.008652	99.9595556	100.090667
99.7938444	100.005479	100.22276	99.9021308
100.068911	100.288292	100.029416	100.042896
99.9591526	100.197591	100.042633	99.9404669

99.7872917	99.9282642	99.9125414	99.8363904
100.113589	99.9738792	99.8481565	100.047688
99.8173748	99.6680602	100.012989	99.9214486
100.029446	100.011958	99.66482	100.204477
99.9758323	99.9153069	100.160452	100.103395
100.096909	100.116939	99.6025121	100.151915
100.084102	100.226944	99.9004575	100.141282
100.090654	100.174057	99.9285905	100.200584
99.9768748	100.195476	100.264109	100.126158
100.050444	100.247702	99.9138631	100.223046
100.123269	100.405834	99.6752047	100.339402
100.106292	100.358764	99.5989246	100.205376
100.236602	100.620158	99.7156105	100.373545
100.045977	100.413502	100.006947	100.097855
100.218582	100.61249	99.6961628	100.338054
100.080974	100.268989	99.971262	100.224544
99.9534934	100.220729	99.6878551	100.006806
100.19118	100.093139	99.9593668	100.111931
99.8664242	99.6154277	99.7152504	99.9522174
99.9626597	99.6828683	99.8803097	100.049089
99.966527	99.4779067	99.8761549	100.061666
99.8244792	99.4738154	99.8142104	100.127844
99.8392046	99.3446091	99.8380061	100.022588
99.8378659	99.6482901	99.8974955	100.126497
99.5687932	99.6053973	100.366234	99.8358814
99.9753027	99.6125242	99.9360219	99.965992
99.9540327	99.6418232	100.22327	99.8647782
99.8189758	99.6864317	99.7169501	99.9117918
99.8630032	99.5312258	100.221004	99.9432339
100.18354	99.3204572	100.006654	99.7685054
100.089833	99.26793	100.560188	99.6657943
99.9177399	99.1900631	100.396262	99.8872369
99.8185296	99.5255508	100.00231	99.8200106
99.8612183	99.6661072	100.019496	99.8539981
100.050119	99.9476159	99.728848	100.192226
99.8856118	99.9418089	100.06331	100.010909
100.076447	100.086061	100.189465	100.036213
100.31354	100.476319	99.8604799	100.252265
100.132968	100.328504	100.234413	100.242683
100.074959	100.210516	100.141685	100.077986
100.145463	100.225825	100.035171	100.232352
99.9576025	99.9893209	100.580017	99.9225719
100.092362	100.063888	100.245366	99.9848573
100.15052	100.451507	100.09466	100.236694
100.053987	100.528186	100.166614	100.295536
99.8469391	100.099654	99.8285634	100.047592

99.8824883	100.024427	99.8699226	100.013006
100.013678	100.252353	99.7367799	100.291793
99.8383121	100.045016	100.03857	100.095504
100.040451	100.074051	100.146973	100.187584
100.029444	100.038285	100.14093	100.017348
99.9793187	99.9852296	100.375865	99.8731628
100.147099	100.078934	100.12601	100.172163
100.06306	100.375224	99.8752106	100.274724
100.102476	100.401884	100.136019	100.315898
100.039113	100.331671	100.039137	100.036213
100.169707	100.413894	99.9764369	100.2063
100.128209	100.245358	100.224592	99.9712324
100.060234	100.111136	100.071431	99.8024928
100.197671	100.147298	100.184933	100.176355
99.9662295	100.080122	99.9226132	99.9860551
100.055325	100.017168	99.6529282	100.113171
99.9941928	100.010965	99.7297922	99.9847076
99.8713327	100.006346	99.8767214	99.8674733
99.9723279	99.9575142	100.067654	99.9574577
100.037774	100.066528	99.7075073	100.159736
99.8054404	99.8997079	100.030261	100.000429
99.8671679	99.7932018	99.7316808	100.11407
99.8313213	99.9247837	99.9303562	100.023935
99.8225456	99.8342469	99.9441427	99.8207592
99.878621	99.9070987	99.7896593	99.9863546
99.8769848	99.9398292	100.028183	99.8212084
100.05592	99.9465601	100.087295	99.9857557
100.011596	100.088568	99.6580273	100.066008
99.8002344	100.075238	100.005143	100.072296
99.9735178	100.211836	99.7904147	100.197017
100.027511	100.203785	100.112601	99.8930762
100.1529	100.067848	100.011186	99.8674733
100.16108	100.310291	99.5116647	100.002525
100.08195	100.096091	100.151694	99.9288603
100.16227	100.138192	100.209862	99.9622489
100.086561	100.246546	99.9018391	99.9450306
100.093552	99.9895849	100.09636	99.9791678
100.048186	100.061645	99.8412167	100.084274
99.8766873	100.078142	100.166047	99.7816811
100.112293	100.171846	100.188143	100.057773
99.9590899	100.301185	99.8795542	100.016449
99.9471906	100.242058	99.7643527	100.045795
100.177144	100.315966	99.6799345	100.1518
100.055028	100.034325	100.123933	99.9164332
100.027511	99.9604178	100.005898	99.9847076
100.051756	99.899312	100.035926	100.094007

99.899296	99.7569079	100.082007	99.8453141
100.021412	99.9024795	100.064254	100.054479
99.8563098	99.7103197	100.405704	99.8286946
100.004753	99.7875268	99.9940004	100.031422
99.9973164	99.9781028	99.7354579	100.105386
100.001184	99.8822869	100.223648	99.76596
100.159147	100.092264	100.327896	100.011957
100.066035	100.163796	99.799102	99.9947392
100.011596	100.002387	99.9703935	99.944282
100.071836	100.09939	99.8191206	100.029326
100.069605	100.042772	100.039514	99.7972525
100.14338	100.073655	99.9152478	99.9984823
99.9927054	100.084345	100.165858	99.9688368
100.011893	99.8803072	100.010997	99.8331864
100.109467	100.034853	99.7809719	99.9083481
99.8790672	99.8751601	100.073131	100.05942
100.011893	99.9916965	99.3932526	100.198963
99.8090101	99.7309083	99.8179875	99.9641953
99.9956802	99.6795689	99.7753063	100.102241
100.017248	99.9548747	99.665015	100.067056
99.9001884	99.9069667	99.8905078	99.8981668
100.093552	99.9436566	99.8767214	100.164826
99.7697425	99.8628861	100.343004	99.7767402
99.8827857	99.8553634	99.991923	99.6900497
99.9579	99.8543076	99.9815359	99.9537146
99.7746509	100.010833	99.8523591	99.7108614
100.128357	100.04396	99.8126996	99.9454798
100.034617	100.107704	100.10749	99.8126533
100.08185	100.136931	99.9635671	99.925225
100.390129	100.223553	100.342452	100.168482
100.110905	100.146188	99.986421	99.8861543
100.216992	100.177266	99.8298431	100.017438
100.22027	100.203716	99.8936831	99.9125008
100.360031	100.357122	99.717651	99.9604037
100.460457	100.30105	100.140732	100.101717
100.421121	100.292321	100.064993	99.9102554
100.663542	100.411211	100.162642	100.042587
100.499345	100.459085	100.375316	100.137495
100.425442	100.569776	100.149232	100.152464
100.362564	100.086809	100.548326	99.9622001
100.378358	99.9683155	99.8611965	100.10501
100.057712	99.6827938	100.163586	99.9644455
100.488766	99.6607085	99.8838616	100.019234
100.337085	99.5776573	100.319975	99.8458859
100.065162	99.5690612	100.570613	99.8979803
100.055477	99.7946749	100.148665	100.08555

100.018525	99.9586615	99.813411	99.923279
99.9554984	99.8942571	99.7695918	100.020582
99.7775934	99.4918288	100.288055	99.9123511
99.8656519	99.7285513	99.8219104	100.105909
99.8033702	99.7862111	100.258969	100.098274
100.09377	100.11392	100.132422	100.196026
99.9343405	100.055466	100.280123	100.254408
99.8148432	100.058773	100.036662	100.097975
100.044749	100.119077	100.055927	100.269377
100.261543	100.220908	99.702541	100.384194
100.071271	100.186788	99.8942497	100.270575
100.358392	100.646347	99.4046841	100.446318
100.332317	100.657324	100.051772	100.421918
100.089449	100.59768	99.9913318	100.275964
100.076486	100.5141	100.244047	100.284796
100.097644	100.481831	99.5193316	100.279557
100.043855	100.459349	99.8356983	100.255605
100.182424	100.321151	99.9994535	100.237941
99.9814242	100.094744	99.9201257	100.008157
100.178699	100.068427	99.9930317	100.352758
100.081105	99.8479706	100.52585	99.9337577
99.836001	99.9291704	99.8402313	99.8894476
100.110905	99.8853965	99.7493821	99.9873491
100.100475	100.153329	99.4981775	100.234049
100.209095	100.468342	99.4492587	100.211295
100.108223	100.101753	100.03534	100.175667
100.071569	99.9700348	99.9839657	100.037198
100.100922	99.8881737	100.317898	99.8795676
100.004817	100.005874	100.10258	99.9632479
99.8769758	100.179911	100.252925	99.8675919
100.207307	100.336889	99.8305986	100.192433
100.146068	100.384894	99.9189924	100.257401
100.168418	100.383836	100.007386	100.146626
100.050858	99.9525781	100.213827	99.9660922
99.8121612	99.8028743	99.8566635	99.9081596
99.838981	99.8293237	100.162264	99.7961867
100.098091	100.203054	99.7692141	100.134201
99.8000922	99.9535038	99.7703473	99.913399
100.012118	99.9876236	99.8849948	100.192433
99.9362775	99.7144009	100.126378	100.062347
99.7181427	99.6265887	99.9127595	99.8337605
99.9660773	99.7448177	99.7763913	99.8891482
100.009138	99.8536572	99.7082072	100.066988
99.8129062	99.9084075	99.5780719	99.9557631
100.030594	99.8094866	100.060649	99.9665413
100.085873	99.9491397	99.9545011	99.9340571

100.15173	99.7319898	100.41838	100.024324
100.106733	99.7676965	99.7371052	100.020133
99.9295725	99.5717061	100.366817	99.7437929
99.8891938	99.6161412	100.329041	99.8903458
99.7170997	99.9683155	100.120334	99.8327126
99.6528811	99.8909509	99.7652477	99.7851092
99.9504324	99.8641047	99.9601673	99.8346587
99.8276571	99.816628	99.9533678	99.9623498
99.7984533	99.6813391	99.7932013	99.8187909
99.7439196	99.5970976	100.390615	99.814749
99.9155666	99.7769538	100.295422	99.9319614
99.8835318	99.7975844	100.572691	99.714153
99.9021567	99.7834339	100.199284	99.8282217
99.9617564	99.9065561	100.322053	100.033456
99.9154176	100.025975	99.7012188	99.9767206
99.9030507	99.9673898	99.6845978	100.114441
100.110309	99.9751924	99.6504113	100.139291
99.8243791	99.9127717	99.9443018	99.8460356
99.8357031	99.9580003	99.9731998	99.8470835
99.7471975	99.8041967	100.181152	99.7204403
99.9133316	100.216808	99.7440936	100.036449
99.843004	99.9416016	100.15792	99.8653465
99.7984533	100.24339	99.9099264	99.7845104
99.7467505	100.112597	100.101446	99.7288233
99.8194621	100.174489	100.252169	99.6803216
99.4681221	100.028224	100.046672	99.6315206
99.7290196	100.05626	99.6377566	99.8434908
99.7375126	99.8658239	100.168308	99.8702864
99.5359167	99.7581747	99.9208812	99.6940937
99.7577765	99.6398135	99.9724442	99.7345118
99.7921953	99.9062916	99.7992453	99.8563647
99.7592665	99.9127717	99.7826242	99.8454368
99.9724843	100.055995	99.9216367	100.053066
99.8032212	99.8190084	99.9265475	99.9166923
99.6682545	99.6221241	99.8689184	99.8249897
99.6742388	99.4499741	99.6485406	100.18231
99.7648955	99.3802524	99.6416693	100.309539
99.8090578	99.6116601	99.8983122	100.106668
99.886597	99.5231178	100.013578	100.273526
99.8861597	99.6013943	100.060646	100.283537
100.083068	99.9579874	100.012376	100.176331
99.8089121	99.594693	100.28654	100.035753
99.7957946	99.6260607	100.011173	100.253364
99.5978657	99.3130971	100.152035	100.137397
99.7689765	99.547499	100.273313	100.096656
99.5936389	99.2721766	99.950534	100.030608

99.8768317	99.4378548	99.8600047	100.073296
99.6838584	99.499592	100.086242	100.207756
99.7125712	99.457816	100.109089	99.9227069
99.7501747	99.3095326	99.720861	100.04493
99.6918746	99.2758837	99.5683182	100.060643
99.8239244	99.3658518	99.7985066	100.09026
99.6411535	99.3133823	99.6615961	100.112091
99.6910001	99.3689886	100.298737	100.154083
99.5480191	99.5186978	99.9113676	100.167154
99.4659617	99.3597209	99.9513929	99.9215945
99.6968301	99.6919327	99.9962281	100.141291
99.4799537	99.5256842	100.13039	100.035475
99.7796163	99.9090824	99.8160284	100.203862
99.7114052	99.5797221	99.8830235	100.146575
99.6487325	99.5908434	100.018044	99.9524633
99.4808282	99.4864747	99.5167835	99.9945949
99.6647651	99.4967404	99.7201738	99.9608061
99.6589351	99.4494038	100.233631	100.035614
99.9991162	99.8975334	100.211471	99.8967049
100.105514	99.9403074	100.127985	100.096795
100.133352	99.9578448	99.9941667	99.9663681
100.184073	100.112544	100.0244	100.254198
100.310002	100.233167	100.063395	100.140735
100.104785	99.9775209	100.303203	100.093875
100.028558	99.9477216	99.8022858	99.9299374
100.191798	99.9036643	99.9534543	100.168266
100.100996	99.9926344	99.9029503	100.033806
100.139911	100.191534	100.37518	100.106529
100.25826	100.314723	100.222294	99.902823
100.246017	100.204081	100.165949	100.17202
100.215701	100.35436	100.065628	99.9680367
99.9074393	99.94045	100.467942	100.086089
99.9491239	99.9050901	100.294099	100.000713
100.158275	100.336253	100.089162	100.062589
99.8855767	99.9658293	100.144476	100.083447
100.137287	100.210212	100.150832	100.069264
100.034534	100.173141	99.9154904	100.044096
100.152008	100.226894	99.6765411	100.053273
99.9814804	100.153465	99.8653299	100.046738
100.104056	100.10456	100.046217	99.9616404
100.067182	100.106413	100.013063	99.9731814
99.9730269	99.9403074	100.199447	99.8962878
100.207393	100.29519	100.265411	100.007387
100.017043	99.9685383	100.01083	100.11946
99.9886222	99.8784276	99.9378221	99.937307
99.9513101	100.037832	99.5257162	100.041871

100.153903	100.181981	99.626896	99.9663681
100.087878	100.227464	99.8397344	99.8477599
100.133061	100.319856	99.6909708	99.7598814
100.119943	100.614569	99.8079546	99.8919773
99.9196823	100.10128	99.9989766	99.7925578
100.23013	100.467141	100.083837	99.9069945
99.9988247	99.9976247	100.002584	99.8950363
100.280851	100.484393	100.133482	99.9453718
99.9698204	99.8570406	100.187078	99.8908649
100.268754	100.407685	99.7346036	99.9456499
100.18349	100.242577	99.6914862	99.9302155
100.294115	100.548982	100.12558	99.9491261
100.240333	100.30959	99.9098216	99.9371679
100.391767	100.748452	100.123691	99.9976539
100.117757	100.135215	100.043468	99.9043526
100.229256	100.387154	99.8764958	99.9930653
99.7625635	99.7915963	99.9328404	99.9375851
100.327783	100.36648	100.13228	100.064675
100.020396	100.150471	100.105997	100.006692
100.050712	100.149615	100.122316	99.9955682
99.7137372	99.8202549	99.8364705	100.06245
99.8835362	99.8973908	99.7392417	99.9192307
100.059311	100.21677	99.6390926	100.06106
99.9306136	100.336253	100.021824	99.9346651
100.024768	100.472702	99.9945103	99.8559638
99.8208636	100.04767	99.9156622	99.7754548
99.8567182	100.067632	100.085898	99.9580252
100.052752	100.059077	99.9166929	99.8330208
100.375589	100.449746	99.796617	100.013227
100.305629	100.33169	100.067518	99.9507947
100.402844	100.346804	100.051542	100.123493
100.26409	100.09686	100.009971	99.8723715
100.370196	100.093296	100.138807	100.070515
100.099684	99.83822	99.8230715	99.8217579
100.27473	100.415385	99.7309961	100.030052
100.064412	100.076899	99.8991711	99.8957316
100.110615	100.355644	100.176428	100.101662
100.028558	100.076329	100.10239	99.7733691
100.190195	100.41624	100.199275	99.9174231
99.7216077	100.064067	100.000866	99.7336013
100.331573	100.637525	100.015468	99.8822439
100.2243	100.320284	100.166808	100.121685
100.00859	99.8131259	100.732831	99.8412247
100.158794	99.9903245	100.38872	100.128172
100.641718	100.717398	100.000094	100.134579
100.740438	100.879113	99.9036683	100.183463

100.589944	100.695626	100.179196	100.062436
100.625191	100.929962	99.7458803	100.03876
100.453784	100.735159	99.7812881	99.7811064
100.665996	101.037963	99.6424072	99.7057603
100.645228	100.890572	99.8256337	99.786538
100.563034	100.864359	99.8852769	100.038481
100.19404	100.273362	100.087754	99.9089581
100.56786	100.691185	100.330796	99.8141139
100.126326	100.291983	100.294013	99.8535278
100.095028	100.370047	100.325296	99.943219
99.8522493	100.178968	100.295388	100.022743
100.070165	100.342402	100.069019	99.8731652
100.216417	100.585333	100.063691	100.074135
100.389725	100.58032	99.9196533	100.017451
100.340292	100.539927	99.5379027	100.119537
100.404789	100.648214	99.6131872	99.9851398
100.408884	100.680299	100.246917	99.915086
100.097806	100.358158	100.37497	99.9600709
100.041353	100.154331	100.332515	99.9018552
100.15426	100.12153	100.447848	100.068564
99.9669109	99.5792331	100.383564	99.8536671
99.8450829	99.5396996	100.324092	100.007423
99.9673496	99.4275447	100.71839	99.8060361
100.022925	99.4288338	100.567822	99.9317987
99.6787944	99.093515	100.265481	100.022604
100.062852	99.6952554	100.191571	99.9632741
99.886326	99.4720915	100.212885	100.016058
100.115064	99.611748	100.234026	100.097672
100.253126	100.0078	100.093598	100.119537
99.936783	99.6794993	100.353828	99.9237209
99.8651195	99.5026011	100.302091	99.7975405
100.140658	99.848806	100.198446	99.8258127
99.7253025	99.5346863	100.118006	99.8400185
99.8929074	99.5010255	100.5649	99.8227487
99.760403	99.5603258	100.308107	99.9962816
99.6916646	99.395746	100.294701	99.8780397
100.199744	100.023126	100.082082	100.143631
99.8762346	99.70342	99.956608	100.058258
100.379342	100.325357	99.6987846	100.068285
99.8864723	99.7582799	100.054409	99.9600709
100.086984	99.9126899	100.338531	99.9278991
100.027605	100.065238	100.008173	100.05018
99.7659606	99.7356484	100.041862	99.9700985
100.065485	100.091593	99.823743	100.100875
99.8367466	99.7340728	100.072285	99.9260885
99.9777335	99.945348	100.011095	100.017312

99.7462166	99.7181734	99.8978243	100.03681
100.10936	100.239128	100.099958	100.141124
99.6793794	99.6591596	99.931857	99.9308238
100.036527	100.108209	100.003876	99.9163395
99.9227428	99.9694119	100.091364	100.153241
100.014443	100.080564	99.7321297	99.9993456
100.066216	100.20561	99.6408602	100.223573
99.8814997	100.180687	99.8239149	100.057561
100.177075	100.575306	99.6181718	100.163826
99.8964174	100.363458	99.816524	100.126501
100.464314	100.779849	99.9232629	100.217028
99.811445	99.8734428	99.8216804	100.269812
100.023072	100.101906	99.8907771	100.073717
99.702341	99.8669971	100.064378	99.9937747
99.6352113	99.6794993	100.050971	99.9330521
99.7305676	99.8847586	100.115943	100.001156
99.793456	99.7822006	99.9031526	99.8891815
100.072651	100.032436	99.8545099	100.033606
99.5531639	99.4168019	99.8319934	99.9012981
99.8576606	99.7435265	100.215463	99.8865353
100.050567	99.9321701	100.015048	99.8934989
99.6173686	99.4473115	100.098411	99.8597951
100.085083	99.8214476	100.339562	99.8763684
99.7270576	99.4819749	99.8945585	99.9784548
99.7845346	99.4924312	99.9868593	99.7735857
100.032432	99.8400685	100.018486	99.9713519
99.6157598	99.3639473	100.053034	99.8283196
99.7696169	99.6227773	99.9371853	99.8313836
100.024534	100.03258	99.852963	100.026086
99.8939311	99.8685727	100.014532	99.8900171
100.100146	100.143875	99.7964137	100.157698
99.8589769	99.6737698	99.7579121	99.9217711
99.9911887	99.9931893	99.9543736	99.9787333
99.7808783	99.7718875	100.025877	100.017869
99.6723593	99.7859247	99.7407239	99.9128577
100.280329	100.397119	99.566779	100.043773
99.7936022	99.842217	99.492182	100.04252
100.278574	100.338391	99.3510667	100.223991
100.37276	100.626299	99.4535085	100.28917
99.9171852	100.268492	99.7496617	100.25477
99.8165638	100.397835	99.7671937	100.331927
99.5898732	100.083286	99.7795693	100.104078
99.9265453	100.119954	100.278544	100.166611
99.6356501	99.6759184	100.169742	100.140707
99.9062163	99.7491127	100.355031	100.067032
99.5879719	99.5220814	99.7335048	99.9794297

100.022194	100.054925	99.4543679	99.9866718
100.183218	100.389384	99.5602474	100.091404
100.05554	100.370333	99.6241877	100.100736
100.708555	100.849463	99.3385193	100.197808
99.5739474	99.5668871	99.2978952	100.223054
99.6863258	99.8204973	99.3937241	100.04626
99.3199315	99.3385096	99.8263277	100.121571
99.1778572	99.1447736	99.8718378	99.9155433
99.4633158	99.3219729	99.8113866	100.063243
99.4864611	99.1432055	99.7674221	99.9009264
99.5394478	99.133369	99.8526033	100.016191
99.3026089	98.7833897	99.8601597	99.7013022
99.5634665	99.0177547	99.9827794	99.9368421
99.0934279	98.6995658	100.020733	99.7482153
99.4909737	99.0756331	99.9741926	99.7619969
99.5055305	99.0759182	99.3719136	99.8877017
99.357488	99.1181153	99.7961021	99.714527
99.5506565	99.6226272	99.3234839	100.122267
99.492575	99.7312562	99.5743906	100.093173
99.8567857	100.044598	99.7504202	100.188948
99.8281089	99.9428118	99.7868283	100.152336
100.272091	99.7878516	99.9072154	99.6748527
100.29873	100.213386	100.244162	99.7838525
100.531784	100.050728	100.788222	100.168902
100.575745	100.27711	100.349093	99.9509021
100.523195	100.064699	100.583856	100.232798
100.340362	99.859273	100.718154	99.9648229
100.194066	100.024212	100.612536	100.226395
100.457544	100.092925	100.361973	99.9603682
100.274857	100.071541	100.46553	100.097488
100.360596	100.024925	100.496786	100.020645
100.475158	100.305336	100.116905	100.236696
100.332938	99.9837259	100.179246	99.9673286
100.553328	100.29108	100.182165	100.014799
100.126232	99.972749	100.593817	99.7768921
100.323185	100.306762	100.63469	99.9791613
99.8803677	99.9110215	100.467247	99.8701615
99.8949245	99.7921283	101.169133	100.346113
99.9598478	99.7745937	101.190772	99.9840336
100.067859	99.8977636	100.761775	99.9141512
100.482582	100.358653	100.195561	100.020506
100.677788	100.621387	99.8909005	100.202729
100.170048	99.945663	100.130816	99.9074692
99.9946385	99.9121619	99.8325102	99.778841
100.13482	100.122862	99.7864848	100.117673
100.121428	100.085512	99.9293694	100.148438

99.8473238	99.7579144	99.8725247	99.9969799
99.7905524	99.6385936	100.25292	99.8989775
100.084599	99.924707	100.240555	100.12533
100.065239	99.9161535	100.119138	100.159018
100.276603	100.140539	100.13614	99.9618995
100.414893	100.206971	99.900346	100.173078
100.026518	99.9966987	99.8031433	99.8826902
99.8580959	99.7993988	99.9506647	100.153171
100.122738	100.079382	100.084104	99.9649621
100.16932	100.380179	100.014379	100.043615
100.039619	100.154653	99.8220343	100.01285
100.269179	100.265563	99.9511799	100.098741
99.8813867	99.9453779	99.8742421	99.9618995
100.127542	100.201839	99.8623923	100.091363
99.7806538	99.8311891	99.7110926	99.9822239
100.010942	99.9681871	99.5951707	100.030251
100.067568	100.178887	99.8797376	100.034705
99.9282596	99.9791641	99.9441387	99.9710872
99.6644906	100.035189	99.9247325	100.241847
99.684579	99.8290508	99.7797871	99.9673286
100.101049	100.111885	99.919237	100.263424
99.8543111	100.329428	99.7986781	100.045703
99.8685767	100.27098	99.7045667	100.191871
99.6873448	99.9596337	99.5297392	99.9319698
99.9969676	100.217093	99.3561138	100.210664
99.6928763	99.9596337	99.637933	100.134517
99.8666844	100.135407	99.9717883	100.164029
99.7123824	99.8599858	99.9267934	100.010483
99.7598375	100.131416	99.997377	99.9786045
99.7310151	100.034476	99.8514011	99.7143877
99.7527047	100.121864	99.9889619	99.8764258
99.6567755	99.923424	99.8388644	99.7632497
99.8797855	100.167198	99.8311363	99.9470043
99.7441162	99.5447906	100.100247	99.7980517
100.149086	100.084372	99.7833936	100.018975
100.09639	100.101764	99.6952929	100.031782
100.320419	100.581898	100.036018	99.9305777
100.161168	100.226929	100.121027	99.9176314
100.047043	100.194996	100.267518	99.8771219
99.9487846	100.116875	100.105399	99.8412062
100.27966	100.336841	99.9838098	99.9373989
99.9019118	100.052296	100.270266	100.058092
99.9765881	100.130133	100.191611	99.9172138
99.7736665	99.9659062	99.7971325	99.9227821
100.067131	100.32729	99.606677	99.8828294
99.8457226	100.086653	99.6738259	99.924731

100.100321	100.62937	99.9800316	100.073127
99.908899	100.216665	99.9204391	99.9179098
100.061309	100.207827	99.9499778	99.9233389
99.8537288	100.116447	100.188004	99.8877017
100.109783	100.402133	99.837834	99.9248702
100.204838	100.385596	99.9489473	100.036097
99.9831386	99.911164	99.7265489	100.078277
100.002354	100.059282	99.6355287	100.218738
99.9911449	99.9445225	99.3365359	100.077999
100.176162	100.368774	99.6655825	100.154285
99.9070067	100.036615	99.7504202	100.06547
100.188972	100.423802	99.690656	100.045424
99.9250571	100.101906	99.7837371	99.8844999
100.234104	100.588506	99.8559143	99.9439952
99.9165228	100.163913	99.8504193	99.8640356
100.610794	100.838603	99.6354261	99.8438719
100.092535	100.431016	99.5136768	99.862645
100.197617	100.291248	99.4601002	99.8524936
100.623054	100.29525	99.5124747	100.148413
100.092535	100.031004	99.3311386	100.027153
100.32211	100.384427	99.6443555	100.188185
99.6952668	99.8875203	99.7679938	100.075824
99.6414122	99.7790497	100.067817	100.043006
99.7311698	99.8119196	100.090655	99.9964208
100.308099	100.078451	100.069019	99.97139
100.210898	99.9967054	100.202273	99.8184239
100.75499	100.338266	100.098726	99.8715449
100.386765	100.236084	100.160889	99.9103426
100.561026	100.373423	100.13204	99.7694748
100.450836	100.279529	100.277315	99.6714374
100.71646	100.414153	100.443883	99.8873977
100.525415	99.9431132	100.607188	99.7397159
100.113114	99.7619002	100.599118	99.5795187
100.231331	99.7593278	100.550693	100.002261
99.7764134	99.5082306	100.59139	99.9937787
99.7661971	99.3691768	100.448176	99.7669717
100.247385	99.9002395	100.266668	100.038973
100.090054	99.8673696	100.477712	100.026319
100.131503	100.003994	100.324022	99.989885
100.298175	100.353987	99.7343367	99.9962817
100.03401	99.8344998	99.7379428	100.039668
100.199223	100.211932	100.287274	100.022981
99.8022461	99.6272767	100.375195	99.9175738
100.050065	100.05387	100.10216	99.8462359
100.225201	100.272098	100.170333	100.030073
100.039702	99.9987062	100.309598	100.069288

100.424565	100.364133	100.545541	100.119906
100.266212	100.119324	100.15007	100.231849
100.080276	99.8446466	99.8825309	100.007128
100.281829	100.365705	99.6196279	100.119628
99.99621	100.04258	99.8476718	99.9839054
100.358159	100.325118	100.153848	99.9672182
100.386619	100.521051	99.7183668	100.033967
99.8406302	99.8097759	100.183212	100.100438
100.240088	100.196497	100.30376	100.168299
100.317586	100.356559	100.003422	100.264111
99.9705233	99.8323561	99.910006	100.152585
100.294526	100.348556	99.8837329	100.278017
100.27745	100.521194	99.8682781	100.112258
100.10027	100.171059	99.7630139	99.8554139
100.113698	100.383284	99.9385115	99.9572059
100.01854	99.9921322	99.880642	100.04384
100.059697	99.7253147	100.128434	99.9601261
100.312332	100.189066	100.11109	100.144659
100.176163	100.18535	100.184414	100.13117
99.9204634	99.7941985	100.14303	99.9823757
100.157482	100.178061	100.220819	100.054965
100.332327	100.322975	99.9144708	100.072626
99.9255716	99.7746194	99.8531668	99.9453858
99.9293662	100.004994	99.5205456	99.9075614
100.160693	100.165199	99.7971861	99.9834882
99.8390248	99.8376438	100.019735	99.8789151
100.036346	100.055157	100.248809	99.89741
99.5833252	99.5005134	100.109888	99.8586123
99.8254517	99.8577945	99.9695928	99.9844616
99.6882613	99.8893782	99.8456111	99.9941958
99.8930253	99.8022016	99.7013665	99.9983676
100.185066	100.140047	100.117959	100.147162
99.7142399	99.6459982	99.9058848	100.007963
99.658488	99.7669022	99.9155011	99.9823757
100.077211	100.325976	99.5313639	100.135203
100.19499	99.9992778	99.7867112	100.055104
100.247093	100.348413	99.7899739	100.076519
99.7908622	99.8938084	99.7542562	100.115456
99.8631061	99.9634068	100.122767	
99.9122903	100.299823	100.029008	
99.7561268	100.052298	99.8078328	
100.391581	100.652102	99.7987316	
99.8995929	100.105033	99.691235	
100.095892	100.335265	99.3486541	
99.871717	100.374995	99.6903764	
99.8544952	100.28496	99.9651281	

100.061594	100.427301	99.6922654	
99.8096894	100.053585	100.100443	
99.999129	100.148621	99.9967245	
99.8698197	99.9519738	99.843207	
100.119098	100.342982	100.176172	
99.675126	99.7468944	99.9213396	
99.713948	99.7806218	99.6670225	
99.8822252	99.9965625	99.788085	
99.8270571	99.679011	99.6694266	
100.115449	100.109035	99.4801914	
99.8063326	99.7063073	99.9747444	
99.5188165	99.6755811	99.843207	
99.4532862	99.4099069	100.065756	
99.7514565	99.8369293	100.045665	
99.1987541	99.1166505	100.014412	
99.5698981	99.5175199	99.8845915	
99.2321761	99.3060095	100.091342	
99.4184048	99.5576783	100.147666	
99.3175552	99.5625374	100.140626	
99.3308364	99.3853259	99.8844198	
99.6082822	99.9479723	100.029008	
99.6938302	99.4974679	99.8593524	99.9009333
99.6699204	99.3380665	99.8992783	99.88162
99.8353708	99.611558	99.9297744	99.9018315
99.8988352	99.7336182	100.053364	99.7557097
100.023845	99.7610706	100.1918	99.882668
99.7822377	99.569051	99.9885597	99.8525753
99.8932268	99.6409292	99.9761205	100.132542
99.7754485	99.4992391	100.113554	99.8687445
99.9081335	99.6875689	100.099911	99.9091676
99.66933	99.5560627	99.8936606	99.9076704
99.8610517	99.6812223	100.03932	100.096611
99.9056245	99.7808482	100.094895	99.9733953
99.8207592	99.8254216	100.09971	99.9185996
99.8862899	99.7370128	100.256203	100.091371
99.9255494	99.6834362	99.846512	99.9813302
99.9001636	99.6241035	100.04273	100.099905
99.9367663	99.6615923	100.325622	100.009027
99.9286488	99.7610706	99.9323826	99.9468958
99.9023774	99.7157593	100.123987	99.7890961
100.183097	99.9817235	99.9211472	100.127302
99.8976545	99.7495583	100.143448	100.1285
99.9336669	99.7491155	100.131811	100.08658
99.8377322	99.8508077	99.9093099	100.02864
99.8634132	99.921948	100.135623	100.001242
100.04687	99.8382622	100.290913	100.065171

99.8758109	99.6072778	100.120977	100.017262
99.9091667	99.6890448	100.101716	100.00768
99.9248114	99.5556199	100.113353	99.8883572
99.9420796	99.7387839	99.9165327	99.9474946
99.8728591	99.583958	100.052762	99.7730766
100.063695	99.9175201	100.010629	100.248422
100.055135	99.9433491	100.066204	100.212939
99.9645136	99.9858561	99.8178215	100.088526
99.9868	99.9613555	99.814812	99.9970503
99.7274812	99.8738323	100.252993	100.082538
99.9966886	99.9585512	100.308769	100.029389
100.053216	99.9269661	100.059383	100.010525
100.041114	99.9671117	100.35973	99.8790749
99.8963262	100.041499	100.078242	99.8804223
100.085096	100.09478	99.8471139	99.92893
99.9264349	99.969916	100.151273	99.8561685
99.9898994	99.997516	100.343679	100.038521
99.8302051	100.010209	99.9795312	100.047355
99.8603138	100.01995	100.104124	100.022202
99.8079187	99.6041783	99.9323826	100.032832
100.045984	100.029396	99.949637	99.8849138
99.9068052	99.9845278	100.227112	99.9178511
100.090705	100.103046	100.081252	100.043163
100.068418	100.036776	99.8727948	100.033132
100.060301	100.047108	100.018855	100.105594
99.8397985	99.9855609	100.149467	100.092568
99.9326338	100.063343	99.9530478	100.083136
99.877582	99.7587091	99.9875565	99.9847737
100.042442	100.362663	99.9000808	100.136435
99.9633329	100.166068	99.6825953	99.9668079
99.8778772	99.9669641	100.043332	100.162485
100.040671	100.16046	100.453826	99.9368649
100.224866	100.316319	99.6613283	99.9979486
99.9735167	100.009766	99.6968402	99.9894148
99.9906373	99.9635694	100.081854	100.166079
100.062072	100.112344	100.299139	99.9835759
100.192395	100.272336	99.8262481	99.9698022
100.019565	100.107326	99.8750018	100.162336
100.001559	100.063195	100.011231	100.185691
100.020598	100.200458	100.044937	99.9852228
100.17985	100.327388	99.8621613	99.8852132
100.204055	100.281339	99.7993633	100.212939
100.092033	100.186141	99.8545373	100.094814
100.146642	100.370191	100.106532	100.035976
100.208778	100.414174	100.11556	99.7898447
100.219552	100.579479	100.0331	100.051397

100.015433	100.261561	99.9975881	100.047953
100.290987	100.422734	99.8683809	100.126105
99.9887186	99.9626839	100.01725	99.8929984
100.204498	100.221268	99.8426999	99.9513872
100.023403	100.016703	99.9957825	99.7798138
100.114467	100.216545	99.676777	99.9853725
99.9153655	99.9659309	100.066004	99.7940367
100.176013	100.222744	99.99819	99.998248
99.9172842	100.100832	99.9297744	99.9112636
99.9437032	100.135221	99.7269351	99.9732456
99.9711552	99.9663737	99.7092794	99.9560284
100.061924	100.115148	99.8045798	100.021454
100.004511	100.103784	99.9759198	99.9274328
100.056758	100.17271	99.7975577	100.16533
99.986062	100.143044	99.9598693	100.092119
100.034325	100.141272	100.256805	99.9762399
100.015876	100.006076	100.063997	100.051097
100.054692	100.149538	99.9221504	99.897939
100.018237	100.191454	99.9466275	99.8322141
100.12406	100.228796	99.7939463	99.8632051
100.21173	100.205033	100.08707	100.237792
100.089081	100.136697	99.9570604	100.105743
99.986062	100.098618	99.8202291	100.116373
100.063253	99.944825	99.9490351	100.008728
100.151808	100.302888	99.7770932	99.9675564
100.061777	100.212708	99.8842308	99.7049563
100.080964	100.096847	99.8471139	99.9233905
100.168485	100.044156	99.7981596	99.780263
100.101331	99.9522047	100.133617	99.903179
99.7093066	99.6491371	100.18052	100.032196
100.055371	100.167431	100.145005	100.231781
99.8319418	100.058961	100.051502	100.057519
100.030578	100.324159	99.8907817	100.083441
99.9835017	100.226463	100.236702	100.113109
100.150114	100.317961	99.8233633	100.055121
100.027184	100.076228	99.859079	100.164653
99.951773	99.9373571	99.8771375	100.083441
100.014345	100.20285	99.9357273	100.011968
100.099644	100.302613	100.039263	99.7972491
100.206931	100.33021	99.9030213	99.904084
100.152033	100.195766	100.132766	100.023655
100.083705	100.258487	100.212022	100.23298
100.006081	99.9729234	100.202993	100.027551
100.108941	100.194438	99.8805485	99.9944366
99.8571772	100.018673	99.9455591	99.7905064
100.138013	100.272507	99.8089165	100.044033

99.8508314	100.047008	99.9951197	99.8433994
99.9628412	100.15961	99.9812748	100.128542
100.019805	99.9248129	99.943352	99.8583833
100.223754	100.383486	99.6734778	99.8260181
100.07736	100.247566	99.9630157	99.8935953
99.982026	99.9941746	100.210016	100.118653
100.161478	100.364153	100.151627	100.091682
99.9293415	99.9367668	99.978867	100.158809
100.079868	99.8803919	100.052104	100.418779
99.9671208	99.987386	100.253757	100.045681
100.125764	100.327849	99.8679076	100.111311
99.8778377	100.007457	99.8691115	100.164204
100.085329	99.8917555	99.9483682	100.177389
99.9867484	99.8929361	100.483702	100.032645
100.093741	99.9484254	99.9987314	99.8469955
100.158674	100.120059	100.124338	100.035942
100.15159	99.9600841	99.9006135	99.9010872
100.252532	99.9912231	99.6925395	99.956977
99.9225531	99.7601158	99.9511773	99.7870601
100.081639	100.044646	100.011172	99.977355
99.8716396	99.8945594	99.8699141	99.8165783
99.974352	99.9921085	100.016389	99.9343514
99.9495594	99.7215979	99.9782651	100.164953
100.01036	99.8548609	99.9939158	99.984847
100.032939	100.006866	99.9533845	100.081942
100.09197	99.8622398	99.8650985	99.9686644
100.211358	99.9993399	99.8456354	100.090633
100.311267	99.7949442	99.9160636	99.9358498
100.113958	99.7100869	99.9277013	100.052424
99.9386388	99.8042417	99.9531838	99.7611381
100.05906	100.091133	99.9772618	99.9721107
99.9389339	99.8790637	100.22045	99.8032427
100.244563	100.171268	99.694546	99.9351006
99.9330309	99.9776459	99.9710417	99.8210735
100.182728	100.07608	99.8323925	100.015864
100.102595	99.9510818	99.9529832	100.08374
100.156017	99.948573	100.110895	100.126295
99.9837969	99.8623874	100.07558	100.024404
100.116615	99.9510818	100.060331	100.225637
100.032054	100.173925	100.021405	100.135585
99.9579712	100.075343	99.9443552	99.9210158
99.9848299	100.206392	99.9004129	100.07505
99.707093	99.8821629	99.9696371	100.006124
99.9491166	100.079622	99.9670287	100.264296
99.9597421	99.8899845	100.215233	100.277482
100.047254	100.047155	100.012576	99.9875441

100.058027	100.118288	100.003346	100.096926
99.8278097	99.6638949	99.8769368	100.008521
99.9597421	99.9795644	100.125943	100.226686
99.8314991	99.6572539	100.168682	100.15806
99.9711054	99.9673154	99.9234876	99.9881434
100.071161	100.11386	100.135575	99.9141232
99.9464603	99.8138342	99.9264974	99.8661749
99.9541342	100.134817	100.181323	99.9947363
99.9749423	99.8792113	99.9921099	99.7672814
99.9752375	99.9469497	99.8889758	100.050776
99.7907683	99.8772928	100.080195	100.026652
99.973319	100.049516	99.9242902	99.8847548
99.9842396	100.144262	100.015385	100.080144
99.9601848	99.7559836	100.007761	100.082392
99.8799038	100.002734	100.138183	100.011818
99.9060246	100.067373	99.9971262	99.7591902
99.8531926	100.041842	99.786243	100.048379
99.7646474	99.7087587	100.373546	99.961622
99.9367203	99.9853199	100.255563	99.8477447
100.084148	100.15164	100.069561	100.003127
100.002391	100.020296	99.9050278	99.9772052
99.8889059	100.025018	100.135374	100.1215
99.7861934	100.000373	100.252955	99.782565
99.9966359	100.204326	99.9423487	99.9130743
99.8636705	99.912564	99.9439539	99.9436414
99.8010985	99.9316015	100.376355	100.071154
99.8314991	100.212885	100.109089	99.9295566
99.8971701	100.157986	99.9907054	99.8798102
99.9305221	100.058224	100.105477	100.022756
99.9514778	99.8325767	100.177109	99.9427424
99.8751814	99.9836966	100.044078	99.8690218
100.03353	99.9082842	99.8432276	99.8538881
100.006671	99.894707	100.000738	100.111311
100.079868	99.9763177	99.6648498	100.049577
99.974352	99.8452684	99.8426257	99.9752573
99.8794611	99.4404617	99.7023714	99.8646765
99.8838267	99.7887463	99.4312318	100.074605
99.8599184	99.7996665	99.8495074	99.8532629
100.153754	99.9565336	99.9522206	100.038938
100.135601	100.030466	99.8653557	100.162572
99.9973171	99.9848671	99.9002621	100.16482
100.118039	100.068097	100.191751	100.216821
99.9762129	99.5783111	99.996957	99.9220481
99.9731137	99.8471841	99.7750804	99.9296909
99.8444223	99.9382349	100.193155	99.8982205
100.085866	99.963617	100.012404	99.8360291

100.030966	100.023235	99.988732	100.072806
99.9286916	99.9779313	100.023037	100.215322
99.9738516	100.080197	100.06998	100.200486
99.9269206	99.6900218	99.8489055	100.051077
99.9815259	99.8622362	100.091646	100.030246
99.8732008	99.9155091	100.442917	100.057371
100.081439	100.114581	99.9833154	100.055572
99.8895824	100.189104	99.9883307	100.124957
99.930315	100.326197	100.032666	99.992332
99.7859799	99.9652402	100.046709	99.9385326
99.9332666	99.9974105	99.8808028	99.9536683
99.9959889	100.109416	100.104886	99.7467131
100.092803	100.126387	100.080612	99.8147491
100.022258	100.104694	99.9339649	100.070858
99.8332061	99.9609607	100.06035	100.042535
100.027867	100.021907	100.243308	100.131401
99.6549273	99.6953343	100.190146	99.9331377
99.9297246	99.9205265	99.8352639	100.061867
99.8270077	99.8846669	100.158851	99.7901722
99.9260351	100.060128	99.9907381	99.8701969
99.9117197	99.9199362	100.144607	100.018857
100.026391	99.9224449	100.269388	100.063365
99.8953381	99.8918978	100.199976	100.065613
100.050299	99.9130004	99.6663488	99.7772844
100.025505	100.12314	100.041292	99.9154543
99.8038373	99.8067498	99.8954475	99.7576528
100.077454	100.018808	100.008191	100.011664
99.8368957	99.9236254	100.158048	99.9493224
99.9005034	100.029286	99.9833154	100.088541
99.7756492	99.8926357	100.092448	100.002223
99.9601265	100.119746	99.9945497	99.9031659
99.9205746	99.9822108	100.154839	99.9584638
100.067566	100.039763	99.8717753	100.02575
99.9993833	100.140996	100.131768	100.126006
99.8962236	99.8358212	100.077402	99.9815421
99.9102438	100.008331	99.9223294	100.06831
99.947582	99.9786691	99.9634549	99.8349801
100.13265	100.26466	99.8611428	100.072207
100.059301	100.062194	100.078004	99.9896345
100.179728	100.072819	99.9714793	100.115516
99.9670628	99.884962	99.8555257	99.8591074
100.154787	100.155016	99.9983613	99.9374836
99.9753274	99.9718809	100.047712	100.074605
100.117596	100.004641	99.9249374	100.104426
99.8931243	99.942662	100.103883	99.9292413
100.120991	100.234851	100.002374	100.163021

100.047938	100.267464	99.9907381	99.9364346
100.234038	100.36973	99.7947403	99.9863376
100.045281	100.087871	99.9239343	100.076852
99.9921518	100.14321	99.9656616	100.205132
99.9658822	100.141587	99.9817105	100.149984
100.159805	100.227177	99.8402792	99.8911772
100.278165	100.269973	99.8041691	100.052276
100.262079	100.059538	99.7415782	99.9619106
100.089113	99.9909174	99.8047709	100.107274
99.8647886	99.7375394	100.084223	99.9506712
100.023144	100.019398	99.9720812	100.176958
100.02521	100.057324	99.9809081	99.9810925
100.027424	100.373567	100.173696	99.9335872
99.855491	100.09038	100.148419	99.9771962
100.118777	100.294617	99.768059	100.012263
100.012075	100.019251	100.003778	100.114167
99.9675056	99.9166896	100.123744	100.049428
100.001154	99.9512211	100.115318	99.9346363
100.1418	100.109269	100.045906	99.8965721
100.043363	100.036369	99.8906328	99.8976211
100.128517	99.9881136	99.8091844	100.050178
100.070518	99.8809776	100.028654	100.02635
100.105937	99.819736	100.332982	100.076703
99.9642588	100.000214	100.105488	99.9712018
100.160247	100.164608	99.9271441	99.9039152
100.026096	99.9557957	99.9710781	99.9299906
100.110808	100.074442	99.7502045	99.9236966
100.032146	99.7308987	99.8842132	99.9632593
99.9781315	99.7145184	99.9464029	99.8865315
100.005729	99.9929834	100.081415	99.8363288
99.9641112	100.050093	100.235685	99.8920763
99.9781315	99.909016	100.120333	100.048229
100.006172	99.8827485	99.9221288	100.154929
99.8920913	99.7958296	99.9971576	100.131851
100.04233	99.8288854	99.8240297	100.024402
100.057088	100.130666	99.820218	100.057521
100.176334	100.09097	99.8545227	99.9610114
100.053693	100.045076	99.8236284	100.094985
100.135749	99.9104917	100.077202	99.9813923
99.9604217	99.9239206	99.9478071	100.096484
100.025653	99.7968626	100.021231	100.083446
100.102395	100.116352	99.962853	100.095285
99.8587378	99.7980432	100.021231	99.8490668
99.9310529	99.9619937	99.8304492	100.073705
99.9474345	99.779892	100.159051	100.062466
99.9741407	99.9325726	99.7902144	99.8752015

100.189833	100.23413	100.004661	99.7627698
100.198838	100.314467	99.6947266	99.7957059
100.053272	100.090292	99.8048586	99.9484093
100.060506	100.081432	99.7906156	99.8996041
100.127236	100.349319	99.8885107	99.8557393
100.235451	100.422862	99.8883101	99.7230969
100.084423	100.363939	99.5262186	99.91712
99.9514052	100.196768	100.392229	100.166536
99.9801936	100.317716	100.589022	100.079555
100.136537	100.230438	99.9689532	100.281812
99.631632	99.5034214	100.077681	100.313101
100.053272	100.014681	100.308778	100.268937
99.8560342	99.9034801	100.017299	100.074165
100.051796	100.019407	99.9442788	100.311604
99.8080535	99.7264146	100.279489	100.233156
100.079551	100.047318	99.9226135	99.9870342
100.069216	100.14065	99.8062628	99.9856868
99.8386135	99.5593912	100.187813	100.019072
99.8198641	99.7063304	100.254013	99.8786448
99.7051533	99.7996627	99.8718605	99.7470504
100.046924	100.069765	99.8154906	100.067877
99.7351228	99.6757612	100.243982	99.9590386
99.8638588	99.8736492	99.8784805	99.9295459
99.9057866	99.9452729	99.8794835	99.8901724
99.9881658	99.9372983	100.0177	100.159649
99.9230596	100.080546	100.071863	99.8540925
100.04781	100.230438	99.5872024	99.7674108
99.9491907	99.9528044	100.022314	99.8358279
99.8489478	99.6394325	100.176178	99.763219
100.075565	100.046727	99.6349464	99.8993046
99.7953571	99.7284821	99.8361529	100.013682
99.9137587	99.7162248	100.037761	99.8876273
100.000715	100.050419	100.055614	99.9204136
100.154401	100.030335	99.8054604	99.7722015
100.086342	100.037424	99.6999423	100.120725
100.289632	100.167528	99.9029543	99.9746084
100.095938	99.8413078	99.8550097	100.033743
99.7919615	99.5331046	99.788409	99.8260968
100.063606	99.8683328	99.8128827	99.7686085
100.07276	99.937446	100.167752	99.620097
100.228217	100.1089	99.848791	99.9258032
100.054158	99.9634372	99.9571175	99.8317858
100.245785	100.145524	99.8694533	100.006047
100.018431	99.8811808	99.9966367	99.8286419
100.342337	100.256282	100.033949	100.074015
99.8619396	99.8640502	100.178986	99.940325

100.111882	100.028563	99.9964361	100.108149
100.001305	99.7664352	99.9147899	100.011137
100.222607	100.157929	99.9302365	99.8660692
100.018726	100.203856	100.269258	99.7460024
100.100515	100.258349	99.9145893	99.8882262
99.9890516	99.9609267	99.975172	99.8644224
100.169902	100.286704	100.122616	99.9605357
99.8967809	99.9009696	100.265648	100.115784
100.101991	99.9325726	99.8754714	100.08165
99.9778315	100.033879	99.8662436	99.9654761
100.10642	100.218181	99.8211075	99.9701171
99.9221738	99.8987544	100.075073	100.096771
100.05962	100.060904	100.088514	100.108598
99.9703022	100.155123	100.034551	100.062488
100.050319	100.079364	99.9976397	100.048116
100.06213	100.259531	100.081894	100.038235
100.165768	100.303982	100.000849	99.9216113
100.150857	100.223498	100.048995	100.096322
100.096381	100.190122	99.8016489	100.11803
100.097857	100.195439	100.014691	100.18989
99.8640064	99.7109085	100.290523	99.8439122
100.119264	99.8204853	99.9192032	100.182704
99.8712404	99.8068989	100.161133	99.8967596
100.048991	100.158962	100.036557	99.8729558
100.049286	100.029597	100.061231	99.8955619
100.098595	100.105946	99.8102749	99.9915255
99.8845274	99.7930172	100.090118	99.7909152
100.11513	99.9984368	100.225326	100.211299
99.8733073	99.7259716	99.9876095	100.147672
100.09269	99.9909053	99.9206075	100.216688
99.8423044	99.8915182	99.8684503	100.131653
100.065968	100.204447	99.8562134	99.91712
99.9221738	100.005378	100.178385	100.126413
100.155877	100.113182	99.8040561	100.155457
99.8591345	99.9727409	100.010679	100.112341
100.07084	99.9901669	100.1834	100.004101
100.060506	100.008036	100.261636	100.131653
100.00721	99.794494	100.047791	100.057248
99.8597251	99.8566663	99.7332427	100.01488
99.9674972	99.9572348	99.8688515	99.9662246
100.026698	100.043183	99.8750702	100.050212
99.8755218	99.9008219	99.7448778	100.058895
99.9837368	99.9962217	100.255617	100.17971
100.010901	100.058246	100.280492	100.049613
100.047514	100.093984	100.033949	100.142283
99.9590821	100.101811	100.088514	99.8909209

100.061244	100.275775	100.081292	99.9626316
99.7798556	100.010251	100.081292	99.9325401
100.025812	100.224384	99.8931246	99.9942203
99.950667	99.9544289	100.037961	100.024611
99.9131682	100.032255	100.13706	100.114736
99.6737074	99.7958231	99.9757738	99.982543
99.8904327	99.9285853	99.8949301	100.035241
99.8434983	100.018261	100.760871	100.103338
99.8902507	100.230105	100.424533	100.058978
100.089464	100.327976	100.121316	100.040057
100.052854	100.164436	100.152513	100.092844
99.997385	99.7609543	99.9978063	100.154217
99.9482554	100.076668	100.222815	100.093957
100.04287	100.196481	100.155078	100.112877
99.9794765	100.086771	100.14653	99.9904501
99.7796297	99.9596961	100.099733	100.04753
99.7796297	99.9516454	100.27474	100.016843
100.124964	100.415429	100.02879	100.143564
100.058242	100.222686	99.9967379	100.194761
100.045247	99.8891341	99.8510056	100.240552
99.905782	99.6441403	100.259355	100.11097
100.055865	100.105714	99.8114741	100.075672
100.0042	99.9380697	100.160633	99.8845583
100.103885	100.065618	100.279442	100.192217
100.112127	100.276988	100.075587	100.227196
99.8615653	100.171698	99.99396	100.234828
99.7667926	99.9527504	100.315768	100.174409
100.044454	100.22237	99.7606173	100.082986
99.7067277	99.923389	99.9089139	99.9675545
99.6797856	99.8531427	100.000584	100.025747
99.8450831	100.194587	99.8918192	100.181723
99.9347843	100.274147	99.8315604	100.173455
99.7243193	99.9375962	99.9448128	100.134342
99.785969	99.775635	100.087554	100.012233
100.023852	99.968694	100.220251	100.065337
100.036213	100.035625	100.005499	100.133547
100.176946	99.6836045	100.310212	99.9206505
100.290103	99.7688472	100.04097	99.9713705
100.15777	99.7630065	100.126017	100.071856
100.171399	100.177539	99.5935166	100.092367
100.178848	99.7355394	99.6772807	100.074559
99.9889854	99.8351471	99.7845499	99.9538808
99.9614095	99.8834513	99.6437323	100.172183
99.9167173	100.036415	99.7165985	100.119873
100.04699	99.9827432	99.4422285	100.015254
100.042394	100.175013	99.4676569	100.180292

99.8433398	99.9066562	99.6873238	99.9223995
99.7228929	99.8263071	100.220037	99.9758224
99.703875	99.8217292	99.7232227	99.9618307
99.8312951	99.8806098	99.8693824	100.064542
99.8040361	100.014315	100.026867	100.014459
99.9533268	100.012262	99.8072004	100.051982
99.8715497	99.9076034	100.052082	99.896642
99.6266939	99.9207055	100.132427	99.898232
99.639848	99.7895264	100.097383	100.068835
99.7545895	100.1848	99.8708782	100.188719
99.7209911	100.040677	99.8294235	100.057388
99.8420719	100.06546	99.4911621	100.01589
99.7568082	99.8477756	99.5945851	99.9879061
99.7574422	99.8971848	99.8678866	99.94768
99.7346206	99.7792657	100.035842	99.7818464
99.7140179	99.7475365	99.8708782	99.8209596
99.6953169	99.7098087	100.058493	99.9901321
99.7943686	99.9402797	100.045671	100.018433
99.8839114	100.253468	99.8807076	100.052141
99.8175071	100.067512	99.5685156	100.015095
100.096437	100.01021	99.368294	100.074241
100.013392	99.9213369	99.7512153	100.051187
99.8748779	99.9667997	99.8420309	100.071538
99.6542699	99.78558	100.025799	99.7599049
100.339074	100.029153	100.76429	100.130367
100.477746	100.403905	100.444192	100.194284
100.228294	100.336185	99.9787885	99.9181066
100.165535	100.070827	100.387993	100.03163
100.178531	100.460576	100.02238	100.113036
100.136374	100.153229	99.8168162	100.026383
100.048733	100.059146	100.047381	99.9603997
100.307536	100.245733	99.9777201	100.061204
100.102776	100.08093	100.075801	99.9972869
100.066008	100.173276	100.134991	99.9821822
100.066959	100.191114	99.73583	100.049915
100.099289	100.007527	100.114691	99.8001311
100.129877	100.23342	99.9294275	99.9004579
99.9910457	100.164279	100.261492	99.9006169
100.116881	100.362231	99.8153204	99.8783574
99.9243245	100.038309	99.8121151	99.947044
99.9588737	99.9497511	99.9702411	99.8758135
100.006102	100.181327	99.9651127	99.9158806
100.043345	99.9584333	100.163625	99.9734374
100.194538	99.9830589	100.137342	99.9247844
100.108323	99.9445419	100.233927	99.8432192
100.163475	99.8736641	100.186062	99.8804244

100.164584	99.9732718	100.316623	99.8842403
100.208326	99.9971082	100.155505	99.9085668
100.146042	99.8813991	100.051655	99.8363823
100.096912	99.9690097	99.8847676	99.7508421
99.9818537	99.8405142	100.408934	99.9103157
100.051586	99.8962377	99.9762243	99.8842403
100.191685	99.9608011	100.278159	99.9634206
100.220529	100.187641	99.9242991	99.9402071
100.107055	100.074458	99.7830541	99.9618307
100.31229	99.8155728	99.9798569	99.8785164
100.214982	99.7398015	100.524537	99.8093529
100.411342	99.9775339	100.21961	99.8449681
100.140812	99.7570079	100.070672	99.8270015
100.230989	99.795525	100.327948	99.7522731
100.226868	99.8327792	100.5021	99.8594368
100.387339	99.9139512	99.9363101	100.024718
100.422278	99.9412916	99.9049356	99.9842683
100.602372	100.012408	99.9459145	100.016119
100.398297	100.128882	100.026592	100.115173
100.384957	100.082735	100.255604	100.168045
100.471351	100.031057	100.017414	100.061824
100.008571	99.786415	100.262648	99.8829842
100.443241	100.219438	99.5941787	99.9915939
100.468016	100.050654	100.086139	100.021692
100.433236	100.136784	99.6915036	100.042714
100.369076	100.013199	99.7467825	99.9597436
100.32302	99.9792206	99.9211564	100.018507
100.891411	100.582291	99.6795514	100.141291
100.270612	100.111182	99.9273459	100.119473
100.487391	100.106125	99.6701604	100.211361
100.387498	99.9457167	99.5491445	100.145272
100.370188	99.8263984	100.159133	100.063894
100.57458	100.034534	99.7689794	100.007201
100.337313	99.6816361	99.8120927	100.030133
100.491679	100.065825	99.8684387	100.084756
100.244567	100.198893	100.079736	100.092719
100.557269	100.264636	99.6242726	100.104026
100.372252	100.270168	99.9977785	100.049721
100.261242	100.168708	99.8199897	100.185562
100.231703	100.218489	100.296156	100.159286
100.562669	100.447486	99.9973516	100.004653
100.026993	99.8319297	100.190081	100.032044
99.9969774	100.039749	99.8925565	99.9387223
100.203911	100.146898	99.6861678	100.057683
99.8591279	99.7506985	100.157852	100.035707
99.8489639	99.7054998	100.091048	99.9871349

100.170719	99.8941965	99.9661905	100.019941
99.8958137	99.7973195	100.285912	99.980924
99.8799324	99.7758265	100.411623	99.9485959
100.084166	100.083683	99.9813442	100.013093
99.8381646	99.8874009	100.262434	100.070423
99.8127545	99.7840444	100.185385	100.023603
100.167066	100.057607	99.7849868	100.043988
99.9390107	99.7164044	99.9258519	99.9769428
99.8511873	99.5920289	100.482909	100.045102
99.7479589	99.8946706	99.8746282	99.99669
99.8213304	99.9199566	100.163829	100.032362
99.7473237	99.9869645	100.04516	100.093356
100.058438	100.150849	99.5600295	100.037299
99.8880318	100.056975	99.8840192	99.840464
99.7797215	99.6609332	100.244292	99.8942911
100.016829	99.7122954	100.238103	99.9141976
99.9644208	100.197154	100.186239	100.109918
99.761458	100.115133	100.005462	100.187155
100.097188	100.28692	99.8334359	100.021215
99.9334523	100.034376	100.203313	100.056887
99.840388	99.8466272	100.072266	99.9535328
99.7603463	99.8496299	99.7290676	99.8664221
99.7841682	99.8039571	100.32625	99.791096
99.7478001	99.8730195	99.9745144	99.8291572
99.9911014	100.244091	99.7228781	99.8696071
99.7101615	99.7894177	100.241518	99.8202391
99.8659568	99.7636576	100.083578	100.080934
99.8241891	100.249149	99.6693067	100.079501
99.8513461	100.128882	100.121142	99.9350596
99.8181542	99.7413743	100.26841	100.000193
99.8511873	100.007667	100.210997	99.8930171
99.9836372	100.207269	99.7719675	100.044784
99.8426114	99.8380932	100.060527	100.033636
99.9388519	99.8279788	99.9416459	99.9320338
99.788615	99.934338	99.9371638	99.9586288
99.7674929	99.7601807	100.26713	99.8635555
100.024611	99.9352862	100.120929	99.8848953
100.201846	99.9171119	100.017414	99.837279
100.165795	100.235557	100.171939	100.165178
99.9884015	100.22007	99.7591615	100.108644
99.7573289	99.8154938	100.106202	100.038414
99.9178886	99.947139	100.165109	100.150049
99.9042307	100.157487	99.7173289	100.073768
99.8448347	99.9616785	99.9932964	99.7606789
99.8667509	100.013357	100.281643	99.8689701
99.7435121	100.025842	99.8415463	100.153871

99.7706691	100.02837	100.04388	100.031885
99.6151914	99.9542507	100.348447	99.8969984
99.8122781	100.05255	100.18816	99.9799685
99.5753294	100.033427	100.389213	99.8711996
99.7813096	99.8576898	100.528157	99.8882396
99.7433533	100.067722	100.367016	99.9350596
99.9078834	100.05255	100.02339	99.8468341
99.9334523	100.187988	99.7344034	100.07966
99.9402813	99.9947083	99.8052629	100.110236
99.8702448	99.7469056	100.147821	99.7149737
100.062408	100.037694	100.237249	99.9903199
99.8380058	100.234135	100.049002	99.9993972
100.030328	100.304936	99.5995144	99.9761465
99.8175189	99.9363925	99.767272	99.9683432
99.850552	99.8142295	99.8466686	99.8995464
99.9323406	99.9885448	99.8795372	100.053861
99.6101094	100.029318	99.8492298	100.05832
99.8130722	99.976534	100.141845	99.9589473
100.180247	100.013989	100.338416	100.009112
100.043033	100.289764	99.6957723	100.108803
99.7873445	100.122561	99.5918309	99.9337855
99.8273653	99.9940761	99.7595884	100.020896
99.7191613	99.5972753	100.12341	99.9901016
99.6061245	99.7351854	100.347152	99.8834971
99.6262587	99.8164482	100.248637	99.8330588
99.6308563	99.856054	100.073191	99.8570846
100.114711	99.7642191	100.214659	99.7426837
99.8371126	99.9824452	99.8515854	99.8559709
99.556344	100.035779	99.7812787	99.8615397
99.7407223	99.8966065	99.9926263	99.8796784
99.9767835	100.102683	100.023826	99.9439594
99.9404786	100.248956	99.9900619	100.077454
99.7835272	100.199882	100.108237	100.014446
99.7391369	99.9933329	100.116358	99.8553344
99.9669543	100.113255	100.008654	99.7614588
99.6657344	100.20509	99.8432512	100.03879
99.5195635	100.193729	99.7255034	99.8841335
99.6449661	99.8210242	99.9554428	99.7733921
99.7118686	99.7795249	100.112725	99.8217619
99.7572101	100.045878	100.065497	100.014605
99.804137	99.7135679	100.0281	99.8359228
99.7840028	99.832543	100.597392	99.8578802
100.087284	99.88635	100.242653	99.9393451
100.483784	100.155858	99.9349278	100.098775
100.391516	100.030887	100.251629	100.05836
100.123906	100.333059	100.379634	100.054064

100.274833	100.464499	99.7220843	100.12646
100.470943	100.446038	99.9577935	100.271251
100.083479	100.327378	100.045623	99.8324224
100.374077	100.334163	99.8312841	100.072999
100.265162	100.1972	99.9475359	100.014127
100.258503	99.9324252	99.8855635	99.9173878
99.9954909	100.136292	100.014851	100.044836
100.226955	100.191204	100.093064	99.8941575
99.9564908	100.174636	99.8071362	99.9484145
100.176223	100.34158	99.4263259	100.075703
99.6781002	99.8746734	99.6199365	99.8984535
99.8193565	99.8314384	99.965914	99.8714046
99.5826611	99.8614189	100.146489	99.8666313
99.8314053	100.021578	99.9417661	99.9980572
99.7665638	100.04714	100.003098	99.9732358
100.133735	100.235543	100.187519	100.09957
99.5547586	100.082959	100.050539	99.9515967
99.5813928	99.8352254	99.9240291	99.9255025
99.4605878	99.6493466	100.22513	99.9781683
99.3960633	99.7913593	100.286034	99.889066
99.612466	99.832543	99.7799965	99.9095913
99.8049297	99.8756202	100.04776	99.903386
99.9985031	100.291086	100.00844	100.071089
100.057796	100.126983	99.7094761	100.058042
99.9308079	100.070966	99.8248731	100.150008
99.9686982	99.9805517	100.031733	100.114527
99.6776246	100.080592	99.9560839	99.8435602
100.029101	100.086114	100.009508	99.9522331
99.8141248	99.9870212	99.8765881	99.8591531
100.085857	100.028836	99.9379195	100.020969
99.9920031	100.125878	99.792391	99.943482
99.8394907	99.7366055	99.6137393	99.9644847
99.8060394	99.8257579	99.8340622	100.007922
100.054467	99.7811028	100.178971	99.9601887
100.004369	99.9919128	99.7819198	100.095433
99.9797957	99.6212597	100.066138	100.085409
99.9319176	100.010532	99.9706154	100.146826
99.9997714	99.9898615	100.152259	100.055974
99.9206615	99.932583	99.778928	100.044359
100.08982	99.9322674	99.9498866	100.107207
100.010869	99.8808273	100.462335	100.007763
99.9173322	99.9994868	100.15867	99.952074
99.8413931	99.8740422	100.066993	100.080636
100.091881	100.053136	99.7725171	99.9576429
100.034015	99.6274136	100.250133	99.9283665
100.125015	99.8319118	100.304839	99.8997264

99.9921616	99.8806695	100.027673	100.009036
100.034808	99.9297428	100.147344	99.9476189
100.106467	99.9215376	100.169782	99.9956705
100.002942	99.9679284	100.347365	99.9358447
100.16893	100.030887	100.170851	100.001399
100.059698	99.7834697	100.080456	99.8101468
100.029893	99.9095454	99.9806592	99.9985345
100.089345	99.8986578	100.162089	100.062497
99.9771006	99.855265	100.089004	100.137598
100.278638	99.9701375	100.189229	100.09782
100.197308	99.9370012	100.229404	100.065361
100.03132	100.073649	100.379206	99.9247069
100.291796	100.222289	99.650068	100.18008
100.215857	99.9163305	100.14713	100.115322
100.292272	100.158541	100.03558	100.086205
100.219503	99.9392103	100.101399	100.144758
100.190491	99.9108077	99.9834373	99.9972616
100.166394	100.002958	99.826369	100.016196
100.340467	100.076963	99.8699635	100.025424
100.261833	100.028363	99.9612126	100.111344
100.117564	99.9259558	100.221497	100.101798
100.275467	100.017317	99.8353444	100.211744
99.9723445	99.7626412	100.284111	99.9813505
100.244235	99.93637	99.9813003	100.051041
99.916381	99.8121878	100.022971	100.047223
100.074601	99.8929773	99.796665	99.8796784
100.133735	99.8988156	99.8453882	100.071885
100.073174	100.001065	99.7056295	100.017151
100.299247	100.162486	99.5464243	100.192173
100.397699	100.167377	99.8870593	100.144758
100.364882	100.069862	99.7690979	100.097979
100.37971	100.14061	99.9294011	100.030732
100.541147	100.140136	99.7602801	100.001472
100.326426	99.9352934	99.797649	100.091796
100.746987	100.34103	99.5093745	100.440214
100.319448	100.012998	99.7587853	100.200567
100.363217	100.272328	99.625752	100.050133
100.140884	99.8779627	99.5232544	100.023099
100.333721	100.030845	99.8072582	100.064604
100.407462	100.244058	99.7884669	99.97412
100.067936	99.8545882	100.265721	99.8491287
100.459159	100.072066	99.9744574	100.088457
100.202573	100.126712	99.8787929	100.202158
100.299149	100.293176	100.159807	100.231418
100.328963	100.241057	100.058591	100.201681
99.8685973	99.8629588	100.220879	99.9677591

100.026863	99.8853857	99.9584421	100.060628
100.0451	100.040637	99.8136643	100.117717
100.134541	99.9973621	99.7502439	99.9642606
99.7211154	99.6161051	100.391281	99.8899974
99.7114418	99.7903083	100.237535	100.016261
99.7985037	99.9713027	100.157672	99.9284807
99.8368808	99.862327	99.7049741	99.9049454
99.8035784	99.6596954	100.0477	99.8892023
99.9287002	99.7863599	100.282164	99.8352938
99.847823	99.5872029	99.9334583	99.9062176
99.9626369	99.7527196	99.9823582	100.091001
99.7699588	99.9084443	99.8264765	99.9693494
100.060324	99.9521925	99.6029036	100.030414
100.127722	99.9766725	99.5755709	99.952016
100.133589	100.091018	99.7938053	99.9528111
99.8665357	99.9814106	100.218103	100.007515
99.8403696	100.093229	99.9447758	99.8053977
100.107423	100.26759	99.6714488	100.062695
99.9591481	100.105232	100.07845	100.006561
99.6979623	99.9790416	100.591792	99.786474
99.9228326	100.13524	100.38701	99.9065357
100.229214	100.379251	100.038732	100.071123
100.018458	99.9381362	100.235399	99.9256183
100.128039	99.9935717	99.8920322	100.03757
100.114083	99.884596	100.016097	99.9475633
99.8730376	99.8684865	100.204436	99.9030372
99.8654257	99.9926241	100.006701	99.8403825
99.9018997	99.9776202	100.170698	99.8330675
99.7247628	99.9120768	99.905485	99.8025353
99.5983723	99.5246603	100.157458	99.6872443
99.6656114	99.65322	100.401958	99.7510121
99.7109661	99.9086022	100.154469	99.836725
99.461674	99.6487978	100.370141	99.7090303
99.8249871	99.8680127	99.9200055	100.054744
99.7934291	99.9465068	99.9268387	99.9512208
99.8291102	99.8586945	100.148063	99.9213247
99.8176923	99.8392684	99.9003602	100.083209
99.8151549	99.6375844	99.9680513	99.9062176
99.9810325	99.7568259	100.245649	100.134573
100.113132	99.8541144	99.8990789	100.31236
100.130735	100.072066	99.6644021	100.146977
99.9475715	99.7599846	100.070122	100.129166
99.7713861	99.5969949	100.327861	100.059356
100.178944	99.8528509	99.8442	100.067943
100.18053	100.042374	99.6923754	100.146977
99.9261629	99.9146038	100.063075	99.9311841

99.8907989	99.9847273	100.000295	99.95949
100.06524	100.272802	99.6784955	100.146659
99.9810325	100.089755	99.7895346	100.168445
100.125343	100.231423	100.058377	100.064285
99.8977765	100.06559	100.09959	99.9690313
100.063971	100.13066	100.092116	100.156041
100.366864	100.31813	99.8576528	100.173057
100.207647	100.367406	99.9808635	99.9765053
100.158169	100.339451	99.8262629	100.045839
100.104727	100.08628	99.7382858	100.097521
99.8573379	99.8782785	100.03681	99.8208228
99.8343434	99.6304773	100.445519	99.9268905
100.331183	100.181199	100.555277	100.284372
100.681334	100.555191	100.076741	100.297412
100.422051	99.9619845	99.9099693	100.036139
100.200987	99.6636438	100.183296	99.7694587
100.250782	100.054535	99.8858397	99.9990865
100.109802	99.8553779	100.243941	99.9245051
100.088393	99.8847539	99.9806499	99.9602851
100.076816	99.9495076	99.9842801	99.9925666
100.050967	100.117077	100.107277	100.022622
100.150399	100.149612	99.7910294	100.04234
99.8695488	99.8237907	99.8542362	99.9175082
100.021471	99.9733559	99.8952353	100.015625
99.8795395	99.9010213	100.006915	99.8480156
100.122964	100.113445	100.015243	100.066512
100.199401	100.196361	99.7389264	100.143637
99.9347263	99.9839376	100.099163	99.9486765
99.9738963	99.995151	100.223014	99.9086029
99.8490916	99.9649853	99.9283335	99.8996977
99.660061	100.002574	99.9466976	99.8281378
99.9821426	100.424894	99.8574393	100.173693
99.9564522	100.362036	99.9738168	100.132029
99.9440827	100.392202	99.7034793	100.052518
99.7805839	100.163195	99.8017062	100.070169
99.7602853	100.175198	100.248211	99.9402483
100.042721	100.446374	99.9992276	100.124237
99.882394	100.269012	99.8572257	100.077166
100.21177	100.320183	99.678282	100.143797
99.8824442	99.6342589	100.314124	100.403795
99.6936376	99.4895762	100.019831	100.198586
100.087594	99.8135896	99.9129126	100.208298
99.9571574	99.7318419	100.197437	100.186931
99.6716905	99.5928437	100.14309	100.17708
100.055374	99.6718845	100.086789	100.110897
99.728815	99.4596652	100.149839	100.218011

99.8159805	99.5937911	100.227808	100.086477
99.8503797	99.6617337	100.450526	100.242708
99.8243858	99.5617145	100.220171	99.9903245
99.8250084	99.5150209	100.075777	100.068995
99.7507621	99.4431532	100.400263	100.02959
99.851625	99.5829635	99.8020866	100.101462
99.9834627	99.5527818	100.116271	100.018213
99.9408139	99.5408716	100.087677	100.245622
100.204489	99.6574027	100.051445	100.21038
99.7762891	99.4918771	100.213244	100.123107
99.6945715	99.3488185	100.03031	100.02071
100.030625	99.5801213	100.167422	100.214265
99.8920946	99.5094718	100.44946	99.9932382
99.9269608	99.567805	99.9566037	100.104514
100.140828	99.6569967	100.178078	100.124356
99.965407	99.5301794	100.297962	100.119222
100.151101	99.6407554	100.079507	100.17819
100.086505	99.6104384	100.347159	100.266711
99.818471	99.3092981	100.369537	100.0196
99.2903416	99.0892288	100.08377	100.169449
99.6749592	99.4419352	100.287128	100.035834
99.4262263	99.5596844	100.406835	100.368275
99.2543858	99.7942354	100.078086	100.165703
99.2486267	99.6529364	99.7921406	99.8661446
99.5348719	99.9352638	99.8855613	100.136843
99.6407157	100.103902	100.246634	99.9069367
99.8197162	100.282827	100.107569	100.067052
99.7398664	99.9202407	100.224256	99.8887606
99.895986	99.8693513	99.905986	100.071909
100.057398	100.016876	100.313592	100.074684
99.7289707	99.9990108	100.510201	99.9788084
99.8156692	99.9781679	99.8546579	100.177219
99.829211	99.8359214	100.024094	100.017103
99.6377583	99.7916639	100.082881	100.208576
99.7020428	99.9720774	99.8651367	99.8895931
99.7987031	100.073044	99.5909131	99.8721108
99.914353	100.097541	99.6688821	99.9678473
99.7378429	99.7528202	100.229229	99.7201813
99.8450876	99.7954535	99.9102486	99.8991668
99.8646998	99.9563775	100.027646	99.8353425
99.7599456	100.019448	99.8168278	99.8698908
100.15219	100.203786	100.125862	100.04624
99.8147353	99.9621973	100.028356	99.7652745
99.8986321	100.00253	99.9406192	99.947035
100.330879	100.210147	99.9860863	100.134345
100.05086	100.080082	100.249121	99.9337151

99.7901422	100.028787	100.100464	99.9758947
99.7653934	100.09294	99.8598085	99.9822771
99.8720155	100.140175	99.9892832	99.8134202
100.253209	100.188357	99.942928	100.041106
100.203556	100.301911	99.910071	100.090917
99.9213573	100.211907	100.02587	99.8654509
99.8762181	99.9252484	99.5722645	100.022653
100.086505	99.9917023	99.6839786	100.14822
99.925093	100.08333	99.9230362	99.8170277
100.170402	100.207034	100.028534	100.186792
100.382245	100.250886	100.004913	100.300289
100.103627	100.068172	100.047893	99.7316974
100.165732	100.261037	99.76177	99.9573024
99.891472	100.052742	99.9180632	100.027509
99.8892929	99.8724642	100.008997	99.8927843
99.9047025	99.9922436	99.8443568	100.02848
99.9075043	100.121497	99.9450593	100.011969
100.256322	100.388801	99.7797082	99.9291364
100.421469	100.597908	100.022318	100.194978
100.222856	100.571786	100.028889	100.186792
100.017862	100.378651	99.8985266	99.9166491
100.348779	100.370259	99.7438318	99.9958744
100.196707	100.169003	100.021785	99.7482085
100.186278	100.138415	99.9113142	99.8120327
100.142073	100.266044	100.158186	99.9782534
100.323719	100.376485	100.057484	99.701589
100.527002	100.509122	99.8088356	99.9380163
100.279047	100.28215	99.910959	99.8038466
100.056153	100.160882	99.7129284	99.6566345
100.330568	100.425209	99.5523727	99.9767272
100.344732	100.334258	99.8228665	99.7366923
100.258968	100.289594	99.7276697	99.7937179
100.422403	100.489091	99.7496928	100.009611
100.415554	100.383252	99.7845036	99.8425574
100.273599	100.308542	99.7411677	99.9942095
100.384424	100.402065	99.7797082	99.9951807
100.359986	100.278767	100.293167	99.809119
100.536963	100.385418	99.8889358	99.8648959
100.653859	100.547018	99.9610438	100.109648
100.24994	100.17198	100.150372	99.8633697
99.9104617	99.9524525	99.6161332	99.8980568
100.272821	100.50831	99.5601873	100.154048
100.143007	100.354153	100.052511	99.8019041
99.8567615	100.040832	100.004557	99.948145
100.086505	100.292978	99.9106038	100.072464
100.286207	100.275112	99.8352989	99.9413463

100.403258	100.349416	100.033685	100.042771
99.8963824	100.37501	100.138392	99.9131501
99.6478593	100.194078	99.9467614	99.5830907
99.9253742	100.175714	99.5436459	99.5912592
99.809097	100.15033	99.5069506	99.7396751
99.8224301	100.084438	99.3529017	99.8190057
99.9352965	100.256729	99.2415751	99.69108
99.9070799	100.082413	99.4670645	99.7223692
99.8683209	99.9769588	99.7723262	99.5720149
100.33312	100.315195	99.4103377	99.6919107
100.09886	100.14844	99.5879637	99.7706874
99.6481694	99.8696145	99.6843995	99.766534
100.017621	99.9688573	99.8444757	99.5074983
100.236067	100.224998	99.8595438	99.7701336
99.9185526	100.171394	99.6595814	99.846003
100.143355	100.325456	99.6211135	99.6743278
100.179479	100.492751	99.4917053	99.6902493
99.9506451	100.398505	99.6709268	99.7370446
99.7258425	100.264155	99.5766184	99.3984015
99.7501832	100.086598	99.2750795	99.7146161
99.8588636	100.215952	99.6523133	99.5728456
100.110643	100.328697	99.7531808	99.5933359
100.206765	100.277523	100.011465	99.6819424
98.7461683	99.0625745	100.609934	100.295958
96.1156679	97.5201923	100.091415	99.6739124
99.95018	100.603876	99.8776254	99.7094935
99.9822725	99.9568401	100.48478	99.4568264
98.8768639	98.9568505	99.6212908	100.44119
102.565487	101.392958	99.9721112	100.867886
99.0377915	98.5960656	99.7262355	97.8403109
101.145355	100.234585	100.217632	100.564824
98.5469467	98.3122446	99.9825702	99.7216769
99.0562409	98.9823701	100.231459	99.6834653
99.4539089	99.3149348	100.140874	99.5905669
99.8673906	99.7873847	100.122969	99.6779274
97.7751761	97.3187361	100.337468	99.863032
97.7503703	98.019377	100.305559	99.3567287
101.31853	100.948728	100.30485	100.516367
100.435599	100.437662	100.41724	100.765157
100.359011	100.583488	100.526971	100.355629
99.7894074	100.081603	100.491162	100.050352
99.9832027	100.122515	99.9573976	100.164848
99.5294116	99.7202776	100.130946	99.9621606
99.6534406	99.889193	100.345268	99.7910392
99.8396391	100.342064	99.88188	100.172878
99.6633629	99.761325	100.81681	99.8000383

99.9551412	100.030158	100.238728	99.9866658
99.6410377	99.7753675	100.002602	99.8748001
99.7463073	100.002478	100.016961	100.002726
100.070643	100.09254	100.050111	100.006049
100.530481	100.54028	100.139456	100.131067
100.364747	100.220272	100.262837	100.163464
99.5016601	99.5315137	99.9721112	99.9859736
99.5232101	99.7520083	100.049933	100.084271
99.8342129	100.266046	99.8127441	100.15308
100.293275	100.421594	99.9416205	100.053951
100.179169	100.286029	99.9522568	99.8408804
101.179463	101.195823	100.417772	100.842688
101.855886	101.469517	100.986104	100.168448
100.095759	100.086328	99.2101981	99.9116272
101.103495	100.710141	99.5328323	100.53215
100.678075	100.488836	100.182887	100.374043
101.492171	100.912272	100.038588	100.460434
101.270004	100.579842	99.8852481	100.221889
100.492807	99.8688043	100.199551	100.008679
100.114673	99.8648886	99.9651976	100.143112
100.50769	100.098076	100.381608	100.24404
100.837763	100.668418	100.043197	100.288897
100.387847	100.178145	100.245464	100.197937
100.202114	99.9752034	99.8528074	100.007018
99.7783998	99.7528185	99.896239	100.179108
100.201959	100.060269	99.9896611	100.1852
100.132968	99.8943239	99.7168402	100.211228
100.33498	100.034614	100.214087	100.114453
100.544434	100.271852	99.7411264	100.182431
100.413894	100.044471	100.104356	100.099501
100.24754	99.933886	100.102583	100.140897
100.277927	100.115359	99.9882429	100.175509
100.182734	100.048657	99.8671664	100.183539
100.260098	99.9965373	99.9363023	100.030554
100.454358	100.230939	99.9490659	100.205136
100.293895	100.257404	100.162324	100.18963
99.7332843	99.9029655	100.056492	100.099778
99.7211914	99.6288662	100.448794	100.051875
99.8911112	99.989381	99.7827852	100.27256
100.236687	100.038125	100.009515	100.350922
100.322422	100.111443	100.032206	100.338738
100.154208	100.019356	100.037702	100.131205
99.9174674	99.889328	99.9604113	100.135359
99.5590235	99.8146596	100.00012	99.914673
99.2796481	99.3951392	99.9664385	99.8606784
99.5692559	99.6676182	100.013415	99.9376554

99.5604188	99.8638084	99.9595249	99.9751747
99.8235154	100.019356	100.412986	99.9573149
99.9627379	100.137503	99.8572392	100.115422
99.9559164	100.128726	99.9547386	99.8933521
100.030799	100.276848	99.8625574	100.105316
100.195447	100.212036	100.050465	100.164156
99.7762293	99.9826298	99.7540672	100.013248
99.9348314	100.173149	99.8591892	100.069319
100.19376	100.564241	100.281314	100.269383
100.300316	100.493382	100.368282	100.001686
100.407956	100.712179	100.316989	100.199483
100.445281	100.721239	100.226826	100.053315
100.271044	100.59345	100.222921	100.058298
100.710432	101.010219	99.9563365	99.9258336
100.213894	100.652815	99.8677709	99.942582
100.139708	100.294463	100.204107	99.964867
100.004809	100.056599	99.9575789	100.098439
100.244095	100.292705	100.131515	100.123215
100.274761	100.275396	100.2964	100.03712
100.21343	100.285132	100.119624	99.9183591
99.9735241	100.201292	99.9717778	100.081137
99.9041389	100.046322	100.278297	99.8725433
99.9185425	100.062549	100.189553	100.002239
99.8556622	100.385877	100.472821	100.276719
98.5814837	98.3320493	99.5520229	99.1401822
99.0109595	99.4432094	98.6651242	99.8605011
97.765898	98.6871554	100.39881	99.1543007
99.1182897	99.3919583	100.159736	100.154636
99.866039	99.9346242	100.07898	99.7781433
99.9118828	100.127864	100.323733	99.8533034
100.025873	100.360319	100.001241	99.958223
100.234183	100.596966	100.053421	100.020234
100.057778	100.321509	100.055196	100.089995
99.9397607	100.369244	99.930956	99.9753867
100.270115	100.452408	99.8081355	100.081552
100.390919	100.721104	99.9130299	100.191178
100.071562	100.350177	100.534942	99.9328928
100.136765	100.121778	100.147312	99.9297092
100.076053	99.9401685	100.270665	100.065219
100.077912	100.277019	99.9066404	100.040027
100.044768	100.23307	100.118204	100.081137
99.5308837	99.5760022	100.43271	99.8185609
99.973679	100.166809	99.8461176	100.115326
99.9073913	99.8340153	100.118736	99.8766958
99.9725948	99.6524055	100.188311	100.062174
99.9733692	99.8030483	100.138615	100.024248

99.9908704	99.699194	100.249721	99.9896435
100.196393	100.010216	100.252206	100.163633
99.9019706	100.063901	100.134533	100.05899
99.6021275	99.5695113	100.060876	99.9805081
99.7433759	99.3634254	100.200558	99.9594688
100.083023	99.8468619	99.7264919	100.007638
99.8008355	99.7109588	100.043127	99.8783568
99.8392451	99.4699843	99.6652591	99.9227884
100.218695	99.9758684	99.7224097	100.121139
99.8971694	99.7918245	100.00337	100.143147
99.6818895	99.4154878	100.036738	100.07477
99.7641295	99.4637639	99.9098351	100.040996
99.5841617	99.5935817	99.4018696	100.162526
98.9933034	98.8709288	100.452765	98.3012405
99.9425485	99.7059554	100.071348	100.038089
99.9727497	99.7388155	100.111637	100.23907
100.179047	99.8588971	99.8936839	100.071724
100.593344	100.397912	99.8727405	100.16211
100.274761	100.289189	100.142697	100.273951
99.8558171	99.895814	99.5394214	100.082659
100.139708	100.046186	99.2598806	99.9867368
100.539757	100.524619	100.021829	100.141902
100.110591	100.518129	99.7976638	100.085566
100.003106	99.8841845	100.214934	99.7085199
100.075588	99.9612639	99.8555243	100.073524
100.293656	100.299331	99.6450257	100.385238
100.082403	100.090676	99.924389	100.10799
99.6453383	99.7151508	99.8803724	99.9990559
99.6030567	99.6486191	99.6258572	100.125015
100.025253	99.8601141	99.724717	100.215262
99.5291801	99.4952717	100.018279	99.9122688
100.140172	99.8603846	99.6677439	100.130967
100.127937	99.6718782	99.7321714	100.229519
99.9978399	99.6689032	99.8132826	100.081829
99.8895804	99.7344882	99.8739829	100.082521
100.555399	100.26566	100.279539	100.0774
100.463712	100.008458	100.04082	100.331809
100.104705	99.7454416	100.309889	100.072001
100.197013	100.140575	99.7511624	99.7987673
100.272593	100.357614	100.022539	99.950887
100.202743	100.350853	100.380884	100.038505
100.285448	100.108256	100.194523	99.7750981
100.664278	100.740171	99.7925167	100.170277
100.684103	100.701226	100.012955	100.230073
100.071252	99.9946649	100.212449	99.9856295
99.9371278	99.8557868	99.8313862	99.893721

100.319211	100.094733	99.9810071	100.114772
100.202123	100.139223	100.366685	100.111588
99.8705304	99.9121765	100.158138	99.8162078
100.074969	100.226038	99.3711644	99.9917198
100.412757	100.293381	99.8496673	100.099961
99.9910252	99.9630218	100.246349	100.020234
99.849622	99.903522	100.011357	99.9173902
100.271199	100.393449	100.070283	100.061758
100.174555	100.327594	99.8150575	100.031722
100.038882	100.115964	100.054309	99.9227884
100.301865	100.359372	99.9501245	100.002516
100.442029	100.529217	99.9989332	100.136919
100.051582	99.9764093	100.350178	100.005008
100.095103	100.142468	99.8830347	99.8724049
100.521791	100.659982	100.07756	100.163218
99.9217949	99.8501073	100.495362	100.003762
100.02324	99.8872947	99.8131051	99.9446582
101.022406	100.455453	100.291299	100.235081
100.884983	100.180692	100.140762	100.116277
100.98419	100.524821	99.6935893	100.142092
101.193991	100.586602	100.466333	100.131683
100.933807	100.172157	100.48302	100.063537
100.971711	100.578337	99.8789201	100.235498
100.764094	100.377821	100.107388	99.8120511
101.306768	100.354247	100.758531	100.663385
100.251369	100.447731	99.1422124	100.863658
99.08912	99.1927439	99.6481442	99.5812444
98.5519053	98.5558343	100.336211	99.8176027
97.8292237	98.3211763	99.5689703	99.4302415
98.5478497	98.8434666	99.7610469	99.5312802
99.1398153	99.0630859	100.317749	99.651333
99.4759645	99.4840342	100.14236	99.8702039
100.011307	99.9281503	100.451244	99.8561861
99.5715837	99.8178665	100.173248	100.035502
99.2822303	99.4677762	99.9376792	99.800948
98.8743277	99.0729762	99.9277381	99.6839485
98.7576504	99.0754149	99.8783875	99.6657671
99.6637712	99.5704729	100.054132	99.8457769
99.3581953	99.3485504	100.204847	99.7411297
99.2967369	99.5433762	100.496157	99.9416804
99.0268817	99.4099246	100.398521	99.7336351
99.2000258	99.4863375	99.9643072	99.9416804
99.8704522	99.8545826	100.084488	100.133349
100.202078	100.050086	100.122832	100.142231
100.140931	99.959989	99.8941868	100.378589
99.6124519	99.7540536	99.7770236	100.165131

99.4248012	99.7249245	100.106678	100.149448
99.6395934	100.125957	100.306743	100.046883
100.013959	100.065666	100.060523	100.068395
99.7581426	99.6861761	100.236445	100.018986
99.4497589	99.6944407	100.328401	100.041192
99.3806572	99.7291245	99.8127052	99.9069831
99.9763665	99.9216471	99.8238889	100.023427
100.013023	99.9007826	100.190645	100.063399
99.8447146	99.4721117	100.086618	99.9591679
99.948913	99.9144664	100.310471	100.206907
99.65566	99.5952665	100.178574	100.079637
99.5316514	99.6502729	100.03638	99.8993496
100.06949	99.7360342	100.27834	99.8253749
100.482228	100.093034	99.628617	100.155277
100.720262	100.325524	99.8861984	100.283102
100.400024	100.135441	99.8462564	100.099762
100.538071	100.134086	100.307986	100.179288
100.28943	99.8091955	99.4667188	100.084495
99.8743519	99.7881955	99.9291582	99.8636808
100.548366	100.237054	100.207332	100.095181
100.483476	100.16335	99.8966721	100.118914
100.716675	100.332163	100.105258	100.074779
100.680486	100.359802	99.9248977	100.197053
100.233587	100.20115	99.8966721	100.181647
99.7564267	99.7503955	99.8119951	99.9797087
99.8598452	100.030712	99.8222912	100.06673
99.8674885	100.023666	99.8682689	99.9015703
100.200986	100.338531	100.133839	100.062705
100.35666	100.308995	99.8872635	100.124466
100.554137	100.46575	99.8040067	99.9711038
100.185855	100.082195	100.011883	99.9565309
100.060131	100.028544	100.10295	99.748902
100.138124	100.112679	99.7356615	99.8432787
100.290054	100.32986	100.000699	99.8796416
100.178524	99.9800406	99.731046	99.8453606
100.305184	100.200337	99.9334187	99.8434175
100.368047	100.417383	100.091056	99.9747123
100.490495	100.676428	99.8324099	99.9826233
100.48722	100.458163	99.8844232	100.059929
100.097099	100.239492	100.120347	99.8924102
100.783904	100.657189	100.044369	100.105729
100.650069	100.85906	100.273724	100.225505
100.448535	100.754737	100.49953	100.101427
100.582839	100.776279	100.015433	100.437159
100.215649	100.222286	100.036203	100.176096
99.9482891	99.9480664	99.9602242	100.194693

99.9064849	99.9919632	99.7148917	100.059929
99.7052634	100.064176	99.6713993	100.114473
99.9025853	100.253312	100.035138	100.09532
100.081969	100.278376	99.7686802	99.9974737
100.042816	100.306828	99.7564313	99.8030298
100.087272	100.09046	99.7459577	99.9829009
100.253397	100.323899	99.9556087	99.9948367
100.448691	100.403292	99.9431823	100.159996
100.096943	100.172157	100.004604	99.9033745
100.486752	100.28935	99.7985036	100.227031
100.341997	100.474963	100.23325	100.076445
100.09086	99.9242213	100.506275	99.8413357
100.352604	100.339886	99.7463127	99.9905343
100.254333	100.268621	99.9158442	99.8904671
100.493303	99.9836987	100.421776	99.9877585
100.413282	100.17026	99.6254216	100.113224
100.16121	100.121486	100.083246	99.8833889
100.069334	100.091273	99.8991574	99.9509793
100.146703	100.210634	99.9619994	100.002331
100.271335	100.399905	100.289879	99.8610438
100.299413	100.431473	99.6571977	100.065342
100.193499	100.204673	99.8989798	99.9550042
100.182892	100.210092	99.895962	99.9339082
100.134692	100.216324	99.8609906	99.7927594
100.258389	100.342324	100.008687	100.034531
99.5276929	99.7110308	100.542997	100.161458
99.4942056	99.6955243	100.504207	99.991857
99.402413	99.6058559	100.617246	99.8759607
99.3951035	99.3848875	100.649135	100.046179
99.2608143	99.325221	100.764553	100.214699
99.4030929	99.643611	101.745255	100.365009
99.6376741	100.050152	100.231723	100.244483
99.6113262	99.6601289	100.397831	99.9798198
99.6682716	99.9062112	100.070137	100.226736
99.4857063	99.7833386	100.390216	99.7577495
99.4438896	99.5949002	100.668411	99.9940175
99.4709175	99.8008677	100.52848	100.291397
99.4190716	99.7858668	101.128895	99.9611468
99.3682457	99.6463078	100.591306	100.12226
99.3684157	99.7878894	100.835232	99.8588308
99.3548168	99.9239089	101.281914	100.190933
99.2956615	99.9925085	100.488738	99.9768877
99.5868482	99.570292	100.04158	100.030592
99.5657698	99.8209251	99.9528148	99.9736469
99.5368722	99.8333978	100.659843	99.9318255
99.5222533	99.8686246	100.412586	100.103741

99.3944236	99.6967041	100.524435	99.6210195
99.6036768	99.7047945	101.155073	99.7693237
99.5431617	99.6700733	100.766695	100.091395
99.5973873	99.858006	101.111761	100.114698
99.8078304	100.054198	100.866407	100.150192
99.8719153	100.111842	100.714816	100.075191
100.400403	100.319157	100.761697	99.9781223
100.332748	100.355901	100.514678	100.133834
99.9480692	100.277189	100.545377	100.262693
100.131654	100.292864	100.537523	99.6683966
100.326969	100.284099	100.623671	99.8991091
100.230586	100.286965	100.613676	100.046796
99.9050626	100.006667	101.030611	99.9342946
100.31031	100.212971	101.068687	100.095562
100.43219	100.391297	101.009669	100.333836
99.9883559	100.063974	100.324772	100.189853
99.9144119	99.8371059	100.849273	99.6208652
100.19234	99.8514326	100.800488	100.205748
99.9179816	99.9326734	100.476125	100.169328
100.106666	100.077963	100.18032	99.924881
100.068589	99.8896933	100.817622	99.8506517
100.31133	100.194431	100.800488	100.282447
99.9679576	100.199993	100.421391	99.7912375
99.9045527	99.8709843	100.478267	99.4329002
100.158172	99.866602	100.31216	100.017938
99.9696574	99.7447407	99.8609558	100.20544
99.8941835	99.5825961	100.412348	100.001425
99.8826244	99.4686566	100.988489	100.14834
99.8535568	100.001273	100.587975	100.133988
100.162252	100.019982	100.305972	99.658057
100.051081	99.7113679	100.050623	99.8319787
100.425561	100.225107	100.260281	100.152816
100.507834	100.387589	100.118447	99.9577517
100.241636	99.9210435	98.6470369	99.9637703
99.8841543	99.7649667	99.4749577	99.5065121
100.630054	100.176733	99.5006591	100.082599
100.333088	100.203195	99.2088998	100.23507
100.246395	100.056557	99.3405009	99.7018846
100.382554	99.963518	100.336433	99.8771952
100.423351	100.271121	99.9135486	100.450504
100.303681	100.125157	99.5863307	99.9876903
100.328839	100.011892	99.7374459	99.9225661
100.495255	100.444221	99.2077099	100.201427
100.143553	100.101897	99.5070845	100.057753
99.8861941	99.7108623	99.9335387	99.8805903
100.177551	100.301797	99.7902767	100.222415

99.6650419	99.9014918	100.021114	99.8648494
100.050911	99.7469319	99.3707239	99.9034301
100.236706	100.17134	99.9323488	100.268558
99.7153578	99.8689617	99.6586756	100.023493
99.9218913	99.8863223	99.9361564	99.8318244
100.06111	100.018297	99.6867569	99.866547
99.8474372	99.9950368	99.3293159	99.8011141
100.118226	100.179936	99.6194095	99.623643
100.152053	100.22005	100.004694	100.114698
100.055331	100.034646	99.5677686	100.142167
100.253365	100.044927	99.4880464	99.692008
100.30742	99.8910417	99.7541043	99.9721037
99.9322604	99.8485672	99.5318341	99.9680913
99.962008	99.6287786	99.0568327	99.7974104
100.092898	99.6505215	99.7588638	100.033524
100.204919	100.006667	99.0925292	100.061611
100.082698	99.9072225	99.1836742	99.9321341
100.05737	99.973631	99.2614926	99.8866089
100.407372	100.116898	99.3819088	99.83167
100.215118	100.087739	99.2355531	100.176427
100.202199	100.158698	98.8983402	99.5804327
100.342268	100.06785	99.1786767	100.011456
99.9917557	100.134427	99.1622563	100.196026
100.032042	100.095998	98.7303287	99.9609925
100.348387	100.012397	99.1051419	100.323959
100.270023	100.328428	99.2146112	100.314391
100.095787	100.319495	98.717002	99.9886162
99.9990651	100.037848	99.1594006	99.9009609
100.344307	100.499505	98.9185682	100.254823
99.8834744	100.194262	98.6087226	100.054049
99.8897638	99.9043571	99.2495938	99.8329046
100.4412	100.396859	99.2914777	100.379207
99.8946935	100.143697	99.1391726	99.9261155
100.044298	100.113909	100.13379	99.6829026
100.428772	100.316233	99.876298	100.118668
100.340021	100.495816	99.4657994	100.114968
100.374261	100.353969	99.7910276	99.8713335
100.326393	100.154003	99.6312959	100.015047
99.9952376	100.18281	99.9795832	100.248195
99.9083603	100.166132	100.149884	99.8463533
100.376816	100.405688	99.8457928	100.25498
100.07002	100.671355	99.8839843	100.30417
100.446999	100.721052	99.3257639	100.216739
99.0101174	100.484192	101.947767	100.516347
99.3170838	101.683821	100.36366	99.8226067
99.3228756	100.98335	99.2695575	100.224295

99.8105808	100.757103	99.4761279	100.105407
99.7579434	100.603464	99.4268873	99.8756511
100.540861	100.995647	99.6802963	99.9968514
100.18739	100.654172	100.365101	99.9761887
99.9109155	100.455721	100.050442	99.7936173
100.646988	100.873848	100.028343	100.156293
100.465738	100.97341	99.562359	100.188829
100.263535	100.594535	99.3831712	99.6841362
100.746811	100.94494	100.19432	100.204866
100.968604	101.184158	99.5594767	100.369858
100.643921	100.84184	98.8314362	99.9892956
100.281592	100.675566	99.0747569	99.9990101
100.012954	100.461281	98.6404787	100.262536
100.0552	100.379744	98.6813123	100.276105
99.7405679	100.057642	100.033147	99.995001
99.8395399	99.9009705	100.459739	100.110496
99.8834896	100.042143	100.360537	100.028771
99.9076789	100.00188	100.943738	100.089216
100.085692	100.076004	100.361498	100.01258
100.20732	100.301409	99.6728502	100.006257
99.8882593	100.074825	99.6036731	99.990375
99.9482217	100.060506	99.953882	99.8281579
100.042765	100.079037	99.795111	99.9305459
99.6962775	99.8810918	99.9817449	100.249429
99.7570916	99.9585851	100.335797	100.178652
99.8073442	100.010809	100.2904	100.325912
99.8971174	99.8554854	100.438121	100.183124
100.09932	100.081732	100.880086	100.350738
100.230488	100.293659	99.8371457	100.15213
100.079219	99.9181538	99.6802963	100.242027
100.117888	99.977453	99.5532315	100.117281
100.198973	99.9668398	99.5378588	100.209029
100.164052	100.002554	99.4972653	100.219977
99.9563983	99.7860783	100.029544	99.9834361
100.061503	99.8987805	100.265899	100.219823
100.135434	100.046355	100.69081	99.9080328
100.079049	99.8684571	99.9860685	99.9055657
100.107667	99.6915702	99.5498687	99.8943091
100.063377	99.606833	100.020417	100.033551
99.8378364	99.4499933	100.15733	100.119285
99.9332311	99.6299125	100.374709	100.118206
99.947881	99.558147	100.246203	100.104791
99.8039372	99.6181201	101.335982	99.8320128
100.043446	99.8354382	99.6007907	99.6415773
100.025389	99.4663343	100.604338	99.7846738
99.9914899	99.5525877	99.6778943	99.7529088

99.9546949	99.3930526	100.167898	100.013505
100.09915	99.6142454	99.6154428	100.121752
100.005118	99.5288343	100.440523	99.6458949
100.124531	99.7384031	100.278149	99.8354052
100.075812	100.180789	100.792173	99.9239153
100.366425	100.564043	101.044622	99.9442696
100.060651	100.24969	99.8580429	99.8878328
100.053496	100.208754	98.5355121	99.6620857
100.109882	99.8748587	99.9536418	100.111421
99.9708779	99.5808896	100.029304	100.016897
99.7932053	99.6250271	99.9349063	100.072563
99.8857041	99.6041376	100.232272	100.021369
99.458813	99.6157616	101.294908	99.8468159
99.5027627	99.525465	99.821773	99.8296998
99.6061637	99.3836186	100.879125	99.8614648
99.8788902	99.6241848	100.288238	99.8869076
100.002733	99.4788006	100.213296	99.8512877
99.9284613	99.4715567	99.5248881	99.8210647
99.8771867	99.4028235	99.4021469	99.9263825
99.9700261	99.5438276	98.6520082	100.104328
99.8780384	99.5340567	99.2318464	99.7717211
99.9463478	99.5503977	100.35141	100.067783
99.6806056	99.7323384	100.832767	99.9066451
99.8686693	99.6472643	99.6262517	99.8529839
99.9778621	99.6769139	100.199845	99.3754301
100.0012	99.6828101	99.8333025	99.6842904
99.9787139	99.6482751	99.8570821	99.9285413
100.094039	99.5685917	100.349248	99.883361
99.9848464	99.6610783	99.5931044	99.6787392
99.9719	99.7451417	100.280551	100.015818
100.315491	99.7656942	100.1458	99.8775015
100.011932	99.5276551	99.6545951	100.007954
99.7211483	99.4877292	99.4259265	99.9522879
99.7218297	99.6304179	100.176546	99.9535215
99.9691744	99.8396498	99.6913454	99.960152
99.9139818	99.7897845	100.913954	99.9653948
100.172399	100.027487	100.756864	99.9666284
99.9512879	99.7904584	100.053084	100.262228
99.8535084	99.6892117	99.7533165	100.14442
99.9391932	99.7358762	100.817394	99.7777348
99.8627033	99.9242594	99.8742102	99.7605924
99.615851	99.6127886	99.7825183	99.9398045
99.8300392	99.6871571	99.6416375	99.7876054
99.9656293	99.6109336	99.7803692	99.9621867
100.053074	99.6514063	99.0055249	100.230156
99.7136733	99.6849648	99.9542018	100.164861

99.8291885	99.8033473	98.8142613	100.139392
99.6287806	99.7126212	99.7975615	100.097869
99.8584501	100.107229	99.1480772	100.083205
100.134564	99.9203807	99.2631696	100.148962
100.004588	100.05529	99.2827496	99.9118653
99.8137071	99.8274623	99.303046	100.225525
99.8050307	99.8778844	99.270333	100.168566
99.8086033	99.8665858	99.7755936	99.7331163
99.9149318	99.695083	99.4157507	99.8343765
99.7868273	99.7586588	99.3842316	99.9413481
100.055456	99.9417975	99.6282657	99.9394957
100.299926	100.107567	98.9195638	100.164861
100.312685	100.333202	99.0967393	99.9677436
100.521259	100.330841	99.8400645	100.147573
100.450657	100.330672	99.2980316	100.043534
100.381416	100.448211	99.8426911	99.7761828
100.578081	100.240789	100.223308	99.9606431
100.437898	100.226961	99.3242975	99.962341
100.520239	100.387334	99.3911562	99.690513
100.650045	100.09104	99.5229633	100.139701
100.143411	99.975862	98.9494114	99.8876307
100.110066	100.132862	99.8083066	99.9771596
100.234087	100.207736	99.7400153	99.9671262
99.762159	99.9478684	99.7899205	99.973918
99.932795	100.004699	99.678171	99.6107088
100.531297	100.375192	99.3875745	100.033655
100.150556	100.169625	99.4365246	99.9631128
100.031298	100.137584	99.7791753	99.8133836
100.064472	100.049725	99.5499456	100.131828
99.9986336	99.8917126	99.4100199	100.010501
100.22388	100.1047	99.4408227	99.8657116
100.146473	100.261363	99.3921113	100.023468
99.8873715	100.042305	99.1975048	99.912174
100.085398	99.9952551	99.9019088	99.9078519
100.513264	100.437925	100.248858	100.183385
100.06175	100.133368	99.5750176	99.9824078
100.233067	99.9910392	99.8348113	100.113305
100.15651	100.153773	99.8195293	100.243894
99.8150681	99.7227393	99.7834734	99.8189405
100.171481	99.9512411	99.7930246	99.8070548
100.212822	100.202677	99.6924978	100.26782
99.9074463	100.039606	99.6946469	99.9578646
99.9390897	99.7244257	100.330759	100.166714
100.310474	100.234044	100.052818	100.134607
100.249058	100.196944	100.227845	100.332651
100.193598	100.232526	99.6091633	100.139392

100.187133	99.9782229	100.110603	99.9055365
99.9048944	99.8519145	100.184625	100.196814
99.8916246	99.9775483	100.272736	99.9549317
100.038103	99.9079017	100.460895	100.089842
100.114829	100.105374	100.77919	100.139083
100.060219	100.166421	100.408124	99.5857025
100.080294	99.8976149	99.9510977	99.837155
99.9401104	99.9664184	99.4002299	99.890718
99.6216353	99.7792324	99.9233991	99.7778807
99.9077866	99.7008166	100.470924	100.167794
99.867807	99.713633	100.716152	99.9956828
99.79057	99.8778844	100.853929	99.6915935
100.220307	100.013299	100.555452	100.056964
99.8863507	99.7343752	100.347713	99.9402675
99.7196276	99.8428082	100.566914	99.7173716
100.085738	100.023755	100.342221	100.129513
99.5081615	99.6944085	100.055206	99.9404219
99.6548098	99.8340391	100.076458	99.7474718
100.165357	100.217686	100.739791	100.229384
99.9113592	100.102339	100.517009	99.7971758
99.8256159	99.8190305	100.539454	99.9705221
99.8382052	99.9996397	99.8063964	100.345926
99.7165654	99.8724881	100.174358	99.7485523
100.047119	99.9794033	100.162896	99.883463
99.889413	99.9239221	99.4537168	99.9527707
99.6435815	99.8768726	100.650965	99.6130242
100.028065	100.114481	100.754118	100.062366
99.8444998	99.8213914	100.708033	100.073635
99.633374	99.7473602	100.980244	99.7349686
99.9989739	100.033198	100.560944	100.313819
99.8072423	99.8213914	100.584584	100.218424
99.7989062	99.9048662	100.149525	99.8654029
99.9952311	100.002169	100.691558	100.24127
99.7402128	99.8905321	100.244798	100.007414
99.8133669	99.8131282	100.0447	100.15807
99.9739654	100.052929	100.073831	100.572526
99.7558644	99.8765354	100.578853	99.8652485
99.8701888	99.7819306	100.629952	100.116084
99.9572931	100.021225	100.408602	100.252384
100.015136	99.9947492	100.416004	99.7253983
99.9562723	100.026959	100.125169	100.219196
100.042526	100.094076	100.739075	100.36661
99.8536866	100.005879	100.797815	99.954623
99.7886986	99.905878	100.570973	100.075641
99.6803286	99.8534322	100.36395	100.127352
99.7223497	100.015323	100.797099	99.5128445

99.8511347	99.8635504	100.786354	99.994911
99.7131629	100.023923	100.54423	100.08552
99.7844455	100.098292	100.263423	99.8476515
100.225516	100.167959	100.798987	99.9937381
100.034431	100.117747	100.930323	99.8606562
99.9166835	99.9952498	100.563727	99.2038821
99.960924	99.9687959	100.58804	99.734051
99.8140798	100.000136	100.579459	99.6595683
99.8367104	99.9216169	100.352541	99.4361201
100.205948	100.151783	100.095828	100.043393
99.8700609	100.156838	100.273167	99.6959615
99.9636465	100.024063	101.002785	99.7790799
100.356025	100.7058	99.8920309	100.386353
100.256314	100.871432	98.7240711	99.9698358
99.9483325	99.8328192	100.050301	99.8410717
99.9473115	99.533401	99.2923193	100.248953
99.592197	99.3280038	99.9408945	100.068067
99.6481781	99.5662578	100.487452	100.03522
99.5745008	99.5494081	101.060944	100.223817
99.4671326	99.3551317	99.5154234	100.086726
99.4147247	99.2971689	100.938904	99.8261135
99.4802346	99.3205899	100.59519	99.9311295
99.4504574	99.2014629	100.026704	99.6762228
99.7155597	99.33963	100.150651	99.9946634
99.7684781	99.6757805	99.9961939	99.9874156
99.7400621	99.4553871	100.104886	99.878236
99.7504416	99.5861404	100.029326	100.040001
99.6941201	99.6402277	100.230501	99.8597309
100.089902	100.118084	100.341576	100.124507
100.445187	101.002859	101.169636	100.248182
100.529073	101.063686	99.7895365	100.242785
100.236576	100.655251	98.5729514	100.151339
99.9896802	100.233335	98.7600633	100.222892
100.227898	99.8720789	99.2956563	100.155194
100.150988	99.817149	99.5659556	100.255276
99.8344984	99.5647413	99.908001	100.097366
99.9188956	99.5367709	100.189026	100.062669
99.8433465	99.7438531	100.672895	99.9841772
99.7524835	99.6334879	99.0110555	100.040772
99.7904281	99.7160512	99.3809889	99.8410717
99.8816315	99.8707309	99.0611109	100.04108
99.575862	99.5239652	99.5025521	99.9224938
99.8664877	99.4457828	100.344913	100.128979
99.8498124	99.7795743	99.5874079	100.116951
99.7972344	99.7179046	100.741066	99.893811
100.106407	99.9797482	101.076914	99.8999794

100.127336	100.076971	99.6169644	100.142858
100.060975	99.8230464	100.688866	99.9525645
99.9030711	99.7175676	99.5020753	100.165218
99.7810696	99.6223671	99.8898857	99.8685208
100.006355	99.8321452	99.2055565	100.116026
99.6777852	99.626074	100.038383	99.9485551
99.7422741	99.7389667	99.3669257	100.145942
99.6522618	99.7256555	100.409747	100.051721
100.169705	100.038048	99.56095	100.022267
100.190804	99.7918746	99.3171086	99.9963597
99.9571805	99.8771338	98.572713	99.9561113
99.937953	99.8343357	99.6953846	100.074081
99.9595627	99.725487	99.5385443	100.275323
99.7238973	99.7211061	99.8095586	99.8836333
100.059104	99.9570011	99.8817815	99.8820912
100.268055	100.290287	100.660262	99.6533999
99.9442487	99.9944074	99.373123	99.6677413
99.9076653	99.5445217	100.454559	99.7676685
100.231641	100.129373	99.6784611	99.7428409
100.116616	99.9593601	100.327275	99.7932671
100.275712	100.008056	99.6069534	100.050487
100.336798	100.141168	100.879076	99.9849483
100.207649	100.320111	99.4632228	99.8770023
99.8241189	99.8011419	100.076521	100.008234
99.9263824	99.8240574	100.070085	100.152419
99.9619449	99.7888416	99.9888047	100.155657
99.9842353	99.818497	100.881698	100.34117
100.269416	100.02659	100.483877	100.116334
100.158645	99.9834551	99.4524966	100.025659
100.223984	100.124655	99.6901407	99.7875614
99.8831629	99.6922932	100.356116	100.08796
99.8840137	100.22828	101.542191	99.7570281
100.287282	100.315899	100.43835	99.5915623
100.111852	100.309664	99.5814489	100.03044
100.16392	100.364763	101.14723	99.9571907
100.352622	100.457773	99.7685609	99.7618085
100.308041	100.343701	99.4148359	100.0821
100.349219	100.347407	101.136742	99.8927316
100.255633	100.312697	100.192363	99.7392941
100.096708	99.8885916	99.1984058	99.9124703
99.9786201	99.8837052	101.005168	99.7392941
100.036813	100.323818	100.645246	99.8515579
99.9813426	100.311855	99.5464101	100.047711
99.9787903	100.042597	100.421188	100.013323
99.9202568	100.233841	101.115052	99.8600394
100.104365	100.316236	99.9256396	100.00222

100.064379	100.039227	99.6267372	100.138077
100.064038	100.375041	100.279126	100.084875
100.018947	100.242434	100.723666	100.103226
99.7905983	99.9699754	99.1795754	99.9439288
100.141119	100.107805	99.6972915	99.8924232
100.353983	100.597119	99.237735	100.060665
100.217348	100.576226	98.6902241	100.329604
100.090412	100.834868	100.082242	100.200223
100.454545	100.946749	100.294143	100.421513
100.474964	101.122154	100.57636	99.990654
100.320463	101.086602	100.618073	99.9009046
99.8317317	99.7657449	99.9199008	100.063688
99.9210047	99.7299474	99.9260795	100.101031
99.8600724	99.6734921	100.249918	100.01634
99.716165	99.5853426	100.40275	99.9199539
99.9191153	99.9458642	99.815044	99.8356863
99.7092373	99.9400631	99.6892887	99.9167128
99.4075671	99.5951055	100.365496	99.7873522
99.6429517	99.7196185	100.256278	100.107935
99.6484624	99.3347602	99.9555193	100.000417
99.4812527	99.1931267	100.338782	100.098917
99.7618249	99.6774539	100.118711	100.124282
99.7703271	99.5788339	100.233381	99.8740154
99.8714086	99.6923105	100.304618	99.7259129
99.8726682	99.8195119	99.8199507	99.8652786
99.7577312	99.577985	100.298621	99.6692648
99.5654873	99.4933727	100.612827	99.7277448
99.9510773	99.9805298	100.188676	100.003517
99.9046301	99.8302653	99.8808294	99.9430641
99.5711555	99.844273	100.224112	99.8697879
99.7254544	99.8414431	100.011491	99.9592694
99.9011663	99.8647893	99.883737	99.8201855
99.8136252	99.8090415	100.424013	99.6935023
99.9814647	99.9471377	100.287172	99.7694559
99.8191359	99.6606163	99.771066	99.9915391
99.8566085	99.7555575	100.100901	99.8843022
99.9628858	100.049295	100.039841	99.7970754
99.9620986	99.8664872	99.8112278	99.9577193
99.9280899	99.7210334	100.021486	99.7115395
99.6424794	99.7439551	100.675887	100.267452
99.4072522	99.1099294	100.270998	99.7694559
99.9318686	99.0990345	100.26064	100.197135
100.101597	99.6515608	100.154874	100.041141
100.022401	99.8512061	100.29753	99.7366225
100.087585	99.8506401	100.110533	99.6333313
100.278411	100.117636	99.293123	99.8535826

100.119389	99.9864725	99.5384549	99.8917707
100.003822	99.8712979	99.8862812	99.9546191
100.111989	100.000905	99.9358928	99.9474324
100.30691	100.240875	99.6334984	100.18558
100.157019	100.091601	99.7941454	100.122309
100.067589	99.6829721	100.047292	99.9864661
99.8484212	99.0762543	99.927715	99.6195216
99.864166	99.5389332	99.8370331	99.4305536
99.8997492	99.9775585	100.060558	99.4826924
100.125215	100.219085	99.7625248	99.8959982
100.229445	100.592907	99.6434934	100.248851
100.249126	100.371897	99.7597989	100.201644
100.255581	100.293227	100.04602	99.9496871
99.9181706	100.005857	100.061285	99.8906434
100.020354	100.045192	100.175955	99.8306133
100.173394	100.13801	100.224294	99.9761792
100.298722	100.365105	99.9188104	100.1333
100.158436	100.182014	99.861748	100.024654
100.348948	100.473912	99.6338618	100.290562
100.458847	100.576919	99.5631699	100.411327
100.056095	100.019016	100.078549	99.8919116
100.027439	99.5600155	100.073824	99.7624101
100.052159	99.9029922	100.023485	99.8263858
100.101125	100.194324	100.028937	99.9454596
100.122066	100.325912	100.279903	99.7273221
100.291952	99.9120477	100.283537	100.070311
100.13844	99.8193704	100.150876	99.8246949
99.9312388	99.6652856	99.6545788	99.8028529
100.04901	99.9379407	99.9402542	99.9654697
100.024605	99.9806713	100.251008	99.859501
100.245977	100.507871	100.280993	99.958142
100.098448	100.17876	99.9611529	100.07482
100.009176	100.16164	99.9195373	99.807644
100.208032	100.45184	100.009492	99.8504824
100.04775	100.238187	99.5373646	100.033673
100.161743	100.273701	99.5838868	100.094408
100.150564	100.340344	100.032208	100.117518
100.321395	100.438822	100.051835	100.248992
100.00335	100.239036	100.128887	99.9251678
100.042554	100.409109	100.039841	100.070734
100.191973	100.502352	99.6680266	100.447683
100.16867	100.416891	99.7332667	100.428378
100.212913	100.105609	99.9133586	100.262379
99.9598943	99.5148795	99.8695623	99.9182629
99.945724	99.7361731	99.7934185	99.9068487
99.8021315	99.9646827	99.9238988	99.7974981

99.959422	100.32761	99.9667864	100.163738
100.228343	100.637336	99.5522662	100.39484
100.147257	100.385904	99.4037949	100.320014
100.146628	100.411232	99.8913695	100.262379
99.9698136	100.195173	100.01131	100.124423
99.8337786	99.8072021	100.200851	100.037478
99.9099833	100.100657	100.099993	100.198826
100.106478	100.285304	99.7138219	100.54266
100.091993	100.308508	100.13761	100.533501
99.9515496	100.231395	100.32697	100.357638
100.191185	100.462593	100.113077	100.373702
100.18347	100.668889	99.935711	100.574507
100.000516	100.505041	99.6752957	100.45952
100.064282	100.616395	100.029301	100.249697
100.139542	100.66903	99.8542972	100.318886
99.9773711	100.439954	99.8753775	99.8603465
99.9520219	99.4994569	100.313704	99.6478456
99.9342303	99.3931964	100.548132	99.7053392
99.6037472	99.250148	99.8144989	99.9005075
99.5645427	99.3162247	99.9258978	99.7938343
99.9832516	100.191765	99.6275816	100.00419
99.9351434	100.206412	99.5553241	100.001375
99.8942908	100.23855	99.6705015	100.440134
99.7543826	99.9938211	99.6500376	100.232012
99.9173197	100.084261	99.8001667	100.048094
99.8354568	100.047858	99.6860758	99.7846697
99.9591186	100.325577	99.7702858	99.8304031
100.170637	100.596328	99.463327	100.238485
100.176789	100.455549	99.9602561	100.130132
100.036407	100.208118	99.8247958	100.020935
100.151078	100.313774	99.5712606	100.146033
99.9804124	100.167022	99.5330492	100.252979
100.061329	100.231582	99.9984675	100.286329
99.9004423	100.202146	99.7902064	100.092561
100.270639	100.686768	99.6741235	100.314473
100.168587	100.632305	99.2898365	100.102974
99.9767846	100.518971	99.6594546	99.8437713
99.9159001	100.462374	99.5502534	99.8692412
99.83593	100.509159	99.5154828	99.7866398
100.12868	100.662736	99.5212779	100.124363
100.252342	100.410613	99.7393183	100.171081
100.218272	100.619791	100.029073	100.150677
100.040508	100.627328	99.961886	100.061321
99.860063	100.51186	100.13103	100.038806
99.7152651	99.7901889	99.7472865	99.9763276
99.9570681	99.5851346	99.3325753	100.093686

100.213225	100.249926	100.139723	100.192048
100.02158	100.123224	100.406478	100.138998
99.9747341	100.17072	99.641345	99.8886603
100.332312	100.379187	99.8094026	100.025016
100.086881	99.9429131	99.187698	100.039088
99.9676361	99.8246016	99.6529352	99.9963096
99.6646332	99.6310656	99.9767359	99.779041
99.8435011	99.7438312	100.013499	99.9374894
99.9379825	100.076156	99.6898789	100.129007
99.840662	99.8386795	99.8669914	99.9649294
100.098869	100.082839	99.9747438	99.9805491
99.9521784	99.8091016	99.8132057	99.9577528
99.7763073	99.8381107	100.266128	100.068639
100.073159	99.9855734	100.020018	100.042887
99.9586454	99.7886246	99.3691568	100.095234
99.9078557	99.9205875	99.8041508	99.7988822
100.168744	100.208403	99.8946991	99.9509984
100.140668	100.226036	99.6636199	99.8896453
99.5226745	98.8795317	100.044647	99.8257594
99.3059508	99.2690211	100.750018	99.5194163
99.7515434	99.871528	100.781348	99.6946102
100.10092	99.9676561	100.51242	99.8864088
99.9436609	99.9102068	100.639368	99.6815234
99.9329351	99.9709267	100.024545	100.042043
100.13499	100.078573	100.236247	100.140827
99.9539135	99.8954178	100.241861	100.071453
100.003599	100.096064	99.9137143	100.107477
99.9144805	99.9126242	100.167974	99.8973848
100.115904	99.8595831	100.098795	99.8544658
100.314173	100.155362	100.015491	100.082429
99.9092753	99.8964132	100.408289	99.9778755
100.125999	99.9690781	100.6314	99.9487469
100.129942	99.96339	100.056962	100.289988
99.778831	99.5989281	100.22176	99.9799863
99.9766268	99.7923219	100.254719	100.001094
100.282311	100.143417	99.9830743	100.039088
99.9280454	99.7866338	100.174312	99.8641754
99.8018599	99.8164961	100.228279	100.014884
100.166378	100.070752	100.082859	100.043309
99.9892454	99.7219322	100.161273	99.8616424
100.356288	100.21793	100.153124	100.156728
99.9854598	100.046293	100.194957	99.8550287
100.043663	100.1191	100.579063	99.8315288
100.326949	100.467209	99.961886	100.115498
100.036092	99.9505919	100.138636	99.981112
99.5061126	99.1140217	100.224476	99.9939174

100.135463	99.7489505	100.090827	100.25706
99.8187372	99.588974	100.22683	99.7577926
99.8163712	99.547309	100.416076	99.8560137
100.100762	99.983156	100.03197	99.9480433
99.9805701	100.024821	100.429296	99.8278701
99.8220496	99.6647673	100.187713	99.8764178
99.9102217	99.4776304	99.9254856	100.106351
99.9769423	99.6431527	99.8595664	100.032193
99.9651124	99.7542119	99.9999163	99.9826599
99.9239444	99.7887668	99.9184228	100.011085
99.8775712	99.9291195	100.165257	99.9411481
100.04177	100.091798	100.032151	99.7088227
100.228051	100.104312	99.4432253	99.9943395
100.233099	100.269692	99.9327294	99.9221512
99.8674764	100.010459	100.235885	99.8249151
99.960065	99.9773258	100.243853	100.255371
99.9184238	99.8311428	99.8097648	100.181494
99.94303	99.7808036	99.9457684	99.8422234
100.131362	100.025248	100.104409	100.006582
100.108491	99.9104912	100.119802	100.177273
100.004703	99.7830788	100.358125	100.037118
100.263857	100.017995	100.422415	100.265925
100.05849	99.6582261	99.8702511	100.285344
99.977731	99.7699963	100.192603	99.9210254
100.076944	99.8133677	100.250011	99.7929721
99.9496547	99.8594409	99.9664134	100.238344
99.8542277	99.5011517	100.049858	100.12202
100.011556	99.719333	100.239696	100.044371
100.023064	99.8375027	100.337789	99.8683551
100.041193	99.8531073	100.322921	100.005898
99.9790815	99.7238725	100.164631	99.9603792
99.6152397	99.551654	100.596709	99.8348149
99.4613794	99.1679217	100.386744	100.12188
99.902309	99.4289448	100.355376	99.9751764
99.673095	99.4070983	100.452199	99.6852932
99.7946383	99.4286611	100.737229	100.070301
100.220592	100.056251	100.232988	100.253786
100.052701	100.010856	100.055298	99.8830113
100.321641	100.197828	100.417749	100.131181
100.074141	99.8126771	100.012507	100.081857
99.6273783	99.6387563	100.478128	99.9531921
99.8565923	99.8745282	100.568967	100.025064
100.075244	100.008019	100.29246	100.215031
100.000994	99.7748004	100.227186	100.00928
99.7550697	99.7546562	100.151577	99.9427636
99.8628981	99.7167795	100.083764	99.7671708

99.6191808	99.4000053	100.208329	99.9182426
100.000048	99.8062934	100.05421	100.00308
100.028424	100.005323	100.206334	99.7959195
99.7728835	99.97227	100.436243	99.69699
99.9521244	100.058237	100.510221	100.045357
100.09495	99.9898607	99.5749905	99.9496689
99.7875444	99.6407423	100.083946	99.7061501
100.042612	100.067316	99.8148721	99.746032
100.228001	100.216412	100.054572	99.9715123
99.8796083	99.8426096	100.264718	99.8852661
100.200886	100.367635	99.9390739	99.9529102
100.225006	100.426081	100.160099	100.027037
100.219961	100.273298	100.100445	100.069596
100.226109	100.283086	100.136346	99.894849
100.109453	100.295002	100.098632	99.9902554
100.314547	100.498714	99.9958259	100.178391
100.112133	100.217405	99.876701	99.9413543
100.237775	100.307628	100.235526	100.014917
100.171092	100.132572	100.044056	100.032251
99.8997867	99.9658863	99.8359048	99.9540376
100.097472	100.194282	100.072523	100.09003
100.017231	100.214142	100.273603	100.272106
100.312971	100.316281	99.862921	100.283521
100.133415	100.343944	99.9550297	99.9887052
100.051125	100.101505	99.8600199	100.096372
99.9972105	99.9498561	99.8174105	100.141609
99.9981564	99.863605	99.9441507	100.386255
100.118596	100.145056	99.794746	100.531408
100.048129	100.141226	99.558672	100.215172
99.8685732	99.8373608	99.8170479	99.7852092
99.9923236	100.161086	99.9621011	100.065791
100.03536	100.093844	100.148313	100.1006
99.895688	99.8834654	99.7115218	100.331576
100.160529	100.033979	99.7831417	100.135127
99.8805542	100.053982	100.576764	99.9141558
99.9943729	100.042207	100.366074	100.026473
99.9740369	99.7911143	99.8444267	100.039297
100.130262	99.9013398	99.4206902	100.273515
100.146342	100.311316	99.82285	100.158238
99.8966338	100.092851	99.8712615	100.043807
100.053332	100.137537	99.9466892	100.061
100.077766	100.176265	99.6420776	100.355956
99.9306849	100.131721	99.8569375	100.167117
99.5955343	99.7460027	99.8986403	99.7374355
99.8293199	100.027737	100.04732	99.6898028
99.7716223	99.932691	99.9006348	99.8906213

99.8830765	99.8552352	99.1523419	100.222641
99.9956341	99.9863142	99.7211315	100.090312
99.843035	99.9424794	99.9546671	99.9693985
99.964263	100.102782	100.106429	99.923175
100.037883	100.222654	99.9780569	99.8940035
99.9133441	100.264219	99.8172292	99.9196519
99.9332072	99.9185049	99.6600279	99.9667209
100.116389	100.367635	99.9831338	100.008998
99.8815	100.045612	100.102984	99.7812633
99.8143438	100.26507	100.012688	99.8490483
99.7114024	100.119805	99.9427002	99.7833772
99.984599	100.386502	99.8643715	100.017031
100.077294	100.314437	100.120209	99.6871252
99.9190192	100.136828	100.468337	99.7033316
99.7966877	99.437882	100.416117	99.9940604
99.5912779	99.1090496	100.495534	99.6487936
100.126636	99.994684	100.089023	99.7816861
100.247706	100.005749	99.3572295	100.157675
100.217439	100.219249	99.7791528	100.185719
99.8963185	99.9261654	99.8873987	100.04578
99.9584302	99.7861492	99.8814152	100.08383
100.040405	99.7465702	99.5222274	100.119202
100.118123	100.013835	99.7037251	100.139072
99.77178	99.6674121	100.184395	99.9078141
99.7873867	99.4821424	99.9782382	99.7144647
99.9401435	99.6601772	99.8478717	99.908096
100.10898	99.9494305	99.6127043	99.992933
100.144135	100.370898	100.201983	99.9781358
100.106615	99.9900026	100.153209	99.8094483
100.02401	99.6674121	99.8065316	99.8155081
100.172668	99.9958189	99.4529645	100.015058
100.313917	100.265637	99.7347303	99.9622113
100.264417	100.39856	100.025924	99.9996974
100.232415	100.302805	99.398207	100.28338
100.25748	100.41374	99.748329	99.9716533
99.8712613	99.9134337	100.21446	99.8096801
100.112798	99.7740404	99.8646323	100.151691
99.9328667	99.4945454	99.6924432	100.039565
100.16306	99.9614565	100.157427	99.9308207
99.8427433	99.5888909	100.433874	99.7338969
99.937751	99.5480929	99.706429	100.000265
100.06301	99.7264427	99.9343798	99.6696642
100.076245	99.8441621	99.9449146	99.6259972
100.031656	100.202987	100.293471	99.7971435
100.157072	99.8081804	100.343238	99.9209604
99.8449491	99.4698966	100.265317	100.020127

99.7924821	99.6040485	100.201927	99.9036345
99.7680605	99.5990904	99.836479	100.159297
99.8099711	99.601357	99.7970644	99.9892781
99.7688483	99.6893278	100.13672	99.96505
99.6483161	99.3694597	99.9846925	100.152677
99.8036688	99.6461215	100.005217	100.278325
99.7680605	99.6999523	100.04445	99.9167346
99.8287206	99.6118398	100.157427	100.144648
99.9963627	99.8122886	99.7207781	100.387633
99.877091	99.8145551	100.179404	100.035903
99.9921087	99.8349542	100.316175	99.9246228
100.006289	99.8203632	99.840475	100.227615
99.8416403	99.8410455	99.7472967	100.149719
99.8295083	99.9080507	100.090767	100.130139
99.8726794	99.8268795	100.260413	100.194654
100.203867	100.105666	99.7333108	100.506098
98.7617347	98.6203622	100.578636	99.2728584
99.0508545	99.1714192	100.436053	99.6886804
99.6795127	99.4527558	99.9719781	99.7708025
99.633033	99.468055	100.277305	99.6927654
99.8573962	99.9457322	100.273128	99.880815
99.9922662	99.9713726	99.7712724	100.194372
100.23853	100.326231	100.114743	100.183244
99.9259341	100.071101	100.448223	99.9068743
99.9462592	99.9113088	100.543036	99.7948897
100.284222	100.359521	99.724774	100.047595
100.027087	100.067276	100.013209	99.9127905
99.8532997	99.8464286	99.9781537	99.8329222
100.108072	100.251718	99.8484669	100.305089
99.9221527	99.9271748	100.225903	99.8830688
99.9328667	100.051269	100.208829	99.8888441
100.281859	100.384595	100.207194	100.261703
100.019681	100.110058	100.157245	100.049426
99.9612272	100.049286	100.413349	99.9067335
100.091686	100.278775	99.8924223	100.119715
100.126664	100.183296	99.644855	100.137041
99.9440533	100.237835	100.087497	99.9146217
99.8640136	99.999138	100.419161	99.9108184
100.115792	100.143773	99.7035228	100.136478
100.116422	100.084559	99.7707275	99.9065926
100.031341	100.215453	100.00885	99.8743354
99.9804493	100.157655	100.099304	99.9018033
100.183227	100.224094	99.9935926	100.091121
100.139583	100.182729	100.11892	100.03703
100.117683	100.077759	99.8453791	100.014492
100.179131	100.36972	99.7783561	100.198034

100.022045	100.320281	99.9156715	99.9156077
99.9607545	100.191654	100.179404	99.7658723
100.02236	100.292799	100.087679	99.9643457
99.9442109	100.210636	99.7258638	99.9005356
99.8772486	99.9821388	100.072603	99.6886804
100.198983	100.237977	100.152704	100.008435
100.169047	100.165022	99.6706471	100.212402
100.14305	100.269567	99.9995865	100.011957
100.101297	100.121107	99.8061461	99.8589815
100.323612	100.392528	99.7522008	100.171693
100.103345	100.310931	99.8491934	100.063934
100.184488	100.408677	100.138718	99.9575843
100.167786	100.242226	100.070424	100.033368
100.123512	100.259934	99.758558	100.262689
99.929558	100.108216	100.033552	100.118025
99.8700009	100.035828	100.200111	99.9992792
99.9766679	99.9113088	99.9078612	99.9465972
100.000932	99.7893397	99.678639	99.8627848
100.067737	99.9376576	99.9085878	99.8771526
99.990218	99.8970012	99.9536331	99.9816716
100.014797	100.062602	99.7547437	100.101967
99.9878546	99.8557782	99.8388403	99.8317953
100.000774	100.021662	99.9563576	99.8578546
100.13722	100.214319	99.8368423	100.107742
99.9292428	99.8869433	99.9618066	100.015478
100.152503	100.269142	100.110383	100.035199
100.263109	100.34323	99.6608388	100.323401
100.296669	100.193212	99.6572061	100.259027
100.116265	100.192079	100.069334	99.9757554
100.250977	100.34238	100.079687	100.220431
100.175034	100.385586	99.8666303	100.196485
99.9730441	100.02917	99.8575486	99.9963211
100.052926	99.9638647	99.6152486	100.088585
100.164793	100.054102	99.6172466	100.191695
100.005974	100.034978	100.057891	99.7710842
100.016845	99.8339625	100.053895	99.9261723
100.096412	99.7547747	99.6315957	100.021254
100.054817	99.8751856	100.068244	99.6800879
100.140371	100.100283	100.187215	99.7874241
100.126348	100.003529	99.9065898	100.19733
100.155339	100.072659	99.9696169	100.065907
100.237112	100.250726	99.9247532	100.023648
100.128082	100.197887	100.067699	99.9082829
99.9003231	99.6805692	99.9830126	100.241129
99.8901508	99.8311746	100.019757	100.117347
99.8516108	99.8978336	100.006985	99.9129626

99.8695198	99.9473517	99.9162038	99.780999
100.003908	99.8292701	100.059253	99.7367587
100.290308	99.8367417	100.216647	99.9220531
100.436875	99.9281598	99.8653113	100.194768
100.267671	99.7875166	99.9731878	100.327943
100.085143	99.9505748	99.9612015	100.414303
99.8656514	99.9486703	99.7558629	100.477331
100.091734	99.9828056	99.748003	100.445665
100.027691	100.182636	99.8360334	100.358245
100.195606	100.010348	99.8466442	100.168557
100.097321	99.9467657	99.69377	99.8109976
99.8282576	99.8541756	99.6493619	99.827512
100.113798	99.7284758	99.8230646	99.8137248
100.111792	99.7968928	99.5825531	99.8594803
100.195033	99.8412833	99.6299087	99.9593241
100.055056	99.8370347	100.092265	99.8185731
100.27899	99.8310281	99.8943928	99.8672072
100.232713	99.7273038	99.9140424	100.031897
99.8232431	99.8946105	100.059646	100.04432
100.068667	99.8201869	100.026045	100.048714
100.18486	100.152163	100.065345	100.098712
99.9460267	100.100448	100.24337	100.248402
99.8761102	99.982659	99.9399799	100.271279
99.8980308	100.13429	99.8706167	100.124771
99.5746671	100.069096	99.5334291	100.275825
99.680688	99.8820113	100.041175	100.294309
99.4558951	100.069536	100.664265	99.9714448
99.8766833	100.431838	100.279722	99.8203912
100.212798	100.404735	100.048642	99.947355
100.29432	100.177215	100.134315	99.7956953
100.049325	100.29603	100.213699	99.5355558
99.7960215	99.8408438	100.183439	99.6096433
100.113081	99.937243	100.134118	99.6275213
99.812641	99.9496958	100.20525	99.8166034
99.6285372	99.8070016	100.502745	99.8244819
100.185863	99.6792507	100.52829	99.5619182
99.8205209	99.8005554	100.683129	99.5670695
100.036718	99.935778	100.644616	99.6240366
99.8907239	99.9896912	100.346727	99.7964529
99.8667976	99.9830986	100.290726	99.9049327
100.221108	100.175897	100.150427	99.8566016
100.187582	100.201975	99.879459	99.8403902
100.102336	100.218676	99.7397502	99.8688738
99.9258254	100.139857	99.7454486	99.8643285
100.155346	99.8672144	99.7572383	99.9325072
99.9798388	99.9212742	99.9120774	99.883267

99.8375702	99.9640531	99.9307446	99.8578137
100.167954	100.034961	99.8928208	99.8108461
100.210649	100.5675	99.827584	99.8458445
100.020528	100.356975	99.9350675	99.8243304
100.156779	100.104843	99.9800651	99.6531261
100.14016	100.159196	100.005413	99.595553
99.9827042	100.104843	99.9566821	99.798271
100.260221	100.190987	99.8804415	99.8314512
100.062363	100.280647	99.933692	99.8887213
100.038867	99.9879332	100.140013	99.984323
99.8070534	100.080816	100.038621	99.965839
100.004052	100.102792	99.8890874	99.9773536
100.073538	100.303794	100.200141	99.9552334
100.098181	100.296176	100.251623	99.9979587
100.343032	100.173699	99.9997148	99.9505367
100.214088	100.081549	100.032137	99.8144823
99.8646485	100.052688	100.050804	99.8519049
100.021817	100.061185	100.399781	99.9408401
100.091017	100.255302	100.241798	100.067046
100.297472	99.826926	99.8228681	100.047653
99.9331323	100.009762	99.7002544	99.9206896
100.082994	100.17326	99.7375887	99.8232698
100.074828	100.063382	99.5196743	99.9120536
100.044597	100.101034	99.7163671	99.9522032
100.057922	100.048879	100.152589	99.9234167
100.129701	99.958779	99.9297621	99.8256939
100.083711	100.018699	99.9238672	99.8403902
100.206064	99.9316759	99.3614948	100.61187
99.6935824	99.8937315	99.5194778	100.488694
99.8390029	99.6436504	99.5314641	100.309762
99.8877152	99.9845636	99.8112748	100.222494
99.9481758	100.043165	100.164379	100.251129
99.8719554	99.9643461	99.9366395	100.154164
99.8155064	99.8585707	99.750754	100.072349
99.7273945	99.7857586	99.775316	100.124317
99.7488852	99.4199398	99.8458582	100.245978
99.8963115	99.869998	100.05159	100.190223
99.9099223	99.8390858	100.40548	100.148255
100.111362	99.5501812	100.349871	100.159012
99.6818341	99.8176963	100.358713	100.062501
100.213658	99.9202486	100.134511	99.9979587
99.9941659	99.9832451	100.199551	100.056289
99.8246758	100.141322	99.9165968	100.313247
99.9295505	100.136634	99.5756758	99.9702327
99.7922964	100.029247	100.147873	100.13871
99.7829838	100.042432	100.32806	100.166436

100.022104	99.9505748	100.311751	100.214918
99.802612	99.8203334	100.208787	100.266886
99.8690899	100.05049	100.188941	100.260219
99.7839867	99.7459097	99.9474467	100.286582
100.050042	99.9549699	100.109556	100.219767
99.885871	99.9738211	100.128502	100.165738
99.8751246	99.895804	99.8223504	100.128338
99.8951845	100.061938	99.7154621	100.181486
100.005657	99.9628431	99.5928837	100.085637
99.9149579	100.018319	99.7099706	99.9903938
99.714072	99.8352053	99.6697649	100.138483
100.031592	99.9060501	99.9818004	100.10305
99.9079369	100.014367	100.186163	100.094722
99.7702398	99.6459441	100.244805	100.222067
99.9030652	99.6948329	100.089669	100.245688
99.9785765	99.8767754	100.269124	100.120312
100.085897	99.7203019	100.206756	100.074432
99.8596498	99.8537947	100.139681	100.109259
100.03675	99.8862897	99.8637329	99.9743432
100.268586	100.148299	100.042992	99.90257
100.022995	99.8496962	99.8562801	99.9828228
100.067987	100.067647	99.5475787	99.9796429
99.8583602	100.107607	99.7099706	100.135606
100.327046	100.539995	99.7203652	100.568062
100.285207	100.382204	99.834118	99.7479699
100.364157	100.273741	100.328942	99.6474268
100.189063	100.211386	100.078294	99.9735861
99.8446048	100.084919	100.216759	99.9648037
99.8269807	100.010268	100.225388	99.9499645
99.6397069	100.108924	100.240686	100.072615
99.8033387	99.9016589	100.032989	100.201474
99.847184	99.97104	99.9776818	100.123946
99.9557942	99.9561099	100.010042	99.9616239
99.8984801	100.088724	99.8517692	99.8444246
99.6461548	99.9244932	99.9698368	99.9343683
99.9691197	99.8744334	100.395233	99.8945447
99.9986365	99.8173477	100.465838	99.9649552
99.6518862	99.4874213	99.7195807	100.129852
100.180752	99.8249591	99.8551034	99.6793765
100.008666	100.218265	99.9053115	99.5685368
99.9573703	100.299649	99.8235272	99.6819506
99.6806865	100.006023	100.044364	99.7738627
99.7882937	99.9682589	100.01926	99.7190486
99.7146451	99.9995829	100.011808	99.8887907
99.9224087	100.058572	100.072018	100.08064
99.8745514	99.8763363	100.089081	100.215556

99.9504926	99.866822	100.275596	100.158925
100.221732	99.7403551	100.381112	100.102142
99.8338584	99.7510404	99.9219821	99.9470876
99.7668009	99.6850258	99.9066844	99.8330681
99.8135119	99.8407675	99.8676554	99.6466697
99.9530718	99.7525041	100.060054	99.8191374
100.278902	99.9427899	100.154391	99.7873391
100.038756	100.033542	99.843532	99.8984816
100.154817	99.6804883	99.9955292	100.022495
100.142208	99.5846135	99.9982749	100.052022
99.9851677	99.7396232	99.860791	100.039757
99.911519	99.648286	99.6401499	100.048085
99.8966174	99.7608474	99.5377724	100.053082
99.9694063	99.6951256	100.079471	100.160287
99.914528	100.104679	99.9088417	100.31307
100.034458	100.108046	99.8953091	100.167404
99.8595065	100.022564	99.8805997	100.190571
99.8716857	99.7229366	99.7248761	100.168464
100.007234	99.8037349	99.8290187	99.9542043
99.8573572	99.9597693	99.8207814	99.9210433
99.8334286	99.8384255	99.7899897	100.10305
100.060536	100.088432	99.863929	100.126672
99.9398895	100.207287	99.6170071	100.17225
100.097933	100.223535	99.696634	100.2469
99.9440448	100.063548	100.020437	100.07852
99.8317092	100.001779	99.8915827	100.1297
99.7238154	99.909124	99.845101	100.120918
100.310139	100.642163	99.9239434	100.031732
100.452564	100.6104	99.9476746	100.017498
100.442391	100.625038	99.8166628	99.8980274
100.136047	100.189722	99.7833215	99.842759
99.7046151	100.0877	99.8523576	99.9084754
99.7258214	99.8638945	100.054367	100.182092
100.057097	99.8548193	100.390722	100.306257
100.124728	99.9231758	100.240882	100.179669
99.9391731	100.033688	100.324235	99.9708605
100.111832	100.116243	100.437596	99.6896731
99.8686768	100.023003	100.356596	99.7832508
100.004225	100.085651	100.40092	99.7882477
100.143498	100.074965	100.367775	99.9952392
100.280335	99.9929961	100.188713	100.032489
100.225027	100.085504	99.9814082	99.8120207
100.080596	100.444998	100.180672	99.870166
99.9546479	100.31414	100.123207	100.000085
100.277756	100.133515	100.237352	100.04218
100.385937	99.9001952	100.142231	100.16589

100.010959	99.6823911	99.9384567	100.206319
99.7666577	99.9603548	99.7848905	100.086697
99.8269807	99.8574541	100.084962	99.9327026
99.9706959	100.236562	99.9961176	99.8668348
99.97829	100.527992	100.089669	100.003719
100.200525	100.262909	99.902958	100.035366
100.310855	100.177134	99.741939	99.7405503
99.9870304	100.040421	99.7774377	99.7859764
100.118136	99.9827499	100.046914	99.859718
100.160835	100.045983	100.069468	100.089725
100.138483	100.027248	99.7782222	100.079277
100.24609	99.765385	99.7046752	100.003264
99.9373133	99.9065621	99.7705896	100.18822
99.7638726	99.7885913	100.101046	100.15243
100.140949	99.8289883	100.365922	100.011394
100.11519	99.9052448	100.14012	99.9906171
99.7763225	99.6416402	100.049014	100.072358
99.9669355	99.8851927	100.054904	100.114366
99.8550062	99.9339453	99.9883991	100.05246
100.050633	100.063779	99.8509578	99.9648085
100.049058	100.172535	100.173749	99.7939031
100.136496	99.8244577	99.9772075	100.06171
100.15968	100.016647	99.8605786	100.249145
100.086266	100.011816	99.8299489	100.28645
100.162542	100.13316	99.957573	100.167711
100.073673	100.228742	99.9856503	100.138292
100.120468	100.080758	100.067722	100.322997
100.293055	100.443326	100.252483	100.193946
100.164402	100.122182	100.218123	100.159674
100.308367	100.518855	99.8827656	99.9416066
100.10072	100.333692	99.8164011	99.9655667
100.199606	100.220838	100.125644	99.8647219
100.105442	100.542129	100.143512	99.7332445
100.244971	100.314078	100.24836	99.8343926
100.376486	100.308223	100.294501	99.58084
99.9849467	100.33457	100.298624	99.6926033
100.025732	100.347744	100.265638	99.9344792
100.068664	100.316127	100.366167	99.8484957
99.9311387	100.309101	100.219301	100.070203
99.9458787	100.376433	100.399349	99.9625338
100.131917	100.424737	100.393655	99.881403
99.983945	100.349793	100.343391	100.091282
100.065372	100.131257	100.151169	99.9616239
100.124332	100.087638	100.273099	99.7867757
99.8760428	99.9417032	100.168055	100.149665
100.305219	99.9343845	100.215177	100.13071

100.373337	99.8683699	100.441563	100.154821
100.12147	100.395608	100.532078	99.9021785
100.126765	100.345256	100.377751	99.9884653
100.234381	100.641956	100.144297	99.9328111
99.8382628	100.234597	99.8105107	100.240805
100.000975	100.006693	99.6685534	100.108114
99.9604755	100.092175	99.5454452	100.20441
99.85472	100.14326	99.7845932	100.278868
99.9723534	100.241916	99.7323655	100.192126
100.117034	100.112814	99.9141808	100.05064
99.78746	100.083539	100.04907	99.9265936
99.7203431	100.005522	100.042787	99.8870139
99.9733551	99.8489022	100.143119	99.821351
99.7243501	99.8150898	100.139781	100.005146
100.031886	99.9706852	100.276633	100.082638
99.9410131	100.048263	100.278793	100.140112
100.072098	99.9547304	100.159023	100.098257
99.8983674	100.072122	99.9061306	100.036234
99.8462767	99.7660546	99.9591437	100.000445
100.127481	100.093932	99.8696105	100.030623
99.9676309	99.9355555	99.9911479	100.218513
100.179285	100.022209	99.8226841	99.9772435
99.7694286	99.8585628	99.8821766	99.9949861
100.010992	99.8955954	99.6514714	99.9422132
99.7687131	100.037432	99.8947426	99.9453977
100.078538	100.107691	99.9546278	99.7949646
99.9912434	99.8917897	99.4148759	100.142386
99.9531771	100.168875	99.0698981	100.058981
100.010563	100.144577	99.6526495	99.8177115
99.966486	100.211031	99.9383312	99.898994
99.9785069	100.05968	100.077736	99.9629887
99.8309643	100.060851	100.070668	100.184999
99.8487095	100.065828	100.020796	99.8901985
99.8771877	100.078563	100.11406	99.7609958
99.880336	100.132867	100.140959	99.8952028
100.057072	99.8197738	100.092266	99.9849774
99.7761546	99.7521491	100.207127	100.127373
100.01872	99.8872521	100.248752	100.143903
99.9305663	99.9730272	99.9314591	100.406706
99.835973	99.880958	99.8199353	100.208504
99.7761546	99.4923355	99.9300847	100.148149
100.001118	99.5258551	100.007052	99.9021785
99.7798754	99.7364871	100.050051	99.8674515
99.8384059	99.6094346	100.127215	99.9998388
99.934144	99.8199201	100.033362	100.073387
99.9420148	99.8705655	99.7313838	99.9464593

100.081114	99.7982568	99.791858	99.7857142
99.9627652	99.8342648	99.5187423	99.8104325
99.7943291	99.5933335	99.5364134	100.000597
99.8813378	99.5943581	100.00823	99.8158918
100.147802	99.6984299	100.258766	99.6989724
99.7604129	99.7631271	99.9194821	99.7978459
100.105013	99.8727611	99.7710454	99.5514206
100.00813	99.7338523	99.8535102	99.8348476
100.011135	99.732535	99.7669222	100.08006
99.8342558	99.8205056	99.6624667	100.022282
99.9008002	99.5293681	99.9236053	100.282053
100.064228	99.684378	100.081074	100.015458
100.032887	99.7001864	100.108169	99.9261387
99.9513167	99.4816502	99.8564554	99.8797349
100.133634	99.8361676	99.9161442	99.9023302
99.9629083	99.8162608	99.9935041	99.9886169
100.096999	99.7802528	99.9344043	99.8025469
99.9133936	99.8382169	99.6220197	100.103565
99.9275611	99.8079175	99.8270037	100.120398
99.6832786	99.6720826	99.9789746	100.191975
100.092276	99.8090885	100.253072	100.19152
100.174732	99.8055682	100.345965	99.9575392
100.350585	99.7444807	100.182744	99.776756
100.153378	100.386559	99.8953878	99.7831152
100.0231	100.392272	99.8839957	99.7908371
100.093757	99.8432168	100.13639	100.000994
100.063516	100.061931	100.221045	100.044751
99.9325224	100.075701	100.072947	100.135294
100.145065	100.134738	100.063323	99.7228541
100.245102	100.186157	100.048788	99.6801566
100.201533	100.466397	100.145425	99.8218761
99.9137476	100.267021	99.5500892	100.068523
100.16513	100.278008	99.7600575	99.8512496
99.9490042	100.276982	99.9808286	100.050808
99.9712187	100.359604	100.118909	100.080938
100.13274	100.436073	100.203367	99.9811592
100.05893	99.6543876	100.252078	100.05853
100.040299	99.6914502	99.9154222	99.9080282
99.9167573	99.6798773	99.8674968	100.028096
100.100923	99.8136253	100.051538	99.8495841
100.049471	100.030142	100.075697	100.101984
100.103789	100.161106	100.143264	100.128178
100.09605	100.265263	100.138943	100.010987
100.008482	100.135324	100.15996	100.029459
100.413789	100.098261	100.18019	99.8760808
99.9868405	99.8794006	100.055859	100.05323

99.9313759	99.6047265	99.9221003	100.168604
99.9226334	99.9457618	100.00597	100.172087
99.971792	99.676801	100.117337	100.320317
100.16771	99.9592391	99.9503842	100.27232
99.961043	99.8401405	99.9468487	100.186319
100.195657	99.8471721	99.7783241	100.203126
100.042019	99.9038649	99.6987758	100.171027
99.8756246	99.6918897	99.6158885	100.093808
100.000169	99.8522994	99.756522	100.198584
99.7930726	99.8823304	100.034254	100.206911
99.8751946	99.6908643	99.8539441	100.244461
99.9643394	99.9571882	99.832142	99.991455
99.7632621	99.9132404	99.9482236	100.01886
99.8145705	99.8786681	100.001452	100.269141
99.8890966	100.023696	100.055859	100.197372
100.076559	99.8963937	100.350876	100.227049
100.118121	99.9620225	100.222616	100.189196
99.9932899	99.9851684	99.9847569	99.9946346
99.826896	99.7447736	99.728631	99.9929691
100.018657	99.8612354	99.847266	99.9673809
100.086304	99.9147053	100.223009	100.045357
100.203683	99.5900772	100.309825	100.131206
99.9983061	99.8190455	99.9908458	100.185108
100.010345	99.9270107	100.063323	100.105164
100.429124	100.077019	100.152496	100.005082
100.117691	100.095184	99.9596157	99.9664724
100.056207	99.9508891	99.7453263	100.150889
100.20354	99.9202721	99.7916804	100.055804
100.055061	99.9189536	99.8604258	100.144682
100.133456	99.8552292	99.8329277	100.182988
99.9932899	100.046402	99.9672759	100.061255
100.012351	99.9816526	99.8339097	100.123484
99.9018521	100.012856	99.9480272	100.205094
99.8188701	99.9032789	100.007934	100.155735
100.095047	99.9340424	99.9662938	99.8662391
99.9759482	100.016371	99.9543125	100.047779
100.003752	99.8309114	100.03936	100.057772
100.077992	100.121846	100.215938	99.8992465
100.237363	100.021645	100.176066	99.8653307
100.461515	99.9548444	100.223795	99.8998521
100.279929	99.8731014	100.072947	99.9019718
100.138759	99.9268642	100.036807	99.7192203
100.022957	99.932138	99.9254394	99.7469283
100.011491	99.9478127	99.892638	99.7214914
100.171723	100.174291	99.7457191	99.6890897
100.148505	100.06823	99.5110025	99.7336042

99.9831142	100.105878	99.7545578	99.7838722
99.7366047	99.8903875	99.7608431	99.8533693
99.8462441	100.02853	99.9607942	99.885771
99.6076172	100.133273	100.018148	99.8748695
99.9634795	100.12712	100.065091	99.8978838
99.8585696	100.327376	100.312967	99.9431553
99.7880564	100.077898	100.035432	100.02098
99.8632991	99.9987922	100.075304	99.9442152
99.6547693	100.014613	100.099071	99.9737401
99.8138539	100.032632	100.209456	99.8320205
99.7625455	100.068816	100.153871	99.8470101
99.7341683	100.113496	100.009898	99.9551167
99.9074415	100.176635	100.20926	99.9805535
99.78476	100.080975	100.172334	99.9632928
99.9077282	99.8016129	100.170566	100.121213
99.8853703	99.9382907	100.274666	99.9599618
99.9352455	100.038638	100.157406	99.9004578
100.162694	100.203882	100.100642	99.8763836
100.118838	99.993079	99.8934237	99.8851654
100.048755	99.8583056	100.019719	99.9349792
99.9325224	100.091962	99.8629792	99.8944014
99.9415516	99.9412205	100.06568	99.9938776
100.16126	100.00641	100.158388	99.8895562
100.070252	100.28753	100.095339	99.901669
99.7092307	100.139425	99.933296	100.021586
99.8085511	100.251639	99.7952159	100.044903
99.7648386	100.119942	99.8739785	99.8648765
99.9001322	100.07907	99.9690437	99.8830456
100.070969	100.250906	99.9093333	99.8825914
99.8562184	99.8750473	100.512144	100.22906
99.9232761	99.8314616	99.853728	100.169077
100.166129	99.9144972	100.328684	100.09286
99.7706364	99.7051585	100.504925	100.016984
100.113335	100.098225	99.1395518	100.143273
100.419541	100.230891	99.8066805	100.040054
100.129821	100.211803	99.982673	100.20787
100.088327	100.339537	99.6145076	100.322196
99.8725197	100.154855	99.3700597	100.089271
99.9886667	100.151832	100.261473	99.8515607
99.7639677	100.105065	100.034201	99.877878
99.8923407	100.053685	99.7832812	99.7652605
99.7232143	99.9054301	100.310014	99.7543235
99.6198491	100.071342	100.270434	99.7025433
99.9166074	100.026802	99.7758133	99.6991255
99.957546	99.6407344	99.6396494	99.7992679
99.706913	99.6358032	100.274168	99.8014895

99.8041653	99.8710705	100.121326	99.985027
99.9143845	99.8238262	99.9632565	99.8095214
99.8573298	100.010258	100.046399	99.9342722
99.9158664	99.9356537	100.169618	99.8623269
100.029605	99.900817	100.293087	99.898385
99.8740017	99.9345402	100.125558	99.8770236
99.992742	99.9708086	99.5828936	99.9537539
100.181875	99.9078161	100.131034	99.8597635
99.860479	99.9663546	100.250769	99.9578552
99.7800838	99.8830009	99.9254194	100.157286
100.056651	99.942812	99.8129037	100.219832
100.403239	100.287521	100.213181	99.9166704
100.385456	100.307723	100.340881	99.9470891
100.315805	100.243776	100.260477	100.090638
100.092588	100.252366	99.6672804	100.092176
100.287833	100.341287	99.9821751	100.062612
100.096848	100.249025	100.259731	99.9829763
100.167055	100.534082	100.102408	99.9706721
100.017194	100.276863	99.7554012	99.9513614
100.092958	100.168376	99.8353072	99.9395699
100.112223	100.096475	100.477045	100.03732
100.051834	100.068956	100.109627	99.8642067
100.091106	100.031097	100.150202	99.9277783
100.054613	100.252207	99.7877619	99.958197
100.243005	100.396167	100.090708	100.014762
100.220775	100.256661	100.190279	99.9954514
100.076842	100.129403	100.138751	99.7490258
99.9727359	100.409529	100.417551	100.023136
100.012563	100.370715	99.7038729	100.16378
100.227444	100.267478	99.7842769	100.060219
100.077398	100.021075	100.138751	100.08329
100.147975	100.265887	100.209447	100.084144
100.055169	100.116359	100.303791	100.252472
100.058503	100.005327	99.8031955	100.147374
100.091291	100.264137	99.7491779	100.252301
100.080547	100.050345	100.090459	100.022281
100.191137	100.193668	100.245293	99.8525861
99.9127173	99.9464706	100.54326	99.9826345
99.9036404	99.7931253	99.7526629	100.111145
99.9082715	99.8580266	99.5908594	100.051846
99.9706982	99.8615262	99.9055051	99.7758558
99.724511	99.7767409	99.9729648	99.9544374
99.7882344	99.7988519	100.203971	100.08346
99.9555083	99.7889894	99.7596329	100.049453
99.8888211	99.788035	99.5248933	99.9759697
100.009043	99.8903182	99.6857012	100.077479

99.9794046	99.7913755	100.07403	100.047915
99.8293582	99.6976821	99.8689126	99.8896695
99.8662215	99.9205419	100.186297	100.217781
99.9371693	99.847687	99.7855215	100.239997
99.9616213	100.185556	99.6267051	100.192318
99.9041962	99.9342221	100.176837	100.237092
99.7560022	99.7115214	100.208451	100.203939
99.8958603	99.8258941	100.343868	100.06808
100.03683	99.9181558	100.248778	100.143443
100.084622	100.005009	99.5276315	100.04313
100.0372	100.105383	99.7583883	99.9951096
100.096107	100.053208	100.287113	99.8589091
99.9510625	100.041755	99.9776944	99.8954799
99.9882962	100.090113	100.231104	99.9337595
100.001263	99.9113157	99.9045094	99.8775362
100.09833	99.918474	99.3217675	99.9870777
100.070359	99.6720716	100.023746	99.9470891
99.884931	99.7442902	100.102657	99.9129108
99.9099387	100.001987	100.048888	100.109778
99.9473577	99.7045222	99.804938	100.163096
99.9271662	99.8777515	99.2458443	100.298955
99.9060486	99.74604	100.00433	100.021769
99.8280615	99.6855927	99.7810408	99.9995528
99.6752366	99.7237699	100.307774	100.027066
99.7447025	99.7174071	100.06233	100.032193
100.013304	99.874411	99.9846644	99.9884448
99.8113897	99.6233956	100.177335	99.810205
99.7808247	99.7331552	99.3872358	99.8462631
100.217256	99.894295	99.8303287	99.9990401
100.069432	99.8869777	100.268194	99.9870777
100.235039	99.9278592	100.231851	99.9327342
100.468259	100.096316	100.038931	99.944013
100.196509	100.059889	99.9401062	100.018864
100.20892	100.011213	100.059841	100.1255
100.212625	100.036823	99.9891451	99.9253859
99.9471724	100.080886	100.249524	100.070473
99.909383	100.141016	100.448667	100.118151
100.181328	100.489212	99.9590511	100.136141
100.369362	100.515054	100.186376	100.016505
100.195249	100.407537	100.170938	100.028639
100.159796	100.586839	100.360417	100.045388
100.118959	100.627516	100.234679	100.19801
100.106337	100.525104	99.7506496	100.133407
100.056776	100.404985	100.016318	100.224501
100.175945	100.27705	100.195588	100.012232
100.095014	100.330329	100.550145	99.9447228

100.122115	100.273859	100.397765	100.016163
100.165736	100.167618	99.8241005	99.9623264
100.203231	100.307039	99.4946916	100.007447
100.229404	100.368136	99.852485	100.176476
99.9459603	99.9816174	100.365646	100.083501
100.454933	100.37659	100.148032	100.002661
100.017981	99.9178091	100.672148	99.7502279
100.185968	99.6933636	99.7919813	99.7825298
100.452891	100.068078	99.2748367	100.002661
100.355812	100.047499	99.943614	99.9635228
100.099098	99.8071018	100.154007	100.020948
100.365835	100.203511	100.445321	100.274578
100.267456	100.019264	100.216005	100.150839
100.327226	100.173042	99.8350559	100.00437
100.191166	100.235255	100.452293	99.9286573
100.025777	100.213401	99.8990454	100.113752
99.8783943	100.241955	100.166706	100.119221
100.097241	100.335753	100.161477	100.18468
99.8037746	100.048776	100.090516	100.105036
99.8685564	100.21691	100.35718	100.069658
100.168891	100.279921	99.7728093	100.073418
99.9288832	100.0006	100.104459	99.8746499
100.146988	100.119603	100.299415	99.8517481
100.021879	100.149912	100.306636	100.172032
99.9129198	100.018148	99.852734	100.035988
100.044339	99.936154	99.8748937	99.9859119
99.7790871	99.7215988	100.457522	99.8495262
99.8340309	99.9921458	100.103712	100.086065
99.9036387	100.02389	99.6971175	100.141952
99.8399707	100.001079	100.225466	99.9859119
99.7803864	99.9993242	100.256839	99.8804608
99.8828492	99.9889553	100.534708	99.8843918
100.021508	99.9840102	99.8624444	99.9170354
100.125456	100.019264	99.3878765	99.9508755
99.9940362	99.8551175	100.155252	100.086065
100.039328	99.8284776	100.222977	100.082134
100.063087	99.9588059	99.8547258	100.288592
100.038585	99.8988262	99.6719699	100.204676
100.145503	99.8468225	99.7172854	100.063676
99.9190453	99.769455	99.8011938	100.203992
99.9893957	99.5673424	99.9007883	100.099738
100.337621	99.6794854	99.3612349	99.9560028
100.298826	99.935835	99.6229196	100.045559
100.080164	99.8115684	99.906764	100.100763
100.056776	99.7932236	99.6283973	99.8187626
100.193579	99.7530244	99.6933827	99.8862718

100.363979	99.8413988	99.9724964	99.7765479
100.240355	100.0207	99.7093178	99.804748
100.116175	100.085146	99.5888084	99.9038754
99.8945433	99.8812789	100.583758	99.7932971
99.9361224	100.090091	99.6129601	99.9327591
99.7833563	100.205744	99.7165384	99.9713846
100.08926	100.09392	99.432943	100.083672
99.8965851	100.117688	99.4302041	100.094439
99.7087367	99.9462038	99.3711944	100.074785
99.996078	99.7951378	99.359741	99.825428
100.056776	99.8661245	100.097737	99.7818461
100.02652	99.9908696	99.4526129	100.029665
99.9541277	99.9701319	99.0636962	99.8606354
100.025777	99.8709101	99.8390397	99.6172604
100.087218	99.7664241	99.6963705	99.7815043
100.038585	99.572447	99.5143615	99.7751807
99.9305538	99.5421381	99.6376098	99.9438682
100.255576	99.5955775	99.4575926	99.7779152
100.098726	99.5400643	99.8415296	99.6295659
100.160909	99.6124867	100.13857	99.8741372
100.10875	100.001877	99.8736488	100.110505
99.9138479	100.091846	100.080805	99.9971919
99.8912021	99.9951766	100.429386	99.8982354
99.7987629	100.075096	100.405235	100.112385
99.7612674	100.140659	100.353943	100.184509
99.69927	100.149752	100.636294	100.131869
99.753657	100.382333	101.015998	100.037868
99.7094792	100.075416	100.4996	100.147079
99.8156544	100.128536	99.97897	100.048465
99.6385719	99.858946	100.715721	99.7727879
99.7878112	100.170809	100.284227	100.043679
99.7135628	100.203351	99.9729944	99.9799301
99.9331525	100.1204	100.334772	99.9525846
99.9080937	100.137788	100.24663	100.119905
99.7640517	100.169692	100.409716	100.074443
99.9402061	100.176871	99.5912983	100.063334
99.6454399	99.8876598	100.175669	99.9896719
99.6239079	99.7946592	100.332282	99.8452535
99.824193	100.060261	100.137325	100.056668
99.7840988	99.940142	99.9144826	99.9700174
100.004802	99.8437916	100.024286	100.117854
99.8438688	99.8265634	100.212021	100.333883
99.6719837	99.785088	100.342739	100.114436
99.8713407	99.9372707	99.9363934	100.138021
99.8483237	99.8654864	100.444823	100.056668
99.6245008	99.8183938	100.443015	100.098979

99.7393556	99.8874691	99.8908049	100.333563
99.7660316	99.6296305	100.087649	100.038838
99.7236094	99.5416152	100.249654	99.9568276
99.6532145	99.7532975	100.266577	100.000737
99.680261	99.5390687	100.295693	99.982285
100.027419	99.6673513	100.220538	100.057632
99.7984503	99.4174707	100.700083	100.180306
99.8890374	99.6387026	100.051814	100.069763
99.9010786	99.8686883	99.5837173	100.222678
99.8440217	99.4962547	99.2161579	100.321774
99.9831442	99.6702162	99.5063233	99.9735714
99.5681849	99.4749274	100.124231	100.123753
99.7599184	99.4174707	100.093373	100.263683
100.027789	99.7052313	100.216059	100.076084
99.9535043	99.7201923	100.420369	99.9248778
99.9662865	100.174434	100.536087	100.096074
100.148016	100.060794	100.028173	100.074376
100.359386	100.269611	99.4378881	99.9906569
100.118747	100.198626	100.188187	100.013893
100.138383	100.08801	100.253636	99.9908278
100.096332	100.361606	100.400461	100.060878
99.9375728	100.179686	100.299425	100.152627
100.233417	100.353966	99.3547703	100.175009
100.133937	100.136076	100.28972	99.94931
100.131344	99.6700571	100.25488	99.8659328
100.004633	100.003656	100.205607	100.165442
99.8844061	99.8271476	100.406931	100.107351
99.9701768	99.6940902	99.8920491	99.942305
100.074102	99.630108	99.5483798	100.142376
100.069656	99.6940902	99.8711453	99.9868981
100.03909	99.841472	99.9756646	99.7589776
100.018342	100.182074	99.9873608	99.7880229
100.106335	100.330251	100.243931	99.9906569
99.9485025	100.112998	100.023943	99.9669081
100.067433	100.158359	100.083668	100.030124
99.7688103	99.7907001	100.295195	100.037984
99.7110124	99.8647093	100.154592	100.002958
99.7521379	99.6897929	100.1745	99.9566568
99.9286808	99.9056133	99.7616489	100.128708
100.002595	99.8088443	99.8310796	99.9945866
100.051687	100.021004	99.8452643	99.9675915
100.048908	99.9280548	99.9072293	100.117602
100.099666	100.153584	99.4281827	100.192778
100.163207	100.17905	99.5018439	100.060537
100.088737	100.222659	100.000301	99.8592694
100.087996	100.256719	99.7852902	99.8722544

100.12875	100.594297	99.6735541	99.9915112
100.044462	100.170137	99.6421983	99.7921234
99.9294218	99.9888538	99.9239026	99.8416714
99.9679538	100.242713	99.467253	100.03918
99.9811065	99.9915595	99.20048	99.6979823
100.026122	100.240008	99.5317065	99.7195101
100.018156	100.100584	99.7021725	99.7890481
100.049093	100.138941	99.2811092	100.021582
100.089478	100.039148	100.132195	99.8980535
99.8862586	99.9804183	100.323564	100.01731
99.9351646	100.12032	100.11353	99.9349582
100.000002	100.263563	99.6120868	100.04123
100.080956	100.202605	100.08541	99.9578528
99.8751437	100.056815	100.234225	99.9011289
99.965916	100.38723	100.228502	100.122215
100.070953	100.294122	100.083917	100.109914
99.95406	100.009545	100.333021	100.166979
99.7541756	99.9355353	100.226013	100.027732
99.7838156	99.8890607	100.520658	99.9628076
99.669887	99.9150037	100.407926	100.148014
99.7352802	99.9657757	99.5772471	99.9351291
100.287695	100.028007	100.384036	99.9325662
99.9612848	99.6905887	100.964367	100.054044
99.6289468	99.7860844	100.706055	99.9722046
100.125786	99.9091148	100.384783	99.9877524
100.109485	99.9304422	100.451725	100.111451
99.7495444	99.9345804	100.62891	100.138446
99.8827389	99.9143671	99.7643863	100.053019
100.084476	100.008908	99.5242409	100.078647
99.9242348	100.009385	100.036136	99.8403045
100.022047	100.377522	99.8071895	99.8541438
99.9162691	100.155016	100.039372	100.044476
100.386618	100.511216	99.8524811	100.081039
99.8738469	100.036443	100.305149	99.9083048
99.7525084	100.267701	100.087401	100.012185
99.8912604	100.15804	99.873385	100.1012
100.031309	100.144512	99.7708566	100.158095
99.9507255	100.031986	100.005527	99.8153597
100.107262	100.349828	99.8213742	99.8937822
99.8908899	99.9964935	99.9313683	99.8667871
100.125786	100.258311	99.6578762	99.8714002
100.126527	100.118091	99.9562538	99.7024246
100.00204	99.9484273	99.7865345	99.7376207
100.239345	99.9070458	99.4754652	99.7234397
100.048352	99.71685	99.8233651	99.9161642
100.037978	99.7171683	99.4533171	100.01133

100.206926	99.7147809	99.1601654	99.8859228
100.186363	99.6581201	99.7230763	99.9744257
100.382728	100.087055	100.11577	100.180989
100.191365	99.8889016	100.225515	100.153653
100.066507	100.140851	100.56147	99.9394004
100.126342	100.389936	100.668976	100.064808
100.154315	100.284572	100.357409	100.182698
100.216402	100.369047	100.514734	100.17728
100.030346	100.274821	100.763675	99.9705182
100.121521	100.372389	100.11344	100.009649
100.120965	100.207813	100.315332	99.9230142
100.111699	100.359179	100.63074	100.101923
100.15321	100.232484	100.44503	100.180527
100.196018	100.388783	99.93719	100.101239
100.189902	100.29233	100.155511	100.103461
100.057958	100.210678	100.727329	100.115251
100.161734	100.408679	100.319315	100.145155
100.107067	100.466137	99.8234239	100.118669
100.052955	100.17789	100.669824	99.9426651
100.255504	100.342148	100.621778	99.914983
100.060182	100.263202	100.078091	100.108245
100.165811	100.172001	99.6999491	100.117985
100.186196	100.069181	100.266788	100.034255
99.9940246	99.9994666	100.545851	99.9365136
99.8807972	99.9291159	99.9650714	100.09355
99.8728287	99.8824806	99.823175	100.107562
99.8774616	99.8184965	100.559543	99.8587643
100.199724	100.003605	100.347694	99.8022038
100.024416	99.9423265	99.9551138	100.085177
100.031458	100.0426	99.649663	99.9770115
100.052769	99.8325029	100.014611	99.8965282
100.002549	99.7234752	100.656381	99.9383932
100.082976	99.9251368	100.132111	100.079709
100.090574	99.8239081	99.6526502	100.030667
100.098727	99.8815256	100.113689	99.9618034
100.10929	100.056607	99.721607	99.8946486
100.011259	99.7637438	99.7811039	99.9033633
100.286637	99.9822769	99.6862573	99.8462902
100.347791	100.026525	99.9259877	99.942836
100.563868	99.9967608	100.099749	100.047242
100.205839	99.8632217	99.93719	100.109612
100.104843	99.8705433	100.031539	100.103119
100.119853	100.065361	100.310104	100.052198
99.7712762	100.05215	100.916276	100.164293
99.8685664	100.220387	100.293176	100.272288
99.9061854	100.086052	100.108959	100.192488

99.8187168	99.8067183	100.638955	100.038869
99.963633	99.958243	100.353918	100.196418
99.8131573	99.6464395	100.006396	100.115251
99.9519581	99.8340946	99.6446841	100.013579
100.011629	99.9018987	100.464199	99.864745
99.815937	99.6117416	100.158748	100.068431
99.732916	99.4571928	99.7524757	100.002301
99.789437	99.5867528	100.108462	100.01768
99.6315488	99.4511445	100.172439	100.169078
99.9564057	99.7809336	99.8851613	100.216069
100.220665	99.777432	100.342715	99.9120781
100.078343	99.7799786	99.8774441	100.059716
100.077602	99.7244302	99.36587	99.9575315
100.252539	99.8250222	99.8296474	99.8765355
100.373549	99.8764324	99.5027877	100.06638
100.448972	99.8762732	99.2033114	99.9389058
100.427105	100.114543	99.6904893	99.728043
100.316843	100.056766	99.7900658	99.7996407
100.190644	100.01029	99.4908385	99.8910601
99.9602973	99.9596755	100.20057	99.9001166
100.169703	100.356314	99.7865806	100.252124
100.013112	100.338806	99.840103	99.9424943
99.7773916	100.306654	100.319066	99.7215496
99.8483671	100.397378	100.127132	100.129263
99.4731046	100.087325	100.071618	100.178134
99.5425977	100.235985	100.786328	100.022636
99.6146851	100.24267	100.603606	100.144642
99.6662027	100.357905	100.180406	100.155066
99.5959683	100.098626	101.028299	99.942836
99.8978462	100.126321	100.507514	100.224442
99.956591	100.19126	99.8381114	100.048438
99.7408845	99.8648134	100.201068	100.093721
99.6480418	100.117408	99.755463	100.244264
99.6786188	100.125843	100.260565	99.956848
99.6473006	99.9751144	100.325787	100.09936
99.7949965	99.8769099	100.10871	100.061254
100.075378	99.9829135	99.9364432	99.8327909
99.913042	99.7963726	99.9889698	99.919084
99.9780875	99.7825253	99.7420201	100.027762
100.362986	99.9767061	99.4042069	99.8095516
100.181748	100.058039	100.220236	99.8186081
99.9908742	100.191897	99.7816018	100.156433
99.7584894	99.9187702	99.5543185	99.8199751
99.9104476	100.169295	99.2359227	99.7910968
100.06778	100.034642	99.2662935	99.8336453
99.9612239	100.097353	99.7621844	99.8650868

100.214179	100.077935	99.4408013	100.099872
100.24142	100.086371	99.3165796	99.9899982
100.367249	100.062337	100.023822	99.8931107
100.271997	100.154493	100.034028	99.8850794
100.127822	100.103401	99.5279307	100.015117
99.9258287	99.8372779	99.7738846	100.185311
99.8908042	99.8275688	99.607094	99.9753027
99.7507062	99.9499664	100.022079	99.9153247
99.8541119	100.070295	100.122402	99.9127616
100.059255	99.9443957	99.7430159	99.8121147
99.7484824	99.8533535	100.049214	99.9705182
99.7419964	99.8969646	99.6309924	99.9701764
99.8120454	99.9138361	99.2540954	99.7695662
99.9829057	99.8630626	99.8732121	99.7637564
99.9767903	100.035597	99.9424178	99.9202802
100.009142	99.4740756	100.043899	99.6954378
100.177531	99.7012008	100.087534	99.887714
100.617047	99.7888576	99.6081729	99.999047
100.372537	99.6182398	99.3127939	99.8102274
100.208228	99.6863304	99.4573366	99.7583776
99.9874943	99.3242763	99.9190761	99.6049887
100.14506	99.8167199	99.4730706	99.935387
100.06397	99.9026549	99.6509693	99.813684
100.131575	99.9165861	99.9597746	99.62256
100.028305	100.057463	99.6528574	99.6876603
100.097506	99.8375384	100.185924	99.8051864
99.9676212	99.8306511	100.237322	99.9503658
99.9330206	99.9230039	99.7537645	100.088488
100.123412	99.7099664	100.07222	100.177785
100.015885	99.5697155	99.6471931	99.90903
99.7965701	99.252116	99.5039092	99.6552542
99.8295738	99.3261547	99.8573989	99.7779653
100.047824	99.5265132	99.9711031	100.07855
99.8975328	99.5639239	100.040542	100.008265
99.8609804	99.6979136	100.033829	100.000343
99.8734012	99.7732046	100.15089	99.8056185
99.9846553	99.7681956	99.8133438	99.7216507
99.8120073	99.4881633	99.7556526	99.4703234
100.05705	99.7306284	99.8345322	99.728276
100.121816	99.611196	100.151939	99.6731136
100.034516	99.7564559	99.8246723	99.6202556
99.8875963	99.6052478	99.6203405	99.569126
99.7387251	99.3604348	99.9169782	99.574455
100.293044	99.7312545	100.095297	99.8208854
99.8370262	99.8503739	100.009494	99.8237659
100.107798	100.344696	100.269209	99.8426335

100.160142	100.35174	100.257881	99.9896852
99.8599158	99.993912	99.8557206	99.7423906
99.8778371	99.9870247	99.8301267	99.9215604
99.6693465	99.9663627	99.972152	99.8180049
100.021208	100.074055	99.9383764	99.9518061
99.7580659	100.152633	99.9469777	100.027276
100.041968	99.92363	100.008235	100.070484
99.9369243	100.064037	100.20984	100.099434
99.8900804	99.8519392	100.133478	99.8672621
100.153755	100.108335	100.180889	99.9541105
100.015175	100.124928	99.9671171	99.8784963
99.9599913	100.067481	100.467247	99.7480077
100.083844	100.239664	100.262706	99.6892446
99.9876718	100.038367	100.458227	99.8483946
100.119509	100.217437	100.53312	99.9277535
100.262347	100.468824	100.21991	99.9928538
100.121993	100.279423	100.174596	99.9605917
100.1243	100.219002	100.061731	100.056226
100.056518	100.07609	99.9350198	100.229634
100.29127	100.040558	99.7904771	100.23986
100.274235	100.148407	99.8569794	100.27846
100.047291	100.058872	99.8773286	100.029293
100.152512	100.280205	99.6094316	100.02382
99.9699279	100.089395	100.011172	100.062131
99.9429572	100.084699	99.808309	100.059538
100.278317	100.376002	99.7411774	100.019499
100.293754	100.417013	99.8181689	100.056658
100.106023	100.185192	99.5756561	99.9925657
100.105669	100.246552	99.3956595	99.9775869
99.8950487	100.164686	99.987886	100.210191
99.5873698	99.793397	100.105366	99.8437857
99.9055176	100.118353	99.8953702	99.9594395
99.9211322	100.059185	100.084178	100.120606
99.9614108	100.105518	100.462842	100.173464
99.8544152	100.183157	100.360886	100.170727
99.9459736	100.133067	100.225784	99.9696654
99.8773048	99.9305173	100.146904	100.080566
100.314514	100.396507	100.261237	100.042831
100.160142	100.291476	100.235014	100.181961
100.068584	100.151538	100.126555	100.11182
99.863642	99.8762014	100.187812	99.8051864
99.7135288	99.78604	100.110821	100.043983
99.8627548	99.9751284	100.166414	100.240148
99.9025011	99.9984514	99.9408938	100.215088
99.9182932	100.210863	100.026067	100.257
100.093425	100.239821	100.095506	100.251671

100.082602	100.21415	99.9293556	100.229634
99.8808536	100.190514	99.9585159	100.246054
100.105314	100.040558	100.59207	100.164246
100.383538	100.439083	100.463681	100.097418
99.9017914	100.094248	100.064248	100.235395
99.8751756	99.913299	99.5968445	100.079846
99.7600177	99.8319034	99.7936239	100.074805
99.8746432	99.9610407	100.145016	100.105627
100.116137	100.167973	99.7820857	99.9073017
100.003641	100.382107	99.5502719	100.182682
99.9929949	100.305563	99.7596386	100.316051
99.6769764	99.8605484	99.986837	100.146531
100.085973	100.043845	100.112289	100.294159
100.024047	100.001425	100.163267	100.104043
99.8820957	99.8331556	100.252007	99.9071577
100.14435	99.8658704	100.025228	100.215664
99.7816653	99.7999712	100.134736	100.117293
99.6988013	99.7891707	100.130331	100.097562
100.109217	100.121484	100.25872	100.328149
100.197937	100.117414	99.8907549	100.266361
100.172386	100.046663	99.8479586	100.158053
99.9693956	100.077812	99.9450896	100.064003
100.185871	100.405743	99.8179591	100.310578
100.172592	100.47389	99.857349	100.174843
100.083343	100.466985	100.116845	100.026561
100.394027	100.666465	99.6949545	100.274657
99.7868546	100.279589	100.306445	99.8824497
99.9736905	100.327772	99.8638364	99.9819757
99.9964018	100.304701	99.5484646	100.088405
100.129831	100.191856	99.7604564	99.9335071
100.334055	100.357749	99.2619722	100.173836
100.148284	100.335934	99.9389648	100.078769
100.193174	100.306114	100.172721	100.047272
100.071988	100.280217	99.8864377	100.015199
100.075891	100.249142	100.10701	99.930343
100.008467	100.383489	99.751667	99.9372465
100.051406	100.405618	99.8864377	100.076036
100.261485	100.559113	99.5264911	100.084953
100.45187	100.683729	99.7656882	100.179014
100.141364	100.402008	99.5250262	100.22777
100.076601	100.307055	99.6415903	100.06942
99.8970399	100.289948	99.5827851	100.01937
100.127524	100.688438	99.8069146	100.206628
100.241435	100.436537	99.8856006	100.14838
100.340975	100.5464	99.6543559	100.339377
100.170995	100.271428	99.6847002	100.379504

100.106232	100.134884	99.7985438	100.461052
100.039695	99.9175114	100.006559	100.322838
99.7481744	99.8928706	100.017442	100.386839
99.6715238	99.832132	99.823447	100.388277
99.8022912	99.6659245	99.8420722	100.190232
99.5079315	99.5263981	99.6997677	99.9053177
99.5068669	99.7316855	99.8029385	100.03706
99.5274491	99.7596222	100.684389	99.8034906
99.8592468	100.094548	99.9546601	99.9651483
100.087069	99.9721292	99.9368721	100.228777
99.9222352	100.022823	100.041298	100.149242
99.9018305	99.963497	100.049251	99.8427544
100.099667	100.082463	100.518856	99.6995062
99.8887005	99.8785884	100.398525	99.8759777
99.7464001	99.6828748	100.141121	99.7264012
100.130363	100.055154	100.177534	99.8480759
99.6860732	99.6872694	99.9971422	99.8631774
99.612439	99.5499403	100.005932	99.6777888
99.4227642	99.5714421	100.161002	99.5506488
99.7788702	99.7786128	100.09843	99.6806653
99.6839441	99.5976523	100.202019	99.7229494
99.7483519	99.6452074	100.223574	99.7822047
99.9454788	99.6852291	100.154723	99.8174415
99.8191472	99.7334119	100.193857	99.7612065
99.7662725	99.6739288	100.246803	99.7256821
99.7125106	99.7615056	100.166024	99.6993623
99.8239379	99.9780932	100.121659	99.8427544
99.9220578	99.6924486	100.384294	99.832543
100.086892	99.7200714	100.296191	99.8401656
100.286326	99.8997763	99.8165411	99.9464512
100.30815	99.742358	100.006978	99.9290486
100.362621	100.038361	99.952986	100.069277
99.9335908	99.912803	100.437868	99.9135156
99.6938802	99.8712119	100.191974	99.9520604
99.7769184	100.302504	99.9580085	100.090706
99.8077916	100.467299	100.064946	100.087111
100.013435	100.698482	99.913643	100.195697
99.8377776	100.381448	99.6453572	100.321831
100.029404	100.314903	99.902133	100.175418
99.8384873	100.011837	100.129192	99.9398354
100.260243	100.350373	99.9364535	100.085672
100.190867	100.402322	100.061179	100.058634
100.207368	100.257774	100.222318	99.9867218
100.139234	100.138023	100.232572	100.087255
100.181108	100.186833	100.238223	100.188938
99.8358258	99.9255157	99.9958866	100.057195

99.8661667	99.9760528	99.9778893	100.053744
99.8787644	100.01529	100.263545	99.9509098
99.8423908	99.9093501	99.8393517	99.9631348
100.056906	100.126095	100.176488	99.9844207
100.046792	100.117776	100.058459	100.048422
100.148638	100.110243	100.147818	99.9581009
100.056551	99.9253588	100.16728	99.8666292
100.351798	100.283827	99.336682	99.8966883
99.7242211	99.7641737	99.7179743	99.459177
99.762369	99.7748461	99.9228509	99.7251068
100.031711	99.897893	99.8441649	99.809675
100.162478	99.9412105	100.018279	99.9378218
99.8320997	99.7756308	100.177953	99.9848521
99.9570119	99.8767051	100.284053	99.8882027
100.09949	99.7958771	100.419661	100.032889
100.248178	100.304544	99.8560934	100.305435
100.155558	100.01937	99.9724482	100.130545
100.075359	99.7170894	100.056994	99.9389724
100.238774	99.9193948	100.136517	99.9402668
100.339023	99.8993055	100.384713	99.8784227
100.311344	100.010895	99.658332	100.093439
100.047502	99.7348245	100.10408	100.003118
99.7652079	99.5855674	99.7537597	100.227339
99.7574009	99.556846	99.9492191	100.136298
100.331926	99.8575575	99.8828801	100.265596
99.9979986	99.5345594	99.9370814	100.126518
100.214998	99.5887063	99.9969329	100.048566
100.147219	99.6233917	100.088594	99.9588201
100.085118	99.553864	100.151375	99.8633212
100.487179	99.9192378	100.028533	99.9639977
100.368969	99.7814032	100.01252	100.098016
99.9202819	99.4258129	100.371956	100.142006
100.111715	99.5432484	100.086084	100.199223
100.156424	99.4602147	100.296296	100.070989
100.458387	99.8600584	100.378034	100.104197
99.930927	99.4458285	100.328572	99.9627373
100.002603	99.5601366	100.041023	99.8651241
99.9589588	99.6072046	100.273032	100.117998
100.012894	99.4689716	100.397106	99.9732318
100.028861	99.2015752	100.27932	99.9692065
99.9962164	99.2739755	100.099078	99.9279473
100.051038	99.4165869	100.072251	99.9191779
100.061683	99.4006369	100.126953	99.805751
100.465306	99.7097847	100.370279	99.8812252
100.266245	99.7151014	100.331925	100.025129
100.324083	99.6043899	100.102012	99.9372917

100.16423	99.6172124	100.059676	99.9969523
99.9225884	99.582185	99.9848548	100.010322
100.080312	100.277572	99.9636869	100.163283
99.8150738	100.391411	99.9485968	100.168171
99.884976	100.373428	99.8081757	100.009891
99.9539911	100.457087	100.070155	100.094278
99.9323463	100.405797	99.9697648	100.200517
99.8739761	100.192818	99.8375175	99.9049457
99.994797	100.006579	99.922818	99.9577057
100.168488	99.9405901	100.243691	100.110235
100.149505	100.201575	100.444472	100.07487
100.19102	100.350129	100.175786	100.099453
100.029748	100.129019	100.135546	100.205117
99.8290897	100.020027	100.199259	99.8911447
100.077473	100.2169	99.9997353	100.085077
100.325679	100.39704	99.8643442	100.196923
100.09486	100.367955	99.7134439	100.125762
100.124311	100.228471	99.9003926	100.099597
100.129811	100.301184	99.7509594	100.158826
100.012716	100.173585	100.220637	100.207849
100.188181	100.319011	100.226086	100.292811
100.070199	100.359824	99.9844357	100.400344
99.9179755	100.020809	100.403813	99.9887579
100.227568	100.219714	99.9789865	99.9542555
100.034893	100.201888	99.9232372	100.089678
100.009877	99.9989169	100.028867	99.9568431
99.9962164	100.158729	100.052341	100.017798
99.8384928	99.9909419	99.8209603	100.049569
100.144359	100.289613	99.8930571	100.016647
100.144892	100.183436	100.026772	100.227113
100.00349	100.031442	100.023418	100.052012
100.119876	100.19923	100.216654	100.049137
99.9958615	100.239574	100.1433	100.021679
99.8516216	99.9093157	100.263182	100.0569
100.092554	100.086329	99.9687169	100.149913
99.8888792	100.015805	99.7941336	100.23085
99.8860405	100.118854	99.8331162	100.114117
100.121118	100.205641	99.778834	100.01061
100.067893	100.038635	99.9561418	99.7923813
100.060974	99.9157269	100.128001	99.9321164
100.114553	100.07851	100.051293	99.968344
99.7827839	100.025187	99.7530552	100.072858
99.9875229	100.13371	99.8775479	100.035049
100.133182	100.043483	99.8046128	100.122455
100.009523	99.8550545	99.9156922	99.9709317
100.003668	99.8964931	100.224828	99.9244971

99.9541686	99.8720991	99.8884463	99.9745257
99.7173171	99.7854689	99.7260189	99.9646062
99.9185078	99.8494251	99.783864	99.8720246
99.7043656	99.7011843	100.018388	100.071133
99.7625584	99.6072046	100.201355	99.8162455
100.037554	100.055836	100.121923	99.8066136
99.9374914	99.8871108	99.8062895	99.811789
99.7517359	99.8245619	99.8471583	99.7210762
99.9979905	100.048331	99.8037745	99.950949
99.8486055	99.8356644	99.9882082	99.9009204
99.6571728	99.7604493	100.194019	99.5528766
99.8313961	100.020965	100.045425	99.8234336
99.8716697	99.85568	99.8526075	100.043243
100.05299	99.9856253	99.8029361	100.159114
100.06807	100.259746	99.9387464	100.176653
100.033474	100.043639	99.8999734	99.9348478
99.9850391	99.9337097	99.9848548	99.8536232
99.8587183	100.051927	100.055694	100.051869
100.070909	99.9434048	100.080844	100.001696
100.030635	99.8800741	99.8243137	100.111961
99.8491378	99.9222945	99.9731181	100.191029
100.163698	100.097431	100.064706	100.111961
99.9509751	100.092897	99.9546748	99.9847327
99.8746858	99.8605275	99.5262856	99.7065564
99.9499106	99.9102539	99.5264952	99.8299028
99.7591874	99.8428575	99.6547604	99.8392472
99.9101692	100.080387	100.06764	99.8968951
99.9534589	100.191411	100.090485	100.060638
99.8122351	100.06084	100.03264	99.9830075
100.069844	100.266626	99.8781767	100.066389
100.2336	100.257557	100.298811	100.044681
100.203262	100.242858	100.203451	100.19376
100.100892	100.188284	99.8232658	100.099453
99.8888792	100.086954	99.9846452	99.8199833
100.13194	100.097431	99.8674879	99.8812252
100.000652	100.074601	100.069107	99.8730309
99.8546377	99.8011061	99.9341356	99.6730603
99.9179755	99.7673297	100.075185	99.6169937
99.6694145	99.8314423	99.732516	99.8475853
99.6686273	99.9793096	100.00591	99.9879525
100.100782	100.22987	99.7624205	100.221793
99.8705125	100.280672	99.8375017	100.23865
99.8987196	100.431353	99.6942601	100.168772
100.086767	100.522608	99.8565866	100.20897
99.9489248	100.479489	100.153556	100.135346
100.075413	100.274713	100.030867	100.128574

99.9569079	100.309993	100.047436	100.060857
99.7979545	100.09283	100.017026	100.050771
99.73267	100.089851	100.313366	100.026422
99.7780853	99.9192567	100.298895	100.114454
99.8071794	99.8662596	99.9654334	100.379992
99.8479822	99.699115	99.7743747	100.213148
99.8433697	99.5689742	99.9390082	100.22698
100.237205	100.045948	100.182288	100.374373
99.8006155	99.7933494	100.173899	100.257669
99.7631834	99.5616048	99.4211993	100.028007
99.4378255	99.3886587	100.054147	99.7062777
99.8353866	99.7303174	99.9824211	99.9679255
99.9757127	99.8722178	100.166978	100.15422
100.071688	99.9829159	100.058551	100.271068
100.080203	99.8621829	100.07428	100.255796
100.038691	100.06837	100.382784	100.017345
100.093331	100.078875	100.134261	100.17223
100.414964	100.07668	100.270372	100.197011
100.058383	100.123876	100.14328	100.231014
100.376822	100.161977	100.330353	100.339218
100.147084	99.8115378	100.313785	100.081028
100.019354	99.995146	100.136359	100.144711
100.019354	99.8612421	99.6642696	100.363423
99.8117919	99.5689742	99.7479495	100.302189
100.034256	99.8231407	100.123356	100.411978
100.175114	100.090321	100.305396	100.19874
100.031417	100.082952	100.093575	100.066332
100.147794	100.312188	100.046387	100.251041
99.9466185	100.034972	100.179562	99.959713
100.045432	99.9982819	100.141392	99.9994789
100.026628	99.9586125	100.100706	99.9721038
100.177953	100.021958	99.9325068	100.049618
100.08109	99.882096	100.1502	99.9280156
100.10025	100.018822	99.8194654	100.026566
100.070269	99.9694314	100.168656	99.9765703
99.989905	99.9063994	100.135729	99.8904109
100.131828	99.9909125	100.029399	99.8277364
100.392433	100.288982	99.7542412	100.052644
100.338148	100.222814	99.4530774	99.808718
100.120651	100.325202	99.6170816	99.866926
100.183275	100.243197	99.5753465	99.9291682
100.125796	100.415203	99.6273581	99.9891052
100.265235	100.353425	99.7691316	99.9138958
100.188774	100.320341	99.685242	99.9749854
100.195693	100.306857	99.6441361	99.8480516
100.102379	100.304661	99.9838892	99.817795

100.220352	100.36691	99.8341461	99.8994879
100.03603	100.085931	99.9887128	99.7600192
99.6225024	99.8389771	100.049113	99.5506721
99.5329137	99.6757523	100.117274	99.7950305
99.5504767	99.7215368	100.214376	99.9056833
99.9459089	100.005024	100.161106	99.987088
99.9091864	99.8179664	99.9750808	99.9294564
99.8941071	99.8214159	99.8660242	99.7816311
100.052528	100.002515	100.144118	99.861451
100.051287	100.056924	100.095253	99.9875203
99.6817552	99.6561528	100.514282	99.819668
99.9711002	99.9125144	100.094204	100.035066
99.9964689	99.8504232	100.335387	100.23721
100.115507	99.8377227	100.308332	100.113157
100.322891	100.067115	99.8972731	100.203351
99.7009148	99.6867281	100.109304	99.6803434
99.9499892	99.7754747	100.407322	100.041118
100.331761	99.9286644	100.189419	99.9559669
99.8710447	99.7833145	100.316512	99.7763002
99.8811567	99.6738708	100.057083	99.6637743
99.9322489	99.6925295	99.6028204	99.7568495
99.7651348	99.57603	100.079523	99.8117436
99.9042191	99.7958581	100.001716	99.9118787
99.9608108	99.907497	100.046597	99.9405505
99.9519407	99.9656683	100.076168	99.7873943
99.8087761	100.051122	100.174319	99.8529503
99.7417176	99.9010683	100.605931	99.6173808
100.031063	100.089224	99.7712289	100.041262
100.100073	99.8852319	99.9044037	100.065179
99.9883084	99.8381931	99.8412768	99.9831979
99.8823985	99.5697582	99.5910758	99.6830809
99.774892	99.8077747	99.5468241	99.8242785
100.051819	100.01255	100.230105	99.6725631
99.9879536	100.080129	99.9002092	99.8914194
100.005694	100.032777	99.871477	99.8643326
100.036385	100.010669	99.9872448	99.9468899
100.195161	100.18628	99.9769683	100.035499
100.034256	100.2098	100.02877	99.9803163
100.199064	100.382432	99.9952143	100.09241
99.9934531	100.257936	99.9392179	100.036363
100.162519	100.365185	99.6472819	100.083621
99.9400546	100.467886	99.530885	100.001928
100.046851	100.421004	99.8523921	99.9807486
99.8854144	100.518218	99.8257572	100.2277
100.261864	100.656826	99.7043269	100.171654
99.9542469	100.209329	99.7678733	99.9515005

99.8737057	99.8684547	99.8666534	99.9790196
99.7757303	99.7827872	99.8316236	99.9457296
99.7754538	99.798335	99.6716616	99.9514548
99.8972458	99.8867191	99.6795187	100.004047
99.9208853	99.6788553	100.196273	100.041061
100.074611	99.9148732	100.368322	99.9413358
99.9996838	99.8365741	100.261345	99.902724
99.8321334	99.8643079	99.8501583	100.038931
99.7034292	99.9288802	100.168068	100.157828
99.6867018	100.077915	99.666625	100.305618
100.027747	100.110691	99.6358011	100.113225
99.9562755	99.7890903	99.8862203	100.04612
100.062723	99.9033875	99.5556186	100.021755
99.9175675	99.9080098	99.9003227	99.9936615
100.215758	100.06895	100.318964	99.9943272
99.9056786	99.9057687	100.27444	100.08846
100.007978	99.8474995	100.112665	100.057171
100.080141	99.7260588	99.7224304	100.167947
100.261239	99.8928822	99.7318992	100.05584
99.790937	99.8427372	99.9053593	100.038265
99.9253091	99.8413365	99.6823392	100.09565
99.9637406	99.8007162	99.7302875	100.047452
99.936645	99.7459488	99.7145733	100.149973
99.7760067	99.7105111	99.7508368	100.047319
99.8532845	99.8320918	99.8463305	100.082069
99.961114	99.876494	99.8999198	100.050381
100.210228	100.163918	100.028655	100.039064
99.9413453	99.8486201	99.7941514	99.9446644
100.154792	99.8047782	100.189221	100.022953
100.362572	99.7868492	100.33488	100.182593
100.097283	99.6357137	100.753722	100.158361
100.167925	99.8676696	100.629621	100.117885
100.336167	100.419685	100.356436	100.134661
99.9539254	100.006199	100.422315	99.875962
100.065211	100.215744	99.9688203	99.9428004
99.9983014	99.9658587	100.119515	99.8035316
100.015305	100.371641	99.8151036	99.8891433
99.6536617	100.003538	100.028051	99.9157722
99.8480313	100.213643	99.8557993	99.9258911
100.098389	100.105929	100.023417	100.072749
100.007287	100.066709	99.6235118	100.000718
99.9797768	100.042617	100.069351	99.8940697
100.025812	100.203838	99.9962193	100.049316
99.9703763	99.9179547	100.217426	99.9832762
100.088574	100.150191	100.44508	99.9037892
99.8915778	99.9947131	100.878025	99.8951348

100.227785	100.066429	100.046787	99.9594434
100.187279	100.140106	100.135229	99.9931289
100.090648	100.080996	100.032281	100.000851
100.099633	100.129601	99.970432	100.074214
100.025397	100.241236	99.9033447	99.9647692
100.101845	100.361837	99.9009271	99.9059195
99.977012	100.201877	99.8894437	100.016296
99.9398246	100.235073	99.8042246	99.984874
99.6688685	99.9073094	99.7881075	99.9818117
99.8362807	100.316314	99.9921901	100.123876
99.853561	99.8361539	100.057867	100.010837
100.114011	100.214343	99.7921368	100.047452
99.7469757	99.9923319	99.8360558	99.9527862
100.208984	100.224428	99.6936211	100.119216
100.061202	99.9151533	100.299422	99.8304267
100.167787	100.180726	100.411637	99.9768853
100.124241	100.149631	100.510959	100.058236
99.9356773	99.9783249	99.9645896	99.9742224
99.859229	99.770461	99.653127	99.9981884
100.221564	100.158875	99.7623202	99.9358769
99.9323595	99.8358737	99.9277217	99.8422766
99.9723117	99.9668392	99.7401592	100.059035
99.7833336	99.8353134	100.248049	99.9617069
99.7891398	99.8776146	99.7609099	100.056772
99.8722238	99.9340628	99.9607618	99.9898003
99.8447135	100.047379	99.8666783	99.9384067
100.155345	100.28872	99.6607826	99.9206985
99.9753531	100.193472	99.8175212	99.9567806
100.109863	100.370381	100.523248	99.984874
100.024568	100.016284	100.413853	99.9148401
100.373907	100.207199	99.7919353	100.092188
100.033968	99.8759337	99.7675582	99.9518542
100.224882	100.245298	99.8688944	99.9342792
99.8972458	100.02917	99.8862203	99.976619
100.164193	100.200336	99.8555978	100.073814
99.9529577	99.9560538	99.9555237	100.027214
100.001481	99.9580148	99.8171183	100.07195
99.7158711	100.004238	99.9406154	99.7466791
100.157557	100.326119	100.19184	99.968231
100.000375	100.110131	100.664877	99.9807465
100.063829	100.025949	100.181163	99.9880694
99.8073879	99.8115015	99.8529788	99.9104464
99.9598698	99.996534	99.6239147	100.030276
99.8279861	99.770321	100.119918	99.8621151
99.7413078	99.6358538	99.6841523	99.9603754
99.9203324	99.7609363	99.7145733	99.9634378

100.037424	99.8220068	100.47812	99.9570468
100.155898	100.018245	100.326418	100.007642
100.026365	100.038135	100.066933	100.063695
100.112767	100.135764	99.8316236	100.052511
99.9800533	99.7610763	99.8302134	99.9221631
99.9290417	99.7486101	99.986952	99.9491913
99.9648466	99.5948132	99.9255056	99.8788912
100.101431	99.8910613	99.8529788	99.8177781
100.15344	100.424298	100.407201	100.0413
99.9756238	100.31334	100.289474	99.8353201
100.307078	100.566296	100.356488	100.073143
100.267978	100.478653	100.047381	99.9837426
100.389423	100.588768	99.6821266	100.122572
100.178724	100.26053	99.696616	99.9147275
100.228325	100.361937	100.016389	99.9026032
100.022185	100.377948	100.057443	99.8383845
99.9196676	100.311655	99.7189539	99.9718848
99.8706196	100.131313	99.8791424	99.9909372
99.9669195	100.197747	99.5277741	100.004394
100.005605	100.378089	99.7783202	99.9841423
100.140176	100.755907	99.858012	100.09859
99.7772211	100.513205	100.240371	100.00546
99.8793239	100.380477	100.399554	99.9173921
100.054377	100.332161	100.175572	99.9814776
99.9077856	100.259125	99.7759053	99.8639654
100.010027	100.396348	99.8173611	100.007725
99.7556676	99.9724606	99.6903775	100.047029
99.8026432	100.354493	99.216453	99.988539
99.7613323	100.201961	99.7954258	99.9472365
99.9706499	100.376965	100.256471	99.8760897
100.054101	100.12106	100.265124	100.039168
100.236338	99.8855203	100.104131	100.204644
100.150262	99.7527922	99.9399174	100.03224
100.341757	99.6244182	99.9284466	99.9781468
99.9289246	99.4886001	100.008742	99.8423815
99.8956272	99.5567198	99.7006409	99.9100643
100.067088	99.6773689	99.9067125	99.9746827
99.9770054	99.6925379	99.8928268	99.8338545
99.9548993	99.8602387	99.8892045	99.7918859
99.966505	99.7054595	100.133713	99.8898127
100.156894	99.7933831	100.68572	99.9436392
100.000079	99.6984369	100.925801	99.8161344
100.244628	99.8067261	99.9749335	100.052491
100.150677	99.7433818	99.9006753	100.043431
99.8997721	99.7085495	99.981172	99.9945345
100.067779	100.123026	99.7696668	100.078605

100.01652	99.9209144	99.7187526	100.212105
99.9379052	99.74591	100.133512	100.157346
100.102596	99.9636121	99.6151131	100.222498
100.235647	99.9011105	99.6006237	100.160544
100.051476	99.8692277	99.8346679	100.025178
99.8038866	99.992405	99.7221737	100.034238
100.045396	100.036788	100.435174	100.175066
99.9912363	99.9883318	100.336163	100.043831
99.9759001	100.054626	100.254861	100.048894
100.107294	99.9954949	100.30497	100.087931
100.060042	99.8947902	100.561553	100.001862
100.107846	99.9869273	100.249427	100.183993
99.9287864	99.9335552	100.191872	99.9300494
99.9082	99.9279371	99.734047	100.05902
100.131196	99.8838348	99.8551946	100.128967
100.001599	99.6602337	99.9960639	100.026244
100.046225	99.9247066	99.8670679	99.9526991
100.051752	99.7273702	100.040538	100.053957
99.8322101	99.7397301	100.020615	99.9966663
99.8649549	99.8766717	100.058248	99.9558967
100.173059	100.182438	99.7308271	99.9892052
99.9399776	99.9976017	100.397139	100.02358
100.15344	99.9502691	100.144379	99.9496347
99.996901	99.7480168	100.164302	99.8309234
100.41678	100.203225	99.9153659	99.949768
100.181764	100.05589	99.7690631	100.047029
100.101076	100.041985	99.9316665	99.9999971
99.9160754	99.8506879	100.323484	99.8565043
99.868409	99.7773715	99.9487721	99.8928771
100.304729	100.063053	100.275589	99.8510417
100.29561	99.9557467	100.63722	99.8908786
100.282485	100.027799	100.020213	100.044231
100.141005	99.8043384	99.7626233	99.9590943
100.217548	100.006591	99.8757213	99.9204565
99.8702051	99.8852393	99.7950233	99.8210641
99.8834688	100.043951	99.7167402	100.063017
100.052443	100.314183	99.7547749	100.182661
99.9913744	100.238338	100.055631	100.133897
100.153716	100.383567	100.095075	100.163742
99.8689616	100.267271	100.046173	100.020648
99.9495109	100.098868	99.7684594	100.096059
99.8779423	99.8887507	100.309397	100.079138
100.029093	99.9810283	99.9690975	100.120973
99.8746263	99.8273727	99.9479671	100.011322
99.8480989	99.9039196	100.046173	100.138294
99.9348656	99.8341145	99.9630602	100.151617

99.9641562	99.9100995	99.9940515	99.978813
99.9296154	99.9140322	100.144178	99.9832097
99.8065117	99.902796	100.326905	99.9588279
99.8881663	100.086087	100.757563	99.9279176
99.8335918	100.099851	100.377216	99.9383099
100.039593	100.138617	100.278607	99.8950089
99.9607022	99.9995681	100.236347	100.002928
100.057831	100.320784	100.039935	99.9128622
100.031718	100.131453	100.041343	99.8427812
100.022461	100.205332	99.9427348	99.978813
99.8432632	99.850407	99.7517563	99.9718848
99.9164899	100.067688	99.6310112	99.8201314
99.8135581	99.936926	99.605856	99.9491018
99.8932784	99.9199312	99.4901419	99.9896049
99.723061	99.8678232	99.6515379	100.158812
99.8156305	100.11474	99.641677	100.097391
99.7561404	99.9093007	99.8638915	100.089111
100.064793	100.177824	100.008895	100.066584
99.7787989	99.7949401	100.309174	100.1327
99.8600379	99.9248571	100.52386	100.348644
100.051806	100.321195	99.5180978	100.309321
100.061478	100.299472	99.6095307	100.328649
100.068386	100.061501	99.8036747	100.232807
100.29428	100.130734	99.5392442	100.375437
100.214284	99.9690037	100.043334	100.163625
100.070182	99.9290616	99.9968116	100.082046
99.8998285	99.8341815	100.123891	100.220944
100.011463	99.9073387	99.9164554	100.136299
99.9287042	99.9394325	99.9116219	100.198416
99.9713962	99.980636	99.6810256	100.254535
99.9570274	100.020858	99.80589	100.05432
100.049181	99.9499436	99.831467	100.034325
100.186514	99.9858215	99.9243097	100.131633
100.017957	100.067388	100.334952	100.099375
100.258634	100.257148	100.438066	100.111105
100.240949	100.251682	100.72465	99.9728744
100.254075	100.447749	100.756067	99.9355507
100.045865	100.090792	100.815479	99.9095574
100.019753	100.244534	100.106974	99.9596778
99.9262173	100.020298	100.105363	99.9710082
99.8882229	100.109852	99.7416453	99.9580782
100.057609	100.145029	99.7420481	99.9758069
100.075708	99.8075534	100.040313	99.8350433
100.167586	99.9584926	100.052598	99.9254199
100.100992	99.805311	100.173636	99.9959351
100.342636	99.8567453	100.044945	100.056053

100.258911	99.8458138	100.196393	99.9420823
100.103341	99.8585672	100.076362	99.861703
99.7739632	99.6368533	99.8912811	99.9159557
99.9830018	99.8615103	99.7368118	99.8879629
99.9567511	99.7037038	99.7285546	99.8489063
100.14921	99.932285	99.7519164	99.9246201
100.154875	99.9352281	100.448941	99.8151817
99.9870084	99.9447581	100.186726	100.010731
100.197429	100.020438	100.036083	100.165758
100.025141	99.8809909	99.8469744	100.002067
100.095465	99.9254177	99.9921795	99.9466145
99.9133683	99.9603146	99.9486784	99.9150226
99.8760647	100.05043	99.9049759	99.9199547
99.913092	99.9540079	99.9899642	99.9236871
100.080958	100.086027	99.5106462	100.044189
100.079991	100.002779	99.7519164	100.008465
99.8938875	100.042301	99.88101	99.95088
100.117847	100.063183	99.9802973	100.039124
100.063274	100.112375	100.001645	100.074448
100.054293	100.221971	100.397183	100.008199
99.7801805	100.091353	100.301521	99.9503468
99.9979232	100.248038	100.348647	99.9863376
100.006351	100.100883	100.103148	100.106173
100.250206	100.320775	100.074953	100.090844
99.990048	100.070751	100.155913	100.010198
100.099886	100.143488	99.8826211	100.123236
99.8148591	99.9869426	99.9132331	99.9530128
100.029148	100.270602	99.5903983	100.115638
99.8839399	100.101023	99.9061843	100.046322
100.08082	100.330865	99.7396313	100.139631
99.8916769	100.025904	100.026014	100.031659
100.011049	100.146711	99.9599565	100.017663
99.8137538	99.9299025	99.9446505	99.8566377
99.8654262	99.9093007	99.6012736	100.078447
99.8666697	100.020298	99.7724586	100.229208
99.8469126	100.080001	99.5269591	100.128967
99.7178696	99.7287903	99.756347	100.01433
99.7091654	99.7157566	100.076564	99.9220875
99.8281226	99.7922773	99.9237055	100.014464
99.8462218	99.7139346	99.7015678	99.9315517
100.137052	99.8456736	99.9394143	100.01673
100.029839	99.7585016	100.58307	99.939283
100.087728	99.9119635	100.511172	99.9743407
100.005937	99.8434312	100.526881	100.057919
100.087037	100.071452	100.607237	99.9634101
100.14396	100.105508	100.284201	100.078714

99.8109905	99.9104219	100.130537	99.8834308
99.8831109	99.9719468	100.309174	99.978073
99.8800714	100.097379	99.8238141	100.01673
99.9821728	100.112235	99.9895614	100.096176
99.9737449	100.213001	99.88101	100.060052
100.193422	100.206975	99.7913896	100.167757
99.8927822	100.1547	99.7329853	100.039391
100.051392	100.356372	99.8290503	99.9863376
99.9539878	100.156241	99.6818312	100.046189
99.7504758	99.9224746	99.7023734	99.9414158
99.9176513	99.8184849	99.7134501	100.025661
99.7933059	99.9118234	99.586169	99.9242203
99.7314095	99.6672654	99.7215059	99.895561
99.9112959	99.9589131	99.5901969	99.7285374
99.8554786	99.9088803	100.177261	99.8910288
99.8890519	99.9031342	100.581862	99.9306186
99.9816201	99.961716	100.370599	99.8307777
99.9749884	99.9865222	99.8522106	99.9387498
100.054984	100.023661	99.7158668	100.027394
100.073221	100.046786	99.6304757	100.052454
99.8565839	100.040059	99.9823112	99.6808164
99.9744357	99.841329	99.9700262	99.8218467
99.9637973	99.8386662	99.6514207	99.8763659
100.000825	99.9001911	100.197803	99.7627952
100.102097	100.016654	100.716393	99.8250458
99.976875	100.082036	100.545188	99.6289494
100.120345	100.172886	100.088425	99.6865798
100.083717	100.099561	100.10071	99.7581852
100.049577	100.09157	99.7839166	99.7617788
100.160843	100.174148	100.015722	99.7777502
99.8343728	100.249015	99.9097881	99.7877324
99.7015457	100.112039	99.8229871	99.86373
100.04709	100.132368	100.000818	99.9812533
100.127532	100.092832	99.9369764	100.040481
100.147159	100.235415	99.9623521	100.038884
100.224146	100.385429	99.8654813	99.9715374
99.8541379	100.212983	100.181469	99.9761957
100.095327	100.151015	100.298278	100.035423
100.093392	100.003104	100.400989	100.207915
100.259668	99.7507438	100.724429	100.094518
100.22	99.6416682	100.589696	100.039549
100.216959	99.668867	100.383871	99.9773936
100.122418	99.5219375	100.378634	100.018653
99.9951197	99.5855882	100.249742	99.9281482
99.9380359	99.3967389	100.168983	100.056452
100.039349	99.7250872	100.185094	99.9015291

100.138866	99.6032534	100.045528	100.067632
100.025942	99.736864	99.7968058	100.107295
99.9689966	99.6605952	100.183685	100.03742
100.29671	100.016283	99.8415154	100.060046
100.182543	99.7759798	99.9955822	100.035689
99.980054	99.7315364	99.9768525	99.9079177
100.037553	99.8652872	99.8211746	100.068431
99.9446704	99.7686894	99.7571311	100.078812
100.075839	99.9395932	99.716248	100.081607
100.045154	99.8512672	99.920462	100.095982
100.105832	100.121433	99.9629563	100.196602
99.9170268	99.8724374	99.7543116	100.216433
100.034235	99.8982342	99.6379056	100.136842
99.9949815	99.9446404	100.043313	99.8529492
100.271831	100.150454	100.339564	100.029833
99.9369302	99.7238254	100.168983	100.125795
100.179087	100.044182	99.5666119	100.129788
100.022901	99.9729608	99.7180606	100.062841
100.129329	100.181859	100.005048	99.9908362
99.8303645	99.8010756	100.146427	99.8393735
99.8063146	99.7765406	99.9808804	99.738487
100.219447	99.8948694	99.8473558	99.9707388
100.08662	100.027499	99.7216857	100.036355
99.8627074	99.9073472	99.8139244	100.033294
99.924767	100.062268	99.6930877	99.9820519
100.003689	99.9136562	99.9975961	99.8771726
100.101547	99.9551554	99.8302373	99.8546795
100.078741	99.869353	99.9780609	99.9330728
99.9778426	99.9855788	100.049757	99.8734459
99.9843388	99.9006176	100.128503	99.9583609
100.129053	99.9868406	100.31439	99.9204287
100.164298	99.9833356	100.15408	99.8951405
100.09049	99.9841768	100.054994	99.8071644
100.039902	99.928237	100.477721	99.9007305
100.200649	100.008151	100.443484	99.9301447
99.924767	99.7455564	100.713352	99.8167472
100.224699	99.8219654	100.357488	99.9719367
100.046813	99.830097	100.18308	99.9377311
99.8938063	99.9735216	99.8676967	99.9037917
99.9026522	99.9279566	100.232019	99.919497
99.9626386	99.9930094	99.7867361	100.061244
99.7547595	99.866549	99.9504698	99.9976241
99.8931152	100.00058	100.092654	99.9666128
100.064781	99.9208064	99.8644744	100.069895
100.075839	99.9824944	99.5907793	100.043276
100.043358	99.949968	99.9649702	99.8910146

99.9235231	99.6713906	100.205234	99.9574292
100.189177	99.9993184	100.45738	100.016391
100.047366	99.9749236	100.342988	100.053524
100.11454	100.136294	100.278541	100.284046
99.9052784	99.9980566	100.580835	100.17371
100.15172	100.335238	99.9714148	100.096514
99.664227	99.9700166	100.302709	99.8770395
99.8909037	100.136154	99.7645827	99.8828957
99.8982293	100.013338	99.6373014	100.106896
99.7660933	99.947865	99.9601367	99.8452297
99.7691341	100.283504	99.9075728	99.8890181
99.9753546	100.73677	99.5672161	100.067499
99.7649876	100.657417	99.6955044	100.035423
99.9560042	100.795093	99.6711357	100.056719
99.6041024	100.345052	99.8511823	99.7640414
99.8642278	100.344771	99.4675258	99.9119106
99.6709997	100.325283	99.9436224	99.7048139
99.9177179	100.315329	100.083592	99.8433663
99.8805374	100.047547	100.31298	100.001085
99.8350639	99.6202176	99.8813915	100.030499
100.122418	99.8051414	99.9436224	100.016524
99.9376213	99.6312934	99.8022435	100.124863
99.9572481	99.8033188	99.6143424	100.161731
99.8946356	100.076849	99.7347763	100.024643
99.8736265	100.473335	99.8427238	100.055121
99.7145382	100.375335	99.8304387	100.204588
99.7528244	100.261913	99.8902529	100.001883
99.8531704	99.9607634	100.085807	99.9643502
99.9947051	100.034088	99.9083784	100.128324
99.9847534	100.075026	99.579904	100.221225
100.009771	100.24579	99.5935988	99.9164358
100.158355	100.468708	99.7927779	100.080543
100.298784	100.662464	99.8608493	99.9572961
99.8295697	99.4836364	100.001231	100.103655
99.9659443	99.646722	99.8556583	100.221906
99.9409107	99.6605716	100.138324	100.106936
100.178584	99.8835777	100.334423	100.235713
99.9186425	99.63047	100.120481	100.015206
99.8535844	99.8010457	100.18514	100.095863
99.8464527	99.5736585	99.844175	100.053621
99.9493523	99.8291687	99.9897477	100.179937
99.8601339	99.6813459	99.914488	100.217258
100.132446	100.10079	99.8183818	100.250478
100.069863	99.7806953	100.080554	100.095043
100.20158	99.9778395	100.32718	100.071393
100.114108	99.9970593	100.001231	100.111858

100.164612	99.8974273	99.9872744	100.059226
99.9780244	99.7640193	99.9625412	100.135918
100.09999	99.9728932	99.7768653	100.191011
99.8895337	99.7842283	99.8498283	100.27112
99.8397577	99.7059359	99.7459487	100.168591
99.9986917	99.8444315	100.156344	100.209329
99.6148924	99.4645579	100.158287	100.057722
99.7444265	99.805144	99.9445213	100.089848
99.558858	99.5958461	99.7441821	99.9773388
99.7061484	99.9036454	99.9899244	100.10967
99.4354368	99.684455	99.8858682	99.9823969
99.5668629	99.9521189	99.8729715	99.9252537
99.543867	99.8800447	100.098574	99.7772009
99.6065964	99.8387786	100.128077	100.130723
99.5606046	99.8784901	100.238847	100.092309
99.968273	99.9138206	100.081791	100.358202
99.8885149	99.8803273	99.8070751	100.089711
100.096643	100.358561	100.171714	100.31596
99.9889402	99.9237132	99.8623716	100.068522
100.131864	100.177528	99.8035418	100.00632
100.510133	100.326481	99.8337517	100.046375
100.172908	100.035075	99.7464787	99.9335928
100.337081	100.032672	99.7318154	100.080689
99.9224267	99.7024029	99.7512487	99.8608651
100.127207	100.243384	99.5858894	100.021221
100.285995	100.207912	99.5666328	99.9721439
100.136813	100.135696	99.8332217	99.9439825
100.400975	100.416644	99.9007081	100.079185
100.233891	100.064753	100.276123	99.8809609
100.395153	100.321817	100.213937	100.097504
99.9291217	99.9922544	100.260047	99.992103
100.207838	100.47176	99.931448	100.170094
100.283812	100.184594	99.7166222	100.030244
100.037406	100.177104	99.9761445	99.7802084
100.117601	100.115346	99.7171522	99.9357801
99.9329058	99.9104289	99.6268759	99.9162311
100.114399	100.357289	99.7629087	100.055535
99.8352459	99.7146979	100.056174	99.8519792
100.027509	100.029139	100.220827	99.7826692
100.242041	100.120433	100.028084	99.9643517
99.9231544	99.7830978	99.8653749	99.8193064
100.160682	100.202683	99.8392283	100.062233
100.019068	99.9399652	99.7134422	99.9955207
100.07845	100.330721	99.6952456	100.149725
100.222393	100.123119	99.7199788	99.9312688
100.135648	100.23335	100.330536	99.865103

100.341448	100.381314	100.3081	100.023682
99.9308682	100.215967	100.185317	99.8187596
99.8131232	100.135979	100.022254	99.9646251
99.8694487	99.7299607	100.187614	99.8605917
100.168832	100.122412	100.093804	100.228605
100.224576	100.170037	99.9279147	100.004133
100.249173	100.338069	99.9577712	99.8715282
100.134484	100.103616	100.052287	100.035439
99.8906981	99.5737998	99.8941715	99.7797983
100.071027	99.9230066	100.133907	99.9605239
100.058365	99.8882413	99.9572412	99.8280556
99.9337791	99.9976246	99.9296813	99.7290803
100.240149	100.230523	99.9909844	100.087524
99.8873506	99.7650085	100.105287	99.7788414
100.095915	100.136968	100.087797	99.9979814
100.254412	99.9931023	99.9477012	99.9379674
100.179312	100.017834	100.104757	99.8821913
100.358185	100.243101	99.7793386	100.021768
100.040608	99.8994058	99.7524854	99.8199899
100.115418	100.160569	99.8998248	100.230382
100.151804	99.9025149	100.207577	99.9833538
100.006697	99.9979072	100.24821	99.9024238
100.026927	100.07521	100.019428	99.8782268
99.8297152	100.088919	100.158287	99.7700922
100.220209	100.300054	100.31022	99.9145906
99.8380112	99.9914064	100.361276	99.710625
100.067534	99.9991791	100.234253	100.069889
99.7893995	99.6816285	100.455439	99.8506121
99.9291217	100.022497	100.143447	99.990736
99.5182513	99.7391466	100.302623	99.7983904
99.8908436	100.103051	100.019604	100.055535
99.8866229	99.9280942	100.14168	100.030791
99.7010544	100.069981	100.235667	99.9847209
99.7994421	99.9511296	99.931448	99.9923764
99.6140192	99.917071	100.082674	99.8426832
99.7247781	99.9899932	99.9968144	100.018897
99.822438	99.8212547	99.9839178	99.9119932
100.025035	100.216815	100.352796	100.09559
100.040463	99.9645552	100.264287	100.024229
99.925192	99.8724133	100.100341	100.075494
99.9889402	100.001299	99.9895711	100.022452
100.079307	100.279264	99.9318347	99.9459699
100.149035	100.371491	99.7134789	100.09764
100.078432	100.228468	99.6637403	99.9916486
100.355448	100.448252	99.728471	100.309622
100.118985	100.227901	99.8785686	99.9668946

99.9856557	100.303669	99.8607544	99.8557064
100.102209	100.189449	99.8385308	100.033088
100.094624	100.010671	99.9928614	99.8443551
100.225911	100.187037	100.092339	99.8505095
100.292722	100.304946	100.016672	100.06509
100.098854	100.035927	99.8238914	99.9037102
100.058885	100.170294	99.8933843	100.009291
100.305705	100.178666	99.8335922	99.9794768
99.9675672	100.142626	99.876452	99.7990866
99.9462695	100.075229	99.7166537	99.9945207
99.9805501	99.9134775	99.7619828	99.9221732
99.9917824	100.017339	100.024609	99.8568005
100.225619	100.119357	100.042776	99.97934
99.9932411	99.8293381	99.7803261	99.8170026
100.09404	100.057635	99.6949592	100.119932
100.1273	99.9715096	99.6259954	99.9823488
99.9862392	100.004711	99.6919607	99.8224731
100.12059	100.144187	99.9392426	100.089708
100.267923	100.143619	100.254253	100.034319
100.168437	100.135532	100.081932	99.9668946
100.35924	100.320411	99.7972584	99.9886399
100.108044	100.29927	100.247198	100.038148
100.412047	100.431368	100.300641	100.215529
100.13153	99.9581721	99.9716961	100.269824
100.029564	99.902694	100.091633	99.9813914
100.283532	100.219529	99.9972709	100.365695
100.126133	99.9818674	100.004679	100.128411
99.9693177	99.9774688	100.226562	99.9496625
100.158517	100.226766	100.122499	100.324529
99.5367995	99.648857	100.235205	99.9030264
99.7443791	99.9302202	99.9533528	99.8555697
99.7083481	99.8158588	100.318808	99.9299686
100.027813	100.001732	100.625529	100.151935
99.9112596	99.713274	100.321983	100.011069
99.9430603	99.8896404	99.9519418	100.217991
99.8454702	99.6641808	100.124439	99.9065822
99.8976933	99.763786	100.369957	100.00847
99.9630451	99.8667965	100.233794	99.9685357
99.937517	99.7874812	100.289705	99.9303789
100.072597	99.8081968	100.210688	99.997803
100.060343	99.8148656	100.134846	100.063039
100.162747	99.71824	99.876452	99.9350289
99.9786537	99.9385916	100.018613	99.8399787
99.9535633	99.9828606	100.011028	99.9415935
100.000972	99.7226386	100.24226	99.7445183
100.061073	100.235137	100.024433	99.9972559

99.9618781	99.8009606	100.117031	99.854202
100.172813	100.303101	100.083872	100.113641
99.9855098	99.9147545	100.272244	99.8373802
99.857286	100.019751	100.392005	99.8042836
99.91549	99.9864078	99.9907449	99.9201217
99.923659	99.9757662	99.8602253	99.8399787
100.19659	100.436901	99.8217749	100.261892
99.9560431	100.061183	99.935715	99.8670577
99.9052788	100.10531	99.9785748	99.797172
100.2157	100.362694	100.039072	100.018864
99.9804042	100.186044	99.9403008	99.8691092
100.207969	100.579498	99.9628772	100.128275
99.7802642	100.1422	100.129907	99.9149247
100.194256	100.534378	99.9055544	100.047995
99.8743534	99.977327	100.015438	99.7434242
99.8969639	100.178098	100.159362	100.143455
99.6641481	99.828203	100.134493	99.8105747
99.9544385	100.033798	100.303992	100.167389
99.8711441	99.7836503	100.286531	100.068646
99.9308068	99.9926508	99.976811	100.171492
99.7566326	99.5923857	99.9519418	100.003821
100.108482	99.9983263	99.9732835	100.186399
100.147722	99.9401524	99.9223103	99.9839899
100.110524	99.8551617	100.015791	100.166568
99.970193	99.7425028	99.7805025	99.8492786
99.9487494	99.9458279	100.153189	99.9028896
100.039338	100.085587	99.8783922	100.01777
99.8750827	99.9805904	100.15072	99.8661004
100.033794	100.140214	100.009088	100.075074
99.8393434	99.9024103	100.090928	99.9359862
99.9392675	100.189591	99.9815733	100.064953
99.8246101	99.7278884	99.9695796	99.9544492
100.024021	100.087574	100.141724	100.190775
99.9122807	99.7911703	99.8480552	99.9696298
99.7559032	99.679079	99.8311229	99.9705872
99.9362042	99.8537428	99.8163072	100.029258
99.8742075	99.7687521	99.8953245	100.081228
99.5384042	99.5518058	99.6833182	99.920532
99.8962345	99.9327742	99.9325402	100.169577
99.8532015	99.7657724	99.9334221	100.078767
99.7661144	100.060331	100.020553	100.031857
100.07887	100.393767	99.6736174	100.096819
99.8585989	100.075371	99.8021969	99.9997177
99.7588207	99.7364017	99.5711419	99.9693563
99.9471448	99.8638168	99.8910914	100.179561
99.9941164	99.6346682	99.9392426	99.9298319

100.303225	100.142484	99.7131261	100.138395
100.03219	100.107722	99.4385057	100.122257
99.8802295	99.9260061	99.8210834	100.223959
99.8489075	99.8736091	99.8154276	100.031438
99.7405188	100.058273	100.252161	100.211097
99.8701773	100.06238	100.34831	100.148428
99.7703839	99.9893074	100.552272	100.164985
99.9036846	99.9833596	100.172096	100.109705
99.6812254	99.7574858	99.9661899	99.8710724
99.8926126	100.141542	99.719279	100.020355
99.7387705	99.9799609	100.08178	99.8981648
99.8442456	99.9488059	100.113417	100.049089
99.7380421	99.6746418	100.082134	100.028154
99.9785661	100.154854	99.9313714	100.198235
99.6296534	99.9497972	99.8703948	99.9486554
99.9243717	100.174821	99.8751669	100.180447
99.5884249	99.8414627	99.8705716	100.056067
99.751445	99.9265726	99.852367	100.16088
99.8221017	99.8128568	100.450821	100.02446
100.016007	99.9238819	100.855564	100.093285
100.104728	99.9234571	100.821275	100.18852
99.8241413	99.6989994	100.245268	99.9293622
99.8653698	100.025985	100.081427	100.069203
99.9216037	99.9282719	100.01992	99.9813579
100.074572	100.121433	100.260468	99.9802633
100.228705	100.211783	100.303063	100.229021
100.085352	99.983218	100.552979	99.9866943
100.174365	100.197904	100.547853	99.922247
100.313056	100.083905	100.093269	100.235863
100.121919	99.9910068	100.080013	100.005714
100.224626	100.506481	99.9184691	100.195087
100.462673	100.545991	99.8587298	100.223685
100.35749	100.367133	99.8887762	100.033764
100.412995	100.58281	100.05085	100.369957
100.406585	100.358494	99.9851014	100.057983
100.272119	100.355804	100.22565	99.9412665
100.402943	100.416839	100.146822	100.376114
100.552706	100.234441	100.125789	100.157185
100.298196	100.436948	100.201259	100.012966
100.478699	100.380444	99.926776	100.073171
100.284939	100.33527	100.088496	100.075908
100.049805	100.131063	100.084255	100.15007
99.9309275	99.833674	100.246859	99.9975039
99.9220408	100.044537	100.022218	99.9258046
99.7952959	99.7542287	99.8361066	99.9379826
99.739936	99.6750667	99.9078645	99.8014256

99.8321539	100.017205	100.277612	99.8935126
100.01994	99.9823684	100.053501	100.069203
99.7014755	99.6170051	99.8986738	99.7795326
99.9319473	99.960135	100.049966	100.01967
100.114635	100.00446	99.9760875	100.086444
99.9399599	99.7952967	99.9960595	99.9210156
100.130223	100.160518	100.215045	100.031712
100.283774	100.16066	99.8507763	100.082202
99.9874528	99.930821	99.9000878	99.9245732
100.049223	100.0308	99.7624045	99.9750637
99.9315102	99.5902401	99.990934	99.752577
99.9042673	99.9421501	100.067464	99.9092481
100.232784	100.089995	100.063045	100.099443
100.032469	99.7889241	99.9900503	99.9905256
100.10196	99.8615719	99.8898366	100.121473
99.9076181	99.6400881	99.8475949	99.8947441
99.8612906	99.9267142	100.071176	99.7261687
99.9622495	100.134461	100.033706	99.9045959
99.8819777	99.9924229	99.7922742	99.9206051
100.226374	100.346457	99.9799759	100.193445
100.168829	99.9881745	99.7378371	100.128177
99.7473659	99.8829556	99.8364601	99.957002
99.7259504	99.7727801	99.8350461	100.017755
99.9645804	99.7996867	99.7786649	99.821266
100.024748	100.150181	99.8528972	99.9565915
100.106185	100.113786	99.7417255	100.11942
99.8547349	99.8226281	99.7376604	99.8464428
100.052282	100.208809	99.6289631	100.049636
99.9306361	100.066912	99.6535304	99.9498868
99.796607	99.9969546	99.9278365	99.9377089
100.05607	100.188416	99.7721254	100.268155
100.203648	100.060964	99.8212601	100.048131
100.029847	100.171423	99.7986369	99.8790086
100.20321	100.436523	99.7305906	100.057983
100.24575	100.385542	99.7109721	99.8690199
99.8902817	100.28613	99.6494653	99.8222238
100.042813	100.31077	99.8156043	100.19153
99.8690119	99.9109951	100.005604	99.9840945
99.9679312	99.9602766	99.9320784	100.091096
100.039608	99.6306	100.008255	100.065509
99.6242631	99.6795983	100.205501	99.9185526
99.7543587	99.682289	100.219994	100.085897
99.8263265	99.3915561	100.201259	99.8784612
99.7132759	99.5470479	100.28097	99.9539918
99.9336955	99.6267764	99.9792689	99.9601491
99.6870528	99.6289006	100.083548	99.75504

100.036403	99.8137065	99.9603573	99.9580967
100.025039	99.6283342	100.064459	99.8805137
99.8985857	99.7243482	100.046962	99.8896813
100.074135	99.9585773	99.914404	100.01926
99.9549653	99.7984122	99.9172319	99.8088144
99.7473659	99.8757333	99.9168784	99.699213
100.038588	100.130779	99.7883858	99.9193736
100.051117	100.055016	99.9656596	99.8084039
99.8978573	100.22297	100.061101	99.8417906
99.9925517	99.9710393	99.8274461	100.057162
99.8797925	99.7362438	99.6964791	99.8222238
99.5320991	99.8804404	99.4489008	99.6153954
99.7436369	100.0209	99.4016036	99.7329225
99.8845651	100.31172	99.8122775	99.9077098
100.253337	100.504658	100.314369	100.269311
99.876849	99.918066	100.261071	99.9875189
100.102509	100.03165	100.156771	100.179662
99.923	99.7143785	100.068706	99.7908661
99.6452202	99.6269626	99.9922892	99.7159767
100.127113	99.8902004	100.117415	99.9071632
99.7803248	99.5822645	100.186596	99.7005342
100.059706	99.8037747	100.498616	99.9672933
99.814101	99.4029063	100.664686	99.8714951
100.201945	99.9992581	100.372961	99.8556426
100.118232	99.9783236	100.239541	100.003918
100.201945	100.024861	100.246247	99.9257489
100.202964	100.008169	100.25966	99.9622369
100.173264	99.7054672	100.384609	99.9817792
99.7169945	99.3925805	100.515029	99.7356557
99.7835277	99.6780259	100.495793	99.9314886
99.8900974	99.7220168	100.314016	99.8593324
100.114884	99.9210365	100.346312	100.117482
99.7963394	99.496546	100.114238	99.8746383
99.9020355	99.6804306	100.659392	100.0251
99.8557389	99.4078571	100.379138	99.810545
99.7752293	99.7152272	100.553149	99.7841698
100.110953	99.7674221	100.526324	99.861519
99.8914076	99.5869324	100.826697	99.8601524
100.123473	99.9184904	100.757692	99.9868356
99.9183412	99.9525798	100.454319	99.9757662
100.144438	100.030236	100.29125	100.145497
99.9377043	99.7334742	100.027409	99.9026535
100.275757	100.206058	99.9585812	100.177475
100.206312	100.02288	99.9299911	99.9135862
99.7370855	99.8856741	100.133828	99.8654821
99.9407616	100.010857	100.018056	100.014031

99.8340464	99.7377177	99.8539273	99.918096
100.068296	100.26872	99.7880994	100.214647
99.9465851	100.027548	99.9024598	100.215603
99.4329544	99.902648	99.8549862	99.9328552
99.9435278	100.248351	99.9063424	100.15069
99.7632912	99.7826987	100.165418	100.051066
100.022145	99.9405565	100.103826	100.21902
99.7992511	99.4382688	99.890812	99.947751
99.9969582	99.9868106	99.6605029	100.072521
100.218541	99.9655931	99.9758764	99.9789094
99.8263303	99.7736459	99.9689936	99.8712218
99.9224177	99.6863715	99.8352202	99.766814
99.700252	99.7224411	99.6380897	99.6726557
100.032627	100.169564	99.5710265	100.067875
100.183164	100.364481	99.4476655	99.8344604
100.163655	100.951073	99.545966	100.141124
100.380143	100.971866	99.4596662	100.320831
100.00846	100.619091	99.3802493	99.9518508
100.177049	101.151083	99.5775563	100.14618
100.461235	100.996195	99.821631	100.102039
100.111681	100.764925	99.7436259	99.9381849
100.138177	100.862243	99.718742	100.03043
99.9743922	100.410452	99.9931716	100.004601
100.253773	100.323036	100.210068	100.1511
99.8464213	99.8138176	100.084236	100.006241
99.9358117	99.6486044	99.881282	100.129781
99.8662211	99.3763138	99.7296839	99.9038834
100.126094	99.9849717	99.6347365	99.9634669
99.7175769	99.6000872	99.7190949	99.9243823
99.8925723	99.8262652	99.6756803	100.027423
99.7590691	99.5508627	99.6883871	99.9310786
99.9761392	99.6094229	99.5999695	99.9014235
99.5939736	99.5873567	99.7446848	99.7706405
99.8717534	99.7603497	99.654679	99.9375016
100.127986	99.8322061	99.3377171	99.7236297
99.8842739	100.13406	99.5053751	99.6645928
100.364711	100.336899	99.8230429	99.9262955
100.330789	100.386547	99.6094996	99.8277641
100.599688	100.699717	99.5124344	99.9641502
100.254647	100.585567	99.9015774	99.8869376
100.328314	100.62786	100.271131	100.138938
99.9444013	100.088371	100.431024	100.112562
99.9621629	100.143395	100.261601	100.262888
99.6229454	99.7968437	99.7928645	99.9733064
99.7705705	100.05018	99.6675622	100.134291
99.6500245	99.762047	99.9031658	100.02469

99.9551747	100.032074	100.046293	100.196744
99.8033276	99.6733581	100.137534	100.057352
100.014574	100.016939	100.437024	100.196061
99.8686961	99.7323426	99.9825827	99.9823259
99.9742466	100.006189	99.9146371	100.229679
99.7581956	99.7599253	100.064117	99.9726231
100.034228	100.080167	99.935109	100.15766
100.114156	99.9728071	100.114238	100.266167
99.8790328	99.8262652	100.101885	100.243755
100.071353	100.008877	99.8158071	100.221616
100.063055	100.205209	99.8129834	100.353083
99.9398881	100.093747	99.6610323	100.15971
99.7924085	99.9846888	100.026527	100.041226
100.085621	100.087806	99.683975	100.095343
100.047622	100.118925	99.9938776	99.9774061
100.400234	100.33506	99.8031004	100.139211
100.221599	100.244673	100.258071	100.061998
100.488023	100.493483	100.158535	100.135521
100.334574	100.195449	100.276778	100.152057
100.547846	100.344505	100.708846	100.328614
100.419119	100.291137	100.481306	100.297835
100.067111	100.11885	100.363247	100.329383
100.603251	100.29453	100.59806	100.359546
100.036422	100.227435	100.321656	100.291372
100.271647	100.213862	100.421997	100.338771
100.20662	100.150623	100.222434	100.311224
100.241622	100.050212	100.563929	100.210117
100.288567	100.265379	100.181775	100.470656
100.03974	100.189955	100.163871	100.200422
100.545855	100.154017	100.46191	100.308146
99.974879	100.380751	100.258803	100.359084
100.177259	100.10759	100.112954	100.359854
100.055665	100.176536	100.111835	100.319688
100.044053	100.062243	100.324827	100.297528
100.255888	100.275559	100.113327	100.440955
100.063959	100.103888	100.278759	100.266595
100.152874	100.510314	99.9318553	100.35693
100.0341	100.130572	99.726697	100.085156
100.324067	100.688617	100.270926	100.297374
99.7127808	100.473605	99.7114034	100.303222
99.9163216	100.769131	99.7962643	100.47989
99.9277677	100.422088	99.6519075	100.34031
100.190861	100.97782	99.4594317	100.60285
100.91329	100.843013	99.7964508	100.52575
99.2172824	99.8260994	100.108851	99.9526552
100.005236	100.263991	99.9197323	100.277214

99.7691817	100.281574	99.738074	100.382168
100.021327	100.072423	99.7955183	100.123168
99.7569062	99.4610101	99.9432323	99.7027344
100.175102	99.4216785	100.435985	99.567617
99.2763375	99.546614	100.106426	99.5756194
100.375491	99.9093898	100.406517	99.8158451
99.747119	99.636074	100.056629	99.6167086
100.327716	99.5045061	100.440648	99.5734649
100.207284	99.7610095	100.297224	99.7664457
100.002914	99.6066139	100.644314	99.679035
100.513342	99.9598267	100.488394	99.9549636
99.8149659	99.8045056	100.239593	99.9137205
100.42393	99.7895442	100.544346	99.9355732
99.894093	99.8310351	99.998252	100.067613
100.025972	99.6124751	100.526628	99.9683522
99.9989323	100.077359	99.2259243	100.189341
99.8849694	100.317512	99.614606	100.240434
99.7693476	99.9436313	100.310279	100.050531
100.029621	100.088001	100.22374	100.128862
99.7895855	99.8436829	100.29797	99.8424684
100.433551	100.160957	100.431882	100.140404
99.7583992	99.7159711	100.371267	99.7362829
100.112729	100.262603	99.4614833	100.307684
100.314446	100.287435	99.8967919	100.299528
99.9148287	99.8662022	100.178232	99.9822025
100.186548	100.040341	99.8995895	100.076231
99.9196393	99.7219865	99.8773951	99.8863277
100.348452	99.9886698	100.011867	99.9121816
99.7943962	99.6186448	100.108851	99.7373601
100.563605	99.9442483	100.113514	99.9517319
99.8194448	99.7590044	99.887653	99.6744182
100.318261	100.02939	100.188676	99.9298792
99.9551387	99.7600841	100.048609	99.7567505
100.276292	99.7966393	100.186998	99.7124296
99.6339854	99.698696	99.8042842	99.6507188
100.334186	100.019981	100.071549	100.021753
99.4840254	100.045122	99.4922571	100.020829
100.203303	100.26723	99.6369869	100.166411
99.4058936	99.6022952	99.428658	99.6998104
100.20098	100.181009	99.6125544	100.030832
99.6281795	100.092166	99.4234358	99.9471151
100.373666	100.771291	99.3311145	100.418949
100.316436	100.52142	99.6893955	100.175183
100.156855	100.442294	99.6071457	100.105778
100.181903	100.184402	100.085538	99.7285882
99.7899173	100.009492	100.135895	99.6787272

99.7832819	99.5873338	100.480747	99.4589692
99.8244214	99.6491845	100.088335	99.5553057
99.5591713	99.4341721	100.098593	99.5696176
100.27397	99.7229119	99.9803473	99.8969463
99.7696793	99.5617297	99.9383831	99.6231721
100.400706	99.8085159	99.936145	99.8855583
99.747119	99.636074	99.651721	99.7790649
100.564102	99.8888757	100.065768	99.8537026
100.132636	99.8982844	99.528253	99.908796
100.554149	99.9374617	99.8145421	99.9942061
99.7091313	99.7688759	99.6125544	99.8161529
100.092491	100.14615	99.5946497	100.111934
99.3191358	99.3268201	99.6867844	99.54915
100.14375	99.761935	99.7216613	100.119628
99.5530336	99.7465109	99.5248959	100.029447
100.017843	100.117461	100.153799	100.068536
100.734466	100.495044	100.018022	100.342926
99.4395683	100.051138	99.7203558	99.908796
100.459761	100.225122	100.217398	100.037142
99.3926228	100.01921	100.160141	100.038065
99.963433	100.834838	100.274843	100.214118
99.2416675	99.7725777	100.048982	99.6297895
100.159343	100.331086	100.130859	100.148406
99.2360274	99.6343774	99.5663005	99.8610894
99.1230598	99.29736	100.002542	99.4737428
100.242617	100.211703	100.610743	99.9306487
99.044928	99.0572061	99.8887721	99.5866997
99.6426684	99.508737	100.159406	99.8478181
100.54569	100.47418	100.066383	100.315008
99.2345304	99.9112628	99.5652204	99.9887459
100.329656	100.676062	100.127091	100.226504
100.451799	100.737679	99.4484753	100.29373
99.5918173	99.6281014	99.5975354	99.8251525
100.755908	100.629617	100.74145	100.294347
99.7257584	99.924732	99.841299	99.7745788
99.4115121	99.5318048	99.6806579	99.372765
100.980417	100.426341	100.625265	100.185181
99.3604948	99.4406172	100.402796	99.6341136
99.0276364	99.5773212	100.526079	99.6194657
100.409257	100.408382	100.006797	100.243464
99.4469084	99.5410938	100.009225	99.6814493
99.4781502	99.2687696	100.475645	99.4675906
99.2425071	99.0987798	100.248132	99.5209396
99.6202341	99.4472743	100.426519	99.5858527
99.6898635	99.4821083	100.545318	99.6873084
100.051305	99.7220757	100.260647	99.8646246

99.9668855	99.691267	100.335177	99.677132
100.327164	100.153398	100.471162	100.02791
99.9730342	99.8369504	100.553724	99.8650872
100.5309	100.129401	100.31463	100.008636
100.119937	99.6787267	100.349934	99.7457457
100.222803	99.8244101	100.467239	99.9673138
99.8063557	99.3916948	100.328826	99.9423354
99.9672179	99.6189671	100.512069	99.928921
99.9522617	99.524838	100.641143	99.8777306
99.8487316	99.6110714	100.370668	99.7799754
100.400449	100.421232	99.8461556	100.250557
99.8882824	99.9642105	99.8186972	99.9782612
99.6601173	99.4819535	99.9718667	99.7372653
100.073573	99.9412975	100.413817	100.058439
99.6162458	99.694673	100.248319	99.9045593
99.9575794	100.141632	100.328266	99.9946051
99.6712514	99.919623	100.071053	99.9044051
99.8440785	99.651324	99.9376838	100.031764
100.077561	100.001831	99.8693179	100.26243
99.3583345	99.7104644	100.152121	99.8636995
99.9854977	100.59277	99.4959205	100.248398
100.213829	100.639835	99.469396	100.422477
99.8734924	100.397855	99.6718786	100.266901
100.837336	100.901012	100.129519	100.527941
99.556421	100.013133	99.8932272	100.071391
100.315697	101.257402	99.8616594	100.596555
100.088529	100.52465	99.26187	100.259192
100.104649	100.353112	99.7507049	100.087426
101.103723	100.813076	99.9313328	100.359876
99.8374313	100.305583	99.4525847	100.105004
100.817228	100.608252	99.5420581	100.509901
99.3998795	99.6342941	99.5807241	99.7240052
100.480382	100.364259	99.4897564	100.140467
100.634929	100.713838	99.1780004	100.333048
99.5278381	99.9304602	99.0988005	99.9306171
100.198374	100.891568	99.7210049	100.245315
100.294759	100.650982	99.7591105	100.207076
99.685709	100.005547	99.7619124	99.7724202
100.660687	100.69882	99.7081163	100.245777
99.4146695	99.8934588	99.8418594	99.8364082
99.1286738	99.2523589	99.6281693	99.6786739
100.48088	100.415039	100.100566	100.471663
99.4204858	99.6158708	100.130827	100.039936
100.267837	100.838156	100.145957	100.407366
99.2268861	100.186219	99.1690344	100.338444
99.0274702	99.6358422	99.2069531	100.12952

100.866418	100.271678	99.7368823	100.382542
99.1474521	99.8157404	99.2719568	100.253641
100.501486	100.574966	100.120553	100.477676
100.526912	100.480682	99.2284342	100.2006
99.7289158	100.065771	98.752488	99.9853538
100.767541	100.22771	100.313136	99.9835035
99.7339012	99.4098085	99.9358158	99.6325717
100.14503	99.5858361	100.557086	99.7303269
99.9117138	99.4465003	100.472656	99.8556817
99.9630634	99.7883376	100.550922	99.7551511
100.01541	99.9041412	100.639648	99.7714951
99.6263828	99.9560052	100.307532	99.8530605
100.027541	99.8121796	100.375524	99.6871542
99.8457403	99.8821571	100.372909	99.8809685
100.210007	99.7189793	100.479754	99.762398
100.318024	100.364104	100.012214	100.153573
100.638253	100.475418	99.9038744	100.370361
100.036681	99.8375696	99.7564954	100.04302
100.304231	100.158816	100.327519	100.073395
100.055958	99.8583152	100.315191	99.9791863
100.048148	100.190089	99.8639009	100.179322
99.9964655	99.6432735	99.9023801	99.8066499
100.226459	99.8079995	100.102808	99.9016297
99.9755269	99.6099877	100.094029	99.9110352
100.547851	99.9436197	99.9388045	100.141084
99.9881566	99.4082603	99.7966557	99.8687877
100.451466	99.9913036	99.6197636	100.143088
99.7995423	99.6333652	99.7837671	99.756693
99.6835487	99.6751659	99.8698782	99.8148219
100.717188	100.161603	99.8642745	100.161436
99.7815949	99.8151211	100.115323	99.8282362
100.47872	100.66538	100.340034	100.305448
99.2835535	99.8964004	99.5803505	99.7603935
99.6051117	99.9024382	99.9247951	99.8954622
100.79672	100.303766	99.9937146	100.142827
99.4834289	99.8242882	99.7507142	99.4612158
100.14373	100.333134	100.118761	100.127883
98.8179773	99.4771227	99.5129397	99.6151331
99.3639633	99.7116063	99.8343272	99.7738266
100.551641	100.298047	99.8634425	100.163319
99.1863434	99.256396	99.5607185	99.606351
100.4674	100.273006	99.9108481	100.344969
100.800209	100.427113	99.9270854	100.39535
99.4731272	99.6233465	99.5448545	99.9460783
100.33298	100.012864	100.501365	100.203223
100.356907	100.017347	100.075088	100.212314

100.055834	99.8252156	100.346644	99.8684264
100.468895	99.8474738	100.455639	99.8664235
99.5508879	99.3713965	100.116521	99.6705988
100.505449	99.5231848	100.578446	99.8733567
99.6264885	99.4466723	100.304651	99.9077146
100.349596	99.9235225	100.478782	99.8645746
99.8396656	99.8538112	100.230929	99.7753673
100.456268	99.7456118	100.809128	99.8206643
100.039716	100.031567	100.610921	99.7235993
100.066301	99.8678771	100.646941	99.7692044
100.085741	100.139921	100.088712	99.9054035
99.7366494	99.9601557	99.6896842	99.9406858
100.306562	100.220916	99.6392924	100.166709
99.7095661	99.7695702	99.6557164	99.863188
100.400273	99.9561369	99.9871823	99.9548604
99.5068567	99.8547386	99.8124908	99.7582654
100.610127	99.8037303	100.356909	99.8063356
99.8489703	99.9031192	100.034028	99.7570328
99.9902022	99.7828633	100.255565	99.8146555
99.8298624	99.8700411	100.022457	99.9297468
99.7785205	99.5677011	100.265644	99.7701289
100.021273	99.8822521	100.072288	99.895697
99.62167	99.4987626	100.084793	99.8109578
98.8463898	99.0812676	100.22589	99.634238
100.200389	99.8451552	100.309316	100.137281
100.121963	100.215352	99.6316403	100.453589
99.9405218	100.295265	99.680539	100.344507
100.069292	100.409956	99.8871453	100.40367
100.031741	100.361575	99.7165598	100.13081
100.357405	100.492806	99.9252191	100.324323
100.072449	100.37595	99.5622116	100.160392
100.32002	100.215661	99.7501543	100.093525
99.7549264	100.035586	99.9009564	99.804949
99.9843868	99.8989458	100.435483	99.8450075
99.9845529	100.209942	99.9446292	100.008631
99.9873776	99.9068289	100.376319	99.9594826
100.177791	100.223698	100.24922	99.9295927
99.8634258	100.101279	100.217118	99.8807521
100.429018	100.400991	100.054745	100.160083
99.6977691	99.8853436	99.7034953	99.770437
100.461751	100.366831	99.6740067	100.198293
99.790816	99.7598323	99.3542989	99.8462401
100.312377	100.407947	99.8905047	100.091059
99.9584666	100.268833	99.227013	99.9725786
100.158684	100.383834	99.5612785	100.024193
100.048024	100.268833	100.181097	99.9201944

99.9051307	100.258786	100.279455	99.7685882
99.6625442	99.96402	99.9487352	99.7554921
100.214346	100.446435	99.709281	99.9665698
100.090061	100.159243	99.6344398	100.021111
99.7899852	100.301602	99.5194719	100.142827
100.547154	100.303921	99.8029724	100.059475
99.6191777	99.8570571	99.2367181	100.104464
100.054504	100.157542	100.030482	100.087978
99.5130045	99.7616871	99.9039426	99.8702752
99.9518204	100.232818	100.30857	100.003855
99.8215547	99.7119154	100.234662	99.9109501
100.194739	100.367295	99.9957676	100.173642
99.5686665	99.7508672	100.077514	99.8687345
100.229964	100.274707	99.9506016	100.098455
99.6389501	99.7967746	99.8839725	99.8691967
99.797296	99.8672588	100.177738	99.8124985
99.8265393	99.6575066	99.6667279	99.6308484
100.080757	100.030022	99.7124538	100.003701
100.842578	100.519701	100.126413	100.274713
99.302652	99.6969221	99.6741934	99.8131148
100.388975	100.652632	100.747725	100.463142
99.448204	100.101279	99.6824054	100.107699
100.163668	100.690656	100.149556	100.27271
100.247577	100.61538	99.5573591	100.422313
99.7991237	100.082576	99.3916261	99.9422266
100.487837	100.58122	100.459559	100.370853
99.352997	99.7190256	99.504541	99.9926079
99.8109207	99.8691136	99.2796444	99.9463865
100.976998	100.23931	100.180911	100.343891
99.6254916	99.6813104	99.464041	100.204918
100.570416	100.137139	99.4737461	100.361609
100.681242	100.130029	100.512563	100.409679
99.2072789	99.3387821	99.8507512	100.216474
99.7785205	99.3859261	99.7671382	99.9836718
100.786584	99.9293962	100.353549	100.344969
99.3340553	99.3638225	100.266763	99.9360637
99.3609725	99.3001395	100.481582	99.7471722
100.203213	99.9181125	100.242687	99.7638119
99.4712995	99.959692	100.335259	99.6812297
100.467898	100.223544	100.859707	99.865345
99.5934236	100.29681	100.042613	100.07103
100.760996	101.058379	99.7714308	100.378094
100.482354	100.969038	99.977104	100.176415
100.082433	100.606091	99.92913	100.006055
100.214112	100.420117	100.527864	100.047449
99.8840848	100.395423	100.367879	99.9289613

100.11726	100.372428	100.492996	99.9331161
99.8754609	100.406073	100.254136	99.8720256
100.102997	100.124565	100.417478	99.8681786
100.166681	100.133208	100.22859	99.8334016
99.8295225	99.8316366	100.145055	99.6453599
99.9464416	100.280752	99.9684738	99.870179
100.091886	100.239545	100.016395	99.9334238
99.5683202	100.036594	99.5778328	99.8543294
99.9119463	99.8746962	99.6848628	99.9034172
99.8398048	99.7646551	99.7879771	100.088689
100.206981	100.164538	100.01136	99.9837427
99.5771099	99.6171105	100.479757	99.8361715
98.8202037	98.8232097	101.068981	99.5821151
99.9928776	99.577292	100.662491	99.8851054
100.035002	99.9549505	100.437989	100.088074
99.5630133	99.7192805	100.32984	100.13116
100.024388	99.9608153	100.589211	100.05422
99.6762838	99.7030753	99.8336607	100.024213
99.9003373	99.9393626	99.8413057	100.08792
100.588253	100.464411	100.013971	100.229643
100.657078	100.749006	99.278186	100.297505
99.6731328	100.413326	99.2205688	100.10854
100.85725	101.007517	100.100303	100.515553
99.5892164	100.365328	100.0177	100.029753
100.14479	100.549604	99.969779	99.9675853
100.436507	100.811666	99.9408772	100.322279
99.7336654	100.097402	99.865546	99.7772353
100.040806	99.9878239	100.020497	99.9485041
99.4331584	99.4792891	99.6423492	99.8295546
100.31909	99.7271516	100.101422	99.8569453
99.8467701	99.3288122	100.143563	99.5835
100.20499	99.8466071	99.7909606	100.103769
100.199186	100.234297	99.5957333	100.23703
100.221741	100.000942	99.9285706	99.9946682
100.276634	100.078573	99.5287929	100.037755
100.413289	99.808795	100.000359	99.9135733
100.055234	100.021778	99.862749	99.9954376
100.57482	100.021932	100.074199	99.9685086
100.288575	99.5302198	100.037652	99.9326544
100.531037	100.340326	99.7527356	100.297966
100.333353	100.178428	99.6539099	100.347362
99.7267	99.7316273	99.7754841	100.031138
100.449443	100.050021	99.9634393	100.226412
100.009628	99.783021	99.8252698	99.9583525
100.298692	99.9555679	99.8129633	100.180248
100.057225	99.8424401	99.7665338	100.197482

100.41445	99.8813326	100.134799	100.081149
100.163861	99.7297753	99.7920793	99.8880291
100.708821	99.9382823	100.025159	100.035446
100.104987	99.7415048	99.6345177	100.028676
100.304994	99.7925898	99.8435433	100.093767
99.8529063	99.858954	99.7396831	100.003439
100.470339	100.137992	99.6292967	100.235799
99.7782771	99.8171291	99.8265751	99.9488119
100.195869	100.185527	99.847459	100.105308
99.6127661	99.7269973	99.5507956	99.7850832
100.261045	100.150493	99.8077423	100.163013
99.8358245	99.85988	99.7079843	100.028214
99.9573872	100.016993	99.7788404	99.9572753
99.6048056	100.015604	99.7719413	99.9469653
100.004984	100.31733	100.106457	100.11608
99.6936973	99.9205337	100.010987	99.7456898
99.8393072	100.000171	100.356691	99.7229156
99.4271881	99.8251545	100.30243	99.7870836
99.9199068	100.100643	100.116899	100.012211
99.4769409	99.8731528	99.6596903	99.8787963
100.168505	99.8517002	100.09471	99.9926678
99.4751166	99.8422857	99.7503115	99.8898757
100.387252	100.085981	100.084268	100.113156
99.6847418	99.9384366	99.9220444	99.9952837
100.247778	100.316712	100.097879	100.45554
99.5320007	99.8225308	99.8482048	100.139316
100.058551	100.117003	99.8017754	100.071454
100.214278	100.016067	100.276139	100.042217
99.7417917	99.9268615	100.355945	100.044525
99.5392977	99.6433475	100.067486	99.9340394
100.069331	99.8980008	100.0177	99.9108034
99.7560542	99.6648001	100.324246	99.7459976
100.146614	100.119935	100.385593	99.9757409
99.9792785	100.000634	100.251152	99.8847977
100.385759	100.107125	100.134426	99.8991085
99.9286965	99.7790083	100.215165	99.7638477
99.9961944	99.9978557	100.213114	100.028984
99.7633513	99.7935158	100.247237	99.7912384
100.03069	100.029186	100.020311	99.969278
99.9492609	99.8253089	100.555834	99.8043182
100.163696	100.125645	100.245372	99.9354243
99.46102	99.7672788	100.017327	99.8366331
99.7809306	100.099254	99.9507598	99.8446349
100.21859	100.205128	99.9022793	100.132237
99.9306866	100.102186	100.175448	100.167476
100.437004	100.369804	100.333196	99.9674314

99.6228825	100.369804	99.8079287	99.9526589
100.466359	100.405455	100.47379	100.253341
99.5457656	100.102186	100.176008	99.930654
100.270664	100.63063	100.429039	100.231336
99.3084447	100.129658	99.8338472	100.041756
102.718961	99.9457216	100.180882	100.132296
103.201643	100.948099	99.8629544	100.46929
102.486416	100.855095	99.186032	100.521952
100.806683	99.4937541	98.7147473	100.167403
101.286362	98.9602041	99.6771682	100.067289
101.367452	99.1554584	100.675504	99.9039035
100.24334	98.8780776	100.05556	99.7889357
97.9389102	96.3691416	100.045407	99.5381671
100.118057	98.6877183	99.6611051	100.090051
99.034276	96.7824933	100.453045	99.9044822
97.4532252	96.1042702	100.967064	99.8375463
101.493164	102.26321	100.651712	100.36879
105.027681	104.944558	99.7170228	100.460996
103.173325	103.823069	98.8914411	101.404657
102.333674	102.469886	98.7274765	101.578652
102.432784	103.20141	98.6435242	101.584632
101.994724	103.200322	98.9767573	101.929535
101.807658	102.008129	100.447438	101.254582
102.102416	103.784454	100.351969	100.708485
100.617043	100.786566	100.304538	99.7574932
99.1608458	98.8215137	100.154363	98.7231691
100.930679	100.340581	99.9322075	99.0914132
102.230273	101.893369	99.7720313	99.521192
101.524914	101.184145	98.985395	100.109534
99.8280192	100.08278	98.4574349	99.8448764
101.628744	100.54345	98.865225	99.794144
102.930483	101.998883	100.558213	99.5015163
102.346974	102.22949	100.327268	99.9243508
100.980449	101.309782	99.583972	99.7171773
100.426974	100.510273	99.2322512	99.1321148
101.200981	99.8597879	100.321964	100.168947
101.468279	101.15967	100.480625	100.862611
100.611036	99.6150402	99.1848197	100.987803
100.02195	98.7230707	99.7368744	100.785645
99.4534582	98.4021793	101.503662	100.380364
99.5358359	99.2120223	99.8608329	100.346414
99.5319745	99.6215668	99.4190982	99.8585722
100.09446	99.7966974	99.4842597	99.6685668
98.9845061	98.1035871	100.10875	99.7949156
98.9162871	97.954019	101.043742	100.5451
100.203438	99.4235931	99.9370567	100.738191

99.8202963	99.3822579	99.3853051	100.517708
98.6777351	98.3891261	99.7459667	100.357216
100.638495	99.4143471	101.192553	99.593722
100.147233	99.9239662	100.595643	99.8419829
99.7280505	99.6302689	99.7394505	100.167211
99.5701599	98.740475	99.4713789	100.085807
99.8507589	98.7943195	99.6980805	100.207526
99.8018472	99.0434183	100.823102	99.6286367
100.44671	99.9179835	100.715965	99.7632802
100.60031	99.5541252	99.7929436	99.4610075
99.9906294	99.4904908	99.2696812	99.1786035
100.37892	99.7542744	99.7003536	99.2339655
100.469021	100.080605	101.076929	99.4415247
100.174263	100.343301	100.464411	99.7491985
100.190567	99.4453484	99.8096128	99.9656312
99.1724302	99.302307	99.5069905	100.120143
99.4251409	98.5854681	101.524119	99.5923717
100.18928	99.8282426	100.975853	99.5485836
99.2372168	99.1451246	99.7605144	99.5659446
99.9811902	100.307948	99.2940789	99.9096905
100.626482	100.051235	99.5601805	99.8271297
100.482321	99.9886884	101.171641	99.708304
99.8288773	99.5416159	100.649439	100.281021
99.9867679	100.052323	99.4715305	100.131717
99.6744192	100.140976	99.298322	100.008648
99.8048505	99.6003553	101.140121	99.3539486
99.93614	100.627752	100.403795	99.0003649
99.264676	99.451875	99.8749259	99.1033729
99.5821734	99.597092	99.5942767	99.3026375
99.9271299	99.9919517	99.2478597	99.5238926
99.1149374	99.2598841	100.472897	99.6650946
99.3727968	99.5095267	101.452442	100.364932
98.9403139	99.658007	100.095718	100.52253
98.7373731	99.4486117	99.1636043	100.432061
99.0647386	98.8884114	100.101021	100.018871
99.3195945	100.237787	100.613676	100.015785
99.3848102	99.8418397	99.4744097	99.9023603
100.290107	100.340037	99.0038827	99.8624302
99.6400952	99.2903416	99.6924736	99.3788326
99.6722739	100.163275	100.805221	99.5208062
99.7906919	100.575539	100.276958	99.8836491
99.1917375	99.8304182	99.890989	100.021572
99.7010203	100.32916	99.4641051	100.213892
98.6717284	99.0706125	99.5844267	99.9613874
99.6225041	100.33623	100.980854	99.8969591
99.4834918	101.250499	100.487748	100.120529

99.2273487	100.560854	99.7383898	100.280056
99.5015119	100.659297	99.2286143	100.377856
98.9918	99.9457216	100.013129	100.280828
98.9107094	100.015339	101.058442	100.043755
98.6017931	100.161643	100.481535	99.9642809
98.762258	99.5508619	99.7593021	99.8614657
99.2359297	100.240507	99.2730151	99.7441832
98.2881573	99.5780561	99.6000351	100.035653
98.2971673	99.1652483	101.016314	99.9909009
98.4782266	100.613067	100.20528	100.126702
98.4400411	99.7183781	99.8779567	99.9515495
98.6610021	99.8423836	99.341662	99.8871213
99.2209129	99.7123954	101.046773	99.7995452
99.4467145	99.670191	100.846694	99.9760925
99.434282	99.4563795	99.5207198	100.061104
100.019467	99.7949144	99.0540131	99.6533975
98.7920791	98.430516	99.9470937	99.2655465
99.8634177	99.5341291	101.075196	99.4453999
98.638602	99.4660982	100.290376	99.4507974
98.7342036	99.0892283	99.971354	100.133777
99.0621644	99.550327	99.151509	100.328088
98.7016219	98.8575992	100.129197	99.9126716
98.553718	98.7625718	101.265184	100.20356
98.7865059	99.6340158	99.9774191	100.125681
98.8045116	99.5206309	99.7918281	100.074212
98.3629435	98.6837423	99.5898615	100.228427
98.5781543	99.5470874	100.826528	99.7765768
98.6158805	99.723104	100.394999	99.7490108
98.34751	98.6103121	99.4811452	99.8463591
98.6630383	99.9293565	99.3328544	99.8702624
98.1987487	98.7550128	99.8101749	100.138789
98.205608	98.8635384	101.097485	100.053971
98.3479387	99.5816428	100.620619	100.353534
99.2422213	99.7036665	99.5837964	100.323655
99.7245166	99.9574328	99.2350553	100.036236
99.6580671	99.2350089	100.834261	99.2719079
100.301127	100.361299	100.040951	99.8995633
99.9830269	99.2458075	99.339526	100.448569
99.6735005	98.8905348	100.109789	100.189102
100.29684	99.9720108	100.655645	100.281824
99.6237705	98.0606653	99.4323215	99.761348
99.0132919	98.0223304	99.808507	99.1471865
100.280978	99.3915881	100.672627	99.4108943
99.5830433	98.5752168	99.5402796	99.4128219
100.182804	99.4617787	99.1577257	99.4363397
99.1586235	97.5353152	100.755718	99.0922474

100.794998	100.047601	100.070215	99.7343604
100.02847	99.2447276	99.6218547	100.073248
98.9138318	98.009912	99.6756821	100.139946
100.008749	98.6977805	100.040496	100.594302
99.7729605	100.215518	99.7866728	100.940515
98.648891	98.5827758	99.9078224	101.105525
99.7858217	98.5223038	101.090207	100.799023
99.4162764	98.5919545	100.03625	100.619748
101.378468	99.9185579	99.188051	100.804613
102.637151	101.617712	98.7613738	100.539941
100.690822	99.1324226	100.34572	100.953238
98.9768518	97.6476203	99.6556674	101.12538
100.662099	99.9012802	99.6232193	101.064851
100.483328	99.9714709	99.2696261	100.666205
100.741838	100.347801	101.119319	100.578302
100.656954	100.27977	99.8911435	100.687988
100.816862	100.43041	99.3031356	100.617434
100.066625	98.8683977	100.818795	99.77889
100.110782	99.6647917	100.291589	99.5167244
100.157082	98.9180711	99.4298954	99.3008232
99.8415536	99.2350089	99.8212437	98.6548548
100.657383	100.713872	100.757993	98.6307587
99.5285976	99.3051996	99.3546886	98.7876726
100.337568	100.251694	100.131775	98.789793
100.501334	101.084263	100.460198	98.9503695
99.9633064	99.4768967	99.4957014	99.050031
101.565384	101.981083	100.462776	99.1421745
99.7759615	100.535696	99.5716663	99.1294517
100.841298	101.687362	99.2835758	99.2578358
100.516338	101.839622	100.671869	99.6281447
100.88374	101.126917	99.4311084	100.009249
101.044934	101.222484	99.7546796	100.041827
101.134105	101.720838	100.417439	99.8239979
99.593761	100.059479	99.0371826	99.8367206
101.588963	102.700268	100.179386	100.347365
101.9778	103.687796	100.063998	100.792083
100.532629	100.476844	99.5333048	101.009141
100.869164	100.640442	100.602879	100.667362
100.998205	100.57835	99.2535537	100.151705
100.799285	100.090255	99.579096	99.8991777
101.139249	100.552973	100.899309	99.4972547
100.343141	100.169625	99.4030575	99.6102172
99.917006	99.7333626	99.8035034	99.6086751
100.498333	100.531916	100.90219	99.92231
101.719719	102.037236	99.2735684	100.082115
100.012179	100.309466	100.888695	99.7977813

99.8552722	100.439589	99.9429998	99.7042884
100.167371	100.73061	98.9592465	99.9317557
100.065339	99.9207177	100.019874	99.7808177
100.828437	101.693302	100.242917	100.006164
100.05162	100.720351	99.140137	100.080188
99.7116554	99.8813029	100.422443	100.067079
99.3536851	100.05678	100.336471	100.447991
99.4304237	99.8413482	99.9393608	100.454159
100.201239	100.435269	101.237588	100.041056
100.978913	101.005973	99.5345178	100.183512
100.649666	101.110179	100.346175	100.125874
100.653525	100.858033	100.695826	100.688373
99.5740405	100.88071	99.5117738	100.88519
100.707542	100.640442	101.165868	100.100043
100.381296	101.692762	99.9138875	100.293968
100.91032	100.567012	99.832464	100.134934
99.9311534	100.589149	100.812123	99.9192257
99.6241992	100.298127	100.119342	100.012333
99.9954594	99.9876687	99.59532	99.4812549
98.9146893	100.770025	100.664136	99.3960511
100.530691	99.6625357	99.5965284	99.496716
99.3426956	99.9907471	99.499365	99.7261185
100.009997	99.5255102	101.061861	99.5566604
100.774171	100.344956	99.4904217	99.153765
101.061764	100.395325	101.060648	99.1898853
101.221346	100.512311	99.9668405	99.7391833
100.90521	100.386117	99.7565978	99.5779867
99.6112595	101.205563	100.535724	99.8146902
101.156475	101.078286	99.2888192	99.8385142
99.9490186	101.392957	100.095078	100.165134
101.065224	100.956967	100.69261	100.365141
100.856773	101.271097	99.4923923	100.251977
100.052379	100.78907	100.962121	100.203944
101.162097	101.615015	99.7038476	99.749174
101.426336	101.45795	100.742935	99.7945166
101.861834	101.666467	99.4072038	99.4532947
101.572079	101.744458	100.140552	98.8915083
100.632754	102.909446	99.8663424	99.4519498
100.976135	100.979173	99.4096291	100.107688
100.275966	100.365536	101.106729	100.31903
100.948024	100.497688	100.557854	100.296743
99.6056374	99.8125598	99.2434965	100.42643
100.496958	99.6527869	99.8986291	99.7991277
99.6774274	99.6289563	101.018509	99.6135307
100.723572	99.4318128	100.223012	99.4740447
100.6933	99.3077857	99.5099757	99.5093965

99.8646869	99.5049292	99.3194385	99.6235214
100.384083	98.9151236	101.208136	99.3299476
98.8522746	98.8111358	100.620457	99.8402434
99.7924645	98.6784431	99.471171	99.6189103
98.5962523	98.9427453	99.3594558	100.025072
100.396193	99.6284147	100.703372	99.7507111
98.6905308	99.7215704	100.407031	100.203944
99.9252328	99.0061129	99.6503396	100.184155
98.9703389	99.3159098	99.3088278	100.235646
99.6086647	99.3034529	100.144948	100.055044
99.4010791	99.6311227	100.671388	100.274264
100.158334	100.113149	99.4814785	99.9772317
98.8972514	99.6229987	99.2146961	100.061384
99.9442614	99.1366392	100.480245	99.6974912
98.8310835	100.231219	100.092198	99.7572435
99.7764631	99.6078338	99.1830157	100.14131
99.9987527	99.1826755	100.102051	99.9347711
99.0827811	99.7475674	100.261362	99.9585951
100.18385	99.0754381	99.6573123	100.070222
99.4300545	99.9918303	100.517989	99.8104633
99.6506143	99.9457941	99.9221241	99.8519633
100.877532	100.053031	99.180742	99.5954705
99.720242	100.62009	99.7806991	99.56204
100.528528	100.567013	100.202246	99.9801136
101.33941	100.551848	99.3126174	100.14035
98.9344439	99.7389017	100.863745	100.391655
100.481389	100.403449	100.435225	100.377245
98.80946	100.246384	99.3402051	100.560921
100.555342	99.813643	100.894061	100.306349
99.3682113	100.84323	99.9635057	100.489257
100.465388	100.041658	99.3402051	100.420474
99.6376402	99.6939487	100.222861	99.8481207
100.625402	100.860562	100.393541	99.6292853
98.962122	99.6961151	99.340963	100.181849
100.547557	99.9057155	99.386892	100.026417
99.0897006	100.641212	100.792956	100.138428
100.074435	100.591926	99.5801576	100.459669
100.020376	100.416447	99.2654757	100.421435
100.831258	99.9647502	100.759912	99.9030697
99.294259	100.398574	99.3339903	100.095391
100.307104	99.8369319	99.5775808	100.233532
99.3275591	99.4675586	100.461449	100.064843
99.0006118	99.4161063	99.5627258	100.103076
99.972372	99.7389017	99.1014652	100.121137
99.8569025	99.8293494	99.9689626	100.112491
99.907069	100.056281	100.859955	100.382817

100.597724	99.8131014	99.968811	100.257741
98.7510766	99.6549533	99.5839471	100.119023
99.1766271	99.3706117	100.976369	100.444106
100.170876	100.171643	99.994125	100.463703
99.2613912	99.6078338	99.1419373	100.632009
100.68292	99.8650952	99.4840553	99.9705071
99.0520757	100.343872	100.801293	99.9368845
99.6705079	100.209555	99.8883216	100.834321
98.9690415	100.284296	98.985051	100.830671
100.514689	100.344414	99.6601923	100.442761
98.5062985	99.7432346	100.711254	100.223157
99.7794903	100.562138	100.15086	100.456402
100.093896	99.7416098	99.6318467	100.164942
100.316186	99.9837063	99.8437568	99.8606091
100.746061	99.6533285	101.420804	99.9818428
101.097659	100.285379	99.6072906	100.156488
99.3344787	99.9793735	100.012618	99.9514863
101.395198	100.440819	100.800232	99.6873084
100.082652	100.527476	99.2297026	99.2444501
100.118547	100.725161	99.4690488	99.7789541
101.130959	99.527135	100.809933	99.551665
99.3262617	99.7632739	99.8049521	99.6661741
100.594696	99.0873533	99.7341638	99.8565744
98.9448231	98.4731755	100.504346	99.7983592
100.270776	99.0526907	100.531025	99.4780794
99.2025753	99.0624396	99.2844233	99.8759795
100.195094	98.3150274	99.458135	99.5226535
99.2151169	98.962243	101.001532	99.7599333
100.184282	98.7916381	100.158742	100.001824
97.1808538	99.06769	99.0983612	100.263782
99.0362539	99.1443249	99.7469361	100.093966
99.210518	99.122041	100.859385	99.8415565
97.39185	99.5834808	99.959084	100.113649
98.9649252	99.4535819	99.3485713	100.246801
97.8475848	99.9905695	99.8109293	100.286939
99.2844094	99.8606707	101.20513	100.403109
99.1665249	100.30961	99.4996072	100.340586
99.6799206	100.556907	99.6711149	100.087019
99.9656624	101.040087	100.321206	100.257221
99.3471957	99.4840185	98.4505446	99.8319078
98.6843088	99.1709569	99.308841	99.8238029
99.5180429	99.6655507	100.308165	100.431283
99.4407346	99.9367621	99.1129189	100.697008
99.2839823	99.2215033	100.896234	100.822247
99.667107	99.4552125	99.285943	100.547067
98.3464585	98.8144145	99.4055889	100.093001

98.975176	99.1861751	99.5523788	100.172699
97.9953676	98.3725411	98.7656576	100.181383
98.8816372	99.3122694	99.7017467	100.137964
98.9709049	98.8660479	99.2917054	100.756444
97.6652055	98.536681	99.7106936	100.901367
99.0969046	98.8877883	99.6380568	101.099743
99.3583008	98.9997513	99.0357329	100.653782
98.6872986	99.793819	100.41022	100.385935
99.5833919	99.4073836	99.1250503	100.364322
98.1730486	99.5476091	98.9059269	100.476053
98.4007025	98.7888695	100.425991	100.643554
98.4575092	98.6317952	98.8073593	100.753742
99.9695065	100.583539	99.9589323	100.640852
100.10832	100.39331	100.357752	100.979907
98.6215224	99.5823937	98.9674938	101.285963
98.5758208	98.9203989	100.944608	101.461375
100.176659	100.472663	100.225671	100.295623
99.5808292	99.8019717	99.2616802	100.624064
99.2280298	100.045464	99.7736252	100.829773
99.7042663	99.851431	100.306042	100.677903
99.3343822	99.8856722	99.5235667	100.790407
99.6320834	99.7851229	100.512731	99.9886023
98.0684047	99.7313154	99.4019495	100.33827
100.084828	100.477011	100.821171	99.3469656
98.5732581	100.022093	99.599388	99.5702359
100.417126	100.771593	99.7561863	99.4222252
99.3433517	101.124331	100.513792	100.22596
100.450014	101.361301	99.8852341	99.5850948
98.1375978	100.979757	100.791753	99.8023829
100.054503	101.206401	99.6410897	99.7591568
99.5376903	101.746106	100.697128	99.779998
100.913864	101.472177	99.6150072	99.7037735
101.779205	101.889592	100.556404	99.6806167
101.089837	101.771107	100.322268	99.9444113
100.508956	102.580393	99.4756477	100.277676
101.605368	102.106453	100.521526	99.5355006
100.466672	101.655883	99.4430446	100.483
100.171106	101.132483	100.026413	100.085668
100.497424	101.425979	100.092226	99.8548717
100.288564	100.867251	100.047795	100.278448
100.051513	100.214496	100.01231	100.016004
99.9793302	100.182972	99.7439033	100.451546
100.093371	99.2220468	100.795089	99.4542588
99.9353371	99.5813067	99.8323108	99.8458019
99.8704152	99.5970685	101.382855	100.350813
100.097642	99.6628332	99.6971974	100.73483

100.366726	99.2785718	99.7375343	100.151858
99.7879814	99.0769297	100.813741	100.114614
100.044252	99.1840011	99.6441225	100.018706
99.6051749	98.8763746	101.204069	100.030863
100.177086	99.8275166	99.7467845	100.348884
100.722515	100.041116	99.6918899	99.8400127
100.642217	99.931327	100.461172	99.6620912
100.620861	100.182972	99.9797073	99.488994
101.906913	100.68137	101.30203	98.842726
101.811666	100.710176	99.7175175	99.1161694
99.9524218	100.231888	100.494988	99.4826259
101.926561	100.693328	100.380953	99.7689985
101.611775	101.00802	99.5363047	99.2796179
100.907457	100.322654	101.51903	99.6175144
100.719526	100.596583	100.294669	99.4936254
100.438055	99.8150159	100.220212	100.24024
101.155186	100.686262	100.920042	99.3544916
101.11461	100.069378	99.4130194	99.6684593
100.576868	99.6693553	100.366092	98.8002719
100.453858	99.3997745	100.491349	99.2199891
101.072752	101.149332	99.8345855	99.3137742
101.616046	100.348742	101.080328	99.7915764
102.028215	100.234605	100.013675	99.3606667
101.381985	100.478641	101.482939	100.032793
100.59737	100.37157	99.6692952	99.4226112
100.96939	100.135687	98.8008387	98.7609053
100.017344	99.8970859	100.066144	99.0557687
101.149633	100.477011	100.893505	99.1659565
100.716536	100.294935	99.6203147	100.265326
100.649051	99.8133854	101.11005	100.139314
100.009228	99.3514021	99.4618483	100.373005
100.794271	99.1274761	99.5282677	99.1466592
99.8230051	98.2323156	99.7853017	99.0723644
100.192462	98.3078634	99.6520079	98.9332306
100.229621	98.2703613	100.269799	99.5702359
100.848515	98.7921306	99.2506103	99.3431061
99.7862729	98.4845041	100.205655	99.7487363
99.3213502	99.0970051	99.7399575	100.134013
99.4563975	99.2341413	99.4404284	99.9798074
99.4892553	99.1515859	99.6316073	100.059619
99.6640778	99.5342597	99.7009793	100.215099
99.2999691	99.4983793	99.9515537	100.197894
99.7664244	99.8585518	99.6118862	100.088771
99.6164418	99.4267705	99.9322965	100.232304
99.3487056	99.3594187	99.6378717	100.106772
99.7307367	99.6484385	99.7336932	100.357835

99.1957359	99.2604435	99.8051533	100.269581
99.8738019	99.6086052	99.5937892	99.881517
99.6546449	99.484088	99.4355561	99.9340872
99.6996083	99.5129747	99.7977288	100.003384
99.3792052	99.139119	99.9362408	99.9070056
99.7261775	99.5978106	100.16663	100.084948
99.2774874	99.3249066	99.8325308	100.101356
99.5417649	99.5298507	99.8589804	99.9085986
99.3829783	99.5664913	99.4968076	99.7969267
99.5279301	99.6266974	99.7965688	100.015014
99.4911419	99.4228176	99.9856596	99.7806778
99.6544877	99.5362362	99.9448253	99.7908732
99.4199237	99.1581235	99.9464494	100.131465
99.4724334	99.6190956	100.240642	99.9396629
99.220733	99.4652355	99.9063111	99.8829507
99.6274468	99.710317	99.8573563	100.02951
99.2293798	99.3916503	99.6346235	99.9885691
99.4521527	99.6031319	99.6181506	100.122066
99.4829667	99.4319397	100.424165	100.147554
99.4760493	99.42449	100.72323	99.8861368
99.2985541	99.2355096	100.436693	100.245207
99.450895	99.4184086	100.305142	99.9949413
99.4832812	99.3124397	99.8963345	99.9978087
99.8857502	99.8088362	99.3666482	99.9611689
99.7005516	99.7127496	99.2622422	99.9036602
99.7060541	99.6399245	99.539034	99.7942186
99.7371825	99.6932891	99.4954155	99.8404167
99.9997307	99.8489736	99.7928566	99.8716402
99.6411245	99.6397725	99.7077077	99.8408946
100.006963	99.8320976	99.8313708	99.7696858
99.8384287	99.7717395	99.9065431	99.8875706
99.7725558	99.6758049	100.238554	99.9230953
100.079596	99.8964087	100.05596	100.04321
100.074879	99.8235836	100.06408	100.137199
100.028187	99.9511416	100.015125	99.9237325
100.259606	99.9281842	100.251779	100.163007
100.13918	99.7328183	100.100274	99.8999962
100.177383	99.9260557	100.428573	100.020111
100.187288	99.9395868	99.9148956	99.9753469
100.21653	100.176915	99.8387952	100.023935
100.222346	100.035673	99.9341527	99.982197
100.065761	100.171289	100.067792	100.186106
99.7488164	99.6771733	100.093778	99.7230098
100.103807	99.9052268	100.105842	99.9105103
99.6941058	99.8203909	100.123475	99.9202278
100.054441	99.8916956	100.344352	100.009279

99.8148465	99.7241523	100.111875	100.245048
100.08557	99.8664577	100.239946	99.657536
100.369971	100.360118	100.370338	99.8213002
100.042965	100.059239	99.7102598	100.232144
100.201594	100.370152	99.6014456	100.303353
99.9778779	99.9893025	99.9536418	100.085266
100.116069	100.165816	100.446206	100.187062
100.218102	99.9964482	99.8854299	100.085107
100.470274	100.53131	99.9543378	99.8232119
100.4654	100.438568	99.817682	100.256677
100.407073	100.24305	100.077305	100.039706
100.361953	100.327582	99.8898381	99.95177
100.233194	100.192118	99.9100233	99.784979
100.299067	100.308882	100.124404	100.059619
100.319348	100.202152	99.7336932	100.023297
100.408017	100.401471	100.049927	99.8335666
100.455653	100.207018	100.171966	100.123499
100.47106	100.449971	100.26802	99.7269924
100.293565	100.428077	100.150853	100.185309
100.214014	100.172049	99.7457579	100.00434
99.8176764	99.9862618	99.9197678	100.227047
100.226748	100.273457	99.957586	100.060096
100.106008	100.177827	100.396555	100.123021
100.346703	100.154261	100.053872	99.924529
100.425153	100.786578	100.316975	100.171609
100.057743	100.001617	99.9719708	100.113782
100.375788	100.561109	100.180087	99.831177
100.017182	100.307209	100.30955	100.02266
100.220932	100.468671	100.183567	99.9756655
100.292779	100.354188	100.144125	99.9261221
100.181785	100.243962	99.9780032	100.111552
100.258663	100.520819	99.9374008	100.136562
100.46147	100.601094	99.9490015	99.9814004
99.9969009	100.338072	100.159206	100.215577
100.467758	100.61873	100.443886	99.9301046
100.275013	100.608696	100.248299	100.131942
100.536933	100.686842	100.030902	99.8010686
100.868341	101.196314	100.343656	99.9616468
100.405501	100.489044	100.262915	100.191044
100.468544	100.605351	100.094938	100.029351
100.357236	100.678936	100.119763	99.8810391
100.470116	100.806798	99.8738292	100.010872
100.416349	100.673159	100.03299	99.7948558
100.909059	101.113606	100.202592	99.6623151
100.759077	100.999123	100.252939	100.108206
100.552252	100.683726	100.030719	99.8595195

100.410935	100.711844	100.177746	100.369731
100.235979	100.586	100.276769	99.8977376
99.965764	100.28963	99.7869873	100.030068
100.450391	100.794979	99.806931	99.9268788
100.046404	100.493138	99.9838739	100.198546
100.25217	100.405899	100.082201	99.8561754
100.485759	100.490554	100.110726	100.129116
99.89094	100.13263	99.9567411	100.048062
100.154553	100.377325	99.9405078	100.048062
100.239123	100.128831	100.216242	99.8537868
100.29807	100.383861	100.109566	100.012073
100.170901	100.399515	99.9996434	99.7198643
100.545335	100.584784	100.254042	99.8219384
100.454163	100.76975	99.7452445	100.217336
100.392072	100.502713	100.100522	99.7109467
100.222775	100.699229	100.115364	100.013347
100.505723	100.794979	100.097739	99.8996485
100.209413	100.632356	100.272363	100.207622
100.585262	100.667008	100.013094	99.6526642
100.524114	100.796499	100.63367	100.066534
100.481986	100.740417	100.034197	100.094083
99.9008432	100.343433	99.9439864	100.103319
99.9906005	100.188409	99.7141693	99.9628675
100.020782	100.022745	99.7916253	99.9983785
100.063695	99.9602797	99.6357857	100.10093
100.094034	99.5974925	99.8850827	99.9963083
99.8349792	99.6797161	99.5087022	100.258739
99.8719196	99.8566262	99.8556309	100.033412
99.863274	99.987333	99.7109227	100.233738
99.9506734	99.9213717	99.8904165	99.8910494
99.5389844	99.5254517	99.751274	100.347278
99.6754281	99.5404982	100.0175	99.7792615
99.7233721	99.7236397	100.038371	100.267497
99.64116	99.5363946	100.185862	99.8623858
99.8365512	99.6063076	100.559228	100.020035
99.9129471	99.7151285	100.233867	99.8058549
99.7359475	99.8818557	100.465307	100.180711
99.7175559	99.7231837	99.7740006	99.863819
99.7782325	100.015754	100.193515	100.22275
99.8683042	99.8008479	99.9878163	100.0401
100.055993	100.13719	100.59355	100.13883
99.7887644	99.9599758	100.112117	99.9880277
99.8398522	99.809207	100.28906	100.008729
99.877893	99.9066292	99.8329043	100.180074
99.9395127	99.9148363	100.103305	100.030864
99.7293454	99.8064713	100.041386	100.226413

99.9791254	100.031105	100.067128	99.917802
99.983684	100.160443	100.255434	100.076407
100.053478	100.086123	100.120466	99.8814949
99.7936374	99.9488809	100.373009	100.127842
100.16367	100.119104	99.8338319	100.06526
99.8805653	99.9064772	100.267957	99.9568163
99.9761387	100.084603	100.257057	99.9679633
99.6320428	99.6371604	100.160585	100.159054
100.107867	99.6940027	99.9882801	100.154276
99.9674931	99.8107269	100.097507	100.173067
100.181276	99.8950783	99.8164391	100.169404
100.013079	100.159532	99.7932487	100.058572
99.7985104	99.780026	99.8271067	100.161442
100.194166	100.022593	99.8315129	100.131982
100.11384	100.009979	99.952335	100.263198
99.8914116	100.175186	100.267725	100.062871
100.072341	100.036424	100.177282	100.328806
100.078943	100.131718	100.076636	100.087872
99.9762959	100.044327	100.101681	100.167334
99.6993215	99.8858073	100.202791	100.05698
100.009464	99.9511606	99.8662985	100.066534
100.129559	100.024873	99.8973737	99.9106362
99.826648	99.8203019	99.9551178	99.8590418
99.9895002	99.9949322	100.079882	99.9302229
99.8921976	99.8110308	99.9463054	100.005067
99.8239757	99.7766823	100.0175	99.9142987
99.7914367	99.7206	100.033501	100.253166
99.803069	99.4616182	100.000107	99.9424845
99.7928515	99.5497693	100.294626	100.056024
100.128931	99.8994859	99.7280836	99.7458207
99.9434426	99.9484249	100.317352	99.9211461
100.184734	100.010891	99.7535931	100.031023
99.877107	99.9934124	100.198153	99.952676
100.26616	100.251482	99.5026727	99.8982153
100.122643	100.047063	99.9748297	99.8157279
100.136319	100.206495	99.4913094	99.8427991
99.9736237	99.9437134	99.7559121	99.8284673
100.112582	99.7886891	99.9405078	99.8845205
100.074856	99.8776001	99.9476969	99.8448692
100.20454	99.7655874	99.5144998	99.7125392
100.243682	100.143269	99.6740499	99.7587193
99.9667072	99.9041974	99.7431574	99.8467801
100.132074	100.043111	100.011471	99.5575967
99.8329357	99.9104288	100.267493	99.8584048
99.8661035	100.066365	99.8686175	99.6818055
99.6163235	99.7262234	100.257289	99.9705111

99.9010004	99.6836677	99.7784068	100.038348
99.8532137	99.8447714	100.141569	100.066375
99.877893	99.780178	99.7742325	99.9673263
99.6862745	99.6066115	100.089854	100.061757
99.9038299	99.5854857	99.3876482	99.8773546
99.8164304	99.5754547	99.5256312	100.038667
100.026704	99.650634	99.5053266	99.7919815
100.026861	99.6964563	99.7871568	99.965748
99.8129668	99.5366851	99.6723371	99.848364
99.9646716	99.9208643	99.7284712	99.84486
99.8198768	99.4652205	99.9949921	100.022131
99.902011	99.6623172	100.160843	100.05733
99.8409207	99.5812936	99.9926725	100.210869
99.6344075	99.3961836	100.062724	100.032324
99.7240798	99.4109014	100.158987	100.089662
99.8572533	99.55641	100.151101	100.301973
100.07366	99.6753659	99.6233937	100.30038
99.7259643	99.4435232	99.9344508	100.328412
99.8863065	99.3641687	99.6215381	100.109571
99.8663619	99.4165154	100.134863	100.205772
99.8239599	99.4077151	100.056461	100.031368
100.541181	99.9431685	100.529426	100.184429
100.198354	100.105519	100.617338	100.498037
100.387749	100.340093	99.7303269	100.041562
99.926824	100.151948	99.5220276	100.340198
100.189245	100.231151	99.2733675	100.134258
100.152026	100.430979	99.7484197	100.108456
99.9536785	99.930575	99.2896047	100.150504
100.273578	100.151948	99.5841927	99.8490011
99.8635351	99.5797763	100.002647	100.32889
99.8267867	99.4281985	99.6551721	99.6175779
99.9346762	99.7516859	100.200972	99.9627218
99.930593	99.6712692	99.5802494	100.014008
100.232118	100.052262	99.9583426	99.8775109
100.135065	99.9078155	100.236461	100.095555
99.9005976	99.5897905	99.9543993	99.8032899
100.005032	99.8959806	100.125817	99.9933022
100.253162	99.9219264	99.5129812	100.105112
100.11779	99.791439	99.7815898	100.043792
99.7185832	99.5680932	99.6222339	99.8145982
99.9048378	99.8612346	99.8669507	100.189048
99.9393875	99.5618722	99.9467446	99.9018796
100.154538	99.9926323	100.052518	99.8990127
99.8492441	99.778542	99.7799661	100.169139
99.9100202	99.8689728	100.117698	99.9958505
99.7941215	99.7709555	99.853961	100.067842

99.958861	99.6823455	99.740765	99.913188
99.7715071	99.6366749	100.044863	99.8044048
99.9136322	99.6404681	99.8806363	100.024042
100.087637	100.103395	99.5489349	99.9343713
99.9651428	99.8481858	99.6776722	100.00031
100.043665	100.009626	100.111204	99.997284
100.057485	99.9314853	100.324374	99.8540978
99.8528561	99.8446961	100.377261	99.8857931
100.024191	99.7850664	100.246204	99.8824484
100.218298	100.307168	100.071075	99.7459517
100.220654	100.124334	99.8646311	99.9612884
100.001263	100.044524	99.8804044	99.8808556
99.838251	100.002495	99.9736519	100.123587
99.7652254	99.8847527	100.098214	99.9389902
99.8227036	99.9575828	99.6280329	100.270277
99.8726437	99.9454445	99.8739095	99.7787619
99.6158762	99.8662416	99.8347084	100.071027
100.168201	99.9826182	100.019348	99.7661793
99.6870173	100.067587	100.270328	100.000469
99.9973369	99.8017566	100.033729	99.9904353
99.9254106	100.057269	99.9024405	100.008433
100.391204	100.238738	100.02074	99.7700019
100.386964	100.306106	100.101461	99.9123916
100.553588	100.484084	100.020508	99.8942346
100.287869	100.322796	100.060405	99.6307975
100.231961	100.227054	100.227879	100.094122
100.038954	100.136927	100.048575	100.134577
100.027332	100.082001	100.023987	100.262314
100.022621	100.247993	99.6486773	100.093644
99.8390362	99.9961221	99.6345278	100.165635
100.150769	100.156348	99.5941669	99.8819706
99.9946671	100.166059	100.145302	100.212939
99.7812439	99.7671623	100.052286	99.9582622
99.6028415	99.706167	100.322518	100.176466
99.8792395	99.9150986	99.9488323	99.7435626
99.7898813	100.082456	100.076874	100.112757
99.799304	100.072139	99.9040642	99.9023575
99.7828143	100.197771	100.222776	99.9708447
99.6994238	99.7211882	99.8699662	99.7430848
99.8734289	100.108554	100.223936	99.9751451
100.084653	100.116444	100.15574	99.7550302
100.136164	100.298367	100.254786	100.066249
100.235887	100.471946	99.9975437	100.021334
100.292266	100.568749	100.080817	99.8926418
100.020736	100.546749	100.045327	100.042358
100.003933	100.279705	100.284709	99.7631531

99.6747678	99.9798871	100.345946	100.006522
100.354927	100.484843	100.185431	99.8386484
99.8514427	100.334024	100.378652	99.9606513
100.03864	100.418385	99.8423631	99.9010833
99.9327916	100.028137	100.188446	99.9907538
100.127841	100.242076	100.285173	100.161016
100.012256	100.143148	100.804993	99.9647924
100.138677	100.315209	100.467725	99.90618
100.014141	100.260738	100.249683	99.8160317
100.107111	100.381819	100.141126	99.9421756
100.342834	100.684367	100.084992	99.9276818
99.8181493	100.236462	100.249915	100.125658
100.178723	100.530817	100.021667	99.8687509
100.187832	100.545838	100.110276	100.056215
99.9795909	100.143907	99.9952241	99.7298651
100.289298	100.719683	100.289137	99.8756515
100.506666	100.89911	99.7943344	99.9555871
100.23106	100.504767	100.36967	99.9065429
100.44591	100.733391	100.477124	99.9844086
100.365636	100.642917	100.344605	100.189662
100.237986	100.47994	100.05891	100.013867
100.565061	100.859508	100.219512	100.048102
100.328333	100.641089	100.382434	100.203674
100.459131	100.659214	100.178201	99.7758115
100.442447	100.648248	100.679966	100.187273
100.716479	100.892408	100.773727	100.12549
100.404514	100.682366	100.75841	100.607015
100.129695	100.432418	99.9472775	100.160681
99.9663149	100.270507	100.014118	100.247464
100.022979	100.109359	99.7688052	99.9546317
100.183526	100.110882	99.7836586	100.202878
100.189664	100.039751	99.5214041	100.015619
99.8810047	99.8274244	100.046377	99.9856825
99.7673626	99.8073188	99.9089837	100.185522
99.9365665	99.6959769	100.222297	100.195394
99.9433347	99.3554015	100.187252	100.342527
99.8940688	99.5930122	100.320004	99.9654597
99.918623	99.8840854	99.8662803	100.088707
99.8043514	99.8333646	100.239935	99.9821793
99.8163137	99.4908092	100.080958	99.931065
100.129538	99.7297907	100.396359	100.083293
99.6881909	99.7811207	100.229955	99.9296319
100.188248	100.195569	100.572047	99.8711929
99.8514137	99.6627723	100.612661	100.085045
100.18447	99.9377001	99.9825542	100.009249
100.034784	100.153378	100.223689	100.29189

99.9036701	100.061075	99.981858	99.872626
100.09554	100.22923	100.155224	100.062593
100.057134	100.159622	100.286816	99.833932
100.047533	100.025281	99.9243012	99.8267665
100.292603	100.461815	100.077244	99.9783577
99.9027257	100.006089	100.35296	100.103675
100.210126	99.7660416	100.294475	100.208133
99.9076051	99.7898027	100.389165	100.243005
100.059653	99.725069	99.9073591	99.7941234
100.079642	100.045539	100.017367	99.9506509
100.074291	100.13114	100.103934	99.8434861
99.9689907	100.413988	99.8853112	99.9785169
99.640499	100.222224	100.025258	100.224216
99.8366182	100.144391	99.4761478	99.7546333
99.9286966	100.123067	99.9428679	100.175968
99.7310034	99.6907982	99.8667445	100.083612
99.8592836	99.8792113	100.023865	99.9253326
99.7542985	99.5639202	100.13805	100.464978
99.8460621	99.5741252	99.6335005	99.8539956
99.7638998	99.5963632	99.6541559	100.020714
99.7785379	99.443744	99.7363135	100.134885
99.9549822	99.4233338	100.199088	100.150012
100.033839	99.6527195	99.7302793	99.9274027
99.6242869	99.6090053	100.295635	100.11307
99.8662092	99.8706817	99.6803813	99.9301096
100.199738	100.101286	99.9003966	99.9238995
99.9146881	100.094432	99.8416795	99.6979459
99.8344146	100.062903	99.7453648	99.8938042
99.9930728	100.036857	99.679453	99.8242187
100.075392	100.147437	100.252235	99.8761292
100.116159	100.189933	100.048002	99.7735822
100.087512	100.093213	100.083743	99.849537
100.340295	100.649314	100.136194	99.9906187
100.064689	100.290765	99.7001085	100.267527
99.7125877	100.26213	99.8498024	100.021988
100.133158	100.502483	99.2419754	99.857021
99.6050842	99.8728141	99.6177188	99.9019251
100.049107	99.93572	99.1349848	99.712277
100.342971	100.183383	99.5186191	99.6796339
99.5528277	99.6973477	100.381274	99.7686459
99.7232909	99.4903522	100.147334	99.9842493
99.9266504	99.7779221	100.488264	100.049058
99.7492617	99.6050451	100.049162	100.092529
99.9057163	99.8650461	100.21928	100.004472
99.9235024	99.8962705	100.146173	100.052402
99.5523555	99.3112303	100.164972	100.139184

99.9030405	99.4438963	99.9331204	100.356221
99.7657886	99.411301	99.8110444	100.219916
100.144805	99.7224796	99.5506466	100.062274
99.8607002	99.6114423	100.024329	100.190936
99.9559266	99.6754144	100.015046	100.084726
100.064847	99.8619998	100.091866	99.9987397
100.08767	99.9504945	100.116234	99.6356852
99.8392939	99.8198086	100.215798	99.9162562
100.147009	100.051631	100.084439	100.055268
99.8588114	99.9995398	100.096739	99.9130716
100.391922	100.567978	99.9001645	99.974536
100.007868	100.307824	99.8284507	100.02358
100.36784	100.528528	99.8936662	99.9224664
100.187775	100.353976	99.8853112	100.371826
100.132843	100.277819	99.768109	99.990778
99.95451	100.156728	99.6070429	99.8976258
100.431744	100.460596	99.8170786	99.7369583
99.9263356	100.156272	99.6894326	99.8482631
100.06044	100.026347	99.8205599	99.8008113
99.8542468	99.8121929	99.5026053	99.9500139
99.7206151	99.7661939	99.8022252	99.7289966
99.9077625	100.028937	99.4346048	99.4536802
99.961593	100.040969	100.069586	99.57645
99.6649616	100.130749	100.039943	99.838334
99.4019068	99.9252098	100.442875	99.6597069
99.3216042	100.066054	100.016111	99.7610535
99.3985608	99.8085105	100.560703	99.7348947
99.6554018	100.026896	99.9537441	99.7800373
99.6402654	99.7626976	100.812396	99.7032052
99.6550831	99.9792258	99.8283431	99.7326525
99.5496063	99.8227497	100.883004	99.5849676
99.8556804	99.9386751	99.7459304	99.8308601
99.6526932	99.6930495	100.674076	99.8447616
99.6321395	99.845811	99.995396	99.8662865
99.6334142	99.6806676	100.351553	99.8413236
99.487786	99.920257	99.8900413	99.5978227
99.4065274	99.6797389	99.898728	99.6716652
99.4944779	99.8592763	99.8993963	99.6718147
99.5473756	99.7890091	99.7762226	99.7151635
99.4462007	99.9236621	100.233725	99.9893075
99.975975	99.7123962	99.7766681	100.121297
99.5346292	99.7972121	100.674076	99.6828761
99.5757365	100.011109	99.5494762	99.8541787
99.6721315	99.5085594	100.13973	99.9866169
99.9420376	99.9727253	99.8067376	99.8458079
99.8324181	99.6972284	99.4345438	99.8111289

99.6257663	100.093758	99.7746635	99.6420684
99.6098332	99.8055698	99.7831275	99.7885576
99.349965	100.131832	99.7412529	99.7117255
99.251658	99.8309527	99.5955828	99.6003639
99.4227791	99.7596021	100.125252	99.7767488
100.044328	99.9606529	99.4040288	100.023688
99.781751	99.9023033	100.468044	99.8395298
99.7462202	100.105211	99.7641948	99.9132228
99.5242727	99.6763339	100.488759	99.6619491
99.5330359	99.8679436	99.5109427	99.68751
99.3456631	99.7952	100.357344	99.4945329
99.4242131	99.9899051	99.6677496	99.8017117
99.5707972	99.7591378	100.589659	99.6470012
99.3609588	99.897041	99.2804097	99.6473002
99.918138	99.6357833	100.745575	99.9549274
99.7618346	99.7842111	99.9532987	100.176156
100.355341	100.067756	99.6719816	100.260462
100.088144	99.8489064	100.140398	100.021595
100.403618	100.296666	99.4913418	100.32698
100.632576	99.9078752	101.03892	100.194243
100.675118	100.187551	100.390309	100.194392
100.716384	100.274224	100.214569	100.142075
101.007641	100.214482	100.1845	100.125333
99.9790023	99.6546656	100.684545	99.8742089
100.47691	100.014669	100.08605	100.046857
100.523116	100.276855	99.8359162	100.166589
100.216724	100.323752	100.58409	100.072119
100.078903	100.016836	100.276713	99.9638961
100.195214	100.396495	99.5984784	100.036543
100.552911	100.36616	99.5617267	100.124436
100.207004	100.10645	100.270254	99.7623988
100.23186	100.196683	99.8777908	99.9656899
99.9092155	100.171455	99.2841962	99.9015635
100.044487	99.9716419	100.112556	100.21233
99.8639656	99.8300241	100.128593	99.8492459
100.028554	100.210303	99.5719726	99.9396806
99.9586079	100.325764	99.6902461	99.7676306
99.9493668	99.843025	100.298987	99.6788402
99.9219619	99.8759918	99.6757682	99.9414743
100.145503	100.171455	99.4038061	100.025631
100.112362	100.061875	100.194746	99.9792924
99.8529717	99.8784682	100.10966	99.8147164
99.8963097	100.557924	99.4104882	100.148951
100.237915	100.317251	99.3975694	100.363154
100.013099	100.19173	99.8601945	100.200371
99.9264232	100.10258	100.279386	100.020399

99.8977436	99.8106774	100.230384	100.018157
100.194895	99.9993463	100.005419	100.293945
100.302603	100.190337	99.956417	100.314424
100.361874	99.7401006	100.61906	100.209639
100.323475	99.8849687	100.156435	100.147456
100.494277	100.429926	99.4719637	100.280791
100.217202	100.244198	100.459357	100.32115
100.075079	99.9377465	100.31636	99.9996215
100.085116	100.08927	100.058431	100.022492
100.149486	100.088186	99.8098559	100.106349
100.204136	99.9125184	100.45958	100.04297
99.8462799	99.9547715	99.9332523	99.9452113
99.9641845	100.398972	99.8815773	100.148203
99.8732067	99.8612883	100.854493	100.080041
99.9643438	99.8023196	99.9241201	100.149997
99.9245112	100.261068	99.652158	100.024286
99.8663555	99.7291117	100.127925	100.094092
99.9797989	99.9656057	99.7958235	100.14342
100.613457	100.261068	99.8370299	100.24372
100.325387	100.192658	100.292973	100.049398
100.414453	99.9115897	100.113669	100.248504
100.506865	100.156287	99.8103014	100.53924
100.439946	100.307191	99.6185247	100.50486
99.9656185	99.7792583	100.138616	100.137291
100.290653	100.045933	99.3922238	100.578104
100.346578	100.445249	99.8178745	100.240282
100.423853	99.9564741	100.1894	100.308594
99.9836228	99.8275477	100.139952	100.099772
100.802264	100.183991	100.016111	100.370478
100.483284	100.312298	99.9127605	100.182434
100.313119	99.8337386	99.8746724	100.018456
100.527892	100.195491	99.5096959	100.134626
100.148124	100.093446	99.6814676	100.192185
99.9221731	99.9602753	99.7230756	99.8314285
100.281401	100.256347	99.4625254	100.128197
100.190957	100.182639	99.7072779	100.135373
99.9887322	99.8757277	99.8296542	99.914105
100.353055	100.316583	99.3296915	100.074076
100.407194	100.395711	100.008101	99.9764489
100.344457	100.084619	100.188105	99.7982383
100.199237	100.311009	99.4605229	99.8939218
100.236657	100.077961	100.341632	100.257669
100.496683	99.7992322	101.205386	100.044025
100.411971	100.40748	99.6449773	100.083943
100.213568	100.409493	99.5815641	100.302371
100.484263	99.8659722	100.378345	100.205192

100.341113	100.120699	99.2527057	100.072132
99.7923989	99.855907	99.520376	99.8433889
99.4732976	99.8772762	99.5842341	99.5195599
99.9963753	100.291498	99.3730795	99.8670108
99.8549772	100.007659	99.7164005	99.6781853
99.6228166	99.9742118	99.3096663	99.8417444
100.039209	100.280658	99.3704095	100.179328
100.026789	99.5592159	100.499609	99.9783924
100.15927	100.028564	99.3975547	100.255277
99.9670766	99.8422803	100.481586	100.137167
100.192231	100.140675	99.4823281	100.022646
100.023286	99.5956055	100.634222	100.140157
100.330285	100.068979	99.5962493	100.174693
100.104335	99.6188328	100.794424	100.318518
100.261338	99.8768116	99.7088354	100.356193
100.325508	99.8171947	100.563022	100.147633
100.216275	100.033829	99.5800066	100.179926
100.130927	99.7823536	100.476246	100.126253
100.091915	99.9056136	99.6467573	100.153463
100.050674	99.5671132	100.564357	100.258117
100.195256	100.203853	99.6469798	100.254081
100.075036	99.7106584	100.220368	100.37533
100.110863	100.00162	100.102664	100.682713
100.135226	99.9921743	100.033244	100.678826
99.9414402	99.7719787	100.209243	100.410613
99.9567265	99.8806829	99.5895742	100.111153
99.7861889	99.6183683	100.065284	100.078262
99.9656435	100.054424	99.6038143	99.9801865
99.842557	99.6857277	100.64379	99.8788217
100.019942	100.094994	99.809629	99.8867455
99.8564102	99.7750757	100.681838	99.6548624
99.9487649	99.8190529	99.9057499	99.9740568
99.8283854	99.7777081	100.624432	99.9266635
99.8110291	99.8450675	99.8300992	100.045221
100.045419	99.7216527	100.215696	100.102034
99.9194662	100.092207	99.9506954	99.8819614
99.7858704	99.6716364	99.8347717	100.003808
99.957045	99.8495582	100.152727	100.392373
99.9430325	99.6536739	99.8216441	100.227768
99.8928744	99.5602998	100.492933	100.061368
100.140003	99.9167627	99.8712621	100.306109
99.735553	99.656616	100.867627	100.114592
100.021693	100.001156	99.7951663	99.9360824
100.000356	99.9014326	100.031019	100.116685
99.7398523	99.9940325	99.7215181	99.9157496
99.8178761	99.9368931	99.7637935	100.234645

99.6818918	99.761449	100.141825	100.110705
100.216434	100.058759	99.6547674	100.17574
99.9033837	99.8538939	100.819567	100.051202
100.028381	100.142068	99.6296246	100.178431
99.6787071	99.7585068	100.519411	99.8909317
99.8376208	100.026241	99.671455	99.9329427
100.034273	99.9037554	100.108672	99.7327548
100.05338	100.197969	99.5962493	99.7859788
99.8537033	99.8927611	99.9996459	99.9704686
99.8400093	99.8682949	99.9248851	100.007845
100.070896	100.033364	99.5201535	100.176637
99.7603932	99.9548556	100.011661	99.8927258
100.068189	100.009363	99.8730421	100.109957
100.145735	100.055508	100.197005	100.102632
100.166117	100.154611	99.8781597	99.9324942
100.054177	100.07378	100.525196	99.7489014
100.122487	100.244269	99.8681471	99.8403988
100.044782	100.031351	100.476023	99.7711777
99.9538603	100.139746	99.9039699	99.7166082
99.7603932	99.984277	100.464898	99.602685
100.13236	100.514945	99.4380501	99.8030224
99.8544995	99.9293055	100.743249	99.7034517
99.9291793	100.255263	99.3977772	99.8273918
99.9656435	100.170561	100.561464	99.736642
100.054017	100.21717	99.8260941	99.6236158
99.8753589	100.027944	99.9491379	99.696425
99.9715351	100.124416	99.808739	99.6994151
99.8994029	99.9839673	99.6961528	99.7181033
99.8901675	100.28964	99.8292092	99.7137676
99.9099123	100.167154	99.9793982	99.7091329
99.8661234	100.142378	100.093542	99.7888194
100.048444	100.199053	100.166078	99.8353156
99.8282261	100.186975	100.241951	99.9257665
99.7750426	100.351579	99.955368	99.9148526
99.6720194	100.147798	100.264646	100.024291
99.7229737	100.266877	99.9044149	99.885998
99.7307761	100.221351	100.62599	99.7390341
99.9798152	100.558458	99.5584239	99.8073581
99.3919687	100.020519	101.171598	99.9720887
100.107187	100.124648	100.166269	100.005592
99.9602267	99.4567098	100.235579	99.9819603
99.8379454	99.9361956	99.887469	99.8169847
99.7026079	99.5951867	99.7662322	99.9457644
99.5421137	99.7723442	100.027203	99.9508497
99.5548513	99.8490867	99.5511708	99.784528
99.5167976	99.9818388	100.029432	99.7320289

99.5846255	99.5800238	99.607109	99.7734598
99.6171065	99.9315539	99.9980083	99.7212599
99.8299843	99.5560418	99.7468433	99.9779219
99.7728242	99.92815	99.7599921	99.8216213
99.7916122	99.5117911	99.9980083	99.8974533
99.5492786	100.093858	99.8908119	99.5043835
99.5409991	99.6544455	99.7439461	99.8368775
99.3074226	99.745113	99.9596761	99.7345716
99.9185109	99.5599099	99.3755558	100.066916
99.7615195	99.5653252	100.287728	100.331804
100.302392	99.7817823	99.8790002	100.438597
99.9484444	99.5769294	100.351244	100.133175
100.150654	100.174623	99.5184101	100.075591
100.339172	99.6522794	100.753509	100.107599
100.170716	99.7078248	100.190115	100.060933
100.420692	99.7839484	100.06665	100.043434
99.9839506	99.5894619	100.866723	99.7688232
100.160685	99.99236	99.9385043	99.8617061
99.8156545	99.7726537	100.190784	99.7082473
99.7225105	99.6479471	100.399382	99.5779719
99.8947871	100.043728	99.9291441	99.7424988
99.7362035	99.682605	100.335867	99.8862355
99.5080405	99.7392335	99.8723144	99.8539284
99.7051555	100.150332	99.4885467	99.8027755
99.981403	99.7375316	100.264774	99.8346339
99.6164696	99.8156666	99.9790651	100.028028
100.075502	100.259876	99.5560737	100.161743
99.7414577	99.868427	100.654336	99.9801655
99.679521	100.001643	99.7221056	100.06213
100.159252	100.182669	99.7764839	99.9384354
99.999395	99.7732725	100.344112	99.8256597
100.004649	99.8602267	100.21463	99.9046326
100.146674	100.230324	99.6142406	100.026981
99.8963793	99.6343315	100.675731	99.8560224
99.8686749	99.9012283	99.7517462	99.6855127
100.100022	100.22135	99.9364985	100.087108
99.911346	99.7916846	100.365284	100.016661
99.8462248	99.8223197	99.8346508	100.137663
99.8787058	100.232335	99.3637442	99.7570071
99.9183517	99.9724007	100.16872	99.8463004
100.219756	100.030731	99.8491368	100.250289
100.425151	100.437961	99.7499633	99.9807637
100.359233	100.073899	100.057958	99.9859987
100.107506	100.090764	99.7024938	100.130334
100.205108	100.404077	99.554068	100.132278
99.9201031	99.8650231	100.51839	100.251037

99.9821991	100.006285	99.7820554	100.335992
99.9941407	100.353173	99.7466204	99.9255724
100.108779	100.095715	100.145988	99.925722
99.9947776	100.089216	99.9746079	100.006041
100.167691	100.390926	99.547605	100.083219
99.9419164	99.8979791	100.311575	99.8970046
99.9382543	100.011391	99.6842192	100.103262
100.471802	100.145381	99.8027815	100.216636
100.013406	99.7896732	100.149554	100.076339
100.144763	100.100975	99.8009986	100.170418
100.679426	100.549362	99.6659445	100.24625
100.397128	100.14832	100.215298	100.176401
100.24348	100.177254	100.02698	99.9571317
100.215298	100.425429	99.7760381	99.9949729
100.192529	100.008296	100.154903	100.055997
99.9239244	100.026708	100.24271	99.7541653
100.267363	100.335226	99.7998843	100.2464
100.014521	99.925365	100.195464	100.178196
99.8080119	100.105308	99.9157724	99.9284143
100.179473	100.261268	99.7880727	100.046126
100.125816	100.026863	100.23023	99.967452
100.031398	99.9488829	100.423228	100.141103
100.064198	100.294998	100.027426	100.414666
100.082827	100.032124	100.108325	100.327916
99.8982899	99.8591437	100.283271	99.9261707
100.068019	100.233882	99.6768647	100.130483
99.9782186	99.9569284	100.037009	100.045677
99.8409706	99.9788991	100.21307	99.8476465
100.231698	100.220731	99.5883887	100.180739
99.8717001	99.9055605	100.273688	100.062728
99.9129382	100.055177	100.330518	99.8591634
100.028692	100.342962	99.7671237	99.9490549
100.013247	100.112115	100.1656	100.145291
99.9868165	100.054868	100.307563	99.9607214
100.325638	100.4474	99.5279932	99.9124103
100.16785	99.9662118	100.346341	99.8442064
100.000987	100.158996	99.8137018	100.013669
100.241729	100.406243	99.5649882	100.12106
100.142534	99.7837937	100.630044	100.151273
100.226443	99.9629626	100.097182	100.110291
100.077095	100.431463	99.7109626	100.070356
100.015158	100.045275	100.827722	99.8507875
99.8701079	100.085194	100.001574	99.9480079
100.020094	100.204021	99.7470661	100.205119
99.9694615	99.8418147	100.558951	100.026233
100.118014	100.173076	99.7504091	100.100868

100.3457	100.166578	99.881006	100.238921
100.006713	99.995915	100.610829	99.7350093
100.25971	100.29533	99.6079572	100.070167
100.28171	100.199604	99.8009627	100.140427
100.112407	100.024881	100.142006	100.071512
100.120378	100.190465	99.7301717	100.138932
100.213638	100.211996	99.6026145	100.108287
100.266725	99.9996325	99.9953038	99.8276933
100.449897	100.407631	99.621314	100.016799
100.435231	100.290684	100.237952	100.066579
100.141581	100.094429	100.214578	99.8100535
100.559418	100.420642	99.8724214	100.050882
100.564679	100.166611	100.04695	99.9076706
100.444158	99.892444	100.221033	99.7581803
100.643591	100.295021	99.6331125	100.247313
100.574244	99.9437148	99.8931245	100.460934
100.379275	100.000717	99.9022516	100.512807
100.662084	100.076926	99.6371195	100.469007
100.531679	99.8586765	100.48995	100.371091
100.53152	100.076616	99.8034114	100.280201
100.143653	99.8592961	100.75063	100.133551
100.207102	99.9271409	99.7170376	100.310099
99.8842788	99.6992878	100.639101	100.217116
100.198494	99.9065396	99.7555496	100.47095
100.306101	99.6714063	100.497519	100.338651
100.288246	99.8448907	100.084126	100.193496
100.244087	99.7022308	100.122638	100.279453
99.9875823	99.8131369	99.9091526	100.100214
100.149233	99.8199523	99.5620989	100.199924
100.039393	99.962922	100.035152	100.42416
99.8171634	100.045792	99.6130773	100.229822
99.6367011	99.713848	100.268673	100.113369
99.8654673	99.9819743	99.5193573	100.123236
99.8233807	99.9229586	100.348813	100.096327
99.9258871	100.265745	99.5692226	100.129215
99.6497735	99.9237331	100.654683	99.9428009
99.8174822	100.092571	99.5202477	100.046099
99.899583	99.6061948	100.800718	99.8856956
100.121654	100.059732	99.4750573	100.200821
99.8941628	99.9526988	100.490172	100.181686
100.055654	100.041455	99.943435	100.054321
99.7675841	99.7570642	100.412703	99.7825472
100.073828	99.9463481	99.7864928	99.9408575
99.9058003	99.8311049	100.045614	100.081528
99.947887	100.195112	99.6987833	99.8360648
100.005915	99.9051455	99.6843135	99.7517522

99.9474087	99.9746942	99.6800838	99.8658134
99.9592057	100.042539	99.6593808	99.8001871
99.8401197	99.913355	99.9213963	100.053424
99.8903367	99.8569726	99.8583968	99.8433898
99.9257277	99.9175373	100.334121	99.9779311
99.9397566	100.058493	99.5262583	100.121741
99.8378879	99.7720892	100.048508	100.2071
100.123886	99.9511499	99.6847587	100.098121
100.066495	99.8630136	100.384209	100.021433
100.268957	99.9345759	99.744419	100.027263
100.064263	99.9889447	100.517331	99.7900218
100.045611	99.9691179	99.6268793	99.7164725
99.959684	99.9517694	100.202334	99.7371022
100.059002	100.042694	99.5876994	99.8282913
99.7773086	100.028753	100.211016	99.8489209
99.8574964	100.09412	99.425192	100.087956
99.8292792	99.9990129	100.562299	99.9611882
100.150349	100.360542	99.761115	100.178846
99.8653079	99.9065396	100.769552	100.090049
100.025683	100.158402	99.5378341	100.240137
99.7889462	99.8481435	100.291824	100.178248
99.9469304	100.105117	99.7655672	100.189161
99.6748022	99.9393777	100.300061	99.8972063
99.971481	99.9423207	99.7706873	99.941306
99.6873963	99.9669493	100.092363	99.840998
99.8192358	100.215249	99.7673481	100.024124
99.6580633	100.04037	100.252199	99.8100535
99.9458145	100.351713	99.7136984	99.7963004
99.9316262	100.266675	100.115737	99.7136322
100.103161	100.330802	99.603505	99.9553581
99.8066417	100.022557	100.257319	99.8803139
99.8873077	100.231823	99.9563465	99.7841916
99.7049324	99.9610632	100.050289	99.5970298
100.002727	100.154219	99.6832004	99.7608711
99.7687	99.8552688	100.088801	99.7722324
99.9372059	100.313453	99.4641492	99.701374
99.8772643	100.158402	99.6889883	99.6879199
100.049756	100.077855	99.7900546	100.032196
99.6540778	99.9159883	100.300284	99.9481825
99.9509159	99.9378287	99.7673481	100.190955
99.6776718	99.8063214	100.604818	99.9185834
99.9274813	99.881911	99.717928	99.8058677
99.6512082	99.8817561	100.620846	99.6417274
99.9351334	99.953938	99.6295507	99.9383162
99.4619779	99.8495376	100.464127	99.7526492
99.7696565	100.077081	99.3283553	99.7535461

99.8624384	100.114566	100.318983	99.8181259
100.083553	100.214319	99.560318	99.901392
99.9171191	99.9743844	100.542932	99.8549006
99.9799302	100.063915	99.6854265	100.021881
99.9027714	99.9296192	100.75063	99.956554
99.9112206	99.9320976	99.8063054	100.08377
99.7994679	99.8792778	100.391332	99.8395031
99.9459739	100.000252	99.8750928	99.9143977
99.7334684	100.049509	100.467911	99.7587783
99.7472863	99.5778983	99.7066573	99.8891874
99.797047	99.6531732	99.4916321	99.971553
99.6588781	99.5683423	99.622942	100.030739
99.7824505	99.6965945	99.9265694	99.9602721
99.8715221	99.5550979	99.5570746	99.9268932
99.9325619	99.6633998	99.7381037	99.947446
100.071726	99.9455548	99.7676377	100.049901
99.8511203	99.7943345	99.8279808	100.171981
99.7613852	99.6766442	99.5126672	100.106768
99.9523003	99.8481501	99.7576514	99.8238203
99.9901184	99.8100936	99.6537509	99.8176391
99.8925875	99.6830149	99.8917234	99.9747981
99.8131363	99.5706894	99.9720391	100.041247
99.74513	99.7596309	100.346846	99.9993687
99.8048428	99.8872126	100.051505	99.9106673
99.8018572	99.665244	99.7593512	99.9621265
99.7827823	99.7120183	99.895548	100.035838
99.7720008	99.8828537	99.8407293	99.8165573
99.9347182	100.016974	99.7208931	99.8375737
99.9403578	99.7115154	99.6318659	100.01714
99.9255954	99.896098	99.9605655	100.12284
99.6454428	99.5250886	99.5883085	100.200569
99.5981701	99.4597051	99.8069457	100.14911
99.840007	99.5924839	99.8182069	100.036302
99.9901184	99.9292927	99.8505032	99.9423464
99.9965873	99.9522608	99.7703999	100.029039
100.039879	99.9728817	99.9932867	100.085134
100.041206	100.018315	99.7072947	100.224213
100.072389	100.056707	99.8549652	100.01714
99.8317136	99.990485	99.8883238	99.9205574
100.098099	100.239278	99.2678955	100.084979
99.8842941	99.8473119	99.6607626	100.10955
100.113027	100.108678	99.7100569	99.8119214
100.245723	100.381948	99.7659379	99.6593983
100.122316	100.325953	99.7672128	99.9459006
100.240746	100.32545	100.062766	100.210923
100.090967	100.117731	99.8375422	100.050673

100.028434	100.167188	100.040669	100.052837
100.049997	100.030553	100.05533	100.234103
100.062935	99.6582027	100.176441	99.7870417
100.087152	99.9735523	99.6478016	99.8462275
99.9819909	100.108678	99.2698078	100.083743
100.108051	100.292926	100.280553	99.969544
99.8874456	100.004735	100.150519	99.9398739
99.875503	99.6734588	99.9204076	100.000141
99.9977484	99.9529314	100.052992	99.8981502
100.097436	100.022841	99.9684271	99.9831429
100.101582	99.967014	99.8915109	100.085752
100.113857	99.8820154	100.049805	99.9703167
100.004051	99.8905656	99.9966863	99.9060314
99.8098189	99.725598	99.9962613	100.063963
100.055637	99.9294604	99.7606261	100.224213
100.032581	99.8508325	100.023671	100.168736
100.286692	100.255875	99.8590022	100.0567
100.149519	100.048827	99.7200432	100.166881
100.098099	99.8820154	100.199813	99.9608902
100.110871	99.9416988	100.078489	100.040011
100.032415	100.211615	99.8483784	100.062418
99.8829671	100.090237	100.11801	99.9247298
100.230794	100.387983	99.8600646	100.010959
100.047343	100.212454	100.275454	100.097187
99.8595796	99.9547755	100.060004	100.020385
99.9401919	99.9894791	100.332185	100.006632
100.104734	100.11572	100.190889	99.933229
100.206412	100.206753	100.246982	99.8567357
99.9393625	100.251684	100.331973	99.8782156
99.8567598	99.94304	100.375318	100.069217
99.9715411	99.9589668	100.017934	99.9615084
100.066086	100.069783	100.159442	99.7998679
100.255841	100.673659	99.7899477	100.110323
100.194137	100.516235	99.9584407	100.163636
100.17274	100.307176	100.362144	100.055927
100.101417	100.036253	100.2064	100.124849
100.219183	100.183115	99.7729496	100.089461
100.231955	100.173056	100.030257	99.9069586
100.222003	100.091913	99.8050334	100.040474
100.099758	99.9442136	100.194926	99.9540908
99.9448362	100.004903	100.353433	99.8677074
99.9498123	99.8092553	100.696368	99.9806703
99.9673944	100.117061	100.430136	99.9570269
100.16992	100.317235	100.027283	100.110477
100.136747	100.250342	99.9282692	99.9486822
99.8569257	99.8134466	100.337922	99.882388

99.8864503	99.6679264	100.274392	99.8174845
100.126794	100.170876	99.6803103	100.148338
99.6918861	99.6392582	100.314975	99.9888605
99.8494616	100.051342	100.236784	99.9364742
100.08483	100.375409	100.116947	100.052991
99.9254296	100.220668	100.568033	100.120058
99.9595986	100.11069	99.9941366	100.115731
100.146367	100.347579	99.9843627	100.021312
100.26148	100.391839	100.071265	99.8168664
99.9529638	100.220668	100.456483	99.916076
99.840007	99.8677652	100.626676	100.254192
99.9103355	99.9004569	99.8441289	100.143702
100.073385	100.171547	100.180478	99.8017223
99.9196241	99.8444618	100.11461	99.9139125
99.9634135	99.9541049	100.102712	99.8451458
99.9143163	99.7929933	100.153281	99.7893597
100.003056	99.6794942	100.490267	99.8996956
99.9433764	99.6733196	100.220609	100.055061
99.7992435	99.6209616	100.185142	100.002511
100.01893	99.9458501	100.043062	99.9886009
100.222177	100.217542	100.120792	99.7406901
100.255886	100.190691	99.9281664	100.015339
100.195443	100.39425	99.8636039	100.162324
99.9543358	100.038148	100.387537	99.972836
100.088007	99.8569085	100.103802	99.9320328
100.107602	100.021534	100.194699	100.07469
100.025406	100.308161	100.236113	100.163406
100.281624	100.480339	99.9423956	100.203591
100.043173	100.456173	100.273491	99.996638
100.183653	100.228449	99.6503776	99.9876736
100.380923	100.346087	99.4443721	100.241458
100.184317	100.440735	99.2976197	100.339911
100.001661	100.049392	99.6909415	100.094319
99.9714391	99.9669947	100.480346	99.9964834
100.212048	100.343067	99.9436699	100.053979
100.300222	100.335683	99.622981	99.8824197
100.248579	100.210158	99.6847826	100.145632
100.275812	100.100071	99.6960386	100.00823
100.274317	100.139676	99.8890891	99.8535173
100.322971	100.391229	99.7249218	100.128012
100.248413	100.307993	100.071733	100.222756
100.060609	100.055936	99.8531974	100.140531
99.9910332	100.028918	99.9090525	100.095555
100.112251	100.07792	99.3549614	100.140377
99.9179704	99.7716588	99.7070822	100.035741
99.8694833	99.691108	100.062813	99.983346

99.9158118	99.774176	100.064512	100.176543
99.8115313	99.8082424	100.111448	100.113947
99.8792804	99.8664739	99.96512	100.184117
99.9254428	99.8845979	99.9037431	100.208228
100.030554	100.060299	99.7826883	99.9835005
99.8321217	99.7763576	100.234838	99.8372888
99.926273	99.9550799	100.089148	99.8666548
99.9852214	99.8683199	100.068335	99.7315712
100.000332	100.120041	99.9028936	99.8564539
99.8922324	100.031603	100.118244	99.9403789
100.065092	100.157632	100.106775	99.9224502
100.029889	100.131788	100.304498	99.7767021
100.028727	100.210158	100.348672	99.8263152
100.035369	100.057111	100.412598	99.8488806
100.002657	99.9473604	100.116545	99.9213683
99.9800738	100.092184	100.358442	99.9324964
99.8938929	100.10477	100.46548	100.026931
100.01428	100.072886	100.268606	100.10251
100.011956	100.098729	100.226768	99.9893737
100.105609	100.070033	99.7060203	99.9649536
100.106439	100.100071	100.036903	99.8655729
100.004649	99.7842449	99.6457053	99.9159587
99.7002766	99.5246361	99.9186094	99.888602
99.977417	99.7246708	99.9670314	99.9912284
99.7994095	99.5670932	100.526219	99.8587723
99.8590221	99.7449764	100.085962	99.9226047
99.8742988	99.7349075	99.7425491	99.9105492
99.6765312	99.5526611	99.9298654	99.872528
99.5415312	99.494933	100.033718	99.888602
100.015443	99.8713405	99.8330216	99.8765465
99.9802398	99.9755532	100.017365	100.023376
100.055129	100.220898	99.9855081	99.8912295
100.135498	100.187335	99.8238894	100.003284
100.066753	100.058621	99.7690962	99.9547528
100.027066	99.738935	100.050071	99.9122493
99.9510147	99.8406305	99.8449147	99.9212137
99.8111992	99.4222694	99.8916376	99.9799457
99.8397601	99.4986249	99.6875435	99.9655718
99.9730996	99.9231952	99.9731903	99.9388333
100.041679	100.180287	100.2805	99.9352785
99.804225	100.029254	100.077043	100.009775
99.8294649	99.8810738	100.18854	100.019513
99.9380627	99.7716588	100.037328	99.9527435
99.9609778	99.9102735	100.332107	100.094009
100.026402	100.001397	100.027559	100.016421
99.9008671	99.7933069	100.277739	99.9954015

99.8884132	99.7929713	100.044549	99.9528981
99.7130626	99.6301914	99.7242847	99.955371
99.7630442	99.7966632	99.7578402	100.009775
99.861845	99.8768784	99.8782579	100.116575
99.9722694	99.7884403	99.6803227	100.195399
99.8244833	99.7119169	100.278163	99.9164224
99.9875461	100.05795	100.265421	99.9196681
100.052804	100.070033	100.233352	99.9681993
100.204908	100.249258	100.061327	100.067116
100.005314	99.9758889	100.590145	99.9107038
99.9528413	100.104099	100.260111	100.138368
99.9035239	100.207976	99.9587487	100.119821
99.9755904	100.153772	99.7631496	100.111011
99.9516789	100.184482	99.5441892	100.015958
99.9845572	100.082619	99.9806235	99.8881383
100.087841	100.306819	100.045823	99.8380616
100.113745	100.293897	100.14118	100.064489
100.156919	100.186831	100.381803	99.8295609
100.334594	100.32897	100.020763	100.138677
100.128856	100.151423	99.7852368	100.324919
99.8706457	99.9695119	99.7153649	99.9498069
99.9940221	100.037141	99.7744056	100.013639
99.8914022	99.7726657	100.134809	99.981955
100.061439	100.002571	100.219123	100.065107
99.8630429	99.8997975	100.4476	100.021589
99.9259217	99.9831216	100.306841	99.9171234
100.031438	99.9024799	100.398696	99.9667293
99.8547476	99.8552014	100.53095	99.8206932
99.7359582	99.6689376	100.29217	99.8973428
99.7001223	99.8625782	100.26793	99.8573181
99.738115	99.8397773	100.325978	99.9080058
99.9028606	99.9970369	100.482258	99.8760169
99.9559508	100.03543	100.405713	99.9563754
99.7225198	99.5718658	100.541156	99.9645658
99.62928	99.5083248	100.141417	100.040134
99.6609683	99.6038877	99.989814	100.001345
99.7792599	99.7794217	100.15375	100.099012
99.7356264	99.7588003	100.019794	100.040906
99.8487749	100.037442	99.9256007	100.085104
99.8164231	99.9459024	100.466311	99.9277864
99.9737029	99.9692063	100.307054	99.9854281
99.8786382	99.7738891	100.193723	99.9280954
99.780919	99.9935162	99.6238828	99.9573026
99.7779327	99.9975399	99.9540927	100.06795
99.9144741	100.124119	100.001509	100.031789
99.7995006	100.029562	99.927089	100.044461

99.7643283	99.8848762	100.134188	99.9903732
99.9541259	100.005252	100.009588	100.213677
99.9043538	99.6897267	100.120367	100.09793
100.167814	100.195875	99.9372951	99.9709018
100.145417	100.316586	99.9747175	100.145991
100.174284	100.286408	100.069549	100.238094
100.207466	100.312227	99.9317668	100.022053
100.100456	100.122945	100.025961	99.8889979
99.9866436	99.9683681	100.030426	100.072895
99.9187877	99.7809306	100.231146	100.015408
99.9816664	99.928634	100.079543	99.9732198
99.7863939	99.8206647	100.334908	99.9161962
99.7082518	99.7919958	100.408689	99.8860618
99.8303593	99.8459805	100.05828	100.041215
99.9106582	99.7552795	99.9762059	100.025298
100.068768	99.8724698	99.7737849	100.020817
100.048527	99.8790083	100.133337	99.7880862
99.9405215	99.7643328	100.429527	99.8276473
100.15703	100.130993	99.3032411	99.9268591
100.117212	100.144908	99.8118451	99.9276318
99.8814586	100.009276	100.396995	99.9699746
99.8240548	99.7490763	99.8379983	100.221713
99.9624212	99.8144615	99.6963886	100.123119
99.9144741	99.8067494	99.7272196	99.9226867
100.077063	99.8785054	99.7325352	99.6258239
100.05732	99.7800923	99.9117799	99.7253448
100.0472	99.6067379	99.6823552	99.919905
99.8816245	99.6209885	99.8473539	100.029934
100.033429	99.7061568	99.7076579	99.9724471
100.033761	99.7380111	99.2732607	99.8554637
99.786228	99.609588	99.8092936	99.7367804
99.8360001	99.7039773	100.144181	100.046779
99.9433419	99.9254486	100.032339	100.135173
100.137785	100.166032	100.154175	100.11972
100.187723	99.9908337	99.7036179	100.112302
100.331564	100.194869	100.017668	99.9953184
100.166653	100.101988	100.017668	100.051569
100.15056	100.304012	99.8579852	100.268537
100.238656	100.371577	99.8543706	100.092212
100.315139	100.538392	99.8803111	100.040288
100.066777	100.185816	99.7780374	100.254938
100.217586	100.238459	99.8054663	100.032252
100.059974	100.027047	100.052539	100.090976
99.9821641	99.9584765	99.8811616	100.160053
100.085192	99.9790979	99.8775469	100.077377
100.226047	100.292611	99.8920056	100.220786

100.094483	100.272493	100.240501	100.064087
99.9707166	99.9876483	99.8817995	100.050951
100.061965	99.9794332	99.9485644	99.8651995
100.174118	100.237621	99.6079357	100.085413
100.046868	99.9484172	99.6232449	100.034725
99.9639144	100.128143	100.015329	99.8809621
100.096972	99.9821157	100.043396	99.9860463
100.120033	100.012796	99.6806542	99.960857
100.044379	100.05186	100.069124	99.7911769
100.050186	100.147926	99.7920708	99.9251592
100.014018	99.937352	99.7988749	99.9579208
100.153546	99.8372624	100.044459	99.952512
100.213107	100.24265	100.182454	100.248602
99.8970539	100.00039	100.10038	100.272401
100.127499	100.077511	99.7270069	100.152636
100.364414	100.260757	99.9204976	100.133937
100.135462	100.250195	99.9275143	100.270546
100.217918	100.264445	99.7810142	99.9971728
100.197511	100.242986	100.357234	99.7574882
99.9839891	100.018497	100.079755	100.002427
100.040729	100.131831	100.071888	99.9495759
100.301037	100.35632	99.6832057	99.9756924
100.113397	100.442327	99.7176513	99.9749197
100.022977	99.8880617	100.601117	99.6405048
99.8763155	99.5530885	100.949188	99.6757389
100.017834	100.27484	100.052964	99.9480305
99.9980912	100.547781	99.9217734	99.9817193
100.016839	100.352464	100.386789	99.9613206
99.9172945	100.052866	100.229232	100.07614
99.8391523	99.9487525	99.760602	99.9877461
99.6395663	99.7792541	100.05424	99.80014
100.014682	100.081535	99.9411224	99.8758624
100.041725	99.9489202	100.002997	100.057287
100.162802	100.026747	100.140899	100.000687
100.298078	100.250266	99.9056425	100.079974
100.321481	100.256979	100.131123	99.9727127
100.49875	100.456501	99.9844863	99.943038
100.238656	100.186164	99.9156308	99.7974469
100.124792	100.182304	99.6914256	99.9166091
100.178072	100.1865	99.9768357	99.9615847
100.129938	100.024398	99.8758902	99.8563325
100.256748	100.169887	99.8163855	99.9099632
100.326129	100.411025	99.7855705	100.056791
100.092758	100.22778	100.140474	100.132059
100.061055	100.089004	99.9923494	100.077656
100.123298	100.094541	100.014451	100.168071

100.044457	100.153442	100.183402	100.146742
100.111016	100.136158	99.9910743	100.155242
100.214091	100.131123	99.9451707	100.092802
100.052756	100.177774	99.5356508	99.9737946
100.044125	99.8760567	99.7645314	100.056636
99.9639554	99.955765	99.8144728	100.169616
99.7226172	99.706236	99.8933166	100.009342
99.920634	99.7436569	99.7073643	100.018152
99.8474359	99.8367898	99.3418354	100.052
99.510326	99.4847307	99.7313787	99.8462864
99.5831922	99.5431275	99.8867285	99.9057902
99.7946535	99.8970325	99.9878865	99.9744128
100.002629	99.9129742	100.094995	99.9901774
99.8788065	99.8085982	99.9553715	100.016452
100.064707	100.078264	99.9330572	99.9071812
100.042797	100.176263	99.702689	99.8322219
99.8439502	99.9676793	99.7696318	99.9530841
100.011592	100.105281	99.3711627	99.8795158
100.065868	100.093535	99.3820011	99.8343857
99.911173	100.029936	99.4474563	100.104239
100.108194	100.259999	99.5647656	100.039017
99.9700967	100.415221	99.4415058	99.8778157
99.8167291	100.135151	99.4344928	99.9006899
99.8683496	100.065175	99.8518758	99.8657604
99.8527473	99.8220228	100.071406	99.9006899
99.6479252	99.6280379	100.082669	99.9170728
99.6856032	99.5280249	100.247795	100.05169
99.8522493	99.8507178	100.084369	100.063591
99.9129988	99.8312522	100.155987	100.097748
100.016738	99.9695252	100.172139	100.240093
99.8806323	99.8747142	100.304537	100.158488
99.8814622	99.8252111	100.25757	100.179044
99.9335806	99.9743916	100.457761	100.238393
99.8778106	99.8327624	100.144724	100.12665
99.9229578	99.7706738	100.298161	100.237466
100.041137	99.9198543	100.154075	100.240712
100.01292	99.8584369	100.121985	100.141487
100.033834	99.8156461	100.011051	100.194036
100.117821	99.8755532	100.163	100.058336
100.063047	100.024062	100.146849	100.101303
100.101721	100.030943	100.404207	100.065137
100.132427	100.03648	100.37828	99.8974442
100.072176	100.045877	100.515141	99.7824551
100.153507	100.182808	100.364254	99.8224849
100.076657	100.071887	100.418446	99.8934258
100.1633	100.241037	99.9812985	99.9609665

100.21658	100.340547	100.231643	99.9455109
99.9282692	100.073566	100.320475	99.7918829
99.9694328	100.136158	100.300074	99.7023954
100.074831	100.053932	100.035065	99.8207848
99.808762	99.6934826	100.402507	99.9376286
99.8866077	99.8317556	100.222505	99.8716335
100.015576	99.878406	100.024014	99.9343829
100.067196	99.989662	100.115822	99.9883228
99.9128328	99.7881258	99.8639892	99.9948141
100.031012	99.9404946	100.311762	99.8006926
100.354844	100.468583	100.06078	99.9350012
100.076657	100.261845	100.032728	100.144424
100.022547	100.201267	99.8501756	0
100.064707	100.177438	100.233556	0
100.040639	100.201938	99.7830203	0
100.038315	100.115014	99.754118	0
100.024373	100.128271	100.040591	0
99.9176463	100.027754	100.194453	0
99.9847031	99.8933408	99.8907664	0
99.6512449	99.5719903	100.039316	0
100.025203	100.085312	99.9572841	0
100.196662	100.401125	100.006588	0
100.162304	100.284331	100.266496	0
100.369284	100.297588	99.886516	0
100.281977	100.010638	100.344277	0
100.007941	100.020371	99.810435	0
99.7694242	99.9809361	99.7296786	0
99.9918404	100.022049	100.179577	0
100.007277	99.860954	99.9311446	0
99.9667771	99.9112961	99.4608448	0
100.076989	100.216202	99.7188403	0
99.8019567	99.8169886	100.018701	0
99.8291778	99.711438	99.7951338	0
99.8230365	99.6605924	100.338327	0
99.9314229	99.935796	100.255658	0
100.102053	99.8572623	100.25077	0
100.225045	100.115349	100.198703	0
100.146536	100.100918	99.9158433	0
100.077653	100.017686	99.9324197	0
100.118485	100.055778	100.016789	0
100.006779	99.9607992	100.009138	0
99.9732411	99.957341	99.8515302	99.9355249
99.9112762	99.8158225	100.001363	99.7929964
99.979075	100.087618	100.175933	99.7966797
99.8113124	100.077301	100.173922	99.7443125
99.8858911	99.9479475	100.193631	99.8517694

99.8985048	99.8346095	100.108559	99.9561835
99.9112762	99.7181916	99.9967372	99.9292792
100.09591	99.8272179	99.9744131	100.193838
100.105212	99.8687957	100.004179	100.208891
100.034733	100.104249	100.016849	100.000543
99.9035503	100.023557	100.437386	99.9824472
99.9076497	99.9320863	100.031933	99.884599
99.9071767	100.036647	100.27247	99.8584954
100.014078	99.8905085	100.166682	100.023284
100.04104	99.7617713	100.285944	100.076132
100.147468	99.8792671	100.260604	99.8520896
100.170173	99.9655025	100.25638	100.018159
99.6963698	99.5745172	100.236872	99.8456839
99.9191598	99.7588455	100.041385	99.9749204
99.9792326	99.7191156	100.277699	99.9553828
99.8469462	99.6675283	100.014436	100.067804
100.161028	99.8376893	99.9993517	100.206329
99.7709485	99.6869313	100.230436	99.8485665
99.9505364	99.7660831	99.9814522	100.094868
99.9212095	99.672918	99.9490723	99.9814863
99.8910942	99.7245053	99.7670607	100.00759
99.9853818	99.8992861	100.05124	100.113926
99.807686	99.7454482	100.25457	99.8360752
99.9976802	99.9012879	100.342257	99.9047771
100.087868	100.08038	100.263419	100.05259
100.177899	100.428248	99.8796867	100.155243
99.8869948	100.35079	100.14295	99.8376767
99.8354362	100.352792	100.059285	99.7185293
99.9489597	100.374967	100.132894	100.043943
100.008402	100.267019	99.9709941	100.006469
100.070209	100.531115	100.202481	100.1301
100.09654	100.327229	99.8816978	100.031291
100.017862	100.144749	100.024089	100.040259
100.156455	100.314602	99.8330274	100.157325
99.7127677	99.9239248	100.065519	99.784829
99.9115915	100.024789	99.9768265	100.062519
99.8494689	99.9097575	99.8286028	99.9488168
99.949275	100.024635	100.016648	100.017519
100.000045	99.957649	100.070145	99.9960594
99.9402878	99.8832709	100.185587	99.9433719
100.200446	100.249618	99.873452	100.301455
99.9136413	100.070987	99.9518879	100.013996
100.129809	100.24161	99.7063232	100.097431
99.8917249	99.8641759	100.101318	99.9952587
99.9396571	99.8416931	100.18619	99.981166
100.129967	100.128426	99.9858768	100.110242

100.122714	100.001691	99.9510835	100.018319
100.330367	100.05143	99.9563125	100.038338
100.355437	100.230523	99.9822567	100.253091
100.192405	100.206654	99.6475968	99.9742798
100.400373	100.383282	99.7632395	100.242522
99.8499419	100.093316	99.6958651	99.9619487
100.041986	100.186327	99.9615416	100.01928
100.110415	100.227905	99.8332285	100.108641
100.162605	100.398374	99.9170946	100.12962
100.135012	100.187713	99.7051165	99.9979812
99.9114339	100.072527	99.8497201	99.8008435
100.014236	100.060977	100.017251	99.9803653
99.637716	99.8361494	99.8181446	99.7108423
99.8551451	99.9203829	99.9800444	99.7857899
99.6129616	99.8943583	99.8798878	99.7263763
100.034575	100.003847	99.8161335	100.144834
99.8042172	99.8413851	99.724826	99.8849192
99.895982	99.8110487	100.03676	100.030651
99.7834045	99.7574596	100.008402	100.128499
100.004933	99.9618067	100.359352	100.125296
99.9754485	100.052354	100.050838	99.9915754
99.8225071	99.8527805	99.8748598	99.800363
100.124764	100.264247	99.854547	100.206489
99.9426528	99.9209989	100.055866	99.9709168
100.095121	99.9333183	99.8627928	100.112164
99.7331073	99.5463367	100.138324	99.6054673
100.08235	99.8467748	100.100112	99.9483364
100.005248	99.6947849	100.064715	99.8765917
100.011871	99.824446	100.286347	99.9958993
100.182787	100.232524	100.044201	100.089584
99.9886929	100.082382	100.041385	100.136186
100.231034	100.251465	99.7155746	100.280636
99.8622403	99.8416931	100.088246	99.8717874
100.038202	100.05605	99.5989263	100.206649
99.9270433	99.9872154	99.6313063	99.9975007
99.9292507	99.9296224	99.791396	99.8283883
100.123975	100.066213	99.9484689	99.9470553
100.228984	100.067907	100.073966	99.9369662
100.137693	99.7850241	100.039173	99.7329422
100.287796	100.261167	99.7672619	100.011754
100.140373	100.062671	99.6582561	100.069886
99.9899543	99.8316836	99.9359996	100.125136
99.9658306	99.9120674	99.9927148	99.9635501
99.874381	99.844465	99.950279	99.9568241
99.9560549	100.018938	100.032938	99.8067688
100.061064	100.090236	100.160447	99.9949384

99.9565279	100.13166	100.023084	99.9292792
99.895982	99.9998427	100.033743	99.9883725
100.152356	100.229599	100.092067	100.264301
100.038192	100.404743	100.141868	100.134468
100.055707	100.333875	100.137644	99.9796187
100.041821	100.274278	100.136034	100.095716
99.9223694	100.178398	100.219911	100.024776
100.068804	100.466965	100.148908	100.099719
99.8056005	99.9583837	99.9745169	99.7930627
100.071013	100.390847	100.096812	100.026218
100.386447	100.557595	99.4887575	100.032783
100.169004	100.307319	99.9483684	99.8462271
100.142968	100.442879	100.259536	100.047676
100.154961	100.171296	99.9389147	100.007002
100.179261	100.15632	100.154741	100.009083
100.092631	100.23151	100.083536	100.075859
100.107937	100.365681	100.109282	100.080023
100.168373	100.460326	99.9057262	99.9709715
100.008053	100.35827	100.073881	99.8531129
100.153856	100.598665	100.025406	100.029741
99.8676143	100.393163	100.014947	99.8929862
100.1092	100.483331	99.9719021	100.003479
100.002372	100.333412	99.8546359	99.9634452
100.164428	100.340205	99.6814522	100.056483
100.251058	100.199705	99.953397	100.075379
100.279146	99.8918389	100.058595	100.14856
100.194567	99.8402706	100.025205	99.9770566
99.9850144	99.7857688	99.9192027	99.911882
100.10415	99.7795929	99.8492051	100.012126
100.045608	99.7229295	99.9865855	100.001717
100.149753	99.9237989	99.937909	100.025897
100.156223	99.8447481	100.093593	100.019492
100.17863	99.7777402	100.218704	100.04095
100.140601	99.6800074	99.9061285	99.9712917
100.171056	99.8554014	99.7458178	100.017731
100.046713	99.6381661	99.9572187	99.8820971
100.120877	99.8342492	99.9648621	99.8894633
100.04908	99.7172169	100.098823	100.037747
99.8837094	99.520053	100.068652	99.8486291
100.050658	99.8333228	100.025205	99.9065976
100.191569	99.8631212	100.288702	100.012286
100.125295	99.7885479	100.370768	99.9155651
100.234648	99.8235958	100.277035	100.037267
99.7042955	99.4632352	100.363728	99.8918653
100.143915	99.8399618	100.047733	100.106125
100.008368	99.7501033	100.006297	100.068013

100.145966	99.8260662	100.123966	100.141834
100.134605	99.7283334	100.042704	100.039028
100.179419	99.8192727	99.9483684	100.126622
100.177683	99.775733	100.017561	100.2685
100.089318	99.8401162	100.279851	100.126462
100.039927	99.5436756	99.7419961	100.087549
100.051289	99.7442362	100.221721	100.079062
99.9591359	99.6878816	100.079111	99.9884261
100.184626	100.058587	100.092185	100.188754
100.121823	100.090392	100.152528	100.225424
100.232281	100.143968	100.064227	100.229267
100.587164	100.346844	99.7061927	100.111729
100.114249	99.99127	99.801132	99.9514351
100.068015	100.341286	99.7955	100.234552
99.7503719	99.882112	99.8964736	99.8832181
99.9720752	99.887979	100.031038	100.206208
100.112514	100.067851	100.066439	100.102121
99.8545172	99.8137145	100.06483	99.9317387
100.036456	99.9271956	100.006297	100.194038
99.9461966	99.7930254	100.124167	100.111889
99.8895479	99.6911239	99.6506773	100.154005
99.9633964	100.023384	99.839148	99.9796187
99.778144	99.905117	99.9409261	99.8904241
99.7282805	99.8067666	99.9236279	100.003959
99.8261139	99.8682163	99.7174567	100.069614
99.5794789	99.8564822	99.8741468	99.8665641
99.7278071	100.017209	99.7866497	100.096677
99.806705	100.151996	100.184108	99.9800991
99.7667827	100.122507	100.094398	99.907078
99.7035065	100.011805	99.8592622	99.9195685
99.901856	100.155702	99.9537993	99.9144442
99.6703694	99.9611628	100.105058	99.8463873
99.709345	99.9057346	99.6711939	99.974014
99.6261865	100.012577	99.9783387	99.8020302
99.6638997	100.070475	100.011527	99.8470278
99.5618058	100.008717	100.091381	99.7565521
99.9301014	100.320134	99.9829649	99.8934666
99.5163606	99.857563	100.068048	99.7522285
99.9078522	99.9668755	99.834924	100.061928
99.9390958	100.055962	100.051957	99.7964255
99.8482054	99.8958532	99.8099823	99.8065139
99.8617758	100.047316	99.9000942	100.003959
99.9263143	100.14968	100.111294	100.017891
99.8822893	99.8740834	99.9103524	99.7676014
99.984541	100.049323	99.834924	100.041591
100.163008	100.224871	99.9473627	100.096356

99.9561378	100.15246	99.9807524	100.040309
99.8691922	99.9380034	99.7522544	99.9767363
99.9392536	100.091164	99.9688849	100.002358
99.8592511	100.017054	100.01555	99.8820971
99.8119123	100.026627	99.9043182	99.9011531
100.080954	100.285704	100.289707	100.044633
99.9711284	100.231665	100.328327	99.9187678
99.9259988	100.046853	100.068048	99.9294968
99.9102192	99.9696546	99.8836005	100.004279
100.093105	100.009334	100.378814	100.059686
99.861525	99.5060045	100.275232	100.072543
99.905345	99.4595827	99.980941	100.08119
100.064705	99.8028277	100.093186	100.133553
99.9882561	99.5688739	99.9455376	100.00817
99.781136	99.3733487	99.888007	100.038275
100.009063	99.6182163	100.137641	100.191201
99.9868374	99.7135194	100.162786	100.058131
99.8068289	99.5144588	100.00347	100.078948
99.9136991	99.4952445	100.045713	100.086475
99.9307227	99.5590361	100.083128	100.060533
99.8218034	99.3229303	100.118733	99.9916765
99.7838156	99.4374478	99.9028925	100.029948
99.6849843	99.4343735	100.054564	100.016817
99.7689988	99.3630498	100.171033	100.00865
99.8925773	99.3298474	99.8968578	99.9859118
100.08425	99.6403512	100.211265	99.9583691
100.10348	99.8718456	100.251496	99.9596501
99.8654657	99.8727679	100.070858	99.9791862
100.070379	100.222623	99.9006798	100.072383
99.8605793	100.207251	99.915163	99.9079275
99.8887943	100.14346	99.7003285	99.9734215
99.8525404	100.245372	100.015741	99.9104896
99.9515293	100.341752	99.9604231	99.9482808
99.9097585	100.06691	99.9612277	99.9742221
100.003388	100.189728	99.8176024	100.036033
100.034913	100.211401	99.5208974	99.9812679
99.953736	100.04831	100.16178	99.9119308
99.8903705	100.001273	100.061001	99.8965581
100.084881	100.293946	99.7765666	100.158534
99.8571115	100.079822	100.065829	99.9224995
99.9999204	99.8833742	99.9216	99.9798267
99.9810053	100.02141	100.223133	99.7308216
99.9097585	99.9424007	100.001861	99.967977
100.03649	100.085355	99.9348763	99.9143328
99.9368701	99.8655433	99.9722913	99.8341067
99.9540513	100.016799	100.088962	99.8331459

99.9609868	100.09919	100.125773	99.9652547
100.160383	100.106414	99.8962544	100.076226
100.143833	100.230923	99.9312555	100.114338
99.997556	100.013417	99.8387238	100.031229
100.070379	100.020795	100.198994	99.9367512
99.8960451	100.167285	99.9346751	99.97166
99.9578343	99.9337926	100.048731	100.006249
99.9737545	100.022025	99.8936393	100.004967
99.9633512	99.9769865	99.9093295	99.9036039
100.028608	99.9711453	100.005482	100.001284
99.892262	99.9553127	99.8043261	99.8451558
100.004807	99.9891299	100.17385	99.94732
99.8340982	99.8826056	100.22273	99.8088058
100.101274	99.9048943	99.7735492	99.9551664
100.080309	100.15299	99.9264277	99.887911
100.079837	100.171436	99.868696	99.9756633
100.072586	100.037396	100.077697	99.8225772
99.8301575	99.9539293	100.087554	99.8971987
100.203573	100.176816	99.8948463	100.100566
99.9140144	100.109335	99.9861711	99.8421133
99.8886366	100.017875	99.7347261	100.018899
99.9896747	100.357277	99.8526035	100.015216
100.001339	99.9356372	100.076088	99.9867124
100.074162	100.028173	100.089364	99.8925548
100.145882	100.176201	99.9604231	100.039236
100.245028	100.258284	99.9881826	100.136756
100.213818	100.300095	99.7522267	100.120102
100.253225	100.399702	99.8990705	100.162537
99.997556	100.133929	100.037466	100.083112
100.155655	100.223237	99.9028925	100.116579
100.184185	100.202793	100.279658	100.081991
99.9196889	99.9736048	99.9608254	99.9165746
100.251649	100.308703	99.9036971	100.09304
99.827005	99.8767645	100.110687	99.9351499
100.170156	100.069676	99.9008809	100.276391
100.075896	100.030172	99.8324879	99.992317
99.9406531	99.7640916	99.8336949	100.025304
99.984473	99.973451	100.102842	100.052046
99.7206077	99.7205903	100.221926	99.8044823
100.066754	99.9310258	100.027207	100.17759
99.9652427	99.9826739	100.394518	100.002085
99.9406531	99.8355689	99.9447329	100.008811
99.9931425	100.194493	100.123762	99.9164145
99.8689334	99.9010514	100.080513	99.9878333
100.058084	99.9480881	99.872518	100.114338
100.070852	100.087046	100.109882	100.109213

99.9501107	99.8922897	99.7069666	100.000324
100.010008	100.125475	100.252099	99.8994405
100.030342	100.133468	100.027609	99.8228974
100.211296	100.304091	99.8954497	100.078308
100.044056	100.107798	100.184109	100.067739
100.362775	100.302401	99.7954752	100.28728
100.092762	100.1948	99.8061365	99.9468396
100.074793	100.198643	100.103847	100.051886
99.8091933	99.9502401	100.170229	99.9468396
100.013949	99.9928191	99.7642961	100.022582
100.041218	100.05615	100.077898	99.9164145
100.111992	100.035552	100.093186	100.145563
100.179614	100.170206	100.108273	100.32379
99.9043992	99.8580113	99.9584115	99.984951
100.17788	99.9471658	100.011718	100.205132
100.03712	100.089813	99.9698774	99.9426761
100.063916	99.9843648	100.110485	100.144282
100.055877	99.9786773	100.092784	100.03235
99.8405608	99.750411	100.306814	99.8533225
100.257503	100.257196	99.9698405	100.205307
100.096919	100.308865	100.148586	100.122334
100.272173	100.299765	99.9792905	100.415942
100.20103	100.388142	100.262186	100.316311
100.226269	100.430866	99.8290963	100.237983
100.135882	100.482072	99.6841297	100.167504
99.9888639	100.212621	100.038202	100.156132
100.054012	100.212313	100.017694	100.139794
99.9535291	100.391227	100.031567	100.004442
99.9749824	100.122393	100.042424	100.064509
100.107172	99.9524246	99.8341228	100.175513
100.093449	99.8455388	100.018699	100.269538
100.106857	99.9113978	99.8055719	100.169907
100.052435	99.7937154	99.8182388	100.216519
100.012526	99.7303242	100.12486	100.187046
100.155442	99.9366925	99.9241992	100.229974
100.116637	99.877003	100.0189	100.101831
100.281953	100.033553	99.9193736	100.102952
100.342054	100.049902	99.9891426	100.140114
100.024356	99.9345332	100.034784	100.056501
100.057167	100.157096	99.847393	100.197778
100.04928	100.180386	100.105156	100.113684
99.9010002	100.030314	100.113802	100.039201
99.8054071	99.7289361	100.187391	99.8626842
100.031139	100.174062	100.124458	100.05602
100.088874	100.222184	100.189804	100.103753
99.9292365	99.8109899	99.9328449	100.027188

100.085246	99.9703161	99.8998705	99.9965936
99.7852158	99.7698088	100.071579	99.873256
99.8110859	99.7087312	99.8723249	100.030872
99.9358618	99.8102187	100.011863	100.006044
99.8151872	100.071187	100.197444	99.8577186
99.7628161	100.107895	100.351861	99.7656158
99.7695991	100.005328	100.20991	99.8189553
100.176107	100.378117	99.9628033	100.051054
99.9131466	100.285884	99.8544302	99.7423899
99.6957747	100.117766	100.126871	99.7662565
99.8948482	99.9653805	100.054287	99.9165042
100.081618	100.241001	100.415196	99.9354053
99.9820809	100.192416	99.9989947	99.8495495
100.106384	100.234677	99.7671688	99.8780613
100.061426	100.049594	99.8562398	99.8718143
100.005112	100.254728	100.001407	99.9004864
99.885699	99.976177	99.875944	99.7715424
99.8973721	99.9474891	100.034985	99.833852
99.8827019	100.329378	100.01126	99.8295271
99.6806312	99.9843516	99.6969977	99.6770369
99.9469039	100.163728	99.9624012	100.005884
99.8006747	100.201516	100.111992	99.7033062
99.9084142	99.9618331	99.9376704	99.8626842
99.9754556	100.161106	100.053684	99.7736247
99.9484813	100.085376	100.022921	99.9024085
100.049596	100.181466	100.277668	99.9246734
100.152445	100.255499	99.9953755	99.9462975
99.764709	99.8831725	99.9024843	99.7042673
99.9603122	100.089078	100.053081	99.9168246
99.9117269	99.9828092	100.129485	99.9096165
99.9399631	100.086302	100.277467	100.065791
99.9052593	99.9172587	100.001407	100.022062
100.096603	100.214318	100.167687	100.023023
99.9316027	99.9692364	99.8672983	99.9334832
99.7367882	99.8543303	99.8502079	100.047691
99.84437	99.772585	100.02071	99.9419727
99.8916933	99.9607534	99.8723249	99.8952005
99.9087297	99.8982877	100.091685	99.9339637
99.8435812	99.9135571	100.038403	99.9124998
100.024672	100.001318	99.8196463	100.080527
99.8173957	99.7310954	99.8286941	99.9623154
100.137617	99.9973074	100.025334	100.016776
99.9525827	99.8711421	100.044837	99.8112667
99.8976876	99.7829189	99.9519459	100.087735
99.9377547	99.9368468	100.277266	100.018698
100.091398	100.035867	100.134712	100.043846

99.8571473	99.7347971	100.027948	99.8493893
100.014576	99.7747443	99.822059	100.061306
100.116479	100.046972	99.949131	100.058583
100.165695	100.039568	99.9563693	100.098147
100.519832	100.389993	99.8170325	100.195856
100.535449	100.486545	100.0945	100.058102
100.265548	100.217865	99.996783	99.9751297
100.293626	99.9857397	100.048456	100.058423
100.168219	100.071187	100.033175	100.005243
100.244252	100.164191	100.224185	100.160296
99.9098339	99.8927352	100.390465	99.904651
100.116637	99.900447	100.080626	100.137231
100.172794	99.9578229	100.041419	100.177756
100.206709	99.9295977	100.035588	100.19009
99.9536869	99.78369	99.9885394	99.9903467
99.9639403	99.815617	99.8672983	100.154049
100.142034	100.136274	100.029154	100.023984
100.16538	100.250872	99.9660203	100.210112
100.017889	100.115915	99.8481973	100.089337
99.8980031	99.8876454	99.6732723	100.126018
99.7956269	99.6863669	99.6831244	100.007326
99.9355463	99.7832273	99.7643539	100.175994
99.9909146	99.6973177	99.7595284	100.036638
99.8265448	99.7665698	100.197645	99.9733677
99.9729317	99.7061091	99.7211253	99.9978751
100.000222	99.657062	99.917363	99.9975547
100.019782	99.5043679	99.9601895	100.053137
100.734894	100.459649	100.067401	100.562999
100.57476	100.301749	100.325048	100.470835
100.67514	100.501511	100.30072	100.495586
100.477596	100.450836	99.9392733	100.556974
100.616232	100.446062	100.018977	100.363365
100.718813	100.304687	100.162165	100.437454
100.684111	100.382719	100.454103	100.32347
100.664137	100.891855	100.016197	100.449341
100.448312	100.546311	100.558135	100.06782
100.634345	100.992471	100.069487	100.481745
100.674463	100.882491	100.022684	99.9815183
100.554108	100.893508	99.7794027	100.351803
100.458807	100.663267	99.6255563	100.243681
100.539889	100.529419	99.773842	100.499331
100.556647	100.466075	99.7186983	100.462856
100.676325	100.753784	99.3642026	100.824511
100.392282	100.176714	99.6939067	100.346593
100.535827	100.428804	99.6433969	100.768333
100.599982	100.322313	99.3491423	100.524896

100.872006	100.273657	99.675371	100.727136
100.874038	100.363991	99.7909876	100.485653
101.012166	100.738912	99.3037298	100.892739
100.967985	100.352975	99.7604036	100.708247
100.891134	100.431925	99.6225442	100.685939
100.797525	100.435781	99.4142491	100.4539
100.901799	100.818413	99.8396438	100.946962
100.466085	100.333513	99.7705983	100.341545
100.478273	100.170839	99.7988653	100.18441
100.391605	99.9820927	100.403825	99.7061656
100.281237	100.385656	100.744187	99.9014039
100.282592	100.306339	100.759016	99.4127384
100.642131	100.704395	100.540526	99.7931191
100.187628	100.325618	100.319256	99.6143271
100.341499	100.798217	100.165409	99.7525734
100.33439	100.614428	99.8873733	99.7771614
100.387711	100.507754	100.447384	99.7528991
100.760454	101.054897	99.9951122	99.8966817
100.716273	100.6741	99.2161486	99.6727846
100.263294	100.0772	99.8820443	99.5871338
100.087249	99.9211358	100.084315	99.6491737
100.182042	100.25089	100.233064	99.4179491
99.7993122	100.282654	99.8581796	99.6719704
100.196092	100.714493	100.141776	100.31598
99.5538638	99.9741976	100.646179	99.6703421
99.4145507	100.094643	100.104241	100.155914
99.4969875	100.021751	100.277319	99.8831664
99.5095138	99.7408357	100.360729	99.4259279
99.7312637	99.8816607	100.503918	99.7006293
99.3316061	99.5298736	100.614437	99.3705643
99.5486163	99.6350792	100.378802	99.5674309
99.0448546	99.212237	100.518746	99.4242996
99.440619	99.5858731	100.208041	99.5721531
99.5452308	99.8693592	100.454335	99.8460402
99.6877601	99.904795	100.103314	99.8608582
100.057118	100.039561	100.066011	100.157379
99.4177669	99.4481694	100.080377	99.9976389
99.7512382	99.6528889	99.727271	100.3321
99.4756589	99.5678798	100.060914	100.114228
99.6693092	99.6978721	99.8382537	100.115857
99.6557672	99.8207039	99.8836662	100.12872
100.090465	100.34508	100.093352	100.527501
99.4108266	99.5243654	99.5620714	100.014411
99.1135802	99.3974944	99.9939537	100.167312
99.2777767	99.5667782	99.8500702	100.111297
99.4682108	99.7252293	99.6445554	100.203461

99.4131965	99.5348309	99.9886247	100.408632
99.541676	99.7511176	99.4985866	100.48028
99.9325314	100.037358	99.8699961	100.467253
99.5692678	99.7703961	99.8408023	100.216325
99.8011742	99.8298841	99.7979385	100.386813
100.051024	99.8948803	99.991405	100.460577
99.9017234	99.7164162	99.7476603	100.244496
100.001257	99.8495299	99.9955756	100.115368
99.8746394	99.6965869	100.105168	100.067983
100.040867	99.8273137	99.9649916	100.163893
99.2804851	99.2320663	100.148727	99.8462031
99.8616053	99.7140294	99.7747688	99.7361271
100.003627	100.011469	99.543304	100.141422
99.3874667	99.6460955	100.095437	100.248241
99.4148892	99.4228319	99.8060478	99.9054747
100.01446	99.7990385	99.6802366	99.9999186
99.757332	99.6604167	99.6966871	100.17578
99.7925412	99.7619503	99.7119791	100.100387
99.7613946	99.6181876	100.023843	100.008874
99.8463706	99.8686248	99.8528505	100.140119
99.8199637	99.3214819	99.8662889	99.1968202
99.1733342	99.2739282	100.247198	99.3995488
99.3610599	99.1745979	100.100302	99.4321156
99.8666835	99.5592504	100.385057	99.277586
99.2904723	99.1747816	100.116985	99.1935635
99.6102323	99.2864134	100.271758	99.5719902
99.9096793	99.6438923	100.477273	99.3780547
99.5667287	99.4586349	100.53844	99.4295103
100.198801	99.7479963	100.536355	99.2741664
100.048485	99.5893616	100.289367	99.0676926
99.6484884	99.8225399	100.129496	99.5588007
99.9029083	99.5956042	99.7849635	99.3918956
99.6269905	99.6451775	100.023147	99.7750445
99.5423531	99.7186195	99.8180961	99.8181956
99.9599539	99.6587643	99.5873263	99.8991242
100.269589	100.458781	99.7058429	100.491161
100.340316	100.158876	100.015178	99.9882572
100.503531	100.257303	99.9994684	100.202548
99.9989232	100.229921	99.8657082	99.9596087
100.28064	100.360354	99.5686174	99.9704133
99.9319369	100.140375	99.6534015	99.8623676
100.203963	100.238061	99.5600697	100.159493
100.127455	100.337968	99.8846518	100.006592
100.403052	100.093382	99.9800628	99.8612217
100.460857	100.094307	100.087949	99.951096
100.535324	100.519835	99.7483504	100.128225

100.217224	100.533711	99.9491062	100.380005
100.239666	99.9832991	100.023263	99.6669032
99.8154758	100.110773	99.8426063	99.9029666
100.412913	100.223815	99.5810925	99.879393
99.9741009	100.295785	99.6961401	99.8581113
100.334365	99.8097575	100.216858	99.737624
99.8979336	99.5450047	100.47583	99.4292027
100.008104	99.4985666	100.426161	99.5066354
99.4946553	99.7031904	100.253128	99.2124565
99.5602815	100.14093	99.9775216	99.7148689
99.9093247	100.583294	100.134846	99.9948054
100.081381	100.187923	99.6326098	100.195508
100.570518	100.013641	99.9574229	99.6912953
100.001473	99.9076291	100.074319	99.7341861
100.248507	100.051569	99.9546507	100.094338
100.356637	99.8987485	99.5422812	99.9903854
100.38486	99.6206748	100.244811	99.7472826
100.129156	99.6721083	99.8936616	100.112837
100.048568	99.653607	99.3292815	100.127243
99.6243776	99.4824705	99.9904587	100.139849
99.1044681	98.6258631	100.24019	99.669195
99.775692	99.1997716	99.9682808	100.041953
99.4164478	99.3159594	99.9544196	99.8834856
100.397951	100.070995	99.0871733	100.386225
100.085291	99.6956048	99.3777955	100.389008
100.421923	100.093382	99.4771338	100.47168
100.17897	100.001985	99.5554493	100.417493
100.370749	100.359984	99.3186546	100.425024
99.7530798	100.166461	99.3052555	100.374602
99.9574393	100.170162	99.4055178	100.44958
99.9339771	99.1873757	100.098345	99.5120377
99.6888137	99.0410309	100.406293	99.1944489
99.5174374	98.952595	100.250355	99.4192166
100.0654	99.3914444	99.8825727	99.3959704
99.6713021	99.0317803	100.021646	99.350624
100.289481	99.3487066	99.9844522	99.2949641
99.8309473	99.1091155	100.074319	99.5388854
99.765151	99.2103173	100.363324	99.5131836
100.450656	99.2693362	99.9243871	99.437879
100.100083	99.1379774	100.024418	99.3408016
100.17744	99.1649892	99.7890098	99.3558626
99.9089847	99.3357557	100.109665	99.3324527
100.270609	99.6574923	99.8654772	99.5112191
99.923436	99.4613791	99.9544196	99.3859844
100.264488	99.7816356	99.9666637	99.4607978
99.8989537	99.4832106	99.4489495	99.569662

99.9958629	99.4846907	99.6931368	99.5356113
99.7280875	99.5392694	99.0231809	99.7895186
101.183596	101.618439	100.0362	102.180766
99.1961069	98.9407542	100.347845	99.6626468
99.7357382	99.7494435	99.6582529	100.300444
99.7180565	99.7146611	100.380881	100.34579
99.5390295	99.7881111	100.571703	100.085335
99.5682723	99.8082774	100.420617	100.169643
99.7629408	100.07044	100.573551	100.071911
99.5390295	100.075435	100.346921	100.325163
99.5070664	100.153141	100.111051	100.35905
99.6270979	100.22104	100.040128	100.24331
99.8103753	100.357579	100.409528	100.295369
100.087672	100.74111	100.064616	100.620161
100.361398	100.763497	100.445567	100.322217
100.393361	100.695597	100.169499	100.285219
100.27945	100.658409	100.047058	100.49067
100.013715	100.660815	100.79094	100.363634
100.242726	100.980701	100.094648	100.570885
100.224535	101.013818	100.24227	100.547148
100.293901	100.801054	100.698071	100.321398
100.343376	100.922422	100.476061	100.272123
100.310563	100.868954	99.7809242	100.26672
100.17251	100.5968	100.409297	100.122332
100.27809	100.806604	100.231412	100.019689
100.257348	100.939258	100.413455	100.092047
100.037517	100.572009	100.277847	99.8934717
99.9955229	100.482648	100.737575	99.7765859
99.3890752	99.4373275	101.395056	98.8950313
99.4756134	99.5988434	100.399594	99.551327
99.4038666	99.6006935	99.4295438	100.031639
99.5167574	99.8103125	99.8654772	100.028692
99.9669602	100.296155	99.3946599	100.559426
99.8345176	100.275804	99.5177932	100.441231
100.084951	100.548882	99.7407268	100.524066
99.8545795	100.307996	100.005475	100.336623
100.096513	100.618262	99.6210588	100.604445
99.9800514	100.572749	100.088411	100.49378
100.031566	100.583849	100.035738	100.320743
99.9956929	100.381816	99.7238624	100.34579
100.020515	100.186443	100.109434	100.045554
99.8870526	100.29153	99.8696356	100.148034
99.9043519	99.8115439	99.6726149	100.004448
99.9242142	99.7926073	99.7169373	99.9232323
99.7865364	99.5912913	100.098987	99.5122502
100.214	100.037312	99.5941275	100.096613

100.131665	99.7584111	99.9570166	99.7232156
100.136927	100.013779	99.9424733	99.8647307
99.9715781	99.7545503	99.7975023	99.893001
99.8649669	99.621075	99.8224337	99.7696247
99.4665331	99.5260245	99.8695261	99.6684724
99.8501975	100.077391	99.4699326	100.281269
99.9046915	99.9455707	99.642605	100.079291
100.132513	100.073714	99.8162008	100.151683
100.109086	100.230538	99.535031	100.278491
100.097203	100.10644	100.105219	100.171129
100.246424	100.039886	99.8856853	100.118674
100.124704	99.8016159	100.180475	99.9951338
99.7255915	99.6650152	100.818301	99.5522862
99.7968919	99.6227297	100.560447	99.4390415
99.5964018	99.2838938	100.780673	99.2882118
99.8984102	99.5721709	100.297514	99.4591412
99.6651558	99.4956892	99.9824096	99.3775984
99.6362961	99.6102279	100.013574	99.5823541
99.5040507	99.4534036	100.075902	99.4142028
99.937286	99.7799216	99.7762646	99.816524
99.8383141	99.631003	99.5488818	99.7585126
99.9068984	99.8589772	99.2771766	99.9395735
99.7603929	99.500837	99.9106166	99.6586677
99.9169144	99.3905269	99.7104736	99.6465752
100.028279	99.5604045	99.5070987	99.7643955
100.093468	99.4315255	99.7146288	99.5797395
100.810038	100.243776	99.8074287	100.328985
100.209416	99.6139049	98.9678437	99.5867662
99.5393614	99.2531908	100.101757	99.9224152
99.6581956	99.4614931	100.156236	99.9418613
99.7026735	99.3282017	99.6063623	99.7921756
100.224355	99.6648314	99.4263028	99.8312311
99.5410591	98.894499	99.6917751	99.6341558
99.4919975	98.9891819	99.9701748	99.4955821
100.044576	99.6355992	99.9166186	99.9678439
99.6230546	99.237012	99.7878068	99.7807367
99.5296849	99.2601771	100.424017	99.9189836
99.2619687	99.1763414	100.196403	99.9632683
99.5556587	99.7791862	100.229183	99.7424982
99.4960719	99.7416808	100.370923	99.8094973
99.5996273	99.8720306	100.216487	100.043667
99.663628	99.9845469	100.390775	100.099718
99.6841693	99.887474	100.186939	100.121615
99.536815	99.781944	100.376463	99.9952972
99.9075774	100.156447	100.206099	100.317546
99.6955434	99.9290241	99.9872573	100.102496

99.7474909	100.045953	99.9951061	100.139754
100.023526	100.208844	100.12184	100.313624
100.136418	100.263632	100.35107	100.09694
100.361524	100.460535	99.9505529	100.193516
100.097203	100.005138	99.9048455	99.8379311
99.8359374	100.005322	100.092292	100.244338
100.074285	100.414573	100.016113	100.067525
100.121988	100.281649	100.104758	99.942515
100.154583	100.145232	99.9874882	99.8683258
100.086847	99.9904301	99.7483323	99.9296054
99.8766806	100.083826	99.875759	100.287478
100.531626	100.270434	99.3053398	99.7915219
99.9301559	99.9700227	100.118147	99.7088353
99.7865364	99.8222072	99.739791	99.9287883
100.282244	100.170419	99.7938088	100.103313
100.100089	99.7992259	100.106835	99.9188202
100.56507	100.307204	100.153928	99.9273176
100.115028	100.109381	100.348531	99.7349813
100.51482	100.311433	99.9064614	99.8253483
100.133702	99.9667134	100.012419	99.8655477
100.22062	100.326324	99.8649092	100.094979
100.516008	100.220794	100.114684	99.7671735
100.707331	100.545842	100.083751	100.046445
99.8726063	99.8712952	99.7090885	99.8879352
100.116725	100.1456	99.6908517	100.155114
99.8743039	100.038231	100.181168	99.9497051
100.699182	100.309778	99.8388237	99.6601384
100.200249	100.127582	100.226875	99.8830329
99.7812737	99.8067638	100.243958	99.8678355
100.08515	100.197813	100.137076	99.9248664
99.9613923	100.176119	100.036197	99.7456031
100.228769	100.613315	100.127381	100.116876
99.97022	100.418617	99.9994921	99.8740452
100.104842	100.553196	100.562755	100.197602
100.197363	100.42781	100.068515	100.169168
100.320102	100.404828	100.106374	100.204302
100.402946	100.870153	100.227337	100.350065
100.208567	100.665712	99.84875	100.477527
100.396155	100.878978	100.099448	100.734902
100.24965	100.66994	100.130612	100.537173
99.9647876	100.282936	100.518202	100.446152
100.169352	100.729876	100.025809	100.592896
100.286319	100.760579	99.689005	100.634893
100.303125	100.905085	100.38662	100.8622
100.339455	100.860961	99.9990304	100.854029
100.288016	100.718293	100.363305	100.748628

100.392081	100.583163	99.9870265	100.330129
100.535361	100.517345	100.060666	100.237474
99.4049091	99.4096473	100.330755	100.001343
100.403285	100.789995	99.8060436	100.749935
99.4763794	99.6175819	99.7866526	100.091874
98.7284226	99.4812934	100.568943	99.5313592
99.0067262	99.9806381	100.453304	99.9516335
99.6052905	100.382837	100.505446	99.8310897
99.3743577	100.307954	100.520046	100.15444
99.7265936	100.591112	100.019713	100.145644
100.246827	100.715856	100.153429	100.058168
100.016063	100.560938	100.525144	99.9190541
100.229232	100.554314	100.846339	99.7629988
99.6848058	100.317705	101.065105	99.6999576
99.973937	100.577865	101.190941	99.9359953
99.5662095	100.041538	100.877161	99.41163
99.8396069	100.148988	100.898945	100.23931
100.003544	99.7140388	100.835447	99.8908729
100.052776	99.9852379	100.439631	99.8481939
99.8262416	99.3324467	100.791416	99.6950707
99.9979608	99.3780758	100.403711	100.108015
99.6007226	98.9468068	100.720735	99.5945633
99.7020623	99.2831377	100.170577	99.9636878
99.3484729	99.167409	99.8869243	99.8278318
99.5643485	99.5370419	100.125851	100.048394
99.5663787	99.5162512	99.8489185	99.9684118
100.10776	100.094343	99.5240152	100.485121
99.8871469	99.646515	100.331175	99.9881224
99.9769823	99.3707163	100.165016	99.6986544
99.3936444	99.0722869	100.362692	99.585441
99.7646595	99.7510205	100.688059	99.9387646
99.529328	99.6104533	100.112642	99.6854598
99.967339	99.9872617	100.076026	99.9749277
99.730654	99.7730991	100.348556	99.6572786
99.7902059	100.063065	99.9895864	99.9250813
99.5316965	99.5938943	100.18819	99.7913428
99.7682123	99.6489069	100.407187	99.8095873
99.4450756	99.503004	100.089931	99.6351246
99.6687336	99.8525822	100.242418	100.026403
99.8008643	99.7182705	100.312868	99.946095
99.8683678	100.036203	100.266751	99.9680861
99.7516325	100.002533	100.049839	99.9346922
99.7709192	100.135004	100.450754	100.102639
99.6091817	100.026635	100.0605	99.9988736
99.9093097	100.297098	99.6760384	100.161119
99.8819023	100.259933	99.9100986	100.150042

99.9015273	100.256437	99.890864	100.061589
100.028075	100.403444	99.4697874	100.300396
99.9889942	100.221479	100.096188	99.9907287
100.077307	100.228471	99.8924862	100.090585
100.091857	99.9134823	100.059341	99.8744203
100.271527	100.166099	100.41159	100.187834
99.8698904	99.6873605	100.165943	99.7576232
100.258162	100.262877	99.7393042	100.488868
99.9624327	99.8987633	99.8887783	99.9296424
100.349689	100.33776	99.568278	100.244034
100.166635	99.8525822	99.6841494	100.06501
100.221111	100.088455	99.6911017	100.355618
99.8903614	99.6271962	99.9147335	99.689858
99.9842571	100.095447	99.879972	100.125119
99.8487427	99.7409011	99.8572613	99.7643019
100.010142	100.032523	99.7729069	99.9995252
99.9429768	99.8339993	99.9411521	99.8172434
100.468624	100.229207	100.05656	100.158187
100.136013	99.7661076	99.7117268	99.7074509
100.238537	100.018724	99.6046616	100.045462
100.035519	99.8785245	99.9425426	100.005389
100.69059	100.137396	99.5263326	100.52047
100.255117	99.8735568	99.9420791	100.142875
100.429543	100.483663	99.8848386	100.35741
99.9274121	99.8205682	100.05262	99.9252441
99.9121858	100.107406	99.9221492	100.202169
99.5663787	99.7648196	100.002564	99.6517401
99.8451899	100.184313	99.8090588	99.9420225
99.8265799	100.084776	99.880899	99.9793259
99.7759946	100.322305	100.428275	99.9482126
99.726932	100.202896	99.8264395	99.7913428
100.006589	100.440609	99.9008289	100.108177
99.917938	100.167386	99.7738339	99.8822393
100.168496	100.379709	99.770126	100.1072
100.041948	99.8925077	99.6586577	99.85585
100.31027	100.152851	99.7615515	100.337699
99.990686	99.9692309	99.8021065	100.034385
100.224157	100.397924	99.6222741	100.363763
100.233123	100.139052	99.7541357	100.336559
100.3639	100.438402	99.7450978	100.136359
100.454413	100.491942	99.735828	100.090096
100.262561	100.240798	99.9172826	99.8607369
100.329049	100.286795	99.8516994	99.9457692
100.201994	100.072816	99.88901	99.8589451
100.290476	100.015412	99.7919098	100.088304
100.036703	99.6185488	99.6387278	99.7649535

100.400444	100.30041	99.1444205	100.334767
100.156146	100.031419	99.7726751	100.097426
100.43022	100.342728	99.1940135	100.33884
100.379296	100.133717	99.4285371	100.290459
100.521747	100.226631	99.6920287	100.416541
100.236845	99.6650979	99.8915592	99.9288279
100.470146	100.224239	99.5140502	100.304957
100.03112	99.8027213	99.7481104	99.8788185
100.249534	100.364806	99.4931934	100.191092
99.9248744	100.07208	99.6435944	99.8472165
100.123663	100.295994	100.007894	99.9245926
100.178478	100.346407	99.7587706	100.064847
100.240906	100.463608	99.704311	100.114042
99.7714709	99.8222696	101.044714	99.8678394
99.6947528	99.4029574	100.554396	98.9539475
99.931231	99.7846997	100.496268	100.012618
99.1437471	99.1293509	100.193646	100.969876
100.090825	99.8003811	100.002844	100.88635
100.188345	100.041972	101.075553	99.678514
99.822228	99.8689869	101.211333	99.4437976
100.377394	100.488889	100.605425	100.515798
99.6446614	99.7260581	101.102398	100.436828
100.031247	100.213649	100.590338	100.364777
100.22712	99.8996962	101.555887	99.7492157
100.439135	100.10045	100.362041	100.751865
101.417998	101.069262	101.339792	99.4446413
101.547137	101.079553	100.715469	99.394357
100.649153	100.676085	100.215611	100.875719
99.3161548	98.6553174	100.568152	100.917735
101.274713	100.68948	99.2544995	101.133215
100.175031	100.400355	100.382452	99.5826701
100.432644	100.3801	100.064966	99.5404852
99.2447621	99.5040693	99.5211791	100.679475
99.8671605	99.6445479	99.6722679	100.472601
100.257075	100.489706	99.9935258	99.2480601
100.147573	100.162522	99.4734785	100.073701
98.9620201	99.0421235	100.198306	101.256564
100.277378	99.9949275	100.013715	101.165613
100.799593	100.875532	100.607643	99.4157869
99.5509688	100.254323	99.4481861	100.098337
99.5203481	99.740596	100.718797	100.481207
100.420662	100.256773	99.6554063	99.8494469
99.9768291	99.7030262	100.157483	99.2485663
100.169373	99.5823126	100.349616	100.738028
100.18718	100.165789	99.8255754	100.342672
99.5449778	99.4153718	100.4215	98.7516291

99.085002	99.2183751	100.130859	100.45404
98.6070533	98.8299356	99.7998393	100.743596
99.83421	99.988067	100.367809	99.5688334
99.8205638	99.5063562	100.374465	99.7903881
99.3943707	99.3384353	100.150383	100.370514
100.328135	100.013549	99.5644424	99.6861072
99.5775955	99.3849892	100.329649	100.712211
100.71988	100.873082	100.584348	99.6791889
99.3076676	99.3119731	100.638038	99.4912134
99.5000453	99.3972403	100.51912	100.300487
100.085832	99.4923083	100.331424	99.9400599
100.894451	100.303654	99.6523002	100.407805
101.191671	100.676575	99.6951198	99.07139
100.375397	100.319008	98.9356821	99.980726
100.116786	100.30202	100.031908	99.7858322
99.89412	99.7819228	100.333642	99.6783452
100.200327	100.928293	100.471197	99.7120931
99.1407516	99.7020461	100.328983	99.6852635
100.604054	100.643579	98.3577284	100.646571
99.2605717	99.3479094	99.7587946	100.405949
101.159386	101.408534	101.319159	99.6602901
99.4126765	99.4256627	99.7445953	99.0578909
99.89412	99.8235764	99.4756971	100.380807
99.5241757	99.7461498	99.6458662	100.82746
100.641497	100.569256	100.75496	100.167183
99.4006945	99.4344834	100.064966	99.6985939
98.9482075	99.2497378	100.690842	99.9704329
98.9485403	99.2998854	99.7306179	100.020042
100.775296	100.712838	99.0197682	99.6192865
100.063699	99.5032526	99.3505663	99.5636025
99.1986646	99.1082791	99.8402184	100.809742
99.1114623	98.8590114	100.390661	100.530479
100.402523	100.954429	101.467807	98.4654472
99.1622194	99.8925089	99.5185167	100.083657
98.7716394	98.9679639	101.686786	100.777007
100.378725	100.933847	100.443908	99.4181492
100.382719	100.665141	100.200524	98.0680662
101.253911	101.818209	97.3549067	100.970044
100.131264	99.9395529	99.4803562	99.5480785
99.7167198	99.4228858	99.9207547	99.5968442
100.189343	100.140633	99.685136	98.8554037
99.8972819	99.8150823	100.848587	99.854509
100.362583	100.209076	99.4319901	100.541784
99.1948371	99.0787133	99.5613363	100.337441
99.7779612	99.8882619	99.7430423	100.25054
100.044061	99.7391259	99.4359836	99.3754582

100.078177	99.7984209	100.811314	99.8804949
99.4263227	98.9813584	100.552177	99.7272796
100.79926	100.661221	99.4925587	98.3442924
99.6656299	99.8067516	99.7978425	99.8671645
99.2031579	99.1995902	100.80488	100.959583
100.200826	99.9522939	98.8740041	100.421811
101.888291	101.999524	99.4881215	98.4484046
100.704237	100.759392	100.218051	99.1670652
99.1023094	99.2022038	99.3241645	100.787975
98.4356441	98.2005589	99.177513	100.105593
101.845689	101.71432	98.2434689	100.821216
99.8025908	100.319825	99.5047612	99.6329544
99.682438	99.6909385	99.9728926	99.9484968
99.1432479	99.4689497	99.4630509	100.707992
99.4674276	99.4939418	99.1397962	100.699218
99.7731351	99.5532368	99.4364273	100.682006
101.832375	101.933695	99.9107709	99.2077314
99.6746164	100.088199	100.92158	99.2733709
99.4040229	99.7419028	99.6041559	100.703099
100.380889	100.462754	97.9419568	101.302967
100.526503	100.926006	100.13219	98.7796398
100.06222	101.031854	99.8220334	99.8869378
100.729844	101.414047	98.3551244	100.750987
99.9972657	99.9656049	100.854936	99.6348813
100.112443	100.551349	101.12494	99.9600392
99.5948164	100.009647	100.424708	100.557513
100.138391	100.087009	101.360499	101.084079
100.360374	100.608752	101.198274	101.145532
99.6083764	100.304086	100.886048	99.7208135
100.838157	101.43747	98.898243	99.8300436
100.048827	100.141609	100.035147	99.638089
101.776145	102.122515	99.1515798	100.338715
100.542347	101.323818	99.5895858	99.3223854
101.565713	101.525555	100.118037	98.6563689
100.181247	100.59836	100.826047	99.8849119
100.196314	100.54838	99.2302475	101.449924
101.770286	102.39815	100.454487	99.1240155
100.492459	101.151774	100.107815	99.5844025
99.5373954	100.018554	99.4993623	100.023686
100.824764	101.389304	98.5580161	100.06775
99.530699	100.52908	100.75938	99.2285184
100.03895	100.541947	100.438709	100.978563
100.266458	100.203795	99.1075792	99.8151869
100.85992	100.897748	100.239372	98.7856892
99.6370032	99.7785495	100.673823	99.8270047
100.479569	100.337241	99.6893649	100.761285

101.816825	100.940635	99.7355878	99.6421408
99.910046	99.7983437	100.689379	99.7160864
99.9893975	99.9121605	99.8958122	100.684807
99.6750049	99.3018386	101.388722	101.283124
100.38063	101.383366	100.47182	99.4106811
99.3668064	99.8258907	99.6644757	99.3259307
100.967898	100.63201	100.785158	100.860554
100.382305	100.603639	99.2633591	100.196057
99.0043677	99.4867497	99.9860357	99.8948726
99.1101697	99.5347506	99.8351447	100.432582
99.5407435	99.5693905	99.2133584	100.099827
100.018192	100.190269	100.972049	99.3630723
99.2663615	99.5283175	99.5078069	100.506358
99.4908559	99.9786361	99.0762454	100.505008
99.0939311	99.6023809	99.6962539	99.4358361
100.12617	100.462275	100.567599	100.593979
99.5437569	100.215837	99.2109139	100.104047
99.6303069	99.834963	100.455153	99.3880585
99.910883	99.9210679	99.4338059	101.113117
100.652334	100.619144	99.176469	99.8869378
99.763229	99.7752504	99.5840301	99.4847955
99.7076495	100.093608	100.390041	100.580642
100.072097	100.581535	99.452695	99.6814772
99.064802	99.4912034	99.7806995	99.5418585
100.193301	100.658898	99.7549214	100.858191
99.8179715	100.295838	98.7980194	100.765843
99.4108348	99.670506	99.6413643	99.8950414
99.5961556	99.4842754	99.3869163	99.7626822
100.14425	100.023008	98.5222378	100.015076
100.23013	100.12	101.300276	100.060659
99.2815956	98.9163462	100.430708	99.7434361
99.8136189	99.194455	101.392499	100.186603
100.320196	99.8404064	100.470931	100.482216
98.4624674	98.0990097	101.146274	99.455251
99.2385717	99.0811331	100.826936	100.397128
99.5062574	100.063586	100.695601	99.7704482
99.3016846	99.0699163	100.03937	99.6309984
100.252061	99.9124904	100.504043	101.125104
100.84117	100.516049	99.3462491	99.7015674
99.5851067	99.3785412	99.2329142	99.5366249
99.3087158	99.0542459	100.252484	100.507371
99.8859392	99.5108326	100.570044	99.7414102
99.3102224	99.1335877	99.6384753	100.20568
99.8119448	99.7612295	99.5391406	100.245692
99.1126808	99.0176266	99.9073679	99.2955422
100.940778	100.572133	100.169149	100.451659

100.456801	100.217156	99.6291419	99.7253718
99.7389548	99.4372641	99.544474	99.7138917
99.8762295	99.8323238	100.482709	100.902423
100.311156	100.231507	100.264484	99.1652089
100.245197	100.114062	99.7009206	100.209394
100.052176	99.8722421	100.28204	100.5305
100.06289	99.3310351	100.36893	99.4638611
99.8420783	99.4374291	100.497154	99.7876684
99.4334349	99.595288	100.298262	100.363532
99.7441445	99.4727288	100.066259	100.189979
99.0569339	99.0755247	101.149607	100.933824
99.3564271	99.0727205	99.7453657	99.8243035
100.478732	99.7401157	99.3138042	99.7834478
99.7250599	99.2302495	100.545377	100.543669
99.8784058	99.6840321	99.3829163	99.384682
99.5643481	98.9630275	99.2975818	99.3608776
100.195812	99.4031191	99.9684799	99.8531727
100.106081	99.4202741	99.8880343	100.121774
100.018359	99.5525654	99.876923	99.4086552
100.141906	99.6081542	100.067592	99.2256484
100.735034	100.385902	100.440709	101.173557
100.363722	100.511431	100.132482	99.6279595
99.9709826	99.2642296	99.8035887	99.5911555
99.6612774	99.5050593	100.861381	101.015198
100.770524	100.643227	99.5804745	99.3492287
99.5650177	99.3407673	99.6464754	99.8742759
99.8805822	99.2683534	99.9422573	100.41418
101.362312	101.276147	99.9629242	98.7136008
99.087651	98.8287843	100.045551	100.148437
99.2489278	98.8599359	100.268039	100.110957
99.5917657	98.8651825	100.129178	99.454377
99.6424765	99.4259114	99.9328647	100.441188
99.6321681	99.4587025	99.427108	100.228294
100.018567	99.4208287	100.150251	99.337209
99.5008189	99.085867	99.6735534	100.320306
99.4366407	99.1909627	99.2833666	99.7819072
99.1212364	98.5597329	99.3064362	99.6336744
99.884558	98.9983146	100.347674	100.316761
99.6536162	99.104394	99.8896091	99.5430127
99.2983084	98.4079098	99.3716522	99.663895
99.5259249	98.9814272	100.624065	100.704901
100.442875	99.7379192	100.052427	99.434455
99.9454109	99.5623226	99.3871798	99.9142699
99.7412378	99.2294923	99.9448431	100.268475
99.9763361	99.4237799	100.016048	99.3746892
99.6529512	99.0588143	99.88606	99.7209596

100.346608	100.405055	100.777789	101.095573
100.538145	100.482115	98.8541389	100.568486
100.786212	100.152891	100.333034	98.8709005
99.7560353	99.2129328	99.443301	99.5835319
99.7475558	99.9717202	100.037122	99.1886386
100.257989	99.8210448	99.2234743	99.2651185
100.496911	100.346031	100.655786	100.356605
99.5089659	99.4134507	100.166223	100.899224
99.6487946	99.3521313	99.6604659	99.6178044
99.0482462	99.1647298	100.643808	99.1341065
99.9582133	99.590359	99.7902324	99.4211175
100.256991	99.8223565	99.4160168	100.355423
101.24344	100.887413	98.4320095	100.880653
99.4313202	99.3906609	100.109214	99.2614043
100.267632	99.6256095	99.3536845	100.536408
100.073601	100.146169	99.4816764	99.6564665
99.7849654	99.5908509	99.9290937	99.1872879
100.039683	99.8082563	99.8718633	100.648512
98.8237888	99.0778332	99.6054537	100.097957
99.8702592	99.6439726	100.179976	99.1373142
99.8567918	100.013857	99.4093621	100.565785
99.6303392	99.4227962	98.9078201	99.9401009
100.867848	100.615083	100.299316	99.2931443
101.287666	100.901186	99.6209814	100.887068
99.3548384	99.6320038	99.1045772	100.199086
99.3172626	99.7823513	100.946374	100.024854
100.555935	100.470638	100.323495	101.482532
99.5792959	100.99546	102.859155	98.2222556
102.086569	101.836717	99.034481	100.666576
98.3023824	98.6618773	99.9419594	100.070438
99.932276	99.86105	101.112742	99.5842073
100.214594	100.197979	100.048878	100.070438
99.0628775	99.3057318	100.486313	100.004087
99.4053829	99.2394936	99.8481282	100.201281
100.43689	100.131249	100.074388	101.327884
99.4151926	100.103705	98.8379458	99.7881539
98.859702	99.0560271	100.100563	99.0880159
100.291574	100.128954	100.812393	100.671135
99.9104953	99.3545906	100.495186	100.576759
99.7776497	100.083046	100.951033	99.1839113
99.5252598	99.9376174	100.037787	100.510915
99.6963463	99.2235899	99.7904542	100.477993
100.445369	100.353573	100.025587	99.0807562
100.606147	99.9674574	100.215245	100.60985
99.4000625	99.7049641	100.60033	100.220021
99.3280698	99.4646049	100.790876	99.717583

98.8580393	99.4223043	99.4794582	100.044775
99.6211946	99.4823122	100.313735	100.333644
99.9670253	100.211588	101.028227	98.6524345
100.549118	100.311928	99.819513	99.7422321
100.363899	100.287499	99.7028341	100.69207
101.157481	101.073011	100.10389	99.8114524
99.9287844	99.8264554	101.091225	100.385137
99.1909014	100.323405	101.765124	99.2023138
101.30978	101.318289	100.394257	101.057755
99.0931377	99.0663563	99.8865036	100.745926
100.343615	100.256347	101.157106	99.029263
100.161555	100.345375	98.8375021	99.9784253
99.6915246	99.7733337	100.531122	100.458915
101.030289	100.912826	100.502285	100.354579
99.3821059	99.9571281	102.105844	97.8982709
101.660598	101.711127	97.3934337	100.825277
99.6594355	99.3673791	98.6374177	100.167346
100.478456	101.003002	100.74141	98.8410176
99.6657536	99.6821743	100.372518	99.7584398
100.226897	100.050419	101.084348	101.307286
101.496661	102.403021	100.239424	99.8431925
99.7169631	100.365542	99.4262207	99.5416621
99.8735845	100.160269	100.23521	100.806705
100.86419	101.82065	100.782004	98.6163049
100.030705	99.9171229	98.3996233	99.7049207
101.067532	100.623608	99.9355265	100.718745
99.625185	99.9587677	99.7733738	99.553649
98.7777334	99.6359387	99.9193334	99.418585
102.281098	101.756871	100.005401	101.863412
100.407959	101.800155	98.0779798	99.1928593
100.064623	100.672795	100.591014	99.2455343
100.221244	100.542614	100.768694	101.473077
99.130381	99.4450942	99.0568852	100.528642
101.014992	101.529136	100.197943	98.8518227
99.8882158	100.257003	98.5850674	101.393727
98.5123749	98.4639827	100.690169	101.44809
101.474881	102.573043	99.9457304	99.6078435
99.4227754	99.3324714	99.3391382	99.037549
101.168916	100.551239	100.020965	101.572267
100.132906	100.750929	97.8892022	100.024172
98.3946039	98.8577971	99.8836683	98.6304488
98.9971641	99.6264421	100.265837	100.292086
100.155087	99.8701628	99.8355367	100.320598
99.8917484	99.7460928	99.2779202	100.907038
99.5133346	99.9480749	100.829221	98.7578258
101.448098	100.933433	99.6432322	100.365644

100.845371	100.234189	100.071315	99.7682374
100.579031	100.032698	100.815691	99.6835444
99.4426217	98.3841049	102.036059	100.840229
100.637902	100.792013	99.9149427	98.4332253
99.9307739	99.0533957	99.2071645	99.4908761
100.704946	99.9505301	100.173345	101.167022
101.486957	102.280199	100.506495	98.8870586
100.650244	100.397542	99.297439	99.6806763
99.0463629	99.8046905	98.8354202	100.146488
99.8040245	99.8717996	100.734954	98.8408317
100.295179	99.7650798	99.5922171	100.441227
100.09338	99.5144845	99.0609954	100.063651
100.628897	99.9297426	99.8484013	99.5571797
100.310689	99.5311799	100.60298	100.53368
100.160424	99.65214	99.4418337	99.5742195
100.367559	100.29917	97.0864917	100.764815
98.706641	98.451214	99.7973863	100.753849
100.486137	100.136144	99.9431119	98.9393591
99.666101	99.7091009	98.1737774	100.870091
99.1159083	98.5142311	101.19298	101.654935
101.541159	102.040734	99.8909878	99.1134691
99.0155094	99.1936701	98.4993864	100.098911
99.5923862	98.9553509	101.727529	101.717865
101.558003	102.708715	100.014533	98.4921056
99.9596261	100.000125	98.8507247	99.2944962
99.5481907	99.5611335	99.9992284	100.611456
100.095048	100.436335	100.876465	98.5774735
100.54751	100.590686	99.3433525	100.519677
99.0341883	99.6110561	98.9760442	99.8892034
98.031033	98.6792214	99.8182359	100.170445
100.26616	99.9804837	101.386394	102.103876
100.650411	101.755438	99.9621871	99.1794352
100.010826	100.454503	98.453251	100.060783
98.1095843	98.7787393	99.977048	99.8913966
99.3365525	99.5616245	99.8233374	99.4443118
100.216627	100.56908	100.172458	99.7571025
100.083207	99.8154934	100.875578	99.3697415
99.8328767	99.5814299	98.6216006	100.496733
99.0940607	98.7662996	100.810589	100.345567
99.5370167	99.8461017	100.253638	99.4832841
100.773491	100.687912	100.284913	100.926271
100.447778	101.402706	100.244988	99.2772877
99.8041912	100.213565	98.6355743	99.965967
99.090892	99.482403	100.484537	100.247209
99.472141	100.053976	99.8308788	100.126411
99.6450873	99.3966343	100.795285	100.583113

100.329034	100.825077	99.908954	99.3748028
100.035009	100.425041	98.2591721	100.280951
99.3080338	99.3552231	100.132089	100.611625
99.9227687	100.13418	100.684161	99.3791893
100.66442	100.273963	100.640687	100.845796
101.366045	101.972315	99.9016344	98.8212612
99.8090277	100.202599	98.6253713	100.198282
99.3550645	99.3755195	101.021082	100.737315
99.4843156	99.8996254	100.504277	98.9960461
100.915084	100.643882	100.355003	100.654984
100.528998	101.441989	99.6323638	99.6255077
99.8478865	100.284603	98.9090593	100.062807
99.4399533	99.6853672	100.449713	100.63187
99.4047637	99.9498754	101.998574	98.3453268
101.223785	101.077636	99.4535893	100.340337
99.4579651	99.6554136	100.18377	99.7523786
99.2813497	99.230662	100.171349	99.4893577
100.43477	99.6745643	101.317191	100.82437
99.9206006	100.563187	100.896649	98.8705249
100.375564	100.197197	99.6709577	100.151381
99.4399533	99.4717638	100.252307	99.9705222
99.3942568	99.7559136	99.7672209	99.9838504
99.792517	99.8082915	100.656213	100.778986
100.285506	100.480692	99.9834803	99.0331625
100.416591	99.9827752	98.982033	99.9570253
99.6599304	99.0263883	99.920266	100.211273
99.3500613	99.6866767	99.7503638	99.8529305
100.562687	100.068544	101.005334	100.657683
99.6045609	100.056923	101.202074	98.7590068
101.277487	100.777118	99.3515593	100.422668
99.379247	99.8624698	99.6288149	99.9197401
98.801036	99.1110113	99.5529577	100.050491
99.4843156	98.9160675	100.975612	100.842591
100.457785	100.041209	100.644901	99.6315813
100.665254	100.077874	99.1168901	100.412714
100.773324	100.568589	99.9548675	99.7478234
100.638069	100.050703	100.125435	100.702392
100.084875	100.19065	99.930469	100.020967
99.4758101	99.5871587	98.8695781	100.170782
99.3003622	99.0008541	100.88445	101.050443
99.6385831	99.5184128	101.297672	99.1461991
100.887065	100.318812	100.984484	100.689907
100.489472	100.517193	100.106138	99.8571482
100.429433	100.022386	99.5999803	100.126918
99.5014935	99.1789388	100.049134	100.143789
99.3535542	99.4613856	100.083294	100.034678

99.4710561	99.5932368	100.026998	100.081675
99.3334714	99.6516065	100.171105	100.068799
99.2938344	99.4186945	100.273263	99.9983032
99.1793273	99.0546867	100.265438	100.015847
99.463481	99.2509525	100.199144	100.022124
99.508403	99.3144224	100.294564	100.017134
99.4322998	98.9940503	100.162411	99.9760921
99.5494494	99.4615745	99.9565751	99.9421317
99.3130363	99.2286624	100.133285	100.044174
99.3507356	98.8814666	99.9789627	100.028401
99.5633664	99.0847216	100.089814	99.966918
99.4555536	99.1072006	100.106985	100.151206
99.3339999	99.1708594	99.9574445	100.120464
99.5024134	99.2084502	100.101986	100.113704
99.4027042	99.2991216	100.100247	100.175026
99.1816174	99.0497754	100.016565	100.216712
99.4458645	98.9774272	99.9439684	100.145411
99.4007663	99.37487	100.226966	100.242142
99.4458645	99.1602811	100.121331	100.105979
99.676288	99.3975378	99.8970195	100.178406
99.3308289	99.4415512	100.155238	100.174865
99.276394	99.3236784	99.988309	100.21639
99.7730024	99.5363783	99.946794	100.180659
99.4953668	99.4315396	99.9437511	100.006834
99.4978331	99.4043382	99.968747	100.059464
99.8980793	99.5188108	99.9441858	100.072018
99.867779	99.8265267	100.275002	100.093907
99.8513957	99.8059368	100.001785	100.401
100.049757	100.176934	100.053516	99.982852
99.8679552	99.9511998	99.8574607	99.8814536
99.9489909	99.9105866	99.8202929	100.161667
99.7104639	99.6030596	100.120244	100.15877
100.200554	100.785565	99.8478971	100.274815
99.9431775	99.9591336	99.9576618	100.117567
99.8979032	99.9570557	100.102856	100.098897
100.263797	100.461226	99.8781095	100.036287
100.018048	100.586466	100.026129	100.057694
99.9435298	100.032804	99.9670081	99.8701871
100.13643	100.583255	100.083728	99.9274852
100.274367	99.9772679	100.213055	99.6931423
100.492811	100.537541	100.111333	99.8904668
99.9114679	100.190157	100.00548	100.042403
100.235435	100.456126	99.9802669	100.105657
99.9625556	100.266661	100.232182	100.084089
99.9047736	100.059061	99.7670406	100.04852
99.7449922	100.363566	100.033519	100.296865

99.9993741	100.315963	100.103942	100.112899
100.005892	100.143876	99.8750666	100.310224
99.8521003	99.7568231	100.376507	99.9748045
100.156689	100.452726	99.9068005	100.005707
100.151933	100.356387	99.9267973	99.7588098
100.053633	100.318419	99.7459571	99.9213691
100.117757	100.082673	99.7553034	99.9894509
99.9599132	100.031293	100.079599	99.9558124
100.207601	100.25986	100.066992	100.104691
99.9516334	100.266094	99.9391866	100.086987
99.8526288	99.9880351	99.8200755	99.9691713
100.223808	100.321441	100.074382	100.163921
100.087456	99.9425105	100.335861	99.7972768
100.416708	100.626701	100.093727	100.055601
100.211829	100.254382	100.324341	99.7890684
100.004307	100.339764	99.8065994	100.105174
100.186637	100.303874	99.9126691	99.7835961
99.9602655	100.049238	100.011131	99.9694932
100.180119	100.102885	100.004611	99.9300604
100.064731	100.283284	99.8350731	99.9099417
100.17043	100.189401	99.943099	99.8497465
100.532625	100.762897	100.140676	99.8763032
100.308367	100.224914	100.279131	99.6432478
100.430978	100.640869	99.7589985	99.8505512
100.266968	100.535841	100.15263	99.9382689
100.271549	100.552842	99.709876	99.8265697
100.473786	100.33202	100.35977	100.000878
100.265383	100.126309	100.318907	99.7892293
100.301144	100.46916	99.8596343	100.053187
100.099964	100.339009	100.14198	99.9310261
100.037073	99.9353323	99.8165978	100.189512
100.135549	100.007492	99.8396375	99.832042
100.012058	100.223592	100.010914	99.8700261
100.028441	99.9436439	100.035475	99.9554905
100.354522	100.680727	99.8694153	99.98591
100.285113	100.245882	99.9970033	99.9184721
100.441019	100.487483	99.7144405	100.137525
100.336729	100.368855	99.8089904	99.8972267
100.261507	99.8102814	99.6574932	99.864071
100.640437	100.118753	99.8535483	99.7736172
100.241072	100.253438	99.9743982	99.9321528
100.352408	100.006736	99.8709368	99.7810209
100.325279	100.284039	100.002437	99.8964219
100.126917	99.9196538	99.9065832	99.697166
100.452294	100.456315	99.7931234	99.9678837
100.285642	100.398323	100.3902	99.8548969

100.110005	100.141232	99.860069	99.9484087
100.17642	100.139721	100.084598	99.9302214
100.12128	100.10723	100.087641	99.9496963
100.219404	99.781191	100.068513	99.9150921
100.442252	100.31313	99.9317965	100.024377
100.178357	99.7847801	99.65858	99.8397676
100.609961	100.365832	99.738567	100.028079
100.154569	100.213357	100.098434	99.9488754
100.294637	100.182945	99.9717285	99.8978719
100.337626	100.434745	99.8217284	100.001971
100.443161	99.8710741	99.9647619	99.8524996
100.377444	100.046938	99.877026	100.085475
100.342559	100.31064	100.283267	99.9686654
100.107528	100.001414	100.044878	100.09545
100.328993	100.181811	100.269769	99.8491209
100.062248	100.134398	100.064689	99.8335141
100.13836	99.7643468	100.153078	99.8563611
100.524206	100.475359	100.10257	99.8705198
100.323179	99.8984643	100.020059	99.7844413
100.633264	100.451557	100.074051	99.9168574
100.387838	100.45968	100.102353	99.8817824
100.592918	100.532595	99.9347183	99.9314988
100.632207	100.497648	100.165488	99.894654
100.389953	100.584164	100.081453	99.7733396
100.504825	100.100018	100.164182	99.780419
100.500245	100.593609	99.9501755	100.040907
100.277194	100.386388	100.027679	100.006476
100.76417	101.155013	99.9303642	99.9511279
100.465889	100.973671	100.241903	99.9968219
99.9808508	100.346719	100.064472	100.030449
100.337273	100.400744	99.8058357	100.114275
99.7516339	100.079617	100.277389	99.9808934
99.6910262	99.9298214	99.8434991	100.078717
99.9309904	100.311584	100.096039	99.9537022
99.9232382	100.356542	100.207723	99.8491209
99.856464	100.151021	99.9493047	100.136156
99.9607656	100.256237	99.9469099	99.9181446
99.825984	100.346719	100.092121	99.8507298
99.4806611	99.7813476	99.9286225	100.00004
99.856464	100.08774	100.198144	100.019025
99.8790157	100.15801	100.143282	99.8787254
99.3427081	99.4007184	100.119334	100.024978
99.865978	100.059594	100.11498	99.9977873
100.037054	99.998958	100.393427	100.03431
99.8610448	100.145921	100.399523	99.9245803
99.8744349	99.6967214	99.9769535	99.8431678

99.8744349	99.8848637	100.184646	99.9388999
99.8399026	100.104741	100.347709	99.9366474
99.7699571	99.767747	100.148071	99.9643212
99.9443804	99.9222655	100.14633	99.8771165
99.8399026	100.040704	100.183122	99.8697153
99.9997026	100.104552	100.082977	99.9437268
100.465184	100.257748	100.113238	99.9168574
100.162498	99.5542924	100.501627	99.9023769
99.9577705	99.7853145	100.126301	100.050561
100.125851	100.08264	100.177462	100.080004
99.7861662	100.192201	100.273035	99.9070428
99.9708082	99.7817254	99.9651973	99.9084909
100.083391	99.9322771	100.07318	99.9112261
99.8901154	99.9895131	100.035299	99.9384172
99.6610747	99.4498318	100.066866	99.8294917
100.091495	99.9925355	99.9697692	99.8769556
99.8855346	99.7329898	99.8711479	100.099633
99.795504	99.3259148	99.9120768	99.876312
99.98966	100.006703	100.169624	99.9340731
99.7863423	99.8975198	100.130655	99.8621533
99.7711904	99.5102792	99.7026426	99.8573265
99.9545992	99.8648405	100.108666	100.1117
99.9500184	99.9987691	100.403006	99.8417197
100.043749	99.9050758	100.009174	99.7562848
100.015383	100.363531	100.165705	100.074212
100.021197	100.349175	100.22514	99.8965847
100.030711	100.295528	99.8953133	99.9680218
100.199497	100.253592	100.120422	99.9131568
99.9824364	100.222046	99.9658504	99.8468683
99.6041669	99.4913894	100.075792	99.9237759
100.151222	100.341619	100.05707	99.9516106
100.059782	100.341241	100.099305	99.9641603
99.9841983	100.088496	99.7352987	99.9574028
100.274551	100.366553	99.5746309	99.9491972
99.927819	100.164244	99.8489417	99.966252
99.8518832	99.9653342	99.6024974	99.995213
99.8818347	99.8833525	99.9941523	100.076786
100.077753	100.216946	99.8149794	100.173162
99.79938	100.009914	99.9255746	100.172358
99.9336331	100.307239	99.7396528	100.141144
100.034763	100.554507	99.7753568	100.155625
99.7253823	99.8612514	99.7979983	100.248139
99.7454674	100.074895	99.8652698	100.089658
99.7142826	99.653086	99.7263727	100.1907
99.8099512	99.773225	99.9671567	100.170427
99.6475084	99.2278768	100.003078	100.042677

100.065948	100.068284	99.8202044	100.310727
99.832679	99.6440189	99.9954586	100.224487
99.9699273	99.6406187	99.7655599	100.19617
100.041106	99.9613673	99.6662855	100.347089
99.8152367	99.4430315	99.6342826	100.251196
99.9928313	99.6748092	99.7146165	100.158521
99.766962	99.5165128	99.8008285	100.085314
99.9253524	99.6188954	99.683049	100.260689
99.8224603	99.6139841	99.9582307	100.085636
99.9265857	99.4265974	99.5674465	100.135835
99.8920534	99.2641452	99.639943	100.292868
99.7757712	98.8665151	99.6438617	100.150476
100.156155	99.469855	99.6129473	100.163508
99.8162938	99.2031312	99.934936	100.181207
99.6626837	98.8371499	99.7018804	100.189487
99.794182	98.9731063	99.9098474	100.309878
99.8712125	99.3834271	99.8898338	100.222965
99.9036464	99.2201285	99.6470607	100.198501
100.048541	99.4290602	100.051683	100.082777
99.8007041	99.652323	100.073219	100.170978
99.9544126	99.4531967	99.7284202	100.154561
100.012053	100.032096	99.6163878	100.140719
99.9789143	99.4424485	99.4297396	100.247107
99.8414227	99.3888956	99.6181281	100.304084
99.4379379	98.8052821	99.9120228	100.157619
100.239796	99.6279979	99.6981823	100.269319
99.9755651	99.4890244	99.7042734	100.216849
100.369179	99.9153733	99.3894949	100.390353
99.910521	99.6528887	99.7514793	100.197052
99.8470634	97.4868249	100.409969	100.846325
100.480582	99.924236	99.6622884	100.654955
100.487104	100.700564	99.7547423	100.034171
100.479701	100.263467	99.4656335	100.279298
100.344501	100.383584	99.6213911	100.227954
100.206657	99.9212189	99.9455237	100.203973
100.159769	100.007771	99.9890315	99.9062152
100.040962	99.6879621	99.9339942	100.113197
100.298846	100.177669	100.000561	100.22377
100.022806	99.581422	100.196128	100.02226
100.448677	100.08942	100.159364	99.9797694
99.8668058	99.581422	99.9259453	100.193189
100.20084	100.20124	99.8067341	100.180313
99.8713888	99.5299433	99.662941	99.9400148
100.335863	100.069621	100.061689	99.8434447
100.125924	100.155984	100.292933	100.095493
99.9626973	99.5910389	100.020792	99.9379224

100.133857	100.208971	100.025796	99.8429619
99.9316736	99.6745739	99.9594462	99.9395319
99.9986566	100.016822	99.9033212	100.030147
99.5957006	99.201649	100.20396	99.8386162
100.03303	100.012297	99.9118053	99.855355
99.5812464	99.7417035	100.276182	99.8511703
100.003768	100.061512	100.121295	99.9928064
99.9112261	100.170692	100.022097	100.001337
99.9637549	100.298729	99.7675771	100.137179
99.843538	100.122042	100.056686	100.104828
99.7825481	99.782811	100.288365	99.9865293
99.9958363	100.083009	99.8030359	100.095975
100.015226	100.438268	99.4867347	99.8725767
99.8044058	99.9459211	100.001431	100.298933
99.7247312	99.8827513	100.224408	99.977838
99.6850701	99.7626344	99.9738038	100.115611
99.7807854	99.5122181	99.9324714	99.9063762
99.6942363	99.5682223	99.679909	99.736252
99.9491244	99.5936788	100.185251	99.7602335
99.9679854	100.155419	100.327739	99.865012
99.7543447	99.7060645	100.035585	99.9950597
99.937138	100.076598	99.9781545	100.030308
99.7425346	99.9168819	100.164585	99.9723657
100.05806	100.367367	99.8719957	100.038194
99.8861956	100.020971	99.9605339	100.017915
99.8148058	99.9289501	99.8324036	100.09002
99.7534634	100.05793	99.89636	100.080685
99.675904	99.7611259	100.152185	99.8048167
100.158358	100.231034	99.8767815	100.096136
99.9149278	99.9949484	100.250078	99.7111438
99.7740871	99.9798631	100.398222	100.02564
100.001124	100.070941	100.259432	99.6985897
99.8690973	99.8344783	99.8441507	99.9210226
100.11006	99.8414552	100.173939	99.7206398
99.8761481	100.049633	99.9633619	99.9825056
100.08644	100.432046	100.150663	100.144582
99.9106973	100.102431	100.028406	100.074086
99.9103448	100.30778	99.9174613	99.9118485
99.9827922	100.279872	99.952485	99.8687139
99.7411244	100.057364	100.354279	99.6668825
100.23345	100.368687	100.105632	99.8017586
99.8754431	100.403761	100.283579	99.7187084
100.028799	100.155419	100.123035	99.7193522
100.017694	100.452034	100.078875	99.7537955
99.9077007	100.056044	99.9648847	99.8473075
100.168758	100.458822	100.347318	99.8957535

99.8765007	100.384715	100.297936	99.9905531
99.8625753	100.237445	100.170894	99.9882998
100.123457	100.376607	100.029929	99.8659777
99.7978837	100.450337	100.043851	100.052519
100.089965	100.158058	100.08192	100.039482
99.8511176	100.263279	100.351668	99.7805132
100.027918	100.103751	100.196781	99.9924845
99.8195651	99.7147385	100.166543	99.9591678
100.070046	100.434308	100.191777	100.036585
100.287918	100.406778	99.9413905	99.9298749
99.939782	100.164281	100.015789	99.8807851
100.234155	100.389241	100.122818	99.9879779
99.8588736	99.9510124	100.190907	99.8181756
100.25848	100.391315	99.8195689	100.227633
100.083443	100.199543	100.258997	99.8788537
100.437396	100.187475	99.9620567	99.9025134
100.031796	100.122797	100.083008	99.8976849
100.130331	100.211611	99.9052791	99.8481123
100.364772	100.231788	100.311206	99.8286373
100.161531	100.305895	100.075612	99.8440885
100.068636	100.297975	100.087141	99.8949487
100.20225	100.625892	99.9244225	99.9868512
99.9357278	100.08414	99.8970126	99.8112547
100.446691	100.975165	99.823937	99.8683344
100.070195	100.654254	100.129057	99.7014921
100.129243	100.581784	99.9008689	99.8966509
100.340581	101.036282	100.101022	99.9418608
100.271486	100.87488	99.7546113	99.5816296
100.497278	101.050095	99.7522208	99.8804011
100.574657	101.400902	100.338772	99.7169375
100.296339	100.73505	99.861751	100.011526
100.504505	101.11878	99.9884496	99.8379263
100.5283	101.027957	100.154918	99.7956124
100.414082	101.05123	100.297047	100.113851
99.8630877	100.6552	100.17339	99.9491008
99.8255439	100.227949	99.9341191	100.077812
99.9039804	100.267685	100.079507	100.16067
99.8597387	99.831541	100.284442	99.9043736
99.7821835	99.6758159	99.8391495	100.101141
99.7994571	99.8360822	99.9923614	100.074434
99.6510447	99.8235939	99.9478104	100.046278
99.5683779	99.5564203	99.8682706	100.072986
99.5160281	99.3632303	100.171	99.9899667
99.63712	99.6671119	100.076248	99.9090393
99.537532	99.6018322	99.986711	100.109347
99.7613846	99.6232137	100.089504	99.7948079

99.8535695	99.9995652	100.270968	99.8635077
99.6937	99.4430796	100.013224	99.9029256
99.7827123	99.77383	99.9399868	100.037751
99.9170238	100.180645	100.264448	99.9674422
99.9127935	99.3899098	100.065381	99.8845842
99.9834745	99.9596405	99.8356724	100.00316
100.021195	100.118772	100.190993	100.108864
99.8246626	99.3738264	99.9628056	99.9293114
100.340228	100.039301	100.071467	99.9318856
100.041993	99.8629509	100.108194	99.9775782
100.035295	99.8459215	99.8504502	100.059793
100.202921	100.165319	100.068641	100.035016
100.039702	100.028515	100.09472	99.8059093
99.979068	100.167589	99.9630229	100.023753
99.9995143	99.8881167	100.241412	100.027293
100.288936	100.47412	100.130795	100.074594
100.064907	100.368348	100.16448	100.071216
100.039878	99.9471523	100.337034	100.098728
100.130477	100.312151	99.986711	99.9997809
100.051335	99.8517872	100.036695	99.8469361
100.371251	100.376295	100.055819	99.9605239
100.318725	100.278849	100.012138	99.9939889
100.041641	99.6989003	100.003662	99.8741264
100.113203	99.9365561	99.8048127	99.8715521
99.8911133	99.8744931	100.144921	99.9711426
99.9275995	99.7515024	99.9997503	100.022788
100.157797	99.897956	99.7391815	100.015065
99.8764836	99.7227415	99.9845378	100.039842
99.8958724	99.7119562	99.9332498	99.9824049
100.146517	100.114609	100.004966	100.166301
100.175424	100.078847	100.074509	100.019409
100.043404	100.072035	99.7785167	99.9759693
100.297044	100.423978	99.962371	100.169358
100.118139	100.312718	100.139488	100.098728
99.9993381	99.8063752	99.6963691	99.9280243
100.153038	99.9157423	99.8578392	99.9416999
100.18124	100.1095	99.8504502	99.8926286
100.131182	100.117069	99.9128216	100.009434
100.290346	100.139018	100.047778	100.140237
100.366315	100.770055	100.00301	100.056897
99.8678467	100.187836	99.9438986	100.008952
100.099455	100.250088	99.9747583	100.211511
100.421661	100.649713	100.082115	100.181264
100.066494	100.044977	100.371153	100.09004
100.137351	99.835893	99.9654135	100.011848
100.287526	99.8932256	100.16774	100.06478

100.134355	100.074495	100.122102	100.05384
100.084649	100.290769	100.210118	99.9627764
99.9055668	99.8097811	100.197078	99.8628641
100.010443	99.9800759	99.8137229	99.9664768
100.035472	100.083388	99.8913067	100.006056
99.8669654	99.6099686	100.020179	99.9491008
100.111088	100.131638	100.049517	99.9439523
100.184766	100.068629	100.08494	99.8958464
99.9632044	99.8230263	100.217072	99.9351034
99.8482817	99.7753437	99.9343365	100.000585
99.8590337	99.9418542	99.7370083	99.8551414
99.8523357	100.064656	100.026046	99.9693728
99.8477529	99.7613417	99.7211438	99.9439523
99.9603842	99.7000356	99.842844	100.057218
100.049396	99.9409081	99.7204918	100.003803
99.6804804	99.6179156	99.8408881	99.9563408
99.6697284	99.4945465	100.008443	99.9088785
99.9746614	99.9291767	99.8519715	99.9663159
99.8239575	99.6525422	99.961719	99.9746822
99.9283046	99.7149837	99.6618149	100.01764
99.9327111	99.8735471	99.7420067	100.217142
99.8667892	100.139964	99.8715305	99.9175665
99.7058621	99.7255798	99.6570339	99.9936671
99.7497513	99.2760015	100.00953	100.120126
99.8147919	99.4322943	100.16209	100.24385
99.7860612	99.6958728	99.7819939	100.074434
99.8407024	99.9267169	99.8093764	100.068159
100.082886	100.148668	100.064947	99.9204625
99.9043329	99.7861291	100.18556	99.9418608
99.9676109	100.003917	99.8900028	100.104681
99.9549201	99.9011727	99.7213611	100.153752
100.174777	100.117155	99.993549	100.361948
100.035777	99.9777222	100.480839	100.151885
99.9429646	99.895881	100.151532	100.03505
99.8654032	99.8180354	100.10389	100.036843
100.12031	100.096488	99.8508113	100.067769
100.320895	100.189352	99.9672502	100.188937
100.14413	100.046061	100.296366	100.018316
100.16127	99.9906735	99.8441413	100.129175
100.004113	99.9937046	99.9584839	100.111695
99.9696899	100.054603	100.024803	100.0851
100.036213	100.030905	99.6093579	100.082561
99.8206674	99.8061863	100.050149	99.970208
100.148633	100.060115	99.8854952	100.026833
99.8548003	100.113986	100.284741	99.8190101
100.121327	100.111644	99.976207	100.097501

100.106947	100.090426	100.16411	100.029223
100.084144	100.205059	99.8736798	99.9690128
100.037229	100.068932	99.7915437	100.020408
100.020671	100.177641	100.17745	100.023844
99.862934	99.9785489	100.081021	99.8929656
100.120455	99.9888823	99.8677721	100.086445
99.9996106	100.147605	99.4003015	100.094812
99.8681629	100.04923	99.8102197	100.181766
99.698806	99.9297748	100.320759	100.062242
99.7709933	100.043994	99.9752542	100.224944
99.8258963	100.113986	100.16735	100.049691
100.05829	100.084915	100.127139	100.015328
100.07296	99.9322548	100.428432	100.152781
99.9823263	99.9102101	100.559164	100.155769
100.051609	100.151049	100.3442	100.079423
100.089373	100.383622	100.319044	100.136944
100.24406	100.441765	99.9150337	100.172502
100.363307	100.568384	100.091884	100.149643
100.281533	100.398502	100.15077	100.104822
100.179716	100.29682	100.018514	99.9634848
100.132366	100.327821	100.09417	99.9736443
100.176811	100.300403	99.7288459	100.024591
100.215301	100.321896	99.9481931	100.120509
100.383496	100.394368	100.153819	100.05507
100.296929	100.458712	100.024231	100.017569
100.290829	100.452098	99.6998791	100.20313
100.00542	100.054741	100.166206	100.090778
99.8282202	100.054052	99.8845424	100.009352
100.031565	100.052261	99.9203697	99.96468
100.242607	100.126524	99.8207011	99.9422693
100.052044	99.999078	99.8986446	99.9050674
100.134399	100.066315	100.043479	99.9640824
100.023431	99.8906453	99.8710118	100.068965
99.9990296	100.03435	99.7995477	99.9673693
100.153281	100.133276	99.9729673	100.035498
100.388725	100.374252	100.088453	100.197901
100.151683	100.086706	99.9386645	99.9579568
99.8780396	99.9908113	99.8820649	99.8851965
99.8004782	99.9205436	100.142575	99.8376857
100.076591	100.070448	99.8708213	100.054323
100.127282	99.9339082	100.027661	100.114832
100.029531	99.9946691	100.036618	100.074045
99.8170363	99.8231332	99.9674407	99.8070577
99.8905308	99.8377379	99.8079328	99.9637836
100.043911	99.8741118	99.7602901	100.048795
100.157638	100.042065	99.9506705	100.0727

99.9885719	99.8767296	99.9598179	100.005916
99.8807993	99.8110086	100.282264	100.001583
100.026481	100.044132	99.8401393	100.112442
100.061631	100.10076	99.7092171	99.9911247
100.050302	99.842698	99.9716333	99.800185
99.6006196	99.6266592	99.6926374	99.8600965
99.8016402	99.6164635	100.351822	99.9188127
99.9386073	99.7826259	100.294842	99.8968501
99.9754998	99.9829578	100.005174	100.031613
99.7230621	99.7253095	100.025946	99.9485443
99.9740473	99.9329437	100.153629	100.109005
99.7616975	99.7925461	100.004602	100.122153
99.5704084	99.6728154	100.279405	99.9655764
99.8038189	99.8495869	100.143909	100.006364
99.9644611	99.8158309	100.149817	99.8953561
99.9532771	99.8688761	100.058153	99.7489391
99.8211032	99.7687102	100.224902	99.8929656
100.141371	99.8967076	100.020419	99.974989
100.038682	99.9476862	99.7913532	99.9927682
100.008035	99.9676642	100.052054	99.9336038
99.9634443	99.9191658	100.101412	99.9123882
99.8957597	99.8770051	100.056056	99.9171692
99.6771643	99.7317852	99.8193671	99.8893799
99.9946722	99.7470788	99.8729175	99.9824592
100.132947	99.9249525	99.8288956	99.975736
100.059016	99.9862645	99.9996472	99.9228466
100.006147	99.990949	99.9203697	99.9010335
99.9306187	99.9147568	100.104461	99.8364905
99.8765872	99.8984988	99.9929773	100.052978
100.061631	99.9248147	100.018133	99.9926188
100.069329	99.9483751	100.1542	99.9790229
99.9932198	99.7924083	99.8999786	99.892368
99.8957597	99.7924083	99.980209	99.8205041
99.7811605	99.740052	99.6562383	99.9070097
99.8447783	99.9602241	100.012034	99.9412234
99.7051968	99.550467	99.9041712	99.8678655
99.8572694	99.8269911	99.6966394	99.9567615
100.000192	99.8829297	99.798976	99.8919197
99.8639508	99.8837563	100.158393	99.8880352
100.233405	100.292327	99.9417526	99.8719084
100.205528	100.094179	100.125778	99.9386234
100.521378	100.365992	100.127494	100.030768
100.451611	100.445334	100.282723	99.8303237
100.300546	100.466104	99.8208492	99.9404184
100.124522	100.29385	99.9873298	99.9386234
100.127003	100.114257	99.7413275	100.202641

100.309157	100.272664	100.122154	100.168386
100.241433	100.306173	100.035958	100.072053
100.14481	100.070778	99.8473564	100.056048
100.110218	99.8792768	100.212355	99.8192544
100.295583	100.16909	99.9400363	99.9176815
100.229465	100.221708	100.280816	100.050812
100.187283	100.181414	99.9211571	99.9399697
100.036947	100.154551	99.8385842	99.8041463
100.238222	100.148458	99.784807	99.9995046
100.089346	100.116611	99.9945763	99.9576207
100.21808	100.275849	99.9608225	99.9935212
100.31339	100.256463	99.9615853	99.9718313
100.387244	100.306173	100.098889	100.124558
100.326818	100.267264	100.068568	100.122763
99.9930145	100.15469	99.9541481	99.8038472
100.069204	100.163967	100.176122	99.9037701
100.166119	100.067732	100.031572	99.9665959
100.237784	99.9953129	100.301221	99.9389226
100.047748	100.017329	100.379026	100.012668
99.7675111	99.9458799	100.038247	100.013865
99.916825	99.8427213	99.9762692	100.008629
99.8169905	99.677529	100.119484	99.9924741
99.9502491	100.002236	99.7746999	100.017754
99.9950579	99.8814923	99.8813009	100.072652
99.9908251	99.9858971	100.201485	99.8523128
99.7857557	99.8817692	99.9615853	99.7347388
99.5998066	99.8369056	100.043014	99.7818581
99.9174088	99.8295668	99.8954126	99.8553045
100.079859	99.8572604	99.854603	99.8484235
99.957401	99.9522494	100.083442	99.8394484
99.9266041	100.215754	100.179173	99.9262078
99.798162	99.934664	99.9501434	99.8258362
99.8385921	99.7968884	100.12921	99.9018255
99.8851523	99.8694456	99.8418261	100.025383
99.8826711	99.8418905	100.643526	99.9625571
99.7771442	99.9745427	99.9240176	100.087909
99.6806668	99.496136	99.924399	100.145201
99.6479724	99.7185155	99.9329804	99.9475985
99.4661101	99.6588358	99.9366037	99.9665959
99.7923238	99.7884418	99.7373228	99.9015263
99.8704107	99.7023148	100.045493	99.9643521
99.7661975	99.7217003	100.01479	100.060984
99.5404022	99.7254389	100.108042	100.053804
99.7175938	99.7413627	99.9739808	99.9498423
99.7759766	99.8051965	99.9863763	99.9876874
99.9460164	99.9788353	99.8776776	99.8841744

99.8089628	99.7535479	100.288063	99.9665959
99.568134	99.5746473	100.007353	99.9238145
99.5516409	99.6672824	100.043586	100.113937
99.8115901	99.6524663	99.6086007	100.0372
99.8289589	99.6528817	99.9179152	100.089256
99.7390495	99.6199264	99.9104779	99.963305
99.8197637	99.7633791	99.8719566	100.070408
99.8276453	99.7117306	99.8649007	100.071156
100.114305	99.8355209	99.9406084	100.066668
99.903251	99.9035087	100.175931	100.051411
99.7952429	99.8993547	99.530414	99.9659975
99.7259134	99.8590605	99.4768274	100.15971
99.8822332	99.7672562	100.025279	99.9767676
99.8415112	99.884954	99.8065467	100.057992
100.046289	99.9917128	100.171927	99.9522357
100.131236	100.061639	99.7153924	99.8508169
100.07621	99.9781429	99.9285943	99.8599416
99.8615073	99.9054472	99.9249711	100.04797
99.8562529	99.8852309	100.082489	99.9941195
99.9414917	99.9310638	99.8465936	100.167489
100.079859	100.085594	99.8338167	100.137273
99.7021224	99.9205402	99.8248539	100.100774
99.8242883	100.156074	100.231806	99.9772164
100.305654	100.421656	100.146564	100.219844
100.343457	100.362391	100.133787	100.053954
100.207425	100.294958	100.399431	100.149539
100.196624	100.23417	100.268993	99.843936
100.343165	100.208969	100.008497	100.092995
100.151524	100.108718	99.9890461	100.085815
100.033299	100.211323	100.100987	99.9912774
100.304195	100.102903	99.6562756	99.9894824
100.312076	100.304789	100.323533	100.032563
100.161887	100.03533	99.7998722	99.9477481
100.233697	100.121596	99.8296213	100.095987
100.160865	100.151228	100.325249	100.094491
100.099855	100.165352	100.155336	100.16719
100.220269	100.148874	99.8416354	99.9498423
100.166995	100.255079	100.066851	100.084619
100.079567	100.103041	100.043777	99.944906
100.283469	100.225308	99.7878582	99.960762
100.321709	100.204676	100.429943	100.046175
100.047748	100.17186	100.009832	100.247517
100.000312	100.107333	99.9169617	100.024635
99.7886748	100.094179	100.043395	99.9218699
100.41264	100.418609	99.7981559	100.191273
100.228881	100.283465	100.209304	99.8259858

99.9790275	99.9837855	99.7833399	99.8917209
100.021645	99.98558	99.7650269	100.04798
99.9007748	100.030581	100.049451	100.073999
99.702234	99.7885985	100.157803	100.037513
100.001282	100.011531	99.8495339	100.005663
99.7624508	99.7601625	100.302782	100.084466
99.6139452	99.6893485	99.93881	100.106447
99.9785911	99.8173106	99.6265346	100.005364
99.8484124	99.7216496	100.112593	100.027345
99.8030316	99.701634	100.267491	99.8782631
99.7543055	99.6290255	100.111639	99.9454024
99.7180882	99.5573833	99.9727654	99.8997956
99.8888478	99.7877703	100.129762	99.9625984
99.6601986	99.7133673	99.9958474	99.87288
99.9032474	99.9184931	99.9615105	99.9250662
100.127388	99.9952427	99.8319839	99.989514
100.315747	100.245645	100.107252	100.085662
100.295238	100.381061	100.43021	100.141437
100.0055	100.150813	99.8010807	100.189436
100.062953	100.179111	100.012825	100.007308
100.024554	100.172761	99.6988329	99.9225242
100.051898	100.202301	99.9912692	100.061139
100.075025	100.175522	99.9439605	99.9974391
99.9813547	100.007252	100.051359	100.039756
100.133497	100.152193	99.8728067	100.059644
100.211167	100.147638	100.280463	100.241174
99.9436829	99.9716381	100.065475	100.069363
100.175968	100.248268	99.5805612	100.055307
100.10877	100.058188	100.090656	100.081924
100.046517	100.148052	100.078065	99.9053282
100.040262	99.9091065	99.7974562	100.004018
100.175968	100.126794	99.9758176	100.03871
100.087679	100.126932	100.302972	100.029439
99.9876091	100.100704	100.000998	99.9796449
100.243167	100.090075	99.9197339	99.9628975
100.250439	99.8645199	100.168867	99.9929532
100.334801	100.066471	100.249559	100.019121
100.311383	100.180215	100.486866	100.097176
100.055244	100.17566	100.24231	100.000579
99.7307424	99.9408554	100.085505	99.9516827
99.9422283	99.9771596	100.107061	100.015981
100.149642	99.9938624	100.091419	100.045289
100.253203	100.185184	99.7324068	100.052616
100.339164	100.236673	100.362871	99.9913083
100.160987	100.227148	100.046399	100.06413
100.131897	100.117269	100.402549	100.014785

99.8761935	99.9474813	99.8249258	99.8127687
99.9899363	100.00532	99.7406095	99.8707866
99.8994657	100.01236	99.7697959	99.9959438
99.9649187	100.088281	99.9283181	100.063083
100.175532	100.26994	99.8420942	100.317883
100.115752	100.030443	100.183175	100.165362
100.096261	100.062744	100.385953	99.9823365
99.9268105	100.05598	100.197291	100.033626
99.8840479	99.9612852	99.9157279	99.870039
99.8801207	99.7456684	99.8188214	100.000878
100.190223	100.018433	100.022745	99.9175897
99.9580825	99.967911	100.216367	99.9857757
99.8770662	100.011117	99.9454866	99.9687292
99.801868	99.8715599	100.200152	99.9942989
99.9074655	99.8745967	99.8426665	100.105699
100.16855	99.9901353	99.9353763	100.106746
100.051898	100.071578	100.162	100.044242
99.9506645	100.006148	99.9792513	99.9642433
99.8609212	100.036102	99.8815817	99.9247672
99.7519783	99.8634156	99.8459095	100.021813
99.8185949	99.7308982	100.036098	99.9471968
99.810886	99.8486454	99.7459508	99.9820374
100.014808	99.9085543	99.7810508	100.071307
100.085498	99.9750891	100.115073	99.9288045
99.9028111	99.9191833	99.8998948	99.9486921
99.779614	99.9300884	100.087031	99.8425253
99.9432465	99.8675568	100.020646	100.013738
99.9912454	99.8268353	100.003859	100.008654
100.088261	100.091732	99.7615932	100.180764
100.012627	100.168482	99.9605567	99.9373278
99.9172108	99.8678328	100.060897	99.8828986
99.7746687	99.9461009	100.11946	99.9871215
99.8113223	99.8411913	99.9224045	99.8977021
100.037935	99.886192	99.6458014	99.9189355
99.8807025	100.01236	99.9565507	99.9836823
100.111388	100.107192	100.295723	100.261062
99.8104496	100.062468	99.9281274	100.069363
99.6274721	99.8839834	99.9342317	99.8683941
99.9467373	99.9702577	99.7152383	100.020616
100.243458	100.237087	99.7652177	99.9724675
99.9752458	100.024507	100.436696	100.037214
99.882157	99.6861736	100.141398	99.9980372
99.9474646	99.7580919	99.9153464	100.002673
100.070807	100.037207	99.8853969	100.041999
100.220913	100.232117	100.260433	100.007458
99.9413556	100.038035	99.7352682	100.058149

99.87183	99.8019883	99.766553	99.9621499
100.079534	100.066885	99.9000855	99.9931027
100.153714	100.187669	100.290764	100.005215
99.9825183	100.159095	99.9059991	99.8332544
99.8703755	99.9574201	99.5988743	99.8936648
99.9527008	99.7687209	99.8367529	99.9890654
100.150223	100.082345	100.148647	99.724097
100.002154	100.142806	100.064903	99.9473463
100.039971	99.9944145	99.7926872	99.9062254
100.061338	99.9999721	99.7472531	99.8699485
100.057558	100.001903	100.054562	99.9140206
99.8149065	100.03376	100.05399	99.8654666
99.722731	99.773384	100.101811	99.9058037
99.8569235	99.6612622	99.4372772	99.9554035
99.9780311	99.8952975	99.63618	100.12706
100.060029	100.048103	99.9019552	100.043548
99.9854458	100.059274	99.3829791	99.9326952
99.9027204	99.8393056	99.7453479	100.028309
99.8215943	99.9987309	99.9812115	100.096733
99.7769605	99.8260661	99.7356314	100.221031
99.6567252	99.8035866	99.9707329	100.22133
99.8922526	99.8562686	100.425885	100.064762
99.6874019	99.734493	100.372349	100.072531
99.5181711	99.5426585	99.9922617	99.9418084
99.5796699	99.4543954	100.155347	99.8919098
99.8183958	99.8657845	100.172493	99.882946
99.686675	99.5598974	99.5845491	100.204896
99.4991254	99.2614576	99.7613516	99.9004255
99.8790223	99.7311832	99.7141026	99.9325458
99.682168	99.6256811	100.160872	99.8503775
99.8605581	99.616441	99.9754959	99.8334957
99.8407855	99.6749154	100.162015	100.093894
100.134177	99.9899046	99.9539672	100.041755
100.151042	99.8559928	100.323576	100.041755
99.7592232	99.6913268	100.009789	100.078506
99.7544254	99.5705166	99.9210072	99.9455433
99.8234844	99.7655231	99.6725693	99.9643673
99.8538703	99.9914216	100.504951	100.01083
100.027899	100.119127	100.009599	100.038767
99.9691624	99.83241	99.9069087	100.053109
100.07704	99.7946223	99.7684008	100.069094
99.9376134	99.900814	100.205644	99.8234861
99.6980152	99.8609576	99.8480381	99.9452445
99.9880628	99.8467528	99.8066953	99.848286
100.036622	100.005075	99.7821182	100.066106
100.076313	100.067273	100.065993	99.9379241

99.9809388	100.018728	100.019887	100.066853
99.9738148	99.9234314	99.8358448	99.8499294
100.073405	100.030451	99.8236516	99.9905117
100.016849	99.9293616	99.9562534	99.8010766
99.743376	99.7794521	99.9251987	99.9833407
100.073405	99.8984695	100.008456	100.108685
100.027754	99.9158463	100.289854	99.8636739
99.8497995	99.6837417	99.9615879	100.001567
100.105826	99.8697839	99.9749243	99.9573456
100.168052	99.9751481	99.8562305	99.90177
100.090125	99.9694937	100.056276	100.036974
100.289451	100.221182	99.803647	99.9961888
100.072242	100.007695	100.164682	99.9833407
100.10917	100.018452	99.9194831	99.9404638
100.010452	99.8297897	100.048084	99.9168591
100.325943	100.237455	99.7722112	99.9930515
100.13534	100.185463	100.005598	100.069543
100.336556	100.263107	99.6740935	100.107938
100.365634	100.175671	100.157061	100.029355
100.269678	100.170706	99.9090045	100.079702
100.042147	99.8990211	99.8198412	100.066704
100.146244	100.189048	99.861565	100.084183
100.181719	100.29193	99.6544699	100.126015
99.8737884	99.7093932	99.813935	99.9961888
100.15962	100.180774	99.7350598	99.9824443
100.150751	100.092511	100.265467	99.9742275
100.083146	100.092235	99.9467274	100.090906
100.31751	100.244213	99.9899754	100.068347
100.147407	100.113887	100.107145	100.016357
100.166744	100.251108	99.6788565	99.9836395
100.271423	100.125747	99.8941439	100.109432
100.163836	100.144227	100.053037	99.8819003
100.230714	100.237455	99.9034794	99.9655625
99.9765772	99.9143293	100.17497	99.8151199
100.484706	100.480317	100.754722	99.8665124
100.538935	100.638501	100.626502	100.042502
100.290759	100.691459	100.351201	100.03548
100.602179	101.067819	99.4927185	100.275411
100.432948	100.903153	100.170398	100.35743
100.225626	100.693114	100.082759	100.183084
99.869136	100.376194	100.037796	99.9863286
99.7720173	100.166155	100.299189	99.6725954
99.7869922	99.8391677	100.020649	99.627627
99.6983059	99.576171	100.343009	99.8941508
99.8797493	99.7209777	99.985784	99.8950472
99.9988214	99.9064683	100.235746	99.8965411

99.8365692	99.8248249	100.099143	100.025769
99.675044	99.3963347	100.480374	99.9234326
99.9636377	99.6830522	99.9937858	100.038916
99.9395034	99.8125508	100.092285	99.9697456
100.122982	100.109612	99.8964302	100.049374
99.9605846	99.9471521	100.344342	99.9923045
100.060611	100.169189	100.14201	100.023827
99.9800665	100.171396	100.36549	100.012921
99.8197043	100.108922	99.8756635	100.020391
100.031243	100.07734	100.100667	100.065509
100.150315	100.3362	100.030937	100.060579
100.02281	100.066859	100.082568	100.017851
99.9203123	100.216217	99.7964072	100.01561
99.9736694	100.17967	99.7805941	100.004704
99.9140607	100.224767	99.7158173	100.097928
99.8910895	100.113611	99.990928	99.9790082
99.7935346	100.123541	99.7775457	100.052213
99.6982539	99.5600197	100.402653	99.8834955
99.6441482	99.5389567	100.356113	99.8233761
99.5344246	99.66488	100.256687	99.9476552
99.7111922	99.554413	100.636121	99.9216357
99.7153929	99.5779005	100.461308	100.123003
99.9316475	99.8573259	100.223417	99.9904821
99.7489989	99.8329293	100.358805	99.8608699
99.5041792	99.6901859	100.884012	99.7243084
99.4549464	99.4243983	100.559195	99.6131199
99.810834	99.6488176	100.258033	99.8765462
99.8032726	99.9043009	100.629774	100.165669
99.8810705	99.7726194	100.443231	100.086479
100.222507	99.9853707	99.9230236	100.06353
99.8970334	99.8980881	100.894782	99.6734009
99.5992842	99.4725856	100.779009	99.5890398
99.7145528	99.5772944	100.097836	100.037673
99.9123241	99.9426386	100.593043	99.939413
99.7948711	99.7658005	100.341305	100.068056
100.082202	100.243885	99.7999432	100.274756
99.8580504	100.014313	100.355728	99.8340425
99.6085258	99.584871	100.200339	99.6452805
99.8693084	99.8264134	99.6722473	99.9573518
99.8104979	99.9724904	100.164184	100.039612
99.8259567	99.8292925	100.098413	99.8855964
99.7842852	99.8130785	99.9147541	99.6653203
99.6051652	99.9459723	100.60112	99.5064565
99.346231	99.6627585	100.315342	99.5622124
99.6520456	99.6556365	99.8662912	99.7727918
99.5465228	99.7579208	100.06072	100.007128

99.36337	99.6048732	100.174377	100.029107
99.7127044	99.7071575	100.033604	99.8999798
99.6817869	99.6994293	100.465154	99.7496814
99.2775067	99.5759306	99.8978306	99.8114169
99.6856516	99.8173214	99.9626401	99.744025
99.536945	99.5386536	100.388998	99.5365162
99.5725674	99.6142682	99.7397492	99.6738857
100.136476	99.9143021	99.925716	99.8196591
99.8093217	100.025375	100.035334	100.128821
100.039691	100.286617	100.060912	100.212213
99.9760075	99.9862799	100.841511	99.7533985
99.8269648	99.7848935	100.236109	99.9211509
100.176299	100.065683	99.96014	100.231444
100.478417	100.316014	99.8622526	100.263605
100.4475	100.247825	99.9407164	99.8670112
100.057502	100.151147	99.579937	99.8526278
100.164705	100.100232	99.8116743	100.140296
100.231581	100.133266	100.264572	100.025229
99.9492907	99.8905115	100.761317	99.5232641
99.7752116	99.7756501	100.211493	99.6656436
100.383648	100.128569	100.12726	99.7500046
99.9926424	100.050075	99.804751	100.022643
99.9803762	99.8802073	100.413422	99.542819
99.6849795	99.7062483	100.380152	99.7587316
100.437082	100.263735	100.143991	99.9377969
100.271908	100.232217	99.8930227	100.128983
100.183693	100.278434	99.8070588	100.468205
100.037002	100.000372	100.337458	99.9565438
99.9592045	100.002948	100.135337	100.021835
100.687951	100.433148	100.218417	100.043006
100.156808	100.218124	100.141107	100.181668
100.308875	100.507096	100.115144	100.219808
100.204528	100.252371	100.840742	99.7331971
100.083379	100.090837	100.254956	99.8603851
100.604608	100.392689	99.7772502	99.8636173
100.032802	100.248431	99.8287902	99.9542812
99.9639093	100.056288	100.282457	99.6961881
100.038515	99.843385	100.205147	99.7031374
100.347186	100.151147	99.7176332	99.9995324
99.9731509	100.042953	99.7259026	100.031693
99.9447539	99.9159689	99.8730222	99.7222075
100.352563	99.9643077	99.5926296	99.94814
100.370206	100.05553	99.6799398	100.401136
100.334752	100.368747	100.32265	100.324856
100.064055	99.9629439	100.101875	100.101832
100.540924	100.141449	99.6993634	100.219324

100.276949	100.218579	99.6705164	100.203324
100.253929	100.253734	100.051873	99.882041
100.000372	100.046893	100.09899	99.8330728
100.263339	100.20388	99.7643653	99.8755765
99.6735534	99.9170296	99.6162841	100.029269
100.003396	100.035982	99.7305181	99.9785229
100.405492	100.048257	99.5514746	100.202516
100.414062	100.241157	99.2095418	100.42958
100.264179	100.234641	99.5282047	100.128983
100.113792	99.92597	99.5526285	99.8375979
100.308707	100.179332	99.5768599	100.255201
100.233261	100.092656	99.9932179	100.408732
100.349706	100.042044	99.4439716	100.236131
100.528826	100.490276	99.754365	100.54497
99.7085037	99.728372	99.4770494	100.121387
100.172603	100.104475	99.1689638	100.426186
100.017847	100.339956	100.350151	100.493255
99.7511833	99.96552	99.7403261	100.124781
100.151263	100.100384	99.147617	100.341178
100.210409	100.272676	100.660929	100.162921
99.9657576	99.932789	99.1405014	100.347158
100.135132	100.349806	98.4720209	100.780761
100.039355	100.508612	99.4814726	100.284938
99.7489989	100.09508	99.2699282	100.189426
100.143337	100.254747	99.3175414	100.652426
100.384018	100.319143	99.4188552	100.469051
100.435256	100.547567	100.367275	100.033899
99.8266421	99.9403593	99.7869362	100.096641
100.4452	100.521748	99.1316711	100.718563
100.410985	100.675145	100.003817	100.10602
100.015412	100.094819	100.348977	99.651464
99.518543	99.7003923	100.675839	99.843571
99.442361	99.4862445	100.816639	99.8836741
99.704616	99.628554	100.165803	100.20272
99.852598	99.7972904	99.3647313	100.292306
99.5797246	99.6561958	99.5170872	100.062198
99.8559689	99.749297	99.6289946	100.292791
100.255756	100.189591	99.3233198	100.507375
100.485651	100.2227	99.5190133	100.114267
100.309016	100.319143	100.441623	100.323677
100.369692	100.296817	99.6355434	100.530499
100.475369	100.592979	99.4785648	100.507213
100.451436	100.393563	100.288305	99.8227109
100.725489	100.215714	99.240882	100.187197
100.521045	100.338128	100.196044	100.026299
99.913274	100.113045	99.8508833	99.5636575

99.9018129	99.8747481	99.5184355	99.9852256
100.521888	100.278743	101.120965	99.8485839
99.9166448	100.063988	99.8741893	99.997677
100.009513	100.243811	99.5299922	99.7832545
99.4560131	100.045611	99.983978	99.5799898
99.9914785	99.9923016	99.3368026	100.003337
100.365141	100.473147	100.081054	100.157604
99.8023718	100.135522	99.4760609	99.8773676
100.494415	100.579613	99.2724703	100.071253
100.523067	100.497599	100.352444	99.9611314
100.101538	100.224219	99.4427391	100.163911
100.650319	100.616216	99.3450849	100.294408
99.7941131	100.068241	100.66775	99.5584829
99.8859698	99.8001761	100.611892	99.6731326
100.089403	100.098768	100.682581	100.364588
99.7135489	99.9181852	100.044265	99.8692822
99.9727701	99.8820382	99.6694431	99.7586752
99.4453948	99.5043179	100.58146	99.5594531
99.6087143	99.3911689	99.9053924	99.9412415
100.15918	99.8542446	99.5351927	100.170379
99.8989477	99.7369949	99.5143906	99.6289868
100.356546	99.9520539	98.5831129	100.231019
100.130696	100.138712	100.062178	100.319796
100.405592	100.233787	99.6087704	100.15906
100.029064	99.9637485	100.296972	99.860065
99.9945123	99.7647885	100.008247	100.005601
100.385704	100.163316	99.438309	100.174422
100.417053	100.293779	99.9462261	99.6920522
100.442335	100.137345	99.9957273	99.8094509
100.120584	100.042877	100.709739	99.9279816
99.6877616	99.5514001	100.313729	99.6605195
100.079796	99.8237171	99.8038861	100.128659
99.6618057	99.855004	100.441623	100.122352
99.5938824	99.752942	100.546212	99.8817336
99.7430441	99.8258434	100.349555	99.9538546
99.763438	99.81263	100.898306	99.8159192
99.6739409	99.8665467	101.171622	99.5579978
99.4440464	99.5292259	100.622293	99.6889798
99.9176561	99.897226	100.237648	100.022741
100.024513	100.122157	100.165996	100.244279
100.002265	100.040143	100.593594	100.252364
99.8455191	100.015691	100.42217	100.266109
100.117044	100.231357	100.355526	100.067534
99.5426449	99.8436131	100.698568	99.8383964
99.5665782	99.6369073	100.75481	99.869444
99.9891189	99.9251716	100.314115	99.9967068

99.790068	99.8666986	100.744794	100.2501
99.8556318	99.8932772	100.325286	100.301846
100.232666	100.183668	99.6301503	100.373482
99.8200689	99.9402075	100.313537	100.001558
99.6958517	99.7192251	100.360919	100.015303
100.119572	100.027234	99.4885807	100.437033
100.233171	100.098616	99.9558567	100.1382
100.086538	100.013413	99.9312024	99.7014312
100.244127	100.010375	99.5648549	100.152268
100.284072	100.24533	99.7148994	100.088394
100.491718	100.415282	99.1561328	100.087424
100.070189	100.130662	99.4001719	99.9609697
99.9326566	99.9771138	99.3152301	99.8556989
100.641554	100.506104	99.3427736	100.261258
99.9321509	100.208576	99.8622474	100.022256
99.5542744	99.8756594	100.102049	99.673456
99.7802924	99.9210709	100.351289	99.6116842
99.383539	99.7061636	100.667172	99.6498469
99.2908396	99.5144937	100.971498	99.5895305
99.3392118	99.292752	100.283874	99.596969
99.773045	99.5422874	99.8075457	100.132217
99.7187737	99.7699524	100.196236	100.152106
99.6784916	99.7456519	99.9677986	100.071415
99.8178778	99.8102	99.7019944	100.020801
99.5625331	99.7588653	100.423325	99.7184103
99.8982735	99.8680655	99.8799677	100.186065
100.343736	100.376249	99.2343332	100.213878
100.31778	100.31565	99.477987	99.7463855
100.457335	100.206146	99.2634175	100.065108
100.188844	100.066874	99.5534908	100.255275
100.508236	100.366832	99.4670081	100.069313
100.258958	100.228016	100.515586	99.6451574
99.2033467	99.3402685	99.6698479	99.7757392
99.9935345	100.013383	98.9454589	100.244792
99.8157213	99.9390126	100.408682	99.4375169
99.4451794	99.336943	100.258065	99.2277028
99.6871796	99.5266486	101.191816	99.2884342
99.1411708	99.1333307	99.8329847	99.5820771
99.7404733	99.6970057	98.7397563	99.9915296
99.7019276	100.044673	99.3194986	99.581431
99.6067364	99.7518767	99.4273577	99.7476348
99.8088501	100.024569	99.4331358	100.12866
99.9804625	100.163787	99.1030101	100.052261
100.007612	100.248588	98.5527364	99.83534
99.7992975	100.234832	99.5192305	99.5916067
100.115875	100.468979	99.9448885	99.8136964

99.010618	99.635786	99.8849882	99.9984749
99.3333967	99.6085772	99.9310209	99.8902567
100.093921	99.9514077	99.4496999	100.149334
98.9755916	99.2740605	100.654062	99.6491078
99.1766999	99.3443499	101.167355	99.6812502
98.6700747	98.9806593	101.038695	99.3603105
98.9090583	99.0819364	100.589539	99.5875687
99.6437738	99.6691923	100.594739	99.5754548
99.0974297	99.4179647	100.593391	99.7910836
99.7004193	99.9063621	100.784841	100.522283
99.2514451	99.1062731	100.752868	99.3535267
99.8820871	99.6460649	100.3405	100.19569
100.359216	100.270355	100.342041	100.041601
99.633048	99.8174801	100.088187	100.48045
100.551107	100.390678	100.874402	100.218142
100.713837	100.239367	101.316432	99.3620872
99.8504125	100.00386	101.356494	99.9847457
100.258327	100.535338	100.975135	100.42521
100.05504	100.373295	100.465501	100.453476
100.192129	100.447514	100.048895	100.596905
100.122747	100.379795	99.9310209	100.539081
100.239054	100.568291	99.8308661	100.449599
100.355027	100.346842	99.822584	100.525514
100.432286	100.438596	100.21184	100.58043
100.455078	100.387957	100.340693	100.471082
100.342457	100.245262	100.392696	100.702216
100.464128	100.150939	101.061422	100.532298
99.7357808	99.5138	100.861113	100.07019
100.422901	100.093347	100.525786	100.512754
100.167326	99.7680508	100.136338	100.053715
100.042638	99.5608107	99.8566752	100.3094
99.7573999	99.5130442	99.8967371	99.9879761
99.7367864	99.5337531	99.595117	99.9706935
100.433962	100.086393	99.7563278	100.081658
100.478038	100.058278	99.7584464	99.950019
100.258997	99.9683376	99.5565959	100.123814
100.237211	99.9356871	99.1213076	100.075035
100.391059	99.9520123	99.3204617	100.053876
100.491948	100.138241	99.6051325	99.929183
100.371618	100.070673	99.9109899	99.7967368
100.674454	100.211251	100.076631	99.9057626
100.711156	100.472909	100.153865	99.9265986
100.19481	100.056313	99.9728163	99.9311212
100.061409	99.6977615	100.379599	99.2008905
99.7015924	99.5589968	99.8221988	99.4352556
100.636913	100.160613	99.1363309	99.8379243

100.116378	100.192961	99.4849467	100.111216
100.441168	100.494827	98.8612903	101.000382
100.405806	100.273529	99.467227	100.055976
99.5207626	100.005523	99.7243553	99.6986943
100.261511	100.643266	99.3574419	100.049031
100.104144	100.649464	99.5829829	100.360925
100.286817	100.276855	100.10321	100.639547
99.8666688	99.6156817	99.792345	99.7542571
100.137159	100.07687	99.5552477	100.148042
99.7124858	99.845747	100.085298	99.9682707
99.8861092	99.9275244	100.957993	99.8503613
99.3362458	99.2488168	101.090506	99.1876459
99.6655605	99.7393305	100.117078	99.5307138
100.436811	100.415771	100.603406	100.024803
99.6596949	100.219112	100.324706	100.85566
100.697078	100.748171	100.394622	100.456222
99.6464552	99.7914806	99.6295934	99.3943912
99.6888556	99.870386	99.8641868	99.1813466
99.8314748	99.896839	100.076631	99.5124621
100.304247	100.134009	99.7482383	99.5245761
100.480552	100.298168	99.5535142	99.9482423
100.586971	100.322807	99.4924583	99.9978288
100.11068	100.387806	99.5955022	100.133344
99.8341563	100.125846	99.6607955	100.172593
100.066436	99.8718976	99.6191927	100.261106
99.8869472	99.8765836	99.1952681	100.384346
100.496138	100.39748	98.871691	100.27322
99.8148834	99.514707	101.003641	98.8332716
100.020684	99.8256427	100.501711	99.3494887
100.077162	99.8362239	99.9656899	99.540728
99.7087988	99.5015561	100.33164	99.7251835
100.74702	100.793368	100.963386	100.00962
99.6402544	99.4622545	100.115152	99.7545801
99.7062849	99.5073001	99.5477361	99.5851459
100.470831	100.551814	98.7663358	100.798805
99.8554402	100.107102	99.1604065	100.556041
99.5041711	100.080498	99.6997018	100.34865
99.4600949	99.9037923	100.362072	99.880727
100.102803	100.241635	100.533491	100.093933
99.6590245	99.4646731	100.219929	99.4018211
100.729591	100.659894	99.6973905	100.634863
100.452429	100.426879	100.866638	100.031459
99.6152351	99.9242675	99.6796351	99.7888967
99.7541779	100.108609	99.3495491	100.570109
100.03779	100.179521	99.6805958	100.444139
100.785049	100.689421	99.4406206	100.538091

99.8134601	100.108609	98.7205028	100.272728
99.653297	99.9317079	99.1447344	100.040353
100.201153	100.263644	99.6590768	100.118135
100.149954	100.478506	99.9194182	100.470012
100.120313	100.113164	100.875669	99.8008631
99.8338384	99.8888873	100.151708	99.6409336
100.442155	100.129412	99.3727973	100.421338
100.006464	100.047567	100.210885	100.619754
100.193574	100.114835	99.6160388	100.574314
100.388767	100.099802	99.2544429	100.045043
100.117619	100.086287	99.8986678	100.137863
99.9433085	100.090843	99.4235207	100.517231
100.421272	100.397876	100.11136	100.63738
99.8104287	100.084313	99.5282336	100.231816
100.262961	100.247396	99.1107267	100.00122
100.099093	100.057285	100.581127	99.7977907
99.9845703	99.6605105	100.036043	99.9247317
100.216142	99.9690621	99.3843254	100.077384
100.145744	99.8648956	99.6427454	99.5456874
100.295465	99.7693843	99.9753292	99.8996669
100.584635	100.377832	99.7718594	100.35245
100.728967	100.505231	99.5320763	99.9239231
99.9889491	100.017197	100.342497	99.5950085
100.192395	100.081884	99.6477409	99.9176165
100.581435	100.50265	99.221588	100.196563
99.8272702	100.36781	99.4940338	99.7840454
100.77225	100.60378	101.209789	99.1021216
99.440083	99.5930907	100.241435	99.4745358
99.8961521	99.8820542	100.530596	99.8383794
99.0572746	99.013949	100.821295	99.7036764
99.5212593	99.2306337	100.416276	100.093232
99.4153259	99.529619	100.134416	100.263996
99.5170489	99.5873206	100.862796	99.8482436
99.3678328	99.2221303	100.636654	99.8170339
99.6575074	99.4983387	100.076584	99.8076549
99.7691669	99.6785802	99.9630326	100.317683
100.065241	100.084465	100.008953	100.217262
100.094209	100.241778	99.9086587	100.066712
99.9296668	99.8946575	100.201663	100.057979
100.269192	100.173144	99.663688	100.352612
100.32578	100.469244	99.6000917	100.408563
100.194248	100.352322	100.162083	99.9425196
99.999054	100.129108	100.728494	99.4945876
99.6915273	100.002924	101.501833	99.537602
99.5172173	99.5974943	99.7935705	99.2093342
100.648633	100.304491	99.8337265	100.285179

99.7855032	99.9663289	99.9188418	100.210955
100.024653	99.7456962	99.7082631	99.7559082
100.026506	99.6677991	100.119814	99.426185
100.410493	99.992902	100.158433	100.195431
101.960252	101.23789	100.907371	99.3708807
100.114082	100.193035	100.667395	100.68088
100.049915	99.8569996	100.373238	100.256881
100.073662	99.8589736	100.770956	99.8616654
99.9219197	99.9739212	101.327375	99.3378923
99.6718227	99.5249118	100.562682	99.6658367
100.257067	100.343211	100.291197	100.594366
99.8523641	100.119542	99.9778269	99.9797125
100.121492	100.372062	99.0940111	100.230522
100.117787	100.509331	101.352737	99.9328171
99.2906985	99.6749359	100.029895	99.8052292
100.073999	100.11696	99.0158126	100.640453
100.27374	100.472281	99.6763689	100.508984
100.37782	100.436141	99.827194	99.9860191
100.157196	100.302668	99.8227749	100.38269
100.527205	100.507965	99.7090316	100.323019
100.146249	100.20412	100.106941	99.8320728
100.187848	100.072014	99.9272957	99.7413545
101.056703	100.638097	99.4121848	100.326577
100.828332	100.459829	101.811745	100.294559
99.5850887	99.3284227	100.611292	99.7974672
100.087641	99.8691473	99.9173048	100.041
99.7125793	99.9739212	100.078889	99.8852748
99.9456663	99.8178232	100.286394	99.8443626
99.4030316	99.7956537	99.6667622	100.354391
99.9887807	100.076114	99.063846	100.308627
99.7102214	99.9976092	99.1624107	100.237314
99.5748154	99.936567	99.2801888	99.905165
99.3930951	99.8422705	99.4571441	100.116679
100.066589	100.275032	98.7614273	100.342748
99.9473505	100.15477	99.2056409	100.464352
100.261614	100.166159	99.2997865	100.110211
99.3654749	99.7513146	99.906161	99.6486956
99.1120096	99.3149083	101.068187	99.1092367
99.6637388	99.4963647	100.543853	99.567518
99.3718747	99.4363854	101.138508	99.6220137
99.4451355	99.2786171	100.270639	99.7120853
99.6192771	99.4875576	99.5040248	99.835307
99.5505636	99.4426111	100.464694	99.6097239
100.204858	100.09555	99.7076867	100.171822
99.8491642	100.074595	99.9338283	100.053451
99.7972923	99.8781062	99.7964525	99.7358564

99.8075656	100.009453	99.7234417	99.8524481
99.9283195	100.004139	99.3253403	100.113607
99.9625078	100.012186	99.0544315	100.109726
100.452345	100.120645	100.237022	100.168691
100.375948	99.9844381	99.9073322	100.136001
100.215935	99.9627798	100.048208	99.9320742
99.9126321	99.6881202	100.043685	99.938128
100.443192	99.9663093	99.6239979	100.182528
100.16911	100.041873	99.5903053	100.220927
100.004169	99.6563546	99.7981139	99.8142846
99.8994297	99.4603067	100.161044	99.8533749
100.138481	99.6067811	100.09479	99.9002485
100.32349	99.7378541	99.2612938	100.262611
100.130031	99.6712748	99.8428866	99.9758345
100.086904	99.4375254	99.7271108	100.16748
99.9464302	99.3194474	99.8722828	100.206571
99.9379806	99.2923344	99.9435121	100.017865
100.10345	99.455975	99.8288669	99.9440088
100.304655	99.8658788	99.3494825	100.347365
100.017899	99.7559829	100.270036	100.02634
100.227729	99.6606863	99.8413037	100.298588
100.015787	99.6016473	99.4184505	100.127698
100.331412	99.5493465	99.5301561	99.5904672
99.9654416	99.473783	100.345788	99.7291856
100.626089	99.995187	99.3763913	100.115764
99.6976973	99.3627641	99.9279095	99.9555975
99.9643854	99.8090859	100.01655	99.8154954
100.167878	99.5849624	99.9417031	99.872574
99.735016	99.2241508	99.8297714	99.9274041
100.190234	100.033049	100.035997	99.9547327
100.124926	99.6751252	100.033284	99.9837909
100.239347	99.7959305	99.8433389	99.94816
99.9339319	99.7866254	99.6389221	99.9860395
100.471181	100.623599	100.388978	100.032913
99.7652935	100.358566	100.413399	99.4500191
99.9223138	100.500869	100.811379	99.8639258
100.600916	100.71681	100.454328	100.15987
100.19129	100.749538	100.743089	100.09103
100.328067	100.91719	100.447544	100.073733
99.5651454	100.067702	100.359808	99.5333885
100.551979	101.009117	100.043911	100.141708
100.336693	100.71312	100.054313	99.9097616
100.050817	100.408139	99.4614142	100.279562
99.9411492	100.49381	100.493674	99.8512993
100.063139	100.56809	100.568973	100.069063
99.8691522	100.067542	100.475131	100.027551

99.8772497	100.07043	99.5183976	100.205706
100.015787	100.249151	99.7411305	99.862888
99.7156525	100.030161	100.055218	99.5894294
100.357816	100.488836	100.131648	100.248082
99.4790659	99.8121341	100.3632	100.088089
99.85683	100.319741	99.5805819	100.183047
99.541029	99.9343833	100.283378	99.6743555
100.269448	100.581085	100.057027	100.049691
100.100986	100.2007	100.067202	100.042426
99.8636952	100.033851	99.4957852	100.041907
100.237411	100.481617	99.8926341	99.958019
100.37542	100.547715	100.060419	100.269876
99.8802422	100.029519	99.8268318	99.9888069
100.239699	100.281879	99.305162	100.133925
99.7878256	100.152731	100.243806	99.7504604
99.9054148	100.296799	99.7834158	99.6688206
100.334052	100.480975	99.863464	100.291151
99.7123079	99.6723978	99.8465046	100.062317
100.093593	100.298243	99.9934857	100.272816
99.3806641	99.660205	100.240866	99.5304481
99.9615689	100.216102	100.251946	100.184604
100.152387	100.373807	99.6988452	100.051593
99.7733909	100.143426	99.7752753	100.160908
99.5781717	99.6704726	99.7341206	99.5880457
99.8519011	99.8501565	100.291292	99.7615301
100.072293	100.471831	100.004566	100.434366
99.6258763	99.7765182	99.7990184	100.134617
99.906471	99.8560925	100.137301	99.8179169
99.8453879	99.9866841	100.489377	99.8621961
100.317153	100.376053	100.284282	100.037237
99.6005278	100.00369	100.033962	99.9540408
100.108731	100.098024	100.235891	100.048653
99.6466481	99.9939036	100.488247	99.9647647
100.388798	100.610444	100.230691	100.242893
100.13232	100.106046	100.088006	99.6392435
100.095001	100.049734	99.9267789	100.208819
99.7630051	99.9281265	100.387169	99.9234259
100.122638	100.302093	100.00547	100.086532
99.7615968	99.4765103	99.9699687	99.661902
100.017371	100.004332	99.2902378	100.310004
99.8158146	100.004332	100.359582	99.8049445
100.121758	100.152892	100.006601	100.179415
99.6980494	99.4920723	99.9355978	99.928269
100.238467	99.7752348	99.636887	100.450106
100.114364	100.118399	100.364782	100.015617
100.090248	99.9441697	100.186596	100.033778

100.299902	99.9631006	100.137979	100.030492
99.8195113	99.6443222	99.2034059	100.051593
99.5394447	99.5960322	99.8119075	99.6525618
100.304479	100.233429	99.863464	100.42001
99.7976833	99.7129871	99.9118547	100.042772
99.7996197	99.7532556	100.109714	100.186507
99.7774397	99.7636836	100.283378	99.6179687
100.357816	100.372523	100.109714	100.357915
99.507407	99.5602559	100.185918	99.9863854
99.7454019	99.529132	99.7372864	100.26313
99.4693002	99.8173567	99.9844154	99.6237178
100.584068	100.695484	99.8971779	99.9161878
100.130204	100.369769	99.7751357	99.6361633
99.9787979	100.081085	99.1513645	100.006245
99.6552123	100.216008	100.059675	99.9339918
100.261891	100.481185	100.006564	99.9642414
99.6604939	99.903495	99.4853985	100.05378
99.8483426	100.055485	100.339016	99.8135122
99.6157764	100.012818	99.8099403	99.9415974
100.056613	100.00026	100.150754	100.018518
100.046226	100.032944	99.2487723	99.8484288
99.4710608	99.6156161	100.209063	99.579467
99.9581997	100.31873	99.7401051	100.307012
100.092352	100.335796	99.8603392	99.7454075
100.022811	100.375887	99.0141801	99.9381404
99.753626	100.353507	100.035944	99.540229
100.374389	100.592279	99.8011262	99.9037423
100.406607	100.512259	100.085665	99.9609572
100.048515	100.128099	99.6273291	100.028025
99.7143661	99.7373367	99.9193263	99.8807526
100.407311	100.391988	99.2923911	100.170976
99.7965829	99.9426195	99.8732215	100.197249
100.011896	100.311485	99.8164944	99.8007209
100.083902	100.1091	99.8201105	99.8250934
99.999044	99.8350674	99.8115223	100.213671
99.6830287	99.6983732	100.338112	99.9953552
99.7374291	99.5673142	101.282583	100.187397
99.7506331	99.5562048	100.209063	99.8710728
100.099922	100.112481	100.537899	99.8997666
100.481253	100.50759	100.088829	100.054644
100.498155	100.559917	100.185785	100.125342
100.256434	100.601296	100.375176	99.8415146
99.6020443	99.7086776	100.4649	99.793461
99.7824987	99.6454022	99.566986	100.07193
99.8479904	99.7294474	100.406365	99.8902596
100.406959	99.9327981	100.193243	100.216782

100.110838	99.8413466	100.752151	99.8428975
100.037424	99.5718224	99.8983079	99.9946638
99.8999261	99.5335029	99.9175182	99.9438446
100.162421	99.8920636	99.4881106	100.189471
100.245166	99.981905	99.6293631	100.03079
100.01084	99.8574473	100.229404	99.6633015
100.07105	99.9479327	100.140584	99.8740113
100.064008	99.9804559	99.8820356	99.8947538
100.262772	100.009115	99.8180765	99.7955353
100.035839	100.041638	100.379019	99.8605286
100.496394	100.643318	99.6700438	100.182902
100.156788	100.183002	100.206125	100.248069
100.262419	100.141462	100.153466	99.7295048
99.7291546	99.9583981	100.47055	99.6980453
100.172632	99.8331353	100.040916	99.7317519
99.9861921	99.8687177	99.7299349	100.184977
99.8483426	100.159656	100.484562	99.7320977
100.050628	100.288139	99.7999961	100.321705
99.6023964	99.7544033	100.420377	100.156456
99.8479904	100.026665	100.256976	100.068991
99.9351367	100.165935	99.9294965	100.164407
99.9545026	100.125683	99.9950376	99.8878397
99.9953469	99.9261969	99.4476558	100.028543
99.6358465	99.8634045	100.13629	99.7329619
100.448155	100.32356	99.8054202	100.193619
100.004326	100.164647	99.8519771	100.379265
100.01084	100.230981	100.106232	99.6551774
99.7717595	99.8394146	100.664688	100.021456
99.6501068	99.6346148	100.23189	100.068646
99.5816221	99.429493	99.9970717	99.9680442
99.4724692	99.6460462	100.33088	99.6164579
100.394635	100.743786	100.035718	100.607952
99.6525715	99.8120435	100.03617	100.116699
99.7076761	99.8988258	100.316189	100.068818
99.3152538	99.4879382	100.473036	99.7794598
100.502556	100.240159	99.9530009	100.188088
99.8030969	99.7392688	100.075947	100.338644
99.7442952	99.8097894	99.8935618	99.9953552
100.121577	99.8321693	99.9154842	100.072967
99.9550307	99.928451	99.6869941	100.047557
100.168231	99.9345692	99.9306265	100.004689
100.393755	100.348838	99.9771833	100.203818
100.164006	99.9218497	99.8135564	99.8354647
99.9043274	99.8181618	99.7161486	99.9943181
99.9300312	99.8880384	100.181039	100.000714
100.350622	100.251751	100.099452	100.139862

100.129675	100.267208	99.7966061	100.321878
100.005382	99.9909213	100.024418	99.9901696
99.9883048	99.9393994	100.040464	100.03926
99.9066161	99.8569642	99.846101	100.259996
99.8856658	99.9691855	100.286809	99.9236206
100.370868	100.124717	99.9536789	100.235969
100.139006	100.065467	99.6081187	100.297159
100.250272	100.227922	100.566376	100.070374
100.272807	100.283147	100.159569	100.04168
100.348509	100.329517	99.9385366	99.9438446
99.6268677	99.4926074	99.3642603	100.043582
99.6240509	99.4608892	99.9907436	99.5537117
100.228265	100.085271	99.7446251	100.380475
99.7092606	99.6611808	99.9111901	100.261551
99.8782716	99.8065693	99.7674516	99.5884555
99.6691205	99.6645619	99.8698314	99.8952724
100.433367	100.028758	99.4743243	100.044273
100.058726	99.9451956	99.5843883	100.192582
100.172409	99.5687486	100.1421	100.130431
100.118713	99.2568264	99.928529	100.106072
99.9419566	99.3140495	99.7210603	100.168783
99.6926668	99.3406575	99.931693	99.7982167
100.449691	99.6559458	99.8797128	100.210072
100.019596	99.3342459	99.8573388	100.111427
100.008856	99.6410389	100.282898	99.9517987
100.262019	99.7181378	100.331489	100.139932
100.20445	99.5870215	99.933049	99.900835
100.087904	99.419199	99.8925949	99.7346417
99.6990047	99.290487	99.931241	99.7220304
100.598455	99.8216845	100.248546	100.015892
100.070651	99.6787068	99.9951993	100.158417
100.21202	99.6929725	100.522911	99.9201839
100.255857	99.64168	99.9940693	100.048716
100.156388	99.7481119	99.9479651	100.122829
99.9354426	99.560093	100.556359	100.209899
99.7579821	100.004574	100.656252	100.122484
100.390361	100.435591	100.522911	100.542113
99.693371	99.6386345	100.014861	99.7818047
100.199169	100.194837	100.030003	100.44105
99.8173117	100.010825	100.477259	99.8916788
100.066601	100.598284	100.510255	99.8695658
100.449163	100.499867	100.212612	100.140623
99.9317455	100.027495	99.8338347	99.9027354
100.2923	100.688206	100.349795	100.085859
99.5762964	99.9048744	100.491045	99.7405155
100.204274	100.13569	100.144812	100.076876

99.8664302	99.6923313	100.06865	100.10849
100.395819	100.347913	100.070232	100.135959
99.6990047	99.8420412	100.011697	99.9336591
100.306032	99.9691503	100.16809	100.087414
100.565005	100.328037	100.123568	100.001207
99.8317479	99.8636802	99.9425411	100.259654
100.031567	100.043364	100.059836	100.204717
99.4715454	99.6480916	100.290356	99.83726
100.315187	100.454024	99.7380103	100.314591
99.7997065	99.7875429	99.9757632	100.129221
99.8063964	99.9649828	100.063226	100.119892
99.4248916	99.4987023	100.504379	99.7569275
100.346348	100.284759	99.8024206	100.322883
99.5428465	99.6657234	99.9540671	100.318219
100.021004	100.02461	99.923783	99.9395328
99.5701345	99.6695703	100.097804	99.8279311
100.447402	100.373078	99.934405	100.220956
100.05639	100.127355	99.8790348	100.258099
99.7616792	99.9611358	99.9531631	99.9647555
99.842135	99.9133697	99.8358687	99.9511076
100.398636	100.214873	99.7945105	100.080158
100.086143	100.120142	99.9662712	100.427747
99.6711884	99.6078591	100.199504	99.7595189
99.493904	99.4987023	100.420081	99.5475447
100.470113	100.32964	100.052829	100.504106
100.033856	99.9973611	99.7875045	100.258617
100.225929	100.493455	99.8729328	99.8343231
99.4520036	99.8622376	100.397255	99.8217118
100.229802	100.227376	99.9703392	99.9469614
99.9824485	100.241321	99.5703177	100.080158
100.147761	100.116937	100.21442	100.032995
99.9072743	99.9322838	100.182102	99.9797855
99.9384355	99.8944557	100.1534	100.030058
100.116424	99.9045539	99.8532707	100.068583
100.18931	100.470694	100.222104	99.9977523
100.437895	100.225613	100.649472	100.275893
100.2923	100.073178	100.149106	99.9241574
99.7671368	99.659953	99.5219535	99.9839317
100.048644	99.9282766	100.019607	99.8148015
99.7419614	100.121745	100.297588	99.8562634
100.370115	100.696061	100.240862	100.259308
100.142304	100.345829	99.8919169	99.4998634
99.9379074	100.051539	99.4643233	100.01641
99.8287551	100.171755	100.015539	99.7541634
100.585779	100.748956	100.355897	99.9932606
100.129628	100.394397	100.220748	99.8897785

99.8468884	100.149636	99.5705437	100.031613
99.6842163	99.8734578	99.9506771	99.6909339
100.147585	100.372437	99.6941662	100.23685
100.326454	100.383818	99.8713508	99.8106553
99.6972441	100.038555	99.1777542	99.8381238
99.4988334	99.6496945	100.165604	99.8134194
100.350926	100.432706	100.05961	100.137686
100.037729	100.234108	99.8677348	100.103308
100.164839	100.381413	100.112494	99.9552538
99.7370319	99.9508774	99.8012906	99.7291135
100.40515	100.053302	99.6198119	99.9656193
99.8671344	99.7282361	99.381833	100.205926
99.9060419	99.7192599	100.036783	99.8020174
99.5234807	99.8234477	100.159954	99.8020174
99.6819276	99.8393163	99.6821881	100.067374
99.6192531	99.7283963	99.2177564	99.8657651
99.8414308	100.065163	99.4356212	100.017792
100.364658	100.261197	99.6991382	100.145288
99.6433722	99.6221248	99.4708773	100.106417
99.6993568	99.6896064	99.64874	99.5610198
99.6451327	99.5943948	99.8618588	99.9039447
99.9762867	99.7255111	99.9432191	100.059254
99.9002322	99.8253711	99.5282815	100.145806
99.9396679	99.8454072	100.286514	99.7954526
99.717138	99.9428629	99.9979113	99.49088
100.341947	100.376124	99.7601584	99.9652738
99.8394942	99.9669062	99.2374185	99.9023898
100.200128	100.760822	99.8920666	99.70457
100.096496	100.740094	99.8834769	100.199964
99.685489	100.269792	99.904499	99.9534777
99.9043646	100.429184	99.7272808	99.888613
99.8039	100.289556	99.8572559	99.8711427
100.392788	100.58472	99.9478994	100.291293
99.9798449	100.460034	99.6228486	100.257564
100.368155	100.76741	100.072675	100.071272
100.01521	100.429666	99.7634478	99.9422345
99.842256	100.203433	99.9727641	100.023705
99.8012608	99.836446	100.630325	99.9700831
100.334726	100.253243	100.102965	100.123856
100.140483	100.107669	99.8798602	100.185434
100.169514	100.078265	100.644114	100.234558
100.242883	100.29759	100.291712	99.9880722
100.122712	100.193631	99.9802236	100.018688
100.243586	100.265293	100.328105	99.8709697
100.095793	99.8353213	100.643662	100.201694
99.8054835	99.8104163	100.396144	100.036851

100.49554	100.494419	100.086238	100.053283
99.9789652	100.011745	101.056191	100.057434
100.299185	100.265936	100.643436	99.9522669
99.9756223	100.168566	99.8753393	100.033218
99.7915838	99.913089	100.596192	99.742451
100.113387	100.361539	99.8249316	100.235769
99.9344511	100.183669	100.395014	100.138558
99.8952154	99.8856132	100.289451	100.297174
99.7757488	99.7895283	100.078779	100.139077
100.146993	100.166477	99.526328	99.8176941
100.030869	99.9404041	100.355908	99.9856506
100.432023	100.381945	99.7926074	100.188029
100.280887	100.1149	100.001246	99.90124
99.9200237	99.8656893	100.738148	99.9337588
100.19485	99.9011989	100.385972	100.153953
99.9647137	99.7066188	99.4388491	100.204807
99.8283563	99.8528351	100.263456	100.091683
100.183765	100.148802	99.7923813	100.260158
99.5442052	99.5775951	99.9924299	100.214321
99.6902395	99.7374689	99.883929	99.856613
100.047408	100.107026	99.6538166	99.9434453
99.9837157	99.9307635	99.4142104	100.096353
99.8589708	99.8271267	100.556409	99.964029
100.403169	100.360254	100.070867	100.240266
99.8246615	100.071195	100.299623	100.258083
100.086468	100.081157	100.219378	99.9216507
99.4351193	99.7312025	100.64434	99.6767214
99.9626023	100.034561	100.009835	99.9107535
99.7863055	99.9617742	99.8956833	99.8396617
99.8600264	100.232033	100.40722	100.100332
100.139251	100.27638	100.214405	100.316547
99.5044417	99.7617311	99.7654821	100.120396
99.6969254	99.733934	99.852961	99.9117913
100.230918	100.0675	100.132351	100.174191
100.212444	100.369252	99.7932855	100.350277
100.216139	100.25035	100.319063	100.194429
100.208573	100.072481	100.333982	99.8823859
99.8489419	99.8422304	100.093924	99.9536507
99.7935192	99.7691223	99.7539539	100.253066
99.8406725	99.5623307	100.02498	100.136483
99.70801	99.5719714	99.7211776	100.050515
100.273849	100.261116	99.7659342	100.095834
100.21438	100.048379	100.535387	100.039964
99.973335	100.132253	99.6852367	99.9619534
99.7136402	100.194917	99.9639484	99.7768726
100.234965	100.217894	99.9343367	99.9875533

99.813577	99.663236	99.3617682	100.028029
99.871111	99.7819764	100.152243	99.7502348
100.566797	100.332778	99.7444601	100.190969
100.153327	99.8174861	99.666475	100.210169
99.9099948	99.728953	100.115624	99.6011323
100.168986	100.005318	99.8380422	99.6888295
99.6450217	99.6561662	99.5378562	99.9131751
99.4796334	99.5390325	100.112233	99.6007864
100.068345	99.759321	99.3527265	99.8597265
99.7333461	99.5734175	99.6884013	99.9676615
99.7748691	99.4684953	99.9144449	99.9498453
99.6480127	99.7500017	98.9736513	100.306688
99.9816044	100.186722	100.127604	100.032007
100.146113	100.109758	99.7485289	99.8498671
99.8406725	99.9167846	99.9469952	99.8095644
99.532241	99.8133085	100.039673	99.6824295
100.24429	100.001944	99.6908878	100.026126
99.7224375	99.5753456	98.8676369	99.9143859
99.69499	99.6711092	100.175299	99.7004187
100.507328	100.053039	100.101157	99.9922236
100.301824	99.9273893	99.8724008	99.9797696
100.463694	100.475138	100.154277	99.9301264
100.365692	100.366841	100.36834	100.013845
100.293203	100.143018	99.8658455	99.9206129
100.058668	100.07714	99.8296785	99.8887859
100.33543	100.161817	99.4829276	99.8989913
100.113387	99.8499429	99.5410208	99.9877263
99.870935	99.6335107	100.184567	99.6068404
100.261005	99.9620956	99.8766956	99.8962237
99.8769171	99.9645057	99.7663863	100.205326
99.6349928	99.606999	100.58489	100.090472
100.005005	99.9870005	99.5591043	99.768224
99.8339866	100.164549	99.8669757	99.7531753
99.6923509	99.8640825	99.9289117	99.8652616
99.6932306	99.9363872	99.5548095	99.7810239
100.103738	100.509165	100.266191	99.938979
100.290338	100.547352	100.054855	100.19445
100.026729	100.089874	100.087245	100.117159
99.8838996	99.8876366	99.9496464	100.055073
100.387587	99.5409005	100.184184	100.218366
100.335918	99.9143673	100.442841	100.191441
99.9656809	98.7925921	100.974397	100.174652
100.039235	99.1423831	100.105162	100.035751
100.12875	99.1507842	100.164658	99.9505409
100.182228	99.1571996	100.152254	100.02799
100.211683	100.529327	100.096663	100.479221

99.8314082	99.9708838	99.2212254	100.269523
100.60216	99.8573926	99.4580598	100.481122
99.901671	99.892219	100.066111	99.5962394
99.647606	99.1700304	100.052098	99.2930955
99.8567489	99.3722676	99.6163323	99.7324483
99.9421502	98.9248712	101.159546	99.7509791
100.13599	99.1011414	99.8448971	99.7939007
99.9266825	98.5203967	99.9677937	99.9708138
100.351221	98.9676404	99.3452706	100.102113
100.102915	98.3392385	100.393682	99.994096
100.261706	99.2486952	99.4844769	99.9977388
100.210037	99.4029698	99.8419108	99.7600069
100.276845	100.165331	100.127215	99.8783185
100.22353	100.25148	99.375363	99.9876024
96.5637772	96.3455825	99.7438232	97.8535579
99.1031105	99.2503754	101.196301	98.367192
101.523639	102.146462	99.7500255	100.663578
100.060297	99.623384	99.9671046	99.7091661
100.424281	100.842154	100.206466	99.9744566
100.559541	100.402853	100.484878	100.064893
100.202139	100.540325	99.9296613	100.157863
100.986713	100.960685	100.31558	100.324323
100.285237	99.867474	100.193372	99.8818029
99.3749469	99.9122289	100.308918	100.05444
99.7724993	98.1582331	100.216803	100.534497
100.4539	100.036565	99.8635039	100.60561
98.8630323	97.2554974	100.282271	101.336704
99.9451121	99.5369291	99.5336355	101.194951
100.702206	100.362986	98.8768851	100.532121
100.446002	99.8576981	100.521633	100.212505
100.64412	100.752949	100.481203	99.5773919
100.386928	100.443178	99.8784352	99.9754069
101.00432	100.692309	99.8171018	100.526261
100.979802	100.551781	100.380589	99.6575334
100.520543	101.014758	100.57975	99.5994071
100.412763	101.16109	99.2049157	99.1054124
100.067537	100.175718	100.831286	100.918099
100.076258	100.372151	101.122333	100.579794
99.7713475	99.9757717	101.505954	100.087067
99.9600862	100.716901	100.999207	99.5396969
101.446136	101.962401	100.00248	100.808657
101.546511	101.991118	98.6928847	100.328125
100.937019	101.952931	100.216803	100.202052
100.355829	101.517602	100.801653	100.191124
100.369651	101.11664	99.7468095	100.48524
99.0876428	99.9241432	99.5219201	100.711252

99.6043294	99.7723125	100.526457	100.089759
102.159953	102.632198	99.5979552	100.751797
93.681522	93.5758181	100.550347	95.6314528
95.1624707	95.7171806	100.931901	96.4030918
101.137111	98.5130648	96.9760073	103.600147
98.5673362	96.9521415	99.1208407	98.9766475
99.9209233	99.4252709	100.09092	100.020704
99.8264717	99.4865225	99.3728362	99.6914273
100.206582	100.400562	99.821696	100.244657
99.9582761	99.8636553	99.5200824	100.51264
100.546871	100.354432	99.9553892	100.630001
100.672916	100.672604	99.6847869	100.061409
98.4026218	98.9354107	99.9590646	99.8582039
99.748475	98.8900448	99.134164	100.34523
102.097918	103.072721	99.2623441	101.71698
98.8623741	100.276836	100.658771	101.23423
100.07741	100.568125	100.763061	100.858706
99.8498378	100.747603	99.9558486	99.9328021
99.9515296	101.583436	100.382886	100.383717
99.8118267	101.153452	100.536104	99.8007111
100.163634	101.253044	100.121472	99.9541837
99.9400111	100.145168	100.629138	99.6222142
99.8022828	99.8821377	99.5469589	99.2693382
100.651196	101.643924	99.6018604	99.3151107
100.573693	101.340568	100.486027	99.931535
99.8906461	101.140316	100.615815	99.52275
99.6454668	100.620976	100.333957	99.5883203
100.00797	100.615324	99.5003271	99.5566438
100.121674	100.293791	99.5012459	99.6974458
100.199013	100.224597	99.5285818	99.5884787
99.8616853	99.3126962	99.4525467	99.7316564
99.872052	99.2253249	99.6379254	99.7451189
102.578239	102.072379	100.777993	101.430466
100.339209	100.490224	100.523241	99.5377963
99.8008019	100.183202	100.204628	99.8086303
100.123484	100.354279	100.068868	99.6719462
100.073626	100.269046	100.428828	99.6480305
100.235707	100.653205	100.328214	99.2611023
98.84378	98.8080196	99.4745992	99.3770382
99.7594999	99.8894695	99.2526961	99.7535132
100.550162	100.624183	99.8926774	100.270632
99.0861619	99.8699179	99.2226037	101.023898
99.0020769	101.117099	99.4470336	100.913506
99.6937899	100.139832	100.999431	100.263209
99.7023597	100.695977	100.320544	100.309639
99.4210419	101.107041	100.187056	100.035953

99.21405	100.450043	98.2479342	99.5296408
98.9322377	99.4771712	100.499596	98.7875447
100.648161	101.599981	101.015688	99.9898384
100.734517	101.720117	100.90441	99.8965039
100.139416	101.269722	100.536231	99.8086968
99.6405587	100.755969	99.9926632	99.7153623
99.3284229	99.9714886	99.451614	99.6204485
102.578163	103.709783	99.806284	101.576841
101.590172	102.114347	100.91334	101.001831
100.107279	100.866004	100.255059	100.081752
99.4480695	100.136924	99.6521892	99.301753
99.8561204	101.168716	99.0303146	98.2300645
100.572352	101.642985	99.959234	100.370599
99.764655	100.487385	99.7607196	99.8473888
99.7251024	99.9384321	99.8724555	99.7488427
99.8843016	99.4168736	99.8937494	99.9361434
99.7849257	99.0529458	99.8440636	99.8413876
99.8233247	98.9132208	99.7737707	99.8998203
100.057179	99.6058773	99.5049634	99.6806185
99.8996282	99.3582595	100.282536	99.9118227
99.7766856	99.546192	100.502115	99.92193
99.3920366	99.2150146	99.444974	99.7955889
99.7572389	99.7494283	99.430778	100.012422
99.67286	99.3322428	99.9180199	100.161346
99.695438	99.7071895	99.590597	100.152818
99.8938601	99.3985089	99.8456664	100.092333
99.9108348	99.6866822	99.7142393	100.036585
99.6080927	99.0022898	99.8671893	100.059642
99.8637013	99.561802	100.239719	99.952094
100.04037	99.9277193	98.7869227	100.971351
99.9447841	99.2973498	98.2364858	100.421609
99.7872329	98.4571633	100.362445	100.319431
100.167103	99.4274334	99.5267152	100.220095
99.8918825	99.2561822	99.681268	100.243626
99.8673269	99.1609917	100.497307	100.152976
99.6333074	98.5929092	100.136683	100.193248
100.39041	100.843201	99.4083393	100.352279
100.204842	99.8484449	99.4316939	100.337276
99.8726006	99.8475266	100.244298	100.029162
99.8599108	100.285526	100.109894	99.7742688
101.078625	101.53448	99.4710762	100.874858
101.065606	100.761937	100.327184	101.616322
100.310646	100.551508	101.517355	100.377864
99.5963917	99.7038226	101.654277	99.348973
99.5434901	99.6883656	100.850603	99.9181398
100.545489	101.291301	100.666742	100.350858

100.362723	100.728422	100.739325	100.399657
99.740429	100.245582	101.491024	100.25705
100.335036	100.249561	101.381119	100.488096
100.738967	100.965021	100.783058	100.494097
99.7753672	98.0568121	100.342067	99.6624569
100.231705	99.4705905	99.5757142	99.8598649
99.4225251	98.7107496	100.471433	100.261314
99.7524596	99.5781772	99.8122372	100.375495
99.4910829	99.3085217	100.214303	99.7701627
101.238319	101.446483	100.468915	101.180919
98.9304249	98.3460566	99.4218483	96.9266979
99.7862441	98.941074	100.411444	99.5177964
100.066244	100.151769	100.022658	99.8938191
100.64915	100.708527	101.401498	100.637021
100.190175	100.204262	100.246359	100.392709
99.7041725	100.336641	100.200107	99.8500735
99.8773799	100.112897	100.116305	100.012896
99.9975209	100.202884	100.062498	100.007526
99.6725304	99.2064444	99.9324449	99.9811524
100.436884	99.6969358	99.8944363	100.495677
102.052608	102.151076	100.268569	101.703656
99.3653386	99.7056591	99.1649473	99.0290141
99.8287632	100.008065	100.142637	99.8947667
99.2417368	99.8625245	100.01327	99.4249356
99.64369	99.7214221	100.241321	99.6717746
100.827301	100.960583	100.09753	100.201776
100.029822	99.9156292	100.066849	100.286898
99.4310948	99.9024678	100.446247	99.8889234
99.1474698	99.3108173	100.798856	98.2412772
100.605477	100.544621	100.039601	99.9124544
100.94909	100.519982	101.830353	100.741884
99.16395	100.663074	101.734645	100.634968
100.894705	103.040542	101.310827	102.37279
100.795824	100.974968	100.354431	99.9200349
98.9625614	99.3806033	98.5655115	98.6016653
99.4638905	100.002403	98.3711185	99.100081
100.622287	100.741736	98.3544039	99.9952079
99.6608294	99.9035391	98.805469	99.5673852
99.9718117	100.59038	100.025634	100.121391
99.8648549	100.003474	99.8404001	100.017633
99.9162733	99.441513	99.9010764	99.3770839
100.561475	100.027195	98.7571569	100.398552
100.142053	100.184213	102.600369	99.5054781
98.0986669	95.5772669	97.1035568	98.5260185
98.4134396	96.8484126	93.0666386	99.1057664
98.7142041	98.3657987	104.195583	100.155029

100.046632	100.658177	97.9463846	99.2507428
100.340969	100.325163	100.227583	99.6976745
100.391234	100.224922	100.113329	99.8821326
100.147656	99.9721007	99.7307249	100.151555
100.636468	100.385456	100.521078	100.521509
101.408868	100.31873	99.8579756	100.828301
99.940961	100.314761	98.6830682	100.352859
97.8389295	101.177151	100.189413	100.295168
98.8464658	101.179289	102.151483	99.421891
99.0243439	100.140236	99.9953114	100.336895
99.0817387	101.120324	99.9601453	100.992147
98.8423188	100.461672	100.878255	100.514493
99.2609324	100.414179	100.348585	101.037352
99.3988752	100.443652	100.132048	100.485252
99.2167115	100.210001	100.50126	100.250693
99.1602803	99.5864739	99.9564829	100.557011
99.7859732	100.488397	99.9317619	100.250377
99.6635407	100.051027	99.4773997	100.310123
99.9498216	100.590104	100.239172	100.638411
100.038098	100.481831	99.7010329	100.579455
100.210857	100.419676	99.9846373	100.498213
100.420741	100.561852	99.942749	100.482091
100.087269	100.293383	100.286096	100.391365
99.9587318	100.025677	100.013478	100.426138
100.033478	100.152123	100.14807	100.454747
99.8671549	99.9245806	99.7557395	100.651846
99.289808	100.822839	100.230245	100.424241
99.825244	100.509624	100.160202	100.236468
99.4176856	99.947793	100.585494	100.431196
99.7498375	100.229854	99.9326775	100.29574
100.112845	100.353704	100.170045	100.712699
99.4219757	100.039115	99.9036075	100.074299
102.702408	103.613517	98.4299632	101.19952
100.817409	100.851243	100.826981	100.117765
99.5303829	99.1361231	100.163407	100.193318
99.6072745	99.8694512	99.9484715	99.9604969
97.8387681	97.1128279	100.656681	99.9367881
99.3714847	98.8235195	99.6943949	99.9298335
100.295009	100.221608	100.183778	100.197743
99.7953784	99.8131001	99.9516761	100.310123
100.12885	100.079432	99.8978851	100.422187
97.5151964	97.1630705	99.5183724	98.4372843
99.2203416	98.8395544	99.310991	99.9103923
99.7212919	98.9383597	99.2734517	99.8632908
99.5614036	99.8222629	100.282433	99.3775761
99.8930604	99.7724784	99.9166547	99.826305

99.7467024	99.9729907	99.9642654	99.616561
99.4760968	99.2837967	100.028586	99.7151897
99.2549923	99.2462293	99.5769703	99.7381082
99.7041315	99.6616088	99.4178863	99.5141389
99.970117	100.142349	99.9535072	99.8533331
100.018133	100.101881	99.6726496	99.7033353
99.7656778	100.23077	100.225438	99.8414787
100.403746	100.548872	99.4657259	99.5765721
99.240307	99.9648969	100.723062	98.818364
99.3589444	99.8444063	100.227727	99.1553454
99.6510004	100.625228	99.6282434	99.1425426
99.7803631	100.767862	100.274651	99.3846887
99.7297071	100.764961	100.186983	99.3483352
99.5978693	100.164187	99.5831505	99.146336
99.5647037	100.086304	100.002949	99.5093971
99.7437323	99.6073956	99.2365992	99.5101874
99.4241208	99.2104944	99.8729352	99.4033397
100.078194	100.143571	98.0868453	99.2653543
99.8325042	99.866397	98.2674457	99.1066633
100.868231	100.786035	101.374963	101.457946
101.384031	101.182325	100.352018	100.11903
101.34839	100.744344	99.1274149	100.541521
100.300784	99.9639806	100.995679	99.9734578
100.196337	99.7286498	99.6280145	99.9179791
100.330979	100.274904	100.723291	100.036365
100.429651	99.6347313	100.528499	100.183834
99.8486745	98.9470644	99.6451818	98.9626717
100.195512	99.4213911	99.9782282	99.5489118
99.777063	99.2178246	100.406954	99.256503
100.314974	99.8999938	100.113507	99.917663
102.595156	101.81639	99.8832356	101.840923
99.3051533	98.9440101	99.781605	99.7727231
100.029188	99.1008464	100.24993	100.468972
99.5074475	98.6474414	99.5536227	100.042688
101.467193	101.003193	100.931359	99.9318883
100.919712	100.070422	99.7589441	99.2561869
100.126045	99.4490322	100.383377	100.412545
100.063509	99.3157137	100.197283	100.694522
100.62122	101.063973	100.261146	100.56523
101.462903	100.913856	101.062059	100.661488
100.038593	99.7341475	98.9699332	100.354538
100.511988	99.7433103	101.074649	100.418551
100.33923	99.4504066	99.8479853	100.357066
100.421731	100.08157	99.9743369	100.134678
100.313159	99.6674119	100.133192	99.2764184
100.11631	99.4123811	99.4151395	99.7072867

100.280323	100.669668	100.695136	100.738146
103.24428	102.811316	101.023147	102.051457
99.4508513	97.234082	99.8999451	96.2587598
98.6764903	98.0391853	98.9424655	99.2266299
100.487238	100.981813	100.439	98.8300604
100.025723	100.430519	99.2544532	98.0546239
100.423711	100.840706	99.7447524	100.568865
100.506543	100.713954	100.169358	100.272031
100.289069	100.285747	100.60289	100.093266
100.146506	99.9962031	100.688727	100.079989
100.267948	99.792942	99.6980572	100.268079
100.279993	99.632746	99.629159	100.155542
99.9549367	98.937138	100.171418	99.8647133
99.8735626	99.2387479	100.582281	100.080928
100.49255	99.9422936	100.012026	100.465932
100.570684	99.7931816	99.1263148	100.567165
99.8998815	99.483652	100.087156	100.372449
101.339031	101.429288	99.458083	100.815346
100.799987	96.6463997	100.462578	95.3863795
97.4354476	98.2550061	101.746916	99.9132599
101.268792	101.852027	102.643195	100.21095
100.746198	100.744993	99.2616413	100.108925
99.9462685	99.7655287	100.230754	99.7085782
100.316707	99.8590292	100.870395	100.00611
100.518375	100.535686	99.9626279	100.298422
100.202713	99.5912082	100.783777	99.9732091
100.531699	99.8568903	99.8093804	100.154006
100.068158	99.0467051	100.219266	100.152108
99.629784	98.5727857	100.160448	100.276752
99.6115253	98.3750901	100.104847	100.251443
100.329866	99.3507346	100.072222	100.2864
100.182151	99.1706086	100.596526	100.303009
100.366713	99.7966955	100.3006	100.487602
99.8664894	99.4200961	100.369067	99.8659648
100.052038	99.3091788	99.927705	100.099118
100.094477	99.3547069	100.539776	100.346982
99.840993	98.6653696	100.493365	100.131703
99.2743147	98.9426628	99.8387892	101.150049
102.148501	103.060506	99.3425155	100.798579
100.304863	99.9910299	99.2770349	99.7433773
100.03016	100.090947	99.2267183	99.7274014
99.772235	99.2363034	100.30979	99.7781763
99.5154614	98.8456484	98.6144176	99.511331
99.7765118	99.9494741	98.8905847	99.3667568
100.652108	100.805187	98.1213604	100.093108
100.556702	101.377802	96.2846881	100.32895

100.17294	100.183225	97.8571406	100.221864
102.039113	101.391552	100.791589	100.238315
101.374232	101.817193	99.3160935	99.9333484
102.533085	103.809121	100.089453	101.918792
99.0693564	101.380552	99.2411929	100.875453
99.7386784	100.27092	100.150798	100.291304
100.269826	101.30844	99.5483772	100.870866
100.40175	100.997383	99.1635353	100.198137
99.8511916	100.586714	99.4502712	99.6051302
99.6287971	99.9707104	99.3551521	99.9885523
100.359146	100.050614	99.5975451	100.133917
100.530383	100.448755	99.7781336	100.222971
99.8021727	99.5939582	98.9624985	99.67805
100.340065	99.8374874	98.8531345	100.210475
100.738302	100.304073	98.899775	100.366755
100.46327	100.061919	99.7360882	100.115885
100.447479	100.45731	99.2756564	100.375771
100.478568	100.53813	99.3585985	100.114303
100.436129	100.60352	99.8006497	100.433664
100.186099	100.437449	100.180207	99.9656166
100.133462	100.350824	99.4865727	100.067957
100.251568	100.357393	99.2993213	100.460396
100.11767	99.8164039	99.867279	100.524774
100.457513	100.384129	99.1019606	100.435878
99.7376914	99.6125972	99.5488367	100.142617
100.110104	100.411324	98.9519297	100.538693
99.9395243	100.093544	99.295875	100.355208
101.109398	100.805798	99.2016749	100.854574
97.9712012	98.387618	97.3139966	99.4983604
99.161637	99.1686225	95.6032307	100.576972
100.525777	98.9802464	98.7697329	100.727241
100.442544	99.5788331	97.3330664	100.051981
100.607695	99.3495124	98.6203913	99.7378411
101.046069	99.5123744	101.462248	100.342395
99.3057328	100.160767	100.978611	99.0413858
99.4381497	98.8228844	101.775176	99.2987405
99.9768642	99.7655287	101.321637	99.8023775
100.285947	100.158323	100.860516	99.9382519
100.431523	100.124253	99.7645779	100.318985
100.261108	99.827404	100.348389	100.216802
100.082633	99.6972366	100.506231	100.439674
100.278051	100.136781	100.399395	100.315505
100.134942	99.8316818	100.36608	100.047869
99.9044873	99.5824998	100.401922	99.8726083
100.05286	100.21149	100.268434	100.107027
100.448959	101.135495	100.750922	100.284344

100.214557	101.219523	101.680976	100.094373
100.084936	101.027633	100.454307	99.397285
99.3843605	99.3133038	100.174004	99.4210116
98.6883907	100.534158	101.995282	99.4535962
98.9912223	99.959252	102.069264	98.9548627
99.324814	99.8069317	101.509578	99.1728312
99.5384904	99.4697492	100.858448	99.1427775
99.5080592	99.2208728	100.950121	99.1921289
99.584713	98.4759241	100.756666	98.8295862
99.6945943	99.962002	100.846271	98.8166156
99.9740679	100.092017	100.966204	99.1117747
98.3120307	99.7490286	101.332435	97.425445
97.6532363	97.2379586	101.926815	96.4771711
99.1766058	99.8743071	100.435926	98.7043096
98.4284917	99.7927233	102.95911	100.136606
99.7140044	100.742701	100.481877	100.43572
99.6753486	100.370838	100.870855	100.681686
99.4986831	100.023266	100.656952	100.346033
99.4059091	99.4165822	100.392272	100.515441
99.6890015	99.7291673	99.9617089	100.614619
99.3455401	99.8475708	99.7186267	100.246223
99.3850417	99.5368192	99.9243011	99.9202805
99.6556413	99.9071817	100.116193	99.8909977
99.5341405	99.9340833	100.352821	99.9310689
99.0804218	99.3178841	100.374011	99.7217737
99.5941966	100.011743	99.6374442	100.3381
99.4685301	100.046596	99.7025854	100.294638
99.3319285	99.7868858	100.300237	99.6209791
99.6907029	100.17569	100.236469	100.079333
99.5764922	99.8911084	100.091275	100.290477
99.5166097	99.9027827	99.854647	99.9192017
99.3761895	100.02274	100.431696	100.137128
99.3123148	99.5144858	100.296312	99.9341513
99.7703727	99.9897477	99.6957181	100.408996
99.3317549	99.8378127	99.7339788	100.405605
99.1252036	99.3749019	99.9325418	99.7510565
100.142686	100.627773	99.7545807	101.246947
99.6356804	100.101753	100.406385	100.562499
99.6827186	99.8777422	99.9564793	100.292789
99.2560773	99.7423882	100.169366	100.085497
99.3147448	99.6620217	99.8622992	100.098598
99.7387825	100.382782	100.011614	100.543543
99.332102	99.9171641	100.089312	100.217424
99.8151545	100.3706	99.6345011	100.610739
99.6172817	100.141513	99.9272442	100.299262
99.5369177	100.01225	99.5489541	100.409304

99.481548	99.9965154	99.7127883	100.134045
99.2927011	99.9176716	100.035551	100.002581
99.3925053	99.9384823	100.002196	99.9865522
99.6212739	99.8281688	100.031627	100.396204
99.7514533	99.8291839	99.937447	100.382641
99.6205796	99.8768962	100.006905	100.408533
99.4004896	99.6366428	100.02888	99.9289112
99.5999245	99.6471328	100.321623	100.103529
99.4291291	99.4684654	100.140915	100.016143
99.5848237	99.7813024	100.169366	100.139285
99.415764	99.4009576	100.388138	99.7636944
99.3610887	99.6254761	100.358707	100.22775
99.2132048	99.6505166	100.218614	100.122486
99.7346168	99.9049822	100.206253	100.335018
99.788945	99.8895857	100.508021	99.8732739
100.268005	100.198531	100.170739	100.357519
100.004001	99.968091	100.324566	100.053594
100.398011	100.371277	99.8797617	100.138361
100.267485	100.146082	99.9890499	99.7943643
100.323375	100.243536	99.8617106	100.05791
100.250822	100.160463	99.9631504	100.138515
100.08315	100.081281	100.002392	100.171034
100.231381	100.113597	99.6849267	99.9985736
100.10207	100.1652	100.251577	100.023233
100.14338	100.008528	99.7932338	100.079487
100.40426	100.291587	100.098534	100.058835
100.318341	100.235584	100.035944	99.5346718
100.465878	100.27416	100.074597	100.115705
100.514999	100.319504	100.302787	99.9970324
100.38725	100.005652	100.035748	99.9454021
100.191286	100.074513	100.393828	99.9134992
99.9968849	99.7366356	100.311813	99.5238834
100.362602	100.094139	100.179372	100.103067
100.07777	99.8752043	100.236273	99.5642629
100.397664	100.265362	99.8016707	99.8678796
100.210032	100.190918	100.073027	99.8418333
100.319209	100.119349	99.725738	99.9387749
100.247524	100.023925	99.6415646	99.9840863
100.212462	100.111566	99.9360736	99.7712463
100.244052	99.97249	99.6568688	99.8057692
100.333095	100.112412	100.085388	99.8672632
100.52038	100.225771	99.9558907	99.96328
100.550408	100.224587	100.128947	99.9794627
100.427866	100.222557	100.145232	100.082415
100.448174	100.004975	99.8487608	100.057602
100.479417	100.269423	100.174663	100.072397

100.128626	99.78181	99.9076233	99.9549576
100.391415	100.159955	99.9133134	100.044039
99.9602611	99.7061809	100.121491	99.6690646
100.475772	100.114781	99.7110224	100.227904
99.8776406	99.7007668	99.8473873	99.7677015
100.323375	99.9515101	99.7541883	100.131117
100.058677	99.7760575	99.9513779	99.731175
100.224265	100.002099	100.078129	99.8227224
100.156572	99.8750351	99.9574603	99.5359048
100.384646	100.233554	99.9804167	100.218657
100.143207	100.031369	100.01593	99.8839081
100.763902	100.418143	99.450065	99.7677015
100.601091	100.316628	100.101477	99.6840143
99.8698298	99.6161705	100.289641	99.6034094
100.123593	99.8640376	99.859356	99.7959055
100.216975	99.9246085	99.9325418	99.5543992
99.9942813	99.6388423	99.8697551	99.7357986
100.146852	99.9926239	99.7194594	99.7310209
100.370413	100.309014	100.252166	99.5354424
100.180178	100.091263	99.9743343	99.5352883
100.371628	100.20919	99.9103703	100.025853
100.501113	100.357065	100.134833	100.030476
99.8976014	99.700936	99.9982717	99.4793426
100.386555	100.073329	99.9810053	100.126031
100.070133	99.9750279	100.240589	99.395193
100.454596	100.13593	99.6001646	99.9341513
100.511528	100.140329	99.4508499	99.8600195
100.110749	100.022402	99.6747238	99.8398298
100.331533	100.050319	99.5575873	99.8438369
100.317709	100.328461	99.770538	99.9877868
100.155273	100.108641	99.8831872	99.5710856
100.59637	100.457677	100.154998	100.099904
100.429591	100.44379	100.43721	99.6040885
100.574828	100.449717	99.8443291	99.6733331
100.379209	100.407718	100.117121	99.7098832
99.9596538	99.8710388	100.107112	99.4282783
100.42073	100.146745	99.81705	99.9945725
100.08526	99.9745133	99.983865	99.7957835
100.189323	100.061561	99.7685754	99.81722
100.387201	100.234131	99.8953549	99.7763518
100.181679	100.085101	100.141652	99.97036
100.139637	100.137769	99.7656317	99.7661733
100.557108	100.601288	99.9828838	100.208475
100.50968	100.486975	99.8812247	99.8001016
100.575523	100.581812	100.110448	100.205699
100.426985	100.408734	100.4796	99.5016868

100.553286	100.574191	99.6072554	100.187655
100.532612	100.643795	100.466451	100.050246
99.9513148	100.01719	100.60952	99.6242913
100.564752	100.450056	100.026256	100.253661
100.203569	99.9699408	99.8653282	99.4790165
100.143633	99.988739	99.9469695	100.447053
99.9959631	99.855289	100.133999	99.2301061
100.163264	100.127269	100.008985	100.1363
100.121917	100.084254	99.8771034	99.6963118
100.197141	100.179938	99.837264	100.16699
100.154404	100.26326	100.082973	100.034053
100.505163	100.503063	99.8072373	100.166681
100.323269	100.322703	99.6317871	99.9025034
100.614612	100.546248	99.9094851	99.9837771
100.605578	100.426855	100.272553	99.6941528
100.393455	100.261228	100.208967	100.202306
100.392065	100.40975	100.383829	99.8685751
100.431675	100.297977	100.146363	99.9853193
100.74682	100.61636	100.382848	100.271397
99.8295308	99.6173484	100.641117	99.5382368
100.264201	100.172825	100.276871	100.232842
100.007603	99.881708	100.222705	99.5894377
100.281921	100.027859	100.15382	100.018168
100.118442	100.010924	100.213677	99.6631546
99.9943996	99.9999162	100.168147	100.080473
100.198531	100.169777	99.9491283	99.6787308
100.376777	100.284598	99.9720899	100.118719
100.194883	100.320332	99.8396191	99.9492319
100.398319	100.564538	99.6502349	100.235463
100.19054	100.320332	100.122223	99.886773
100.442968	100.371137	100.012714	99.804574
100.419341	100.652094	100.33241	100.170691
99.8436028	100.058512	100.44192	99.6559063
100.266112	100.330323	99.9936777	100.341104
100.167434	100.487652	99.805471	99.8951009
100.330044	100.484942	99.7469876	100.25289
100.269239	100.453782	99.7840794	100.251502
99.9968318	100.403145	99.7725005	100.362078
100.011425	100.236502	99.790752	99.9917965
99.8719207	99.6117597	100.586951	99.6551352
99.6554544	99.7861931	100.041171	100.225902
99.8194545	99.692541	100.420136	100.36763
99.6078526	99.4722131	99.8827947	100.162055
99.6882891	99.7897495	99.676729	100.048858
99.4575771	99.296933	100.379315	99.7067988
99.9799801	99.7670562	99.8148912	100.258596

99.5765814	99.6364852	99.8939811	100.037446
99.453755	99.4473182	100.098869	99.7974799
99.8022553	99.8900063	99.4783172	100.188272
99.3649795	99.5257286	100.117121	99.9799216
99.5835306	99.6503722	99.9646322	100.000124
99.362721	99.6772993	100.047059	99.9109855
99.5691111	99.7660401	100.265488	100.259213
99.5350602	99.9201511	100.227023	100.231762
99.794785	99.7868705	100.253124	100.44659
99.7044459	99.7882253	99.9868088	100.154035
99.8295308	99.8519019	99.7895745	100.164831
99.4061533	99.1719507	100.192286	99.6407928
99.8682723	99.6671381	99.9379418	100.048858
99.7173019	99.7396211	100.196211	100.164214
99.8557639	99.916256	99.8541418	100.200764
99.6849883	99.6854282	99.9932852	99.9025034
99.6580603	99.5573975	100.219957	99.8898574
99.7709841	99.7687497	99.6724115	100.123192
99.3576829	99.5270834	100.065899	100.07307
99.6754332	99.5900826	99.7611178	100.07122
99.8173698	99.9494491	99.8835797	100.498254
99.7124375	99.739113	99.8445254	100.080935
99.9447131	99.8512245	99.5481833	100.239165
99.5623356	99.7431775	99.8743558	99.9079011
99.5779712	99.759266	100.13969	99.9631117
99.4940601	99.72421	99.8368715	99.8597846
99.4971873	99.2427401	100.081207	99.9405956
99.7583019	99.5772118	99.4196375	100.158508
99.4732127	99.5794134	99.9175315	99.8273985
99.6290476	99.4984628	100.099654	99.8657991
100.004476	99.8710388	99.8900561	100.198913
99.7343273	99.8253136	100.301402	99.9102144
100.08248	100.19789	99.592929	100.037291
100.030709	100.053093	99.7385487	100.097745
99.6959332	99.6828879	99.5672198	100.106382
99.7040985	99.7489355	99.4186563	100.235926
99.7274112	99.8107691	99.9022354	100.210898
99.3710737	99.3073906	100.2566	100.02853
99.5269064	99.3368022	99.9273509	100.281253
99.5788506	99.7629329	99.5763224	100.107988
99.2569695	99.3109402	99.5984947	99.9154375
99.5423165	99.4754088	100.101982	99.9645009
99.2935036	99.4806488	100.431231	99.9945869
99.4212864	99.518343	100.043314	100.051828
99.5345249	99.815502	99.8900701	100.18436
99.3378293	99.5771663	100.159866	100.134834

99.580409	99.5322037	99.9532513	100.365494
99.4060494	99.4838604	100.085304	100.198092
99.8721625	99.5817302	99.7554666	100.474266
99.4195549	99.5264566	99.7845065	100.295293
99.1752438	99.4781133	99.4532957	100.14764
99.4015476	99.7627639	99.8657394	100.201023
99.5528785	99.6812903	99.960315	100.236664
99.9812454	100.106407	99.7230912	100.452821
99.9440187	100.10759	100.092564	100.126194
100.041501	100.214588	99.9379465	100.038713
100.115954	100.225575	99.3430229	99.8989288
100.137251	100.21932	99.7586061	100.295139
99.9424604	100.043865	99.7305474	99.9466036
100.24443	100.489434	99.8741767	100.671754
99.7814332	99.9884222	99.8469028	99.7704074
100.280444	100.462727	99.7715563	100.074199
100.165301	100.454106	100.336655	99.8776371
99.8745865	99.9481925	100.263075	99.8762485
100.24062	100.317021	100.030756	100.543696
99.4874288	99.3974848	100.227167	99.9037117
100.073879	99.951066	99.8508271	100.314116
99.682566	99.7609045	100.031149	99.6816921
99.7521713	99.8281794	99.8351299	100.189298
100.137771	100.01496	99.5855445	99.9359577
100.369096	100.398833	99.6983681	100.305013
99.9145836	100.078009	100.229914	99.8015734
100.287543	100.000085	99.7672395	99.9800839
100.522504	100.290821	99.9764046	100.038405
100.169803	99.9184427	99.9191099	100.080062
100.105392	100.031864	99.8635811	100.220309
99.7281038	99.7612426	99.90459	99.6366402
99.7282769	99.8709446	99.8266927	100.123725
100.08271	99.9747305	99.6761958	99.8231737
100.213956	100.136326	99.8576946	100.245458
99.7571925	99.9588415	100.151036	99.802962
100.136732	100.118577	99.7497764	100.16168
100.431256	100.412525	100.210685	100.384934
100.080805	99.8582672	100.308989	99.8424596
100.142792	99.9703357	99.712888	100.24407
100.082364	100.024088	99.9008619	99.8446196
99.804635	99.8714517	100.05391	99.8517168
99.8468829	100.028483	99.6081092	99.5621194
100.332042	100.389367	99.8047166	100.113697
100.137424	100.098969	100.095703	99.7674759
100.135174	100.136833	99.8268889	99.916209
100.310399	100.38193	99.8102106	100.217687

99.7435139	99.8171923	100.334104	99.569988
99.9800334	100.044034	100.241883	99.9290148
99.9907685	99.8452517	99.591431	99.8811857
100.024359	99.9054273	99.8843798	100.083611
100.2285	99.9987331	99.7511499	100.280481
100.485797	100.397481	99.7654736	100.442792
100.187118	100.024595	100.213236	99.9842497
100.479564	100.203769	99.9573718	100.033622
100.421733	100.371112	100.649814	99.8685341
100.029554	99.8006272	100.197146	99.6357145
100.345028	100.150524	99.8922284	100.311339
100.019857	99.9372053	100.022319	99.725664
100.124092	99.9424454	100.197343	100.271224
100.143311	99.8613098	100.253656	99.5272504
100.102968	100.160666	99.8035393	100.048125
99.9597751	99.7827097	100.293292	99.4144663
100.106777	100.096603	99.8939944	100.029764
100.11024	100.127029	100.243649	99.7204182
99.8390913	99.8876788	99.8578908	100.034547
100.265034	100.363505	99.8612265	100.122645
100.226076	100.170132	100.155941	100.139154
100.075611	99.8035007	99.8396428	99.7835218
100.285639	100.296737	99.6212556	99.870077
99.8501727	99.7301406	99.8906587	99.793859
100.048946	99.8941021	100.254245	99.8995459
99.9954435	99.883284	100.060581	100.058925
100.373424	100.258874	100.415534	100.140851
99.9488668	99.6885587	100.259935	99.5033359
100.319056	100.323782	99.891836	100.313345
99.7850693	99.8734801	100.11297	99.7339955
100.338968	100.441936	100.29388	100.046428
100.033536	100.132607	99.8104068	99.7395499
100.213782	100.528143	100.403761	99.974221
99.8233349	99.8871718	100.433389	99.2680476
100.332042	100.347616	100.132592	100.441403
99.8576181	99.8198968	100.242668	99.6054741
100.079766	100.176555	99.957568	100.292979
99.7305278	99.716449	100.048023	99.4570497
100.031112	100.164047	100.483424	100.297145
99.8479218	99.7336903	100.550137	99.4919186
100.184867	100.097279	99.8700562	100.03609
99.8401302	99.8371382	100.267784	99.5613479
99.9104281	99.8924118	100.426522	100.012484
99.842208	99.6610064	100.26798	99.5047245
100.24062	100.244675	99.8294397	100.274619
100.242193	99.8383436	100.238794	99.3171899

100.352253	100.138141	99.8634636	99.6032476
100.260971	100.034202	100.025132	99.249565
100.540555	100.108009	100.421653	99.5804493
100.370161	99.8889587	100.308053	99.5590373
100.888818	100.476026	100.020031	100.068457
100.70834	100.666975	99.8915202	100.296441
100.924287	100.715051	100.257041	100.14286
100.954367	100.617884	99.7637939	99.9324374
101.158491	100.713358	100.186801	100.126686
100.642617	100.1571	99.859932	100.084324
100.536903	100.342802	99.8750394	99.9376749
100.167428	99.9448215	100.045733	99.8296908
99.4575142	99.095199	100.41557	99.4957257
99.7659608	99.6504415	100.464032	99.5425547
100.165516	100.113426	100.067512	99.8723607
100.32878	100.160994	100.200731	99.9328995
99.7370983	99.6529807	100.037885	99.7948771
100.232282	99.8244626	99.6021251	100.17752
99.8626329	99.8610273	99.9829494	99.8024252
99.5733121	99.5456564	100.089878	100.030255
99.917576	99.7900985	99.804996	100.037495
100.017552	100.292864	100.363774	99.9007046
99.4323029	99.3029071	100.524854	99.3347508
99.8313362	99.6738023	100.403602	99.9358264
99.9434828	99.9754614	100.02631	99.6664052
100.00625	99.989681	99.8968176	99.8660449
100.131785	100.187232	100.269598	99.9389072
99.9410486	99.9255235	99.891324	100.09372
100.09788	100.081601	99.9752976	100.246069
100.000686	99.7173076	99.6339095	99.9613975
99.8734129	99.558691	99.7422119	99.7498965
99.9123599	99.8171835	100.258807	99.5488705
99.8137752	99.8542561	99.7416233	99.9159548
99.537147	99.6641532	99.806958	99.7927205
99.756224	99.9443137	100.233104	99.4813997
100.163081	100.164887	99.7530029	100.274567
100.128307	100.357698	100.461089	100.296749
99.7551808	99.9913739	99.6031061	100.348353
99.8266417	100.062811	99.5683787	100.291203
99.8398558	100.153376	100.298047	99.984812
99.3917911	99.364864	100.381432	99.378653
99.5117618	99.663984	100.757155	99.7203203
99.7461395	99.7509945	99.8440398	99.9304349
100.067626	99.985449	99.9725508	99.8410899
99.7021503	99.8176913	99.9203616	99.7680736
99.7483999	99.9219686	99.8463942	100.044273

99.910969	100.213132	100.267047	100.077854
99.6515539	99.4632164	100.183073	99.7828617
99.9669553	99.8789711	100.142264	100.080781
100.319913	100.157947	99.8850456	99.719242
100.316088	100.154392	99.9543042	99.6756478
100.113181	100.023199	99.9278172	99.826764
100.146911	100.190787	100.21525	100.022861
100.287225	100.367517	100.043575	100.442628
99.838291	99.9991608	100.391045	99.719242
100.249321	100.253252	100.210541	99.963246
100.261318	100.132893	100.103416	100.058907
100.024159	100.005763	99.9062352	99.8463274
100.01703	100.037757	100.003354	100.289817
99.9100996	99.6604291	99.97569	100.440163
100.384245	100.22786	100.038278	100.361293
99.9899063	100.04876	100.323553	100.134542
99.570704	99.3098476	99.955089	99.7817834
100.089186	100.181307	99.894267	100.378392
99.87463	99.8635665	99.8872038	100.15657
99.9057528	100.004409	99.6929657	100.087867
99.9815605	100.177752	100.107929	100.064144
100.346689	100.375473	99.7204337	100.310613
99.6458161	99.7992397	100.188371	100.166429
100.2295	100.283723	99.7741925	100.35051
99.8815848	99.8031331	99.7271045	100.251306
99.7035412	99.7135834	99.7480979	100.194002
100.246018	100.150498	99.9780444	100.185992
99.8728913	99.9233228	100.078891	100.124221
100.289833	100.424226	100.059664	100.257468
100.216634	100.267979	99.817749	100.212642
99.5564466	99.4606772	99.8036225	99.7364948
99.8448981	99.920445	100.080265	99.8862245
99.8448981	99.7887442	99.9274248	99.891616
99.9563492	99.7662298	99.4002353	100.178444
100.131785	100.168611	100.016107	99.9922061
100.140652	99.9566712	99.806958	99.9952869
99.8805416	99.9807091	100.089094	99.9789584
100.223762	100.399172	99.835407	100.114362
100.316262	100.407636	99.8414892	100.465118
99.8789767	100.066027	100.374761	100.000678
100.115789	100.262224	99.8930898	100.258546
99.8593294	100.123413	99.6968897	100.252077
100.029897	100.242926	100.176991	99.9231948
100.075451	100.371072	100.254098	99.9538494
99.9022754	100.076522	100.006493	99.9518468
100.217851	100.381229	99.8371728	100.264554

99.9881675	100.211101	99.6176249	100.315696
99.4674248	99.4730347	100.018265	100.032411
99.6955432	99.9077489	99.9105516	100.032719
99.7633527	99.9265392	99.5624927	100.037187
99.7070186	99.777741	99.7465283	99.7032215
100.017552	100.244449	100.263712	99.9264297
99.8349875	99.92163	99.9209502	99.7973417
99.6446798	99.4547365	100.05263	99.9589829
99.435762	99.2810321	100.436869	99.8761134
99.0893811	99.2891849	100.418544	99.8924692
99.1486239	99.2469235	100.264425	99.920975
99.4268508	99.8269364	99.9426827	99.8212824
98.9286497	99.2414329	100.397133	99.7254841
99.3638125	99.5766956	100.370514	100.022848
99.1674364	99.5222882	99.6898029	99.7918419
99.3123256	99.3662205	100.271947	99.8644306
99.1250258	99.3851882	99.9781746	99.9768964
99.5648092	99.4525735	100.043565	99.843869
98.9076919	99.1793718	100.239349	99.8095997
99.2857571	98.7248121	100.2081	99.7923093
99.4590301	99.1141495	99.6899958	99.7338956
98.8938301	99.0707234	100.370322	99.4114523
99.5009457	99.3041594	100.033727	99.9985484
99.1230455	99.6021523	99.6471741	99.8677018
99.3057248	99.4545701	100.298759	99.9957445
99.1557199	99.2337792	99.8898306	99.9206635
99.488569	99.3630592	100.392118	99.8499441
99.0312933	99.0396097	100.333287	99.7572611
99.6115104	99.1609032	99.6764935	99.8577325
99.2141376	99.1785399	100.214466	99.6764166
99.6984769	99.0710562	100.178781	100.036712
99.4738821	99.4158028	99.6178547	99.8237747
99.2692549	98.9454366	100.172994	99.9178596
99.4997905	99.4860166	99.5565154	100.131731
99.5681096	99.3826924	100.461559	99.722836
99.8141573	99.2590695	100.571314	99.7290668
99.6331282	99.3920099	100.021768	99.9410693
100.426223	99.9247699	99.6853664	100.019266
100.041887	100.086661	99.1826931	99.9560232
100.010203	100.271679	100.169715	99.9061769
99.9532707	100.536229	100.116092	99.8887307
99.7083782	100.391476	100.041829	100.171453
100.354604	100.370345	99.8321563	100.150891
100.080668	100.373673	100.043179	99.8184786
100.203114	100.038743	100.126315	99.8893538
100.572928	100.410942	99.7563502	100.398098

100.275558	100.812259	99.5140794	100.091231
100.483156	100.789299	100.219095	100.139676
99.9323129	100.522586	100.325764	99.9761175
100.318794	100.396301	100.381509	100.227686
100.308068	100.440559	100.044143	100.486886
100.255261	100.636891	99.726838	100.275663
100.407246	100.381659	99.9855045	99.9211308
99.9829747	100.382824	100.438798	99.8578883
100.215986	100.122267	100.107219	100.210862
100.119778	100.127758	99.8674553	100.201516
100.17605	100.229418	99.8373644	100.218028
100.433814	100.335571	99.5804339	100.117712
99.8555777	100.388481	100.389418	99.7832746
100.17638	100.055714	100.209451	100.273638
100.102451	100.196474	99.8778714	100.26803
100.349488	100.428413	99.7661877	100.241393
100.240244	100.441723	100.156213	99.9655252
99.6666277	100.117608	100.3302	99.6880993
100.108721	99.993819	100.13866	100.300274
100.0574	100.155045	99.8311919	100.160705
100.106741	100.285489	99.514851	100.116466
100.292391	100.41427	99.6795797	100.117712
99.5991337	100.023269	100.420087	99.7553918
100.015814	99.9444031	100.064011	100.16382
99.947825	99.9770143	99.7017622	100.29638
100.185622	100.175676	99.4793591	100.061324
100.227207	100.556195	100.201156	99.9804791
100.236448	100.218437	99.9600429	100.009452
99.902444	99.5557313	100.18206	100.200737
100.693889	100.236406	99.5160084	100.131108
100.122583	100.383156	100.31149	99.8072632
100.545699	100.208787	100.267125	100.200737
100.02324	100.022603	99.9039117	100.094503
100.242389	100.057045	99.68961	100.068956
100.150967	100.145394	99.6953968	99.7634919
99.6712483	99.7810145	100.029291	99.5891856
100.613688	99.885337	99.8325421	99.9969907
100.180836	100.365853	99.4110758	99.7733054
100.105256	99.9530551	100.333865	100.071916
100.334636	100.275506	99.8757496	100.271301
100.113507	100.317601	100.31014	99.7452668
100.305592	99.935252	100.351032	99.9434059
100.107071	99.9820058	99.7467057	99.8641191
100.260541	100.068359	99.5578656	99.8343671
100.104761	100.158539	100.186497	99.9948099
100.351799	100.605944	99.8460444	100.180487

100.652799	100.454036	100.158527	100.153539
100.240079	100.148556	100.328078	100.208526
100.532003	100.050223	100.118406	100.256191
100.549495	100.474501	99.8915667	100.004779
100.264997	100.366851	100.229511	100.012723
100.824421	100.475998	99.7924208	100.470375
100.604777	100.629238	99.6863309	100.118491
100.341567	100.095313	100.526949	99.857421
100.239089	100.241398	100.027362	100.034375
100.356584	100.221764	99.5428202	100.0445
100.433484	100.433903	100.180903	99.954154
100.107896	100.136742	100.095259	99.9030615
100.536788	100.061703	99.9573424	100.036712
100.304602	100.500956	99.4131976	99.8954288
100.28546	100.13641	100.374565	99.9387328
100.418381	100.150506	100.185149	100.183754
100.626214	100.188279	99.4200732	100.087044
100.649215	100.374487	100.333302	99.9962614
100.408287	100.108072	100.372656	100.172679
100.921416	100.167645	99.7977882	100.35908
100.562838	100.34703	99.762486	100.104514
100.421194	99.9639652	100.445961	100.056939
100.758261	100.137027	100.066703	100.330223
100.427813	100.091598	99.8269174	100.167532
100.816838	100.196933	99.6328514	100.162073
100.57591	100.56785	100.042203	99.8959636
100.508563	100.32157	100.300315	99.9964174
100.73526	100.215237	100.342369	100.215419
101.058923	100.501288	99.6733622	100.416483
100.783412	100.857894	100.584662	100.028238
100.99985	100.515099	100.604725	100.258159
100.804593	100.382641	100.153705	100.211052
100.766534	99.8727751	99.6617877	100.123544
100.606688	100.216568	99.8369487	99.7671208
100.292457	99.9493215	100.492645	99.8614911
100.798801	100.195102	100.044325	100.300586
100.529413	100.472999	99.8226734	100.123232
100.020586	99.6123509	100.480299	99.5599739
99.7841266	99.2171384	100.169716	99.9232609
100.00437	99.5597668	99.7237113	100.10779
100.043918	100.214572	100.30726	99.8594634
99.6625046	99.6213368	100.283146	100.124168
100.113085	99.4902094	99.9449775	100.331003
99.944469	99.9792745	100.39484	99.9755155
99.6692889	99.4512706	100.680538	100.071758
99.7215781	99.6311547	100.330023	100.195765

99.7136355	99.6101876	100.06516	100.070198
99.6287483	99.6651014	100.665298	99.7733601
99.6773971	99.6438015	100.389439	100.100303
100.392898	100.01605	99.8367558	100.177671
99.538897	99.8399933	100.864766	99.7140862
99.917828	99.5967088	100.41606	99.9934537
99.9092234	100.022873	99.5904115	100.00718
99.6921241	99.6860684	100.792232	99.8577475
99.7573201	99.7837483	100.204825	100.292631
99.769565	100.206418	99.6698898	100.02855
99.8577616	99.8401597	100.92341	99.8719421
99.7455716	99.7328283	100.1919	100.13072
99.870503	99.8664517	99.8126422	100.085796
99.7700614	99.9306842	100.489944	99.8288905
99.7142973	99.8301754	100.144638	100.009832
100.262837	99.8250168	99.5668766	100.207464
100.079991	99.6514561	100.281796	99.9128099
99.8660352	99.9173717	100.247844	99.6583998
100.071055	99.9281881	100.224502	100.094219
100.078667	100.4938	99.4320335	100.020751
99.4995146	99.9038929	100.434194	99.67665
100.089919	99.9338459	100.184956	100.128848
99.8817551	100.169809	99.45634	99.9848746
99.8440274	99.7576227	100.548588	99.774452
99.8163936	99.8171958	100.036416	100.143822
99.7591403	100.138358	99.6525281	100.045396
99.8299623	99.951152	100.163157	99.7767918
99.6118701	99.9867627	99.8861404	99.9594492
100.280874	100.311586	99.3490828	100.154741
99.4869387	100.076622	100.268485	99.8685104
99.9896429	99.7840811	99.7586278	100.219319
99.9464547	100.156496	99.3201466	99.9934537
99.8036523	99.9028945	100.361853	99.8646108
99.8458476	100.059981	100.018861	100.085328
99.8276457	100.047168	99.4526748	100.117929
100.225275	100.322735	100.017704	99.882081
99.5989633	100.151005	100.236077	99.9274724
100.125495	99.8672838	99.9606031	100.207932
100.002715	100.188612	99.17778	99.9234168
100.050537	99.8950734	100.296264	99.8652348
99.5896969	99.6754185	100.011917	100.204968
99.9376846	99.7942318	99.3556417	100.083613
99.8365812	100.158826	99.8577828	99.7643131
99.6136903	100.087105	100.04529	99.9745796
100.196152	100.3452	99.688602	100.265958
100.028529	100.511272	99.5236651	99.9260686

99.8208614	99.8612932	100.063616	100.091568
99.8590854	99.9731175	99.7084716	100.113094
99.8534594	100.086939	99.5985137	99.9263805
99.8900287	100.188113	99.9960983	99.7338962
99.6912967	99.8822602	99.9960983	99.8499483
100.139561	100.149341	99.287545	100.004997
99.6560512	100.169143	99.9681265	99.6945882
99.9423179	99.8536385	99.891156	100.039937
99.7978607	100.001074	99.6725906	100.067858
99.8574307	100.084276	99.5524085	99.9560175
99.5372422	100.000907	100.042782	99.7268769
99.5721568	99.8363324	100.030822	99.8759977
100.299407	100.329225	99.1760438	100.093284
99.6649867	100.233375	99.9374541	99.6811735
99.7854503	99.9306842	100.079821	99.9684962
99.7897526	99.9969135	99.6955467	100.177515
100.050537	100.092929	99.9200923	99.91203
99.5609047	99.8576322	99.732778	99.9714599
99.7629461	99.9060562	99.6316939	100.161917
100.159748	100.363671	99.4248959	99.7772597
99.5675236	100.094261	100.070947	99.2193046
99.8595818	99.7925678	99.8020322	100.237413
99.7875507	99.8452959	99.2033112	99.5312678
99.7575031	99.9221976	100.21331	99.6033597
100.214691	100.344325	99.5606893	100.02143
100.296368	100.578027	99.5361823	99.8532677
100.208549	100.45302	99.8204253	99.7998605
100.175347	100.376118	99.8391433	99.9197541
100.497238	100.441201	99.2374667	99.8484408
99.7030522	100.004259	99.9728706	99.5474613
100.219174	99.9464999	99.7308878	99.9250481
99.9163735	100.083658	99.2957432	99.7832
99.8818437	100.002595	100.037129	99.6282726
99.9321443	100.048703	99.6789792	99.7998605
99.9185316	100.057358	99.3667557	99.7542387
100.284581	100.450856	99.7472901	99.5698829
99.887488	100.022902	99.6743479	99.7495675
100.486282	100.401918	99.278569	99.9072976
99.4998575	99.5438476	100.20096	99.5074448
99.6306724	99.8571141	100.26271	100.17698
100.004524	100.426886	101.367456	100.973572
98.5924534	99.4346537	100.673926	99.6553654
98.2127913	98.9917195	100.595967	99.2168976
98.8263598	100.342161	101.04076	99.8076458
98.4457017	99.8499566	98.9015081	99.9736283
98.1810836	98.9805671	100.130333	99.858406

99.3378329	99.7437589	100.249974	99.9851506
99.9691643	99.7718896	99.4400839	100.239574
99.1842748	98.8730378	100.038673	99.8640114
98.6943828	98.69643	100.210222	99.5938616
99.5156283	99.8055133	100.824635	99.8012619
99.3560938	99.2387373	100.090003	99.9614833
99.3957699	99.3241282	100.478449	99.9115017
99.8579384	99.7579075	100.237431	99.9868633
99.7575031	99.8531192	99.9796245	100.008506
100.201577	100.544403	100.483852	100.055218
99.698072	99.8153341	100.06646	99.9266051
100.119402	99.9441696	99.8663519	100.247048
100.016975	100.205669	99.3877893	100.222446
99.8463178	100.115284	100.305742	100.252653
100.145964	100.378615	99.7517284	100.458341
100.055157	100.091148	100.079968	100.242844
99.8974485	99.7474209	100.532287	100.231477
100.152106	99.9438367	99.5633909	100.288777
99.8977805	99.9376779	100.515885	99.9758082
99.9854332	99.9686383	100.158121	100.15051
100.293213	100.074503	99.525762	100.265888
99.9934016	99.8176645	100.412839	99.8165211
99.96435	99.8236568	99.958205	100.115944
100.464535	100.250611	99.4385401	100.244712
100.424692	100.586516	100.37193	100.229142
100.23511	99.6370618	100.060478	100.232567
100.303838	99.9554884	99.8667378	100.191772
100.556005	100.512444	99.5975464	99.9974513
99.9967218	99.8178309	100.27834	100.045409
100.299688	100.091148	99.9199972	100.359779
100.398629	100.110124	99.5479534	100.091186
99.773772	99.8724279	100.276218	99.9502724
100.352811	100.130765	99.8538089	100.282393
100.359949	99.9816217	100.02613	99.7634254
100.448598	100.264927	100.104475	100.173866
100.410748	100.179203	99.7254847	100.165614
100.110438	100.010085	100.267341	100.011621
100.310312	99.9113781	99.8943323	100.259504
100.25802	99.980623	100.340862	99.8574717
100.282257	99.7142965	100.16526	100.091031
100.417222	99.9343488	99.7146784	100.015046
100.225814	99.8852448	100.486361	99.7819544
100.275451	99.8304814	100.230677	100.183987
100.432495	100.10047	99.4655557	99.990756
100.127537	100.016577	99.791287	99.9171071
100.08537	99.6383935	100.177803	100.101151

100.713216	100.45535	99.75443	100.277255
100.579745	100.215323	99.5427433	99.9789223
100.054825	99.9496625	100.117211	99.7819544
100.221498	100.197513	99.8401081	99.8745994
100.178999	100.278909	99.4568722	99.9518295
100.357625	100.258102	100.204433	99.8501535
100.017141	99.7695593	100.398753	99.9955829
100.376052	100.040713	100.256149	100.058799
100.289561	100.173876	99.7409222	99.865257
100.006848	99.9301874	100.188803	99.679967
100.313301	100.19152	100.005096	100.210768
100.357791	100.451355	99.4344878	99.9364146
100.10911	100.181034	100.076881	99.8074901
100.600164	100.5469	99.7872347	100.319763
100.278439	99.9852837	100.089231	99.8454824
100.179995	100.056526	100.388139	100.051014
100.486614	100.541407	99.7511495	100.310732
99.9680022	100.14225	100.311917	100.186011
100.252541	100.499793	99.6928729	100.254989
99.9982158	100.268256	100.311917	99.902315
100.136667	100.136091	100.175295	100.154091
100.440961	100.374453	99.5990901	100.062536
99.7251314	99.549507	100.302075	99.6094322
100.129861	100.113953	99.741694	99.9666216
100.199751	100.341662	100.071092	100.075927
100.058311	100.144414	100.078425	100.121082
100.221498	100.301713	99.7268355	100.093055
99.5338893	99.7803788	100.311145	99.7480105
100.18398	99.9689712	99.9284878	100.038558
99.9082391	100.011251	99.4308214	99.8992009
100.116082	100.372622	100.332757	99.8694611
99.5156079	100.244506	99.8236387	100.157839
99.2296155	100.175536	99.5994454	99.9682942
99.534091	100.447086	100.228266	99.8275386
99.6418538	100.32797	100.203591	99.9117114
99.3240112	99.9576299	99.8970846	100.061352
99.787738	100.821924	99.3280231	100.026748
99.4288035	100.638837	100.084459	99.9315076
99.9157992	100.549375	100.016989	100.210369
99.9623369	100.652831	99.6861926	100.186364
99.2751631	99.857673	100.503929	99.8309679
99.5024057	99.922645	100.25429	99.8925387
99.2193838	99.6884125	99.9834463	99.9263637
99.238527	99.7480535	100.059398	99.9728146
99.3088287	99.7345593	99.5173248	100.114973
99.1863784	99.7362253	100.432026	99.8256681

99.2101423	99.671753	100.056699	100.256041
99.5854145	100.086574	100.139013	99.999937
98.9914811	99.336564	100.201471	100.188235
99.7322888	100.006609	99.6649877	100.14116
99.2068418	99.1901271	100.358194	99.9263637
99.9539205	100.046425	99.4695174	100.056208
99.3788002	99.4461835	100.279736	100.062131
99.9014418	99.8371818	99.5984816	100.285345
99.3188953	99.2332751	100.451303	99.8875507
99.824209	99.869168	99.561855	100.201796
99.7253576	99.9123161	100.10682	99.8537257
99.9380779	100.047924	99.4490837	100.29875
99.8623303	99.8558404	100.259881	100.060261
100.166476	99.9609618	99.7910603	100.062755
99.7808068	99.704239	100.219206	99.8445291
99.9285063	99.7730428	99.8635424	100.10188
100.032804	99.9827858	99.9020967	99.8905123
99.999303	100.162375	99.97188	100.115285
100.208888	100.298483	99.8222893	99.9782703
99.9171194	99.849843	100.045519	100.108115
100.246349	100.190863	99.642819	100.105465
99.6885566	99.7405567	100.130531	100.02098
100.004584	99.9206459	99.5959755	99.887239
99.697303	99.6009502	100.163688	99.8513876
100.297177	100.189697	99.507879	99.9954166
99.9529303	99.7169003	100.340074	99.8075865
100.350316	100.31381	99.4569873	99.9695413
99.9154691	99.8280191	100.160025	99.6344088
100.398339	100.323306	99.4693247	99.9241815
100.122248	100.085408	100.160218	99.8499847
100.386457	100.403105	99.5443128	100.01911
100.211198	100.370619	100.313857	100.069925
100.589111	100.342964	99.7393975	99.9539537
99.9601915	99.8115262	100.408507	99.9157642
100.227041	99.9842851	99.6971806	100.122923
99.9245456	99.8571732	100.140748	99.9450688
100.122578	99.9502998	99.9470124	99.9675149
100.016961	100.116228	100.042242	99.9268314
100.064819	99.9088176	99.8747231	99.8068072
99.612644	98.9324047	100.320796	100.097047
100.235787	99.6224409	99.5477827	100.117311
99.6997784	99.456679	100.149423	100.098606
100.444052	100.172871	99.5169392	100.160489
100.007884	99.7552171	100.376122	99.8878625
100.496695	100.271828	99.7766024	100.075225
100.013825	99.7032395	100.230965	99.902203

100.296187	100.072914	99.8510122	99.9436659
99.9618418	99.7142347	100.216507	99.7443011
100.217469	100.021103	99.8560243	100.027839
100.012835	100.043426	100.2167	99.9190376
100.489269	100.487735	99.6925541	100.066028
100.125879	100.173704	100.165808	100.014122
100.415667	100.452917	99.7284096	100.117779
100.207072	99.9649601	100.466532	99.8453084
100.307574	100.409935	99.7989639	100.023162
100.037259	99.9961134	100.367254	100.08676
100.467981	100.580695	99.6144816	100.04405
100.077691	99.8933243	100.171206	99.9815437
100.485804	100.351794	99.7675422	99.8384499
100.216149	100.124725	99.9558799	99.926052
100.31203	100.596355	100.240603	100.036879
100.179018	100.003277	99.9998318	100.022851
100.234137	100.037596	100.301134	99.8845891
100.095679	99.7318938	99.7839278	100.068522
100.109706	99.8878267	100.293616	100.092527
100.141722	99.8371818	99.7259035	100.222683
100.349491	100.05892	100.271832	100.104374
100.336289	100.016271	99.8269158	100.085045
100.092213	99.9376386	100.228266	100.030177
100.16268	99.8201891	100.033567	99.902203
99.9382429	99.9586295	100.295929	99.7743851
99.98115	99.752385	99.6460961	100.138666
99.9580462	100.222016	100.015061	99.7868551
100.124229	99.9839519	99.9464341	100.032047
100.163175	100.225015	100.346821	99.7715793
100.140236	100.020603	99.8473496	100.134925
100.056898	100.002277	100.559448	99.9519273
100.211033	100.034763	99.8417592	100.100009
100.225225	100.252836	100.22364	99.9642415
100.317146	100.203524	99.8348194	100.234218
100.214334	100.227014	100.361664	99.8560638
100.273909	100.246339	100.059591	100.056052
100.10426	100.351794	100.308652	99.9050088
100.357247	100.448752	99.889181	100.019733
100.215654	100.485236	100.244266	99.9230903
99.4185238	100.120955	100.474606	101.193032
99.5386885	100.08681	100.70119	101.348274
99.5351885	99.8150739	100.892881	100.932042
99.6346869	99.9410621	100.574589	101.700652
99.5615214	99.582383	101.06802	101.140158
99.5685213	99.6863983	100.41891	101.62396
99.7886843	99.6630028	100.543498	101.00772

99.5368552	99.3604414	100.69694	101.127657
99.7718513	99.888106	100.599194	101.209924
99.7396852	99.471096	100.808555	100.508377
99.9111823	99.9500726	100.673231	101.615514
99.4585231	99.3942701	100.369702	100.66514
99.5606881	99.5140933	100.287836	101.100967
99.504189	98.9488061	100.954839	100.675106
99.6210204	98.8251891	100.932248	100.783725
99.832517	98.8323026	100.431213	100.722912
99.9105157	99.3542763	100.257192	100.932042
99.5788545	98.9989168	100.703874	100.467497
99.2256936	98.9714113	100.252942	100.803828
99.4256903	98.8874718	100.264797	100.80028
99.3141922	98.5973986	100.038884	100.61007
99.6425201	99.1724866	99.87985	100.764637
99.4428567	98.7998966	100.280455	100.340972
99.3538582	98.9756794	100.212457	100.806699
99.3171921	99.2335047	100.346216	100.200933
99.4535232	99.2330305	100.018977	100.482701
99.4771895	99.1558884	100.147591	100.663113
99.6746862	99.0227866	100.320493	99.9076787
100.141512	99.6819722	99.9968328	100.440807
100.493006	99.8247167	99.8896918	100.568008
100.308842	100.00951	99.8400356	100.124748
100.05118	99.8174451	100.082054	100.476957
99.9706813	99.4733091	100.141775	99.9793031
99.8813495	99.442642	99.7876953	100.152789
100.144012	99.3340423	100.039108	100.458882
99.9455151	99.4179818	100.087646	99.6632438
99.9591815	99.5736887	99.8919285	100.771732
100.44284	100.605938	99.6637785	99.6291208
100.142845	99.8944291	99.812076	100.116977
100.069346	100.326773	99.8270623	99.8759207
99.9516816	100.021524	100.063041	100.420705
99.6808528	99.699835	99.7749457	99.8010868
99.5778545	100.094872	100.023227	99.5625643
99.8266837	100.164901	99.7753931	99.7968637
99.6640197	99.8961679	100.018082	99.5892545
99.8050174	99.9908567	100.072212	99.9189967
100.06368	99.8953775	99.8353384	99.6007414
100.306342	100.250421	99.947624	99.6720279
100.224177	99.9655642	100.058344	100.05059
99.9573482	100.073215	99.9395716	99.5304684
100.147178	100.206791	99.9037834	99.7039548
100.256677	100.053298	99.8212468	99.7335167
100.059013	100.033854	99.8422723	99.2786008

100.502839	100.192722	99.6695941	100.118835
99.948015	100.273184	99.7621961	99.2726884
99.9601815	100.433001	99.6514763	99.775917
100.074013	100.475208	99.6557261	99.818824
100.126679	100.382258	99.8632979	99.3280959
100.385841	100.402808	99.7190266	99.8985567
100.161512	100.608784	99.6825673	99.4541142
99.9323486	100.395853	99.9641761	99.5710105
99.9555149	100.31302	99.7465388	99.9357203
99.8846828	100.550611	99.9397953	99.1267369
100.285343	100.811756	99.7449731	99.6818255
100.105679	100.511724	99.7252895	99.7438211
100.301843	100.473785	99.8156548	99.5476988
100.326009	100.644668	99.5259937	100.088767
100.284176	100.986907	99.8111813	99.5169544
99.9823478	100.420039	99.9073622	99.6069916
100.122845	100.430946	99.6950932	99.9193346
99.9970142	100.537965	99.8221415	99.427424
100.020847	100.501133	99.5919783	99.9755867
100.014847	100.582069	99.6968826	99.3537725
100.064846	100.094872	99.7029219	99.7399359
100.097513	100.801797	99.6116618	99.4304646
99.9130156	100.520418	99.8212468	99.4086733
100.156012	100.481373	99.5913073	99.7406116
99.8750162	100.311755	99.7362497	99.5277656
99.8665164	100.278243	100.070199	99.8333516
100.03168	100.396643	99.6550551	99.5073257
99.9198488	100.417984	99.5843733	99.7178066
99.951515	100.368821	99.6959879	100.078969
99.5811878	100.358388	99.7729326	99.4466815
99.8796828	100.242517	99.6367136	99.9990673
99.9166822	99.9750489	100.031055	99.3547861
99.7561849	100.042232	99.7760641	99.2402547
100.047847	100.035119	99.924138	99.979472
100.004014	100.375935	100.336821	99.3302919
100.307342	100.202049	99.888126	99.8139252
100.312342	99.9674611	99.7644329	99.8826778
100.612671	100.448967	100.336374	99.3735368
100.935832	100.592186	99.7977607	100.079138
100.648003	100.702524	99.9514265	99.6189853
100.389674	100.495758	100.397661	99.5309752
100.756668	100.506349	100.140209	100.227117
100.390841	100.535752	100.201721	99.2686342
100.370508	100.521999	100.205747	99.3931321
100.726669	100.532748	100.062817	99.9679851
100.493339	100.40597	100.273073	99.7740588

101.009164	100.4594	99.8872313	100.456348
100.441263	100.933423	99.8533493	99.2052253
100.499247	100.733667	99.8645004	99.7099324
100.424601	100.62975	99.664672	98.9615561
100.374615	99.6767547	99.6147148	100.595236
100.538903	100.671095	100.244754	99.8826087
100.319964	100.188736	100.29092	100.590683
100.19	100.243388	99.9026373	100.008406
99.968394	100.47736	99.8190038	99.3382737
100.314299	100.483379	99.9661988	100.236561
100.086028	100.401164	100.1832	99.8006549
99.939402	100.210914	100.094883	100.189851
99.9522318	100.368057	99.8069605	99.6606927
99.8270997	100.2342	100.078156	99.6379278
100.132182	100.165767	99.7193126	100.07434
100.188667	100.582702	99.6022256	99.9252719
100.069866	100.306752	100.268395	99.8352239
100.61105	100.287743	99.9590621	100.52087
100.475087	100.07278	99.8007159	99.9905314
100.578892	100.68155	99.9728895	100.112956
100.677365	100.366631	99.9499182	100.228299
100.397276	100.703886	99.8555795	99.8704674
100.793333	100.810021	99.5977651	100.869089
100.577059	100.758379	100.018609	99.8895225
100.546734	100.647651	99.489376	100.136227
100.220324	100.441084	99.6481683	99.7802508
100.101024	100.184618	99.8885869	99.9014952
100.247817	100.055355	99.7661474	100.10874
100.101357	100.029059	99.8007159	99.6296649
99.9888884	99.9357553	100.16915	100.109078
99.9782247	100.039356	99.977573	99.8687811
100.105523	99.9615762	99.8660616	100.347688
99.8975803	99.8765099	100.028645	99.945676
99.7201293	99.753742	99.7888957	99.8831146
100.014381	99.6384194	99.8720832	100.064391
99.7112984	99.955715	99.8785509	99.8171806
99.8567582	99.7168324	100.148409	100.475003
99.7441226	100.059949	100.033329	99.8418004
99.9602296	99.8882322	100.031768	100.240946
100.067367	99.9202311	100.035559	99.977041
99.6849723	99.8668469	100.184984	99.788345
99.8819179	99.8795197	100.009465	100.306037
99.8914153	99.7857409	100.062768	99.542315
100.171338	99.843719	99.9572779	100.085638
100.052704	99.9744074	100.393065	99.728313
100.161008	100.127748	99.908882	100.247185

100.129183	100.200142	99.7735071	99.7360699
100.24765	100.189053	100.099567	100.23791
100.259647	100.068661	100.344446	99.9805823
100.075698	99.8712824	100.063883	100.16169
99.9447339	100.023673	99.9327454	99.9569742
99.9872222	100.138679	99.752989	99.9991315
100.043373	100.323227	100.083509	99.9767038
99.8755863	100.350315	100.037789	100.19491
99.9135758	99.9664869	99.8952776	100.16911
99.7586186	100.080542	99.6903196	100.037242
99.8221011	100.161173	99.928954	100.065571
99.5816675	99.9685462	99.8691839	100.11987
99.8760862	99.9731401	100.046041	100.068101
99.9602296	99.8750842	100.078156	99.9227424
100.290139	100.036504	99.5373259	100.095081
100.120019	100.26113	100.028199	99.7254463
99.674142	99.8627282	99.8767667	100.084121
99.7851113	99.6777051	99.8923783	99.6856498
99.687305	99.8605105	99.8522342	100.045842
100.034876	99.9140531	100.063883	100.042469
99.7033006	100.01702	99.8640544	100.28597
99.9592299	100.015436	100.056969	100.530482
99.637652	100.006089	99.7425069	100.113799
100.094859	99.9191222	99.8388528	100.562522
99.9927206	99.9596752	100.058307	100.297942
100.099858	99.7870081	100.008573	100.638742
99.6681436	99.2640961	100.309431	99.8298278
99.7419566	99.4448422	100.20238	100.447685
99.658313	99.3374401	100.293596	99.8058824
99.6803069	99.2808876	100.090646	100.382594
99.6918037	99.2374832	100.244977	99.7556309
99.8452614	99.4147442	100.219553	100.244318
99.8645894	99.3876561	100.561001	100.023245
99.8860834	99.634776	100.186545	100.141286
99.7607847	99.7028923	99.8745365	99.9416289
99.7566192	99.572996	100.04894	99.8180237
100.039208	99.8736585	99.9927386	100.05714
100.098358	99.8760347	100.114286	100.033026
100.019213	99.8090272	99.9992062	100.134203
99.7539532	99.9020139	99.9597312	99.4374276
99.9610627	99.8571839	100.195243	100.082603
99.7069662	99.7938198	100.374108	99.5929038
99.9225734	99.886965	100.124768	100.041457
99.7756139	100.004981	100.250553	99.8181924
99.6884713	99.8662132	100.249215	99.5165147
99.9943869	99.847204	100.117854	99.7684467

99.903412	99.9222904	99.8736444	99.6020097
100.193332	99.9496954	99.9590621	99.7708075
100.133848	100.0788	100.045595	99.7515838
99.9968862	99.7127138	100.270179	99.6222452
100.082529	99.899321	100.038681	99.6799164
99.9059113	99.9889811	100.215315	100.127627
99.8425954	99.8415012	100.292704	99.8726596
99.9898881	100.368057	99.9441196	99.9525898
99.71662	99.8408911	100.219619	99.7911625
99.7498828	99.8582941	100.154016	99.6664204
99.7836446	99.9282228	100.100909	99.6778832
99.6189936	100.136901	99.9083387	99.3547337
99.7420661	100.29163	99.9395783	99.958553
99.6660605	99.8581359	100.345917	99.4369961
99.8952414	100.015555	100.152454	99.7775083
99.7567017	100.033749	100.255545	99.5652782
99.8649722	99.7625773	100.092429	100.032893
100.027794	100.104785	100.047801	99.8520165
99.8074275	100.225815	100.161157	100.092398
99.6930034	99.8282343	99.9973716	99.6989545
99.751546	100.040077	100.054272	99.5789324
99.8636417	100.150191	100.162272	99.6001722
100.024634	100.142755	99.9458263	99.6981117
100.112614	99.8942077	100.118983	99.9202875
100.116772	100.263153	100.157586	99.7818912
100.125919	100.240687	100.057396	100.105041
100.329321	100.698071	99.9888923	99.7142944
100.181135	100.75028	99.7302728	99.6259635
100.401668	100.212209	99.9893386	100.02615
100.354435	100.610423	99.9897849	99.6067465
100.212402	100.088015	99.4698683	100.179886
100.172321	99.9489482	100.096223	99.6729947
100.146708	99.931387	99.8904875	99.7941968
100.577628	100.311407	99.5231988	100.004404
100.038272	100.358711	100.041777	99.2225745
100.231362	100.098931	99.840281	100.063067
100.256808	100.187845	100.086628	99.7488514
100.073696	100.171075	99.9641238	99.2424658
100.498629	100.242744	100.102024	99.7690798
100.152529	100.056689	100.406834	99.1035638
100.041099	100.295586	99.773562	99.7539085
100.26529	100.030426	100.158479	99.7736312
99.9757374	100.182466	100.47266	99.210606
100.208245	99.909554	100.10738	100.231131
100.099974	99.9793245	100.011429	99.6792318
100.202424	100.118865	99.9511816	99.8678621

100.20259	99.9881842	100.167628	99.7877911
99.9160306	100.081053	100.113851	99.7330057
99.950125	99.6231946	99.9029833	100.365482
99.9870468	99.8429478	99.9864378	99.7124401
99.9783984	99.7119502	100.144644	100.637049
99.7731668	99.581269	100.129471	99.9774329
99.5898887	99.4942537	99.9101238	99.7046859
99.7442282	99.4773253	100.02995	99.6640604
99.9464661	99.6032602	100.100016	100.463927
100.06904	99.8214313	100.090421	100.504721
99.9253442	100.019035	100.000049	99.7582913
100.234356	100.170916	99.9929088	100.52849
100.046421	100.361243	99.9058842	99.7766655
100.085338	99.9986261	100.132148	100.192697
99.9652596	100.164113	100.487387	99.7358714
99.7179505	100.083901	100.084843	100.17702
99.5286851	99.8397836	99.9685866	99.8189767
99.5418239	100.177719	99.8837933	99.923996
99.7267652	99.935184	100.184586	100.049412
99.7996108	99.9565423	100.073239	99.8437565
99.7490513	100.04071	100.107603	100.365819
99.7858067	99.7469145	100.229214	100.079081
99.6552501	99.8329806	100.075248	100.108581
99.6672247	99.5154538	100.521082	100.095769
99.8125833	99.7378966	100.307983	99.9979984
99.7849751	99.5013731	100.315123	100.224894
99.7934571	99.6928068	99.880223	100.115998
99.9128707	99.8141536	99.9061073	100.308842
100.185459	99.6616395	100.428478	100.483481
99.9306663	99.4116683	100.16919	100.227423
100.011495	99.7847266	99.9574296	100.156455
99.7728342	99.7173293	100.308429	100.24664
99.8335388	99.8021297	100.399024	100.235514
99.6025285	99.6826814	99.9714874	100.576532
99.6056885	99.7113173	100.241487	99.9988413
99.8090907	99.8668374	99.9473882	100.419087
99.8518334	99.8361448	100.109388	99.7911625
99.8134148	99.9459423	99.6160251	100.296874
99.7650174	99.7174875	99.7537026	100.104872
99.4273998	99.7075203	99.6300829	100.240908
99.9048876	99.7905804	99.5818846	100.150892
99.6243157	99.624302	99.7077357	100.071664
99.866469	99.7923207	99.4372898	100.531187
100.049747	99.7614698	99.5751904	100.071495
100.111949	100.111588	99.7389753	100.329408
99.9052202	99.6809411	100.044901	99.611635

100.008668	99.8995868	99.7902976	100.383688
100.126085	99.8062431	100.137727	100.065089
99.6427765	99.8217477	99.8768759	100.437124
100.214564	100.249547	99.7311654	100.454319
100.309197	100.439715	100.059851	99.9445616
100.450398	100.50996	99.9984873	100.517195
100.402333	100.272645	100.162495	99.7645284
100.46653	100.358237	99.8476446	100.470164
100.431105	100.150824	99.9268593	100.147352
100.565321	100.418989	99.7728926	99.9642844
100.574801	100.596659	99.8179669	100.327048
99.9308326	100.476577	99.6178102	99.1494149
100.436594	100.383867	99.851438	100.541469
99.8396925	100.155412	99.8621487	99.7684055
100.133403	99.9981514	99.932661	100.69824
100.057398	100.070295	99.9150329	99.8331366
99.9890425	100.085958	99.6515044	100.343062
100.283909	100.249633	99.5977626	99.8359596
100.366321	99.9482861	99.7584265	99.9375311
100.292584	99.9873585	99.6480399	100.180999
100.090559	99.932151	99.8160778	99.8327539
100.295086	100.083537	99.8956277	100.343817
99.9559316	100.138428	100.008696	99.9829177
100.391011	100.086226	99.8180889	100.242752
100.197661	100.104101	100.153941	99.6623433
100.365487	100.340591	99.8473615	100.467322
100.015321	100.301203	99.6659163	99.7126229
100.253714	100.275418	99.8634502	100.411644
99.7904414	100.004285	100.056068	100.004346
100.481597	100.127354	99.9081411	100.457368
100.145778	100.061707	99.6326215	99.8896137
100.106908	99.9013044	99.8793155	100.56012
99.7861039	99.9190214	99.977859	99.9036177
100.228023	99.761783	100.387675	100.604832
99.9409174	99.6880676	100.391474	99.4867023
100.18665	99.7905731	100.108803	100.422779
100.238032	100.192528	100.085341	99.7328697
100.160959	99.8196796	100.161762	100.234484
100.076212	100.112327	99.8319431	99.821112
100.054025	100.021369	99.7445723	99.6878205
100.142442	100.194584	99.9742837	99.7347256
99.9706122	100.110745	99.8893709	100.109291
99.909888	99.9615739	100.385217	99.9231896
99.8915372	100.169749	99.9698146	99.6098704
100.228023	100.341857	99.9812108	99.789392
100.283576	100.447052	100.075062	99.6923761

100.23186	100.054272	100.260529	99.6409155
100.226355	100.258176	100.163326	99.563134
99.8948737	100.337586	100.068135	99.5509859
99.9212321	100.239984	99.913504	99.6518825
100.032337	100.385042	99.5716184	99.6520512
100.352474	100.364794	100.017187	99.7794373
100.215344	100.274785	99.9226657	99.6815778
100.190321	100.466983	99.8616626	100.002827
100.055359	100.14175	99.8469145	99.4976693
100.09156	100.390262	99.4073792	99.5513234
100.143943	100.189839	99.9528321	99.4389536
99.9966369	100.395641	99.5705011	99.9422553
100.058696	100.322242	99.7215565	100.363558
99.7894404	100.25517	99.659883	99.8700417
99.7709229	99.8597011	99.938531	100.261142
99.8551694	100.227962	99.4992191	99.7806184
99.9827904	100.18446	99.6710557	100.161933
99.8119618	100.17576	99.9613233	99.9807243
100.017657	99.9193378	99.9099288	100.816411
99.9636056	100.070723	100.02322	100.231278
99.9804549	99.8093974	99.8046816	100.426997
99.6905133	99.5950533	99.943	100.008226
99.8069571	99.717965	99.7186516	100.169863
99.9362463	99.7374221	100.098301	99.9652017
99.8366519	99.5634158	100.07104	99.8867454
99.8404888	99.3666304	100.212933	99.8300543
99.6433019	99.4435096	99.8960746	99.9017617
99.9464226	99.6119793	100.065677	99.9754938
99.9500928	99.7209706	100.084223	99.9827489
100.132766	99.6754126	100.358402	100.226385
99.9255695	99.8158831	100.154388	100.343648
99.9494255	99.7141685	100.220084	100.049057
99.6476393	99.3979516	100.338738	100.066604
100.063367	99.5094738	100.283545	100.332344
99.9108889	99.6978752	100.296282	100.023411
99.6589834	99.4653395	100.298293	100.169526
99.6341265	99.4715088	100.437729	99.607677
99.8636775	99.6255835	99.9114929	100.427504
99.8781913	99.6508935	100.359073	99.890626
100.107575	99.4895422	100.401082	100.442183
100.093228	99.5031463	100.133383	99.8325852
100.2035	99.638713	100.010036	100.014469
100.120254	99.9615739	100.284439	99.8907947
100.122089	99.8231598	100.300974	100.010757
99.9522615	99.9256653	100.336727	99.7202154
99.7100318	99.7472297	100.269467	99.7613839

99.7874385	99.7124285	100.107239	99.9054736
99.7759276	99.6991407	100.136065	99.7132978
99.9864606	99.7445405	100.056068	99.8135194
99.8851979	99.7598847	99.8156309	99.5162289
99.791776	99.8146176	100.065453	99.631467
99.5880829	99.9482861	99.704127	99.599747
100.022995	100.405923	99.7602141	99.6183065
100.196994	100.142857	100.348794	100.227904
100.143109	100.061074	100.217402	99.7274705
100.298923	100.093661	99.849596	100.157209
100.010483	99.9274054	100.246675	99.9810617
99.860341	99.8600175	99.8636736	99.718022
100.036675	100.048261	100.05361	100.031341
99.7590783	99.8707742	99.9678035	99.7566597
100.180311	99.9313601	99.8853487	100.762251
99.7944452	99.8424586	99.8165247	99.6893391
100.060531	100.083062	99.766024	100.726988
100.086389	100.072938	99.5416755	99.8111573
100.083886	100.245204	99.7304947	101.10206
100.079048	100.284751	99.8605453	99.9343253
100.068872	100.419211	99.9255706	100.669115
99.9711127	100.334106	100.070816	99.935169
99.9983051	100.980618	100.016293	100.737111
99.7051939	100.599703	99.9005437	100.260299
99.7762613	101.190691	100.27483	100.438302
100.580284	100.118508	101.754558	99.9556029
100.314734	100.168942	100.589045	100.090407
100.445911	100.255638	100.281035	100.834321
100.863943	100.925727	101.252857	100.239377
101.284106	102.258681	100.284193	99.6456778
101.170885	101.693488	100.659364	100.102704
101.340716	101.177896	99.4654289	100.34943
100.78892	100.190616	100.183769	100.056317
99.8372007	99.1291437	99.769018	99.4251032
99.8985283	98.6778791	99.7239639	99.7806376
99.7611119	98.4070925	99.7094371	100.10644
100.243363	99.5541494	100.037027	99.8312281
100.761223	100.618539	99.9361815	99.4925053
100.330865	100.675225	99.9416554	99.3978622
99.6976538	99.8735637	99.2576325	99.3850978
100.377431	100.423334	99.9494451	99.5295531
100.128317	99.4084054	99.7119635	99.2859405
99.8746364	99.6476535	99.229	99.2615014
100.12299	100.033479	99.6913312	99.4322637
100.149165	99.6020824	99.8852324	99.2261659
100.205623	100.466404	99.791124	99.2059297

99.9061372	100.048484	100.165452	99.2727091
99.7786123	99.7187888	100.006921	99.4378676
99.9208984	99.245989	99.7467015	99.8170628
100.377431	99.4321635	100.980006	99.4700899
100.118577	100.435144	100.437251	98.9286938
100.974424	101.246948	100.563781	100.121384
99.7105889	98.4523857	100.302299	97.5464059
99.4871921	95.9848795	100.238718	99.3170731
101.512068	99.637789	101.067798	100.900633
101.827532	100.769285	100.771157	100.570316
101.064057	101.467161	100.076607	100.890204
101.129037	100.605063	101.35665	100.961965
101.659376	101.547328	100.353038	101.686732
101.787053	101.912452	99.6066968	101.589442
101.661354	101.232637	99.9858674	101.407161
100.477564	100.423612	100.094924	100.634294
100.766549	100.844866	100.271561	100.563156
99.9320074	100.072242	99.7408066	99.9085927
99.7188065	100.573524	100.178295	100.364841
100.945967	101.416728	100.536412	101.010376
101.442066	102.397895	100.189243	101.580102
100.471934	101.023538	100.031974	100.973951
101.070448	101.521208	100.830948	100.816731
100.362822	100.135597	99.873232	99.4758494
100.96712	101.313081	100.395144	101.277494
101.553156	101.925651	100.897266	101.24963
101.603679	100.184642	100.070502	100.577165
101.172712	100.603534	100.809263	100.67928
101.396565	102.237007	99.9281813	101.542121
101.007751	100.216736	101.164223	99.9744381
101.414065	101.440208	100.29093	100.634294
100.692135	100.261751	99.6725938	100.518792
101.094797	100.784707	100.496621	100.084803
99.8848323	100.423334	100.15682	99.5826342
101.13208	100.499193	100.416408	100.24249
102.624334	103.255548	101.640449	101.253521
101.514959	102.211998	100.189874	100.665426
100.929988	100.167553	100.576203	101.01489
100.89164	100.571857	99.9376553	99.9373903
100.778419	101.366988	99.9835515	100.665582
100.621981	100.71246	99.6502772	100.191121
99.7066323	99.5556777	100.726734	99.1383719
100.439215	100.386238	99.9839726	101.091165
99.0740299	99.172075	99.9601823	99.9607398
98.9687229	98.5695089	100.275351	99.7616468
99.1027914	98.4232091	100.593046	99.5778087

99.3496236	99.2093099	100.13703	99.3952159
99.0839214	99.1403975	100.15282	99.9654097
99.8682449	99.6201441	99.7098582	100.567359
99.4681699	99.4625905	100.229033	99.9615181
99.6413481	99.7961762	100.258719	100.030321
100.370127	100.31024	100.332405	100.79774
100.275168	100.045567	99.7332274	100.405002
99.7850038	100.028755	99.8233356	100.607208
99.6329783	99.9041296	100.441672	100.17571
99.8455705	100.207149	100.48441	100.150026
99.2434036	100.082385	98.9007785	100.192211
98.2110305	98.262321	98.8329868	98.9171747
98.7517176	98.760685	100.217033	99.4521886
99.1861848	99.5820756	98.7917222	99.3496067
98.0471352	99.2226478	99.554274	98.6016457
99.519606	100.753446	99.9490241	100.237976
99.0828562	98.9702008	99.026888	99.7703639
98.8874601	98.2305046	99.7418592	99.104593
98.7145863	97.9441572	99.0041504	98.7610447
98.9433093	98.7046937	101.065272	99.2579211
99.9358118	100.784707	100.431566	100.884133
99.188924	100.001524	99.1679453	99.4582595
98.3110112	99.5770739	99.5437474	99.2292791
98.5741263	99.631259	99.579117	99.6887964
98.9185043	99.8150715	99.8437574	99.408136
98.5885832	99.0935761	99.1927883	99.5010668
97.7102139	98.1989661	99.3283717	99.2398642
97.2323762	97.9392944	100.145241	98.4369542
97.4954913	98.4912878	99.2793175	99.1101969
97.8999794	98.5433889	99.1397339	99.4934393
98.0822882	98.8939248	99.8241778	100.123408
97.5685366	99.7265692	97.7506348	100.57701
98.3948611	98.7516542	98.8833042	98.5168093
100.785319	100.877246	100.961859	100.057447
99.6807432	100.174155	99.7684058	99.7514716
98.5707924	99.4676875	99.3032618	99.2766336
98.2804054	99.2815381	99.2132818	99.0559451
98.5474509	100.045271	99.7211927	99.4201277
98.1808968	99.6190834	99.1736905	98.7795399
98.1005835	99.3131961	99.2835721	98.5289699
99.0099816	99.9491716	99.7021381	99.0324445
99.262285	99.2427042	100.264884	98.8642046
98.7433968	98.8006168	98.8144055	98.8144018
99.031941	100.610192	100.263825	99.7475808
98.7152948	99.9080864	99.4078504	99.7520941
98.7200553	100.015583	99.9070809	99.6655619

98.8742322	99.7742783	99.458451	99.8490538
99.5565111	100.948441	100.182737	99.5451015
100.585842	102.329577	99.2225973	100.206389
99.5128993	101.253906	98.3236436	99.7696807
99.7343366	101.302167	100.400172	99.3209891
98.8722359	100.835598	99.8583858	99.3037138
99.7232801	101.630004	100.35677	99.9592424
100.17844	103.234576	100.370531	100.694767
100.637746	101.532075	99.239323	99.7811976
100.436886	100.829688	99.8566921	99.0473853
100.849201	100.888502	99.9259238	100.031145
100.935964	100.126738	100.557478	99.700735
100.838759	99.6120483	99.9644565	100.223975
100.609029	99.5927721	99.7252154	100.007489
100.855805	99.7289721	100.224658	99.5581747
100.987254	99.7006909	100.319295	100.131062
100.643735	99.333598	100.079207	100.075656
101.166001	100.897225	99.2001553	100.762467
100.880528	100.092829	100.472367	99.8828263
101.330774	100.921285	100.128749	100.686051
101.001843	100.101271	99.9629744	100.247787
101.152795	101.002471	100.000872	100.683872
100.693182	100.212707	100.434682	100.34179
100.887899	100.57164	99.7260622	100.897868
99.9173833	100.142215	100.12388	99.931384
99.235258	99.6171136	100.037287	99.0227952
100.481572	100.989104	100.112659	100.280314
100.325246	101.241665	99.8018572	100.471277
100.347819	100.682513	99.9466722	100.134641
100.308661	100.391259	99.5003711	100.411202
100.320025	99.8378759	99.90327	100.387546
100.367322	99.959865	99.7828026	100.215882
100.39266	100.02487	99.9447667	100.3835
100.06465	99.6299175	99.3638131	100.384433
99.8640971	99.7029422	99.8772287	100.15643
99.7727273	99.1613781	99.4476533	100.150049
99.9649877	99.4496776	99.7264857	100.285295
99.9941646	99.4688131	100.003836	100.29759
99.8339988	99.5522497	99.9913446	100.216038
99.7896192	99.3303618	100.323318	100.017294
100.034859	99.6543998	99.6983272	100.217594
100.345516	99.8090319	100.248793	100.134641
100.248004	99.6579173	99.8304391	100.436415
100.359029	99.4320898	100.271024	100.431435
100.159859	99.6793041	100.052319	100.44513
100.692721	100.056387	99.7578199	100.448554

100.464988	100.428404	100.278434	100.293855
100.023342	100.211441	99.8617733	100.568237
100.018888	99.503426	99.8321328	99.6246302
100.397881	100.310496	100.433411	99.8456299
99.9990786	100.797608	99.8128665	100.338521
100.041462	100.048367	100.068622	99.9678023
99.88836	99.3938187	99.6832952	100.282649
100.176751	99.648631	100.779782	100.454468
100.437039	99.9065387	100.860446	100.359843
99.971898	99.948046	100.066716	100.006088
100.419994	99.1276095	99.5592286	100.407623
99.9765049	98.7565769	100.103767	100.224753
99.972973	99.2556488	100.331999	99.8941876
99.8992629	99.3971955	99.4048864	100.228488
100.316186	100.01896	100.311462	100.291987
99.9915541	100.652544	99.5526654	100.263973
100.003686	99.6503194	100.486764	100.339611
99.8714681	99.6605907	101.04824	100.170748
100.002764	100.053432	100.184643	100.250744
99.9772727	100.225511	101.116836	100.042351
99.7813268	100.135321	100.279281	100.428633
99.5643428	98.8977015	100.114988	99.8650841
99.719441	99.0257408	99.4952898	100.233469
99.7905405	98.8214408	100.103343	99.7418223
99.4918612	99.6016363	100.646823	99.8954326
99.5248771	99.2214581	100.602786	100.060093
99.9788084	98.8131393	100.142723	99.6682076
99.3358415	99.2206139	100.401865	99.3108729
99.296683	100.243381	100.262767	99.0547001
99.7972973	99.8496949	99.5353045	99.5407437
100.671222	100.700945	100.149921	100.459449
100.412162	100.725287	100.193323	100.4291
100.252764	100.350174	100.383023	100.396573
99.9914005	99.8751621	100.421132	99.6815921
99.5927518	100.038518	100.001931	99.9449241
98.8313882	99.2739401	99.6934577	99.1356295
98.9092445	98.9877512	99.8971302	99.2693188
99.3221744	99.5862997	100.142511	99.7601871
99.352887	98.9952084	100.612525	99.9877234
99.1827396	98.8778625	99.7607839	99.3071377
99.9897828	99.7545583	99.922712	100.317795
99.9685515	99.9058523	100.077523	100.239305
99.7383925	99.175627	99.4160016	100.306672
100.034707	100.182476	99.0293956	100.759754
99.8733186	100.019814	99.303259	100.185255
100.022091	99.6034046	99.9130363	99.7357766

99.700084	99.7635405	99.1882027	100.151571
100.090708	100.023182	99.6290766	100.431849
100.349175	100.46303	99.6686206	100.556086
100.330406	100.727865	100.315208	100.788894
100.090554	100.368296	100.124639	100.841691
100.006706	100.306964	99.2338466	100.64711
100.898111	100.382611	99.0569502	100.72184
100.461793	99.8779232	99.2357397	100.609666
100.471486	101.262165	100.544058	101.310439
100.587181	100.016165	100.209196	100.399419
101.782438	100.592009	100.048706	101.061025
101.947365	101.944392	101.362073	101.053348
100.53318	100.657551	100.328249	100.400829
100.163325	100.222194	100.21193	100.301502
99.9185504	99.1697324	100.662059	100.048328
99.6105435	99.5301434	100.471911	99.4913757
99.4953101	100.050269	100.436364	99.8043968
101.345659	102.315469	100.876817	100.53243
101.681359	102.104107	101.485753	100.655413
101.66582	101.782712	100.673207	101.300726
100.251635	101.039715	99.5846947	100.867227
99.3742305	99.1478383	100.559203	99.8144235
99.0848394	98.9565454	100.589281	99.9433607
99.9214735	98.8594253	100.766598	99.679533
100.065477	99.1005414	100.26704	99.9372506
100.377945	99.1961177	100.368844	100.531176
99.5519268	98.4668748	100.31037	99.0569374
100.453793	101.08603	98.8646989	100.64664
99.5059258	100.068093	99.1549689	99.7061665
99.8807034	99.6396141	99.5905842	99.8141102
98.3849775	99.1575222	99.8648684	99.9099905
99.5356187	98.653817	100.248109	99.400822
99.5597731	98.4752956	100.45866	98.954477
100.137632	99.323272	100.729999	99.6398962
100.879341	100.739794	100.528914	101.178839
101.516125	102.121931	100.03882	101.53275
99.8490104	100.125355	100.692138	99.6480429
101.093654	102.510131	98.3600919	101.391436
99.2065344	99.2605369	100.548896	99.1689545
99.6759296	98.9655276	100.710648	99.8465403
99.8519335	99.2401866	99.8162797	99.8800671
100.178402	100.562255	100.818763	100.329232
100.997652	102.623812	99.9868659	101.011361
100.282405	101.796747	99.5783845	100.906551
99.4628479	99.2922553	98.6829647	99.8877438
98.7936021	98.47305	99.6896547	99.3608719

98.506211	98.2698276	100.028934	99.6054294
99.0906857	98.702798	99.4557559	99.8465403
99.4090005	98.8866526	99.7657979	99.8357303
99.4223854	98.3356504	100.052913	99.6608896
100.026245	99.1907845	100.64397	100.593843
99.9610129	99.3415171	100.406495	100.533056
100.46964	99.8563098	100.329721	100.143425
100.738877	99.5579322	99.5056067	99.9778275
99.6442365	99.5288803	99.5274821	100.137158
100.412869	100.166056	100.379361	100.275025
100.155786	100.754951	99.5899532	100.449553
99.1197632	100.093216	99.226064	99.6906564
100.037476	99.8071884	99.4092707	99.8526503
99.7376233	99.4272691	99.3291309	99.583966
99.3977695	99.4786361	100.387354	99.4587889
100.198095	100.568571	99.3800333	100.418219
99.2886901	99.896449	100.053754	99.1888512
99.4933101	99.5373011	99.650952	98.9776638
99.8620876	99.8710462	100.339397	99.228018
99.673468	100.103882	100.134735	99.3082317
100.828571	100.462468	100.52029	99.6259528
99.3233063	99.4321813	100.541324	98.214381
98.8902196	100.022761	100.247478	99.8210036
100.031938	100.678462	99.8682338	100.121178
99.7767011	100.395944	100.184376	99.4135121
100.441178	100.714812	100.432367	100.252465
100.302251	100.795512	100.172807	99.4044254
100.863033	100.607447	100.752506	99.2316214
99.4363857	99.3257983	100.076681	99.7020931
100.320713	99.8835371	99.6520037	100.296175
99.2759206	99.3231317	99.071674	99.0655541
99.3462299	99.55302	100.248319	99.5465225
100.095477	100.385278	99.864658	100.085145
99.1063783	99.5924575	100.172386	99.3046283
99.6265438	98.9533174	99.9856038	98.7779131
99.4016157	99.2898694	98.5552879	99.0581907
99.3876154	99.559476	98.9063463	99.1946479
98.9802216	99.2303623	99.1065906	98.9635637
100.206249	100.214054	99.9948588	100.094545
100.018706	100.080584	99.5274821	99.7360899
99.6273131	99.9323779	100.426057	99.389542
100.044553	99.9040278	99.9431151	99.568926
99.5659271	99.7885222	99.4134775	99.4260454
98.9766831	99.669929	100.223078	98.8180199
100.66549	101.004629	99.9172432	100.391586
100.936112	100.260088	100.474856	100.666223

99.8325485	100.295034	99.6801893	100.516136
101.76213	101.707346	99.3596303	101.839191
99.281613	99.1200496	100.511455	99.5568626
98.7006038	99.9421887	100.459556	100.693507
99.1843878	99.7215728	100.310054	99.985972
99.7375877	100.400508	99.6251708	100.331664
99.8361672	99.4478869	100.153383	99.0523234
99.5217456	99.4327438	100.204833	100.097476
98.6231376	98.889123	99.9079381	98.9673756
99.1298577	98.5979322	99.1408186	99.0645919
99.6511597	98.9585864	99.3672854	99.8339357
99.1313766	99.6539154	99.7797334	99.9070809
99.1129974	99.2656148	100.584387	99.0206427
99.413141	99.574866	99.8396185	99.8362652
98.4203584	97.7709005	100.047319	99.4009663
98.9974057	98.1221077	99.7738293	99.4849822
98.5011663	96.9938825	100.126603	98.8451565
98.6360486	97.1254463	99.5865829	99.6859372
98.6255679	97.6868498	99.6150494	99.6244394
99.2850939	98.4687302	100.146846	99.5045497
98.7642476	96.7692378	99.3074003	98.0626124
99.0769984	97.3038284	100.542214	98.785367
99.300739	98.2959052	100.689396	98.7645571
98.984039	98.0790403	100.551703	99.2816978
98.9314835	98.2454747	100.166245	99.6834525
98.8664726	98.5951537	99.237183	99.8938806
99.0217088	98.5530588	99.6489984	99.6481999
99.0950739	98.8222991	99.7255416	99.7093871
99.1799829	98.849112	99.178563	99.9471476
98.5810628	98.8449442	99.3398732	99.967181
98.2942859	98.3781499	99.4547936	99.6637297
98.1153542	98.3862077	98.8650098	99.7086107
98.227908	99.2757565	99.6749345	99.9341026
100.187955	98.4742873	102.329489	99.7314394
102.870412	102.487746	102.855381	101.375729
101.332024	102.005253	101.727475	98.4871957
99.9857833	99.9213497	99.212512	100.782338
101.539057	101.414119	100.668521	99.4936789
100.991325	100.608899	100.234776	100.296256
101.004996	100.750882	99.8562767	100.500007
100.706675	100.82229	99.6667109	100.242523
100.541565	100.603203	99.4630172	100.05042
100.598526	100.740879	99.7181614	99.9907862
101.126055	101.441765	100.044366	99.9111186
99.850901	100.197258	99.4478351	99.9120504
98.874523	98.8895397	99.6671326	100.246406

98.9700647	99.1543344	99.8396185	100.114092
99.2680817	98.8930129	99.8853758	99.7061259
99.8910011	100.00415	99.6302316	100.95068
99.01138	100.332712	99.8141041	100.192518
99.0829223	101.019011	99.7242765	100.892133
99.1388195	100.401898	100.697198	100.810757
99.3848886	100.297008	99.8760978	100.439907
99.1892485	100.793672	100.095606	100.583867
100.337115	101.154326	99.9094142	100.889182
100.773508	100.572083	99.9277592	100.590856
100.775635	100.948992	100.067983	100.161769
100.516351	99.8887018	99.7708772	100.041569
100.762572	100.194758	99.7468388	100.202146
100.190233	100.423015	99.7601232	100.415836
99.4863541	100.616123	100.015057	100.502647
102.107142	103.187104	100.816758	102.671842
100.045022	100.754911	103.280059	96.8797112
98.642732	97.1580941	100.501096	100.530756
101.462958	102.083052	100.091389	100.869925
100.810419	100.283532	99.9178487	100.218608
101.178155	100.608065	99.8100977	100.504821
101.04145	100.681558	99.8387751	100.435403
101.075475	100.781724	99.86197	100.42096
101.117701	100.174058	99.7259634	99.6020766
101.416174	100.888559	99.5897459	100.430123
99.5086826	100.107373	99.6504744	100.328558
99.5507574	100.529294	99.3147805	100.353872
100.695738	100.824513	98.9573678	100.416457
100.337571	100.963996	99.6743019	99.8932594
99.8344964	100.798395	100.076207	99.4882435
100.421265	101.07611	98.7275271	100.704068
100.473365	101.116538	99.8946537	100.735748
99.9997576	101.06708	100.764674	100.540384
99.4356214	100.271445	99.2660712	100.440373
100.277117	101.197532	99.4834709	100.263178
100.567539	100.767414	99.3911129	100.021225
100.045934	100.70837	99.166333	99.9361215
100.802368	102.426201	100.291709	99.7977514
100.831684	101.556658	99.7386151	100.490223
100.667942	100.920373	100.225709	99.8531926
100.781711	100.899673	100.110156	100.038152
100.691181	101.042073	99.6795735	100.68714
99.2032227	99.4772004	99.3624356	99.4797021
99.7395623	99.6821176	100.286859	98.4008503
100.171854	101.15641	101.06663	100.26939
100.849607	101.225873	99.8503725	99.9608138

101.170257	101.487056	100.153804	100.487117
101.109651	100.972054	99.3683397	99.999017
100.599285	100.260192	99.5825765	99.4831187
96.1894836	98.2228297	100.058283	96.6185008
100.773205	98.2642299	101.856312	100.98174
100.749509	100.125294	100.930624	100.644123
100.422024	100.180031	99.5874264	100.812621
100.72551	100.242826	99.995868	100.338187
100.912644	100.659746	100.545166	100.296567
101.095828	100.591672	100.005146	100.12838
102.736897	100.910509	101.461577	100.775194
100.427481	100.472356	100.244981	100.240525
100.389753	100.313378	100.156946	100.166809
100.319224	100.22435	99.9395087	100.127603
100.263324	100.199801	99.6057106	99.9884352
100.207732	100.260287	99.7277772	99.8983569
100.4957	100.489511	99.8834681	100.016463
100.208964	100.28661	99.8881551	99.9817927
100.201111	100.262357	99.7510086	99.9660776
100.330465	100.478715	99.7192183	100.037687
100.615045	100.45668	99.8329297	100.106055
100.232679	100.509771	100.0952	100.00253
100.459358	100.533137	99.6165112	99.9845469
100.159994	100.423997	99.5329598	99.9156921
100.212352	100.445884	99.3693213	99.6002564
100.17678	100.638876	99.5669917	99.8615804
100.248233	100.607672	99.6868166	99.6921167
99.9414776	100.368096	99.6735707	99.8879882
100.117492	100.361145	99.6747934	99.6606866
100.153527	100.6547	99.7722021	100.17005
99.9009773	100.181907	100.176509	99.6835301
99.8492354	100.360997	99.7768891	99.914072
100.000765	100.316779	99.7699605	99.7408821
99.8654048	100.408469	99.3870505	99.812005
100.004923	100.468955	99.4795684	99.6128933
100.230523	100.746833	99.5140079	99.9885972
100.383901	101.021163	99.5144154	100.063446
100.121188	100.280103	99.6754047	100.066849
99.6786106	99.7870496	100.710219	99.6026866
99.9054431	99.8667605	100.201371	99.5825972
100.134739	99.7701906	100.533539	99.8179994
100.484459	99.8490141	100.416363	99.6691111
100.616123	100.002668	100.276975	99.7162564
100.760261	99.7534794	100.123526	99.6728374
100.843726	99.9683586	100.216247	99.666519
100.277029	99.348566	100.134937	99.1808095

101.151097	100.172442	100.278809	99.9463123
100.909481	100.309089	100.734877	100.298687
100.54005	99.9553446	99.5881852	99.5686642
100.904245	100.131921	101.02262	100.066687
100.854043	100.413497	99.9342103	100.002692
100.564689	100.039936	100.544747	100.159357
100.29905	100.150851	100.413714	99.8330664
100.593486	99.9698374	100.408619	100.142022
100.254238	99.8663168	100.889957	99.8614184
100.500628	100.022781	100.651326	100.333843
100.148445	100.04526	101.065415	100.191759
100.302438	99.750078	100.675576	99.9633234
100.345557	100.044224	100.576945	100.359279
100.099013	99.9281334	100.375606	100.128251
100.390985	100.369575	100.461399	100.642312
100.087309	100.226569	100.026321	100.23048
100.283959	100.468215	100.201167	100.246357
100.076684	100.200245	100.126175	100.05972
100.176164	100.266942	100.386407	100.295932
99.9263862	99.962591	100.005331	99.8638486
99.9414776	100.045555	100.003701	100.02991
99.9182245	99.9759008	100.472607	100.151904
99.9391677	100.066555	99.945826	100.161139
99.9407076	99.893528	100.448153	100.090826
99.8393799	100.130294	100.070542	100.03315
100.106405	100.248604	100.484223	100.283133
99.9728922	100.137245	99.9395087	100.025374
100.073758	100.427546	100.295315	100.47139
99.6678311	99.9551967	99.9753747	100.055994
100.333545	100.736038	99.6760161	100.479167
99.4992082	99.7551061	100.566348	99.8444072
99.8415358	100.013464	100.16632	100.211525
99.5701992	99.9214785	100.499303	100.082078
99.3102582	99.5399312	99.6664382	99.5137424
99.7383601	100.243723	100.506639	100.26353
99.313646	99.7165078	100.136364	99.8968988
99.3767834	99.7835004	99.8954914	99.9296251
99.3064083	99.5777902	100.382739	99.5741726
99.5397085	99.3121859	99.6254777	99.9041894
99.4205175	99.7111838	100.210745	100.066849
99.7203429	99.7310006	99.5932798	100.271631
99.4416146	99.4590372	100.010018	100.281189
99.4386888	99.2596862	99.4329019	100.131491
100.027714	100.112548	99.2446055	100.372726
99.4272932	99.3689743	100.228067	100.12339
99.64581	99.8997392	100.487891	100.221083

99.4237514	100.002372	100.745678	100.007714
99.4197476	99.4157065	99.6984323	99.9633234
99.6510458	99.6934374	100.025098	100.057128
99.574665	99.829641	98.8934858	99.7384519
99.4859648	100.168301	100.424311	100.188033
99.2789975	99.3939672	100.170396	100.008201
99.4825769	99.7601343	100.237237	100.138295
99.645656	99.5039948	99.2097585	100.286374
99.339209	99.1222995	99.6399463	99.7640497
99.7345103	99.7864581	99.973133	99.9555469
99.4223654	99.7101486	100.745678	99.8312843
100.193565	99.7727046	98.8951161	100.298687
99.5138376	99.4309388	100.36501	100.147692
100.030486	99.6100294	98.9802978	100.006094
99.9420935	99.5201144	99.3744159	100.025374
99.6807665	99.2446017	98.5768056	99.8965748
99.7800924	99.5408185	98.8741263	99.8079547
99.6237889	99.6651911	99.6175301	100.486133
99.9026712	99.3540378	99.1355811	100.070413
99.2645075	99.6801688	100.120646	99.7258388
99.7386856	99.4278383	99.6340691	100.219543
99.0141956	98.5092487	99.8873668	99.5367743
100.63661	100.328786	99.1072505	100.385946
100.182113	99.6969216	100.184583	100.002637
99.7923458	99.102418	99.1534161	99.8134106
100.771914	100.745679	99.7692974	100.490514
100.322337	99.4315447	99.9030958	99.947601
99.4653105	99.4982595	99.8322133	99.8160005
99.2282215	99.0275491	99.6001598	99.6675655
100.71441	100.16185	99.4943467	100.186845
100.731938	99.8834267	100.558197	99.8828529
100.256684	100.046804	100.377416	100.139417
100.239309	99.6393986	100.224416	99.9344895
100.553891	100.521962	100.138622	100.045371
100.262373	99.5074514	100.628467	99.8404429
101.037755	100.568366	99.9641733	99.973824
100.794977	100.109813	100.350044	100.267133
100.771453	100.277044	100.208279	99.8440041
100.944119	100.447538	100.73714	100.517061
100.41136	99.8592611	100.223395	99.8911083
100.414897	100.433157	100.034239	100.260982
98.593524	99.0990081	100.818236	99.3447962
98.4797458	99.2856615	99.939252	99.4513068
98.9371647	99.3636438	100.172531	99.9929247
99.1227461	99.5019659	99.8628541	99.7646876
99.8217129	99.7391744	99.8514149	99.9930866

99.76467	99.6198289	99.6706338	100.122745
99.9889975	99.6401398	99.6640971	100.324273
99.6201411	99.5500006	99.6414229	99.9571514
99.7532922	99.6343579	100.0563	99.9848312
99.6974794	99.4877334	99.7825751	100.001342
99.8446222	99.5615645	99.8085177	100.123716
99.7883482	99.7565202	99.9784723	100.124201
99.7855806	99.6632677	99.9682587	100.090047
99.7768166	99.7156018	99.5433721	99.9061622
99.80726	99.708782	99.870208	99.9296334
99.7674376	99.7133779	100.393962	100.001666
99.9851536	99.8472524	100.239124	100.140065
99.8470823	99.6819478	100.186626	100.093608
100.024822	99.9569613	100.043839	100.247223
99.9969927	99.8433978	99.8659182	100.100083
100.066797	100.055106	99.4890356	100.082115
99.9854611	99.765119	100.020144	99.9445255
100.189339	99.8376158	100.0563	100.164183
100.054958	99.805741	100.41439	100.107853
99.9560941	99.9738624	99.873272	100.559471
99.9213456	99.8035171	99.8077006	100.166611
100.198103	100.076603	99.9063642	100.273446
100.151977	99.6534828	100.07448	100.192349
100.0751	99.80752	99.5558327	100.176
100.182728	99.8128572	99.941499	100.222295
99.7213113	99.5510384	100.091231	99.8119538
100.594328	100.235088	100.343916	100.226827
99.9055089	99.9400602	100.750826	99.559598
99.9457925	99.8970662	100.273646	99.6835906
100.218553	100.315443	99.843244	100.093446
100.267139	100.103734	100.488745	100.066252
99.8281705	99.9663014	99.7962613	99.8930508
100.124916	99.9757898	100.176821	100.418158
99.7617487	99.5359164	99.9801065	99.8933745
100.470863	100.097359	100.313071	100.486467
99.7560598	99.6585235	99.8812387	99.871522
100.537285	100.17549	100.289988	100.416215
99.9162717	99.9018104	100.062837	99.9254248
100.201947	100.019673	100.073255	100.279273
99.8433922	99.7829097	99.672268	99.9679967
100.0628	99.9290893	100.07448	100.177942
99.6542745	99.9040342	99.8961505	99.8338062
100.320338	100.201434	100.237285	100.325406
99.9554791	99.8770517	99.7756298	100.087133
100.165815	100.142132	100.127795	100.35082
100.155206	100.090391	100.047721	100.159165

100.075869	100.098693	99.9208675	100.166126
99.9783884	99.9319062	99.8356859	99.8945076
99.6388991	100.106551	99.3887379	99.9072953
99.7683601	99.9029964	100.132494	99.6735547
99.7875794	100.026641	99.9098368	99.8406048
99.9139654	99.69366	100.021574	99.7183927
99.8041849	100.059109	99.8489636	100.028051
99.8227891	100.022935	100.039754	99.9058385
99.9702395	100.266667	99.9600878	100.073212
99.7643625	100.080903	99.5946445	98.9703903
100.143982	100.533378	100.185605	100.019633
100.199026	100.63775	100.117786	99.8033746
99.8489274	100.634636	100.090618	99.8436803
99.8369345	100.466515	99.9966526	99.7433208
99.746527	100.451244	99.7327326	99.8948313
99.9696244	100.646348	100.089188	99.6513785
100.175655	100.843973	99.8871626	100.057835
100.452874	100.863394	99.3740303	100.088104
99.7154687	100.498983	99.4933254	99.731828
99.9580929	100.656133	100.022391	99.9981046
99.9382586	100.625889	99.968463	99.8380149
99.870453	100.340498	100.078974	99.8592199
99.8195603	100.35221	100.269356	99.965083
100.169351	100.603058	99.9498742	99.922673
99.9385661	100.525076	99.5917847	99.4998679
100.178423	100.709802	99.7627607	100.090047
100.064239	100.178916	98.8672052	100.14969
99.8751505	100.209933	99.5571748	100.379599
100.003158	100.722454	99.4765528	100.523577
100.074701	100.893934	99.4152719	100.633992
100.058546	100.640332	99.8894351	100.385761
100.100395	100.728805	99.5763123	100.353658
100.112395	100.507697	99.4928401	100.231569
99.924538	100.127664	99.547606	99.9212391
99.8028385	100.096057	99.6211023	100.031168
99.7316035	100.174485	99.633725	100.202222
99.4943587	100.043623	99.5398696	100.191845
100.025929	100.42144	100.228007	100.742623
99.9454624	100.291316	99.7324665	100.264158
99.6825237	100.033432	99.9824762	100.386733
99.8053002	100.312142	100.163468	100.198331
99.7360653	100.092216	99.8892315	100.129423
100.22394	100.1318	100.492268	100.61713
99.9257689	99.7262149	99.8961536	99.6921401
100.157936	99.6355269	100.324509	99.9771763
100.103933	99.4229862	100.670206	99.6123689

100.494418	99.8969565	100.112164	100.013981
100.323485	99.6628515	100.357694	100.115641
100.053007	99.4831002	99.7247301	99.5047101
100.271636	99.7796824	100.522399	100.285074
99.9471548	99.8155736	99.9256743	99.7733705
100.289175	100.040521	100.095876	99.9791219
99.8868435	99.714842	100.611572	99.4466652
99.8394561	99.4664101	100.485549	99.5063315
99.9668482	99.7935663	100.893749	100.141907
99.86715	99.7127742	100.568004	99.7304043
99.9273074	99.7724451	100.821474	100.115965
99.6265204	99.6341976	100.28033	99.6464176
99.7656055	99.6712704	100.847738	100.035221
99.5868257	99.5102771	100.609333	99.69441
100.026083	100.136674	100.532782	100.025493
99.7134486	99.7297597	100.476388	99.699112
99.9260766	99.9600245	99.9930629	100.034086
99.5683631	99.6424689	100.266485	99.6874381
99.6942167	99.8941502	99.8303938	99.7234325
99.586518	99.8477723	100.136187	100.15731
99.4495868	99.390049	100.074906	99.6768992
99.7846836	100.02457	100.380904	100.087753
99.6075962	99.7548688	100.270557	99.8828128
99.7840682	99.9830658	100.208258	100.206437
99.3018857	99.4667055	100.319419	99.6854925
99.8128391	100.098715	100.370113	99.9387499
99.2018798	99.4593205	100.772409	99.5313005
99.5792868	100.04894	100.295192	99.7153256
99.4852812	99.8510217	100.31718	100.180658
99.2623449	99.6788031	100.207241	99.7868279
99.5409768	100.194573	100.692601	100.081592
99.4705111	99.8705181	99.9002255	99.7266752
99.5691324	100.160011	100.116643	100.0002
99.3468115	99.8058254	100.047014	99.8254164
99.6686767	100.130175	99.7554723	99.9252926
99.2863464	100.08232	100.486974	99.9478296
99.236805	99.5986019	99.8951357	99.7738569
99.5745173	99.9879399	100.578998	100.140934
99.6220586	99.6457183	99.5691866	99.7218111
100.077316	99.8588498	99.9914342	100.256538
100.133473	99.9189638	99.6783114	100.134287
99.9588478	99.8666779	99.7913043	99.9074575
100.089009	99.8275373	99.417715	99.9966327
100.165783	99.8968088	99.8776269	100.154229
100.305484	99.8957749	99.3287458	100.145636
100.411952	100.356895	99.4838821	100.373438

100.117934	99.7225224	99.5983001	100.061649
100.100087	99.985429	99.6665031	100.336147
100.324869	99.9820319	99.5152351	100.488231
100.425952	100.186449	99.1577294	100.40619
100.228094	99.8392057	99.4270802	99.9513966
100.290252	100.279796	99.1394062	100.306152
100.216555	99.9204408	99.7021315	100.020142
100.548113	100.283045	99.689916	100.194277
100.5855	100.248483	99.767077	100.428403
100.445646	100.254834	99.9089799	100.135746
100.089779	99.714842	99.6958202	99.9392363
100.285636	99.9235425	99.2135133	99.9544772
99.9920805	99.6052484	99.4443854	99.8056357
100.420106	99.9889738	99.4867323	99.9358314
100.347794	99.9443683	99.5099417	99.7094887
100.390412	100.175962	99.7180116	99.946046
100.035468	99.8721428	100.274426	99.8531418
100.370257	100.243018	99.9588596	99.5773473
100.310869	100.152773	100.598135	99.8035279
100.39872	100.397956	99.6390183	99.7997988
100.041776	99.9316661	100.608315	99.7206761
100.239787	100.263401	99.949698	99.981554
100.056238	99.9944387	100.066356	99.6778721
100.227787	100.217023	99.7619872	99.9283732
99.9860801	100.041998	100.066152	99.7709385
100.212709	100.130175	99.400817	99.7260266
100.251173	100.26281	99.6412578	99.8182824
100.213171	99.9151236	99.5140135	99.8528175
100.470109	100.604293	99.7652447	100.078998
100.643965	100.971181	100.122954	100.521307
100.613194	100.714625	99.864597	100.441373
100.971062	101.083728	99.9883803	100.624264
100.472878	100.436949	99.8033162	100.10494
100.732894	101.225373	99.9743325	100.559895
100.183169	100.45098	100.228414	100.221516
100.49463	101.085524	100.666268	100.135941
100.649651	101.289096	100.400484	100.325228
100.47939	100.945956	99.7561154	99.6061304
100.485394	100.885803	99.5655995	99.9863244
100.653038	100.943141	99.7006333	100.163306
100.69645	101.175013	100.262186	100.125254
100.708149	100.814834	100.412926	100.338506
100.813293	101.125675	100.053108	100.212692
99.7997316	100.209302	100.123685	99.3038212
99.690432	100.211673	100.807421	99.5473526
98.8685301	99.8024531	100.963056	100.052227

99.2935669	99.9080916	100.32338	99.8778364
99.5394139	100.047955	99.9743725	100.217226
99.2946445	99.7514858	99.9352086	99.9009913
100.020948	100.184856	100.658924	100.464805
99.5585028	99.7914892	99.8574928	99.9461677
100.223537	100.141296	100.155913	100.393883
100.006631	99.7824514	99.9664173	100.011908
100.317134	100.005878	100.0527	100.351945
99.8591536	99.8497163	99.8921691	100.344335
99.9348936	100.106775	99.9431638	100.477759
100.012173	99.9061655	99.75122	100.219493
100.256788	100.113442	99.6051713	100.357937
99.8708533	99.8837932	99.7381653	100.085097
99.8834766	100.082032	99.6796235	100.257707
99.8951763	99.8679401	99.5048139	100.321828
100.010172	100.039362	99.7903838	100.197795
100.098535	100.127666	99.5741666	100.085259
99.8106615	99.9589107	99.551525	100.030691
100.170734	99.8683845	99.4191429	100.089793
100.261253	99.6744423	100.134904	100.161525
100.070825	99.7113343	99.836483	100.056599
100.103923	99.5667296	99.9817157	99.9110305
99.7746389	99.5819901	99.6694246	99.8622918
100.234775	99.6251049	100.015168	100.105499
99.9179598	99.5642109	99.5662114	99.8279643
100.277417	99.8131206	99.7181754	100.277461
99.7440042	99.4779814	99.4428044	99.6228085
100.108541	99.6722199	100.075342	100.126225
99.5409533	99.4173837	100.033118	99.7437646
100.020332	99.5511727	100.283196	100.119587
100.024334	99.6925179	99.4909433	100.100318
100.48093	99.8304554	99.8903333	100.107604
99.9736872	99.2821131	99.7065487	99.2725701
100.457838	99.9106103	100.024143	100.159905
100.002782	99.9008317	99.6949219	99.8503096
100.155186	100.08944	100.345409	100.213664
100.040037	99.886312	100.025163	99.8252116
100.161652	99.8197879	99.6928821	99.6030539
100.427357	100.233452	99.874627	100.102423
99.7888016	99.8123798	99.7502001	99.7712914
100.380097	100.141889	99.9054277	100.307579
99.7407714	99.7428925	100.012312	99.4455033
100.000935	99.8947571	100.350509	99.9550734
99.5448019	99.4080496	100.231793	99.4765925
100.018331	99.7642276	100.36132	99.8211635
99.8422199	99.4447934	100.468001	99.6207035

100.132864	100.153742	99.9046118	99.9659222
99.7592446	100.075217	99.5961963	99.2894101
100.149182	100.362648	99.959482	100.518078
99.9456696	100.43658	99.9095073	100.457195
100.555592	100.63882	99.8024186	100.81051
100.069286	100.233452	99.171309	100.07943
100.208604	100.484584	99.8379108	100.525688
100.328526	100.361907	99.4707495	100.144685
100.407961	100.379094	99.44668	100.321666
100.453528	100.549627	99.1925227	99.8758933
100.159958	100.431691	99.4281179	100.027291
100.342689	100.123073	99.9250097	100.027291
100.161344	100.302347	99.9050198	100.304826
100.185821	100.178633	100.546736	100.484722
99.3346697	99.6117704	100.578353	99.3023639
99.674268	99.882608	100.880037	99.4150619
100.224306	100.348425	100.598955	99.7232004
100.239239	100.407096	100.489622	99.978876
100.358853	100.403244	100.090844	99.801247
99.993084	100.044547	100.197525	99.8543576
100.090684	99.9052765	100.011497	99.5609541
99.7455436	99.9725414	100.01578	99.8300692
100.01556	100.059215	100.12654	99.5913955
99.7681732	99.8226029	100.152242	99.8127435
100.238623	100.221599	100.430672	99.6428869
100.266795	99.7963785	100.082889	99.9644649
100.008632	99.6729607	99.8287318	99.3812201
100.330065	100.033435	99.7234789	99.9738564
99.2772489	100.080402	100.273813	99.5687264
99.0531078	99.503465	100.497781	99.9280323
100.00386	100.60978	99.7752894	100.288796
99.7141392	100.083217	100.251579	99.9442246
99.8046577	99.9243892	100.279116	99.9694845
99.7053645	100.226489	99.9802879	99.3983839
99.7758704	100.425617	100.020268	100.201357
99.6622604	100.000544	99.5443857	100.146466
99.7901871	100.044399	100.418842	100.304826
99.8240546	100.059364	100.355812	100.205082
99.9844632	99.8907568	100.840057	100.159096
100.002167	99.8482347	100.482891	99.989401
100.177816	100.005878	100.361116	100.259973
100.449063	100.115665	99.8101698	100.114081
100.595463	100.196857	99.4899234	100.32199
99.6986206	100.183412	99.8696237	100.088545
99.6003414	100.151317	99.8997555	100.12048
99.7221667	99.9172583	99.8944381	100.038694

99.6542585	100.110982	100.222343	99.6183902
99.9460249	100.266395	99.9292965	99.9234142
99.9393705	100.24124	99.8989677	100.087766
99.6617659	99.9804354	99.5127685	100.010965
99.4083898	99.6191549	99.7768649	99.940083
99.4140204	99.4534776	99.5107991	100.083092
99.6037539	99.5279312	99.4889388	99.9165597
99.5926633	99.5820004	99.7548077	99.9972556
99.6540878	99.5593029	99.9107841	100.156778
99.3705114	99.2087206	99.5102083	100.046795
99.4245991	99.3748315	99.5927261	100.086987
99.5571736	99.3618202	100.111269	100.39575
99.649481	99.508848	99.8613522	100.231242
99.46862	99.612071	99.6283722	100.277198
99.7583389	99.6114927	99.8438245	100.305551
99.8105497	99.8367328	99.6614581	100.133878
99.789563	99.4555016	99.8054212	100.397308
99.7175598	99.5001738	99.9682906	100.084494
99.710223	99.6136612	99.5616096	99.9336959
99.8687324	99.7981326	99.7555954	100.095399
99.7752306	99.794952	99.7567771	100.052247
99.6511872	99.7946629	99.9115719	99.9938283
99.7742069	99.7621346	99.6768195	100.132787
99.5836203	99.5331357	100.549854	100.240901
99.9782727	99.9499311	100.55399	99.7056289
100.053688	100.493804	99.4806673	99.7593742
99.9219669	100.226204	99.7355076	100.237318
99.8061135	99.935185	99.5728352	99.7289964
100.203667	100.880962	100.017722	99.8829105
99.8511581	100.515489	100.086061	99.5085628
99.9753721	100.232855	99.376683	100.29044
100.178926	101.033194	100.46005	100.19292
99.6124557	100.052865	99.5456575	99.9643852
99.6919663	100.528934	99.5610188	100.449028
99.5392582	100.075562	99.9708508	99.8945943
99.9965294	100.009928	99.766821	100.551066
99.699815	99.6623813	99.8310234	100.051936
99.2785453	99.1508926	99.7528383	100.239499
99.5865209	99.9983621	99.6460968	99.8137426
99.4464389	99.2113228	99.6762286	99.8838452
99.6720033	99.2784033	100.456899	99.7380319
99.7892217	99.7911932	100.116192	100.192764
99.8347783	99.9009219	99.8698206	100.200709
100.017346	99.899187	99.6275845	100.077796
100.073139	99.8569726	99.8530807	100.187623
99.7339397	100.023373	99.4798795	99.8685784

99.8736804	99.8718633	99.6720929	99.8138984
99.3921806	99.1396161	99.3705778	99.8248033
99.5711648	98.9580362	100.173502	99.4009164
99.9366406	99.7715317	99.3126775	99.6551551
99.8586656	99.8542257	99.6492479	99.9452239
100.061196	100.014698	99.6267967	99.5741476
100.3388	99.7541833	100.215253	99.8093807
100.309112	100.01094	100.985288	99.4111981
99.917872	99.7163059	99.6435366	99.989778
100.262702	100.272611	99.8583981	99.6632558
100.06785	99.9934467	99.6374315	99.1979306
99.7861505	99.53169	100.138447	99.5261663
100.10897	99.7158722	100.275517	99.9950746
100.670493	100.712249	99.966912	99.9576866
100.743861	100.873011	100.319828	99.7737063
100.943832	101.123695	99.7575648	100.000839
100.454313	100.825158	100.526615	100.19697
101.187653	100.589653	101.060716	100.039006
100.947074	100.323934	100.331645	99.3928157
100.644387	100.153197	99.5027245	100.253052
101.523952	101.262338	100.757823	100.025141
100.628007	99.6054207	100.926403	99.7023574
100.525804	100.135559	100.080743	100.279691
100.563512	100.758945	100.152232	100.218312
100.680048	101.144513	100.817496	100.016728
99.9908989	99.8065176	100.772593	99.8676437
100.418652	99.8535029	99.7981345	100.287169
100.389305	100.148426	99.8999524	99.9107957
99.9141182	99.4576702	99.5230094	99.8727846
100.622206	100.821688	99.3152377	100.585961
99.9113883	99.6645499	100.178229	99.9344748
100.693015	100.20582	100.530751	100.812159
100.254512	100.149582	100.247354	99.8382006
99.9716184	99.4586822	99.707936	100.107239
100.700181	100.729308	100.756838	100.420519
100.715196	101.155211	100.655217	100.134501
100.468645	100.831953	99.806209	99.8883629
100.015639	99.9863627	100.479941	99.8640607
99.4199922	99.0093585	100.210133	99.660919
99.7489546	99.108389	99.9182678	100.159582
100.662303	100.576642	100.005906	100.474109
100.17995	100.853205	100.634932	99.4560638
99.4684494	99.6570322	100.070305	100.235293
100.146508	100.638229	100.43878	100.222363
100.15265	100.375979	100.065579	99.7129507
99.8465513	100.19917	100.463791	99.4741347

99.9552385	99.4422012	100.6306	99.6931662
100.071604	100.436265	100.406679	100.119857
99.6890657	100.026409	100.463201	100.203513
99.4711793	100.213193	100.111466	100.314275
99.9388587	100.884865	100.561868	100.15413
99.5542934	100.615254	100.440493	100.513904
99.552421	100.644112	100.46649	100.351525
98.7622806	99.0062443	100.35896	99.9583316
99.0662855	99.2157575	100.408983	100.64082
99.1965004	99.7088062	100.429662	100.400052
99.1182012	99.9595871	100.78258	100.116667
99.1238183	100.30892	100.585639	100.424471
99.5132715	100.805143	100.344189	100.121022
99.5113991	100.255676	100.417057	99.7471147
99.468675	100.0958	100.602576	100.14684
99.6296989	100.491595	100.560824	100.128021
99.4851859	100.080505	99.9310071	100.294754
99.5599105	99.9236582	99.8026016	99.9835283
99.5747193	99.5105478	100.220116	100.109356
100.103919	100.555517	101.068932	100.444379
99.4456959	99.6868737	101.052192	100.521836
99.302034	99.8010093	100.60553	100.47502
99.7090193	100.414975	100.259308	100.244672
100.170303	100.795043	100.309331	100.301442
100.061195	100.686246	100.126766	100.15104
99.9607681	100.630982	100.182698	99.9192922
99.9641724	100.374429	100.341235	100.128332
99.9258739	100.285833	100.220707	100.263025
99.7743821	99.7959591	100.434782	100.06534
99.8278297	99.6404114	100.523209	100.19459
99.994641	100.009224	100.404453	99.8656326
99.8160849	99.7541141	100.164382	99.9387341
99.6940403	99.8920581	100.266595	99.9978376
99.6215285	99.5886102	100.335721	99.7824212
99.6925084	99.3335005	100.222086	99.7552025
99.9023841	100.04674	100.079698	99.8972062
99.986811	100.025529	100.143506	100.186968
99.7587222	99.6483475	100.220313	100.225697
99.8317447	99.721504	100.203967	100.151351
100.000769	99.9806539	100.222086	100.225852
99.8458726	99.7744594	100.272897	100.153217
99.9881728	99.6402671	100.038931	100.114956
99.7051044	99.6160259	99.6905422	100.116511
99.5667191	99.6789376	99.9209631	100.108268
99.8039996	99.9943617	100.154535	100.069695
100.105792	100.005328	99.9069803	100.091314

99.8884265	100.256109	100.05961	100.080893
99.9226398	100.298675	100.037158	100.012458
100.137282	99.7971134	100.185258	100.048698
100.078557	99.8230861	100.166745	100.202833
100.12843	99.7981235	100.067684	99.9429336
100.16775	99.9154335	99.8752728	99.8726316
100.10426	99.7168866	99.9343551	100.152595
99.9588957	99.4749075	100.131493	100.094425
100.205708	99.8349181	99.8431714	99.849768
100.15992	99.6763403	100.060397	100.031744
100.463925	100.219603	100.262065	100.119933
100.403669	100.230713	100.033219	100.033922
100.176601	100.005905	99.8675921	100.155706
100.274135	99.8025965	100.42336	99.9857058
100.27822	99.9405405	100.033023	100.106401
100.176091	100.228549	99.9538523	100.23114
100.185623	100.009224	100.201998	100.153528
100.334221	100.053955	99.9503073	100.059896
100.05745	100.237207	100.003284	99.9962822
99.6908062	100.071414	99.9467624	99.9533545
99.82017	99.6819677	100.050353	99.8930068
99.6038261	99.5443123	99.8671982	99.9857058
99.6996575	99.6508005	99.8719248	99.9872612
99.7931058	99.5232456	99.9292347	99.8040406
99.8814476	99.5458996	99.6732114	99.9035832
99.8925116	99.6080898	100.011753	100.025989
99.8245956	99.4547065	99.9477471	100.287444
99.5410166	99.6023181	99.7494275	99.8709208
99.6911467	99.6961084	99.6722267	100.017124
99.6848487	99.6304552	99.6893605	100.033766
99.8348086	99.7300172	99.7468673	99.8307927
100.114132	99.8161601	99.8217049	99.9158705
100.005194	99.8598808	99.6919208	99.9530434
99.7927653	99.6757631	99.6373681	99.6705913
100.383754	100.502273	99.3262014	99.9902163
100.275667	100.045297	99.6355956	99.9261358
99.997024	100.123071	99.6501693	99.583336
100.409797	100.879598	99.3951307	99.7706005
100.375924	100.334316	99.5503202	99.7150744
100.276007	100.403865	99.5708021	99.7618905
100.342051	100.232733	99.54776	99.7933087
100.252688	100.030724	99.5723776	99.9278467
100.3412	99.9201952	99.6732114	99.7494477
100.387158	99.9927745	99.6649399	99.8860077
100.315157	100.046307	99.5459875	99.6203534
100.298476	99.7829727	99.731309	99.7962638

100.382222	100.209935	99.5741501	100.107645
100.344774	100.582211	99.2785416	99.8692099
100.659162	100.595341	99.3393964	100.242183
100.353285	100.249183	99.6927085	99.7046535
100.146643	99.5370977	100.180925	99.708853
100.279412	99.9300071	99.5995554	99.9483773
100.40469	100.236197	99.8303703	99.543519
100.656439	100.346725	100.065321	99.5782033
100.442478	100.097675	99.6513509	99.6441503
100.48316	99.860458	99.7994505	99.625175
100.495585	100.382365	99.9321888	99.9202254
100.298646	100.418871	99.9589727	99.9783956
100.412828	100.189797	100.152604	99.7732631
100.153278	100.006412	99.9333438	99.744791
100.283138	100.047742	99.8096283	99.7522591
100.441459	99.9986081	99.885867	99.9490745
100.293704	100.035892	99.8143563	100.060785
100.086814	99.833142	99.6998998	99.9764575
100.288251	99.9595899	100.020614	100.060007
100.414191	100.125345	100.018053	100.207968
100.184124	99.7514928	99.8356322	100.089879
100.149358	99.6432534	99.8417392	100.18323
100.212073	99.923751	99.7253127	100.169072
100.098402	99.7513483	99.6116441	99.7843097
99.9886518	99.4295203	99.9630907	99.8949308
100.417088	100.019996	99.3338753	99.9294707
99.2103423	99.0883278	100.210128	98.5124
99.5447069	99.1744569	100.338177	101.014056
100.872622	101.605725	100.469182	100.143245
100.772585	100.325783	100.676425	99.8288071
100.903638	100.420583	100.816294	99.9927939
101.093998	100.820736	100.72134	99.8493443
100.890516	100.485613	100.295231	99.8585238
99.6776348	99.3685363	98.9107211	99.5143692
100.086132	99.595131	98.3362715	99.3741869
99.8414087	98.7461237	99.9654546	99.1202717
99.3340675	98.2531936	99.8161293	99.5929398
99.2347124	98.3737165	99.5598334	99.8115371
99.5862895	99.0589919	99.4700017	99.9677447
100.104026	99.9382022	100.324387	99.8152711
99.7096738	99.3527845	99.633708	99.3993917
99.5946401	98.81679	99.7302376	99.8222725
99.8134597	99.3639119	100.355513	99.9153125
100.294556	100.281851	100.629736	99.6956261
100.667777	100.574777	100.168758	100.191165
99.7423945	99.5523554	99.5590454	99.3449368

99.459156	98.6873073	100.017462	99.577848
100.574557	100.069997	100.327145	100.074632
100.372097	100.137773	100.205006	99.3729422
99.3655952	98.9289312	99.0342396	99.5317948
100.191111	100.199335	100.260165	100.617158
100.40959	100.647755	100.200278	99.5517097
99.7275679	99.3396339	99.5962782	99.3654741
100.372268	99.9234619	99.6015972	100.340994
100.927668	100.857153	100.049179	100.099059
100.231842	100.280551	100.023569	99.442022
99.8720843	99.5195512	98.7738067	100.140133
99.7132526	99.389057	99.2387247	99.8043802
100.36903	99.680682	100.168364	100.079766
99.8584507	99.3783631	100.759361	99.8098257
99.2193746	98.9355787	99.7530895	99.6164331
99.1423446	98.6214099	99.6898528	99.9654109
100.116126	99.8895017	99.9552107	100.513071
99.8497593	99.4303874	100.1463	99.1442318
100.220935	99.5416616	99.888428	99.56509
100.338525	100.18604	99.8374052	100.521317
99.6266791	99.9910935	99.5499834	99.9453404
99.5907204	99.8736053	99.9723496	99.8748603
99.8673126	100.670588	100.6518	100.231151
99.5089187	99.8000488	99.7838214	99.9058218
99.2601051	99.5153604	99.396718	100.217304
99.7285904	100.778539	100.025933	100.258222
99.4894908	100.607292	100.282426	99.7121181
99.323331	100.541973	100.218402	100.054561
99.0216871	100.559603	99.7830334	100.359353
99.5003977	100.861055	100.47647	99.9657221
99.5508421	100.558447	100.407127	100.005085
100.784344	101.606447	100.255043	100.626493
100.114422	100.54573	100.931539	100.218237
100.309553	100.464659	100.275531	100.210147
100.380278	101.369303	100.153786	100.218704
100.049321	100.789811	99.9039909	100.185564
100.277344	101.058169	100.213477	100.124108
100.314836	101.218577	100.51587	100.178874
99.9290047	100.858598	100.192004	100.405562
100.043698	100.539805	100.610036	100.038692
100.280241	100.797903	100.206976	100.622603
99.9966616	100.411623	100.308824	100.551034
100.285183	100.316679	100.024948	100.534542
100.090393	99.8893571	100.076168	100.590397
99.9860955	99.8546743	100.448497	100.133443
100.033643	99.7883433	99.877199	100.293851

100.1543	99.7637763	99.8167203	99.8358084
100.323528	99.7402209	99.5476194	99.9910825
100.194349	99.6922429	99.6713349	100.815218
99.8402157	99.498886	100.552315	100.04087
100.293022	99.6717222	99.8431182	100.615135
100.59194	100.294857	99.6973388	100.631783
100.185658	100.321881	99.8492251	99.6470834
99.9995587	100.051066	100.202839	100.097036
99.751938	99.7562617	100.14039	100.312677
99.5406169	99.6500455	99.8661671	99.9677447
99.4110974	100.04832	99.7690465	100.423298
99.6285537	100.302372	100.360438	99.8832619
99.7354073	99.8536627	100.192595	99.9056662
99.8158456	100.175491	100.265681	99.9935719
99.9763815	100.385611	99.7917014	100.03169
99.9676901	100.863801	99.7247217	100.013954
99.9685422	100.602812	100.375016	99.9590319
100.057161	99.97881	100.549163	100.294007
100.086984	99.9283753	99.7985963	99.9468963
100.068749	100.47463	100.352361	99.8421874
100.006716	100.23185	99.2296627	100.073854
100.032762	99.8350543	99.646833	100.034847
99.843024	99.9106605	100.040201	100.2986
100.553007	100.862459	99.3507239	100.287546
99.9012082	100.026166	99.5336264	100.288791
99.8826097	100.172607	100.052408	100.210008
100.123878	100.72021	99.749605	99.7628411
100.291776	100.602825	99.706488	100.135895
100.42606	100.704019	99.9667648	100.380809
100.153055	99.7150676	100.156164	100.049015
99.7060096	99.2841272	100.234917	99.8120419
100.558126	100.579117	99.9955094	100.365395
100.755543	100.496716	100.207353	100.16828
100.85536	100.666577	99.577334	100.120948
100.646341	100.614968	100.028585	100.248154
100.211751	99.9343687	100.08824	100.024103
100.418894	100.030069	99.7486206	100.114564
100.45046	99.5469416	99.7970533	100.20487
100.757591	100.431085	99.7688993	100.330986
100.566146	99.7328488	99.6064724	99.9654051
100.446877	99.8330305	100.155968	100.091365
100.535774	99.7697122	99.9994471	100.110205
100.633715	100.133286	99.9870435	100.370844
100.655726	99.9443435	100.016773	100.091521
100.736774	100.572756	99.8490299	100.240369
100.655896	100.355768	99.736217	100.061938

100.46138	100.288546	99.7066849	100.222619
100.419064	100.208748	100.049652	100.023792
100.406438	100.189088	100.298313	100.096815
100.318564	99.8768329	100.226648	100.523118
100.463257	99.9793276	99.9998408	100.221685
100.544817	99.5719509	100.067962	100.148662
100.865769	100.096423	100.072884	100.688625
100.511204	100.262959	100.053786	100.619184
100.246048	100.13054	100.103007	100.449784
100.244341	100.163934	99.9069129	100.072993
100.613069	100.463756	100.127026	100.485127
100.529973	100.379332	100.032129	100.151154
100.422818	100.006505	100.026617	99.9921853
100.624159	100.478357	99.9275855	100.290504
100.38255	100.059126	100.027601	100.124529
100.553861	100.595452	100.168765	100.341417
100.351496	100.109289	100.182546	100.141189
100.351837	100.100326	99.8214666	100.198019
100.310204	100.357503	99.7964627	100.143213
100.303208	100.152369	99.9285699	100.107402
100.132409	100.135455	99.8466673	100.436082
99.9361869	100.009397	100.035673	100.15925
99.9093983	99.96863	99.9116381	100.013205
99.9796971	99.9207798	99.8407609	100.292372
99.8848279	99.9702202	99.8291449	100.2863
100.067229	99.8486432	99.9525894	100.061004
99.9024026	99.9469456	100.253227	100.135895
100.051702	100.052621	100.20755	99.9615126
99.9425002	100.025443	100.035476	100.054776
99.7322863	99.9262732	100.089815	100.076418
99.8167473	100.086882	99.9352638	100.006821
99.7718721	99.9754244	99.7074724	99.9976347
99.9597336	100.187497	99.819104	99.917917
99.7350164	100.095267	99.910063	99.6991603
99.7519086	100.176944	100.071112	100.054776
99.7121522	100.05291	100.198297	100.017564
99.8387583	99.9550411	100.042761	100.016319
99.9614399	100.062596	99.9773964	99.9031256
99.7645351	99.8042626	99.9508174	99.7538106
100.033445	100.247057	100.000038	99.8515893
100.028838	100.29245	100.079775	99.9612012
99.9134934	100.374417	99.7216478	99.9759926
99.9056445	100.239974	99.9460923	99.6929324
100.080197	100.329313	100.100644	99.6915311
100.043853	100.154537	100.289256	99.6135261
99.8848279	100.073871	99.9838935	99.7573916

99.7836454	100.097724	99.7192853	99.6224009
99.683828	99.9885796	100.048864	99.656966
99.8097515	100.114927	99.9732619	99.7435345
99.7928593	99.9638594	100.084303	100.065208
99.5990258	99.7308249	99.8167414	99.9431402
99.4137237	99.3815622	99.9651897	99.5651038
99.6377584	99.3428195	100.06934	99.8825734
99.5744554	99.665049	99.8427297	99.8884899
99.5689953	99.4075835	99.7249948	99.8531463
99.7875698	99.5356657	100.276656	99.9235221
99.5386235	99.4074389	100.340839	99.4534678
99.4120174	99.447049	100.074459	99.5859674
99.5161006	99.6080914	99.8685211	99.4425689
99.4616702	99.4675768	99.7915406	99.6300301
99.3176601	99.7514973	100.214441	99.7902441
99.4055336	99.6170543	99.9657804	99.1995233
99.4290802	99.6147413	99.9569207	99.3626955
99.3429131	99.6890464	100.22212	99.4450601
99.3778918	99.9621247	99.7562989	99.9853345
99.5771854	100.389306	100.046305	100.08903
99.226886	99.8304284	100.483184	99.7321685
99.178257	99.7212838	100.114622	99.9348881
99.2099938	99.6104044	100.116985	99.9803522
99.2231321	99.5310397	100.117182	99.923055
99.2035099	99.6540623	99.9974782	99.8279231
99.1572697	99.7036472	99.8413515	99.9387806
99.1813283	99.6173434	100.12427	99.7583258
99.2886533	99.8460411	100.385137	99.7318571
99.17365	99.9723886	100.173687	99.7278089
99.4845566	100.034969	99.6285758	100.101145
99.5256946	100.062561	99.6414458	100.116806
99.4895531	100.120069	99.6355215	100.143478
99.5191991	100.3191	99.725611	100.045166
99.4201016	100.149365	100.280039	99.9032798
99.4647371	100.174012	100.352356	99.7843438
99.7177272	100.439386	100.200981	100.04315
99.7906763	100.310884	100.416502	99.9789522
99.9030978	100.429466	100.119472	99.9391001
99.9175877	100.014353	99.8179477	99.8573801
99.5703301	99.7035613	99.4882323	99.9071564
99.6732582	99.9525048	99.5290892	99.9056058
99.9067619	100.005208	99.5728061	99.8780039
100.010523	100.156961	99.4878237	99.9180111
99.948566	99.9742059	99.6637128	99.9439072
100.016019	100.367462	99.2185767	100.081296
100.07681	100.550217	99.1785369	99.9603442

100.027344	100.632527	99.4065185	99.7590679
100.082639	100.500769	99.6590142	99.8355157
99.9595584	100.471008	99.8884258	99.8770735
100.108288	100.512395	100.050424	99.8697854
100.165415	100.547272	100.094345	99.9696482
99.8712867	100.396139	99.5004894	99.9811231
99.4146054	100.127199	99.4144856	100.07928
99.0781736	99.993427	99.4624925	100.15976
98.9327751	99.7818405	99.5869018	100.007795
99.3894563	99.8148573	100.047564	100.085948
99.6416136	99.920108	100.38259	100.144718
99.8542985	100.176957	100.510064	100.264585
99.9125912	100.343436	100.511494	100.121303
100.084804	100.382963	100.562361	100.156813
100.1864	100.330725	100.385859	99.9839143
100.142098	100.543862	100.287394	100.077109
100.222875	100.491004	100.174629	100.192944
99.9459012	100.588969	99.8575789	100.193409
100.049662	100.310109	99.6271458	100.237603
100.044832	99.9518847	99.4432897	100.197286
99.6670959	99.5170862	99.3701558	100.30118
99.4527455	99.7948612	99.8279576	99.9766262
99.6629321	100.183002	100.011814	99.7677517
99.7105655	99.752389	100.22713	99.9116534
99.6004757	99.8294281	100.286577	100.133398
99.6484422	99.8577947	100.186068	100.020355
99.7851801	99.8509743	100.064519	100.015548
100.166414	100.224079	100.239591	99.9791073
100.174742	100.204393	100.275749	100.040359
100.071314	100.091237	100.267987	99.9687178
99.7978379	100.021018	99.91151	99.9446825
99.6654303	99.9287885	99.9413355	99.9211124
99.515535	99.9492496	100.113547	99.7300705
99.8086637	100.056205	100.157264	99.7911667
99.884111	99.9354539	100.126622	99.9769363
99.8368107	99.8213677	99.8976186	100.153092
99.915256	99.8184225	100.201594	100.092771
99.9059291	99.678915	100.21855	99.9842245
99.8712867	99.8660101	100.13847	99.8719563
99.9970322	99.7663397	100.166253	99.9265397
100.064152	99.7385932	100.102516	99.871181
100.129773	99.7939312	100.343163	99.8913396
100.144929	99.7734701	100.009158	100.134019
100.00436	99.7613794	99.6733142	100.214653
99.9928685	99.8475641	99.9615597	99.9984906
100.013521	99.9500246	100.089238	99.9180111

100.505344	100.053415	100.096388	99.8914947
100.404748	100.272132	99.9627854	99.9321221
100.568966	100.544017	100.10701	99.8088443
100.828785	100.796525	100.3926	99.6229196
100.419071	100.50511	100.20282	99.8531933
100.281667	100.14642	100.139083	99.9325873
100.145762	99.7978064	99.9672797	99.8793995
100.141765	99.6641892	100.076368	99.8888586
100.010356	99.8802709	100.08127	99.9991108
99.9827089	99.5646738	100.178306	100.076489
100.1386	99.6195468	100.124579	100.135104
100.490688	100.003038	100.064111	100.132468
100.735017	100.33336	100.333358	100.075869
100.485025	100.159751	100.151953	99.9881011
100.196393	99.9556049	100.027952	99.8721114
99.9487326	99.5887001	99.945217	99.798765
100.027511	99.8108271	99.9787196	99.9925981
99.9197528	99.8681802	100.147254	100.034776
99.9757138	100.015748	100.118246	100.034776
100.040002	100.177887	99.9725911	100.03214
100.133604	99.7843207	100.136632	100.194184
100.180737	99.512901	99.954614	100.265205
100.328301	99.698601	99.8189691	100.256211
100.378932	99.9904818	99.7321481	100.067495
100.314644	99.8215227	99.9852567	99.8686999
99.9259152	99.5276268	100.284329	99.7423208
99.8857765	99.5284018	100.189541	99.8052778
99.8572964	99.6782949	100.13418	99.8643581
100.095297	99.8798058	100.167683	99.7243331
100.029176	100.032024	100.290458	99.7675966
100.086303	99.8114471	100.487797	99.955072
100.265678	99.5235966	100.579725	99.9935284
100.280668	99.5651388	99.9613554	100.215894
100.288495	99.5153811	99.6240816	100.334365
100.164915	99.4518277	99.6114159	100.343979
100.123611	99.5652938	99.6490043	100.294357
100.280668	99.6224919	99.968914	100.182865
100.535125	100.022583	100.227637	100.19416
100.49335	100.071243	100.29748	100.114209
100.47831	100.199968	100.674882	100.179878
100.191728	100.028646	100.722465	100.135943
100.201922	100.05694	100.357317	100.161869
100.173347	100.101559	100.288699	100.066859
100.300178	100.285007	100.028929	100.070895
100.290988	100.562823	100.179645	100.015628
100.410801	100.095651	100.152483	100.0518

100.414978	100.109798	99.9115018	100.060494
100.722615	100.241166	100.381824	100.170873
101.026075	100.677712	100.655889	99.9920307
100.946868	100.914796	99.8769884	100.011436
100.950711	100.745961	99.7164703	99.9305535
100.846272	100.547121	99.4164688	100.013765
100.722949	100.187842	99.6543869	100.048695
100.425004	99.8674285	100.326684	100.217913
100.38891	99.9669261	99.9427477	100.100237
100.254058	100.136849	100.065281	100.033171
100.471292	99.9645941	100.164328	100.08347
100.564034	100.277545	100.164328	100.127094
100.772412	100.312836	100.898096	99.9122346
100.809342	100.195771	100.915659	99.9468543
100.82906	100.582723	100.561539	100.068722
100.665967	100.561424	100.216813	100.268523
100.219635	100.049167	100.077534	100.336365
99.92052	100.076373	99.9010865	100.333571
99.8145766	99.6788495	99.901495	100.291033
99.5196394	99.2917417	99.9415224	100.188882
99.8941177	99.4865393	99.909868	100.046056
99.9109951	99.5959867	99.9842047	100.052732
100.036322	100.052121	99.7995884	99.9629998
100.086955	100.062693	100.034239	99.8318174
100.134245	99.8350918	100.408169	99.9057143
100.306863	100.046524	100.45228	99.9966881
100.303019	99.8528148	100.359564	100.036586
99.951267	99.818457	100.119604	100.053818
99.7873387	99.5409521	100.184954	100.129268
99.7801533	99.4277736	100.034443	100.20689
99.6601732	99.3413351	99.8808686	100.224278
99.7662837	99.7721285	100.00238	100.057079
99.6700323	99.7514516	100.089991	100.10831
99.6770506	99.7369934	100.25051	100.192453
99.860697	99.6726309	99.9652121	100.280787
99.8657101	100.042482	99.8040813	100.210772
99.9008018	100.002528	100.199454	100.031929
99.9016373	99.9075385	100.596869	99.8824275
99.5863136	99.6757402	100.255002	99.9443704
99.5634205	99.588058	100.104491	99.8873953
99.5328406	99.6236594	100.281347	99.7958005
99.6944294	99.8745799	100.153913	99.8745099
99.975664	100.131563	99.8500311	99.9170472
99.9955493	99.9723674	99.6043526	100.157833
100.247374	99.8612099	99.9409097	100.242442
100.172512	99.8447306	100.174948	100.067635

99.897961	99.6690552	99.7887647	99.9075772
99.8949532	99.4521816	99.9627614	100.053353
100.006244	99.6650131	99.9255931	99.8470315
100.054704	99.7135182	99.7011536	99.8456343
99.8754021	99.6376513	99.7573146	100.094648
99.6075357	99.7993349	99.7332164	100.037052
99.802378	99.9219967	99.919058	100.074
100.110683	100.07202	100.270115	99.8605379
100.34797	100.201367	100.548469	99.8718708
100.448399	100.42959	100.140434	99.9620684
100.261745	100.198413	99.5710645	99.9010569
100.044845	99.947493	99.4362783	100.022148
100.105336	99.9807625	99.5553395	99.872647
100.001899	100.23417	99.636007	99.6584083
99.7976991	100.266818	99.696865	99.8575882
99.6842361	100.183644	99.8390032	99.8977968
99.7741376	100.169497	100.22539	99.9134765
100.019946	100.047457	100.476991	99.9394025
99.8479972	100.129542	100.406943	100.081763
99.567598	99.8358691	99.7673214	100.289015
99.7933545	99.9583755	99.2220499	100.262158
99.7729679	100.011078	99.5095938	100.221173
99.7799862	100.044348	99.6423378	100.151002
99.8936164	99.9756321	99.4375036	100.088438
100.108344	100.173539	100.002585	100.008332
100.534123	100.423993	100.240298	99.7667696
100.086119	100.166388	99.8455383	99.7729794
100.058881	100.219868	99.7959124	99.7374282
99.7453958	100.339265	100.122258	99.6834028
99.6123817	100.298377	100.132061	99.8204845
99.6414577	100.183489	100.067323	99.7812074
99.6324341	100.122547	100.153504	99.8437713
99.7851664	100.069533	100.202109	99.9015226
99.418542	99.6550634	99.5665717	100.038139
99.3892989	99.9597747	99.4705875	100.078347
99.3341549	99.7262663	99.5822967	100.008487
99.2561177	99.7683973	99.7389346	99.9555481
99.4665006	99.8731807	99.8949599	99.8858429
99.7562575	100.138093	100.344247	99.7582311
99.9079872	100.078861	100.151666	99.6386921
99.6085383	99.8027551	99.7742649	99.7869515
99.3916384	99.7651326	99.565959	99.8937604
99.2751675	99.8601217	99.6907383	99.7500031
99.0512492	99.9824726	99.9956411	99.8083754
99.0633972	99.5910858	99.8317121	99.6409418
99.2291795	99.4298339	99.9526439	99.7528563

99.1953225	99.1728821	99.8278374	99.7932137
99.5620782	99.4174655	99.6063671	99.8034583
99.8706267	99.7789296	99.3940738	100.020302
100.132809	99.9315237	99.3781671	99.9752881
100.272907	100.092003	99.8039774	99.821464
100.357633	100.088447	99.7250556	99.6280585
100.125471	99.7820217	99.5427403	99.8047001
100.077771	99.5406849	99.4935927	99.9535572
100.168334	99.9476026	99.5019539	99.7436982
99.8991465	99.9700201	99.7487118	99.7545637
99.466178	99.7064204	99.8557761	99.7921272
99.4678458	99.543004	99.7177141	99.8447472
99.1030915	99.0921788	99.8963586	100.043275
99.1057601	98.9782357	99.7383112	100.252824
99.3612715	99.2393618	99.6544951	100.357598
99.5900977	99.4568895	99.7246478	100.301563
99.9176594	99.7835677	99.6069789	100.103346
99.7785625	99.7640876	99.7648224	100.016577
100.210364	100.066184	99.7138394	100.119799
100.188181	99.9650728	99.9991404	99.815876
99.7608835	99.6988448	99.8506778	99.8639946
99.8254285	99.3502128	99.6253328	99.918322
99.7270265	99.7328576	100.010357	99.8689617
100.039244	99.8936458	100.390486	99.7410595
99.8087502	99.6974533	100.693937	99.7811065
99.616783	99.4935307	100.389467	99.9276352
99.6956713	99.5264613	100.348476	99.9318262
99.6980063	99.4848729	100.167384	99.8222401
100.090947	99.8421626	99.7189377	99.8064075
100.224373	99.9256488	99.5863818	99.8029927
99.7787292	99.7801665	99.4201771	99.8064075
99.7572142	99.6754996	99.7511589	99.9608526
99.8796329	99.920083	100.105389	99.8042344
100.240384	100.038819	100.160043	99.9167698
100.170169	99.8297943	100.327675	100.040791
99.9023154	99.6009804	100.594418	99.9735807
99.8752966	99.6220066	99.9298035	99.9974848
99.8329337	99.6680786	99.9257248	99.8906926
99.8644557	99.6795193	99.9605972	99.895194
99.9375066	99.6815291	99.6285957	99.9676823
100.107292	99.987645	99.8488424	99.9692345
100.379648	100.028151	100.221223	99.9420708
100.210364	99.9976942	100.581978	99.7904198
100.230377	99.8924089	100.135163	99.943623
100.077104	99.7956268	99.8333436	100.075095
99.7373671	99.6798285	99.9936342	100.04669

99.8908074	99.6182961	100.078062	100.191666
99.9251647	99.655401	99.7399427	100.311031
100.169668	99.9612077	99.7574808	100.294268
100.24422	100.485161	100.338891	99.865702
100.201524	100.21816	100.342154	99.7191733
100.068431	100.25913	100.068885	99.7921272
100.322108	100.512525	99.8180487	99.6306972
100.212865	100.340297	99.6024924	99.9006268
100.064929	100.094476	99.9016609	99.8399353
100.174172	99.9675465	100.24549	99.7111018
100.124971	100.19667	99.9860888	99.8354339
99.9441779	100.27258	99.9563147	99.7745872
100.101788	100.115657	99.8098914	99.7848318
99.8107516	100.029388	99.8102993	99.8992298
99.943344	99.9392539	100.327879	99.8251893
100.034908	100.031862	100.360508	99.8582514
99.9838723	99.8735473	100.031158	99.8883643
99.8029128	99.8599421	100.166773	100.1555
100.107458	100.035263	100.046453	100.179249
100.267737	100.004342	99.982418	100.192132
100.337285	100.271962	100.270778	100.271295
100.228042	100.211357	99.9565186	100.303736
100.501567	100.339678	99.7260753	100.107381
100.512241	100.454085	99.6799866	100.233266
100.475382	100.475575	100.384368	100.031168
100.105624	100.459651	99.9979168	99.9551094
99.7879023	100.523812	99.9920028	100.076027
100.043747	100.432904	100.38967	100.020147
100.141816	100.33303	100.420056	100.174437
100.043747	100.467381	100.22367	100.061902
99.8147544	100.241814	99.8045892	100.333228
99.7698898	99.9485302	99.9683467	100.259808
99.9253315	100.029543	100.346641	100.166676
99.9286671	100.054279	100.235294	100.031789
99.7306958	100.101897	100.154537	99.9135101
99.9803698	100.193577	100.094173	99.928877
100.15816	100.196979	100.161878	100.017043
100.18668	100.24707	100.515904	100.061436
100.203192	100.402911	100.000772	100.234352
100.35363	100.632962	99.681822	100.269898
100.190683	100.688929	99.8066285	100.219451
100.105123	100.626159	100.332366	100.087358
99.9953803	100.587972	100.335017	100.137805
99.9656929	100.607298	100.191041	100.27114
99.8247613	100.317106	100.019126	100.382278
99.9338374	100.268715	99.7319893	100.332607

99.9094871	100.053197	99.8451717	100.254065
100.028737	99.9689379	100.165753	100.060505
100.155158	100.098651	99.9236855	100.174592
100.356299	100.346172	100.315847	100.130354
100.352129	100.407859	100.270166	100.010523
100.560608	100.534788	100.09193	100.151154
100.407501	100.62415	100.114362	100.278745
100.170369	100.783645	100.173309	100.119798
100.448749	100.680043	100.397716	100.2023
100.57677	100.63531	100.441045	100.126622
100.505425	100.607352	99.8201425	100.294262
100.410242	100.584208	100.132024	100.295813
100.413076	100.432767	100.212958	100.322486
100.692623	100.383218	100.162068	100.30093
100.540931	100.355726	100.251177	100.004265
100.578604	100.574112	99.7845806	99.9760403
99.9958397	100.491479	99.9744483	99.9611528
99.9141594	100.409624	100.61681	99.9273456
100.025011	100.458861	100.27468	100.05327
100.164034	100.705206	99.9885505	100.004265
100.158367	100.63034	100.141834	99.9485914
100.173869	100.492722	100.490096	99.8981908
100.47642	100.448455	100.267118	100.194236
100.325561	100.191703	100.100754	100.187413
100.282221	99.617469	99.7226539	100.427785
99.8468149	99.4251774	99.7490187	100.485319
99.6299454	99.4217602	99.8336315	100.168183
99.5742694	99.3386617	99.8540693	100.154691
99.7169599	99.5536307	99.9895723	100.070173
99.7526325	99.3944232	100.066827	100.144456
99.8561498	99.7474755	100.297775	99.9777462
100.19904	100.028147	100.435526	99.9788317
100.331896	100.162658	100.264257	100.102274
100.1602	100.102392	100.222155	100.007987
99.999507	99.7960921	100.160433	100.019152
100.17537	99.6225947	99.9977475	100.200129
100.247548	99.7296132	100.073776	100.214396
100.189705	99.8487471	100.649511	100.24169
100.281887	99.826691	100.426125	100.297209
99.8716524	99.9156919	100.000404	100.235952
99.861484	99.8116245	99.9241712	100.040708
100.109692	99.9719193	99.9409302	100.119953
100.465251	100.383218	99.8434416	100.110494
100.341897	100.38881	99.9350033	99.9413027
100.282054	100.277287	100.1169	99.9383562
100.501091	100.44131	100.376665	99.9237788

100.598107	100.763453	100.051703	99.9837943
100.658284	100.624748	99.7300115	99.786534
100.424411	100.573801	99.960755	99.7649781
100.308392	100.282568	99.9010764	99.8172396
100.318394	100.236281	100.23544	99.9611528
100.216543	99.7010335	100.41284	99.9549496
99.8838211	99.3773375	100.091966	100.088938
99.9993403	99.6443401	99.7197926	100.164306
100.251716	99.9511059	100.004288	100.101034
100.145198	99.7016548	100.005309	100.058697
99.3579	99.2532333	99.834449	100.012484
99.679787	99.4708428	99.8299527	100.152055
99.8226442	99.4904136	100.288783	100.169889
100.126528	99.7238662	100.688138	100.204937
99.9458314	99.6792881	100.032083	100.119023
100.198374	100.137495	99.416086	100.034815
100.107525	100.312235	99.5924647	99.7642027
99.7494653	100.062473	99.9674995	99.5733007
99.6259447	100.011838	100.358884	99.7545878
99.7768032	100.02473	100.173513	99.914474
100.015843	100.166696	100.075616	99.88687
99.8913223	100.122118	100.018185	99.772732
99.7966399	100.146349	99.8078798	99.7815715
99.8779867	100.124914	99.8642883	99.8484105
99.845648	99.85496	99.9805797	99.9003619
99.9209939	99.9456695	99.7343035	100.007366
100.106191	100.118856	100.320257	99.9779013
100.111192	99.9526591	100.609044	100.030008
100.097023	99.9466014	100.382388	100.161204
99.7137927	99.6535043	99.9462441	100.237813
99.4687518	99.4674256	99.7453399	99.9847247
99.5579334	99.5627948	100.175966	99.8812872
99.835813	99.7296132	100.093397	99.8220471
99.8024742	99.8822971	100.097075	99.7914966
99.5719357	99.5165081	100.118126	99.8865599
99.6526158	99.566212	100.00858	99.7918067
99.5869382	99.4897923	99.9977475	99.8936935
99.8586502	99.8235845	99.7287853	99.9924787
100.244048	100.365511	99.9364339	99.7103904
100.226712	100.251659	100.109543	99.7457483
100.440914	100.17353	99.9957037	99.7852934
100.302391	100.388033	99.6689023	99.6733265
100.417743	100.240009	99.4937499	99.8685707
100.177037	100.085461	99.7042598	99.9053244
99.6312789	99.8686286	99.9149742	99.8842337
99.663451	99.9057511	100.110769	99.8341432

99.9925058	100.083908	99.9282588	99.8479452
100.21871	100.313322	99.3948306	99.8056087
100.329895	100.634533	99.1360873	99.7576894
99.8559831	100.563395	99.3823635	99.642776
99.7142928	100.533106	99.613107	99.6184286
99.8433143	100.308507	99.9155873	99.7286897
99.8428142	100.108449	99.8702152	100.018532
100.027012	100.335223	99.8988282	100.150349
100.175536	100.323729	100.107294	100.180279
99.9508322	100.027215	99.9826235	100.200749
99.5972733	99.5806571	99.7473837	100.396459
99.1426975	99.4818708	99.8530474	100.413363
99.0958563	99.5728909	99.8242301	100.206332
99.1815373	99.4410205	99.8097192	100.169424
99.2987235	99.3734544	99.6705374	100.170354
